# Plastid genomes reveal evolutionary shifts in elevational range and flowering time of *Osmanthus* (Oleaceae)

**DOI:** 10.1002/ece3.8777

**Published:** 2022-04-01

**Authors:** Yongfu Li, Xuan Li, Steven Paul Sylvester, Min Zhang, Xianrong Wang, Yifan Duan

**Affiliations:** ^1^ 74584 Co‐Innovation Center for Sustainable Forestry in Southern China College of Biology and the Environment International Cultivar Registration Center for Osmanthus Nanjing Forestry University Nanjing China; ^2^ Department of Botany and Biodiversity Research Centre University of British Columbia Vancouver British Columbia Canada

**Keywords:** ancestral state reconstruction, ancestral trait reconstruction, molecular clock, plastomes, purification selections

## Abstract

Species of *Osmanthus* are economically important ornamental trees, yet information regarding their plastid genomes (plastomes) have rarely been reported, thus hindering taxonomic and evolutionary studies of this small but enigmatic genus. Here, we performed comparative genomics and evolutionary analyses on plastomes of 16 of the 28 currently accepted species, with 11 plastomes newly sequenced. Phylogenetic studies identified four main lineages within the genus that are here designated the: “Caucasian *Osmanthus*” (corresponding to *O*. *decorus*), “*Siphosmanthus*” (corresponding to *O*. sect. *Siphosmanthus*), “*O*. *serrulatus* + *O. yunnanensis*,” and “Core *Osmanthus*: (corresponding to *O*. sect. *Osmanthus* + *O*. sect. *Linocieroides*). Molecular clock analysis suggested that *Osmanthus* split from its sister clade c. 15.83 Ma. The estimated crown ages of the lineages were the following: genus *Osmanthus* at 12.66 Ma; “*Siphosmanthus*” clade at 5.85 Ma; “*O*. *serrulatus* + *O. yunnanensis*” at 4.89 Ma; and “Core *Osmanthus*: clade at 6.2 Ma. Ancestral state reconstructions and trait mapping showed that ancestors of *Osmanthus* were spring flowering and originated at lower elevations. Phylogenetic principal component analysis clearly distinguished spring‐flowering species from autumn‐flowering species, suggesting that flowering time differentiation is related to the difference in ecological niches. Nucleotide substitution rates of 80 common genes showed slow evolutionary pace and low nucleotide variations, all genes being subjected to purifying selection.

## Graphical abstract

This is the first well‐sampled report on the plastomes of *Osmanthus*. Phylogenetic results showed that *Osmanthus* species were clustered into four lineages, which had a good correspondence with morphological and biogeographic evidence. Molecular dating results suggested that *Osmanthus* is a tertiary relict genus. The origin and diversification of *Osmanthus* species are speculated to be closely related to the climatic changes and the orogenesis of the Qinghai–Tibet Plateau during the Miocene. The diversity of flowering time of *Osmanthus* is related to niche differences. The plastid genes are likely to not be significantly involved in adaptation to altitude or precipitation.

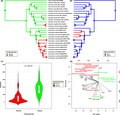

## 1 INTRODUCTION

Attractive fragrance and elegant flowers make species of *Osmanthus* (Oleaceae) widely used in horticulture, food, and medicine throughout temperate to subtropical regions of the northern hemisphere (Xiang & Liu, 2008). Except for *Osmanthus decorus* (Boiss. & Balansa), Kasapligil, which is found in the Caucasus Mountains (Green, 1972), the other 27 currently accepted species are found in eastern Asia, from the high‐elevation Hengduan Mountains to the low elevations of southeast China, Japan, and Korea (Chang et al., 1996; Green, 1958; POWO, 2021). *Osmanthus* is characterized by its cymose inflorescences, corolla lobes united in pairs at their base and usually forming a tube, and an androdioecious breeding system (Chang et al., 1996; Duan, Li, Zheng, et al., 2019; Green, 1958, 1972; Li et al., 2020). Within the genus, previous taxonomic studies divided the 28 species into three sections: *O*. sect. *Osmanthus*; *O*. sect. *Siphosmanthus* Franch., and *O*. sect. *Linocieroides* P.S. Green based on floral characteristics (Chang et al., 1996; Green, 1958; Li et al., 2020).

Genetic studies have discussed the delimitation of *Osmanthus* using a number of gene fragments (Guo et al., 2011; Li et al., 2020; Lu et al., 2011; Yuan et al., 2010) or a few plastomes (Duan, Li, Zhang, et al., 2019; Olofsson et al., 2019; Zhao, Yang, et al., 2020; Zhao, Ren, et al., 2020). They found that *Osmanthus* lies in a generic complex in the subtribe Oleinae (Oleaceae), very close to four genera: *Nestegis* Raf., *Notelaea* Vent., *Phillyrea* L., and *Picconia* DC. (referred to as NNPP here). Within the genus, only sect. *Siphosmanthus* and sect. *Osmanthus* were supported (Guo et al., 2011; Li et al., 2020), although detailed studies on infra‐generic relationships, as well as the divergence time of lineages, are still lacking. Therefore, a more widely sampled and robust phylogenetic study is urgently needed to guide downstream evolutionary analysis such as molecular dating and morphological trait evolution. In particular, plastomes have been successfully used to construct the phylogenetic framework of Oleaceae (Dupin et al., 2020; Ha et al., 2018; Olofsson et al., 2019), laying the foundation for our follow‐up evolutionary analysis.

Flowering time is considered as a crucial life cycle trait that contributes to fitness in plants (Gaudinier & Blackman, 2020). Changes in flowering time between species may reflect the environmental constraints which limit the direction of evolution in certain species (Wadgymar et al., 2018). The variations in light, temperature, and precipitation caused by changes in elevation are also considered one of the main reasons for the high species richness in eastern Asia (Shimono et al., 2010; Sun et al., 2017). In *Osmanthus*, significant variations in elevational range and flowering time between species have been noted by previous morphological and ecological surveys (Li et al., 2019, 2020). For example, high‐elevation species such as *O*. *yunnanensis* (Franchet) P.S. Green and *O*. *delavayi* Franchet tended to bloom in spring (March to April), while low‐elevation species such as *O*. *cooperi* Hemsl tended to bloom in autumn (September to October). Exploring the evolutionary patterns of flowering time and elevational shifts within these species can provide insights into how *Osmanthus* species adapted to different environments (Gaudinier & Blackman, 2020).

Plastomes play an important role in plant phylogeny and evolutionary studies due to them being structurally stable, generally maternal inherited, and with low levels of recombinant DNA (Gu et al., 2018; Wu et al., 2017). Genes of plastomes primarily encode core components of the photosynthetic machinery and factors involved in their expression and assembly, as well as those of self‐replication. (Gao et al., 2018; Mohanta et al., 2020; Pilot et al., 2018). Thus, plastomes are generally considered to be conserved, in terms of genomic structures and substitution rates, among the majority of Angiosperms. Recently, however, increasing studies have detected positive selection signals in plastid genes. For example, *psaA* genes in the 22 closely related *Oryza* species were found to be undergoing positive selection, which could be related to the adaptation of rice plants to habitats with different light conditions (Gao et al., 2019). The evolution rates of some genes (*psaA*, *rpl16*, *ndhA*, and *ndhH*) in *Rhodiola* plants were also found to be accelerating, which possibly allowed *Rhodiola* species to adapt to the harsh environment of the Qinghai–Tibet Plateau, such as low carbon dioxide concentration and high solar irradiation (Zhao, Yang, et al., 2020; Zhao, Ren, et al., 2020). Furthermore, the evolutionary rates of *matK* genes in some low‐altitude and recently derived lineages of *Dysosma* were found to be significantly accelerated, which may reflect the adaptability of these species to new environments (Ye et al., 2018). Thus, genetic content held in plastomes can provide useful information to enhance our understanding regarding adaptive evolution in plants. Herein, we assembled plastomes of 16 of the 28 *Osmanthus* species that are currently accepted (POWO, 2021), with 11 plastomes newly sequenced, which allowed us to (1) provide the most well‐sampled phylogenetic and molecular clock analyses of *Osmanthus* to date; (2) study the evolutionary patterns of elevational shifts and flowering time within *Osmanthus* based on ancestral traits reconstruction; and (3) calculate the substitution rate of plastid genes and explore whether the differentiation of flowering time and elevational range is related to the environmental pressures on plastid genes.

## 2 MATERIALS AND METHODS

### 2.1 Plastid genome sequencing, assembly, and annotation

We newly sequenced and assembled the whole plastomes of 11 species of *Osmanthus*. They are as follows: *O*. *armatus* Diels, *O*. *cooperi* Hemsl., *O*. *decorus*, *O*. *delavayi*, *O*. *enervius* Masam. & K. Mori, *O*. *fordii* Hemsl., *O*. × *fortunei* Carr., *O*. *heterophyllus* (G. Don) P.S. Green, *O*. *serrulatus* Rehder, *O*. *suavis* King ex C.B. Clarke, and *O*. *yunnanensis*. In addition, plastomes of *O*. *austrocaledonicus* (Vieill.) Knobl., *O*. *didymopetalus* P.S. Green, *O*. *fragrans* (Thunb.) Loureiro, *O*. *insularis* Koidz., and *O*. *urceolatus* P.S. Green were extracted from GenBank (https://www.ncbi.nlm.nih.gov/) that had been sequenced as part of previous publications (Duan, Li, Zheng, et al., 2019; Duan, Li, Zhang, et al., 2019; Olofsson et al., 2019; Zhao, Yang, et al., 2020; Zhao, Ren, et al., 2020). The selection of the outgroups was guided by previous research (Niu et al., 2020; Olofsson et al., 2019), and we selected 12 species: *Nestegis apetala* (Vahl) L.A.S. Johnson, *Nestegis cunninghamii* (Hook. f.) L.A.S. Johnson, *Nestegis lanceolata* (Hook. f.) L.A.S. Johnson, *Nestegis sandwicensis* (A. Gray) O. Deg., I. Deg. & L.A.S. Johnson, *Notelaea longifolia* Vent., *Notelaea microcarpa* R. Br., *Notelaea venosa* F. Muell., *Olea europaea* L., *Phillyrea angustifolia* L., *Phillyrea latifolia* L., *Picconia azorica* (Tutin) Knobl., and *Picconia excelsa* (Sol.) A. DC.

Total genomic DNA was isolated from fresh leaves of a single individual using the DNeasy Plant Mini Kit (Qiagen, Valencia, CA), and was used to prepare the shotgun library following the manufacturer's protocol for Hiseq 4000 Sequencing System (Illumina, CA, USA). The library was sequenced by Nanjing Genepioneer Biotechnologies Inc. (Nanjing, China). Raw reads were obtained and trimmed using CLC Genomics Workbench v9 (CLC Bio, Aarhus, Denmark) with default parameters. The resultant clean reads were then employed to assemble the plastome using the program NOVOPlasty (Dierckxsens et al., 2017) with *O*. *fragrans* (GenBank: MG820121) (Duan, Li, Zheng, et al., 2019; Duan, Li, Zhang, et al., 2019) as the reference. The resultant genome was annotated by PGA (Qu et al., 2019). The plastomes generated in the present study are available in the NCBI GenBank database. The accession numbers of *Osmanthus* and outgroups are presented in Table 1.

**TABLE 1:** The features of plastomes of 15 *Osmanthus* species and 13 outgroups

Species	GenBank	IRa (bp)	IRb (bp)	LSC (bp)	SSC (bp)	Total (bp)	Gene	PCGs	tRNA	rRNA	GC (%)	References
*Nestegis apetala*	NC_036983	25723	25723	85898	17505	154,849	133	87	37	8	37.8	Olofsson et al. (2019)
*Nestegis cunninghamii*	NC_042455	25723	25723	85941	17520	154,907	133	87	37	8	37.8	Olofsson et al. (2019)
*Nestegis lanceolata*	NC_042456	25706	25706	86009	17556	154,977	133	87	37	8	37.8	Olofsson et al. (2019)
*Nestegis sandwicensis*	NC_042457	25717	25717	86642	17489	155,565	133	87	37	8	37.8	Olofsson et al. (2019)
*Notelaea longifolia*	NC_042458	25713	25713	86117	17494	155,037	133	87	37	8	37.8	Olofsson et al. (2019)
*Notelaea microcarpa*	NC_042459	25713	25713	86119	17474	155,019	133	87	37	8	37.8	Olofsson et al. (2019)
*Notelaea venosa*	NC_042427	25713	25713	86098	17480	155,012	133	87	37	8	37.8	Olofsson et al. (2019)
*Olea europaea*	MT182986	25742	25742	86611	17791	155,806	133	87	37	8	37.8	Niu et al. (2020)
*Osmanthus armatus*	MW648024	25689	25689	86676	17338	155,292	133	87	37	8	37.8	This study
*Osmanthus austrocaledonicus*	MK299397	25713	25713	86538	17496	155460	133	87	37	8	37.8	Olofsson et al. (2019)
*Osmanthus cooperi*	MW727458	25691	25691	86496	17366	155,243	133	87	37	8	37.8	This study
*Osmanthus decorus*	MW727459	25708	25709	86544	17501	155,455	133	87	37	8	37.8	This study
*Osmanthus delavayi*	MW727460	25715	25715	86474	17460	155,364	133	87	37	8	37.8	This study
*Osmanthus didymopetalus*	MT362090	25707	25707	86384	17360	155,155	133	87	37	8	37.8	Zhao, Yang, et al. (2020)
*Osmanthus enervius*	MW727461	25611	25611	86639	17166	155,225	133	87	37	8	37.8	This study
*Osmanthus fordii*	MW727462	25692	25692	86543	17368	155,292	133	87	37	8	37.8	This study
*Osmanthus* ×fortunei	MW727463	25702	25702	86512	17367	155,280	133	87	37	8	37.8	This study
*Osmanthus fragrans*	MH687871	25687	25690	86547	17372	155,296	133	87	37	8	37.8	Duan, Li, Zhang, et al. (2019)
*Osmanthus heterophyllus*	MW727464	25702	25702	86512	17367	155,280	133	87	37	8	37.8	This study
*Osmanthus insularis*	NC_042264	25702	25702	86526	17368	155,298	133	87	37	8	37.8	Olofsson et al. (2019)
*Osmanthus serrulatus*	MW727466	25707	25707	86536	17464	155,411	133	87	37	8	37.8	This study
*Osmanthus suavis*	MW727467	25686	25686	86477	17454	155,303	133	87	37	8	37.8	This study
*Osmanthus urceolatus*	MH229859	25691	25691	86491	17385	155258	133	87	37	8	37.8	Directly submission
*Osmanthus yunnanensis*	MW727465	25707	25707	86584	17490	155,485	133	87	37	8	37.8	This study
*Phillyrea angustifolia*	NC_042464	25709	25709	86416	17501	155,335	133	87	37	8	37.8	Olofsson et al. (2019)
*Phillyrea latifolia*	NC_042465	25706	25705	86400	17489	155,300	133	87	37	8	37.8	Olofsson et al. (2019)
*Picconia azorica*	NC_042428	25706	25715	86419	17510	155,350	133	87	37	8	37.8	Olofsson et al. (2019)
*Picconia excelsa*	NC_042466	25705	25705	86449	17496	155,355	133	87	37	8	37.8	Olofsson et al. (2019)

### 2.2 Phylogenomic analyses

Two different kinds of plastid data were adopted for the phylogenetic analyses of 16 *Osmanthus* species and 12 outgroups. The first dataset was made by the concatenation of 80 common protein‐coding genes (File S1). The second dataset consisted of whole plastomes, which was designed to be compared with the results of the concatenation sequences. In order to reduce information bias caused by duplicate sequences in the plastomes, one of the two IR regions was removed (File S2). The sequences were aligned using MAFFT (Katoh & Standley, 2013). The poorly aligned sequences were trimmed by trimAL software (Capella‐Gutiérrez et al., 2009). The best‐fitting nucleotide substitution models for each gene and for the whole plastomes were calculated by ModelFinder (Kalyaanamoorthy et al., 2017) (Files S1 and S3). Phylogenetic trees were constructed by maximum likelihood (ML), Bayesian inference (BI), and maximum parsimony (MP). ML analyses were performed in IQ‐TREE v1.6.12 (Nguyen et al., 2015), with 1 million ultrafast bootstrap analyses. BI analyses were conducted using MrBayes v3.2.6 (Ronquist & Huelsenbeck, 2003). The search started from a random tree and used two independent Markov chain Monte Carlo (MCMC) chains run for 1 million generations with sampling of trees every 100 generations. The first 25% of the trees were discarded as burn‐in, and the remaining trees were used to generate the 50% majority rule consensus tree. The average standard deviation of split frequencies reached to 0.01, implying convergence of the two runs. MP analyses were performed in MEGA 7 (Kumar et al., 2016) using the search method of tree‐bisection and reconnection (TBR) with 1000 random addition replicates. Branch support was calculated based on the bootstrap method with 1000 replications.

### 2.3 Molecular clock estimation

MCMCtree in the PAML 4.9j package (Yang, 2007) was used to estimate divergence time with an approximate likelihood calculation. The BI phylogenetic tree based on full‐length plastomes from 16 *Osmanthus* and 12 outgroups in subtribe Oleinae was used for divergence time estimation. Estimation of the overall substitution rate was conducted using the BaseML program in PAML. The overall substitution rate (rgene_gamma) and rate drift parameter (sigma2_gamma) were set as G (1, 128) and G (1, 4.5), respectively. The gradient and the Hessian matrix were estimated using the MCMCtree program in PAML under the GTR substitution model. The MCMC process of MCMCtree was run to sample 20,000 times, with sample frequency set at 500, after a burn‐in of 10 million iterations. In total, the MCMC ran for 20,000,000 iterations. Two independent runs were performed to ensure convergence. Distributions of the parameter from MCMC samples were checked by effective sample sizes (ESS) > 200 using Tracer v.1.7 (Rambaut et al., 2018). The results of molecular dating are influenced by many factors, such as the differences between genes, the selection of evolutionary models, and the setting of calibration points. The calibration data for plants are usually derived from limited fossil evidence or secondary calibrations based on previous molecular dating analyses. Therefore, the final results tend to float with the change in calibration schemes (Sauquet et al., 2012). Due to the current lack of fossil evidence in the genus *Osmanthus*, a secondary calibration approach was adopted. The time calibrations were selected based on previous molecular clock studies of Oleaceae. First, based on age estimations from Besnard et al. (2009), the split of genus *Olea* and the NNPP + Osmanthus clade occurred at 32.6 (28.50–37.80) Ma. Second, the divergence between genus *Osmanthus* and the NNPP clade occurred at 15.99 (12.38–19.60) Ma (Olofsson et al., 2019).

### 2.4 Elevational range shifts and flowering time evolution

In order to investigate the evolutionary patterns of flowering time and elevational shifts, we reconstructed the ancestral states for 16 *Osmanthus* and 12 outgroups based on the time tree obtained in the last step. Data on the elevational range and flowering time were obtained based on field observations, literature studies (Chang et al., 1996; Green, 1958, 1972, 2004; Li et al., 2020; Xiang & Liu, 2008), and herbarium information stored in the Chinese Virtual Herbarium (http://www.cvh.ac.cn), the Global Biodiversity Information Facility (http://www.gbif.org/), and JSTOR (https://www.jstor.org). For the 16 *Osmanthus* species, we collected a total of 431 specimens as well as data on their geographical distribution, of which *O*. *fragrans* had the most locations (183), while *O*. *decorus* had the least locations (3). The distribution data of all sampled points are shown in File S4.

We summarized the traits as discrete variables (coded and listed in File S5). For example, the mean elevational range was divided into 500 m and 1500 m; the flowering time was divided into spring (March to April) and autumn (September to October). The estimation of ancestral states was conducted using the R package: Phytools (Revell, 2012). The best‐fitting evolutionary models for the discrete characters were selected using the function “fitMk” in Phytools. An equal rates ("ER") model was selected with the highest Akaike information criterion (AIC) weights for both characters. Posterior probability was summarized from 100 stochastic maps and is presented as a pie chart on each internal node of Figure 3.

### 2.5 Environmental analysis

To investigate the environmental differences between spring‐ and autumn‐flowering plants in the genus *Osmanthus*, we downloaded high‐resolution (30 arc sec) climatic data from CHELSA (https://chelsa‐climate.org/), and extracted 19 variables for the 431 coordinates of the 15 *Osmanthus* species (except for *O*. *austrocaledonicus*) using “extract” function in the R package “raster” (R Core Team, 2021). The climate information of all sampled points is shown in File S4. Phylogenetic principal component analysis (pPCA) was employed based on the “BM” method using the “phyl.pca” function in the R package “phytools” to dimensionally reduce the 19 high‐resolution environmental variables to two principal component axes, with a consideration of phylogenetic relationships between species, and to detect correlations among them. The data scaling and centralizing were conducted before the pPCA analysis in the R (R Core Team, 2021) platform.

### 2.6 Substitution rates and nucleotide diversity of plastid genes

To recognize the mutation hotspots for 80 common protein‐coding genes across the 16 *Osmanthus* species, nucleotide diversity (Pi) values were calculated by DnaSP V. 5.10 software (Rozas & Rozas, 1995). To evaluate whether certain genes in specific lineages within *Osmanthus* (e.g., species with spring‐ vs. autumn‐flowering time or high vs. low elevation) are undergoing positive selection, we performed pairwise Ka/KS calculations on 80 orthologous genes from the 16 *Osmanthus* species using KaKs_calculator 2.0 (Wang et al., 2010) with the settings genetic code table 11 (bacterial and plant plastid code) and the “YN” method of calculation. If a particular gene in a particular lineage is positively selected for, then we will see that its paired Ka/Ks will be greater than 1, and if it is being subjected to neutral or purifying selection then we will get a value of 1 or less than 1. In our results, some genes had Ka/Ks values equal to “NA,” indicating that no synonymous substitutions (Ks) occurred in these genes.

## 3 RESULTS

### 3.1 Phylogenetic analyses

Phylogenetic trees constructed from two datasets (whole plastomes and concatenation of 80 protein‐coding genes) using three different tree building methods (BI, ML, and MP) are shown in File S6. The topologies of all trees were highly consistent and similar, but the BI tree constructed from whole plastomes exhibited the highest branch support, and so was used for display and downstream analyses.

**FIGURE 1:**
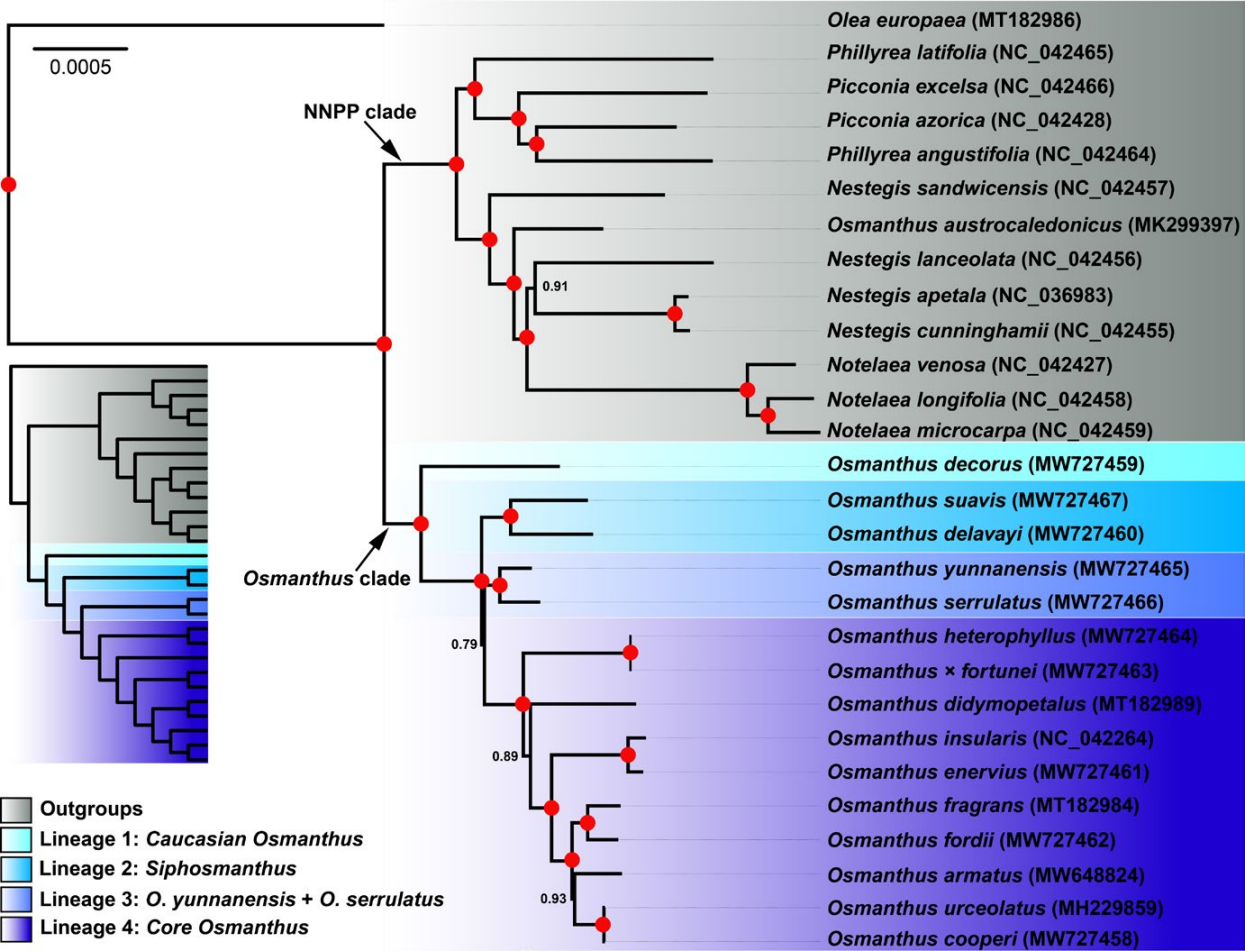
Bayesian inference tree of 15 *Osmanthus* and 13 outgroup species based on whole‐length plastomes. Bayesian inference posterior probability (BIPP) is marked on each node. Nodes with 100% posterior probability are marked as red dots. A simplified version of the cladogram is also shown. The four main lineages in *Osmanthus* and outgroups are shown against colored backgrounds

In our new phylogeny (Figure 1 and File S6), the New Caledonian species *O*. *austrocaledonicus* separated from all other Asian *Osmanthus* species (the *Osmanthus* clade) as a distinct lineage nested within the Australasian genera *Notelaea* and *Nestegis* with strong support (BIPP: 1.00/1.00; MLBS: 100%/100%; and MPBS: 100%/100%). Except for *O*. *austrocaledonicus*, the monophyly of the *Osmanthus* clade is confirmed (BIPP: 1.00/1.00; MLBS: 100%/100%; MPBS: 99%/99%) and is resolved as sister to the NNPP clade. Within the *Osmanthus* clade, the 15 species were grouped into four major clades: the (here designated) Caucasian *Osmanthus* clade (BIPP: 1.00/1.00; MLBS: 100%/100%; MPBS: 100%/100%), the (here designated) *Siphosmanthus* clade (BIPP: 1.00/1.00; MLBS: 100%/100%; MPBS: 100%/99%), the (here designated) *O*. *serrulatus* + *O. yunnanensis* clade (BIPP: 1.00/1.00; MLBS: 100%/94%; MPBS: 99/92%), and the Core *Osmanthus* clade (BIPP: 1.00/1.00; MLBS: 100%/100%; MPBS: 100%/100%). The Caucasian *Osmanthus* clade (corresponding to *O*. *decorus*) was resolved as sister to the remaining 14 accessions of *Osmanthus*, followed by the strongly supported *Siphosmanthus* clade (corresponding to taxa of *O*. sect. *Siphosmanthus*: *O*. *suavis* and *O*. *delavayi*). The *Siphosmanthus* clade was placed sister to a large clade comprising the *O*. *yunnanensis* + *O. serrulatus* clade and the *Core Osmanthus* clade, the branch support of this clade being low (BIPP: 0.74/0.79; MLBS: 87%/78%; MPBS: 88%/75%). The *Core Osmanthus* clade (corresponding to taxa of *O*. sect. *Osmanthus* and *O*. sect. *Linocieroides*) contained the most species, including *O*. *cooperi*, *O*. *heterophyllus*, *O*. × *fortunei*, *O*. *fragrans*, *O*. *fordii*, *O*. *enervius*, *O*. *armatus*, *O*. *urceolatus*, and *O*. *didymopetalus*.

### 3.2 Molecular dating

**FIGURE 2:**
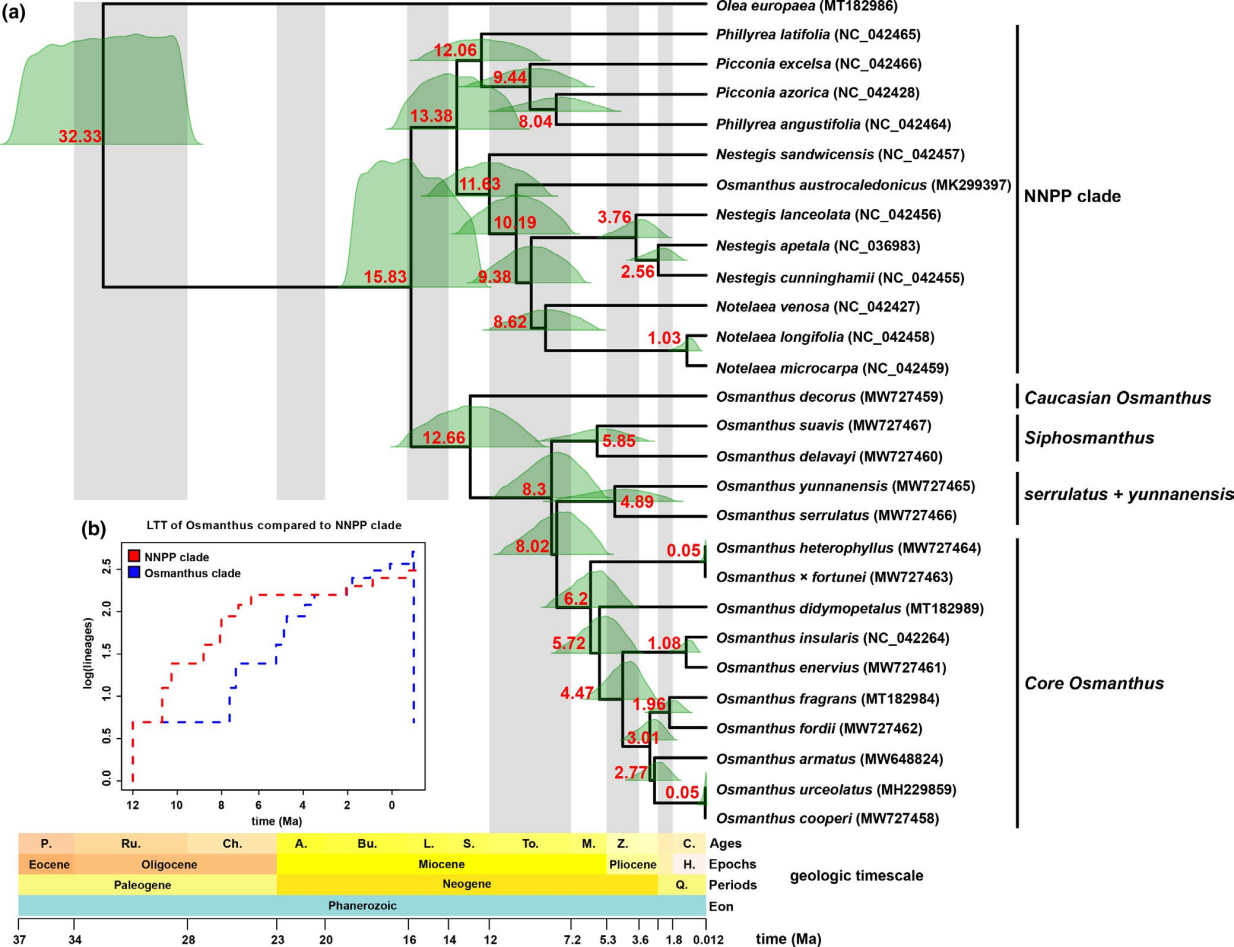
(a) Chronogram illustrating divergence time of *Osmanthus* and close relatives. The full posterior distribution of age estimates is displayed for every node on the phylogeny. The numbers in red font represent the mean posterior of time. The geologic timescale and timescale are drawn at the bottom of the figure. (b) Changes in lineages over time in the *Osmanthus* clade and NNPP clade are depicted by lineage‐through‐time (LTT) plots. The four main lineages in the *Osmanthus* and NNPP clade are marked against vertical lines. Abbreviations of geological time: P. Priabonian, Ru. Rupelian, Ch. Chattian, A. Aquitanian, Bu. Burdigalian, L. Langhian, S. Serravallian, To. Tortonian, M. Messinian, Z. Zanclean, Q. Quaternary, H. Holocene, and C. Calabrian

Based on the results of MCMCtree estimation (Figure 2; File S7), the most recent common ancestor (MRCA) of *Olea europaea* and the remaining 27 species were estimated to have diverged at 32.33 Ma (95% HPD: 27.94–36.88 Ma) in the Early Oligocene. The MRCA of the *Osmanthus* and NNPP clade was estimated to have diverged at 15.83 Ma (95% HPD: 12.23–19.04 Ma) in the Middle Miocene. The crown age for the NNPP clade was estimated as 13.38 Ma (95% HPD: 10.07–16.54 Ma) in the Middle Miocene. The crown age of genus *Osmanthus* was estimated as 12.66 Ma (95% HPD: 9.00–16.26 Ma) in the Middle Miocene. The crown age of *Siphosmanthus* clade was estimated as 5.85 Ma (95% HPD: 3.21–8.69 Ma). The MRCA of *O*. *yunnanensis* + *O*. *serrulatus* clade and the *Core Osmanthus* clade was estimated to have diverged at 8.02 Ma (95% HPD: 3.21–8.69 Ma) in the Late Miocene. The crown age of *O*. *yunnanensis* + *O*. *serrulatus* clade was estimated as 4.89 Ma (95% HPD: 1.87–8.23 Ma) in the Late Miocene. The crown age of the *Core Osmanthus* was estimated as 6.2 Ma (95% HPD: 3.97–8.47 Ma) in the Late Miocene.

### 3.3 Ancestral state reconstructions

**FIGURE 3:**
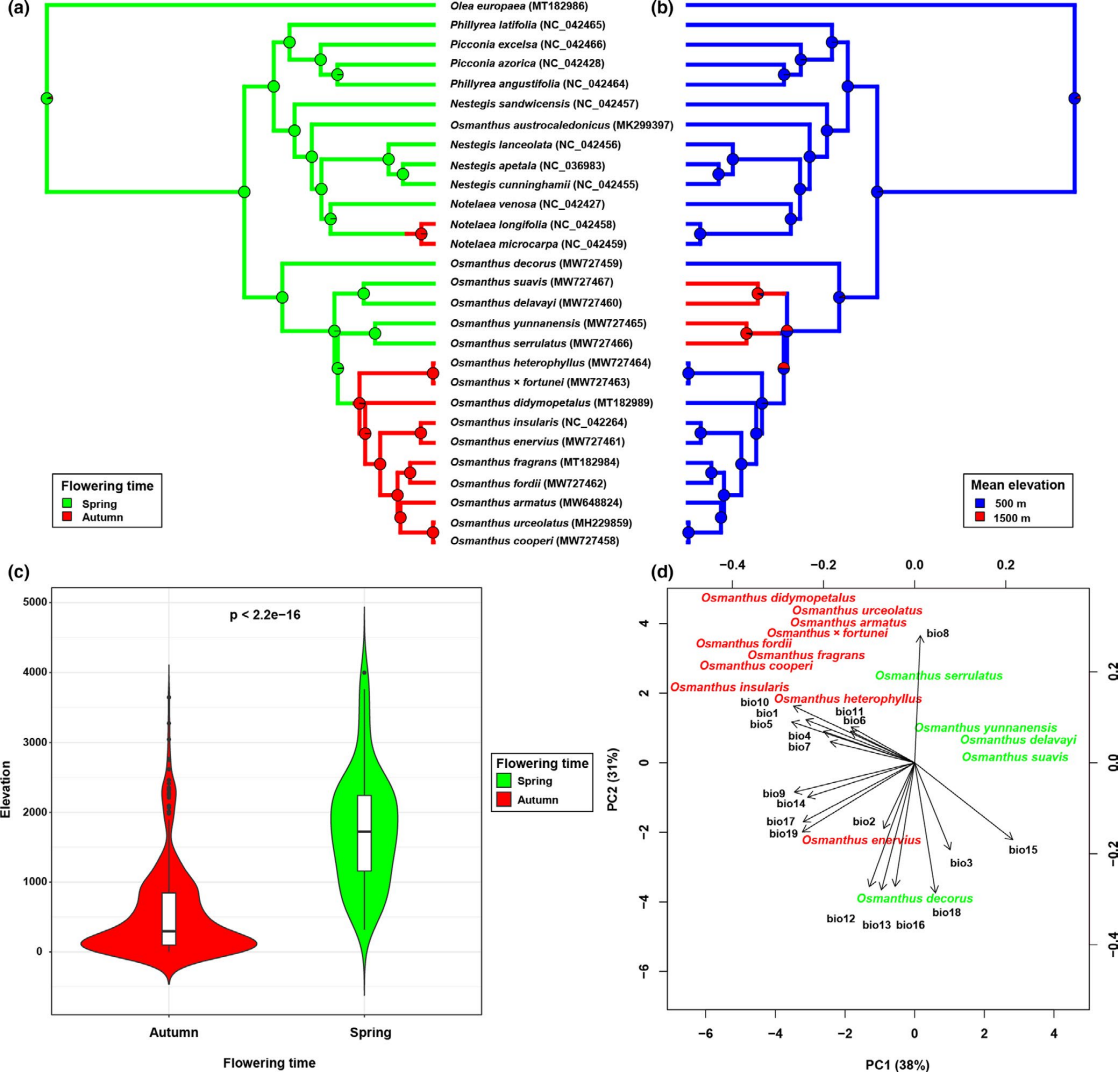
(a, b) Stochastic character mapping of flowering time and elevational ranges. Current character states are annotated beside the species names and pie graphs represent the posterior probability of each character state on the ancestor node. (a) Flowering time is coded as spring (March to April) or autumn (September to October). (b) Elevational range is coded as low elevations (mean elevation ca. 500 m) or high elevations (mean elevation ca. 1500 m). (c) Comparison of elevation range among autumn‐ and spring‐flowering species in *Osmanthus*. (d) Phylogenetic PCA (pPCA) of 19 high‐resolution environmental variables for autumn‐ and spring‐flowering species in *Osmanthus*

Ancestral state reconstructions are presented in Figure 3a,b. Flowering in spring was inferred to be the ancestral state in *Olea*, *Phillyrea*, *Picconia*, *Notelaea*, *Nestegis*, and *Osmanthus*. Flowering in autumn was inferred to be a derived character in *Nestegis* and *Osmanthus*. Autumn‐flowering species evolved independently two times in our analysis, one time in *Nestegis* and the other time in *Osmanthus*. The most recent ancestors of autumn‐flowering ancestral species in *Osmanthus* appeared at 6.2 Ma during the Late Miocene. Low elevation (mean elevation ca. 500 m) was reconstructed as the ancestral habitat of *Olea*, *Phillyrea*, *Picconia*, *Notelaea*, *Nestegis*, and *Osmanthus*. High elevation (mean elevation ca. 1500 m) was recognized as a derived state in *Osmanthus*, having evolved twice in the genus: once with the emergence of *O*. *delavayi* and *O*. *suavis* at 8.3 Ma, another with the emergence of clade *O*. *serrulatus* and *O*. *yunnanensis* at 8.02 Ma.

### 3.4 Environmental analysis

In the pPCA for spring‐ and autumn‐flowering species in *Osmanthus*, the first two axes explained 38% and 31% of total variation, respectively (Figure 3c,d). The PC1 axis represented the global structure, indicating the variables are more similar in closely related species (species with the same flowering time) than in distant species (species with different flowering time), while the PC2 axis represented the local structure indicating the variables can create dissimilarities among closely related species (species with the same flowering time). Species with different flowering times could be distinguished from each other along the PC1 axis: Autumn‐flowering species (*O*. *armatus*, *O*. *cooperi*, *O*. *didymopetalus*, *O*. *fordii*, *O*. × *fortunei*, *O*. *fragrans*, *O*. *heterophyllus*, *O*. *insularis*, *O*. *enervius*, and *O*. *urceolatus*) clustered together on the negative side of PC1, while spring‐flowering species (*O*. *decorus*, *O*. *serrulatus*, *O*. *suavis*, *O*. *yunnanensis*, and *O*. *delavayi*) were clustered on the positive side of PC1. For spring‐flowering species, all East Asian species were on the positive side of the PC2 axis, while only *O*. *decorus* from West Asia was on the negative side of the PC2 axis. For autumn‐flowering species, only *O*. *enervius* from Taiwan island, China, was on the negative side of the PC2 axis, while all other species were positioned on the positive side of the PC2 axis (Figure 3d).

### 3.5 Genome structures and nucleotide variation

The plastome sizes ranged from 155,155 bp to 155,485 bp (Table 1). The length of IRs ranged from 25,611 bp to 25,715 bp. The length variation for the SSC, ranged from 17,166 bp to 17,501 bp. The length of the LSC varied from 86,384 bp to 86,676 bp. Plastomes of *Osmanthus* encoded an identical set of 133 predicted functional genes, in which 87 are protein‐coding genes (eight duplicated genes), 37 are tRNA genes (seven duplicated genes), and 8 are rRNA genes (four duplicated genes). The overall GC content of the genomes was 37.8%, while the IRA and IRB regions showed higher GC contents. The locally collinear blocks (LCBs) showed all the genes maintain a consistent position and direction, with no rearrangement or inversion events being found (File S8).

**FIGURE 4:**
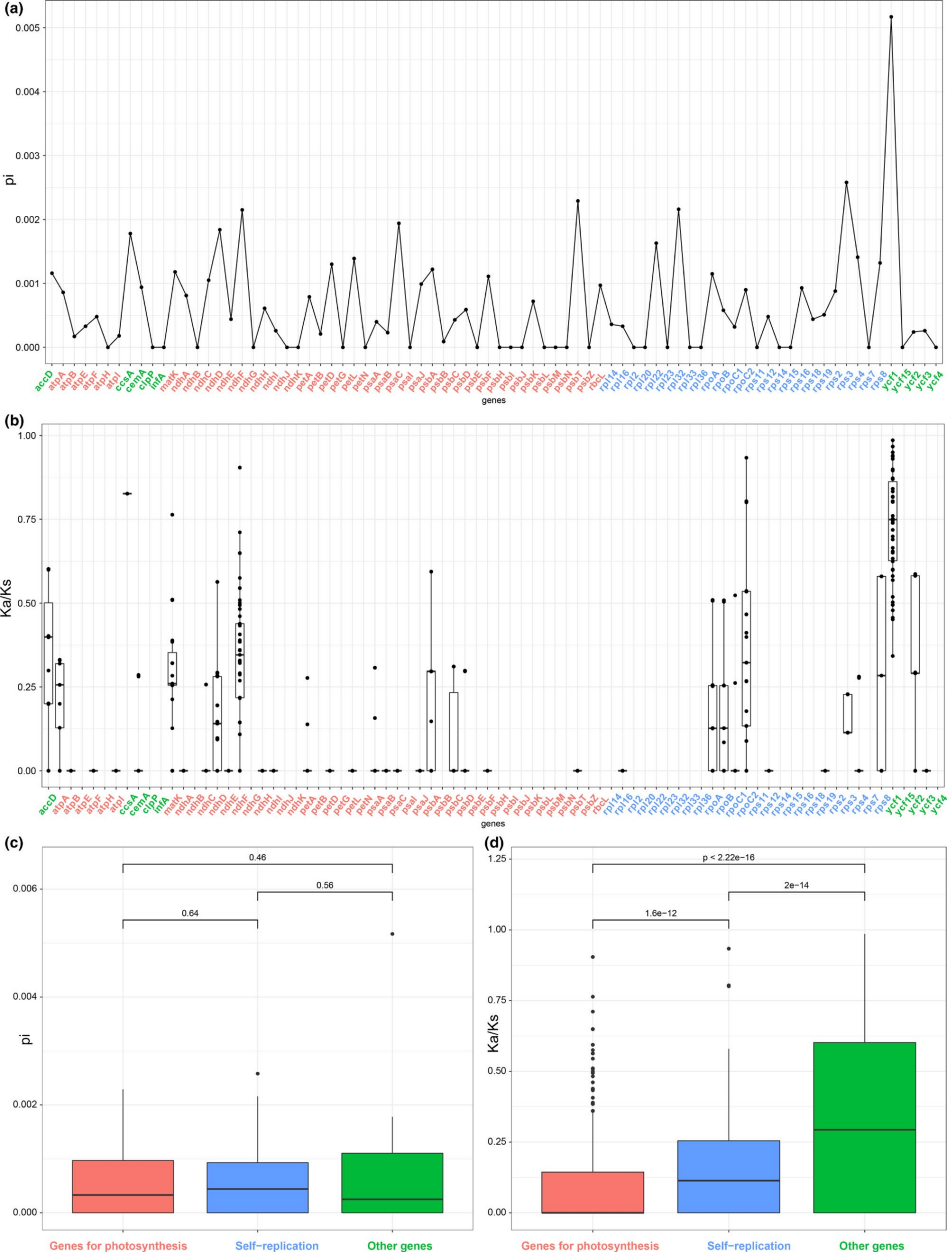
(a) Comparison of nucleotide variability (Pi values) in *Osmanthus* plastomes. (b) Comparison of substitution ratio (Ka/Ks) of 80 common genes in *Osmanthus* plastomes. (c) Comparisons of divergence of Pi among genes for photosynthesis, self‐replication genes, and other genes in *Osmanthus*. (d) Comparisons of divergence of Ks/Ks among genes for photosynthesis, self‐replication genes, and other genes in *Osmanthus*

Nucleotide variation analyses of the 80 protein‐coding genes revealed Pi values in the range 0–0.005 with an average of 0.0006 (Figure 4a,c, File S9). No hypervariable loci (Pi > 0.01) were found. Only the *ycf1* gene had the highest nucleotide variation (Pi > 0.003) of all 80 genes. In terms of gene function, genes related to photosynthesis and self‐replication showed the lowest values of nucleotide variation (Figure 4a,c). A total of 8322 pairs of Ka/Ks values were calculated for the 80 genes among the 15 Asian *Osmanthus* species. The value of Ka ranged from 0.0001 to 0.008, with an average of 0.0003. The value of Ks ranged from 0.0006 to 0.05, with an average of 0.001. The value of Ka/Ks ranged from 0 to 0.98, with an average of 0.02 (Figure 4b,d, File S10). The *ycf1* gene exhibited the highest Ka/Ks value with an average of 0.35. In terms of gene function, the genes associated with photosynthesis had the least values of Ka/Ks, followed by self‐replication genes and other genes (Figure 4b,d).

## 4 DISCUSSION

### 4.1 Phylogenetic relationships of *Osmanthus*

Previous molecular studies have recognized close relationships between *Osmanthus* and the other four genera in the NNPP clade (Guo et al., 2011; Li et al., 2020; Lu et al., 2011; Yuan et al., 2010). Our plastome phylogeny also confirm their results and suggest a polyphyletic status of traditional *Osmanthus*. Particularly, our plastome data find the New Caledonian species, *O*. *austrocaledonicus*, is in a subclade consisting of *Nestegis* and *Notelaea* species. Nuclear‐based phylogenetic trees also agree with this finding (Dupin et al., 2020; Li et al., 2020; Olofsson et al., 2019). Morphologically, Asian *Osmanthus* species are different from species of the NNPP clade and *O*. *austrocaledonicus* in their inflorescences being cymose, axillary, with corollas imbricate in bud, as well as wood with a torus (Dute et al., 2010; Li et al., 2020). Therefore, based on molecular, morphological, and biogeographical evidence, we recommend exclusion of *O*. *austrocaledonicus* from *Osmanthus* to ensure the monophyly of the genus.

Other similarities and inconsistencies are apparent when comparing our findings with previous taxonomic research (Chang et al., 1996; Green, 1958) based on purely morphological characters, which divided *Osmanthus* into three sections (*O*. sect. *Linocieroides* [only one species *O*. *didymopetalus*]; *O*. sect. *Siphosmanthus* (*O*. *delavayi* and *O*. *suavis*); and *O*. sect. *Osmanthus* [all remaining species]). First, our new phylogeny, as well as previous molecular studies (Li et al., 2020), found that *O*. sect. *Linocieroides* should be merged into *O*. sect. *Osmanthus*. Second, *O*. *serrulatus* and *O*. *yunnanensis* were classified into *O*. sect. *Osmanthus* in previous morphological studies (Chang et al., 1996; Green, 1958). Now, the sister relationship of this lineage between the *O*. sect. *Osmanthus* have received low support in our phylogenetic studies. This suggests that plastomes weakly support the inclusion of this lineage in *O*. sect. *Osmanthus*. We also found morphological evidence, such as bracts ciliate, leaf venation reticulate, and spring flowering, which are significantly different from other species in *O*. sect. *Osmanthus* (Chang et al., 1996). Third, *O*. *decorus*, previously considered as belonging to *O*. sect. *Osmanthus*, was distinguished as a distinct lineage sister to all other *Osmanthus* species in the phylogeny, and is here termed the Caucasian *Osmanthus* clade until a more comprehensive phylogenetic study can be done to determine its subgeneric ranking. The Caucasian *Osmanthus* clade is noted for its unique geographical distribution: *O*. *decorus* is the only representative of *Osmanthus* in West Asia and has been considered as a tertiary relict species from high elevations of the Caucasus region (Browicz, 1989; Manvelidze et al., 2010; Melia et al., 2012). Fourth, the Core *Osmanthus* clade, aside from addition of the *O*. *didymopetalus* and removal of *O*. *decorus* and “*O*. *serrulatus* + *O. yunnanensis*,” is largely consistent with that delineated in previous taxonomic research. As delimited here, the Core *Osmanthus* clade contains the largest number of species and characterized by short corolla tubes (≤5 mm) and long leaf length (7–14 cm), which occupy lower elevations (mean elevation ca. 500 m) and are all endemic to East Asia. Fifth and lastly, our findings did corroborate the previous delimitation of *O*. *delavayi* and *O*. *suavis* as belonging to a distinct lineage within *Osmanthus*, which we here termed the “*Siphosmanthus* clade” until a more comprehensive phylogenetic study has been done to determine the subgeneric ranking of this lineage. The *Siphosmanthus* clade is resolved in a grade between the Caucasian *Osmanthus* and the other East Asian species, with strong branch support. This lineage is morphologically distinct from other *Osmanthus* species in having long corolla tubes (6–10 mm) and short leaf lengths (1–7 cm). In addition, they are the species that occupy the highest elevations (mean elevation ca. 2000 m) (Chang et al., 1996).

### 4.2 Molecular dating of *Osmanthus*

Molecular dating shows that the MRCA of *Osmanthus* and the NNPP clade occurred at ca. 15.83 Ma (during the Langhian stage in the middle Miocene). The LTT plot (Figure 2b) suggests that NNPP clade speciation occurred slightly earlier (ca. 1 Ma) than that of the *Osmanthus* clade. Ample evidence suggests that Oleaceae exhibited greater diversity in Europe during the tertiary (Sachse, 2001; Srivastava et al., 2015). After the Oligocene, the Tethys or Tethys Ocean that was found between Gondwana and Laurasia, and influenced global oceanic circulation and climate patterns, climate gradually became cool and arid, with many temperate floral elements increasing in the ancient Mediterranean flora. Analysis of Miocene pollen and leaf fossils in Western Europe has shown that plant diversity in the temperate zone increased significantly compared with that in the Eocene, some modern Mediterranean elements such as *Olea* and *Phillyrea* beginning to appear during this period (Sachse, 2001; Sun & Li, 2003; Sun et al., 2017; Torfstein & Steinberg, 2020). The crown age of the genus *Osmanthus* is here estimated to have appeared ca. 12.66 Ma (during the Langhian stage in the middle Miocene). Despite the lack of direct fossil evidence of *Osmanthus*, the Tethyan relict of *O*. *decorus* is supported by multiple studies (Browicz, 1989; Manvelidze et al., 2010; Melia et al., 2012; Sachse, 2001), and thus agrees with the results of our molecular clock. Within the genus, most species of *Osmanthus* appeared after the middle Miocene and originated ca. 4 Ma more recently than those of the NNPP clade. Two steps of species accumulation were depicted in the LTT plot (Figure 2b), which occurred at ca. 8 and 6 Ma, respectively, corresponding to the formation of the high‐ and low‐elevation species of *Osmanthus*. The emergence of the two high‐elevation clades (*O*. *delavayi* + *O*. *suavis* and *O*. *serrulatus* + *O. yunnanensis*), which are distributed in the Hengduan Mountains, mainly occurred in the late Miocene.

### 4.3 Evolutionary shifts of elevational range and flowering time

Integrating the results of molecular dating and stochastic character/state mapping, we find that *Osmanthus* originated at low elevations in the middle Miocene, then higher‐elevation species began to appear in the Hengduan Mountains in the late Miocene. According to geological evidence, orogenesis was still occurring on the Qinghai–Tibet Plateau and its adjacent areas such as the Hengduan Mountains during this period. Recent studies have suggested that orogenesis was one of the important driving forces for the increase in species diversity in this region (Xing & Ree, 2017; Ye et al., 2019). Thus, we speculate that the emergence of high‐elevation species in *Osmanthus* is closely related to the uplift of the Tibetan Plateau.

The ancestors of *Osmanthus* were presumed to be spring‐flowering species, while autumn‐flowering species are only found in the Core *Osmanthus* clade, which are distributed in southeastern China and Japan. Research has found that species flowering times do shift with climate change to optimize reproductive strategies (Bucher & Römermann, 2020; Gaudinier & Blackman, 2020; Janet, 2020). Abiotic factors such as droughts and seasonal precipitation are also considered as potentially selective forces on flowering phenology (Franks et al., 2007; Lesica & Kittelson, 2010). In the pPCA analysis, we found that spring‐ and autumn‐flowering species were clearly distinguished along the PC1 axis. This indicates ecological niche divergence between spring‐ and autumn‐flowering plants. From the perspective of geographical distribution, the spring‐flowering lineages of *Osmanthus* are native to the Caucasus region and Hengduan Mountains, both of which have their lowest precipitation in spring (March to April) (Yang et al., 2009). On the other hand, the autumn‐flowering lineages are native to southeastern China and Japan where the lowest precipitation is usually in autumn and winter (September to February) (Yao et al., 2017). Based on this, we hypothesize that low precipitation might create relatively favorable conditions for flowering and pollination. Some cases have shown that increased precipitation may alter the flowering time and increase the risk of pollen degradation and nectar dilution (Lawson & Rands, 2019). Although the relationship between flowering phenology and environmental conditions is systematic and complex, our research provides new insights into the adaptation of species to different environments in eastern Asia.

### 4.4 Conserved evolution of *Osmanthus* plastomes

*Osmanthus* species displayed the same genetic composition and high collinearity, with no recombination or inversion being found. Most of the genetic regions exhibited low nucleotide variability (Pi < 0.004) and almost all the nucleotide variations were non‐synonymous substitutions. This indicates that genetic variation within *Osmanthus* is very limited. When comparing between genes, we found that the *ycf1* gene harbored the most nucleotide variability across *Osmanthus* species. It is worth noting that many recent studies have found *ycf1* to be taxonomically informative in phylogenetic research (Dong et al., 2015; Liu et al., 2010), thus making it a good candidate marker as an *Osmanthus* plant barcode.

By calculating the nucleotide substitution rate, we found the Ka/Ks values of 80 common genes were less than 1. This indicates that all the genes are undergoing strong purifying selection, which was expected for genes under strong functional constraints like photosynthesis. Purifying selection usually reduces genetic diversity via selective removal of alleles that are deleterious (Cvijović et al., 2018). More exactly, the functional importance of a protein is a major determinant of its evolutionary rate (Zhang & Yang, 2015). Our Ka/Ks pairwise calculation did not detect the signal of positive gene selection in specific species groups (high elevation vs. low elevation; spring flowering vs. autumn flowering). This indicates that the plastid genes are likely to not be significantly involved in adaptation to altitude or precipitation. Thus, a nuclear genome‐wide or transcriptome approach is necessary to study selection pressures on *Osmanthus* species.

## 5 CONCLUSION

This is the first well‐sampled report on the plastomes of *Osmanthus*. Comparative genomic analysis revealed conserved genome structures and low nucleotide polymorphism, although identifying *ycf1* as a phylogenetically informative marker that could be used in future studies to gain a more comprehensive overview of *Osmanthus* systematics and identification. Our phylogenetic analysis partially supports previous morphological classification systems including the distinctiveness of taxa belonging to *O*. sect. *Siphosmanthus* and *O*. sect. *Osmanthus*. We also provide new insights, such as *O*. *decorus* found to be an independent lineage and sect. *Linocieroides* was found to belong within sect. *Osmanthus*. The phylogenetic relationships between species are also much clearer than those resolved in previous studies. Molecular dating of each lineage was also estimated for the first time using two secondary calibration points, with the results suggesting that *Osmanthus* is a tertiary relict genus. The origin and diversification of *Osmanthus* species is speculated to be closely related to the climatic changes and the orogenesis of the Qinghai–Tibet Plateau during the Miocene. Analysis of character evolution revealed the ancestral states of spring‐flowering time and low‐elevation distribution in the genus. Differentiation of spring‐ and autumn‐flowering time in *Osmanthus* was also found to be related to the differences in ecological niche between species. The evolutionary rates of *Osmanthus* plastomes are very conservative, and so are unable to provide effective genetic information for the study of adaptive evolution. Our results provide a framework for the further study of the systematics and historical biogeography of *Osmanthus*, including formalizing a subgeneric classification of *Osmanthus*. A comprehensive molecular sampling of all species should now be focused on in order to achieve these aims.

## CONFLICT OF INTEREST

None declared.

## AUTHOR CONTRIBUTIONS

**Yongfu Li:** Conceptualization (lead); Data curation (lead); Formal analysis (lead); Investigation (lead); Methodology (lead); Project administration (lead); Resources (lead); Software (lead); Validation (lead); Visualization (lead); Writing – original draft (equal); Writing – review & editing (equal). **Xuan Li:** Formal analysis (equal); Resources (equal). **Steven Paul Sylvester:** Writing – original draft (equal); Writing – review & editing (equal). **Min Zhang:** Software (supporting). **Xianrong Wang:** Funding acquisition (lead); Supervision (lead). **Yifan Duan:** Funding acquisition (supporting); Supervision (supporting).

## Supporting information

Supplementary Material

Supplementary Material

Supplementary Material

Supplementary Material

Supplementary Material

Supplementary Material

Supplementary Material

Supplementary Material

Supplementary Material

Supplementary Material

## Data Availability

The *Osmanthus* plastomes generated in this study are available in the NCBI GenBank repository: *O*. *armatus* (MW648824), *O*. *cooperi* (MW727458), *O*. *fordii* (MW727462), *O*. *enervius* (MW727461), *O*. × *fortunei* (MW727463), *O*. *heterophyllus* (MW727464), *O*. *serrulatus* (MW727466), *O*. *yunnanensis* (MW727465), *O*. *delavayi* (MW727460), *O*. *suavis* (MW727467), and *O*. *decorus* (MW727459). Aligned DNA sequences in FASTA format are deposited in the Dryad repository (https://doi.org/10.5061/dryad.bcc2fqzf6).

## References

[ece38777-bib-0001] Besnard, G. , Rubio de Casas, R. , Christin, P. A. , & Vargas, P. (2009). Phylogenetics of *Olea* (Oleaceae) based on plastid and nuclear ribosomal DNA sequences: Tertiary climatic shifts and lineage differentiation times. Annals of Botany, 104(1), 143–160. 10.1093/aob/mcp105 19465750 PMC2706730

[ece38777-bib-0002] Browicz, K. (1989). Chorology of the Euxinian and Hyrcanian element in the woody flora of Asia. Plant Systematics and Evolution, 162(1‐4), 305–314. 10.1007/BF00936923

[ece38777-bib-0003] Bucher, S. F. , & Römermann, C. (2020). Flowering patterns change along elevational gradients and relate to life‐history strategies in 29 herbaceous species. Alpine Botany, 130(1), 41–58. 10.1007/s00035-020-00231-w

[ece38777-bib-0004] Capella‐Gutierrez, S. , Silla‐Martinez, J. M. , & Gabaldon, T. (2009). trimAl: A tool for automated alignment trimming in large‐scale phylogenetic analyses. Bioinformatics, 25(15), 1972–1973. 10.1093/bioinformatics/btp348 19505945 PMC2712344

[ece38777-bib-0005] Chang, M. , Chiu, L. , Wei, Z. , & Green, P. (1996). Oleaceae. In Z. Y. Wu & P. H. Raven (Eds.), Flora of China. (Vol. 10). Flora of China. Science Press; Missouri Botanical Garden Press.

[ece38777-bib-0006] Cvijović, I. , Good, B. H. , & Desai, M. M. (2018). The effect of strong purifying selection on genetic diversity. Genetics, 209(4), 1235–1278. 10.1534/genetics.118.301058 29844134 PMC6063222

[ece38777-bib-0007] Dierckxsens, N. , Mardulyn, P. , & Smits, G. (2017). NOVOPlasty: De novo assembly of organelle genomes from whole genome data. Nucleic Acids Research, 45(4), e18. 10.1093/nar/gkw955 28204566 PMC5389512

[ece38777-bib-0008] Dong, W. P. , Xu, C. , Li, C. H. , Sun, J. H. , Zuo, Y. J. , Shi, S. , Cheng, T. , Guo, J. J. , & Zhou, S. L. (2015). *ycf1*, the most promising plastid DNA barcode of land plants. Scientific Reports, 5(1), 8348. 10.1038/srep08348 25672218 PMC4325322

[ece38777-bib-0009] Duan, Y. F. , Li, W. H. , Zheng, S. Y. , Sylvester, S. P. , Li, Y. F. , Cai, F. Y. , Zhang, C. , & Wang, X. R. (2019). Functional androdioecy in the ornamental shrub *Osmanthus delavayi* (Oleaceae). PLoS One, 14(9), e0221898. 10.1371/journal.pone.0221898 31487330 PMC6728067

[ece38777-bib-0010] Duan, Y. F. , Li, Y. F. , Zhang, C. , Wang, X. R. , & Li, M. Z. (2019). The complete chloroplast genome of sweet olive (*Osmanthus fragrans* Lour.). Mitochondrial DNA Part B: Resources, 4(1), 1063–1064. 10.1080/23802359.2019.1586476

[ece38777-bib-0011] Dupin, J. , Raimondeau, P. , Hong‐Wa, C. , Manzi, S. , Gaudeul, M. , & Besnard, G. (2020). Resolving the phylogeny of the olive family (Oleaceae): Confronting information from organellar and nuclear genomes. Genes, 11(12), 1–18. 10.3390/genes11121508 PMC7767060 33339232

[ece38777-bib-0012] Dute, R. , Rabaey, D. , Allison, J. , & Jansen, S. (2010). Torus‐bearing pit membranes in species of *Osmanthus* . IAWA Journal, 31(2), 217–226. 10.1163/22941932-90000018

[ece38777-bib-0013] Franks, S. J. , Sim, S. , & Weis, A. E. (2007). Rapid evolution of flowering time by an annual plant in response to a climate fluctuation. Proceedings of the National Academy of Sciences of the United States of America, 104(4), 1278–1282. 10.1073/pnas.0608379104 17220273 PMC1783115

[ece38777-bib-0014] Gao, L. Z. , Liu, Y. L. , Zhang, D. , Li, W. , Gao, J. , Liu, Y. , Li, K. , Shi, C. , Zhao, Y. , Zhao, Y. J. , Jiao, J. Y. , Mao, S. Y. , Gao, C. W. , & Eichler, E. E. (2019). Evolution of *Oryza* chloroplast genomes promoted adaptation to diverse ecological habitats. Communications Biology, 2(1), 278. 10.1038/s42003-019-0531-2 31372517 PMC6659635

[ece38777-bib-0015] Gao, X. Y. , Zhang, X. , Meng, H. H. , Li, J. , Zhang, D. , & Liu, C. N. (2018). Comparative chloroplast genomes of *Paris* Sect. *Marmorata*: Insights into repeat regions and evolutionary implications. BMC Genomics, 19(Suppl 10), 878. 10.1186/s12864-018-5281-x 30598104 PMC6311911

[ece38777-bib-0016] Gaudinier, A. , & Blackman, B. K. (2020). Evolutionary processes from the perspective of flowering time diversity. New Phytologist, 225, 1883–1898. 10.1111/nph.16205 31536639

[ece38777-bib-0017] Green, P. S. (1958). A MONOGRAPHIC REVISION of *Osmanthus* in Asia and America. London: Notes from the Royal Botanic Garden Edinburgh. H.M.Stationery Office.

[ece38777-bib-0018] Green, P. S. (1972). *Osmanthus decorus* and disjunct Asiatic‐European distributions in the Oleaceae. Kew Bulletin, 26(3), 487. 10.2307/4120313

[ece38777-bib-0019] Green, P. S. (2004). Oleaceae. In J. W. Kadereit (Ed.), Flowering Plants. Dicotyledons. The Families and Genera of Vascular Plants (Vol. 7). Springer. 10.1007/978-3-642-18617-2_16

[ece38777-bib-0020] Gu, C. H. , Tembrock, L. R. , Zheng, S. Y. , & Wu, Z. Q. (2018). The complete chloroplast genome of *Catha edulis*: A comparative analysis of genome features with related species. International Journal of Molecular Sciences, 19(2), 525. 10.3390/ijms19020525 PMC5855747 29425128

[ece38777-bib-0021] Guo, S. Q. , Xiong, M. , Ji, C. F. , Zhang, Z. R. , Li, D. Z. , & Zhang, Z. Y. (2011). Molecular phylogenetic reconstruction of *Osmanthus* Lour. (Oleaceae) and related genera based on three chloroplast intergenic spacers. Plant Systematics and Evolution, 294(1–2), 57–64. 10.1007/s00606-011-0445-z

[ece38777-bib-0022] Ha, Y. H. , Kim, C. , Choi, K. , & Kim, J. H. (2018). Molecular phylogeny and dating of Forsythieae (Oleaceae) provide insight into the Miocene history of Eurasian temperate shrubs. Frontiers in Plant Science, 104(1), 143–160. 10.3389/fpls.2018.00099 PMC5807412 29459880

[ece38777-bib-0023] Janet, S. P. (2020). Climate change: Flowering time may be shifting in surprising ways. Current Biology, 30(3), 112–114. 10.1016/j.cub.2019.12.009 32017877

[ece38777-bib-0024] Kalyaanamoorthy, S. , Minh, B. Q. , Wong, T. K. F. , von Haeseler, A. , & Jermiin, L. S. (2017). ModelFinder: Fast model selection for accurate phylogenetic estimates. Nature Methods, 14(6), 587–589. 10.1038/nmeth.4285 28481363 PMC5453245

[ece38777-bib-0025] Katoh, K. , & Standley, D. M. (2013). MAFFT multiple sequence alignment software version 7: Improvements in performance and usability. Molecular Biology and Evolution, 30(4), 772–780. 10.1093/MOLBEV/MST010 23329690 PMC3603318

[ece38777-bib-0026] Kumar, S. , Stecher, G. , & Tamura, K. (2016). MEGA7: Molecular evolutionary genetics analysis version 7.0 for bigger datasets. Molecular Biology and Evolution, 33(7), 1870–1874. 10.1093/MOLBEV/MSW054 27004904 PMC8210823

[ece38777-bib-0027] Lawson, D. A. , & Rands, S. A. (2019). The effects of rainfall on plant–pollinator interactions. Arthropod‐Plant Interactions, 13(4), 561–569. 10.1007/s11829-019-09686-z

[ece38777-bib-0028] Lesica, P. , & Kittelson, P. M. (2010). Precipitation and temperature are associated with advanced flowering phenology in a semi‐arid grassland. Journal of Arid Environments, 74(9), 1013–1017. 10.1016/J.JARIDENV.2010.02.002

[ece38777-bib-0029] Li, Y. F. , Zhang, C. , Zhu, H. , Li, X. , Duan, Y. F. , & Wang, X. R. (2019). Analyses on suitable distribution areas and main climatic variables of *Osmanthus yunnanensis* and *O. delavayi* . Journal of Plant Resources and Environment, 28(1), 71–78. 10.3969/j.issn.1674-7895.2019.01.09

[ece38777-bib-0030] Li, Y. F. , Zhang, M. , Wang, X. R. , Sylvester, S. P. , Xiang, Q. B. , Li, X. , Li, M. , Zhu, H. , Zhang, C. , Chen, L. , Yi, X. G. , Mao, L. F. , & Duan, Y. F. (2020). Revisiting the phylogeny and taxonomy of *Osmanthus* (Oleaceae) including description of the new genus *Chengiodendron* . Phytotaxa, 436(3), 283–292. 10.11646/phytotaxa.436.3.6

[ece38777-bib-0031] Liu, Y. , Yan, H. F. , Cao, T. , & Ge, X. J. (2010). Evaluation of 10 plant barcodes in *Bryophyta* (Mosses). Journal of Systematics and Evolution, 48(1), 36–46. 10.1111/j.1759-6831.2009.00063.x

[ece38777-bib-0032] Lu, L. D. , Duan, H. Y. , Lu, Z. Y. , Li, X. W. , Duan, Z. Q. , & Sun, X. (2011). Studies on ITS sequences and systematic classification of *Osmanthus* . Pakistan Journal of Botany, 43(2), 759–771.

[ece38777-bib-0033] Manvelidze, Z. K. , Memiadze, N. V. , Kharazishvili, D. S. , & Varshanidze, N. I. (2010). Dendroflora of Adjara (Adjara floristic region). Annals of Agrarian Science, 8(2), 112–114.

[ece38777-bib-0034] Melia, N. , Gabedava, L. , Barblishvili, T. , & Jgenti, L. (2012). Reproductive biology studies towards the conservation of two rare species of Colchic flora, Arbutus andrachne and *Osmanthus decorus* . Turkish Journal of Botany, 36(1), 55–62.

[ece38777-bib-0035] Mohanta, T. K. , Mishra, A. K. , Khan, A. , Hashem, A. , Abd Allah, E. F. , & Al‐Harrasi, A. (2020). Gene loss and evolution of the Plastome. Genes, 11(10), 1133. 10.3390/genes11101133 PMC7650654 32992972

[ece38777-bib-0036] Nguyen, L. T. , Schmidt, H. A. , von Haeseler, A. , & Minh, B. Q. (2015). IQ‐TREE: A fast and effective stochastic algorithm for estimating maximum‐likelihood phylogenies. Molecular Biology and Evolution, 32(1), 268–274. 10.1093/MOLBEV/MSU300 25371430 PMC4271533

[ece38777-bib-0037] Niu, E. , Jiang, C. Y. , Wang, W. , Zhang, Y. , & Zhu, S. L. (2020). Chloroplast genome variation and evolutionary analysis of *Olea europaea* L. Genes, 11(8), 1–12. 10.3390/genes11080879 PMC7463426 32756391

[ece38777-bib-0038] Olofsson, J. K. , Cantera, I. , Van de Paer, C. , Hong‐Wa, C. , Zedane, L. , Dunning, L. T. , Alberti, A. , Chrisin, P. A. , & Besnard, G. (2019). Phylogenomics using low‐depth whole genome sequencing: A case study with the olive tribe. Molecular Ecology Resources, 19(4), 877–892. 10.1111/1755-0998.13016 30934146

[ece38777-bib-0039] Piot, A. , Hackel, J. , Christin, P.‐A. , & Besnard, G. (2018). One‐third of the plastid genes evolved under positive selection in PACMAD grasses. Planta, 247(1), 255–266. 10.1007/s00425-017-2781-x 28956160

[ece38777-bib-0040] POWO (2021). Plants of the World Online. Facilitated by the Royal Botanic Gardens. Published on the Internet. Retrieved from http://www.plantsoftheworldonline.org/

[ece38777-bib-0041] Qu, X. J. , Moore, M. J. , Li, D. Z. , & Yi, T. S. (2019). PGA: A software package for rapid, accurate, and flexible batch annotation of plastomes. Plant Methods, 15(1), 1–12. 10.1186/s13007-019-0435-7 31139240 PMC6528300

[ece38777-bib-0042] R Core Team (2021). R: A language and environment for statistical computing. R Foundation for Statistical Computing. Retrieved from https://www.R‐project.org/

[ece38777-bib-0043] Rambaut, A. , Drummond, A. J. , Xie, D. , Baele, G. , & Suchard, M. A. (2018). Posterior summarization in bayesian phylogenetics using Tracer 1.7. Systematic Biology, 67(5), 901. 10.1093/SYSBIO/SYY032 29718447 PMC6101584

[ece38777-bib-0044] Revell, L. J. (2012). phytools: An R package for phylogenetic comparative biology (and other things). Methods in Ecology and Evolution, 3(2), 217–223. 10.1111/J.2041-210X.2011.00169.X

[ece38777-bib-0045] Ronquist, F. , & Huelsenbeck, J. P. (2003). MrBayes 3: Bayesian phylogenetic inference under mixed models. Bioinformatics, 19(12), 1572–1574. 10.1093/BIOINFORMATICS/BTG180 12912839

[ece38777-bib-0046] Rozas, J. , & Rozas, R. (1995). DnaSP, DNA sequence polymorphism: An interactive program for estimating population genetics parameters from DNA sequence data. Bioinformatics, 11(6), 621–625. 10.1093/Bioinformatics/11.6.621 8808578

[ece38777-bib-0047] Sachse, M. (2001). *Oleaceous laurophyllous* leaf fossils and pollen from the European Tertiary. Review of Palaeobotany and Palynology, 115(3–4), 213–234. 10.1016/S0034-6667(01)00070-7 11440770

[ece38777-bib-0048] Sauquet, H. , Ho, S. , Gandolfo, M. A. , Jordan, G. J. , Wilf, P. , Cantrill, D. J. , Bayly, M. J. , Bromham, L. , Brown, G. K. , Carpenter, R. J. , Lee, D. M. , Murphy, D. J. , Sniderman, J. M. K. , & Udovicic, F. (2012). Testing the impact of calibration on molecular divergence times using a fossil‐rich group: The case of *nothofagus* (fagales). Systematic Biology, 61(2), 289–313. 10.1093/sysbio/syr116 22201158

[ece38777-bib-0049] Shimono, A. , Zhou, H. , Shen, H. , Hirota, M. , Ohtsuka, T. , & Tang, Y. (2010). Patterns of plant diversity at high altitudes on the Qinghai‐Tibetan Plateau. Journal of Plant Ecology, 3(1), 1–7. 10.1093/jpe/rtq002

[ece38777-bib-0050] Srivastava, R. , Wheeler, E. A. , Manchester, S. R. , & Baas, P. (2015). Wood of Oleaceae from the latest Cretaceous of India – the earliest olive branch? IAWA Journal, 36(4), 443–451. 10.1163/22941932-20150113

[ece38777-bib-0051] Sun, H. , & Li, Z. M. (2003). Qinghai‐Tibet plateau uplift and its impact on Tethys flora. Advance in Earth Science, 18(6), 852–862.

[ece38777-bib-0052] Sun, H. , Zhang, J. , Deng, T. , & Boufford, D. E. (2017). Origins and evolution of plant diversity in the Hengduan Mountains, China. Plant Diversity, 39(4), 161. 10.1016/J.PLD.2017.09.004 30159507 PMC6112316

[ece38777-bib-0053] Torfstein, A. , & Steinberg, J. (2020). The Oligo‐Miocene closure of the Tethys Ocean and evolution of the proto‐Mediterranean Sea. Scientific Reports, 10, 13817. 10.1038/s41598-020-70652-4 32796882 PMC7427807

[ece38777-bib-0054] Wadgymar, S. M. , Ogilvie, J. E. , Inouye, D. W. , Weis, A. E. , & Anderson, J. T. (2018). Phenological responses to multiple environmental drivers under climate change: Insights from a long‐term observational study and a manipulative field experiment. New Phytologist, 218(2), 517–529. 10.1111/NPH.15029 29451307

[ece38777-bib-0055] Wang, D. , Zhang, Y. , Zhang, Z. , Zhu, J. , & Yu, J. (2010). KaKs_Calculator 2.0: A toolkit incorporating gamma‐series methods and sliding window strategies. Genomics Proteomics Bioinformatics, 8(1), 77–80. 10.1016/S1672-0229(10)60008-3 20451164 PMC5054116

[ece38777-bib-0057] Wu, Z. Q. , Gu, C. H. , Tembrock, L. R. , Zhang, D. , & Ge, S. (2017). Characterization of the whole chloroplast genome of *Chikusichloa mutica* and its comparison with other rice tribe (Oryzeae) species. PLoS One, 12(5), 1–17. 10.1371/journal.pone.0177553 PMC5443529 28542519

[ece38777-bib-0058] Xiang, Q. B. , & Liu, Y. L. (2008). An illustrated monograph of the sweet *Osmanthus* cultivars in China. Zhejiang Science and Technology Press.

[ece38777-bib-0059] Xing, Y. W. , & Ree, R. H. (2017). Uplift‐driven diversification in the Hengduan Mountains, a temperate biodiversity hotspot. Proceedings of the National Academy of Sciences of the United States of America, 114(17), E3444–E3451. 10.1073/pnas.1616063114 28373546 PMC5410793

[ece38777-bib-0060] Yang, Q. S. , Chen, W. Y. , Xia, K. , & Zhou, Z. K. (2009). Climatic envelope of evergreen sclerophyllous oaks and their present distribution in the eastern Himalaya and Hengduan Mountains. Journal of Systematics and Evolution, 47(3), 183–190. 10.1111/J.1759-6831.2009.00020.X

[ece38777-bib-0061] Yang, Z. H. (2007). PAML 4: Phylogenetic analysis by maximum likelihood. Molecular Biology and Evolution, 24(8), 1586–1591. 10.1093/MOLBEV/MSM088 17483113

[ece38777-bib-0062] Yao, S. B. , Jiang, D. B. , Fan, G. Z. , & Zhu, N. S. (2017). Seasonality of precipitation over China. Chinese Journal of Atmospheric Sciences, 41(6), 1191–1203. 10.3878/J.ISSN.1006-9895.1703.16233

[ece38777-bib-0063] Ye, W. Q. , Yap, Z. Y. , Li, P. , Comes, H. P. , & Qiu, Y. X. (2018). Plastome organization, genome‐based phylogeny and evolution of plastid genes in Podophylloideae (Berberidaceae). Molecular Phylogenetics and Evolution, 127, 978–987. 10.1016/j.ympev.2018.07.001 29981470

[ece38777-bib-0064] Ye, X. Y. , Ma, P. F. , Yang, G. Q. , Guo, C. , Zhang, Y. X. , Chen, Y. M. , Guo, Z. H. , & Li, D. Z. (2019). Rapid diversification of alpine bamboos associated with the uplift of the Hengduan Mountains. Journal of Biogeography, 46(12), 2678–2689. 10.1111/jbi.13723

[ece38777-bib-0065] Yuan, W. J. , Zhang, W. R. , Han, Y. J. , Dong, M. F. , & Shang, F. D. (2010). Molecular phylogeny of *Osmanthus* (Oleaceae) based on non‐coding chloroplast and nuclear ribosomal internal transcribed spacer regions. Journal of Systematics and Evolution, 48(6), 482–489. 10.1111/j.1759-6831.2010.00099.x

[ece38777-bib-0066] Zhang, J. Z. , & Yang, J. R. (2015). Determinants of the rate of protein sequence evolution. Nature Reviews Genetics, 16(7), 409–420. 10.1038/nrg3950 PMC4523088 26055156

[ece38777-bib-0067] Zhao, C. H. , Yang, Q. H. , Zhang, M. , Zhang, C. , Wang, X. R. , & Li, Y. F. (2020). Characterization of the chloroplast genome of the *Osmanthus didymopetalus* . Mitochondrial DNA Part B: Resources, 5(3), 2480–2482. 10.1080/23802359.2020.1778551 33457835 PMC7782516

[ece38777-bib-0068] Zhao, D. N. , Ren, Y. , & Zhang, J. Q. (2020). Conservation and innovation: Plastome evolution during rapid radiation of Rhodiola on the Qinghai‐Tibetan Plateau. Molecular Phylogenetics and Evolution, 144, 106713. 10.1016/j.ympev.2019.106713 31863901

