# Supplementary material for: Plastid genomes reveal evolutionary shifts in elevational range and flowering time of Osmanthus (Oleaceae)
Source: Ecol Evol. 2022 Apr 1;12(4):e8777. doi: 10.1002/ece3.8777 (PMC8975774; doi:10.1002/ece3.8777)
Supplement: Supplementary file 2 — Supplementary Material [file ECE3-12-e8777-s004.docx]

>Nestegis_apetala

TATCATTTTAGTTATGGGCGAACGACGGGAATTGAACCCGCGCATGGTGGATTCACAATC

CACTGCCTTAATCCACTTGGCTACATCCGCCCCTCTTAGATTTTTTTATTTTAAGACAAA

AGGTTGAATTTCGACCATTTCATTTATCTTCTTTCTTATTTGCTTATTTGTGAGATATTT

TTATCTCAGAGATAAAAAGATTAAGCAAAAATTTGAATTTTTCTCGTTTTCATTTAAATT

TAAATGTCAAAAAAACTTCGCAAAAGATGGATAGATAAATGAAAATAAAAATGCATATAG

AACGAACAAATAATACTCAATCATCAATCAACCCCTAAGAAAAATAATCCCCTTTATTTC

TTTTTCGGTAATGTAACGAATAAAAGTCTATGTAAGTACAATATGACTAAAAAAAGTAGT

AGTAATTAAAAAGAAAATAAAGGAGCAATAACGCCCTCTTGATAGAACAAGAAAGAGTTT

ATTGCTCCTTTATTTTC--TTTTTTTTTTCAATAACTCCTATACACTAAGATCGGGTCTT

ATCCATTTGTAGATGGAACTTCGATAGAAGCTAGGTCTAGAGGGAAGTTATGAGCATTAC

GTTCATGCATAACTTCCATACCAAGGTTAGCACGGTTAATGATATCAGCCCAAGTATTAA

TTACACGGCCTTGACTATCAACTACGGATTGGTTGAAATTGAAACCATTTAGGTTGAAAG

CCATAGTGCTGATACCTAAAGCAGTGAACCAGATACCTACTACAGGCCAAGCAGCTAGGA

AGAAGTGTAACGAACGAGAATTGTTGAAACTAGCATATTGGAAGATTAATCGGCCAAAAT

AACCATGAGCGGCTACGATATTATAAGTTTCTTCCTCTTGACCGAATCTGTAACCTTCAT

TAGCAGATTCATTTTCTGTGGTTTCCCTGATCAAACTAGAAGTTACCAAGGAACCATGCA

TAGCGCTGAATAGGGAGCCGCCGAATACACCAGCTACGCCTAACATGTGAAATGGGTGCA

TAAGGATGTTGTGCTCAGCCTGGAATACAATCATGAAGTTGAAAGTACCAGAAATTCCTA

GAGGCATACCATCAGAAAAACTTCCTTGACCAATTGGGTAGATCAAGAAAACAGCGGTAG

CAGCTGCAACAGGAGCTGAATATGCAACAGCAATCCAAGGTCGCATACCCAGACGGAAAC

TAAGCTCCCACTCACGACCCATGTAACAAGCTACACCAAGTAAGAAGTGTAGAACAATTA

GTTCATAAGGACCCCCGTTGTATAACCATTCATCAACGGATGCCGCTTCCCAGATTGGGT

AAAAGTGCAAACCTATAGCTGCAGAAGTAGGAATAATGGCACCTGAGATAATATTGTTTC

CGTAAAGTAGAGATCCAGAAACAGGTTCACGAATACCATCAATATCTACTGGAGGAGCAG

CAATGAAGGCAATAATAAATACAGAAGTTGCGGTCAATAAGGTAGGGATCATCAAAACAC

CAAACCATCCAATGTAAAGACGGTTTTCGGTGCTGGTTATCCAGTTACAGAAGCGACCCC

ATAGGCTTTCGCTTTCGCGTCTCTCTAAAATTGCAGTCATGGTAAAATCTTGGTTTATTT

AATCATCAGGGACTCCCAAGCACACTAATTTTCTATAATATAAATCGAAATAGAAAATGG

AAGGCTTGTTATTTAACAGTATAATATGACTTATATGGTCGTGTCAA-----CCAATCCA

ATAACTATCTAGATTTATTCGAATTTTTTGTAAATGAATGAAGTGGATTGCAAAAAGAAA

ATAAAGATTTCTATACATAAAATTTCGATATGACAGTGGGTTGCCCGGGATTCGAACCCG

GAACTAGTCGGATGGAGTAGAAAATTTCCTTGTTATGTTAATTAAATAAGGAAAAACCCC

TCCCCAAGCCGTGCTTGCATTTTTCATTGCACACGGCTTTCTCTATGTATACATCATTTC

CTTTCTTATAAAGACTTTAAAAAGTTGAATACTCAGTTGATTTAACCCTTATTACATACT

ACATCAACATTTCAGAATAGCGGAAATCACATTTTTATTTTATTATCTCTTCATCCATTT

AATTTAGGAAAAATTTCTATTTCCAAGCTTTCAGAATAATTATTTATGATTGACCAGATC

ATTGATACAAATAATATCCAAATACCAAATCCGACTTCTATATACTCCCCGCAAAGTGGA

AGAAGCTTTTGGGAAGGTCAAAGAAAGAACTTGTTCTTCCGACATAAGAAATTCTTCCAA

TAATTCCGAGCCTAATCTTTTCAAAAAAGCACGTACAGTACTTTTGTGTTTCCGAGCCAA

AGTTCTAGCACAAGAAAGTCGAAGTATATACTTTATTCGATACAAACTCTTTTTTTTGGA

AGATCCGCTATGATAATGAGAAAGATTTCTGCATATACGCCCAAATCGATCAATAATATC

AGAATCTGATAAATCAGCCCGAACCGGCTTACTAATGGGATACCCTAGTACGTTACAAAA

TTTCGCTTTAGCCAATGATCCAATCAGAGGAATAATTGGAACAAGAGTATCGAACTTCTT

AATAGCATTATTGATTAGAAATGAATTTTCTAGAATTTGACTCCGTACCACTGAAGCGTT

TAGTCGCACACTTGAAAGATAGCCCACAAATTCAAGGGAATGATTAGATAATTGGTTTAG

ATAAATCCTTCTTGGATGAAACCACAGCGAAAAATAACATTGCCAAAAAGTTACAAGGTA

ATATTTCCATTTATTCATCAAAAGAGGCGTCCCTTTTGAAGCCAGAATTGATTTTCCTTG

ATACCTAACATAATGCATGAAAGGATCCTTGAACAAGCATAGATTGGTCTGAAAATCCTT

AACAAAGACGTCTACAAGACGTTCTATTTTTCCATAGAAATATATTCGTTCAAGAAGAGC

TCCAGAAGATGTTGATCGTAAATGAGAAGATTGGTTACGTAGAAAGACGAAAATAGATTC

GTATTCACATACATGAGAATTATATAAGAAGAAGAATAATCTTTGATTCCTTTTTGAAAA

AGAAGAACTGGCTTTCTTTGGAGTAATAAGACTATTCCAATTACAATACTCGTGAAGAAA

GAATCGTAATAAATGCAAAGAAGAGGCATCTTTTACCCAATAGCGAAGAGTTTGAACCAA

GATTTCCAGATGGATGGGGTGGGGTATTAGTATATCTAACACAAAATTTAAATGTGAAAA

ATTGTCCTCTAAAAAAGGAAATATTGAATGAATTGATCGTAAATTATGAGATTTTACTAT

CTTTTTTTTTTTCCCTTCTAGAGAAAGTATTAATCGTAGAGAAAATGGAATTTCCACAAT

AAATGCAAACCCCTCTGATATGATTTGAGGATACAAATTCTTGTTGCGCCCCAAAAATTG

ATTTTGGTTAGAATCATTAGCAGAAATAAGAAAATGATTCTGTTGATACATTCGAGTAAT

TAACCGTTTTACAATTAGTAAACTGGATTTATTGTCATAACCTGGATTTTCCAACAAAAT

CGATCTATTGAAACCCTGATCATGAGCAAGTCCATAAATATACTCCTGAAAGATAAGTGG

ATATAAGAAGTCGTGTTGTTGAGATCTATCTAGCTGTAAATATCTTTTGATTTCCTCCAT

TTGAAATTTTATTTGAACCAAAGTTAGAAGAATTTTGTGGGTTATCAAATGATACATAGT

GCGATACAGTCAAAACAAGGTATTCTAGTAAGAATAGATACCTCGGAAACAGGTAAACTT

ATCAACAGATTCTCTACCCTCTCTTTTTTCCATTTCATTTAATTGGTCTATGTTATAGGA

TAACAAGATGGTTAGAAATCTTTTATTTTTTGAACCTAATCGCTCTTTTGATTTCGG--A

AAAAAACTTTCTTTATCAATATACTGCTTCTTTTACATACGCATCTCCATTCCATAATGG

AGAATGCCAATAGTTAGGATTCATTAAAAAAATATAGAATCCACTCATGGGGGGAGAAGT

CCTTGCCGTATCAGGCACTAATCTATTTTTAACGTCTAATTAGATCGGGAAATTATTCAA

ATTTAAGAACAGAAGCTGGTTGCTTTTTCTTTCCCATAATTAATTGAAGCCATAGGGCCC

TATCCATTTATTCATTCGACCCAACTTTTTTTTGTTCCGTTCCAAGAATTCGAACAGGGT

TTTGTACCGATCCGATAAAAATGAAATAAACTCAGAACTATCCGTTGATACGACATGCTA

TTTTTTCCATTCATTCCCTTTCAGGATCAGTCGCGGTCTTCCAAACTTTACCGATGGTAT

GGACGAATTCCTCGCTTCATCCAAATGTGTAAAAGATTCTAGCCGCACTTAAAAGCCGAG

TACTCTACCGTTGAGTTAGCAACCCGAAGAAAATAAAACATAATTTAAATATTCAAATTA

AAGGGTGTGAAGATACAATCTAAATCAATTATCAATTAAATAATGAGAAAATAAATTAGA

CGAGGTAATCAAACAATTGAGCTAAGAAATCTAAAAAAAAAAAAGATTTTCTAATGGATT

AGAAACATAAAAATGAATAGATCAGATCAAAATACAAGAAATTATCAGATAAAAGAAAAA

ATATATATATAGAGAGAGAATTGTCAAAATTGATAGAGTACCTCTTATGTTATCTAC--T

TTTTTTTTC--AATTAAAAACCTCTTGTATCAATATCAAAGAAAGGAATGAGGTAAAGTA

AAAAAACAAACCTATGGTACGGAAATAAATAGATCCACTTCACTGAATTATATTTGTTCG

ATACACTGTTGTCAATATAAATGTTGAGAAAATAATACAATGGAAAAAAACAAAAGAAAT

TTCATTGAAATCTTTTTTTTTTTAACTCAAAAAATTTCAATTTAACAATGAAATAATATT

CCAAATTGTCTTGGATTGACACTAGATATCTAATCTACATAGATACAAAAAAGTGTAGAT

GGGAGAATAAGAATTGAAAAAAAAAA--ATATTGGATTTTTAATGTATTTCATCAATCTA

AGTGGAATAAACAAACTGGATTCCTTGTTGGTTAGTCAAATTCCAACGAAAACCACATAA

TTGAAAGAAATGAAATGTCCGAATTTGAGATTTATATGATCAATAAACTAAGGTTTTGTT

GTTATCGACCATGGAATTCGACAGAAGCAATGAGAAATAGATACGAATAAAGCAGGATTA

AGGGGGGAAATAAAAAAATAGTAGAGAAAAGTTATACAAAGTTATATAAAAATCACTACC

CCCCCTTGGTATTTCTTTAATTGAATTTCGTTTGATTAGGTGGAAGTTCCTTAAAAACCT

CTGCCTTCTTTAAAATATCCTGAACAGTTCCTGTAGGTTGAGCACCCTTTTCAAGGAAAT

ATAGAATAGCAGGAACATTTAAATAAGTTTGATTCTTCAGTGGATCATAAAAACCCACTT

TTCGAAGATCTTTTCCTTCTCTTCGGGATCGAACATCAATTGCAACGATTCGATAGACGG

CTCATTGGGATAGATGTAGATGAACAATACCCCCCCCTAGAAACGTATAGGAAGTTTTCT

CCTCGTACGGCTCGAGAAAAAATGATTTGATTCTAAGTTTTGTCTATGTATGGAATTCTA

ATAAATGACAAATGAGCCTATAAAATCAATTAGACTATGATTTAAGTCTTTTTTT-TTCT

TCTTCCTTCCTGAAAATGAAAAAGAAACCATTCGTACTCATAACTCAAGTTGGATAACTC

TCAAATAGCTTAAAGGAAAAAATCTTTAATAAATTTCATTTATTGAGTGGTCTTTACCCC

CTTTTGTTTGTCTCGTTTAAAATTCTATTTTGATTCTTCAGTCTGATCCAGTTATTGAGA

CTATCGAGACAATTA-AAAGGGTGTTTCCTTGTTCTGGGATCCTTTATCTTTGTTTTAAA

TCATTGGGTTTAGACATTACTTCGGTGCTTCTTAATCCTTTCAAAATGGCAGCAACATAC

CCTTTTTTGTGATTTCTCTTTCTATCAAAGAATCCTACGGACAGTTGATTCCCGCGTGAT

ACACTTTGGATCGAAAACGTTTGATCAATTCCAACAGGTTTTGCTTTTGAATTGGAAACT

TGCTCAAATTGGATCCTTTCCATTTCTATCTCGAAGATATATTTACGAAGTTGTTCCAAT

TTATTGATTGGCATTAACCCTAGATCCTTGCCCCCAAGAAATGAATTAATCCTTTCCACT

CGAGCTCCATCGTGGACTATTTACAACCCAACAAAAAGAAAAAACGAAGGGTTCGAGTGG

AACAGAACAAACGATGTCGAGCCAAGAGCACCTTCATTCCTATATAAATATAAAATAGCG

GATGTAAAAATCCACAACGGATCTGTCCTTCAAGTCGCACGTTGCTTTCTACCACATCGT

TTCAAACGAAGTTTTACCATAACATTCCTCTAATTTGGAACCGGTATGGAATTGATTCAA

TCATGGAATCATGAATAGTCATTGGTTCAGTTGTACATAGACATCGCGTAATCTATACTT

TTTCTTCTGATATGTGTAAAGATTTTTCTGAAGAAGTCTTAGCAAGACACCATCTTTAAC

ATATATATAAAGAAATAGAAATAGATAAACGAATAAATAAGTTCTTTTTGAGTCTGCTAC

TTCAAGTGACTCAATAGGATAGATTGACCTATTTCCTACTTTTTGAGTACCAAAAATTCA

ACTTACAAGGAATCATTCAAAATTTTGATTAAAACTATTTCGAATGGAAAAAGGTTCCTA

TTTAGACATCATTAAAAGATAAGTCTTTTTTTTTTTAATTGACCCATCACTGATTTCAAA

TAGATAAATAGATCACAGAAAAGAAGAAAGAAATCCCA-TTTTTTTTCATGAGATGGATA

AAAAAACTGATGTAAGGTAAGATTTGAATCTTTATTCTTTTCATCTGATTGCGAAAATCC

AAATCGAAAAAATCGAGAGGGGTTTTCGTATCTATCAGATAGACTGAACAATTGTCACTG

TTATAGATATTCTATAACACTTAATTTAACTAATTTTAGTTTAAAAAATTTAAGGGATCC

CAAACCTTTTTCACAAAAACCGAAAGACTATTGTCATTGAAATAGAACGATACATATAAG

CTAAACATTTCCTCATTTTATATGCATTTTGTTTAACATGGGGTTGAGTAATATTTCAAT

AATCGAATCTTGTCATAAAGTGAATAAAAGGGATAGGATAAATCACCCCTGCCTCAATCC

AATCGTTACATAGATTTACAATTCTTCATTATAGCCAAAC-AAAAAAAATACCTAAATAA

AATAAAAAAACGAGATTCAAAGAAAATACATCGATTCCATAACATATAAACATATATAAA

TATGTTATTTGATCATTTGTTAATGAGATTTGGACAGATACAGATATTTAAATTAATACT

GATTATCTCCTATCCACTCCCCTAT-TCTTTCTATGGGAATGATAGAGC-AAAAAAAAAG

GAATCCTGATCCGGTATGGGAAATGACTTGTCTAGTTACAAAGACAAACAATAAGTCCAA

ATTTAGTCAAGCTTTAGTCTAACCCACTAAGAGACTTAGTCCTATTGATTGAATTTAATT

AGTATCAAGAACTCGCTCTTGTTTCGAATTCAGAGATCTCGAGAAAAATCTAATTACTAA

TTAGACTCCCTCATTT--------------------------------------------

------------------------------------------------------------

------------------------------------------------------------

------------------------------------------------------------

------------------------------------------------------------

------------------------------------------------------------

---------------------AATGGATATGGCTCTGGGACGGAAGGATTCGAACCTCCG

AATAGCGGGACCAAAACCCGTTGCCTTACCACTTGGCCACGCCCCATTATCAATTTCTAA

TCTACACTAAGAAACAATAATATTGTTATTGGTTGTTTGTCAACTCCAGTCCAAATATCT

ATAGAATAGATTATTTAGGATTTTAATCCATATAGATATAGAATTCAACTCAATTTATTG

ATCATTACATATAATTCAATTAAGATATTGTATGAAAGTATGATTTCTTCTATTCTCCTT

TGAGAATTGGAGGATTTTTGATTGGGTGGGTTCAAAGAAAAAGAAGGA-TTTTTGTATAC

CTTACTTCCTTTCTTCCTTTTTCCTTATATCAATAACTCAATCAAAATGCAATTATCTCC

AAGAACAAAATGTCTGTTATGCTTAATATCTTTAGTTTGATCTGTATTTGTCTTAATTCT

GCCCTTTATTCGAGTAGTTTTTTCTTCGGCAAATTGCCCGAAGCCTATGCTTTTTTGAAT

CCAATCGTAGATGTTATGCCAGTCATACCTGTGTTTTTTTTTCTCTTAGCCTTTGTTTGG

CAAGCTGCTGTAAGTTTTCGATGAGATCCTTAATAATATCCTAGAAGATTCATGATTTCT

TCGAGAAAAAATTCTCTAACAATTGATAAAACCAGATAAGTTTTAGAGTCTGAACCCTCG

ATTCACACATTGAAATTCTTGGATAGTCGCCATAAATCCGGCTTACCCCATTTCCTCCTT

TTTTTGACCCTTTTCCAGTGAAAGACCCTAACCCTATTTCTATTAGGGTCTCCCACAATA

TCGAATTTGGATATGAAAGAAATTTTTGTTAATGAAAAACTCTTATTCCAAATAAATTTC

TGACAATTCATTTCTATTTCTAGAAAAACCTCATTTCTTGGTGTCAAAATAGGATATGTG

GTAGAAAAATGGAAGATCTATTCTC-AAAAAAATCATCTTGGAGATTGTGTAATGCTTAC

TCTGAAACTCTTCGTTTATACCGTAGTGATATTTTTTGTTTCTCTCTTCATCTTTGGATT

CCTATCTAATGATCCAGGACGTAATCCTGGACGTGAAGAATAAAATCCAAAGGGTTTTTC

CTTGGTTAATTTTCAAATTTTCTTAGGATTTTTTCTATTCCACACGTTTAACTAAGATTT

CAAAAATTTGAAAAATAAATAAATAAATCAAGTCATCAACGGAACCGGAAAGAGAGGGAT

TCGAACCCTCGGTACGAATAACTCGTACAACGGATTAGCAATCCGACGCTTTAGTCCACT

CAGCCATCTCTCCCAATTGAAAAAGAGAATTACTACATTACACATAATGTAAGGAGTCTT

TCTTT-CTCTATTCTATAGAGATATACAAATCAGGAATTTCTTTTAGATTAGATAAAGGA

AGGGCTCGAACGAGCCTATAAATAAAAAAATAAAAATAAAG-AAAAAAAAAGAAGACATC

TTTGTGTTGATTTTGTTCGAAAGGCCCTTCTTATTCTGATGGCCTGGCCTGGTCAGTACC

TAGCCGGGCCCCTTTTTTTGTTCAAACGAATTATAGATAAATAATGATTTATTTGATTTG

AAAACAAAAATGCTTGTTATTTATTATATTCAATTGAATATAAAATATTCAAGCAACAAC

AAAAAGAAGAAAGACTATTTACATTCTTTTTCTTTTTCTATTTGAATTTTAGTTTCATTT

TCGGAACTAAAAAGGAGACTATCGATTTTTCCACAATGCATTTTTCTGTTATGATTTTAG

TGTTTTTTGTGATCCGTAGCTATCAAAACTTCTTAGCAAAAGAGAAAACTTTAGTTATTT

AATTTAAATAAAATGAACCCTCCTTTCAGAATCTCATTAAATTGTAAAC-CCCCCCCACG

AAAAATTTCAACACTCTAATTTTGATGATTCTTTGATGATCCAATCCTATCTTGATCATG

CCCAATTCTCCTGTTCGACAAAAGGTCCATTTGTATACAATAATCGCATTGTAGCGGGTA

TAGTTTAGTGGTAAAAGTGTGATTCGTTCTATTACCCCTTTAATAGTTAAAGGGTCTTTC

GGTCTTATTCATATTCCGATCAAAAACTTTATTTCTTAAAAGGATTTAATCCTTTACCTC

TCAATGAAAAATTCGAGAAAAAAATAAAAATTCTCGTGATTTCTATCCATGAGTCACTTA

GAAAGTGAAAAGTTGGATTATGAAATTGCGAAACATAATTTTTGAATTGGATCAAGACTT

CCAATTAAATAAGTATGAGTAAAGGATCCATGGCTGAAGATAGAAAGTCGATTTCTAATC

GTAACTAAATCTTCCATTTTTGATTTGTAGAGAAAGAAATTGAAGCAAAATAGCTATTAA

ACGATGACTTTGGTTTACTAGAGACATCGACATATTGTTTTAGCTCGGTGGAAACAAAAT

CCTTTTCCTTAAGATCCTCTCAAATAGAAATAGAGAACGAAGTAACTAGAAAGATTGTTA

TAATCACCTTCTTCTAGAAGGATCATCTAGAAAGCGATTCGTTTTGTCTGTATTCAGACA

AAAAGCTGACATAGGTGTTATGGGTAGAATTTTGTTCACATCTTAGATCTAGGAATTTAC

TCATCTTCCATAAAGGAGCCGAATGAAACCAAAGTTTCATGTTCGGTTTTGAATTAGAGA

CGTTCAAAATGCTGAATCGACGTCGACTATAACCCCTAGCCTTCCAAGCTAACGATGCGG

GTTCGATTCCCGCTACCCGCTTATATCCTTTTTCTAAATATTCGATTCGATATATTCTAG

AATATACTCTAATTAGAATTAGTGAATCATTGAATATACAATTCCAAAAAATTTCTCACA

TACAATCCGAT-TTTTTTTAACAAGAGGTAGAAAAGTCAAAATACG-AAAAAATCGGAAT

GAACAGCGTCCATTGTCTAATGGATAGGACAGAGGTCTTCTAAACCTTTGGTATAGGTTC

AAATCCTATTGGACGCAATTTATTTCCATATATTTTTGATTTCGATAGCAAGAAATACTT

T-TTACATAATTTGAATCCGAGACACTTGATTCC-TTTTTTTTTTAAGATAAAAGTGAGC

AATTTCTTTATGTTATGCTTGTTCCTGAAGTAGGAATCGGTCCGTTTGTTCCTGAATAGC

TTCTTTCAAAAGGACTTCTGCTTCCTCGGTAAATGTCTTAGTAGAAGATATGATTTCTTG

GAACTGAGGTTTATTAGTTTTTAAGTAAGTACGTAACTCAACAAGAAATTTCCTTACCTG

TCCAATTTCTAATGAATCAAGATAACCATTTGTTCCGGTATAAATAGTCATTATCTGTTC

TTCTACCGTGAGAGGAGCTGCTTGGGATTGTTTAAGCAATTCACGTAATCGTTGACCTCT

TGCCAATTGATTCTGAGTAGCTTTATCGAGATCCGAAGCAAATTGTGCAAAGGCTTCTAA

TTCTGCGAATTGCGCCAGTTCCAATTTTAATTTACCAGCTACTTGTTTCATGGCTTTAAT

TTGAGCTGCAGACCCTACTCTGGAAACGGAGATACCCACATTAATAGCAGGTCTGATTCC

AGCATTGAATAGATCGGCGGATAAGAATATTTGTCCATCAGTAATGGAAATTACATTAGT

AGGAATATAAGCCGAAACATCTCCCGATTGAGTTTCAACTATTGGTAAGGCGGTCATACT

TCCTTCACCTAAACTAGAACTTGATTTAGCGGCTCTTTCCAAAAGGCGTGAATGCAAATA

AAAAACATCCCCTGGATAAGCTTCACGACCAGGCGGTCTTCGTAATAGAAGAGACATTTG

GCGATAAGCTTGGGCTTGTTTGGAGAGATCATCATAAATGATTAAAGTGTGTTGTTTACG

GTACATAAAATATTCAGCCAAAGCTGCTCCTGTATAAGGAGCAAGGTATTGTAATGTAGC

AGGGGAATCCGCCGTTTCGGCTACCACAATAGTGTATTCCATCGCCCCCCTTTCCTGTAA

AGTAGTTACTACCTGAGCCACAGAAGATGCTTTTTGACCAATAGCTACATAAACACATAT

TACATTTTGACCTTGTTGATTGAGAATCGTATCTGTGGCTACTGCTGTTTTACCGGTCTG

TCTGTCCCCAATAATTAATTCTCGCTGACCACGTCCTATAGGGATCATCGAATCAATAGC

AATAAGCCCGGTTTGAAGAGGCTCATATACGGAACGCCGCGAAATAATACCTGGAGCGGG

AGATTCAATTAATCGAGATTCCGAAGCTGAAATTTCACCTCTACCATCAATAGGTTTAGC

CAGGGCATTTATAACACGACCCAAATAGGCCTCGCTCACTGGTATCTGAGCAATTCTTCC

TGTTGCTTTTACAGAACTTCCTTCTTGTATCATTAAACCATCACCCATTAATACAACACC

AACATTATTTGATTCCAAATTCAAAGCAATACCTATTGTACCTTCTTCAAATTCTACTAA

TTCACCCGCCATTACTTCATCAAGACCATGAATACGAGCAATGCCATCGCCTACTTGAAG

TACGGTACCAGTATTTACAATCTTTACTTCTCTATTATATTGTTCAATACGTTCACGGAT

AATATTACTAATTTCGTCGGCTCGAATGGTTACCATGAGTATTTCTTAATTATTTTTTAT

TTTGAAAAGAAAAAAAAATAATGCCTATAGTAGAAAGACTAATCAGTTATTTCTTTCATC

GCTCCCAACATGCCAATATTAGCACTAATGGTACGTAAATGTAACTCGTTGTTTAAACAA

CTATTCAGAGTTCCTAAAGCTCCTTGTAAGGCTTGTTGGAAAACCCGTTGTCGGACTTGA

TTAATCGCCCTTTGCTGTTCAAACTGAATCGTTTCATTTTTGTAATTTTCTAATTGTTCC

AAAGTCTTAGAAGTTGAATTAATCAAATTCCATTTTTCTCGCTCTATCTCAGAGTATCCA

TTCACCCGAAACTGATCTGCTTCCATTTCCACTTTCCGTAAGCGAGCCCGGGCTTTTTCC

AGCTGTTCAATGGCCCCCCCACGCAGTTCTTCTGAATTTCGAATAGTATTCAAGATCCTC

TGTTTTCGATTATCTAATAAATCACTTAATGAAAGTAGATTATCTTTCCATTCATTTCAA

AACTTCCATAATCCCTTCCCGAACCAAACATGAATCTTTCGATTCATTTGGCTCTCACGC

TCAATTACTTAGGGTAAATTCTCATA--TTTTTTTTATGAATGTAATGAGCCTATCCTCT

CTTCTTTATTCATATT-AAAAAAAACTTAATCTAATGCAAAACCAAAATACTCGGAGGAC

TCTTCTGACAAAATAAAAAATATGTAATTGTCAGCAAAGTTGTTTCTTTTTTTTCTTCAC

TTCAAATCCAAAAAATTCGTCTGACTTATACATAAGACATAGGTCGTCGATTCAGCATTG

GATAAAAGAGGGAAAATGCCCATTTTAGAATAAGTGGTTCAAATCATTTTATCGAGATGA

GTGTTCTATATCGATAAAATATTCATTTGAAAACCATCACTATATTAACATAGTGGTAGA

AAGAGTACCATGCCGCATCTAGACTTCAAACGGTTTGCTTTAACCATCTTAACAGTCCCA

CATTATTGGTTCCTAGAGAATCAAAGTAGATTTGCCAATTAATCACGAAATGCTATGGTT

CTTACATATAATTTATTAATTTCTTCAGAAGTAATTCGCGAGATCATGCACCTCTCTTTC

CTAGTTATAAAGGAAAAAGGTCCAGCTGGTTGGATCCAGCCTATTCTTGAAATAAACAAC

TCACACACACTCCCTTTCCAAAAAAGATCAATACACCAATCACTACACTTAGATTTATTG

GATTTGTTGCTAAAATATCGGTATTAAATCCGAAACTCCCGGCAGATGGCCAGTGGCCCA

AAGAAACGAAAGAATCGGTTACATTTTTCATAGGATCTCCTCTTATAGATAGACTCAAAA

ATAGATCAGAGTTCTTTTTGTATCACTTTGCCTATTTATTCTTTTCTATCGAGTCAAAAA

ATATTCGAGTTATAAAGTATGAACTACCCCCTTGCTGTTGCAACACCTCCT--AAAAAAA

AAGAGTTTCCCTTGGACTACGAACGGGAAAGGATGAAAGCGAGTCGGTATGCTAATTCCT

CATCTGCAAATCAGCCCTTCCCGTAGGGTATTTTCTCAACGAATAAGTAATTGTAGGAGT

TAAATCTTGATATAATTTGAAAAAGCAAACAAGAGGTCCAAGGCAATAAAATATGAAAAA

TATGTATTTTTCATATTTCTAAGATTAAACAAAAGGATTTGCAAATAAAAGTGCTAATGC

TACAACCAATCCATAAATTGTTAAAGCTTCCATAAAAGCTAGACTAAGCAATAGAGTACC

TCGTATTTTTCCCTCTGCCTCGGGCTGTCTCGCGATACCCTCTACAGCTTGACCCGCAGC

AGTCCCTTGACCAACTCCAGGTCCAATAGAAGCAAGCCCTACGGCCAATCCAGCAGCAAT

AACAGAAGCGGCAGAAATCAGTGGATTCATGATAAGTTCCTCGTACCAAAAAAAAAGAAA

TGGTTAATGATACAATCAACCAATGAATTATGACTTAATTATTCCATCGGTAGGATTCAT

CCAGTCGAAGTAACTAACAACTTCGAATTGAAGTAATAATATTATTGAATCATCAGAACT

ATTTCGATATCTCTATTTTTCGTTTCTATCCACAGAATCTTTGTGAATCCATA-GACTTT

CGTCCTTGCATTTCTTGGTTCCGAACTGTTATTTCAATTCTTCCATCTTTTTCTTGATTT

CATCCCCTTATTCAGTCAATTCACAGTCACAACGAGATGGAAGGACTTCTATTGGAATCC

ACATACAGTAGTAGAGAAAATGAAATATATCTTTAGTTCATATATAACTAGTCAATATCT

AATATCACATATACATGTCTTTCTTCCCTAACGTAAACCAAGCATTCATCTTAGATTCAA

TCGAATTCGAGAATCAATCGTCGAAACATCTACAAGAGTTGGCTTAGAACCATTCAAATA

CATATATCTAGTTCGACTCCCCTCTTCGAATCAATAATTAATCCATTTTTCAGGAATCCC

GTTTTTTGTAAATTATTTTCTCCCTTCCACTTTCTTTATCATTATTCCACCCGAATACAA

TCTAAATGAGCAAATTAATTAGGGCGAATTATTGCATATGCATAGAAATATCATTATTTG

ATTGATCTAAGTTCATGCAATTTATTATTTATTAATATTTTCTTTTGGTCAATTGTTGAA

TAAAATAAACTGAAAGTAGAATAGTTTCTCGTGTTTTTTATTTAATTACACGTGGTAAAC

ATATATCTTATTCCAATTTTGATTTGAATAACAAGATTGGCTGACCAATATAAAGCGAAT

ATTCGTTGAGGGAAGGTATGATAGTAAGTGGACGAAAAGGGAATTTTAGGAAAAAGATCT

AGATCTCTTTCCCCTTT-TTTGTTCCTATTACTTTCTATTTTCTATCGTATATAGCGTCG

TTGTATATTTCTATTCCTTAAGTCAATTATCTTGAGCCACACATACATTGCTTTGCATTA

AGCT--AAAAAAAGACTATTTCAATGATGGCCCTCCATAGATTCACCTATATAAGCCGCG

GCTAAAGTTGCAAAAATAAGAGCTTGAATACCACTTGTAAATAATCCAAGGAACATGACA

GGGATAGGAACCACTGAAGGTACTAAAGAAACAAGAACAACAACTACTAATTCATCCGCT

AAGATATTCCCGAAAAGTCGAAAACTAAGTGATAAGGGCTTTGTGAAATCTTCTAAGATG

TTAATGGGTAAAAGGATTGGAGTTGGTTGAATATATTTCCCGAAATAACTTAATCCTTTT

TTGGTAAGACCCGCATAGAAATATGCCACTGACGTGAGTAAAGCCAAAGCAACGGTAGTA

TTTATATCATTCGTGGGTGCGGCTAACTCTCCATGAGGTAATTGTATGATTTTCCAAGGT

AAAAGAGCTCCTGACCAATTAGAAACAAAAATAAATAGAAACATAGTTCCAATAAAAGGA

ACCCAAGGGCCATATTCTTCTCCAATTTGAGTTTTACTCACATCTCGAATGAATTCAAGA

ACATATTCGAAGAAATTCTGACCCCCAGTCGGAATGGTTTGTGGGTTCCGAACAGCTATA

GTAGCTGAACCTAATAAGATAGCAATTACAACCCAAGAAGTAATAAGTACTTGGCCATGG

ACTTGGAAACCCCCTATTTGCCAATAGAAATGTTGGCCTACTTCCACAGCGGATATATCG

TATAACCCTTTTAGTGTGTTGATGGAACATGATAACACATTCATATTGCCCTCTG-AAAA

AAATCGAACTTTAAACAAAATTATTTTGATTCCACCATCTCTTTGCCTACTTGAATCGGA

TATTTGAAATACCAACTAATATTTGGAATACTAACTAATCACATAATATCCCCAGTTATT

TTTATCTCTTTTTAATTCAGAAATAATAACCGATTCCATAAATCTATCGGATTTTTGAAG

TCAATTATTTATCTTATTATTAATCAAGGATTTCTTATATAGCTAGAACGACCCTCACAA

ATTGCGAATACTAATTTGTTAAGAATTAAGCGGATTGAAGATATAGCGTCATCATTCGCT

GGAATCGAAATATCTGCAAGATCAGGGTCACAATTTGTATCGATTAAACAAATTGTTGGA

ATTCCCAAAGTGATACACTCTCGCAGGGCCGTATATTCTTCGTGCTGATCAACGATAATT

ACAATATCGGGTAACCCTGTCATATATTTAATCCCGCCCAGATATGTTTGCAAGCGAGAT

AATTGCCTTTTCAACATAGCTGCATCCCTTTTCGGAAGACGGTTGAGTCTTCCTGTTTTT

TGTTCCATTCTCAAGTCCCTGAACTTATGAAGTCTCGTTTCTGTAGTGGACCAATTCGTT

AACATACCGCCGAGCCATTTTTTATTAACATAATGACACCGGGCCTTTATCGCAGCCCAT

GCTACTGAATTAGCTGCTTTATTTTTGGTACCAACAATTAAGAATTGTTTTCCCCTACTT

GCTGCATCAAAAACCAAATCACAAGCTTCGGATAAGAAACGAGCAGTTCTAGTAAGATTT

GTAATATGAATACCTTTACGCTTTGCAGAGATATAAGGTGCCATTTTAGGATTCCATTTC

CTAGTACCATGGCCAAAATGAACTCCTGCCTCCATCATCTCTTCCAAATTGATGTTCCAA

TATCTTCTTGTCATTTCCCCCCCCCCAAAAAAAAAAGAAAGGAGGAACTCTGAACTGAAA

TAAATAATTGTTCCGATGGAACCTTCTCTTCTACCGTAGATTGGCCGTAGATAGACGAAC

AAGCCATTACTTTTCTATTCATTATTATTTTGATTACCAAATCAAATGACTGCACCAAAT

ACAGATAGTCAAAAAGATGAATCTGCTCTTAGGAATCATTAAATCCTATAAATGATTGTT

CTGATGTATCATGGAAATTCTTTGAAAGACAAGAATCAAATAATTTTCTGTGGTGAAACA

AAATATCTCTCATTTCCCCCTCGAATAGATTGTTTTTTTTTGTTTCCAAAAGACTGTTGT

TATCTTGTTTTGAGGGGGGCACTAATCCTTTAAATCCGGTACCAAGGGGTATCATACCCC

CCAAAACAACGTTCTCTTTCAGGCCTTTCAACCAATCGATCCGACCCCGGAGAGCTGCTT

TTGCTAAAACTCGAGCAGTTTCTTGAAAACTCGCTTCAGATATGAAACTTTGAGTATTGA

GAGATGCTCTTGTTATTCCCAATAAGACGGCTCGGTAACAGACCGCTTCTTCCAAAGCGC

GCCCCATTCGTTCCGCTCGCAATAATCCAATTAATTCTCCGGGTGAAAAAACATTAGACA

TTCCATCTTCTGAAACCAACACTTTTGATGTTATTTGGCGTACAATAATTTCTATATGCC

TATTATGAATCTGCACCCCCTGGGATCGATAAACCTTTTGGATCTTATTAACCAAAGAGA

TACGACTTTGCACTATAGTTAGCTCAGCACCAATCAAGAAGCCCCACGGCATTCCAAGAA

TTCTTGTTATACGTTCGTTCCAACCCTCAACCCTCTTTTCTAGATTCATCGATATTGAAT

CAACCGAACGCACTTCTAACACCTGTTCCACTTTTGGAAGCCCCTGGGTTATATCACCAG

ATCTCGATTTTTCATATATAAATGTAATTAATGTATCTCCTTCGTACAGGATTTCCCCAT

AATGGCCATGAACAGTTGCTCCTGGAGTGGCCAAATAAGGCTTAGCTGATCGTATTACTA

CAGAATCAACTTGAACAAGTATAACTTGACCCGATTTTAGGTGTGGTGCATTTTTGGCTA

TACATACATTTTCACAAATAAACTGTCCAAGACTAATTATTGTAGATGTTTCTTGACAAT

AATTGTCATGGAGAAAATACCAATTCAAATTGAATGGATTCAAAATAATGTTACTGCCTG

GCTCGGGGTTATAAATTTTATCATTTTCATCCATTAAATAATATTTAATTACTTGAAAAG

TCTGTTTTAAATTGTCAAGTTGCAAATAGTTATTTACCAAGATCTGATTATGAGTTATTA

AATGGTAAAATGAATAAAAATTCACAATTGTAAAGGCTGTTCCAAAAGGCCCCAGCGAAT

TCTTAATTGGAATTCGAGGATCCTTTGTAATTTGAATTGATTCTTTTATCACATTGTGAT

ATTTTATTACATCGTTGAATGGACCCATTCGAAAACAATTGGATGATGACAAAATTATCA

ACGATTGGAATCCCGTATTTCTATTCAACAACGTATGAATAGTTCCTTGATTTTGGTTAA

GGGATTGTTGAATTCTTGCCTTGGAATAAATGGAAGAAAACGGATTGATATTGGTGCAAT

CTGATCCATTATCAGAGAGCAATCCTGAACCCGATGGATCATTCCTTTTTCCGATATATG

AAATAGGGGATTTCACTAAGTCGATTCTTAGGAAATGTCGAATCAAACCATTTGTCCTTA

TTTCAACAAAGGAAGCACGGGCTTCTTCACTAGAAGAACTTTTTTTTTTGTCTTGGTCCC

AATTCAATACTAAACAAGTCCGAACTAATTGAATATCTGTATCAGAAATTCCTCGAATGG

GTTTGCCATTTCCATAAAGGATATAATTGACAACTCGAAGTTGCACATTATCCCTTTCCT

GCAACAGATCCGGGGGAAAAAGTGTTGCTAAATTTATACCGTCCGTTATTTCATATGTGA

CGACAAGTCGAACCAAAACAAAATACTTTTTCTTACTAGGTGTGATCCGTTGAACATAGA

TCCAATTTTTCCATTTTTTTGATTCCTTGGAATTTCGTTTTCCTGTTCCTGGTGGTATCA

AAACGCCGCTATGTCGAGATATCTTATCGGTCTCTCCAGGAAAATGGATATCTCCAGAAA

AGATTTTAAGTTCTATTCTTTTTTTTTTTCTCTCCACTCGGACAAACCCGCCTACTCGGC

TTCTTATATTTAAAGTAATTTGTGTATCTACCCCAATGAGACTATTGTTCCGTACCATTA

TGGAAGAAGATCCGGGTAAGATATGCACTTCCTCGGGAATAAAAAAAAACCGATCTACTT

TCATTTGGTATTTTGGCCTAAATGCCTTGACTCCTCGATACTCAATCAAATCCTCTTTTT

TGACGATTGAATGCATTTCTACAGTCCCATATTTAGTAATGCCCGAACTCTTTCTTCTGT

ATCGAGGATCATCAAAATAAGCAAGAATACTATTTCTACGGAAAATGCCATTTGGAGGGA

TTTCAATCGAGATACCTGAACAGGGCATTAGTTCGTTCTCACGTTCTTGAATCGATTGGA

GTGGAATGATGAATCTATTTCTCCGCCTCTTTGAGAATAAATCAGAATTCTGGTGGAGAA

TGGTCGGATATATGAGATTACAACGACCATTACATCTAATTCGGTTAAGGTCCGAATAAT

CAGAAATCCTATCTTCTTTTTTTTTTTTACCATAAAAATACGAACTAAAGACTTTTTGTC

TCTCCGGATCATTAGTTTCTGAGAGGTTAGAAGTATATCTTCGCTTGACAGAACGAGAAT

GCGCGTTTATTTGATCTTGATCCTTGTGGAGCGAAAGTGAGACTAGACTGGATCTGCACG

GCCCTCCTAATAATATCCATAAATGACTTGTTTTTGGTAATAGATGAACATTACCATATG

TAAATTCAGGTGGATGATACACATCAGTACTCCAGTGCATTTCTCCGTCTGAGTCAGAAT

AAATATGTTTTCGAACCTTCTCTTTAAAATTCAAAGTGGATGTTCCTGCGCGAATCTCAG

CAATCACTTGTTCTGATTCTACATATTGATCGTTTTGAACTAAAAGAAAACTTTTGGATG

GAATATTTACATTATGTAGAATATCTTCACTCTCAATAGTTACATACAAGTCTATAGAAC

ATAGAAAGGCGGGATGTCCATGACGTGTACGTGTCGGATGAACCAAATCCTCATTGAATT

TGATTTTTCCATTAGAAGGGGCCCGCACATGTTCTGCAGTACCCCCCGTGAATACTCCGC

CGGTATGAAAAGTTCTTAATGTTAATTGAGTACCCGGTTCTCCAATCGATTGTCCTGCAA

TAATACCTACAGCTTCTCCTAATTCAACCAGGTCGCCATGAGTAGGACTCCGACCATAAC

ATAATCGACAGATCCAAGACGCACTCCTACAAGTAAAGGGAGTTCGAATAGATATTGGTT

GTGCTCGAAAGGTTATGAATCGATTTACAAGTCCAATCCCAATGTCTTGATTTCTACTGG

CAATACATCGTGTGCCCGCATATATATCATCTGCTAATACACGACCCATTAATGTTTGGA

TCAAAATCCTTTCCGGCATCATCCCATTCCGAGGACTCACAGAAATACCCCGGATGGTGC

CACAATCTGTCCGACGTACAACAATGTGTTGAACTACTTCAACAAGTCTGCGCGTGAGAT

ATCCAGCATCGGATGTTCGTACAGCAGTATCCACAACCCCTTTACGGGCTCCGTAGCAAG

AAATGATATATTCTGTTAAAGAGAGTCCTTCGCGTAAATTGCTTTGAATGGGTAAATCAA

TCATTTGTCCTTGAGGGTCCGACATTAAGCCTCTCATACCTACTAATTGATGTACCTGAG

ATGCATTTCCTCTAGCTCCCGAAAAAGACATTATATGAACTGGATTAAAGGGGTCAGTCA

TCCTAAAATTAGGATTCATTTCTTGTCGCAAATATTCACTTGTAGCATACCATATTTCAA

TGGATTGGCGTAATTTTTCTACCGCGTGTACATTCCCATAATGATGGTGTTTTTCCAAAA

TCAAACTTTGTTGTTCAGCATCTTGAACTAGCCATCGCTTAGAAGGTATTGTTAAAAGAT

CATCAATTCCTAATGAAATGGATGTAGCAGTAGCTTGGTGGAAACCCAGAGTCTTTACTT

GATCCAGGATGTGTGATGTATATGCCATTCCGAAGTGATCTATTAATCTACTAATAAGTC

GTTTCATGGCAGTTCCATCTATCACTTTATTGTGAAATACCAGATTGGCCCGTTCTGCCA

TAAGTACCTCCATATTCTGCTGAGTACGATTCGACAATGGGTTTGAGTCAGTGATTGGAA

AACTTCCTTTTCTCGATCTTGATTCGCATAGAAATTCCGGAACTATGGGCCTAGTTGAAC

TGAAGAAACCCGAATTCCTACTGGTATTATAGAATTCCTTAACTTAGTTCGGTACCATAT

GAACAGGCCTGAGAAAACCCCTGTACGGCTTCTTCGATTTCTCGATAAAGAGAAATATGA

CCAACAGTGGTTCGGATGTATATAAAAAGAATTTCTTTTTTTATACTTCTTACTATTAGA

TAGTGTCCATAAATCTCATAATAAGTACCTAAAGATTCATAGTGAACTTCGATGGGACTT

TCTCTTGAAGCAATAACGCGTTGATCTAGTCGCCACCGGAGCCACAAAGGACTATCTAAA

TTGATTCGTTTCTGCCGATAAGCTCCAATTGCATCATAGGAATTAGAAAAAAAGGGTTCT

TTCGTATACTTATAGTTATTATTGTTACTTCTTTGATTTTGATAGTTTCTGCGATTCCAC

GGATTATACCTATTTACACAAATACCTCGACGATTCCCGCTCGTTAATACATAGAGTCCA

ATAAGCATATCTTGAGTTGGTATGGAAATGGGATCCCCAATAGCTGGAGACAAAAGATTC

ATATGAGAAAACATAAGTAAACGGGCCTCCGCTTGAGCCTCCAAAGATAAAGGTACATGA

ACAGCCATTTGATCCCCATCAAAATCTGCATTGAATCCCTTACAAACTAATGGATGTAAA

CAAATAGCACGCCCCTCCACTAAAACGGGCTGGAATGCCTGTATGCCTAATCTATGCAGA

GTGGGTGCTCTATTCAGCAATACAGGATGCCCCTGCATAACTTCCTGAAGTATTTCCCAT

ACAATCGGTTCTTTTTCCCGAATTTTACTCTTAGCAACTCCTATATTCGAAGCAAGATGT

TGTCTAATTAGACCACGAATTACAAATGTCTGGAAAAGCTCTATTGCTATTTCGCGCGGC

AATCCACATCGATGTAATGAAAGTGAAGGACCCACGACAATGACGGAACGACCTGAATAA

TCAACCCGTTTCCCCAGCAGAGTCTCACGAAATCTTCCCTCTTTGCCTTCAATTACATCT

GAAAATGACTTGTAAACTTTATTATGACCGTCCCTCATTGGTTGTCCACGGATTCCATTA

TCAAGAAGTGTATCCACGGCTTCTTGTACTAATTTCTCCTGACACATTACTAATTCTCCT

GGCGTGGATTTACTTGTTGTTAATAGATCGGTAAGAGTATTGTTCCGATAGATAACTCTT

CTATAGAGTTCATTAATATCCGAGCTCATTAGTTTACCCCCATCTATCTGAATGATGGGT

CTCAACTCAGGAGGAAGAACTGGTAATAAACACAAAACCATCCATTCTGGTTCTATATTT

GTTCGAATAAAATGCTTAGCCAATTCCATGCGTCTAACTAAAAAATCCTTTCTTCTTCCA

ACCTTTCGATCTTCCCATTCATTTCCTGTGGGTCCTTCTTCCCCTAATTCTTTCCATTCT

ACCAACGAATTATCTAGAATAATTCGTAAATCTAGATCGGCTAATTGTTCTCGGATAGCA

CCTGCACCAGTAGAGATTTCTCGATTGCGAAATGTATCGAAGCCTTGGGTAGTAAAAAAC

AGCGGGATGCTGTATTTCCAAGATTGAATTTCATATTCGAATAAACCTCGTAATCGTAAG

AAAGTGGGTTTTTTAGTTATGGGCCTAGCAAAAGAAAAATTGGGATAGGATCCTATAGGA

TCTCCCCCCTTCAAAATCGGACGTGAAAGTTTCCTTTCATCCGGCTCAAGTAGGTACACC

AAATAAAGAAAGGAGTTCTCGCTTGCAAATTCTAGAAAACCCCAAAACAAAAAGATCTAC

TCCTTACTCAAGTTCCCAGTGAAAACCAAGCAAGATTTCATTGATTCCGCCTTCTTTTTA

TTTAGATTTTCTTAATTCGTTATTCAATTACGACATAAATGAAATGTGAAATTCTTGAGT

AGTCTACTTCCCTTCGAATGATGAATCCCTTAATTCTTAATTTAAGAA-TTAAAGTGAAA

GTAAAGGAGTACCTTGGAATTCATAAGGGATTTACTTGTCTATGTATTGTTCCATTCGAT

CTTTTAGGTCCCGACTTCACCTCGACGGTTATGCTACGATGCCCTTAAAGCCTATACGCG

ATGGATAGACTCCTGTAACCATGACATATTTGCGCTTACTTGAACATAATTTCTTTCCAA

AAGAAAGGTTAATTCCACAAAACAAAAAAGTTTTTTTACGAGGTACAAATATTTGTATTT

ATTTGTTACGAAATCGACCATAGATCAATTCCCCTTTTGATTTGGGAGTATTGACTACAC

CCCTAATTCTGAGCTTCATGTTACTCCTGCCAAGTGACATGTCAGGTCCAGGGCATCCCA

ATTGGATTGACTGGGATGACAGTTTATCATTCCGAATCTGTAAAATCATAATTTGGATCA

AATCACACATCGCAGTATACTAGGCCTTCTAATTCTTTAAGAGGTTTATCTAAAAGATTC

GCAATATAACTAGGAAGACGTTTTAAATACCACACATGGGTTACTGGGCATGCGAGTTTG

ATGTAGCCCATTTGATATCTTCGTATGCGAGAATCGACAAATTCGACTCCGCATTGTTCA

CAAAATTTCGGGTCTTCTTTTTCATCTCCGATTATTCGATAATTTCCACAAGCACAAATT

CCACTTTTTATAGGCCCAAAAATTCTTTCACAAAATAATCCATCTTTTTCAGGTTTATTG

GTTTTGTAATGAAAAGTATAGGGTTTTGTCACCTCTCCAACGATCTCTCCATTAGGCAGG

ATTTTAGTGGCCCAAGCACTTATTTGTTGAGGAGAAACTAATCCAATTCGTAGCTGTTGA

TGTTTATACCGATCGATCATAGAAGAAAAATTCTGATTCATTCCGATTAAGCTTCCTTCC

TATTAATCTGGAAGTTTTTCTCAGATACAAGGAAATGGTTCAGTTCCAGAGCTAAAGATC

GTAATTCTCGAACGAGCAATCGAAAAGATTCTGGAGCATCCTCAGGATTAGGTATTGTTC

CTCCAATGATCGTAGTACCAAGTACTTCTTGGCGCGCTCTAATATGATCAGATTTATAAG

TAAGCATCTCTTGTAAAATATGAGCAACACCAAATCCTTCTAGAGCCCAAACCTCCATTT

CTCCTACTCGTTGCCCCCCTTGTTTTGCCCTTCCTCTAAGGGGTTGTTGTGTAACAAGCG

CATAATGTCCACTGGAACGTCCATGGATTTTATCATCAACTTGATGAATTAATTTCAAGA

TATAAGACTTTCCTATTAGAACGGGTTGTTCAAAAGGATTCCCCGACCTTCCATCAAATA

TTCTGCTTTTTCCTGGATACTCGGGTTCAAATACCCATGGATTCGCTGTTTGCTTACTGG

CTTCATATAATTCAGAAAACACTAGTTTTCTCGAAGCTTCTTGTTCATATCTCTCATCAA

AAGGCGCTATTCGATAATGTCTGTCTAGCAGACCCCCTGCTAACCCGAGCGAACATTCAA

ATATCTGTCCTACATTCATTCGTGAAGGTACTCCTAATGGGTTGAAAACCATATCAACAG

GTCTTCCATCTTGCAGATAAGGCATATCTTGTCTAGGCAAAATTTTGGAAATGATACCTT

TATTTCCGTGTCTTCCAGCTACTTTGTCGCCTACTTTAATTTCACGTTTCTGTGAAATAT

ATATACGAATCGTTTCTGGATTATAACTAGAACCCCCCCTTTTCTGGATCCACCTCACAT

CAATAACCCGGCCCCTACCACCTATAGGTAGTTTTAAACAAGTTTCTTTTGAAGTAGATA

CCTGAATGCCAAGTATGGCTCGTAACAATCTATCTTCGGGGGCATACGACGATTCTTTCA

CCATTTGGGGCGTTAATTTACCTACTAAAATATCACCCGTCTCTACCCAAGATCCCAGCA

TCACAATTCCATTTTTGTCTAAATTGCGGAGTAAATGGGCTTCTAAATGTGGTATTTCGT

TAGTGATCCTTTCAGGGCCTTGGCTTGTCACATGAATCTGAATCTCATATTTCCGTATAT

GAAAAGAAGTATAAATATCTTCATATACCAAACGCTCACTAATGAGTACTGCATCTTCAG

AATTGTAACCTTCCCATGGCATATAAGCTACTAATACGTTTTTCCCCAAAGCAAGTTCGC

CACCAACCGTAGCAGCACCATCTGCTAAAATTTGTCCCTTTTTAATACATTTACCCCGCT

GAACCTGGGGTTTTTGATGCATACAAGTATTTTTGTTGGAACGTTGATACATAACTAATG

GAATGCTTAGAGTATCTCCATTACCTGAGAAAAGGATCTTTTCAGTATCGGTATAAATGA

TCTTTCCCTCACGTTCGGCTATAGCAAGAGCCCCTGAATCTAGAGCTGCTTGTCGTTCCA

ACCCAGTTCCAACAATGCATTTCTCGGGCCGAGAAAGCGGAACTGCTTGACGTTGCATAT

TAGAACTCATTAAAGCCCTATTCGCATCATTATGTTCGATAAAAGGAATGAGGGAAGCTC

CAATAGAAAAATATTGGAAGGGAAAAATACTTCGAAGATGAACCTGTTCCCATGCAATAG

TCAGGAATTCTTGACGGTATCGAGCTGGAACAACCTGTTCTTCCTGAATATCCTGATTCA

ACGCCAAAGAATTTCCTGCCGCTACCATATAGTATTCATCTCTACCTGGTGATAAATAAA

GCATCCGTACCCCTGTTGATCTTTCAGAAATTTCATAAAATGGGCTTTCTAGAGATCCCC

AATGACCAATCCTCGCATGAATTGCTAAGGATCCAATAAGTCCAACATTGATTCCCTCAG

ATGTGTCAATTGGGCAAATACGCCCATAGTGACTAGGATGGATATCTCGTATCCGAAAAC

TAGCAGTTCGCCCTGTTAGTCCTCCAGGGCCTAAATAACTCAATTTTCTCCCATGAACTA

TTTGTGTCAATGGATTAGTTCGATCCAAAACTTGAGATAAGGGGTGTAAACCGAAAAAAG

ATTCATAAGTGGTTGTTAATGGAGTTGAAGTTACTAAATTCTGAGGAGTCGGTATCAATT

TGTGCCGAATTGCTCCACATATAGTTCCTCGAACCACATTTTCTAAACGAACCAGAGCCA

ATCCGAATTGATCTTGTAAAAGATCCGCTACAGAACGAATACGTTTATTTTTCAAATGAT

TCATATCGTCAAGTGTACCCATTCCAAATTTCAGTTCAATCAAATGATCAGCGGCTGCCA

ATATGTCTCGTGGTAACAAAAATGTATTGTTCTGGGGTATATCAAGGTTCAGTCTTCGGT

TCATATTTCGTCGACCAATCCTTCCTAATTCACATCTTTGTTGAAAGAACTTCTTTTGTA

ATTCCTTACATAAGGATTCAGAAAATACCGGATCCCCACCTACACAAGCAAATTGTTGAT

AAAACTCCAAAATGGCATTTTCTTTTGATCCAATTTTTTTTCTCTCCTTATCATTCAGAA

AAGACAAAAAAATTTCAGGATAACAAACATTGTCTAGAATTTCTCTTAGATTCGAACCCA

TAGCTGATGATAGAACTAGAATAGATATTTTTTGTTTCCTACTCACACGAGCCCATATCC

TTGCTTTTCTATCAATCTCTAATTCTGATCTTCCTCCCCAATCTGATATTATGGTGCCGG

TATAGACCGAAATTCCGTTATGGTCCAATTCTGATCGGTAATAAATACCGGGGCTTTGCA

ATATTTGATTGATCACAATTCTATATATTCCATTTACTATAGAAGTTCCCAGGGAATTCA

TTAGAGGAATGTTTCCAATAAAAATGGTTTGTTCTTGCATATCCCTACTAGTTTTCCAAA

TTAATCCCCCGGATACATATAATTCAGAAGAATATGTGAGTGATTCATACACAGCATCTC

TTTCTTTTATCAACGGTTCTACCAATTGATATCTTTCCACAAATAATTGAAATTCTATTT

CTTGATCTGTATCTTCAATTTTTGGAAACTTATAAAGTTCTTCTGTCAAACCCTGATTAA

TGAACCTATAAAATCCTTCAAATTGTATCTGATTAAATCCAGGTATTGTAGACATTCCCT

CATTTCCATCCCCGAGCATTTTGAATTTCCCATTTCTCAAAAAATCCCATT-ATTGGTCC

ATTCTTCATCGAATTAGCGAATTAGATAGATGATCTAGCAATGATGGAATTTCTATTCTG

TTTACTGAATCACATGAAATTTTACCCAACTCCATATTTGTAATGGAATATATGAACTAC

GTATGAACGGAGGAATAAAGAGAATTTTCTACTGGAATTTGCAACAGATCCAAAGGAATT

GATAAAACATTCCTAGAAACAGAATTCTGCTACTTAGACTTATTAAGGTATAGGGTTTTG

TATAGAATAGAAAAACCAAAATGATTGCAATTCTACCATTATTATGATATTACATATTCC

AATTTGAGAAAAATAAAAGGATTCGGCATTTGATCTTTTCACTGAGATAAAGACACAAAA

ATCAGAAACAATATACGATTTTTTTAGCACTTAACCACCTTTATGTTATGGATTTCATTG

TTCAAAAAATGATTCGCAGAGAAAATAGATATTTTGACTTTACTGATTTTTGAGTAGATA

TAGGATTTAACTGTATAAGAAGGGTTGCATTTATTAAACACGTGTGCTGTGCAGATAGCT

ATAATATCCGACTTTTCTTTTTTTGCCGTTCTATTCGGAACAGCCGGGGTCGTGCTCTAT

CAAAACGAAATTTCTATTCAATGCAAAATGGAAGTATGATAATTTTGATAATTCCCTATC

GACAACATATCTAAATAATAGATATCTGTGTAACAATTTCTGTTCTGGGGTTTACATATA

CTCATCTATTTTGTTATAATAGAAATTGAGAAGGATTTTTTGATTGAAAAAATCAATACT

GATTAGTTCTGTCTCAATTTGTATTTTCTAATGTCATTAGGAAAACACAATTTGGAGATT

CAAATCCCAGAATTGTTCATGAATTCGAAGTAAGCAGTCAATAGTTAATGCTTTAAATTT

GTCCTATATTTTGGCTAAAAATCCACATTTTATTTCTCAATAGAAAAATGAGATATTTTT

TAGCATTGTGGATTTTCAGATACTATACAATCAATCGAAGGGATGGATCAAATCCAATCA

AAAAAAGGAGGTTTCTTTTAGGAAAAGGATTAAGAAAAATAGGACTCAAAATGCAAGTAC

AATAAAAATTCCGTAATCCAGGAAAATTATTATATCCATTTTGTATCATTTTGGCGGCAT

GGCCGAGTGGTAAGGCGGAGGACTGCAAATCCTTTTTCCCCAGTTCAAATCCGGGTGTCG

CCTGATCAACAAAAAGCTCTAAATCTTTTCTTCTCTTCTGTTCTGTTGATACTTGTTTGA

TTCTAAGCATCTGGTCTGAGGGTTTTCTAAAAGATTGTGAATCCTTGCATCTAGGATTCA

AGGAAATATTGTAATCTAATGATAGAGGGGCTGTCAAGACTTCACGATTCCCTTCTATTA

CTAAGGTAGTGTCGAATGACTGGATCTTGACTTAGATTGGAGAGCCTGATAGGAAATCTG

ATTCATGTGGAAAGGGGCGGAAGTTGCTTTATTTTATATACGACGGACTCGCGAGAATCT

TGGAGTGCTCAGGTATTCAATCAATATTAGATTAGATGGATAATTGACTTTTATAGAAAA

AGGGGCAAAACCAAATTCTTTCTTTTCGGCAACCCCCCTAGTCAAGCCCCCCTCTTTCAC

AGCGATAATCGGGAAAGGGGATACGGATTAGATCAAACAAAGGGGGAAATAGGGGTCTGT

AATAGATAGTGATTTCTCTTTTTTAGTGATTTCCCCCTTTATGTTTTTACTTACGAAGGT

CAACAAAAAC-AAAAAAAGAATAGGCCTTATTATTCCTACATGTTCCCATTCCCTTGAGA

TGTTACTACGTATTTTGCTTGTGTTTAATCTTTCACGATTCAAAAAAGAATCATTTATTG

GATTGGTTTCTCAATAGTGTTCGGTCAGAATCCCTTTTTGACTCTGCACCATTGATTCCA

CTATTATTAGAAAGGAATAATGGAATAATTCCTTCATATTTATAGAGATAGGGGACATAA

CTCACATGGATATAGTAAGTCTCGCCTGGGCTGCTTTAATGGTAGTCTTTACATTTTCCC

TTTCACTCGTAGTGTGGGGAAGAAGTGGACTTTAGAAGTACTACTAATTGAGTTGAGGAA

TCAAACTGTATCAATTGTTTTATAGATCGTTCTGCAACGCGTTTTGAACTATTTAAAATC

AAAATATCTGAATTTCCAATTCCATTGGAGTCCAATGGAGTAATGTATGATAGGAATCAT

ACTCTTTCAATCAAAGAACTATTTCAATGATTCCCATGTTTGTATTTCGAAAGGAAAGGG

ATCCAGATGATTGGAAATTTTTTCCAATCTAATTCTTCTGAAATTTTCTATTTCAATTAA

GGGGCTCTTACTATCCTTATAGATTAAGATTAGATGGATACTGAGGAAGACCAGACCTTT

TTTTGATCCCTCTTGACTCTTCAAAGAAGAAGTCGTTTTGTTAAGTGTATACGCACTTTC

TATGAGAAATGATATAGACATAGTGGTTGTCTAACGAGATATGCAATAATAAGATCTTGC

CTCAGGCGAGTCACATATTGCGCATTTACCGATGGGTTTCTAATTTTAGAAAGGAGATTT

TATCTTTATCGACTTATTTGATATCATGGTTCGGGCGTTAAAAATCGGTGAGGTTTACTC

TTCCTTTTCGAAATCCGAAGAAGTGCCCGTGGTCCTCCTGTCAATAGTTAAATCAATTAT

TTCTTCGGAATACTAAA-AAAAAGATTACTACGCGATTTTAGTAATCTATATGCCCATAT

CGTTTTTCAATCATTGATTCTTTCCATAAATACCGATATTCAGATTGGAAATCATAAAAA

ATCTAGTAATTCGAATCATAATTCGAATCATAAGATAAGAGTTAAGGCGATTATTTCAGA

TTGATCGGAACAAGTAGATGTAGCAAATAAATAGAATTGGGTGCTATGTCAATTCCATAC

AATATAGGGAATTTATATACACATATATGAAGAGAATATTGTAGATTGATCTATATAGAA

TGAAGCCTCTATCTTTATTCTAGAGTAGAACTTTATAGACTAAGAGATAGATAGTATGGT

AAGAAAGATCTTTCTTACCATACTATCGAATTCATAAAATACTGCCGATTATAGTCCGCT

CATTTCATTTAAGACGCGAAATTGGAATCCTTTTCATTTTACTTCGTCCATTTTTGATAA

GAACTCAGAAGGAAAGTTTCATTCAAATGAA-TTTGAAAATTGAATTGAATTAATCATTT

TGACTGACTGTTTTTACGTAAATGATAAGTAGAAAAGCGGTAGGAACTAGAATGAATAGT

GCAGTCGCAATAAATGCAAGAATATTTACTTCCATAATCTCATCGGTTTTTTTACTTCGC

AATAACTCGGGATTTAATCCCATAGAGATGATAAATCTTTCGCCTGTAAATTCAATGAAT

GAATTACCTCTCGACGATCTTGAATCGGATCAATATCATGAATAACAATATCTGAGCTAT

CAAATCAATTCGTCGTCGAGACTTGAATAGTATAACATAGGAAGTTCTTTTATCCATACC

GAATCCAAACTTGGATTCCTGACCCAATCAATCCAAAATTCCTTTATTTATCATTTGTTT

CCCTTCTTTTTTCTATAACCTACCTTACGTCTTCCTTGTACAATCATCTGATGATCATCA

GATTGCCCTTCCACTTCGATTAGTCACATAGTTACAAACCCAAACAAACAAGAAAAGCGA

AATGG-AAAAAAAAGGGTTAAGTTCTAAACTCCTTGTGATTTTTTGGAAGACGAAGACAA

AGAAGTTTGATAAAGATGAGGCCGGTATAAAAGATCTAATATCACTATTTTAGTGTTTGT

TATTTCTTCGATGGGACCTTAAAAATGGAAAAATAGGAAATAAAAAAAGCCCCTTTGTTT

TGGAAATTCAATTCTGCCCCCTGACATCCTTTCATAGAAAGGGAGAAATTAATTGATGTA

TTTATTGGATCCGTCGGGACTGACGGGGCTCGAACCCGCAGCTTCCGCCTTGACAGGGCG

GTGCTCTGACCAATTGAACTACAATCCCAGGGAAATAAGGGATCTAGCAGAAAATTTTAT

TCTTTTTTTATCTTCGTATTTCGTATGGGGTATTTCGGAAGGACAAGGGGATTATACAAT

CTCATGGTAGATTGGCGAATTATTGGGCCGAGCTGGATTTGAACCAGCGTAGGCATATTG

CCAACGAATTTACAGTCCGTCCCCATTAACCGCTCGGGCATCGACCCAGGAAGAATCCAC

TTTAGGCTTATTGGTAATCCATGATCAACTTCCTTTCGTAGTACCCTACCCCCAGGGGAA

TTCGAATCCCCGCTGCCTCCTTGAAAGAGAGATGTCCTAAACCACTAGACGATGGGGGCC

GACTTGCCCAACCGCCCTCATACTATGATCATAGTATGAACAATTTTTTGAAATTGTCAA

TATAATGGAATGGTATGATTAGACTCGCGGGATCTTTCCGTTTTTCAGAATTGTATAGAA

TTTTTTGATTCGTCATCCATATTCATGAATCGTTCATTAGAATATTAGAATCGCCACACT

CTATATAAATAGAGTATAGAAATCTATATTCTTTAGAA-----TAATTAAAAAAAAAAAA

GAAATACAAACAACTAAAAATAATATGAGGGATAGGATTTGTTCAGGGAATGATTGGTCC

GTCAGAAAACAAAAGGGAGGGTTAATTTCGATTTTTTTGCTTTCATTCATTGTTAAGA--

-------CTTATCTCTATCTCACACTAAGACGGGAAATTAACAACCAATAAATCTAGTAA

GCGGGATCAAGAAGTTATCGAAAATTTTCTCTAAGAATTTAGTTCAGGGGACAAGTAGAA

TCTCTTCATCACATGAAATATCTTTCAATTTATGTAAAATTGGTAAGTGTACACGTATGT

TATGTATCAATCAAGTGAATTTTGTTTTAATGGGGATCAATTCAATAAAATAAATTAGGG

TCGGTCTTGAATTCATTTTATTTTACCCTAGACTTGCTAGGTAAATCCATTTGATTATTC

AAGAATCAGCCACTAGCCACTACGAGTCTACTGCATGTACTTATGTATATAATATATGTA

CATATAGAGATTTTATCTACATAGTGACTCATTCGGGAATTAAATCAAATAAGCCCTTTT

AACTCAGTGGTAGAGTAACGCCATGGTAAGGCGTAAGTCATCGGTTCAAATCCGATAAGG

GGCTTTGGTTTTTTCATAAAAGTGCAGCCGTAGTATTCAGAAAATAGAGATATTTTTTTT

TATTTGGAATAAAAAAGTAACTAACTGGATAATACGTTATCATTATACTGAGTTAGAGTA

TAGTAGTTCTAGTTAGAAAGTTGAACATTTTTTCAGTCAATTCTCATTATTATGAATAAT

GATAAGCCGCCTCTTGAATCACCAAAGATCCCTATTTTACATTATACCAATCAAATCCAT

TGGAAAGATTCGAAATCAACAAAAGAAAAAGTAAGTGGACCTGACCTATTGAATTATGAC

TATATCCGCTATTCTGATATTAAAATTCGATAGAGATGAAATTGGAATTGGAACAGTGGA

CCCCCCTTTTTTTTATTTCATTTCTTTGGACCTCGAAAGAATTTGTCGATATTTCCGATT

AAATCTTCTTGTTCCTAGATTTTCTATGGGAATAAATTGTTATTCCCTTCCTCTACAGAG

AAACCTTTCTTCCAAGTCACAAGATAAGAGCCATTTCCATTATCTTTCTTTGATTACAGA

TCAAGATGAATTTATATCTATCTAAGTATATTTAGATAGATATCTATCAGATCGTGGCTT

CATGTACCAAACATTTCTATATCGCTGCATCCGATATTTTTGTTCCGACAGTGTGATGGA

GAATGGATGCGAGAAAGAGACTTTCA-TTTTTTTTAAGGAAAAAAATAGGAAATTCTCTC

TTTCTAAGAGATTAAACTCAATAGAAAAATATTCGAAGTATCTTTTTTGCTTTGACCCGT

GGGAAGATATACTCTGGAGTTTTAGATTTATCTGAAGGAAAAGGAGATAGAACAAAGAAG

ACACTCAAAGAAAATGAAAAAAAAAAAAATGGAATCAAATAATGTAATTGGAATAGTTTA

GTATACATAGAAATTAGAATAATCTATAAATATCTTTATTTTTCTCAATCTCACGAACAA

GATCTAAGAATAACATTAGTTAATCGAACAAGAGGGGGGTAGATCGGAGGATCAGTTAGT

AGTGAGAGAGGGGATCACTTGTTCCTTGAAAAGTTCTTTCAAAGGATTCATCTATCTGAT

TGATGAATCATAAGAAGACAATTCATGGTTCATATTCTTAGTAAGAAAGAATAATCAAAT

TGAGTTCATGGATTTCCCTAGGTCAGTTTATGGGCCAATCCAATAAAGTATTTTTATCTT

CGAAACCCATTGGAAGGGGCAGTGTAAGAGAAATCATAGAGAAATGATCGAATCTTCGGA

CGCCCCGAAAATACTGTGAGGTGCTCGGAAATGGTCGAAGTAGTTGAATAGGAGGATCAC

TATGACTATAGCCCTTGGTAAATTTACCAAAGACGAAAATGATTTATTTGATATTATGGA

TGACTGGTTACGGAGGGACCGTTTCGTTTTTGTAGGCTGGTCCGGTCTATTACTCTTTCC

TTGTGCCTATTTCGCTCTAGGAGGTTGGTTCACAGGTACAACCTTTGTAACTTCATGGTA

TACCCATGGATTGGCTAGTTCCTATTTGGAAGGCTGTAATTTCTTAACGGCCGCAGTTTC

TACTCCTGCTAATAGTTTAGCGCATTCTTTATTGTTACTGTGGGGTCCTGAAGCACAAGG

AGATTTTACTCGTTGGTGTCAATTAGGCGGTCTGTGGACTTTTGTTGCTCTCCATGGCGC

TTTCGGACTAATAGGTTTCATGTTACGTCAATTCGAGCTTGCTCGATCTGTTCAATTGCG

ACCTTATAATGCAATCGCATTCTCTGGTCCAATTGCTGTTTTTGTTTCTGTATTCCTGAT

TTATCCACTAGGTCAGTCTGGTTGGTTCTTTGCACCTAGTTTTGGTGTAGCAGCTATATT

TCGATTCATCCTCTTTTTTCAAGGATTTCATAATTGGACGCTAAACCCATTTCATATGAT

GGGAGTTGCCGGTGTATTGGGTGCTGCTTTGCTATGCGCTATTCATGGTGCTACCGTGGA

AAATACTTTATTTGAAGATGGTGATGGTGCAAATACATTCCGCGCTTTTAACCCAACTCA

AGCTGAAGAAACTTATTCAATGGTCACCGCTAACCGCTTTTGGTCCCAAATCTTTGGGGT

TGCTTTTTCCAATAAACGTTGGTTACATTTCTTTATGTTATTTGTACCAGTAACCGGTTT

ATGGATGAGTGCTCTTGGAGTAGTCGGTCTGGCCCTGAACCTACGCGCCTATGACTTCGT

TTCTCAGGAAATTCGCGCAGCGGAAGATCCTGAATTTGAGACTTTCTACACCAAAAATAT

TCTATTAAACGAAGGTATTCGTGCTTGGATGGCAGCTCAAGATCAGCCTCATGAAAACCT

TATATTCCCTGAGGAGGTTCTACCACGTGGAAACGCTCTTTAATGGAACTTTAGCTGTAA

CTGGTCGTGACCAAGAAACCACCGGTTTTGCTTGGTGGGCCGGGAATGCTCGACTTATTA

ATTTATCCGGTAAACTACTAGGAGCTCATGTAGCCCATGCCGGATTAATCGTATTCTGGG

CCGGAGCAATGAACCTATTTGAAGTGGCTCATTTCGTACCAGAGAAGCCTATGTATGAAC

AAGGATTAATTTTACTTCCCCATCTAGCTACTCTAGGTTGGGGGGTAGGTCCTGGGGGGG

AAGTTATAGACACCTTTCCCTACTTTGTATCTGGAGTACTTCATTTAATTTCCTCTGCAG

TATTGGGCTTTGGCGGTATTTATCATGCACTTCTGGGACCTGAGACACTTGAGGAATCTT

TTCCATTCTTCGGTTATGTATGGAAAGATAGAAATAAAATGACCACAATTTTAGGTATTC

ACTTAATCTTGTTAGGTCTAGGCGCTTTTCTTCTAGTATTCAAGGCTCTTTATTTTGGGG

GCGTATATGATACTTGGGCTCCGGGAGGGGGAGATGTAAGAAAAATTACCAACTTGACCC

TTAGCCCAAGTATTATATTTGGTTATTTACTAAAATCGCCCTTTGGAGGGGAAGGATGGA

TTGTTAGTGTGGACGATTTAGAAGATATAATCGGAGGACATGTATGGTTAGGTTCCATTT

GTATACTTGGTGGAATCTGGCATATCTTAACCAAACCCTTCGCATGGGCTCGACGCGCTC

TTGTATGGTCTGGGGAGGCTTACTTATCTTATAGTTTAGCGGCTTTATCCATCTTTGGTT

TCACTGCTTGTTGTTTTGTCTGGTTCAATAATACCGCTTATCCTAGCGAGTTTTACGGAC

CTACTGGACCAGAAGCTTCTCAAGCTCAAGCATTTACTTTTCTAGTTAGAGACCAACGTC

TTGGGGCTAATGTAGGATCTGCGCAAGGACCTACAGGTTTAGGTAAATATCTAATGCGTT

CCCCAACCGGAGAAGTCATTTTTGGAGGAGAAACTATGCGTTTTTGGGATCTGCGTGCTC

CTTGGTTAGAACCTCTAAGAGGTCCAAATGGATTGGACTTGAGTAGGTTGAAAAAAGACA

TACAACCTTGGCAAGAACGGCGTTCCGCAGAATATATGACTCATGCTCCTTTAGGTTCTT

TAAATTCTGTGGGTGGCGTAGCTACCGAGATTAATGCAGTTAATTATGTCTCTCCTAGAA

GTTGGTTAGCTACCTCTCATTTTGTTCTAGGATTCTTTTTCTTCGTAGGTCATTTGTGGC

ACGCGGGAAGGGCTCGTGCAGCTGCAGCAGGATTTGAAAAAGGAATTGATCGTGATTTTG

AACCTGTTCTTTCCATGACCCCTCTTAATTGAGATGAGACAGGAGATCCAATGCTTGGAT

TGAAGTAAGAATCACTTTGATTCTATCATACATA-TTGGGATCGGGTTATACTTAAAAAG

TATTCCTTTTTC--TTTTTTTTTTTACTCATTTATATCTAATCTATTTTTTCTGGCTTGG

CTAAGTGGGATAGCCGAGCCATTCCCCTTTCTTTATGATACCAATCCGAGCCAAACCAAT

AGAAACAAATCTATTCAATGAGA-AAAAAAAGGAGAGAGAGGGATTCGAACCCTCGATAG

TTC-TTGTTCAAAACTATACCGGTTTTCAAGACCGGGGCTATCAACCACTCAGCCATCTC

TCCGAAAGACAATTTTTATTTTATTCCTCCGAATAGAACATGGCCATAGGGGTGGATACC

ACTACTATCTGTAGAAAGATCTCAGGTGTG-AATCTACCGATGGATCTATCTATCCGTAT

ATATATAATCCAGCATGTCCATTTGTGAAATATAAAAAACAAAATTCCATTTCCCCTGAC

TCCATGTATGAATAAAGTGGTCAAAGGGGTAGTAATAAGTCATATAGAATCAATTGATTC

ATGGTAAACTCAAATCCCTCGATGATGTATTTTATTACAATTTTTTGGCTGATAGAGGGA

TCAAATGGTATAGTTCATTTGTTGGTATCTTGGAGGATTAAAAGCATGACTCTTGTTTTC

CAATTGGCTGTTTTTGCATTAATTGCTACTTCATCAATCTTATTGATTAGTGTACCCGTT

GTATTTGCTTCTCCTGATGGTTGGTCAAGTAACAAAAATGTTGTATTTTCCGGTACATCA

TTATGGATTGGGTTAGTCTTTCTGGTGGGTATCCTTAATTCTCTCATCTCTTGAACCTAT

TCGTCCCAGATCCAAAACCGAAATGACCCCCCCCAAAAA--AATTTTCTCGGTTATGAGA

CACATTACAATTTAATATAAGTCCCCAAAATAAAATGGGGGGGTCAAACTTCTTGCAAAC

TTCTTGAATTAAAAAAAAATACAATTTCAATTTATTATATTAATAATATAAATCGCCCCG

AAGAGAGTCTCTGGCCCGACACTGCACAAATACGATCCAGG----TATATATATCATATA

TGTGTGGATATATTGTGTATTAAGAACAAAAATTGCGGATATGGTCGAATGGTAAAATTT

CTCTTTGCCAAGGAGAAGATGCGGGTTCGATTCCCGCTATCCGCCGAAGATCAAGATAAA

GTTATTTTTCCTTTAATATGATAAAGGATTGGGTATAGTTGGCCATGATAGTGTCGCGAG

TCTATCCCTTCTTTTTTTCCTTTCCTACCA-CCCCCCTTTTTGGGGGGGTAATTAATTAC

TAGTTAACAGAGCCAAACCCCCTTTTGATAAAAAAAGATATTGCGGAGACAGGATTTGAA

CCCGTGACCTCAAGGTTATGAGCCTTGCGAGCTACCAAACTGCTCTACCCCGCGTTGAAG

AGAAGAAGTGAAAACTAATAGACAAACAAGAATTGAATGTGCCCCTCTACCATATCTGTA

CAAATAGAGTAGCCCATTTATACAGAATGGTAAAGAGGCCCTATATGATCATCGACCATA

GAAATGAAAGGTTAATCCTTACCAACTTGATCTTGTTGCTCCTGGCAACAAACACGCATG

AACCATTTCACGAAGTATGTGTCCGGATAGTCCAAAGTCTCGATAGTTAGCTCTCGGTCT

TCCGGTCGAAAAACAACGTCGATGAAGGCGTGTAGGTGCACTATTCCGTGGTGGGGATTG

TAACTTTCCATAAATTTCCCATTTGTCACTCAACGATGGAACTTTGCTTATTTCTTTTTT

TGAGGATCGACGAATCAAATGATATTTCTGTTCCAATTTTTGCCTCTTCTTCTCCCTCTG

AATCAAACTTTTCTTTGCCATAATGGTTCAGGTCCTATTAGTATCCATGATACAAGTCGA

ATCCTAGATGTAGAAATATAAGAAGGTGGACCCTCTCTCCATCGAAAGAAATGAGATTAT

CGCGGATACAACACATTAAAAAATTAGCCAAATTTCCCCGATGTAGAGGCAATCAAAAAA

GCCGCATAAGTGAATATATAACCTACAGAAAAGTGGGCTAATCCAACCAATCTTGCTTGG

ACAATGGAAAGAGCCACAGGTTTATCTCTCCATCGAATCAAATTGGCCAAAGGTGTGCGT

TCATGAGCCCATGCTAAAGTTTCAATCAATTCCTGCCAATATCCACGCCAGGAAATTAAG

AACATAAATCCAGTAGCCCAAACAAGATGTCCAAATAAGAACATCCATGCCCAGACCGAT

AAACTATTCATACCAAAAGGATTATATCCATTGATAAGTTGTGAAGAGTTTAACCATAAA

TAATCTCTTAGCCAACCCATCAAGTAAGTGGAAGATTCATTAAACTGTGAAACGTTACCC

TGCCATAATGTGATGTGCTTCCAATGCCAATAAAAAGTAACCCATCCAATAGTATTTAAC

ATCCAAAAAACTGCCAAATAAAATGCGTCCCATGCCGAAATATCACAAGTACCCCCTCGT

CCCGGGCCGTCGCACGGAAAACTATAACCGAAATCCTTTTTATCTGGCATTAACTTGGAA

CCGCGTGCATCTAAAGCACCTTTTACTAAGATCAATGTAGTTGTATGTAAACCCAGAGCA

ATAGCATGATGAACCAAAAAGTCTCCAGGACCTATTGTCAAGAATAATGAATTACTATTT

TCATTAATAGCATTTAACCAACCGGGCAACCAGATGCTTCGACCCGCATTGAATGCCGGA

CCGCTCGTTGAAGATAAAAGTACATCGAACCCATATGAAGTTTTCCCATGAGCCGATTGT

ATCCATTGAGCAAATATAGGTTCGATCAAAATTTGCTTTTCTGGAGTACCAAAGGCAAGC

ATGACATCATTATGAACATAAAGTCCCAAGGTATGGAATCCCAGAAAGAGGCTGGCCCAA

CTTAAATGAGATATGATAGCTTCTTTATGGTCTAACATTCTTGCCAATACATTATCTTCA

TTTTGCTCCGGATTGTAATCTCTAATGAAAAATATAGCTCCATGAGCAAAAGCTCCTGTC

ATGATGAATCCTGCGATATATTGGTGATGAGTATATAATGCAGCTTGAGTAGTAAAGTCT

TGTGCTATGAATGCATAAGCAGGTAAAGAGTACATGTGTTGAGCTACTAAGGAAGTAATA

ACCCCTAAAGAAGCTAGAGCAAGGCCTAATTGAAAATGAAGCGAATTATTGATTGTGTCA

TAAAGACCCTTATGCCCTCGTCCCAATCGTCCCCCCGGAGGAATATGTGCATCTAAAAGA

TCTTTGATACTGTGTCCAATCCCGAAATTCGTTCGATACATATGACCAGCAACAAGAAAA

ATAAATGCAATAGCTAGATGATGATGAGCAATATCGGTCAGCCATAAACTTTGTGTTTGT

GGATGGAATCCCCCGAGAAGAGTTAGAATGGCAGTTCCTGCTCCTTGGGAGGTCCCAAAT

AAATGACTACTTGAATCAGGGTTTTGAGCATAAAGATTCCACTGACCTGTAAAAAGTGGG

CCTAACCCTTGGGGATGCGGTAATACCTCTAAGAAATTATTCCATCGAACATACTCCCCT

CTGGATCCAGGAATAGCGACATGTACTAAATGCCCTGTCCAAGCCAAGGAACTTACGCCG

AAGAGTCCTGACAAATGATGATTCAGACGAGATTCGGCATTTTTGAACCACGAAACGCTC

GGTTTCCATTTCGGTTGTAGGTGTAACCAACCTGCTATTAAGGATATGGCAGAAAGAAAT

AATAGAAAAAGAGCTCCAGTATAAAGATCTTCATTAGTGCGTAAACCGATTGTATACCAC

CACTGATAAACACCAGAATAAGCGATATTCACTGGGCCAAGAGCACCCCCTCGAGTAAAA

GCTTCCACAGCCGGTTGACCAAAATGAGGATCCCAAATCGCATGAGCAATAGGTCTTACA

TGTAAAGGGTCCTGTACCCATGATTCAAAATTTCCTTGCCAAGCTACATGAAACAGATTT

CCGGAAGTCCACAGAAAAATTATTGCTAATTGACCGAAGTGAGAAGCAAAAATATTCTGA

TAAAGACGTTCCTCAGTAATATCATCATGACTCTCGAAGTCATGTGCGGTAGCAATACCA

AACCAAATACGACGAGTAGTGGGGTCCTGAGCTAAGCCTTGGCTAAACCTTGGAAATCTT

AATGCCATAATGCCTTTCAAATCCTCCTAGCCATTATCCTACTGCAATAATTCTTGCTAA

GAAGAACGCCCATGTTGTGGCAATTCCACCCAGAAGGTAATGGGTTACTCCTACAGCACG

TCCTTGTACAATGCTCAAGGCTCTCGGCTGAGTAGCAGGAGCAACTTTTAATTTATTATG

AGCCCAAACGATGGATTCAATAAGTTCTTGCCAATAACCACGTCCGCTGAATAGAAACAT

CAAACTAAAAGCCCATACAAAATGAGCACCTAGGAAAAAAAGTCCATATGCAGATAATGA

AGAACCATAAGACTGAATTACCTGGGATGCCTGTGCCCATAAGAAATCCCGGAGCCACCC

ATTAATAGTAATAGAACTCTGGGCGAAGTTTCCTCCCGTGATATGAGTTACTACCCCTTG

ATCACTTATACTGCCCCAAACATCTGACTGCATTTTCCAACTGAAATGGAATATTACTAC

CGAAATGGAATTGTACATCCAGAATAGTCCTAAGAAGACATGATCCCAAGCCGATACTTG

ACATGTACCCCCTCTTCCAGGTCCATCACAAGGAAAACGAAAACCAAGATTTGCTTTATC

CGGTATCAAACGGGAGCTACGAGCAAATAGAACACCTTTCAGGAGTATCAATACCGTGAC

ATGAATCGTAAATGCATGGATGTGATGTACCAAAAAATCCGCGGTTCCTAACGGAATAGG

TAACAAAGCCACTTTGCCACCCACTGCTACTAAATCACCACCCCCCCAAGTTAAACTGGT

GCTTGCTGTTGCACCAGGAGCCGTTGCGCCAGGTGCTAAAGCATGGGTGTTTTGTATCCA

TTGAGCAAAAACGGGTTGTAATTGTATAGCGGTATCTGAAAACATATCTTGAGGACGCCC

TAAAGCGCTCATGGTATCATTATGAATATATAAACCAAAACTGTGAAAGCCTAGAAAGAT

ACATGCCCAGTTGAGATGTGATATGATTGCATCACGATGTCTAAGGACACGATCTAATAG

ATCGTTGTATCGAGTAGTTGGATCATAGTCTCTTACCATAAAAATGGCTGCATGCGCAGC

AGCACCAACTATGAGAAATCCACCAATCCACATGTGATGTGTAAACAATGACAGTTGGGT

CCCATAGTCAGTAGCTAGATATGGATAAGGGGGCATGGAATACATATGGTGAGCTACAAC

AATGGTTAAAGAGCCTAACATAGCTAGGTTAAGAGATAATTGAGCATGCCATGACGTTGT

TAGGATCTCATATAGGCCTTTATGACCCTGACCTGTAAATGGACCTTTATGAGCTTCTAA

AATATCTTTTAGGCCATGGCCAATGCCCCAGTTAGTCCTATACATGTGACCCGCTATCAG

GAACAGAATTGCAATAGCTAAATGATGGTGTGCAATATCGGTCAGCCATAGACCCCCAGT

TACTGGATCTAATCCTCCACGAAAAGTAAGAAATTCCGCATATTTTGACCAATTCAAGGT

GAAAAATGGGGTTGCTCCCTCGGCAAAACTTGGATAAAGTTGAGCCAAAAGTTCTCGATT

CAAGATAAATTCATGAGGAAGCGGTATCTCTTTAGGATCTACTCCAGCGTTTAAAAATTG

GTTAATCGGTAAAGATACATGTACTTGATGCCCCGCCCAAGAGAGAGACCCAAGTCCTAG

TAGCCCCGCTAAATGGTGATTCAACATAGATTCTACATCTTGAAACCAAGCCAATTTTGG

GGCCGCTTTATGATAATGAAACCAACCAGCAAAAAGCATTAACGCTGCAAAGACCAATGC

ACCGATTGCGGTACAATAGAGTTGTAATTCACTAGTTATTCCAGATGCTCGCCAAATCTG

AAAAAACCCAGAGGTTATTTGTATTCCTCGGAAACCCCCGCCCACATCACCATTCAATAT

TTCTTGGCCCACTATTGGCCAAACCACCTGGGCACTAGGCCCAATGTGAGTTGGATCACT

TAGCCACGCTTCATAATTGGAAAAACGAGCACCGTGGAAATACATACCGCTCAGCCAAAG

AAAGATGATGGAGAGTTGACCGAAATGGGCACTAAATACTTTTCGAGAGATCTCCTCCAA

ATCACTGGTATGGCTATCGAAATCGTGAGCATCAGCATGTAGGTTCCAGATCCAAGTGGT

AGTATCAGGTCCTTTAGCTATTGTTCTTGAGAAATGGCCCGGTCTGGCCCATTCCTCGAA

AGAAGTTTTTACGGGATCCCTATCCACCAAAATTTTGACTTCTGGTTCCGGCGAACGAAT

AATCATTGAGTCCTCCTCTTTCCGGACAACACATACAAAGAGACCCGCCAACAGTCAAAT

AATTAGTAAACCTTTGAGAGATATTTCTATAATTAGTTTGTTTCTCTTCTATCTCCCATT

TATCTATTTTCTTTAGTTATTCACTAGAGCAATTAGGATCTGGAAGTCGATCCGGGGCAA

GTGTTCGGATCTATTATGACATAGCCATGAGGCGCTCAACGGACCTTTTTTATCTTATAA

AATCTTATAAAACCTTTTTGTAGCTTTGGATTGATGCAAAAACGACTTTTTTGTACAACC

TAGTGTAGATTCATATCTCAATTAGAAGGTCTTAGATAGAGCTGCTTTATCTTTTCCATA

GATGATATGAATTACTCTATTCCAAATCACGCGAGCAGCCATTACTAAGAGACATCCCGG

TATATA--TTTTATTTAGTTGTTAATTTAGATTTCAATTTAGGTTTTTAAAAAAGAATTC

GTTTTCATATTAATAATAAAATTAAAGTAAAGAAGTATATTTTTGACTCTATCCCTTTTT

TATCCCTACGAAATATCAAATGAAATAGAACGCTTAGAAGGGATATAATGAAATTCTTTG

ATTGGATCTTCCCAAAGCAAAGGAATGATCCA-TTTTTTGATTTGACTGATGGGGCCAAC

AAACAAAAAATTCTAACAAATATATATAAATAATAGAATAGAATTAATAAACTAAATAAA

CGGCGTCTTCTTTTATTCGAAACGCCCCGTGATCTTCAACCAATTATGTGCTTCAATATA

ATTACCAGGAGTAAGCGCTATAGCCTGTTTCCAATACTCAGCTGCTTGATCAAACCAAGC

CTCCGCAATTTCAGAATCTCCCTGGCGAATGGCCTGTTCTCCCCGGTCGGAATAGGTAGG

TTAATTCCTTCCCTTAGAACCGTACTTGAGAGTTTCCTAACTCATACGGCTCAGCAATCA

TTTGGTGTCCCCCTTTAATCTACCATATCTAACGAATGAGATTTCTGCTGGATCTATCCC

ATTTTGGGGGTTAACCAAAGAGGTTCATTACCTGAGTTTTAAACTGAAATTTGGATTCAA

TTTGGATTAATAATCCGTTTTATTTCGTTTTATCTTTTTTCCCACCTTCAGAAGAATAAA

TTCCTCCTATCGTTAGAATTTTCTGAAAGGTAACTATCTCGGTTTCATATATAAATTTCT

ATAGAATCTTTGAAAAAGACTTTCCTTCTTAAGAAAGAAAAGACTTACTATCTTTGGGAT

CTGATCCTACACCGCTGCTCAAGACTTTAGTGGATCGACTCTATTACATAAGTTGATTCC

TAATTTTTATCTCACATCATGAGTAGGTATATCTACCATCATGACATAAGTACGCAGTTA

TTATCGTATCGGCCCAAAACCTCGCTAATTGATCTTTACGGTGCTTCCTCTATCAATTAG

ATCCTTTTTTTATCCATAGAAAAAAGTAGCTAGGCATATCTATTTCTTCATATTTTGACT

TCTATGAAGTTTCTTTCTTTGCTACAGCTGATAAAAATCGTTGTTTTAGACGATGCATAT

GTAGAAAGCCTATTTTTTTCGTATTTACTAGCAGA-TTTTTTTTTCTTTCTATAGTGGAG

ATAGTCGCACGTAATGACAGATCACGGCCATATTATTAAAAGCTTGGGGTAAGAATGGGT

TTCGTTCTAGTGCTCGAAAATAATATTCCAAAGCTTTCGTATGTTCTCCATTACTTGTAT

GGATAAGACCTATATTATAGAGTATATAACTTCGATCATAGGGATCAATTTCTAGTCGCA

TAGCTTCATAATAATTCTGTAAAGCTTCCGCATAATTTCCTTCGGATTGAGCTGACATCC

GTTACGGTCGTAATTCAAGTAAAAGAATCTCCGTTCCAGAACCGTACGTGAGATTTTCAT

CTCATACGGCTCCTCCCTTATGTGCATAAGGAGAATAATACATGAAATCAAAAAAGACGA

AAATATTCTCATTATGAACTGAGCAGGGCTAGTGTTTTTACAAGAAATCTCTAGCCAACC

TTCCTGCAAGAGATCTTTTCTTAACATCAAGCGTGTTGGGACTAGATAGAAATGAGAACT

CCAACAATTTCTTTGTTTTCAACGCCTCCTAATTTCCAGGAATTAGTCACTTCAACAGCC

TTCGATGGTTATACGGGTATCCAAAGTACGAACGAGATGGATGTTTGTTGTCCCAACCAT

TCTTTTAGTCCCGAGCCCGATAAGGAAAGGGATAATTTCTAACAAAGTTTTCGTGTTGTT

GATTCCTAGGTGTAGTGCTTCTTCCCCTATGCTGCCTATTGGTACTAGTGGAGTAGGATT

GACCCATAATAAAGAACCCCTAGGTGTAACCTTTCGCTCAATACTAGAATCAAAATTGAA

ACATAGCATCTGAGGTTGCATTAATCGAGGATACACGACAGAAGGAATTGTTCTATTTCC

AAACTTCACCTTCAACAAGCGTAGATTTATTTCCAAAATTTTCCTGAATCACGTGTCTTT

CTCGTAAGACTGAGAGAAATAAAAAAAAAAA-AGAATCAAATCACACCATCTCTGTAATA

GGTAAATGCCTCTTTTTCTCCTGAAGTTGTCGGAATTATTCGTAATAAGATATTGGCTAC

AATTGAAAAGGTCTTATCAATAAAATTTCCATTTATCCGCGATCTAGGCATAGGTAGCAA

TCCATTCTATAATTCTTCTCATTACCTCTCGTGGGAAAATGATCCCACAAAGAAAAGAAT

TGTACAGTACGAAATAACATAAAAATAGATTGATTAAAAAAAAGATTATGGGCCTTCTGT

TCCTGAATCAATACAAAATAGTTGAACCCGATTCGATTTCATACTAATGTAGTAGTATAC

GGAACTATTCCGATTTCATTGAAGTTACAGATTAGAGTAACTCGATAAATTTGGATTGAA

TTATGATACAAAAACGAAAAAAGATCGAATAATCATTCTATGATGAAAATAGATAGAATA

ACCGCCTCTTTTGTCTGTTGTGTACATAGCAGGTATACAATCAACTATACAAAATGTTTT

AGAAATTTCTAATAGAGGGGGTTTTGTTGTTGAGAACTCTAAAACTGGAAAGGAATTTAA

TAATTTGTAGGGTATGGAATCATAGTCTCTATAGAATTATGATAGAAAGGTATCCATTAA

CCCTAGTCTAAAAAATCTAGACCCATCAATCAGTTGATTCATTCTAATTCATTGATTTAA

TCCATTCTAAATATTAGAAATAGAAGGGATTAATTTTTGCAAACATGAATCCCATTTTTC

ACAATTTTTCGTAAGAAAAGAATTTGATCTTTATCTCGGAGCCTCGAAGGAAATAGCTTT

CTTTACTTTGATGAAAAATTTTCGATTTTTATTTG-TATAGTTAAAATGGATGTACCTAT

CCCAATAATCTATAATGGAATATGAATAACTCCCT-ATTCACTCGGTTTTTGGTTCATAA

TCATTATGTAGGAGAGGTGGCCGAGTGGTTCAAGGCGTAGCATTGGAACTGCTATGTAGG

CTTTTGTTTACCGAGGGTTCGAATCCCTCTCTTTCCGTACCTTCACTTAATAGACCGATT

TGACTAACAACACTGGATCAAATAGCAATGGAGACCTTTATTCCACTAGTTAGACCTTTC

ATTGATATAGATTCTCTATTCCTAATTGCCGTGACACGTAAAATACTATAAAATAGGAAA

AATTCTGGAAAGAAAATGAAAATATCCCTTCGGTCTATGATACATATACATAAATGGGAG

AAAATCCGGATCAAACCCGTATTTTTCTTACTTAATCTTAGGTTCATTTACTTCGATGAA

AGGGAAGAAAATTGCCCGAACCCTTGTTATTTTATTTGAGTTTAGGTTTAAGTCTGACGA

GAATAATATTCTACGACTAGCAATTCATTTATTTTCAAACCGACCCATTTACTATCTATT

ATTTGATTGACTAATCCTTTATATTGGAATGGGTGAAGGGTCAAATGGTTTGGCAATTCC

TCATGAGGGGATGAGTCGAGAGAATTTTGAATCAGAGTTCTGGATTTTTGTTCATCCTTC

GTCATAATAATATCTCGGGGTTTGCAGCGATAACTTGGTATATCTACTATACGCCCATTA

ACTAAAATATGTCTATGGTTAACTAATTGGCGGGCTGCGGGAATAGTCGAAGCCATACCC

AATCGAAAAAGGATGTTATCCAAACGCATTTCAAGTAATTGTAGTAAAACTTGACCTGTT

GACCCCTTGGCTTTTCCGGCGATACGAACGTATTTAAGTAATTGTCGTTCTGTAAGACCA

TAGTGAAAACGCAATTTTTGTTTTTCTTCTAGACGAATACGATATTGAGATTTTTTCCCG

GAACGTGATTGGTTTCTAAGATCACTTCCGGCTTTAGGCCTTTTATTAGTTAGTCCTGGT

AAAGCCCCCAGGCGGCGTATTTTTTTGAAACGAGGTCCTCGGTAACGCGACATAAAGACT

CCTTATTCCTAGTTAGAATTAAT-TTTTTTTTATTTTACAGAATAAACCTAAACTAAAAC

TGAACTAAATGAAGCGAAATCTACTGAAGTAGTGTACTTGTACTATAAAGAAGAATGAGG

TAAATTGGATAAATATCCAGACTTTCTATTATTATATATATATAGAAAGGATCCTTTTCC

TGACATAGTTGGAAGTTCCTATAACTTCCAAAATTCATGGATTTTGGAAAAGGGGGAAGG

CACTTTTTCAATATTCTTTGATTTCAAAGGGACATTATCAATCATTAAAAAATGGAATAA

AAAATGAAGGAAAAGCCGGCTATCGGAATCGAACCGATGACCATCGCATTACAAATGCGA

TGCTCTAACCTCTGAGCTAAGCAGGCCCACATAATAGAAACTTTCTATCCATAGGAATTC

AATACACTACACTATAGTGTCTATAGAATATAGAAAAGGATAGAATATAGAATTTCAAAT

AAATATTAAATATTCTAGAACATAACCATTAATATAGCGATATAAAATTTCGATTTCTTT

ATCACAATTCTAATATTATTAGTATAGTAAATCTTAAATATTTTTAGATAGTCAAATTTT

CTTTTTCATTTTTGTTTGAATTCACATGACATTTGAAATTCTTTTTGTTACACTTCTCTA

TATTTATATTTTGAATTCTATTAATTCTTTCGAATTCGAATTATTTAATTGATTAGAACT

AATCAAACATTCCTCTGCTTTCATTCGTAAAGCGGAAAGAAAAGAAAAAAGAATCGACCG

TTCAAGTATTGCAAATTGCATGGGAAAAGTGGCAGGAAGAGAGACATATATATGGGGTAT

ATATCCATCTATATTGAATTGCCGATACAGAAATGATAAAATCCAATTTGATTGAATCAA

ATACGGGTTTCCGATAAGGAAGAAATCAAAGAGGGGAAAATACTTTACAATGAAATCCTA

ATCTCGGGAAGGGGGATATGGCGAAATTGGTAGACGCTACGGACTTAATTGGATTGAGCC

TTGGTATGGAAACCTACTAAGTGGTAACTTTCAAATTCAGAGAAACCCCGGAATTAATAA

AAATGGGCAATCCTGAGCCAAATCCTGTTTTCCCAAAACAAAGGTTCAGAAAGAAAAAAG

GATAGGTGCAGAGACTCAATGGAAGCTGTTCTAACAAATGGAGTGGACTGCGTTGGTAGA

GGAATCTTTCCATCGAAACTTCAGAAAGGATGAAGGATAAACGTATATATTGAATACTAT

ATTAAATGATTAATGACGACTCGAATCTCTATCTGTATTTTTTTATATGAAAAATGGAAG

AATTGATTCCACATTGAAGAAAGAATCGAATATTCATTGATCAAATCATTCACTCCATAG

TCTGATAGATCTTTTAAAGAACTGATTAATCGGACGAGAATAAAGATAGAGTCCCATTCT

ACATGTCAATACCGGCAACAATGAAATTTATAGTAAAAGGAAAATCCGTCGACTTTAAAA

ATCGTGAGGGTTCAAGTCCCTCTATCCCCAAAAAGCCTATTTGACTCCCCAAATATTTAT

CCTATCCCTTTTTCATTAGCGGTTCCAAATTCCTTATCTTTCTCATTCACTCTATAAACG

TATTTGAGCGTAAATGATTTTCTCTTCTCACATGTGATATATAATACACATCCAAATTAA

GCAAGGAATCCCCATTTGAATGATTCACAGTCAATATCATTACTCATACTGAAATTGACA

AAGTCGTCTTTTTGAAGATCCAATAAATTCCAGGACTTGGAGAAAACTTTGTAATCCCCC

CTTGTCCTTTTAATTGACATAGACCCCAGTCATCTAATAAAATGAGGATGGGATGTTACA

TTGGGAATGGTCGGGATAGCTCAGCTGGTAGAGCAGAGGACTGAAAATCCTCGTGTCACC

AGTTCAAATCTGGTTCCTGGCACATGATTCATTTGTATGAGTCTCTCAATTAGAAATTAA

TTGATATGAATCGAGATACATATTCATTTCTAATCTAGATCATAATACATACTTTTATCT

ATCTTAGCGAGATATACCCCATCTATATAGATGGGTAGAGTTTTTTAAATAAAGTATCTA

AAAGAATTCAATTCTATTTTCTC-TTTTTTTTCTTTCGTTCAAAAAATCTGTTAATACTT

CATACATATTTGAAAAGTTCAATTAGTTGGTTGAAAGACTAAAAAGTCGAAGTTGAAAGA

GACAAGATTCGGTTCAGATACCAT--AAAAATAAAGAAAATCTGATACCCTTTCATTTCT

TTGTATTTTCTCTTTCATATTCGAT-----------------------------------

------------------------------------------------------------

--------------------------------------------------------ACGA

A-CCCCAATTCTGTCTTTTTTTGTTAGCCTATCCATAATTCCTAATATAAATATATAAAC

CAAACTTCAATTAGAATTATTCTGGTTAATCTAGAACCGAAAGTACAATCCTTGAATCTC

TGAAATTGTATAAGTGGAAATTAGTTTCTTATCATTCAATGAGCATCTTGTATTTCGTAA

AAATTGGGGGCAATATAATCCTTACGTAAGGGCCATCCTATCCAACTTTCAGGCATTAAG

ATACGTTTCAAGCGTGGATGATTATCATAAGAGATTCCCAACATATCATAAGATTCTCGT

TCTTGAAAATCCACACTTTTCCAAACCCAGAAAACGGACGGAATTCTAGGATTCCTCCTT

GAGGCAAATACTTTTATGCATACCTCTTCTGGTTGATCCACACCATACTCTATTCTCGTA

AGATGATACACACTAGCTAACAGTCCGCCAGGTGCTACATCATAGGCACATTGGGAGCGT

AGATAATTGTAACCATATACATATAAAATGACGGCAATGGAATGCCAATCCTTGGGCTTT

ATTTGTAAAGTCTCTATTCCTTGATAATCAAAGCCCAAAGATCTATGAATTAGCCCATGC

TTGACTAGCCAAGCAGACAAACGACCCTGCATCTTTTTTATCTCCCGCATTTTTAGCTAG

AATATTTCACATTCTCGATCAAATTTATGAAGATTCACCCACTACTTGTTATTCTGTACA

ATACAAAGGAATCCTGTCTAATTCACTAATTCGTGAGAAGATACTGAATTTTTATATTTG

AAAAAGTTTTCAGTAGGGATCTCTGAAGTAGATGGCGGTTGATAAAGGAATCCTTGATCA

TAATTTCCAGTATTCATACTGCGTCCAATATGAAACTTGTGATTGGTAGTAAAACACCGA

TTTGCTTGTTGAGACCTAATTCTATCTTCATAGATTTCTCGAGATATTTTCTTACGAAGT

TTTGTTATAGCATCTATAACTGCTTCCGGTTTAGGTGGACAACCTGGCAAATAGACATCC

ACGGGAATTAGCTTATCGACTCCCCGAACAGTACTATAAGAATCGGTACTGAACATCCCT

CCTGTAATTGTACAGGCTCCCATAGCAATAACATATTTTGGTTCGGGCATTTGCTCATAT

AATCTCACTAAAGAGGGTGCCATTTTCATTGTTACTGTTCCGGCTGTTAAAATTAGATCC

GCTTGTCTAGGACTCGATCTTGGTACTAATCCATAACGATCAAAGTCGAATCGTGATCCT

ATTAGTGAAGCAAATTCAATAAAACAACAACTGGTACCATAGAGAAGCGGCCATAAACTA

GAGAGCCTTGACCAATTTGAAAGATCATTTAATGTAGTTGAAATAACTGAATTTTGGACT

GTTTGATCAAGTAAAGGAAACTCAATGGAATTCATAACTTTTTCAATC-TTTTTTTTTCC

CTTTTCTTTTTATTGTCTGAATATTCAGGAGCTAAGACCATTCCAATGCCCCCTTTCGCC

ATGCATAAACTAAACCAACAATTAAGATAAGCACGAAAATTAAAGCTTCTATAAATACAG

ATACACCCAATACATCGAAACTCATTGCCCATGGATAAAGAAAAACCGTTTCAACATCAA

AAACAACAAAAACGAGAGCAAACATATAATAACGGATTCGAAATTGTAACCAAGCGTCGC

CCATTGGTTCTATACCCGATTCATAACTAGAAAGTTTCTCCGGCCCTTTCCTAATCGGGG

CTAAAATCCCGGAAATGAAAAATGCCAAAATAGGAATAAGACTTGATATTATTAGAAATG

CCCAAAAAATATCATATTCGTAAAGCAAAAACATAGACGCACTCCTATGAACGTGGAAAA

TATACCGGATGGGTCGATTCGAATTGAAATTGTCAAGTCATCCATAACTGTTTAGTCAAA

ACAAGAATTCATTTTGACCAAACCATCTAGTTTCCTTTGTTTATTGTGGGGCATATCTCA

TTTCAAGATTCATCGACTGACTTGACTGGAATCCTATTTCCAGTCTACTTATTTTTATTT

CTTTTTATTTCTATTTTATTTAGTTAGTATAACTCTAACTATTACTTTTATACAAATTCT

CTTGTTTTCACCTAGGATTCTTGCTAAAGAAACCTAGTTCCAAATAAAAAGAAAATAGAA

TTCTTATTTTGTGTTTAGAATTTCTAACTTATTATTTTTAAAAT--TATATATTATTCTA

AATTCTTTAGAGATTTCTA-TTTTTTTTTTAGGAATAGAATCTGGAGTTTGTCGTTTTTT

TCTTAGTGATTTCGAATGGAACAAGTATTCAAATGTAAGAGAATGGATAGGTATTTTGAT

TTCTAATATCTTAGTGTTGGTATATTCCTTTTATTTTATTAGAGGGGTTTCTCTTGATTG

AATACAGAAAAAGAAGACCATCCCCTTTGTGCTTCGATAGGTCTAGGTAAGGTATACGAA

GAAAAAGCCTATTTGACATTGTTGACAATGAAACTTACCAAAGAGATTCGTTTCTCAACA

AACTTTGGCTTGTTGATTGTGGAC-AGGTAAATTCATATGGAATTTACCTCCGAAGATTA

ATGACGAAAGGTTGGTTTGTTTATCCACGATTGGATAAATATCGATTCGATCCCTTTTTC

TTTCAGTTTTATGTTCTGCTTTAAACGATTCCCGTGAGTGAGTTTATAGGAATAATTTGG

ATTTCGATGAACCAACCCAACCGGTCAGTTACAAGCAACAAACAATAATGAAGAAATGAA

AATTCGAATATTTTCTATTTTGAATTTTCATTTTATAGGGCTCTAGGGCTATACGGACTC

GAACCGTAGACCTTCTCGGTAAAACAGATCAAACTTATTATTATCAAAATGATCTGAACT

GTTTCAAAGACCCAACATGCATTTTTTTTGCCTTGGGCTCTTTCATTAACTGATAGAAAT

ATCAGCCAGTCTGCCATATTTTTCAAAATAAGATTAAGGAGATGGCTCCATGTGCTCTGA

TTCATTATTTGGGATTCTGATCCAGGAGCACTACCAAAGTGTTTCAAAGGTGGGGTTATC

TTGACGTAGGTCTGCCTCTGGCCTAGATCAACCTAAGTTAAATGAAGTCTCTATCGTTCG

GCTTAAAAAATAAAATATGAAACTTCATACACCTTAAAGTTCATAGGACGAAAAGAGATT

TTTTGAGGACCTTATACTCATTATGCCTAGCATTGAATGGACTGGTATTGACCTTATCAA

TATCTCAAATCAATGTATGGGGTCTGTTTGGTACCTAAATGGGCACCAAAATCGGACCGA

ACCATTTGTCAGGCTACTGTTCCCTCACAGTTATGGAGTAAGACATCGATTTCTCAATAA

GATGCATTTTTTTGATTGTATGATGGACCCCCCTGAAAAACATTGGCGCGCGTGTAAACG

AGGTGCTCTACCAACTGAGCTATAGCCCTTAGTGCTTGTGATACATATTTTATCATGGAG

ATAATTTCTTGTCAAGATGAATATTCTATGATCCAACATCCTCCATTTTTGAGTGGTATT

GCTTGTATTAGTATTGCTCATAAGTAATATGATATTTATAATCCATCGATGTCATGGGTT

CCATTTGGTTATCTTTGGGATGATAAATGACCTACTTAACTCAGTGGTTAGAGTATTGCT

TTCATACGGCGGGAGTCATTGGTTCAAATCCAATAGTAGGTAGAACTTATTAGATACCGG

AGTCAATGGTACCTAATAAGTTTTTCGACCCACCCTCTTTTATTTTTATTGATTTTGTAT

CTTTTTTATTTATTTTATTTTCGTTGTAGCAAAATAGGATTCTGCTTGATTGGATTCACT

CGACAGAATCCTATCAAAATAGGCTTAGAAACAGAACTTCTTTTGATTATTCGAACGCGC

CAACTAGTTAGGAAATCACATTGACAGCCTCTACTCTTGTCCTAGCTCGTCGGAGAGCTA

GATTCGCCTCAATTATTTGTCTCTTTCCTTCAGCTTTTCTCAAATTAGCTTCGGCTATTT

CAAGAGTTTGCTGAGCTTCTTGTGGATCAATGTCACTACCCTTTTCCGCATCATTTACTA

AAATAATGATCTCATTATTTCCTATTCTAGCAAAACCACCCATCAGAGCCATCGTTAACC

ATTGGTCCTTAAAGCGTATTCTCAAAATCCCTATATCTACAGCTGTAGCAATAGGGGCAT

GATTTGGTAATACGCCAATTTGACCACTATTAGTATATAAAATGATTTCTTTCACTTCTG

AATCCCAAACAATTCGATTAGGGGTCAGTACACAAAGATTTAAAGTCATTTCTTCAAATT

GCTCTCCATTTCTAAGTTCATAGCCTTCGCGGTAGCTTCATCGATATTACCTACCAAATA

AAAGGCCTGTTCAGGAAGACCATCTAATTCTCCGGAAAGGATCAATTGAAACCCTCTAAT

GGTTTCTGCTAGACCAACATATTTCCCTGGAGAACCGGTAAATACTTCTGCTACAAAAAA

AGGTTGTGATAAGAAACGCTCAATTTTTCGTGCTCTTGCTACGGTTAAACGATCCTCTTC

GGATAATTCGTCCAACCCAAGGATAGCTATAATGTCCTGAAGCTCTTTGTAACGTTGTAA

AGTTTGCTTAACTCTTTGCGCAGTTTCGTAATGTTCCTCACCAACGATCCGAGGTTGAAG

CATGGTTGACGTTGAATCTAAAGGATCTACTGCTGGATAGATCCCCTTGGCAGCTAATCC

TCTTGATAGTACGGTAGTAGCATCTAAATGTGCAAATGTCGTAGCAGGAGCAGGGTCGGT

CAAATCGTCTGCAGGTACATAAACTGCTTGAATAGAAGTTATGGACCCTTCTTTGGTAGA

AGTAATTCTTTCTTGTAAAGTACCCATTTCGGTACTCAGGGTAGGTTGATAACCGACAGC

GGAAGGCATTCTACCCAATAAGGCCGATACTTCGGATCCTGCTTGGACGAAACGGAAGAT

ATTGTCGATAAATAGAAGTACGTCTTGTTCATTAACATCTCGGAAATATTCCGCCATAGT

TAGGGCAGTCAAACCAACTCTCATACGAGCTCCAGGCGGTTCATTCATCTGGCCGTAAAC

TAGAGCCACTTTTGATTCTGCAATATTTTCTTCATTAATCACTCCAGATTCTTTCATTTC

CATGTAAAGATCATTTCCTTCACGAGTACGTTCACCCACTCCGCCAAATACGGATACGCC

CCCATGGGCTTTGGCAATATTGTTAATCAATTCCATAATGAGTACCGTTTTACCAACTCC

AGCTCCTCCAAATAGTCCGATTTTTCCCCCACGGCGATAAGGGGCTAAAAGATCTACTAC

TTTAATTCCTGTTTCAAAAATAGATAATTTTGTATCTAACTGTATAAAGGCAGGCGCAGA

TCTATGAATAGGAGATGTTGTACGAATATCTACAGGACCTAAATTATCAACAGGCTCTCC

AAGCACGTTGAAAATTCGTCCCAGAGTCGCTCCACCGACCGGAACACTTAGAGGAGCTCC

TGTGTCAATCACTTCCATTCCTCTCATCAGACCATCTGTAGCACTCATAGCTACAGCTCT

AACTCGATTATTTCCTAATAATTGCTGTACCTCACAAGTCACATTAATTGCTTGACTAAC

AGTATCTCGACCTTTAACTACCAGAGCGTTATAAATATTAGGCATCTTGCCCGGCGGAAA

GGCTACATCTAGTACCGGACCGATGATTTGGACGATACGGCCCAGGTTTTGTTTTTCAAG

CGTGGAAACCCCAGAACCAGAAGTAGTAGGATTAATTCTCATAATAATTAATAATAAACA

AAATATGTCGAAATTTTTTTTTGCGAAAATTATCGAATTCAAAATAAAGGTCCGATAGCA

CGGAGATCAGTTAATTCAATAAGAAATGTCAATAAGAAATGGGAGTTAGCACTCGATTTC

GTTGGTACCATTCAATCGAATCCAATTCAATTGTTTACTTATTCATCCACTTGCAATTTT

CAAAATCTTGAAAATTGCAAGTGGATGAATAAGAATCTTGAGAAAGTTTTTCATTTGTCT

ATAATGATAGACAATCCCATCTATATTATAGAAAATATTCTATCGAATTCGAACCTGAAC

TCTATTTACATTAGGATTCATTATTTCTATATCATCGGTGCTTCGTATTTTTTATTTCAG

CATATCGATTTACGCTTACGCCTAGCCTATATTTTTTCTTTTCGTTTTTTTATACCCTTT

CATAGACGAATTCCGCATATTTTCACATCTAGGATTTACATATACAACATATATCACTGT

CAAGAGGGAATTTCTTATTAGTTAGGTTAGGTATTTCGATTCCAAAAAAGGT-AAAAAAA

AATTGGGTTGCGCTATATATATGAAAGAGTATACAATAATGATGTATTTGGTAAATCAAA

TACCATGGTCTAATAATCAAACATTCTGATTAGTTGATAATATTAGTATTAGTTGGGAAG

TTTGTGAAAGATTCCTGTGAAAAGTTTCATTAACGCCTAATTCGTGTCGAGTAGACCTTG

TTGTTGTCAGAATTCTTAATTCATGAGTTGTAGGGAGGGATTTATGTCACCACAAACAGA

GACTAAAGCAAGTGTTGGATTCAAAGCGGGTGTTAAAGAGTACAAATTGACTTATTATAC

TCCTGAATACGAAACCAAAGATACTGATATCTTGGCAGCATTCCGAGTAACTCCTCAACC

TGGAGTTCCGCCTGAAGAAGCAGGGGCTGCGGTAGCTGCCGAATCTTCTACTGGCACATG

GACAACTGTGTGGACCGATGGACTTACCAGCCTTGATCGTTACAAAGGGCGATGCTACCA

TATTGAGCCCGTTCCTGGAGAAGCAGATCAATATATCTGTTATGTAGCTTACCCTTTAGA

CCTTTTTGAAGAAGGTTCTGTTACTAACATGTTTACTTCCATTGTAGGTAATGTATTTGG

GTTCAAAGCCCTGCGTGCTCTACGTCTGGAAGATCTGCGAATCCCTCCTGCTTATGTTAA

AACTTTCCAAGGCCCGCCTCATGGGATCCAAGTTGAGAGAGATAAATTGAACAAGTATGG

TCGTCCCCTGTTGGGATGTACTATTAAACCAAAATTGGGGTTATCTGCTAAAAACTATGG

TAGAGCAGTTTATGAATGTCTTCGCGGTGGACTTGATTTTACCAAAGATGATGAGAACGT

GAACTCCCAGCCATTTATGCGTTGGAGAGATCGTTTCTTATTTTGTGTCGAAGCAATTTA

TAAATCACAGGCTGAAACAGGTGAAATCAAAGGGCATTACTTGAATGCTACTGCGGGTAC

ATGCGAAGAAATGATGAAAAGAGCTGTATTTGCTAGAGAATTGGGAGCTCCTATCGTAAT

GCATGACTACTTAACAGGAGGATTCACTGCAAATACTAGCTTGGCTCATTATTGCCGAGA

TAATGGACTACTTCTTCACATTCACCGTGCAATGCATGCAGTTATTGATAGACAGAAGAA

TCATGGTATACACTTCCGTGTACTAGCTAAAGGGTTACGTATGTCTGGTGGAGATCATAT

TCACTCTGGTACCGTAGTAGGTAAACTTGAAGGGGAAAGAGACATCACTTTGGGCTTTGT

TGATTTACTGCGTGATGATTTTATTGAAAAAGATCGAAGTCGCGGTATTTATTTCACTCA

AGATTGGGTCTCTCTACCAGGTGTTCTGCCCGTGGCTTCAGGGGGTATTCACGTTTGGCA

TATGCCTGCTCTGACCGAGATCTTTGGGGATGATTCCGTACTACAGTTCGGTGGAGGAAC

TATAGGGCACCCTTGGGGTAATGCGCCAGGTGCCGTAGCTAACCGAGTAGCTCTAGAAGC

ATGTGTAAAAGCTCGTAATGAAGGACGTGATCTTGCTTCTGAGGGTAATGTAATTATCCG

TGAGGCTAGCAAATGGAGTCCTGAACTAGCTGCTGCTTGTGAGGTATGGAAAGAGATCAA

ATTTGAGTTTGCCGCAATGGATACTCTATAAGTAAGATAACAAGTAATTACCCTCCGTTC

TCTTAATTGAAAACTCGGCCCAATCTTTTACTAAAAGGATTGAGCCGAATACAAAGATTC

TATTGCATGTATTTTGGCTAAATCTATACTTCTCCAGATATACAAGATTTGAAATACAAA

ATCTAAGACTAAATCCAAATCGAAGACTCAAATGTTTCTATTGTTGTTTTGGATCCACAA

TTAATCCTATGGATCTTTAGGATTGGTATATTTTTTCTATATCCTGTAGTTTCCCTGAAT

CGAGCGAAGTATCACAAATCTTTCTACCCATCCTGTATATTGTCCTTTTCGTTCCATGTT

GGAATAGAACCTTAATTTCTTACTTGTTATTAGTTAGTTATTAGACGAGATTTTACGAA-

AAAAAATTCTTTCTAGGAGAGAACAAATA-T--TTTTTTTTTGATGCGAAGACATAGGAG

AAACTACTCTTTTTCATTATATTTAGAATGAAAAGGGATTCCATCATATTCATATATAGT

GAAGTCTTACCCCGGATTCCCACAAAAGAAAATTCTTTTTCACAGTTAATAATGATAGTG

AGTGGATTCCTATGTTTATTCTGATAGGAAATGAAAATATTCAAATAAAGAATTTTGGAT

CGAATGACTATTCATCTATTGTATTTTTATGCAAACAAATAGGGGGGAAGAAAACTCTAT

GGAAAGGTGGTGGTTTAATTCGATGGTGTTTAAGAAGGAGTTAGAACGCAGGTATGGAAT

AAATAAATCAATGGACAATCTTGGTCCTATTGAAAATACTAGTCAAAGTGAAGATACGAA

TAGAAAAGCTAAAAACATTCATAGTTGGAGGGGTCGTGACAATTCTAGTTACAGTAATGT

TGATCATTTATTCGGCATCAAAGACATTCGGAATTTCATCTCTGATGATACTTTTTTAGT

TAGAGATAGTAATGGAGACAGTTATTCTATCTATTTTGATATTGAAAATCAGATTTTTGA

AATTGACAATGATCATTCTTTTCTGAGTGAACTAGAAAGTTCTTTTTATAGTAATCGGAA

TTCTAGTTATCTGAATAATGGATCTACGAGTGAAGATCCCTACTACAATCGTTCCATGTA

TGATACTCAATATAGTTGGAATAATCACATTAATAGTTGTATTGACAGTTATCTTCAGTC

TCAAATCTATATGGATACGTCCATTGTAAGTGATAGTAGTGACAGTTACATTTCTAGGTG

TATTTTTGGTAAACATACAAATAGTAGTGAAAGCGCGAGGTCCGGTATACGAACCCACAC

GAAGAGTAGTGATTTAACTCTAAGAGAAAGGTCTAATGATCTCGATGTAACTCAAAAATA

CAGGCATTTGTGGGTTCAATGCGAAAATTGTTATGAATTAAATTATAAGAAATTTTTGAA

ATCAAAAACAAATATTTGTGAACAATGTGGATATCATTTGAAAATGAGTAGTTCAGATAG

AATCGAACTTTCGATCGATCCCGGTACTTGGCATCCTATGGATGAAGACATGGTCTCTCT

GGATCCCATTGGATTTCATTCGGAGGAGGAGCCTTATAAAGATCGTATTGATTCTTATCA

AAGAAAGACAGGATTAACTGAGGCTGTTCAAACAGGCATAGGTCAACTAAATGGTATTCC

CGTAGCAATTGGGGTTATGGATTTTCAGTTTATGGGGGGTAGTATGGGATCCGTAGTCGG

GGAGAAAATCACCCGTTTGATTGAGTACGCTACCGATCAATTTCTACCTCTTATTGTAGT

GTGCGCTTCCGGGGGGGCACGCATGCAAGAAGGAAGTTTGAGCTTGATGCAAATGGCTAA

AATATCGTCTGCTTTATATGATTATCAATCAAATAAAAAGTTATTTTATGTATCAATCCT

TACATCTCCTACTACTGGTGGGGTAACAGCTAGTTTTGGTATGTTGGGAGATATCATTAT

TGCTGAACCCAATTCCTATATTGCATTTGCGGGTAAAAGAGTAATTGAACAAACATTGAA

TAAAACAGTACCTGAAGGTTCACAAGCGGCTGAATATTTATTCCAGAAGGGCTTATTTGA

CCTAATTGTACCACGTAATCCTTTACAAAGCGTTCTGAGTGAGTTATTTAAGCTCCACGC

TTTCTTTCCTTTGAATTAAATTCAATCAAGTAGAGCACACAAAATTCAATTAGTTTATTT

GTAGCAAACAAGTAGTTAGTTTATAAGAATCAAAGTAAATAAGAATGGAGTTTTCTTTGA

TGACCTAAGATCTAATTGTAGAAAGAATAAAAAGTTGCGGATAACTCTTTTTTTTACCTA

GAATCCCGATTACTAATTAAGAAGTCTCTATCAACAAGATAAAAGAGTGAATTCTTCCTT

TCGTGAAATTAGGCAAATAAAATGAATTTCGTCTTATG-------TATATAATCAAATAG

AGAAAAGATAGATATATAGTTTTTTATCTTTCTCTATCTCCCGAAAACCCCATTTGCACT

AAAAATTCCTGTTGGGTCGCATTCTAACGAATCTTTCGATAATCTGTAAGAAACTCTTTC

TTTATTAAAAATTCGAAGACAAGAACAAAAGACAAAGAAATGAAGAAAAATAATAAAGTG

AATTATAATACATATCTTTCATGTAGAAAGATGAATAAGTCCATTTATTTAGTTCTACAT

TCCTTGGACTTATTCTATATACTCACTTAGATATATAGATACTTATTTCTAT-ACTAAGA

ATTTGAAATTTAATTAATTAATAATAATTACAATTCTTAATTATAATTATTATAAGATAT

TTATTTTTTATAAAAAATAAATAATAGCAGGTACAAATAGTAAATCGAGGTACCCATTTT

ATGACAACTTTCAATTTCCCCTCTATTTTTGTGCCTTTAGTAGGCCTAGTATTTCCGGCA

ATTGCAATGGCTTCTTTATCTCTTCATGTTCAAAAAAACAAGATTGTTTAGATCTGATGG

GACCCGATCTCATCCG-TTTTTTTTTTCAAAACTTAGACTTGTAGCATAACACAGATATC

TATTTCGAAAAATATGGTCTAACGTGTAATTTCCGCCGAACATAAAGGAAAAAGTTCTTA

TGCCTGCATAAAAGGATCTATGGGTAAATGAATTCTAGCTAGTTTCAAATAGATCAGGAT

CGCTGGATGGCTAAAATGTAAAGTCGGTGGATCTATAGGTATATCAATATGTATAGTGGG

CTCATATGAAGGGTATGTTATTATTTTAGATCTAACCAATTTGATGAATTACTCCTAAAG

---------------------GTTCACATCAAACTAGTGCTAGTTGATGAGAGTTACTTC

GGAAACAAAAA-AAAGTAAAGTCAAATTCATTTGGGGTATTCTCTCAATTCCAATAAAAT

GCAATCAGATCAAGTATGAGTTGGCGATCAGAAGATATATGGATAGAACTTATAACGGGG

TCTCGAAAACTAAGTAATTTATGCTGGGCCCTTATCCTTTTTTTAGGTTCATTAGGATTC

TTATTGGTTGGAACTTCCAGTTATCTTGGTAGAAATTTGATATCTTTTTTTCCGTCTCAG

CAAATCATTTTTTTTCCACAAGGGATCGTGATGTCTTTCTACGGGATCGCGGGTCTCTTT

ATTAGTTCCTATTTGTGGTGCACAATTTCCTGGAATGTAGGTAGTGGTTATGATCGATTC

GATAGAAAGGAAGGGATAGTGTGTATTTTTCGTTGGGGATTTCCTGGAAAAAATCGTCGC

ATATTCCTCCGATTCCTTATAAAGGATATTCAGTCCGTTAGAATAGAAGTTAAAGAGGGT

ATTTATGCTCGTCGTGTCCTTTATATGGATATCAGAGGCCAGGGGGCCATTCCCTTGACC

CGTACTGATGAGAATTTGACTCCACGAGAAATTGAACAAAAAGCCGCCGAATTGGCCTAT

TTCTTGCGCGTACCAATTGAAGTCTTTTGAGAAATGTAAATATGGGCTGAAGAATGAATG

CTTTCTCAGCAGGAGGGCAAAATGAAAGAATCCTC--TTTTTTTTCTATAACATAACTTA

ACTGAAGTTTTGTCAGAACGTTAAGTCGAGCCAAAGCCGACATATATGGAACAACCATAA

AAGAAAACTCTTTTTGTGGCGTATACAAATCCACGCAACTCAATTCAACAACAAGTATAA

CAAATTGAACTAATAGATTCAATTCATCTCATATATCAAACGATTTCGAAAGAAAGAAAT

TTAAGTTCAATATTTGTTGGAATTGATACTTTAGATGCAGATAAATCATATCTTGTAAAT

TATTTC-TTTTTGTCAATCGACCTTTTTTTATCCTTTCTTTTGTATTCCTTAATAACCAA

CAATTGGTTTTCTTATAATGATAACTGGACAATTTCTGTCTTGTTTTGCTACTCATTTTT

TTTTGATCATCACAATATCTTTCTCTCAATTATTCTATTCCTGGCTATGGGTAATCGGTG

GAATTTTTTCGAAATATTGGATATTTTGATAGAAAAGGAATTCTTTCGTCTCAAAATCTC

AATAATATTCATTCCTTAAAGTGCTTCTTTCGTTCATTCGGAGACACTTGTTTCGAATTT

GACCAATTGAGATATCTGAAAACAATATTTTTTATTATTTCTTCATTCAAATTCGAAGTG

GCATCTTAGTCTATTTCTGTATTCTTTCTAGATTCAAACAAAATCACAAATAAAATAGAT

TCATAGGTTTGATATCTTGTATAGAACTCATTGGTGAAAGAAATATTCGATAGATCACAT

AGAGCCGACGAATGAGGTGGGTTGATTAACAATTCACAGATGAAAAAATGGCAAAAAATA

AAGCATTCACTCCTCTTTTGTATTTTGCATCTATAGTATTTTTGCCCTGGTGGATTTCTC

TCTCATTTACGAAAAGTATGGAATCTTGGGTTACTAATTGGTGGAATACTGGGCAATCTG

AAATTTTTTTGAATGATATTCAAGAAAAGAGTATTCTAGAAAAGTTCATAGAATTAGAGG

AAATCCTCTTCTTGGACGAAATGATCAAGGAATACTCGGAGACACATTTACAAAAGCTTG

GTATAGGAATCCACAAAGAAACGATCCAATTAATCAAGATACACAATGAGGATCGTATCC

ATACGATTTTGCACTTCTCGACAAATATAATCTGTTTTGTTATTCTAAGTGGTTATTCTG

TTTTTGGTAATGAAGAACTTGTTATTCTTAACTCTTGGGCTCAGGAATTCCTATATAACT

TAAGCGACACAGTAAAAGCTTTTTCAATTCTTTTATTAACGGATTTATGTATCGGATTCC

ATTCACCCCACGGTTGGGAACTAATGATTGGCTCTGTCTACAAAGATTTTGGATTTGTTC

ATAATGATCAAATTATATCTGGTCTTGTTTCCACTTTTCCAGTCATTCTAGATACTATTT

TAAAATATTGGATTTTCCGTTATTTAAATCGTGTATCTCCGTCACTTGTAGTTATTTATC

ATTCAATGAATGACTGATAAATGATCCACCGATATTAATCTAATCCAATTAGAATGTTTG

TTACTTTGTAGTTCTACATAAGCATTAAAAATCGTACTTACTCTTTAGATTTCTAACCAT

CCGGGGAATTCATCCTATATTATTCCAGTAAAATGATTCCAGTAAATAGCAGAATCGTGG

ATAGGGAACTATACTAGCAACCTACTCAATTTATTGTAGAAATTTTTGGATCAATTATTA

GACCATGCAAACTAGAAATACTTTTTCTTGGATAAAGGAACAGATTACTCGATCTATTTC

CGTATCGCTCATGATATATATAATAACTCGGACATCCATTTCAAGTGCATATCCCATTTT

TGCACAGCAGGGTTATGAAAATCCACGAGAAGCGACTGGGCGTATTGTATGTGCCAATTG

CCATTTAGCTAATAAGCCCGTGGATATTGAGGTTCCACAAGCGGTACTTCCTGATACTGT

ATTTGAAGCAGTTGTTCGAATTCCTTATGATAAGCAACTGAAACAAGTTCTTGCTAATGG

TAAGAAAGGAGGTTTGAATGTAGGGGCTGTTCTTATTTTACCGGAGGGGTTTGAATTAGC

CCCTACCGATCGTATTTCTCCCGAGATGAAAGAAAAGATAGGCAATTTGTCTTTTCAGAG

CTATCGCCCCAATAAAAAAAATATTCTTGTGATAGGGCCTGTGCCTGGTCAGAAATATAG

TGAAATCACCTTTCCTATTCTTTCCCCCGACCCTGCTACTAAGAAAGATGTTCACTTCTT

AAAATATCCTATATACGTAGGCGGGAACAGGGGAAGGGGTCAGATTTATCCCGACGGAAG

CAAGAGTAACAATACAGTTTATAATGCTACAGCAGCGGGTATAGTAAGCAAAATCATACG

AAAAGAAAAGGGGGGATATGAAATATCCATAACAGATCCATCGGATGGACGTCAAGTGGT

TGATATTATCCCTCCAGGACCAGAACTTCTTGTTTCAGAGGGTGAATCCATCCAATTTGA

TCAACCATTAACGAGTAATCCTAACGTGGGCGGATTTGGTCAGGGAGATGCAGAAATAGT

ACTTCAAGATCCATTACGTGTCCAAGGCCTTTTGTTCTTCTTGGCATCTGTTATTTTGGC

ACAAATCTTTTTGGTTCTTAAAAAGAAACAGTTTGAGAAGGTTCAATTGGCCGAAATGAA

TTTCTAGACTCGCGGATTTATCGACATCCAGTTCGTAAAAAGAACCAAATTCTTGTTGTC

GATTATGATTTTGTATGATAAAAAAA-AATGAAATTATGAAAAACCTTTTTCTTGTTTAT

ACTC-TTTTTCTACGGAATTCCTTGTACGGCATTCCGAGTCATAATAGGTAGATTTTTTT

TGAAGAAGACTATTTATTTGACTTTACCCCCTCTTTCTTTGTTTTTTTTAGCCAAATTGA

AGTGGTGCACCTATGTTACTATTGCCAGATTTCAATGTCATAAAATTGGACTAGATATTA

GCA-TAAGTAAGCGGGGAGCAAATAATTCTAGGAGGGATTATTCGTCTTCCTAGTCTTCG

ACACAAGA-AAGGGGTGTAGAAAATTCCTTTTCTTGTGTCGAAAGAGTAATGATTTTTGA

TCCTGTTCGTCAAAAATGCCTAGTCTTGGTTTCGGTTTTTCGAGATGTATCAGAACTTTT

TCGATTTTTTTCATATAATAATATAAAATAATATAATAAGTAGTGGACAAACAAAAAAAA

ACACAAAAAAAGAGGGAATTTTATTGATTAAATACAATGAACTTCTAAAAAATTTGAATT

TGGCTGAGATACTCAAATAAATAGAGTAATAGAAAGTATTTGTACGATATCTAGTCGACC

AAAATATATATCATCCAGGAAGTTGAGTGATTCCCCCTTTCTTCTAACTTGGAAAGTACC

C-ATAGATACTGTCAAGTAACAGGTGTTCTGAATCAATCAATGAAGTTCATTTTTCAAAA

GCATCATCAGAAAAAGTTTTTTGAAACAGCAGAAAAATAAATCTACTTTGTCATTTAGAC

GAAAAAAA-GACTCTGATTCTTAAGAACCCAACGGGCCTTTTCCCCTCGAATCAGACAAA

CAAAGAAGGGAATCCCGTTGAGTTCTTACGCTTTCATGTCTACAACTCAATTCATCCGAT

TACTACAGGGATGAACCCAATCCGGAATATGAACCATAAAAGAAAATACCTATTAAACCA

ATCACAAGAATACCAGTTACAGTACCTATTATCCAAAGAGGAATCCTTCCAGTAGTATCG

GCCATTTACTCTACTTCCCTCCAATTTCATCAAGTGGTCATGCTACAGACATAAACAGTC

ATGGATAATTATGAGATGAGATCCTTCCGAATGGGCTAAGAGAATGCCTAGAATTCTTAT

TTCTTTTCTTTCGTTTTCCTAATTGAAGAAATAATTGGAAAATAAAACAGCAAGTACAAA

AATGAGTAATAACCCCCAGTAGAGACTGGTACGATTCAATTCAACATTTTGTTCGTTCGG

GTTTGATTGTGTCATAGCTCTATAATTCGGATTAGGTTTATCGTTGGATGAACTGCATTG

CTGATATTGACCCCAAAAAAAAGACGGTAGGTACAGCTAGGCCGTGAACAGCCAACCATC

GTACTGTAAAAATTGGATAGGTTCGATCTATAGTCATTAGATTAGGGCCTCCTAAAACGA

TCTACTAAATTCATCGAGTTGTTCCAAAGGATCAAAACGGCCAGTTATTAATGGAATTCC

TTGTCGGCTCTCTGTAAAATATTCGTTTGGCCGAGGGCTTCCAAATACATCGTAAGCTAA

ACCGGTACTGACGAATAACCAACCCGCAATGAATAGGGAAGGTATAGTAATGCTATGAAT

GACCCAGTATCGAATACTGGTAATAATATCAGCAAAAGAACGTTCTCCTGTGCTTCCAGA

CATGCTCAGCTCCACATATTCTTGTACAGTCAAAGGGGATCGATTCCGTAAAAGATGAGA

TCAGTAAAAGGCAATCACTGAAATTGCATCCTTGTAGGATCGTCAATATTGTACCGAGGG

CGTCTTTAGAGTATACCGAATCAGTATAACTATCCTTCTTCTGACACAGCAACGCAATTT

GAATCAGTATCGAATCGAAAGGAAGCTTCCTTTCCTTTACCCGTTGATGTAAAATGATGC

TCTATATTAATAGAAAATTCTTACAATGAAAGAGATTATCATATTCCCACAATTTAAGTA

GATGCGCGAGATCTAGAAATTTCCTTTTCGTAGTTGTAGAAGCAGTTTTTTTTGTTGGAA

TCCTTTTTTTAATTGGTTAATCGTCCAGTAACAAATAAGAGTAGTAGAGCGTATTCGATG

AAAGAAAGCGAAGAAAGAATAAAATAATTGGAATCCATAGTTGTGATGCATTGTTGTATT

GGATCGAGATACAAATCTTGATCTAGCTACAAGGATGAGGCTTTATTTAAAAATATGGAA

AGCCAAATTGTAAAAACTAAAAACGATAATAGAAATTACTAGTTTTAGAATCTAATTGGA

TTTTTTTCTAGTGATCCATGTGATACCTTTTTTCTTCACATTCATTCAAGATATTATGGG

AATGAACCTATTACTGAATTTAATGAATTAAACTTAATTAAGGTAAAAAAAGTTTTATAA

GGTCACTGTTCACTCTAAAATAGAAAATGAATTCGATACAATTCAAAAATCGATTTTTGA

ATTGTATTCCATAAAAATTTTATTCACAAATAAAAATTCAAAAAGAGTTTCA-TTTTTGA

GTGAAGTTACACAATCCAGTTCGTATTATTAGTTTATGCTCAATGAATCGGTTGATAGGA

ATCGCGAGATGGATAAATGTTACAAATGATGAATCAATTTCGTTTTATATGCGCCTGTCA

CTTTATCTTTGTTCGTGCCATATATAATGATAGATGAATCAAAAACTTTCAATTGAACTT

ATTCTTTCAATTGGTATTTTTGCGTATCCTCCTATTTTACAAAAATAGAAATTTAGGTAA

ATGCTTTCTAAACATATGTATAAAAATAACATATTTCATTTAGCTCCTTCATGCTTACTA

TAACTAGTTATTTCGGTTTTTTACTAGCGGCTTTAACTATAACTTCAGCTCTATTTATTG

GTCTGAGCAAGATACGACTTATTTGAAATTTATATTTGAAAAAAATAATTCCTAAAAGAA

ATCTTTCTGTGAGATTCGGTGTATTCTATAGTTACTTACTGCGTAAATTCTGGGTCATTG

AGATTCACGTCAATTCGGATTAATATTTAGGTATAGATATTACCTTTTTTTTTTCTCCTT

TTCAAAAAATTGAAATGATTGAAGTTTTTCTATTTGGAATCGTGTTAGGTCTAATTCCTA

TTACTTTGGCTGGATTATTCGTAACTGCATATTTACAATACAGGCGTGGTGATCAGTTGG

ACCTTTGATTAATTAACATTTCTTTTTTTGATTGACCTCCTCCTCCTTTCTTTAATCCAC

AGGAGGTCAAATTCTGATTGCTGTGCAAGTGAATGAATCTATTTCATTCTAATTCGATCT

ACGAAGAAAAAATCACGCTCTGTAGGATTTGAACCTACGACATCGGGTTTTGGAGACCCA

CGTTCTACCGAACTGAACTAAGAGCGCTTTCTTATCAGAATAAGAGAAGACTGTAAAGAA

AAGGATTATTTTTGTAACCCTAATCCATTTTCATTTTGTCTGCATATACTATATAGTTTC

AAAAAAATGAAAGATTCTGCCCAATTTGAATGGATCTCAGTTGATTCCTCGTTACTGCTC

AAAGGAGCAGTAATAGGTAGGGATGACAGGATTTGAACCCGTGACATTTTGTACCCAAAA

CAAACGCGCTACCAAGCTGCGCCACATCCCTTCAATTGTTCTACAGTGTCATTGTAGAGA

ATTCCTGTCTTGTTTTCCACATCCTTAGTTGTTCCATTGATATACACAATTTTTCTGCCC

ATTTCGTATTTTTGGTTTTAATAAGAAAAGTAAAAAACTTATTATATATATACGAAATAT

AGAACCCATTGTAAAAAAAATGAGTATTTTTCGGAAATACTCGCTACGAGGGGATC-TTT

TTTTTTCTGTTTTAAGAAAAAGAAAATCTTATGGATCATTGTACATTTCAATTTGAATTA

GGGATTCCGTGTACAACTATAAGTGGTCCTTAACTACATATCTATCTGATCATATATGCA

TTATCTTTATCTTTATGTATTACAATAAATAAAAGAAGGAGGGTTTTCAATGCGAGATCT

AAAAACATATCTCTCCGTTGCACCAGTACTAAGTACGCTATGGTTCGGGGCTTTAGCAGG

TCTATTGATAGAGATTAATCGTTTTTTCCCGGATGCGTTGACATTCCCCTTTTTTTCATT

CTAGTTATTGACATGGGAAGGAATGAAGAAGATTAGAGATACAATCAAATATCTGTGACT

AATACTAATCCCCCC-TTTTTCTCTTTTTTCCCTTTTTAGAATAAGGGAGGAAAGAGAAA

GAATAAAAGTAGATCCAACTTCTGCGAGACTCGGGTTCAAGTTCGAATTAAATGAATAAT

GAATATTAATAATAGAGGAATGGGGGTAGAGTAGAAAATGCAGATCTAGGGCAAGAATAC

AAGAACAAGATCTTTAACTGAAATACCGTACTTCAATTTTAAATATAGTTTAGAAATCCG

TTGTCTTACTTATTATTTACTATGGCTTTGATTTATTATTACTTTATTGATTTTGATCTT

TTAGAATTGGATTTCAAGTTAGTAACTTCTATTTTTTCCTTCCTTTTCTTCGTTTCGAAT

CGAAAATAAAAGAATTGAGTAAATCAAAAATCCAAAGGAGGTTCATGGCTAAGGGGAAAG

ATGCGCGAGTAACGGTGATTTTGGAATGTACCAGTTGTGTCCGAAATGGTGTTAAGAAGG

TATCAAGGGGCATTTCCAGATATATTACTCAAAAGAACCGGCACAATACGCCTAATCGAT

TAGAATTGAGAAAATTCTGTCCCTATTGTTACAAACATATGATTCATGGGGAAATAAAGA

AATAGATCGAACAGAGTATATCTTAGTCTTTCAAGGAAGGGTAAAAAATGACATTATATA

TAACATATTTAAATAGAA-AAA-AAATCCTATTTGGGGTTAAAATGAATTACAATTAAGA

AAGAAAAATAGGATTTTGGGATAAGAAATAAACTAAACAAAAAAACACAAAAAACCATGG

ATAAATCCAAGCGACCTTTTCTTAAATCCAAGCGGTCTTTTCGTAGGCGTTTGCCCCCGA

TTCAATCGGGGGATCGAATTGATTATAGAAACATGAGTTTAATTAGTCGATTTATTAGTG

AACAAGGAAAAATATTATCTAGACGAGTGAATAGATTGACCTTGAAACAACAACGATTAA

TTACTATTGCTATAAAACAAGCTCGTATTTTATCTTTGTTACCTTTTCTCAATAATGAGA

AACAATTTGAAAGAACCGAGTCGACCGCTAGAACTACTGGTCTTAGAACCAGAAATAAAT

AGGCTTATTCTTTGTTCACTTGAATTAGAATTCCAATCAGAACTCAAACACAGATTGTTG

TTTTGTTCGACAAATCCGGGAATCCAGATTTTATTATCGTGTCGTAAG--AAAAAAAACG

AATCGGAAAAT-AAAAATTTAAATGTGTTCATTCATTTTGACTACTTTAGCATATTTTCT

CATAGTAATTTTGACTCTACCTTCCCGGAGTTCATTCTCCGGGGAACTCCGTTTAAATTA

TTCCGGTGGATTCTTTCCAATCTACTTCTTTTATGATCTCGTTGGAAATCATATAAAGAC

AATTCCTATTTGATATAGCTATTTGTGCAAGTATTTTACGATTAAGAAGCAACTGTCTCT

TGTATAGATCATGTATTAATTTACTATAACTATAAGATACTCCCTTTTCGCGAATTACTG

CGTTTATCCGAGTGATCCACAAACGACGAAAATTTCTCTTTTGCTTATCCCTATCCCGAT

GAGCCGAAACCAAAGCTCTTATTTTCTGTTGAGTAATAGTTCGAGTAAGTCTTGAATGAG

CCCCTCGAAAGCTTGATGCAAATAAACGAATTTTTGTTCTACGTCTCCGAGCTATATATC

CGCGTTTAATTCTGGTCATTGAATAAATGAAACTTTGACGAATAACTAATTGATTTCCTT

TCTTTCAGTTATTCTTTTCCCCTTTCCTGGTCTATTAATAACCAAACGGATTTTGCCAAT

GTAT--AAAAAAAAAATTCCAATGGCTTTGGCTACTATAACCTTCCCGACCACGATTTTT

TC-TTTTTTTTAGGTATTTCACTGCGAAATACGAAAGAAATAAGAAATTTTATTCTTCTA

AGTGTGAAAAATATAGTAAAAAGAAATATAAATTAAATGGATAAAGAAATAGTGGGTTCC

GTCGTTTCTATGGTTACTTCTTAAACGGTGAGGTCTTCTCTATACACCGGAGCCTTTACT

TCATTTAATCAATGTTATTGGTAACTTGTATAGTTCACACCACACTCTTTGGCTCTACCC

ATGAATTATCCAGTAATAGGTCTTTCACAATGAGATCCACCTATACAGTAACGGTATTTA

ATTAGGAAAGTTAGCTGGGTAGCTGACCCTCTTAGTCCGTTCTTGACAGAGTGGGAGCTT

CATTTTTCTGTTTTTGAAATTGAAATAAGATTTCCTCCGCTTAATAGATAACCATTTGCT

ACCAATGGAGAATTGCTTCTCATCTTAAATTCAGGTGATTGGATTTGCACCAATGGAAAC

CATAAACTTCATACACAATAGAGGGATCGATTTGCTTATTTTTAGATAGTGAATGGGGTT

CTTTCTTCCATTCTATCCTATTTACTGGTACTGATCATTGATACTGGAAAGCGGTTTTCT

TGCTT-TTTTTTGTGCCAGCTCATGATCTAAACGAGTCGCACATACACCCTAGTACATGT

TCCTCGACGCTGAGGACATCCCCGAAGAGCGGGGGATTTCGTGACATTTCGAATTGGCTG

TCTTGTATTTCTAATAAGTTGTTTAATAGTTGGCATGTTGAATCATATACATAATGGGCT

GGTTTAGATTGATCCTAACCGGATGATTATGAATTATTTCTATTTAATAGAATATTAAAC

TCGTAGATAAAATCTCAAATCACGGATTTTTATAAAATCCATCTTATTTTCATTCAACTG

CTACAAGATCAACAATTCCATAAGCTTGGGCTTCTGTTGCTGACATAAAAACATCTCTTT

CCATGTCTTCAGATACAACCCATAAGGGTTTGCCCGTTCTTTGTACATAAACCTTTGTGA

GGGTTTCGCGTAGTTTCAGCAGTTCTTCCGCTTCCAGGACAAATTCTCCCGTTTGTGCCT

CATAAAAAGAACTAGCAGGTTGATGGATCATTACCCTGATGATATAACAGTTCTCTATCT

CGCGTGATGAAACGAAGAGAAAAGAAAGAAAGATAAAGAATAATAGGAAAA-AA-AAGAT

AGAATTGAACAACCGTACGGGCATTATCTTTTGTGCATTGCATACGGCTCTACAATAAAA

TTGACCCTTACCTTCCATTGAAGAAAGAGAAAAATAGAATCTATCAGACCCAGATGGATA

AATGATCAAATTGCCACCCTTCCTTTCAGAGGAGTTAAAAAATACTATGATGGCTCCGTT

GCTTTCTATTTTTAAATTGATTCTTTTTTTTGTCTTTGATTCAGCAATCCCAAAGTTTCT

TTTTGATCCAATCAAATAAGGAAAAATCTTGTTTTTTTTTCGCCCTCTTTTTTTATAACA

TAAATATTGTTAAGAGCCCTTCGATGTGAAAACAAAAAAGTTTGTGACGCTGAACTGGAC

TCCCGATAGATAAGAGAAATCGGAAATACCTTTTATCTCATACTACTCTCTCGATACATA

ATCGAATCTTTTGAAAAAAAAACAAGACAAAAATTTTGCATATCGAATTCGAAGTGCCAT

GCTATTATTACTTAATATTCATATGGCGAAGGCATAGTCTTCTTTTTTCTCTCAAAT--A

AAAAAACCTCATTGGCGCCAAGCGTGAGGGAATGCTAGACGTTTGGTAATTTCTCCTCCA

ACCAAGATAAAAGATCCCATTGATGCGGCTAATCCCATGCATATTGTATGGACATCTGGT

CGCACAAATTGCATAGTATCGTAAACAGCTACTCCAGGTATTACCCATCCGCCAGGAGAG

TTTATAAACAAATACAGATCTTTGGTATCATCCTCGATACTGAGATATACCATAAGACCA

ATAAGTTGATTCGAGAGCTCGCTATCAACTTCTTGGCCTAAAAAAAGTAATCTTTCTCGA

TAAAGTCGGTTGATTAGGGTAAAATTGTATCCCTTAGGAACCGTACATGCACCTTTTGAC

GCATACGGTTCAAAAAATAATTGCG--AAAAAAAAAGAATCAATGTATAGATTCAAGTCC

TCTTTCTTTGTTCCTATTCTTTTTTCATAGCAGGTTTTTTCTGACTTCTAATGAAAGGAC

TTTTTCTTCGATTTTTCAATAAAGACGAATTTGAACTTCTTTCTTCCTTAGAATAGAAAA

AAAGTCACTAAACTTATCGAATTAACTTCTCATTGATGTATTGTTTCATCGAGATTCAAT

CCAAATCACGATGGTATTTTCTTGTTCCTGAATGGGTCTCTTTCATCTTTTTAGGTTTAT

GCTCTACTCCGGGTAAAGATCCGCCCGATTTTGATTTGCACATATAGGACAAATCTTCCC

ATTACCATTTCTTTTTGTTATGACTTTCTTTTTTTTTTCAATTCATTTCATACCTTTCAC

CAAGTATTTAGTTTGAGATTCCCTCGCTTGACAAATAGGATCTCTTTACAAATACCAAAC

AGGAATCATTTATGATACAAGTAGTAATCATAGATATATTACCAATTGGGTTTTTTCTAA

ACGGAGCCTGGATACTTCATTTTTTAGTCCAACCAAGCCAACCATAAATTATTCTAATTG

ATAATAGTAATGTGAATCCCCCCAAACAATGGATCTAATTGCGCTTCACGCTCCAAATTT

TTGATGATTCAATTTATCTTTC-TTGGGCGAAACAGAGGATATCTCGATCGGGGGAGAGA

ACGGGGAAATCCCATATGACCCAATATATCTGACAAGTCGCACTATACGTCAACCCAAGC

TGCATCTTCCTCTCCAGGACTTCGGAAAGGTACTTTTGGAACACCAATAGGCATTAATTG

AAAGAAAAAAGAACTAAGTACTATATTTTACTTTGATGTGGAAACGTAACAACATTATTT

TATTGTCTTTATAATATTGGTTTTATCGTATTTATTTTATCCATAGATTAGAAAAATTCA

TAAAGAAAGACAAAAGAAGAAATAAAGGAAAATTTTGACGAATAGGGCCTTCTAATGAGG

AATAAGGAAGGACACATTTACTGATAGAAAATGGTATCAACCACCCATTGCGTATTGGTA

CTTATCGGGTATAGAATAAATCTGCTTCTCTTTGTTCCTACGAATAGAATTGTTTCATTA

TTACCAATAGAATAGAACAAATAGTAACCCTTGTTCAGTGGATTATTTCAGAACAAGGGG

AGTCCATAGAATAGTCATAGTATAGCTTTTCCAATGCAATAAAGTTACGTAGTGTCTATT

TATCTTTGATAAAGAGGTATTTTCCATGGGTTTACCTTGGTATCGTGTTCATACCGTTGT

ATTGAATGATCCCGGTCGGTTGCTTTCCGTTCATATAATGCATACAGCTCTGGTTGCTGG

TTGGGCAGGTTCGATGGCTCTGTATGAATTAGCAGTTTTTGATCCTTCTGACCCTGTTCT

TGATCCAATGTGGAGACAGGGTATGTTTGTTATACCTTTCATGACTCGTTTAGGAATAAC

CAATTCATGGGGAGGTTGGAGTATCACAGGAGGGACTGTAACGAATCCGGGGATTTGGAG

TTACGAAGGTGTAGCTGGGGCACATATTGTGTTTTCGGGCTTATGCTTTTTGGCAGCTAT

CTGGCATTGGGTCTATTGGGATCTAGAAATATTTTGTGATGAACGTACAGGAAAACCTTC

TTTGGATTTGCCCAAGATCTTTGGAATTCATTTATTTCTCTCAGGGGTGGCTTGCTTTGG

TTTTGGTGCATTTCATGTAACAGGCTTGTATGGTCCTGGAATATGGGTGTCCGATCCTTA

TGGACTAACGGGAAAAGTACAACCTGTAAATCCGGCGTGGGGCGTGGAAGGTTTTGATCC

TTTTGTTCCGGGAGGAATAGCTTCTCATCATATTGCAGCAGGGACATTGGGCATATTAGC

GGGTCTATTCCATCTTAGCGTCCGCCCGCCACAACGTCTATACAAAGGATTACGTATGGG

AAATATTGAAACCGTACTTTCCAGTAGTATCGCGGCTGTCTTTTTTGCTGCTTTTGTTGT

TGCTGGAACTATGTGGTATGGTTCAGCAACTACCCCCATCGAATTATTTGGGCCCACTCG

TTATCAATGGGATCAGGGTTACTTCCAGCAAGAGATATACCGAAGAGTTAGTGCTGGGCT

AGCAGAAAATCAAAGTTTATCAGAAGCCTGGTCTAAAATTCCTGAAAAATTAGCTTTTTA

TGATTATATCGGCAATAATCCGGCAAAAGGAGGATTATTCAGGGCAGGTTCAATGGATAA

CGGAGATGGAATAGCGGTTGGATGGTTAGGACACCCTATCTTTAGAGATAAAGAAGGGCG

TGAACTTTTTGTACGTCGTATGCCTACTTTTTTTGAAACATTTCCAGTCGTTTTGGTAGA

CGGCGACGGAATTGTTAGAGCTGATGTTCCTTTTAGAAGGGCAGAATCGAAGTATAGTGT

TGAACAAGTAGGTGTAACTGTTGAGTTCTACGGCGGCGAACTCAATGGAGTCAGTTATAG

TGATCCTGCTACTGTGAAAAAATATGCTAGACGCGCTCAATTGGGTGAAATTTTTGAATT

AGATCGCGCTACTTTGAAATCCGATGGTGTTTTTCGTAGCAGTCCAAGGGGTTGGTTTAC

TTTTGGGCATGCTTCGTTTGCTTTGCTCTTCTTCTTCGGACACATTTGGCATGGTGCTAG

AACCTTGTTCAGAGATGTTTTTGCCGGTATTGACCCAGATTTGGATGCTCAAGTAGAGTT

TGGAGCATTCCAAAAACTTGGGGATCCAACGACAAGAAGACAGGTAGTCTGATACAAGAC

TGCTTTGGTATCTTTCGCCTCTATTTTCTTTTTTGGGGGGAATTTTACATAGAGTACCGG

AGTTGATTTGAATCACTGCTTTTTTGACTCTTGCTCTTTCTTTATCCGAGAGATGATTCC

CAAAGAAACAAAAAACAAACAGGTATGGAAGCTATAATTGTAAACCACGATCGAATCTAT

GGAAGCATTGGTTTATACATTCCTCTTAGTCTCGACTCTAGGGATAATTTTTTTCGCTAT

CTTTTTTCGAGAACCGCCTAAAGTTCCAACTAAAAAGCTGAAATGATTTTGCATTATCTC

AATTGAAGTAATGAGCCTCCCCATATTGGGAGGCTCATTACTTCAACTAGTCCCCATGTT

CCTCGAATGGATCTCTTAGTTGTTGAGAAGGTTGCCCAAAAGCGGTATATAAGGCGTACC

CAGTAAAACTGACAAGTAAACCAGATATAAAGATGGCGACTAGGGTTGCTGTTTCCATTA

TGATTATATAATTTCAAGATCCCAATGGATCTATGATAAGATCGTTTATTTACAACGGAA

TGGTATACAAAGTCAACAGATCTCAATGAATACAATAGGATTTATGGCTACACAAACTGT

TGAGAACAGTTCTAGATCTGGTCCAAGACGAACTACTGTAGGGAGTTTATTAAAACCATT

GAATTCGGAATATGGTAAAGTAGCTCCCGGGTGGGGAACGACTCCTTTGATGGGTGTCGC

AATGGCTCTATTTGCGGTATTTCTATCTATTATTTTGGAGATTTATAATTCTTCCGTTTT

ATTGGATGGAATTTCAATGAATTAAATCTATAAGAACCGCAAAGTCCTGTCTTTTGAATA

AAAAAATGAATCAGTTAGAGCTCGGATTTCCAGCCTATTCTATTTTGGTAGTTCGATCGT

GGAATTTATTTCTTTCTGTATTTCCGGAATATGAGTGTGTGACTTGTTATAATTGATCCT

ATTGATAGTACAGAGAATGGGTCTGTTATCTTGATAGAGATGTTTCTACTTCGTCGGATA

TTTATTCTAGTATCTGGAACACGGAATATATGAACTAGATTAAGAAATATTTGAACTATG

ATTCATACTTAATATTCGACCTCGTGTCTGGACTCCAAAAAAAA-TTCAAAGAATTAGAA

TAAAAAAAAATTTTTTTTTAGTCTATCTATTGATGGAATAAGTGATGATCCAACGGTTCT

TACTCAGATAATCCTTGGCTTAACTTAGTTGAATCATCGTGGTTTTAGTATGAATTTGAG

GTTTGAATCGATTCATAGGGTCTTAACAAGATAATTCCTATCAATTCAATAATAAAGAAA

ACAAAAAAAGCCACATTAGATACATTAGATACAAAAACAAATTAAAGAAATAGTTAAAGA

GATAATTCAAGAGGCCCGTAAGGATCAACATAAAGACGATTGAGCCAACTTGATATTTTG

GTATTATCGCCACAAAGAAGAGCTTTCGGATTA-TTTTTT-TTATTCTTTCGTACATTCA

GATAAGATTGAATCAGAGATTAAGAAGTTTCAAACTTTCTATTACATATCCGTTGCAACT

AGTATTTGGGTGTTTTTGCTTGAGCTGTACGAGATGAAAGTCTCATATACGGTTCTGAGA

GGGGGATTTTCACCTATCTCAATAAAGTCTATGATTGGTTCGAAGAACGTCTCGAGATTC

AGGCGATTGCGGATGATATAACTAGTAAATACGTTCCTCCCCATGTGAATATATTTTATT

GTTTAGGGGGAATTACGCTTACTTGTTTTTTAGTACAAGTAGCTACGGGGTTTGCTATGA

CTTTTTACTATCGTCCGACCGTTACAGAGGCTTTTGCCTCTGTTCAATACATAATGACGG

AAGCTAACTTTGGTTGGTTAATCCGATCAGTTCATCGATGGTCCGCAAGTATGATGGTCC

TAATGATGATCCTGCATGTTTTTCGTGTGTATCTCACGGGTGGATTTAAAAAACCTCGCG

AATTGACTTGGGTTACAGGTGTGGTTCTGGGGGTATTGACCGCATCTTTTGGCGTAACTG

GTTATTCCTTACCTCGGGACCAAATTGGTTATTGGGCGGTGAAAATTGTAACAGGTGTAC

CTGAAGCTATTCCTGTAATAGGATCGCCTTTGGTAGAATTATTGCGTGGAAGTGCTAGTG

TGGGACAATCCACTTTGACTCGTTTTTATAGTTTACACACTTTTGTATTGCCACTTCTTA

CTGCTGTATTTATGTTAATGCACTTTCCAATGATACGTAAACAAGGTATTTCTGGTCCTT

TATAGAGAAGATATATAATAGATATTTGTAATCAATCATTTATCACTTGGAGGAGGAATA

ATAGTATTTCATTGCTACAAGTATGGATTATTGAAAATAATAATCCATGTATTTGGATAT

TTCCCTTCAACCAATCATGTCAAATAAATGTATAGTTGAGGGGAATTCTACGAAGAGAAA

ATGGATTATGGGAGTGTGTGACTTGAACTATTGATTGGTCTGTGTAGATATATGTCTGCC

ACATTGGAATTCACAACCAAATGTGTCTTTGTTCCAACCGTCGCGTAAGCCCATACAGAA

GATAGGCTGGTTCACTTGAAGAGATTCTTTTCTATGATCAGACCGAATCATGTTGTACAT

GAGCAGGCTCCGTAAGATCCAGTATAATTATAA-TAAGTGAAATGGATAAACTAAAACAG

AATCTTTATCTATTTCACTTACTTAATACTTAAAATTGAATAGTATGGAAATGCATTTAT

TTCCTCTGCATTGCTATGATCGATAATACTATCGGAGTGAAACAAGGGATTTAAAGAAAA

ACATAGGCTAGACTAGATTAGTAACAAGTAAACCTTTTGTGTG--TATCTCCAAATA-TT

TTGGAGATAAATACCAATCGTAAGGTCTGAGACGACCCAGAAAGCACTCGATCATATCAT

GATCAACTTTGTAAGCCAACTTGGGTATTGAGTATTTACTTAGAACCGAATTTTTTGCAA

TGGGTAGTTGCAATTCCGGAAAAAGAGTCAAATTTTTCTTACATTGAATCATTCATATAT

GTGTATATCATATATGTGTATATATAGGCAACATATAGATTTTTTATGGATTCATTTGGT

TCTTTTGAATCTTGCTCGAGCCGGATGATAAAAAATTATCATGTCCGGTTCCTTCGGGGG

ATGGATCTATAAGAATTCACCTATCCCAATAACAAAAAAACCTGATTTGAATGATCCTGT

ATTAAGAGCTAAATTGGCTAAAGGTATGGGTCATAATTATTACGGAGAACCCGCATGGCC

CAATGATCTTTTATATATTTTTCCAGTAGTAATTCTAGGTACTATTGCATGTAACGTAGG

CTTAGCGGTTCTAGAACCATCAATGATTGGTGAACCGGCAGATCCGTTTGCAACCCCTTT

GGAAATATTACCTGAATGGTATTTCTTTCCCGTATTTCAAATACTTCGTACAGTGCCCAA

TAAATTATTGGGTGTTCTTTTAATGGTTTCAGTACCTGCGGGATTATTAACAGTACCCTT

TTTAGAGAATGTTAATAAATTCCAAAATCCATTTCGTCGTCCAGTAGCGACAACCGTCTT

TTTGATTGGTACTGCAGTCGTTCTTTGGTTGGGTATTGGTGCAACATTACCTATTGATAA

ATCCCTAACTTTAGGTCTTTTTTAATTTGATTCAATTGTGAAATAACACGACGTGTGTAT

CTAGGGAATAGTCGCTTGAAAGCGAATTCTCCCTAGATACATCTATTCAATTCTGAATTT

CTTTCGAATATATGAATTGTGCTAAAGATTCAAAACCTATTTTCATCTTAATG-------

--AAAAAAAATCCAATAGATTTAAAACTTCTTTTTTGGTAAATCAATTGCGAAATGTTTT

TCTAGAATGACCAATATCTGTTTTATATCTTCTAGGCGCAAATGTTCAATTTTCATGAGA

TCTTCCGGACTGTTATTCAAAAGGTCCAATAATGTATATATATTGGACCTTTTGAGGCAA

TTATAGACCCTGGGAGAGAATTCTGATTGGTCAATAAAAATCGGTTTCAATGCTATTTTT

TTTTTGTTTTTTCTGAGTTTATCCAATTTATCGTGAAAGGTAAGAGGGGATAAAGGAACC

GTGTGTTGATTGTCCTCTAAAGGTAAGTTTTCTTCTTCCTTATGTAAAAAGGGAATAAAT

AAATCAATCAAATTCCGGCAGGCTTCATGAAGTGCTTCTTTCGGAGTTAAACTCCCATTT

GTCCATATTTCGAGAAAGAGTATCTCTTGTTTTTCATTCCCATTCCCATAAGAATGAATA

CTATGATTCGCATTTCGAACAGGCATGAATACAGCATCTATAGGATAACTTCCATCTTGA

AAGTTATGTGGCATTTTGATAAGATATCCGCGATTTCTCTTGATTTGTAATCCAATACAC

AAATCAATTGGTTCTGTCAAGCTAGCTATATGTTGTGTATTATCAACGATTTCTACATAA

GGTGGTAAGATGATATCTTGAGCAGTTACATATCCTGGACCTCTGACACAAATAGACGCG

TCACAAGTTCCATATAGATTACTTCTCAATACAATTTCTTTTAAATTCATGAAAATTTCA

TGGACCGATTCTTGAATACCCGCTATGGTAGAATATTCATGCGGGACGTTCTCAGATTTT

ACACGTGTGATACATGTTCCTTCTATTTCTCCAAGTAAAGCTCTTCGCATCGCAATGCCT

ATTGTGTCGGCTTGACCTTTCATAAGTGGAGACAGAATAAAGCGTCCATAATAAAGACGT

TTACTGTCTTCTCTTGATTCAACACACTTCCACTGTAGTGTCCGAGTAGATACTGTTACT

TTCTCTCGAACCATAGTAATATTATTTGATTTGATTGAATCATTTATTTCTCTTGTTTCT

CTTGAAATTTCTTCAATGTTAATTTCTACACACGTCTTTTTTTCGGGGGTCTGCAACCAT

TATGTGGCATAGGGGTTACATCCCGTACGAAAGTTAATAGTATACCACTTCTACGAATAG

CTCGTAATGCTGCGTCTCTTCCGAGACCGGGACCTTTTATCATGACTTCTGCTCGTTGCA

TACCTTGATCTACTACTGTACGAATAGCATTTGCTGCTGCAGTTTGAGCGGCAAAGGGTG

TCCCTCTTCTCGTACCCTTGAATCCACAAGTACCCGCTGAGGACCAAGAAACCACCCGAC

CCCGTACATCTGTAACCGTGACAATGGTATTATTGAAACTTGCTTGAACATGAATAACCC

CCTTTGGTATTCTACGTGCACTCTTACGTGAACCAATACGTCCATTTCTACGTGAACCAA

TTCTCGGTATAGCTTTTGCCATATTTTATCATCTCATAAATATGAGTCAGAGATATATGG

ATATATCCATTTCATGTCAAAACAGATTCCTTATTTGTACATCGAGTCCTTTAGTGAGTC

TGATTATCCTTGTCTTTGTTTATGTCTCGGGTTGGAACAAATTACTATAATGCGCCCCCG

CCTACGGATTAGGCGACATTTTTCACAAATTTTACGAACAGAAGCTCTTATTTTCATATT

TGTCATTCCTTATCTTAATTCTGAATCTACTTCTTGGAAGAAAATAAGTTTCTTGAAATT

TTTCATCTCGAATCATATTGAATAAAAACCACCTAATCCTTCCAATCCTTTTTGCGGAGT

CGATAAATTATACGTCCTCTGGTTGAATCATAACGACTTACTTCAATTTTGACTCTATCT

CCTGGCAGTATCCGTATAAAACTACGCCGGATCTTTCCTGAAACATAACCCAGGATCAGA

TCTTCATTATCTAACCGAACCCGGAACATACCATTGGGAAGCGATTCAGTAATTAAACCT

TCATGAATCCATTTTTGTTCTTTCATTCCAGGTAAAGCCTCCTTGAAGTATCAACTAATG

GAGGAGGAACGATATTAGACAACTCGTCCCTTTTCTTTTTTTTAGAAATAGGAAGAAGTT

TCGGATCCAATTTGGATATTAAAAGGATTACCATATATAACACAAAATTTCTCCGCCGAT

TCCTTCGAGTCGAGCCTCTCGGTCTGTCATTATACCTCGAGAAGTAGAAAGAATTACAAT

CCCCATCCCACCTAAAATTCTAGGAATTCGTTGAGAGTTAGAATAGATTCGTAGACCGGG

TCGACTGATCCGTTTTAAATTTAAAAAATTTCTATAGAGTCTTTTCCTATTCCTTCTATG

CCGCAGGTTTAAAACCAAAAAAGATTTGTTTTTTTCTCGATGTTTTCTAACGTTTTCAAT

AAAACCTTCTCGGAAAAGTATTTTAACAATATTTTCGGTAATATTAGTAGATGCGATGCG

AACCACTCTTTTTCTATCCATATCAGCATTTCGTATAGAGGTTATTATCTCAGCAATAGT

GTCCCTACCCATGGTGAACTAAAATTATGGGTGCCCCAAAATTGGATATAATCAACATGT

TTTTCTTTTTTTTTTTTACTT-ATTTGTTTTATGAATATGAATTATTAAAGGTATATGCG

TGAGACACAATCTACTAATTAATCTAGTTCTTAATCTATTTCTTTCAAATACCCACTATA

AACATATCAGTGATCTCATTTTATAATACCTCGGGAGCTAATGAAACTATTTTAGTAAAA

TGGAATTGTCTCAATTCCCGGGGGATCGCACCAAAAATTCTAGTTCCTTTTGGATTTCCT

TCTTGATCAATCACAACTGCAGCATTGTCATCATATCGTATTATCATACCGCTGTCACGT

TTAAGTTCTTTACAGGTACGAACAATTACAGCTCTGACTACTTCTGATTTTTCTAGGGGC

ATATTTGGTACTGCTTCTTTGATCACAGCAACAATAACGTCACCAATATGAGCATATCGA

CGATTGCTAGCTCCTATGATTCGAATACACATCAATTCTCGAGCCCCGCTGTTATCTGCT

ACATTTAAATGGGTCTGAGGTTGAATCATATCAATTTTTTAGTCTATTCTTCCAATGCAA

AGGATGAAG-AAAAAAAAGGAATATTTTTTGTCCAAAAAAAGAAACTTTCATCCCCAAGA

TTCTTCTGTTGGTTCTACATTTTTATCCTGAAATAATGAATTGAGTTCGTATAGGCATTT

TGGATGCTGCTATTGAAATAGCCCTTCTAGCTATATTTTCGGTTACTCCACCCATTTCGT

ATAGTATTCGACCTGGTTTAACAACAGCTACCCAATATTCAGGGGATCCCTTTCCCGAAC

CCATACGTGTTTCAGCGGGTCTTACTGTAACCGGTTTGTCTGGAAATATACGGACCCATA

TTTTTCCACCACGGCGTGCATTTCGTGTCATTGCTCGTCGACCTGCTTCGATTTGTCTAG

ATGTGATCCAAGCAGGTTCAAGTGCCTGAAGAGCATATTTACCGAAACAAATATGATTAC

CTCGATAAGATATTCCCTTCATTCTTCCTCTATGTTGTTTACGGAATCTAGTTCTTTTGG

GGTTATAGTTGATGGTTGTTTCACAATTCCATCTCTACTACAGAACCGGACGTGAGAGTT

TCTTCTCATCCAGCTCCTCACGAATAAAAGGATTAAAAACATTTAATTGAAATGATTAAC

ATTTTTTGTAAAAAATTTTTTTTTTAGAACGTTCTAAAAAATCTTTATTTTGTTTTGTCC

TTTATCCAAGTTTAGCAAAAAAAAAAAAAAGTTTTCGCGGGCGAATATTTACTCTTTCAA

TCTCTATTTCATTTGTAGGGCTCGTTCGTGACTTCTCCCAATAGATGAATTAATCTCCGG

TTCATTTCGCCATCCCGACTAGTGAATCATTAAGATTCATTTTTTCAATAAAATCTTTTG

CATGCACAGGTTCCATCGTTCCCATCGCTTCGTACTTAATGATTAGGTCCGAATTCTACA

ATGGAGCTCATAATCCAATTTGTTCCTGAGTCAATCTTCTTAGTCTTTATTGGCTCCAAG

CTCTTTATTTTTTTCTTGATTCATTTAATATTTATGAATCAATTAGTATTGATGCTTTAT

TACACTGTCTTTTATGAGATGACTCGTA-ACCTTACATATTGGAATTCTATATCATTGAT

ATTCTTTTTCTCTCTTTCTCTCATCCTTCCATTTATCCACACCTTTACTTCTTTCATTTT

GTTTTACAACTTCTAATTAAAGTTTATGC-AAAAAAAAATTTCAGTTGCTACAAAGATAT

GACTGATTTATCATATCTTGACTGGTTCTTTATATCCAGATAATACGAAGTGATGAGTTG

GTTATTAGTTCATACTATGGGGCTGGTCCTTTTTTAATCCTAACCCTAAAAAACCAACGA

GTCACACACTAAGCATAGCATTTATATCAAATGGTCAATTGAATTTTTATTCAACCCTAT

AGAATTAAGAATTAGAATTGCTCATTTTGATTCACCAGAAAAAGAATGAACGAGTTTCTA

TTTTTTTTTCTATCAATGGATAGAAGGGAAAGACAAGTAAAGGTTTCTTATTCTTCGTCT

ATAAATATCCAAATTTTGATACCCAAGACCCCATAGATAGTTCGAACTGTATAGGAACAA

TAATCAATTTTAGCTCGAATGGTTTGTAGGGGAACCCTACCTTCTCTGATCCATTCGACA

CGTGCAATTTCTTTTCCGTCGATACGTCCTGCAATTTGTACTTGAATTCCTTTTGTATCT

GCTTGTTCAGTTAATTCAATAGCTTTTTTCATTGCTTTTCGAAATGAAACTCTATTCTTT

AATTGTCCGGCTATAAATTCTGCAAGAATATTAGGGTTTCCATAAGGTTTTGCAATTCTT

GTGATAGCAACGTTAAGTTTTTGGTTCACATAATTAAATTCTTTTTCTAGATTAATCTGT

AATTCTTGGATTCCTCGCGTTCTATTTTCTATTAATAACTTTGGGAATCCCATAAAGATT

ATGACCTGGATCAAATCAATTCTTTTTTTAATTTCTATACGTGCAATTCCCTCGACGCCG

GAGGATATTCTCATATTCTTTTGTATATAATTTTTGATAAAATCTCTTATTTTTTGATCT

TCTTGTAAACCCTCAGAATAATTTTTGGGTTGTGCAAACCAAAGGGAATGATGACCTTGG

GTTGTACCAAGTCTGAAACCAAGTGGATTTATTTTTTGTCCCATACTCCCCCAATACTAT

ACATATCATGATATACCATAGTTGTATACTTATTTTTCCATCTAGGTTTTTTTAACGAAT

ATATCTGTCCATATTCATCATGTAAAGATATATCTTTCACTCCAATAGTTATATGACAGG

TAGGCCTTTTTATCGGATAACTACGTCCTCGAGCTCGAGGTTTGAATTTCTTCACGGTAG

TACCCTCGTTAACTTCGGCCTTACTAATGACTAAATTGGCTTCGTTGGAACCCATATTGT

AACTAGCATTTGCTGCTGCAGAGTAAACCAATTTAAAAATGGGATAACATGCTCTATAGG

GCATGAGTTCTAGTATCATAAGTGTTTCCTCATAGGAGCGTCCACGAATTTGATCAATTA

CTCTTCTTGCTTTGTCAGCCGATAGAGATATATGTCGACCTAAAGCGTATACTTCTGTTT

TTTTCTTCTTTCGCATAGGGTTTACCTCCTATTAATGAATCATAAGTATCTATCTATAAT

ATATATAAAAAAAAA--AGATTAACGCCGAGATCTATTATCGCTTTTTGCATGTCCTCGG

AAATTTAAAGTAGGTACAAATTCTCCTAATTTGTGTCCTACCATAC-GATCTGTTATATA

AGTAGGCAAATGTTCCTTACCATTATGAATAGCAATTGTATGGCCAATCATTGTGGGTAT

AATGGTAGATGCCCGGGACCAAGTTACTATTATTTCTTTTTCTGCTTTTTTATTAAGCTT

ATCAATTTTTCTTAATAAATGATTGACTACAAAAGGATTTTTTTTTAGTGAACGTGCCAC

AACTTCCTCCTATTTTTTTTTTGTTTTTGTAAAGACGAAGAAAGAAATTCTATTTTCTCG

CCTATTTACTACGGCGACGAAGAATCAAATTATCACTATATTTATTCCTTTTTCTACTTC

TGCTTCCAAGTGCAGGATAACCCCAAGGGGTTGTGGGTTTTTTTCTACCAATTGGGGCTC

TCCCTTCACCACCCCCATGGGGATGGTCTACAGGGTTCATAACTACTCCTCTTACTACAG

GACGCTTACCTAGCCAACGCTTAGATCCGGCTCTACCCAAACTTTTCTGGTTCGCCCCAA

CATTCCCCACTTGTCCGACTGTTGCTGAGCAGTTTTTGGATATCAAACGGACCTCCCCAG

AAGGTAATTTTAATGTGGCCGATTTCCCCTCTTTTGCAATCAGTTTCGCTACAGCACCCG

CTGCTCTAACTAATTGTCCACCCTTTCCAAGTGTGATTTCTATGTTATGTATGGCCGTGC

CTAAGGGCATATCGGTTGAAGTAGATTCTTCTTTTTGATCAATCAAAACCCCTTCCCAAA

CTGTACAAGCTTCTTCCAAAGCATACGGCTTTCTGGATGTAGATGATGATATCTATACAG

ATGGATCTTTTATATATCGTACAATGAAGTACCACA----TGGATATCTATATGAATCCA

AATCTGCCGAATCACTCATGGTATGATCTTCTACATCCTAGGTCTTCCCGTTCCGTCATC

TGGCTTATGTTCTTCATGTAGCATTCAGACCGAATGACTCTATGAAATTACGTCGATACT

TCCACATATTATGGGTAACGTAGGAGACATCTCTATTTTTCCCCCGGGGAATCTTTAGAA

TTCCCACTGCTTAGCTTTCAATTCGCCTCTGACCATCAAATGAAATGTGAATAACCCGTC

CTCCTCTCTTTGAAAGTTTGAAAGAAGGGGCGCTTCCGGTTCTGTCGGTGCTTGAAACAA

TTTTGTCTTCTCCATATTACTATATCTCTATAGTCAATAATTTTATATGAGGAACTACTG

AACTCAATCACTTGCTGCCGTTACTCTTCAGTTTTCTGTTGAGGTCTATCCTGTAGAGGT

ACTCAAATTGGATCAGTGATCGATTTCTAGGTTTCGTCGTAAACCTAATTGGTTACTTCC

AATTACGTAAATCAATAGTTCAAACCGCACTCAAAGGTAGGGCATTTCCCATTTTTATAG

GAACTTCTGTACCAGAAACAATGGTATCTCCAATTATAGCCCCTCTGGGATGTAAAATAT

ATCTCTTCTCACCATCCCCATAGTGTATGAGACAAATGTATGCATTTCGATTAGGGTCGT

ATTCTATGGTTACGATTCTACCATATATGTCTTTTTCATTCCGTCGAAAATCGATTTTAC

GGTATAGACGCTTATGACCTCCCCCTCTATGCCTTGCGGTAATGATTCCTCTGGCATTAC

GACCTTTACCACAATGATGCTGTCCATAGATCAAATTATTTCGTGGATTGGATTTCACTT

GACTGTCTACGGTTCCATTGCGTGTGCTCGGGGTAGAAGTTTTGTATAAATGTATCGCCA

TGCTATTAAGTATTTTGATTTAAGTTCTTTTCTTTCTAAGAGGTGGAATAGAATAACCCG

GTTGAAGCGTAATGATCATACGTCTGTAATGCATTGTATGTCCCATAATAGGTCCCATTC

TTCTACCCTTTCCCGGAAGTCGATGACTATTCATAGCTATTACCTTGACACCAAAGAAGA

GTTCGACCCAATGCTTTATTTCTGTCCTAGTTGATCCTGATTCGACATTAGAAGTATATT

GATTTTTCCCCAATAACCGAATACTTTTGTCTGTAAATACTGCATATTTGATTCCATCCA

TAAATCGATTTTCTTCCCTATGAGTTCTAGTCTCAATAAGAATGCTAGTTCTTACTGTTC

ATATATTATGATATGAATATACCACACCAATTCGTTATGTATGGATGATGAGATTCCATT

GATACAGAGCCAATTCCAATAGACTTATTGGAGGGTCCCATTGGCGTGCATCCAGTAGGA

ATTGAACCTACGAATTCGCCAATTATGAGTTGGGCGCTTTAACCATTCAGCCATGGATGC

TTAGCGGGGATCCTCGTACATGGTGAATAACCAAATTCCAATTGAAATGAAATCTTTAGG

ATAAATCAATGCAATTTAGGAGGAATCAATGAAAGGACATCAATTCAAATCCTGGATTTT

CGAATTGAGAGAGATCAAGAATTCTCACTATTTCTTAGATTCATGGACCCAATTCAATTC

AGTGGGATCTTTCATTCACATTTTTTTCCACCAAGAACGTTTTCTAAAACTCTTTGACCC

CCGAATTTTGAGTATCCTACTTTCACGCAATTCACAGGGGTCAACAAGCAATCGATATTT

CACGATCAAGGGTGTAATACTCTTTGTAGTAGCGGTCCTTATATATCGTATTAACAATCG

AAATATGGTCGAAAGAAAAAATCTCTATTTGAGAGGGCTTCTTCCTATACCTATGAATTC

CATTGGACCCAGAAATGATACATTGGAAGAATCCGTTGGGTCTTCCAATATCAATAGGTT

GATTGTTTCGCTCCTGTATCTTCCAAAAGGAAAAAAGATCTCTGAGAGTTGTTTCCTGAA

TCCGAAAGAGAGTACTTGGGTTCTCCCAATAACTAAAAAGTGTAGCATGCCTGAATCTAA

CTGGGGTTCGCGGTGGTGGAGGAACTGGATCGGAAAAAAGAGGGATTCTAGTTGTAAGAT

ATCTAATGAAACCGTCGCTGGAATTGAGATCTTATTCAAAGAGAAAGATATCAAATATCT

GGAGTTTCTTTTTGTATATTATATGGATGATCCGATCCGCAAGGACCATGATTGGGAATT

GTTTGATCGTCTTTCTCTGAGGAAGAGGCGAAATAGAATCAACTTGAATTCGGGACCGCT

ATTCGAAATCTTAGTGAAACACTGGATTTCTTATCTCATGTCTGCTTTTCGTGAAAAAAT

ACCAATTGAAGTGGAGGGTTTCTTCAAACAACAAGGGGCTGGGTCAACTATTCAATCAAA

TGATATTGAGCATGTTTCCCATCTCTTCTCGAGAAACAAGTGGGCTATTTCTTTGCAAAA

TTGTGCTCAATTTCATATGTGGCAATTCCGCCAAGATCTCTTCCTTAGTTGGGGGAAGAA

TCCGCACGAATCGGATTTTTTGAGGAACGTATCGAGAGAGAATTTGATTTGGTTAGACAA

TGTGTGGTTGGTAAACAAGGATCGGTTTTTTAGAAAGGTACGGAATGTATCGTCAAATAT

TCAATATGATTCCACAAGATCTAGTTTCGTTCAAGTAACGGATTCTAGCCAACTGAAAGG

ATCTTCTGATCAATCCAGAGATCATTTGGATTCCATTAGTAATGAGGATTCGGAATATCA

CACATTGATCAATCAAAGAGAGATTCAACAACTAAAAGAAAGATCGATTCTTTGGGATCC

TTCCTTTCTTCAAACGGAAGGAACAGAGATAGAATCAGACCGATTCCCGAAATGCCTTTC

TGGATATTCCTCAATGTCCCGGCTATTCACGGAACGTGAGAAGCAGATGATTAATCATCT

GCTTCCGGAAGAAATCGAAGAATTTCTTGGGAATCCTACAAGATCCGTTCGTTCTTTTTT

CTCTGATAGATGGTCAGAACTTCATCTGGGTTCGAATCCTACTGAGAGGTCCACTAGAGA

TCAGAAATTGTTGAAGAAACAACAAGATCTTTCTTTTGTCCCTTCCAGGCGATCGGAAAA

TAAAGAAATGGTTAATATATTCAAGATAATTACGTATTTACAAAATACTGTCTCAATTCA

TCCTATTTCATCAGATCCGGGGTGTGATAGGGTTCCGAAGGATGAACCGGATATGGACAG

TTCCAATAAGATTTCATTCTTGAACAAAAATCCATTTTTTGATTTATTTCATCTATTCCA

TGACCGGAACAGGCGAGGATACACGTTACACCACGATTTTGAATCAGAAGAGAGATTTCA

AGAAATGGCAGATCTATTCACTCTATCAATAACCGAGCCGGATCTGGTGTATCATAAGGG

ATTTGCCTTTTCTATTGATTCCTACGGATTGGATCAAAAACAATTCTTGAATGAGGCCAG

GGATGAATCGAAAAAGAAATCTTTATTGGTTCTACCTCCTATTTTTTATGAAGAGAATGA

ATCTTTTTCTCGAAGGATCAGAAAAAAATGGGTCCGGATCTCCTGCGGGAATGATTTGGA

AGATCCAAAACCAAAAATAGTGGTATTTGCTAGCAACAACATAATGGAGGCAGTCAATCA

ATATAGATTGATCCGAAATCTGATTCAAATCCAATATAGTACCTATGGGTACATAAGAAA

TGTATTGCATCGATTCTTTTTAATGAATCGATCCGATCGCAACTTCGAATATGGAATTCA

AAGGGATCAAATAGGAAAGGATACTCTGAATCATAGAACTATAATGAAATATACGATCAA

CCAACATTTATCGAATTTGAAAAAGAGTCAGTTCGATCCTCTTATCTTGATTTCTCGAAC

CGAGAAATCCATGAATCGGGATCCTGATGCATATAGATACAAATGGTCCAATGGGAGCAA

GAATTTCCAGGAACATTTGGAACATTTCGTTTCTGAGCAGAAGAGCCGTTTGCAAGTAGT

GTTCGATCAATTACGTATTAATCAATATTCGATTGATTGGTCTGAGGTTATCGACAAAAA

AGATTTGTCTAAGCCACTTCGTTTCTTTTTGTCCAAGTCACTTCTTTTTTTGTCCAAGTT

GCTTTTCTTTTTGTCTAACTCACTTCCTTTTTTCTGTGTGAGTTTCGGGAATATCCCCAT

TCATAGGTCCGAGATCTACATCTATGAATTGAAAGGTCCGAATGATCAACTCTGCAATCA

GTTGTTAGAATCAATAGGTCTTCAAATTGTTCATTTGAAAAAATGGAAACCCTTCTTATT

GGATGATCATGATACTTCCCGAAAATCGAAATTCTTGATCAATGGAGGAACAATATCACC

CTTTTTGTTCAATAAGATCCCAAAGTGGATGATTGACTCATTCCATACTAGAAATAATCG

CAGGAAATCCTTTGATAACACGGATTCCTATTTCTCAATGATATCCCACAATCAAGACAA

TTGGCTGAATCCCGTGAAACCATTTCATAGAAGTTCATTGATATCTTCTTTTTATAAAGC

AAATCGACTTCGATTCTTGAATAATCCACATCACTTCTGCTTCTATTGTAACACAAGATT

CCCCTTTTCTGTGGAAAAGGCCCGTATCTATAATTATGATTTTACGTATGGACAATTCCT

CAATATCTTGTTCATTCGCAACAAAATATTTTCTTTGTGCGTCGGTAAAAAAAAACATGC

TTTTGGGGGGAGAGATACTATTTCACCAATCGAGTCACAGGTATCTAACATATTCATACC

TAACTATTTTCCACAAAGTGGTGACGAAACGTATAACTTGTACAAATCTTTCCATTTTCC

AAGTCGATACGATCCATTCGTTCGTAGAACTATTTACTCGATCGCAGACATTTCTGGAAC

ACCTCTAACAGAGGGACAAATAGTCAATTTTGAAAGAACTTATTGTCAACCTCTTTCAGA

TATGAATCTATCTGATTCAGAAGGGAAGAACTTGCATCAGTATCTCAATTTCAATTCAAA

CATGGGTTTGATTCACACTCCATGTTCTGAGAAATATTTACCATCCGAAAAGAGGAAAAA

ACGGAGTCTTTGTCTAAAGAAATGCGTTGAGAAAGGGCAGATGTATAGAAGAGATAGTGC

TTTTTCAACTCTCTCAAAATGGAATCTATTCCAAACATATATGCCATGGTTCCTTACTTC

GACAGGGTACAAATATCTAAATTTGATATTTTTAGATACTTTTTCAGACCTATTGCCGAT

ACTAAGTAGCAGTCAAAAATTTGTATCCATTTTTCATGATATTATGCATGGATCAGGTAT

ATCATGGCGAATTCTTCAGAAAAAATTGTGTCTTCCACAATGGAATCTGATAAGTGAGAT

TTCGAGTAAGTGTTTACATAGTCTTCTTCTGTCCGAAGAAATGATTCATCGAAATAATGA

GTCACCATTGATATCGACACATCTGAGATCGCCAAATGTTCGGGAGTTCCTCTATTCAAT

CCTTTTCCTTCTTCTTGTTGCTGGATATCTCGTTCGTACACATCTTCTCTTTGTTTCCCG

GGCCTCTAGTGAGTTACAGACAGAGTTCAAAAAGGTCAAATCTTTGATGATTCCATCATC

TATGATTGAGTTGCGAAAACTTCTGGATAGGTATCCTACATCTGAACCGAATTCTTTCTG

GTTAAAGAATCTCTTTCTAGTTGCTCTGGAACAATTAGGAGATTCTCTAGAAGAAATACG

GGGTTCTGCTTCTGGCGGCAACATGCTTGGTCCCGCTTATGGGGTCAAATCAATACGTTC

TAAGAAGAAATATTTGAATATCAATCTCATCGATCTCATACCAAATCCCATCAATCGAAT

CACTGTTTCGAGAAATACGAGACATCTAAGTCATACAAGTAAAGAGATCTATTCATTGAT

AAGAAAAAGAAAAAACGTGAACGGGGATTGGATTGATGATAAAATAGAATCCTGGGTCGC

GAACAGTGATTCGATTGATGATGAAGAAAGAGAATTCTTGGTTCAGTTCTCCGCCTTAAC

GACAGAAAAAAGGATTGATCAAATTCTATTGAGTCTGACTCATAGTGATCATTTATCAAA

GAATGACTCTGGTTATCAAATGATTGAACAACCGGGAGCAATTTACTTACGATACTTAGT

TGACATTCATAAAAAGTATCTATTGAATTATGAGTTCAATACATCCTGTTTAGCAGAAAG

ACGGGTATTCCTTGCTCATTATCAGACAATCACTTATTCACAAACTTCGTGTGGGACTAA

TACTTTTCATTTCCCATCTCATGGAAAACCCTTTTCGCTCCGCTTAGCCTTATCCCCCTC

TAGGGGGATTTTAGTGATAGGTTCTATAGGAACTGGACGATCCTATTTGGTCAAATACCT

AGCGACAAACTCCTATGTTCCTTTCATTACGGTATTTCTGAACAAGTTCCTGGATAACAA

GCCTAAAGGTTTTCTTATTGATGATATCAATATTGATGATAGTGACGATATTGATGATAG

TGACAATATTGATGCTAGTGACGATATTGATGCTAGTGACGATATCGATCGTGACCTTGA

TACGGAGCTGGAACTGCTAACTATGATGAATGCGCTAACTATGGATATGATGCCGGAAAT

AGACCGATTTTATATCACCCTTCAATTCGAATTAGCAAAAGCAATGTCTCCTTGCATAAT

ATGGATTCCAAACATTCATGATCTGGATGTGAATGAGTCGAATTACTTATCCCTCGGTCT

ATTAGTGAACCATCTCTCTGAAAGATGTTCCACTAGAAATATTCTTGTTATTGCTTCGAC

TCATATTCCCAAAAAAGTGGATCCCGCTCTAATAGCTCCGAATAAATTAAATACGTGCAT

TAAGATACGAAGGCTTCTTATTCCACAACAACAAAAGCACTTTTTCACTCTTTCATATAC

TAGGGGATTTCACTTGGAAAAGAAAATGTTCCATACTAATGGATTCGGGTCCATAACCAT

GGGTTCCAATGCACGAGATCTTGTAGCACTTACCAATGAGGCCCTATCGATTAGTATTAC

ACAGAAGAAATCAATTATAGACACTAATACAATTAGATCCGCTCTTCATAGACAAACTTG

GGATTTGCGATCCCAGGTAAGATCGGTTCAGGATCATGGGATCCTTTTCTATCAGATAGG

AAGGGCTGTAGCACAAAATGTACTTCTAAGTAATTGCCCCATAGATCCTATATCTATCTA

TATGAAGAAGAGATCATGTAACGAAGGGGATTCTTATTTGTCCAAATGGTACTTCGAACT

TGGAACGAGCATGAAGAAATTAACGATACTTCTTTATCTTTTGAGTTGTTCTGTCGGATC

GGTCGCTCAAGATCTTTGGTCTCTACCCGGACCCGATGAAAAAAATGGGATCACTTCTTA

TGGACTCGTTGAGAATGATTCTGATCTAGTTCATGGCCTATTAGAAGTAGAAGGCGCTTT

GGTGGGATCTTCACGGACAGAAAAAGATTGCAGTCAGTTTGATAATGATCGAGTGACATT

GCTTCTTCGGCCCGAACCGAGGAATCCCTTAGATATGATGCAAAACGGATTTTGTTCTAT

CTTTGATCAGAGATTTCTCTATGAAAAATACGAATCGGAGTTTGAAGAAGGGGAGGGAGA

AGGAGCCCTTGACCCGCAACAGATAGAGGAGGATTTATTCAATCACATAGTTTGGGCTCC

TAGAATATGGCGCCCTTGGGGCTTTCTATTTGATTGTATCGAAAGGCCCAATGAATTGGG

ATTTCCCTATTGGTCCAGGTCATTTCGGGGCAAGCGGATCATTTATGATGAAGAGGATGA

GCTTCAAGAGAATGATTCGGAGTTCTTGCAGAGTGGAACCATGCAGTACCAGACACGAGA

TAGATCTTCCAAAGAACAAGGCCTTTTTCGAATAAGCCAATTCATTTGGGACCCTGCAGA

TCCGCTCTTTTTCCTATTCAAAGATCAGCCCCCTGGCTCTGTGTTTTCACATCGAGAATT

ATTTGCAGATGAAGAGATGTCAAAGGGGCTTCTTACTTCCCAAACAGATCCTCCTACATC

TATATATAAACGCTGGTTTATCAAGAATACACAAGAAAAGCACTTCGAATTGTTGATTAA

TCGTCAGAGATGGCTTAGAACCAATAGTTCATTATCTAATGGATCTTTCCGTTCTAATAC

TCTATCCGAGAGTTATCAGTATTTATCAAATCTGTTCCTATCTAACGGAACGCTATTGGA

TCAAATGACAAAGACATTGTTGAGAAAAAGATGGCTTTTCCCGGATGAAATGAAAATTGG

ATTCATGTAACAGGAGAAAGATTTCCCATTCCTTAGCCGGAAAGATATGTGGCCATGAAA

GAGAAAGAGGGATTAAGTAGAACAGAATTGACTGGGTGGTAGAGTCGTGGAAACGCTTCT

TTCTTCCATATTTTGGACCTTAGCTCCATGGAACAATATGTTACTGCTGAAACACGGAAG

AATTGAAATCTTAGATCAAAACACTATGTATGGATGGTATGAACTGCCCAAACAAGAATT

CTTGAACAGCGAACAACCAGTTCAGATATTCACGACCAAGAAGTACTGGATTCTCTTTCG

GATAGGCCCTGAAAGGAGAAGGAAGGCTGGAATGCCAACAGGCGTCTATTATTGAATTCA

CCCGACCCGATAGTACCCATTTTGGGAACGTCCAGTGCCAAAGTCACTGAATGGGTAAGT

CGCCAATCCCTGGACTATGTAATCTGCTGGGTTACGGGCGGGCATTTTACCAGAGGTTTC

TAATCTACCCTTGTGTGATTCCTGTTGAAGCATATACTCGGGGGTGGGTGCAGGGCGGAC

GATTTTAAAGCAGACTCCCCATTCATTAGATAGAGAAGATCACCAAGATTTCGTGATTCG

CTGCCGAACTTATTCCAATTCCAAGAGCTCGGATCGAATCGGTATATACCGATTCGATCC

GAGCTCTCTTATTGAATTGCTCATTCAATGAGCATTCTCAATATTATGCCTTGAAGAGGA

CTCGAACCTCCACGCTCTTTAGCACGAGATTTTGAGTCTCGCGTGTCTACCATTTCACCA

CCAAGGCATCTTGAAAGTGAATCCTATTCCATGAATATGATATCTATCTAGTGTGATGTA

TGGAATATATGACAAGGGTGGAGTATTTCTATTGATCGGCCCGAGTTGGACATCCAATTG

CTTCGATTTGAATTATCCGGAGAATGCCTTATGTATATATCAAAAAGATGGCCAATCAAA

CCTATTTCTCGATTCAATAGAAGCTCAAAGAGGTGATATAGGGTCCCAAATAACGAGAGA

TATGTAAAAAGCAGGTCCGATTATTACGCCTATTCCTAATCCTAAATGGAATGGAACGAC

GTAGGGATCCATATGTAAACATAGTATCTATTTAGATACGCTCGAATGACCCCTTGAGAA

TGTATATAACCCTATTCCGGTCTGGTCCGGTATGGAATGAACTTATAATCATGGAATCGA

CTCGATCATCAGATTATAAGTTCATAACCCTAGCCCATTCCCATTTTGGTCGGAACAGAT

CTACTAATTCTTTGATTCCAGTTAGTAAGAGGGATCTTGAACTAAGAAATAGACCCTAGA

AGCTAAAAAAGGGTATCCTGAGCAATTGCAATAATCGGGTTCATTGATATTCCAGGTATA

GTAGATGCTATCACACATACAATCATACTCAATTCGATGGAATTGTTTGATCTTAAAGGA

GATCTTCTATAATTTCGCACGTGAGGGGTGATTTCTTGGTTTCGTCCAGTCATTAATAAC

TTGATTATTTTTAGATAATAGTAGATAGAAACAACGCTTGTAAGGAGTCCTATTAAAACC

AAGGAATATAGGCCTGCCTGCCATCCACACCAGAATAAATAGAGTTTTCCGAAAAAACCT

GCTAGTGGAGGAAGACCTCCTAGGGATAAGAGACATAGGGCTAAAGAGAGAGCCAAAAAA

GGATCTTTCGTGTATAATCCTGCATAATCTCGAATGTTATCAGTTCCGGTACGTAGACCA

AATAATACAATGCAAGCAAAAGTTCCTAGATTCATGGAGATATAGAACAGCATATAAGTT

ATCATGCTTGCATATCCATCATTTGAGTCTCCAACAATTATTCCAATAATTACATATCCG

ATTTGACCTATGGACGAATATGCAAGCATACGTTTCATGCTTGTTTGAGTAATAGCAATG

ATATTTCCCAATATCATGCTAAGAATAGCTAGGATTTCCAGAAGAAGATGCCATTCGTTT

GATGAGAAATAAAAAGGAATATCGAAAATTCGAGTGGCTGAAGCTGAAGCAGCTACTTTC

GAAGTAACAGAAAGAAAAGCAACGACTGGAGTGGGAGAGTCAGAGTCGAAAAGAGGATTC

CTCACTTCTTTCTCTCATTCAAAACCGTGCATGAGACTTTCATCTCACACGGCTCCTAAG

TGATAAAAGAAAGAAGAACTCATCTTCTTTCTTTTTTGATTACCTTCCTCGCGTATGTAT

AAGACCGAATCCATTCGATTTCTAAAAAAGATTACTAATCCTTAACTTTTCGAGGAATCC

TTCATCAGTGGTTGTGAATGACTGATTTTTTCAATCTTTTCGACCTTGGTTCCGTAGGAG

CAAGTCAGAAAGATTGAGAAATAGAACCATCTGATTTAATTCGTTCTCAATAGCCATGAA

ATGATCATCTTAGGGTGATCCTTTTGTCGACGGATGCTCCTATTACACTCGTAGTCTCTG

AAGGATGAGAACCAACTATGTAGCATCTACATCAAGAATTCAAGTATTGTATACGTCATT

AGTCCGATCCTTTGTAGGAACTACCCGTAATAACGAACTTGCAAAATGGATCTGTTTATC

ATAAAGAGATTCGTCGTTCCTGACCCTGCTTCACCTTAATTGTTATTTGAACAAGTAAAA

GTTCTGTCTTGGTCCGAGTGGGGATAGCATTTCTCTTCTGCATGTCCATGGAGTTTTGAA

AAATCCAAACATCTCAGAGATAGATAGAGAGGTAGGAATTTCTCGAACGAACCGCACTCC

TTCGTATACGTCAGGAGTCCATTGATGAGAAGGGGCTGGGGAAAGCTTGAACCCAATTCC

TACAGTGATGAATATGAGCGCAATTGAAATTCCTGGGGAGTTATACATTTGTGTATTGAT

AAGACCATTCACTATTTCTTGAAGCTCGATCTCTCCCCCGGATGAACCATATAGCCAAGA

GAAACCATGAACCAGAATAGAAGAGCTTGCCCCACCCATGAGTAAATATTTCATAGTAGC

CTCATTAGACCGTACATCTTTCTTGGTATATCCAGATAATAGGTAGGAGCATAAACTGAA

ACATTCTGGAGCTACAAAGATAGTTATTAAATCGTTAGCACCGCATAAAAACATTCCTCC

TAGAGTAGCTGTTAATACGAATAAGAGAAACTCTGTTATAGCCATTTCTGTACATTCAAT

GTACTCTACGGATAGAGGAATACATAGAGTTGAACATAGTAAAATAAGAAATTGAAAGAT

TTCGTTGAAATTGTTCGTTTGGAAATTTCCCGAAAAGCTAATCATAGGTTCTTCTCTCCA

TCGGAACAATAGGGCCGTTATGCTCATTACTAAACTTGTTGAAGAGATGAAATATAACCA

AGGTATATCTTTTTGATCAGAGGTTGAATCGATCATCAGAAGAAGAATTAGGCCAAAAAT

TAGGATACATTCTGGGAAAATCAAACTTCCATCGAAGAGAAGCAAATGAAAGGCTTTCAT

AAAAATTCTCGTAGAATCGAGAATGAAGTTTTCATTCTGTACATGCCAGATCATGAATTA

GTAACTGCATCCAATTTCAAAAAAAAATCCCAATTGTGTCGAACTTTCCATTTTTGGAAT

GGAATAGGATCAAGATCAAACCTTATTCCATGGTATTTACATGAGGTTCCTCTTTAAGAA

AGTCCCCGAGAGGGCTTAGTTGATCCATGATTTATGTTTCATCTTTCGTTTCCTTTTCGT

TTGTTTCGAGAAATCTATCGATCAATTCCGATTCTTTCTTTTTCTCTTGATTCTTTTCCG

ATCGAGATGTATAGATCCTGTTCATGGATTAACGAAAATGTGCAAAAGCTCTATTTGCCT

CTGCCATTTTATGAGTCTCTTCCTTTTTGCGTATGGCATCGCCACTCCCTTTGGCAGCAT

CCACTAATTCGGAACTTAATTTGAAAGCCATATTTCGACCCGGACGTTTTCGGGATGCCG

CTAATAACCAACGAATGGCAAGTGCTTTTCCTTGTGTGGATCCTATTTCAATGGGAACTT

GATGAGTTGATCCACCTACACGTCTTGCTTTTACTGCTATATCGGGAGTTACTCCACGTA

TTGCTTGACGTAAAACAGATAGTGGATTTGTTTCTGTCTTTTGTTGAATCTTTTTCATGG

CTCGATAGATAATTTGATAAGCCAATGATTTTTTTCCGTGTTTCAGAATACGGTTAACCA

ACATGTTAACTAATCGATTACGATAAATTGGATCGGATTTTGCTGTTTTTTCTTCTGCAG

TACCTCGACGTGACATGAGCGTGAAAGGGGTTCAAGAATCCGTTTTCTTTTTATAAGGGC

TAAAATCACTTA-TTTTTTTTTTGCTTTTTTACCCCATATTGTAGGGTGGATCTCGAAAG

ATATGAAAGATCTCCCTCCAAGCCGTACATACGACTTTCATCGAATACGGCTTTCCGCAG

AATTCTATATGTATCTTTGAGATCGAGTATGGAATTCTGTTTACTCACTTTAAATTGAGT

ATCCGTTTCCCTCCCTTTCCTGCTAGGATTGGAAATCCTGTATTTTACATATCCATACGA

TTGAGTCCTTGGGTTTCCGAAATAGTGTAAAAAGAAGTGCTTCGAATCATTGCTATTTGA

CTCGGACCTGTTCTAAAAAAGTCGAGGTATTTCGAATTGTTTGTTGACACGGACAAAGTC

AGGGAAAACCTCTGAAATTATTTCCATATTGAACCTTGGACATATAAGAGTTCCGAATCG

AATCTCTTTAGAAAGAAGATCTTTTGTCTCATGGTAGCCTGCTCCAGTCCCCTTACGAAA

CTTTCGTTATTGGGTTAGCCATACACTTCACATGTTTCTAGCGATTCACATGGCATCATC

AAATGATACAAGTCTTGGATAAGAATCTACAACGCACTAGAACGCCCTTGTTGACGATCC

TTTACTCCGACAGCATCTAGGGTTCCTCGAACAATGTGATATCTTACACCGGGTAAATCC

TTAACCCTTCCCCCTCTTACTAAGACTGAAGAATGTTCTTGTGAATTATGGCCAATACCG

GGTATATAAGCAGTGATTTCAAATCCAGAGGTTAATCGTACTCTGGCAACTTTACGTAAG

GCAGAGTTTGGTTTTTTTGGGGTGATAGTGGAAAAGTTGACAGATAAGTCACCCTTACTG

CCACTCTACAGAACCGTACATGAGATTTTCACCTCATACGGCTCCTCGTTCAATTCTTTC

GAATTCATTGGATCCTTTTCCGCGTTCGAGAATCCCCTCCCTTCTTCCACTCTGTCCCGA

AGAGTAACTAGGACCATTTAGTCACGTTTTCATGTTCCAATTGAACACTTTCCATTTTTG

ATTATTTTCAAAGGAGAAGATTATTCTCTTTACCAAACATATGCGGATCCAATCACGATC

CTATAATAAGAACAAGAGATCTTTCTCGATCAATCCCTTTGCCCCTCATTCTTCGAGAAT

CAGAAAGATCCTTTTCAAGTTTGAATTTGTTCATTTGGAATCTGGGTTCTTCTACTTCAT

TTTTATTTAATATTTTTCCCTCTCTTTTTTTTTATATCATTCCTTAAGTCCCATAGGTTT

GATCCTGTAGAATTTGACCCATTTTCTCATTGAACGAAGGGTACGAAATCAATCAGATTG

ATTTTTCGATCAAAAGTACTATGTGAAATCTTCGGTTTTTTCCTCTTCCTCTATCCCTAT

CCCATAGGTACAGTGTTTGAATCAATAGAGAACCTTTTCTTCTGTATGAATCGATCTTAT

TCCATTCCAATTCCTTCCCGATACCTCCCAAGGAAAATATCGAATGGATCCCAAATTGAC

GGGTTAGTGTGAGCTTATCCATGCGGTTATGCACTCTTCGAATAGGAATCCGTTTTCTGA

AAGATCCTGGCTTTCGTACTTTGGTGGGTCTCCGAGATCCTTTCGATGACCTATGTTGTG

TTGAAGGGATATCTATCTAATCCGATCGATTGCGTAAAGCCCGCGATAGCAACGGAACCG

GGGAAAGTATACAGAAAAGATAGTTCTTTTCTATTATATTAGTATTTTCTATTATATTAG

ATTAGTATTAGTTAGTGATCCCGGCTTAGTGAGTCCTTTCTTCCGTGATGAACTGTTGGC

ACCAGTCCTACATTTTGTCTCTGTGGACCGAGGAGAAAAGGGGCTCGGCGTGTACATGAG

AGAAGCAAGGAGGTCAACCTCTTTCAAATATACAACATGGATTCTGGCAATGCAATGTAG

TTGGACTCTCATGTCGATCCGAATGAATCATCCTTTCCGCGGAGGTCAATCTTTGCCTGC

TAGGCAAGAGGAGAGCAAGTTACAAATTCCGTCTCGGTAGGACATGTATTTCTATTACTA

TGAAATTCATAAATGAAGTAGTTCATGGTGGGGTTACCATTATCCTTCTTGTAGTGACGA

ATCTTGTATGTGTTCTTAAGAAAAGGAATTTGTCCATTTTTCGGGGTCTCAAAGGGCGTG

GAAACACATAAGAACTCTTGAATGGAAATGGAAAAGAGATGTAACTCCAGTTCCTTCGGA

ATCGCTAGTCAATCCTATTTCCGATAGGGGCAGTTGACAATTTAATCCGATTTTGACCAT

TATTTTCATATCCGTAATAGTGCGAAAAGAAGGCCCGGCTCCAAGTTGTTCAAGAATAGT

GGCGTTGAGTTTCTCGACCCTTTGACTTAGGATTAGTCAGTTCTATTTCTCGATGGGGGC

AGGGAAGGGATATAACTCAGCGGTAGAGTGTCACCTTGACGTGGTGGAAGTCATCAGTTC

GAGCCTGATTATCCCTAAACCCAATGTGAGTTCTTCTATTTGGATTTGCTCCCCCGCCGT

GATTCAATGAGAATGGATAAGAGGCTCGTGGGATTGACGTGAGGGGGTAGGGATGGCTAT

ATTTCTGGGAGCGAACTCCGGGCGAATATGAAGCGCATGGATACAAGTTATGCCTTGGAG

TGAAAGACAATTCCGAATCCGCTTTGTCTACGAACAAGGAAGCTATAAGTAATGCAACTA

TGAATCTCATGGAGAGTTCGATCCTGGCTCAGGATGAACGCTGGCGGCATGCTTAACACA

TGCAAGTCGGACGGGAAGTGGTGTTTCCAGTGGCGGACGGGTGAGTAACGCGTAAGAACC

TGCCCTTGGGAGGGGAACAACAGCTGGAAACGGCTGCTAATACCCCGTAGGCTGAGGAGC

AAAAGGAGGAATCCGCCCGAGGAGGGGCTTGCGTCTGATTAGCTAGTTGGTGAGGCAATA

GCTTACCAAGGCAATGATCAGTAGCTGGTCCGAGAGGATGATCAGCCACACTGGGACTGA

GACACGGCCCAGACTCCTACGGGAGGCAGCAGTGGGGAATTTTCCGCAATGGGCGAAAGC

CTGACGGAGCAATGCCGCGTGGAGGTAGAAGGCCCACGGGTCGTGAACTTCTTTTCCCGG

AGAAGAAGCAATGACGGTATCT-GGGGAATAAGCATCGGCTAACTCTGTGCCAGCAGCCG

CGGTAAGACAGAGGATGCAAGCGTTATCCGGAATGATTGGGCGTAAAGCGTCTGTAGGTG

GCTTTTTAAGTCCGCCGTCAAATCCCAGGGCTCAACCCTGGACAGGCGGTGGAAACTACC

AAGCTGGAGTACGGTAGGGGCAGAGGGAATTTCCGGTGGAGCGGTGAAATGCGTAGAGAT

CGGAAAGAACACCAACGGCGAAAGCACTCTGCTGGGCCGACACTGACACTGAGAGACGAA

AGCTAGGGGAGCGAATGGGATTAGATACCCCAGTAGTCCTAGCCGTAAACGATGGATACT

AGGCGCTGTGCGTATCGACCCGTGCAGTGCTGTAGCTAACGCGTTAAGTATCCCGCCTGG

GGAGTACGTTCGCAAGAATGAAACTCAAAGGAATTGACGGGGGCCCGCACAAGCGGTGGA

GCATGTGGTTTAATTCGATGCAAAGCGAAGAACCTTACCAGGGCTTGACATGCCGCGAAT

CCTCTTGAAAGAGAGGGGTGCCTTCGGGAACGCGGACACAGGTGGTGCATGGCTGTCGTC

AGCTCGTGCCGTAAGGTGTTGGGTTAAGTCCCGCAACGAGCGCAACCCTCGTGTTTAGTT

GCCATCGTTGAGTTTGGAACCCTGAACAGACTGCCGGTGATAAGCCGGAGGAAGGTGAGG

ATGACGTCAAGTCATCATGCCCCTTATGCCCTGGGCGACACACGTGCTACAATGGCCGGG

ACAAAGGGTCGCGATCCCGCGAGGGTGAGCTAACCCCAAAAACCCGTCCTCAGTTCGGAT

TGCAGGCTGCAACTCGCCTGCATGAAGCCGGAATCGCTAGTAATCGCCGGTCAGCCATAC

GGCGGTGAATTCGTTCCCGGGCCTTGTACACACCGCCCGTCACACTATGGGAGCTGGCCA

TGCCCGAAGTCGTTACCTTAACCGCAAGGAGGGGGATGCCGAAGGCAGGGCTAGTGACTG

GAGTGAAGTCGTAACAAGGTAGCCGTACTGGAAGGTGCGGCTGGATCACCTCCTTTTCAG

GGAGAGCTAATGCTTGTTGGGTATTTTGGTTTGACACTGCTTCACACCCAAAACAAAAAG

AAGGGAGCTACGTCTAAGTTAAACTTGGAGATGGAAGTCTTCTTTCGTTTCTCGACGTTG

AAGTAAGACCAAGCTCATGAGCTTATTATCCTAGGTCGGAACAAGTTGATAGGATCCCCT

TTTTTACGTCCCCATGTCCCCCCGTGTGGCGACATGGGGGCGAAAAAAGGAAAGAGAGGG

ATGGGGTTTCTCTCGCTTTTGGCATAGCGGGCCCCCAGTGGGAGGCTCGCACGACGGGCT

ATTAGCTCAGTGGTAGAGCGCGCCCCTGATAATTGCGTCGTTGTGCCTGGGCTGTGAGGG

CTCTCAGCCACATGGATAGTTCAATGTGCTCATCGGCGCCTGACCCTGAGATGTGGATCA

TCCAAGGCACATTAGCATGGCGTACTCCTCCTGTTCGAACCGGGGTTTGAAACCAAACCT

CTCCTCAGGAGGATAGATGGGGCGATTCGGGTGAGATCCAATGTAGATCCAACTTTCGAT

TCACTCGTGGGATCCGGGCGGTCCGGGGGGGACCACCACGGCTCCTCTCTTCTCGAGAAT

CCATACATCCCTTATCAGTGTATGGACAGCTATCTCTCGAGCACAGGTTTAGGTTCGGCC

TCAATGGGAAAAGAAAATGGAGCACCTAACAACGCATCTTCACAGACCAAGAACTACGAG

ATCACCCCTTTCATTCTGGGGTGACGGAGGGATCGTACCATTCGAGCCGTTTTTTTTTCA

TGCTTTTCCCGGAGGTCTGGAGAAAGCTGCAATCAAGAGGATTTCCCTAATCCTCCCTTC

CCGAAAGGAAGAGCGTGAAATTCTTTTTCCTTTCCGCAGGGACCAGGAGATTGGATCTAG

CCGTAAGAAGAATGCTTGGTATAAATAACTCACTTCTTGGTCTTCGACCCCCTCAGTCAC

TACGAACGCCCCCGATCAGTGCAATGGGATGTGTCTATTTATCTATCTCTTGACTCGAAA

TGGGAGCAGGTTTGAAAAAGGATCTTAGAGTGTCTAGGGTTGGGCCAGGAGGGTCTCTTA

ACGCCTTCTTTTTTCTTCTCATCGGAGTTATTTCACAAAGACTCGCAGGGTAAGGAAGAA

GGGGGGAACAAGCACACTTGGAGAGCGCAGTACAACGGAGAGTTGTATGCTGCGTTCGGG

AAGGATGAATCGCTCCCGAAAAGGAATCTATTGATTCTCTCCCAATTGGTTGGACCGTAG

GTGCGATGATTTACTTCACGGGCGAGGTCTCTGGTTCAAGTCCAGGATGGCCCAGCTGCG

CCAGGGAAAAGAATAGAAGAAGGATCTGACTACTTCATGCATGCTCCACTTGGCTCGGGG

GGATATAGCTCAGTTGGTAGAGCTCCGCTCTTGCAATTGGGTCGTTGCGATTACGGGTTG

GATGTCTAATTGTCCAGGCGGTAATGATAGTATCTTGTACCTGAACCGGTGGCTCACTTT

TTCTAAGTAATGGGGAAGAGGACCGAAACATGCCACTGAAAGACTCTACTGAGACAAAGA

TGGGCTGTCAAGAACGTAGAGGAGGTAGGATGGGCAGTTGGTCAGATCTAGTATGGATCA

TACATGGACGGTAGTTGGAGTCGGCGGCTCTCCCAGGGGTCCCTCATCTGAGATCCCTGG

GGAAGAGGATCAAGTTGGCCCTTGCGAACAGCTTGATGCACTATCTCCCTTCAACCCTTT

GAGCGAAATGCGGCAAAAGAAAAGGAAGGAAAATCCATGGACCGACCCCATCATCTCCAC

CCCGTAGGAACTACGAGATCACCCCAAGGGCGCCTTTGGCATCCAGGGGTCACGGACCGA

CCATAGAACCCTGTTCAATAAGTGGAACGCATTAGCTGTCTGTTCTCAGGTTGGGCAGTA

AGGGTCGGAGAAGGGCAATCACTCATTCTTAAAACCAGCGTTCTTAAGACCAAAGAGTCG

GGCGGAAAGGGGGGAAAGCTCTCCGTTCCTGGTTCTCCTGTAGCTGGAACCTCCGGAACC

ACAAGAATCCTTAGTTAGAATGGGATTCCAACTCAGCACCTTTTGAGTGAGATTTTGAGA

AGAGTTGCTCTTTGGAGAGCACAGTACGATGAAAGTTGTAAGCTGTGTTCGGGGGGGAGT

TATTGTCTATCGTCGGCCTCTATGGTAGAATCAGTCGGGGGGCCTGAGAGGCGGTGGTTT

ACCCTGCGGCGGATGTCAGCGGTTCGAGTCCGCTTATCTCCAACTCATGAACTTAGCCGA

TACAAAGCTATATGATAGCACCCAATTTTTCCGATTCGGCGGTTCGATCTATGATTTATC

ATTCATGGACGTTGATAAGATCCATCCATTTAGCAGCACCTTAGGATGGCATAGCCTTAG

AAGGGCGAGGTTCAAACGAGGAAAGGCTTACGGTGGATACCTAGGCACCCAGAGACGAGG

AAGGGCGTAGTAATCGACGAAATGCTTCGGGGAGTTGAAAATAAGCATAGATCCGGAGAT

TCCCGAATAGGGCAACCTTTCGAACTGCTGCTGAATCCATGGGCAGGCAAGAGACAACCT

GGCGAACTGAAACATCTTAGTAGCCAGAGGAAAAGAAAGCAAAAGCGATTCCCGTAGTAG

CGGCGAGCGAAATGGGAGCAGCCTAAACCGTGAAAACGGGGTTGTGGGAGAGCAATACAA

GCGTCGTGCTGCTAGGCGAAGCAGCACGAATGCTGCACCCTAGATGGCGAAAGTCCAGTA

GCCGAAAGCATCACTAGCTTACGCTCTGACCCGAGTAGCATGGGACACGTGGAATCCCGT

GTGAATCAGCAAGGACCACCTTGCAAGGCTAAATACTCCTGGGTGACCGATAGCGAAGTA

GTACCGTGAGGGAAGGGTGAAAAGAACCCCCATCGGGGAGTGAAATAGAATATGAAACCG

TAAGCTCCCAAGCAGTGGGAGGAGCCAGGTCTCTGACCGCGTGCCTGTTGAAGAATGAGC

CGGCGACTCATAGGCAGTGGCTTGGTTAAGGGAACCCACCGGAGCCGTAGCGAAAGCGAG

TCTTCATAGGGCAATTGTCACTGCTTATGGACCCGAACCTGGGTGATCTATCCATGACCA

GGATGAAGCTTGGGTGAAACTAAGTGGAGGTCCGAACCGACTGATGTTGAAGAATCAGCG

GATGAGTTGTGGTTAGGGGTGAAATGCCACTCGAACCCAGAGCTAGCTGGTTCTCCCCGA

AATGCGTTGAGGCGCAGCAGTTGACTGGACATCTAGGGGTAAAGCACTGTTTCGGTGCGG

GCCGCGAGAGCGGTACCAAATCGAGGCAAACTCTGAATACTAGATATGACCTCAAAATAA

CAGGGGTCAAGGTCGGCCAGTGAGACGATGGGGGATAAGCTTCATCGTCGAGAGGGAAAC

AGCCCAGATCACCAGCTAAGGCCCCTAAATGATCGCTCAGTGATAAAGGAGGTAGGGGTG

CAGAGACAGCCAGGAGGTTTGCCTAGAAGCAGCCACCCTTGAAAGAGTGCGTAATAGCTC

ACTGATCGAGCGCTCTTGCGCCGAAGATGAACGGGGCTAAGCGATCTGCCGAAGCTGTGG

GATGTAAAAATACATCGGTAGGGGAGCGTTCCGCCTTAGAAGGAAGCCCCCGCGCGAGCA

GTGGTGGACGAAGCGGAAGCGAGAATGTCGGCTTGAGTAACGCAAACATTGGTGAGAATC

CAATGCCCCGAAAACCTAAGGGTTCCTCCGCAAGGTTCGTCCACGGAGGGTGAGTCAGGG

CCTAAGATCAGGCCGAAAGGCGTAGTCGATGGACAACAGGTGAATATTCCTGTACTACCC

CTTGTTGGTCCCGAGGGACGGAGGAGGCTAGGTTAGCCGAAAGCTGGTTATCGGTTCAAG

AACGTAAGGTGCCCCTGCTTTTTCAGGGTAAGAAGGGGTAGAGAAAATGCCTCGAGCCAA

TGTTCGAGCACCAGGCGCTACGGCGCTGAAGTAACCCACGCCATACTCCCAGGAAAAGCT

CGAACGACCTTTAAACAAAAGGGTACCTGTACCCGAAACCGACACAGGTGGGTAGGTAGA

GAATACCTAGGGGCGCGAGACAACTCTCTCTAAGGAACTCGGCAAAATAGCCCCGTAACT

TCGGGAGAAGGGGTGCCTCCTCACAAAGGGGGTCGCAGTGACCAGGCCCGGGCGACTGTT

TACCAAAAACACAGGTCTCCGCAAAGTCGTAAGACCATGTATGGGGGCTGACGCCTGCCC

AGTGCCGGAAGGTCAAGGAAGTTGGTGACCTGATGACAGGGGAGCCGGCGACCGAAGCCC

CGGTGAACGGCGGCCGTAACTATAACGGTCCTAAGGTAGCGAAATTCCTTGTCGGGTAAG

TTCCGACCCGCACGAAAGGCGTAACGATCTGGGCACTGTCTCGGAGAGAGGCTCGGTGAA

ATAGACATGTCTGTGAAGATGCGGACTACCTGCACCTGGACAGAAAGACCCTATGAAGCT

TCACTGTTCCCTGGGATTGGGTTTGGGCCTTTCCTGCGCAGCTTAGGTGGAAGGCGAAGA

AGGCCTCCTTCCGGGGGGGCCCGAGCCATCAGTGAGATACCACTCTGGAAGAGCTAGAAT

TCTAACCTTGTGTCAGGACCTACGGGCCAAGGGACAGTCTCAGGTAGACAGTTTCTATGG

GGCGTAGGCCTCCCAAAAGGTAACGGAGGCGTGCAAAGGTTTCCTCGGGCCGGACGGAGA

TTGGCCCTCGAGTGCAAAGGCAGAAGGGAGCTTGACTGCAAGACCCACCCGTCGAGCAGG

GACGAAAGTCGGCCTTAGTGATCCGACGGTGCCGAGTGGAAGGGCCGTCGCTCAACGGAT

AAAAGTTACTCTAGGGATAACAGGCTGATCTTCCCCAAGAGCTCACATCGACGGGAAGGT

TTGGCACCTCGATGTCGGCTCTTCGCCACCTGGGGCTGTAGTATGTTCCAAGGGTTGGGC

TGTTCGCCCATTAAAGCGGTACGTGAGCTGGGTTCAGAACGTCGTGAGACAGTTCGGTCC

ATATCTGGTGTGGGCGTTAGAGCATTGAGAGGACCTTTCCCTAGTACGAGAGGACCGGGA

AGGACGCACCTCTGGTGTACCAGTTATCGTGCCCACGGTAAACGCTGGGTAGCCAAGTGC

GGAGCGGATAACTGCTGAAAGCATCTAAGTAGTAAGCCCACCCCAAGATGAGTGCTCTCC

TATTCCGACTTCCCCAGAGCTTCCGGTAGCACAGCCGAGACAGCGACGGGTTCTCTGCCC

CTGCGGGGATGGAGCGACAGAAGTTTTGAGAATTCAAGAGAAGGTCACGGCGAGACGAGC

CGTTTATCATTACGATAGGTGTCAAGTGGAAGTGCAGTGATGTATGCAGCTGAGGCATCC

TAACAGACCGGTAGACTTGAACCTTGTTCCTACATGACCTGATCAATTCGATCAGGCACT

CGCCATCTATTTTCATTGTTCAAATCTTTGACAACACGAAAAACCATTGTTCAACTCTTT

GACAACATGAAAAAACCAAAAGCTCTGCCCTCCCTCTCTATCGGATGGAAGGGCAGAGGC

CTTTGGTGTCCCCTCCAGTCAAGAATTGGGGCCTCACAATCACTAGCCAATAGGCTTTTC

TCTCATGCCTTTCTTCGTTCATGGTTCGATATTCTGGTGTCCTAGGCGTAGAGGAACCAC

ACCAATCCATCCCGAACTTGGTGGTTAAACTCTACTGCGGTGACGATACTGTAGGGGAGG

TCCTGCGGAAAAATAGCTCGACGCCAGGATGATAAAAAGCTTAACACCTCTCATTCTTAT

TCCTTTTTCAACGAAAAAAATGAAAAATAAAAAGGTCGTCTTATTCAAAACCCCAATTAT

GACATCCCTTCTCTCCCACTTCACACCTCGGAACGCACCGTTCTTATAGAGATAAACGCG

CTTTCACATCTTCTTAACCCGAAATGAAATGGCTGGGGAGGGGAGAGGAAAGGTTCCTTT

TTTTTGAGGGTACTCCCGGGAACAGATCCAGTGGAGACGGGGTGGGGCCTGTAGCTCAGA

GGATTAGAGCACGTGGCTACGAACCACGGTGTCGGGGGTTCGAATCCCTCCTCGCCCACA

ACCGGCCCAAAAGGGAAGTACCTTTCCCTCTGGGGGTAGGAAAATCATGATCGGGATAGC

GGACCAAAAGCTATGGAACTTGGGTGTGGGTCTTTGGAATGGCTTTTTCTTTTTATTTAT

TATTTATCGTGAATGATGGAATCATTACAAATAGTATG-CCCCCGGCCCATCCGCGTATT

TTTTTGTTTTACGCCCCGTAACTCTTCCTCAGCCAGGCTTGGGCAGAATAGCAGAGCAAG

TACAAGTATTAGTAGCATAACAAAAATGCCTTCCTCGTCATTAATATGTTTGCTCGCGGC

AATTGTGAACTCTCGGGAGAATGATGCAGTGCTAGTACATCTGAGAATTCTTAATTGGCT

AGTTGTAAATAGCCCCAGGGCTATGGAACAAAGGATTATCCCGGACCTACACCGAGGTAT

TGACGGTGATTTTCAAATCTCGCAGAACAGAATGTGATACGATGAGATAGAAACAAAGAC

AGGGAACAGGTTACCTACTCTTAACGGTCAAAGCGAGCCCCTTTATTCTGAATTCGTTAA

TTCAGAATGAATCAAATCTCCCCAAGTAGGATTCGAACCTACGACCAATCGGTTAACAGC

CGACCGCTCTACCACTGAGCTACTGAGGAACAACGGGAGATTAGATCTCATAGAGTTCAA

TTCCCGTTCTCAACCCATGACCAATATGAGCTCGAAGCTTCCTTCGTAACTCCCGGAACT

TCTTCGTAGTGGCTCCCTTCCATGTCTCATTTCAGAGGGAACCTCAAAGTGGCTCTATTT

CATTATATTCCATCCATATCCCAATTCCATTCATTTAATACCCCTTTGGTGTCATTGACA

TAACAGATGTCGTTTCTAGTCTATCTCTTTCTATTTCTTTTCTATATATGGAAAGTTCAA

AAATCATCATATAATAATCCAGAAATTGCAATAGAAAAGAAATAAGGGAGGTTTGTGATG

ATTTTTCAATCTTTTCTACTAGGTAATCTAGTATCCTTATGCATGAAGATAATCAATTCG

GTCGTTGTGGTCGGACTCTATTATGGATTTCTGACCCCATTCTCCATAGGGCCCTCTTAT

CTCTTCCTTCTCCGAGCTCAGGTTATGGAAGAAGGAACCGAGAAGAAGGTATCAGCAACA

ACTGGTTTTATTACGGGACAGCTCATGATGTTCATATCGATCTATTATGCGCCTCTGCAT

CTAGCATTGGGTAGACCTCATACAATAACTGCCCTAGCTCTACCATATCTTTTGTTTCAT

TTCTTCTGGAACAATCACAAACACTTTTTTGATTATGGATCTAGAAATTCAATGCGTAAT

TTCAGCATTCAATGTGTATTCCTGAATAATCTCATTTTTCAATTATTCAACCATTTCATT

TTACCAAGTTCAATGTTAGCCAGATTAGTCAACATTTATATGTTTCGATGCAACAACAAG

ATGTTATTTGTAACAAGTAGTTTTGTTGGTTGGTTAATTGGTCACATTTTATTCATGAAA

TGGCTTGGATTGGTATTAGTCTGGATACGGCAAAATCATTCTATTAGATCGAATAAGTAC

ATTCGATCTAATAAGTACCTTGTGTCAGAATTGAGAAATTCTATGGCTCGGATCTTTAGT

ATTCTCTTATTTCTTACCTGTGTCTACTATTTAGGCAGAATACCGTCACCCATTCTTACT

AAGAAATGGAAAGAAACCTCAAAAATGGAAGAAAAGGTGGAAAGTGAGGAAGAAAGAGAT

GTAGAAATAGAAACAGCTTCCGAAATGAAGGGGACTAAACAGGAACAAGAGAGATCCGCC

GAAGAAGATCCTTCTCCTTCCTTTTTTTCGGAAGAAAAGGAGGATCCGAACAAAATCGAT

GAAACGGAAGAAATCCGAGTGAATGGAAAGGAAAAAACACAGGATGAATTCCACTTTCGA

TTTACAGAGACAGGCTCTAAAAATAGCCCAGTTTATGAAGATTCTTATCTGATGAATATC

AATGAAAATCACGACAATTCCAAATTCAAAATACTTATGAAAATAAAGACCTAATAAAAA

TATTGATACACAAGATAAATAGAAGAAAAGATAAGAAGATATGCGTCCACCCCCTACATA

TTTGATACCTTCTCCTACAAAGAAACTCATAACACCAACCCCATTCGTAATTCCATCAAT

TACTTGGCGATCAAAAAAATTAGTTAATTGGGCTAATCCTCTTACCGCCCCAGTTAAGAA

TGTTGTATAAAAAGCATCTATATAAGCACGATTATATGACCAATTATATAGACCATTTAG

AATTTTGTCCCAAAGACTTCTCTTAGGACCTGTTTTAACAAATAAATTTATTAAGTCGAA

ATTTTTGAAAGAGGAATAAATGGGTTTATATAAAAAGGACGCTATAAATATTCCGAAATA

AGCTATACTAACTGAAAAAATTGCATCTTTCCAAAATTCATACCAATCAATCGAATCATT

CAACTTTTGATGTAAAAGGTTTATAGACGGAGTTAACCATTTGGTTAATATATCCAATTC

TTGATTGAAAGGAATTCCTAGAGATCCAACGAACAAAGTAAAGATGCCCAATACAAGTAA

AGGAAATAACATAGTATTGTCGGATTCATAAGGATAGGAATAATCCTTTTTATTCTCAAA

ATGAACAATAGTAATAAAAGGTTCTACCCTGTTTCTTACATTTTTATCACTTCGATATGT

CTTTTTTGAAAAAAAAGAAGAACTTTTACTATTATTCATTTTTAATAAACGAAAATTTTT

GTTAATTCTTTTGGAACCCCCTTTTCCCCATAGAGATATTGAATAGAAAGGGGTATTTTG

TTTGCCACTGTAATTTTGAAAATGAACGTTTAAATGCCCTTCAAAAGTAAGTAAATAGAT

CCGAAACATATAAAATGCGGTTAATCCTGCTGTGGTCCAAGCTATTATTGCGAAAATCGG

CGAATACAACCAACTATCATTAAGAATTTCATCTTTTGACCAAAAACAAGCAAGAGGTGG

AATACCACAAAGAGAAATTGTACCTAATAAAAAAGAAATTTGGGTAATGGGTACATGTTT

TGTTAAACCACCCATAAGAGCCATATTCTGACTTTTATCTGGAGAATAGCCAACAATAGT

TTCCATTGAATGAATAATAGATCCAGACCCTAAAAATAATAATGCTTTGGAATAAGCATG

AGTAATCAAATGAAATAAAGCACTTCGATAAGATCCCATTCCTAGAGCTAACATCATATA

ACCCAATTGAGACATGGTCGAATAGGCTAAACCCCTCTTAATGTCTTTTTGAGCAAGAGC

TAAAGTAGCTCCTAATAATACTGTTATTATTCCTATGAACGAGATTAAATTCATTATGTA

AGGTATAACTATAAAAAGAGGAAGAAGGCGAGCTACAAGAAAAATTCCCGCTGCTACCAT

AGTAGCAGCATGTATAAGAGCCGAAATAGGAGTAGGTCCCTCCATAGCATCAGGTAACCA

TACATGAAGGGGAAATTGTGCAGATTTCGCAACTGCACCGGTAAATAATAGAGCAGCACA

CAAAGTAACAAATGAAGAATTGACTTTATTATTATAAATCAAGTTATTTAATATTTCGAA

TAAATCTCGAAATTCGAAACTACCTGTTATCCAATAAAAACCTAAAATTCCTAATAATAA

ACCAAAATCCCCTACACGATTAGTTACAAACGCTTTTTGACAAGCATTTGCCGCAATAGG

TCGTGTGAACCAAAACCCTATTAATAGATAGGAACACATTCCAACCAATTCCCAAAAAAT

ATAAATTTGTATCAAATTTGAACTAGTAACTAATCCTAACATGGAAGTACTGAAAAAACT

CATATAAGCAAAAAATCTCAAGTATCCTTGATCATGAGCCATATAATTATCACTATAAAT

AAGAACCATAATTCCAACAGTAGTGATTAAAATTGACATAATAGAAGTAAGTGGATCGAT

CAAGTAGCCGAATTCTAAAGAAAAATCATTATTGATGGTCCAAGACCATACATATTGATA

GATACAACTACTATTTATTTGCTGAATAGACAGATTAATTGAAAAAATCATGACTATACT

TAACAATAAAATACTCGGAAAGGCCCACATACGACGAAGATTTTTTGTTGCCGTCGGAAA

AAGAAGAAGTCCGACTCCTATTAACATAGGAACTGGAAGTGGAACAAAAGGTATGATCCA

CGCATATTGATATGTCTGTTCCATAAAAAAAGTTTTTTAATTAATTGTTTCCGATTCACC

GGATCTTACCTCTTTTGAAAGAAGTCAATAAAAAAATCAAGATATGCACTAATTTAAATA

GAATTTTCTAATTTTTGATTCTTACTTATTCTGCTAAATATTCCAAATATTCAAATCAAG

AAGTTCCAATTGGTCAAATCATATGAAAGAAAAAGATTAATTACTAGTCTCTGAAAATTA

AATAATTAGCCGTATTTTTACCGAGTTTGACCAGTTAATAG-AAAAAAA-GCATATTCTT

CTTCTTTTTTCTATTTTTAGTAATATTTTATGTCATTTTCACATATCTTTAACCTATCTA

TTTTTTT-T-ATTTTATAACAAAATATTTTGTAGAAAAGAAATTCCTAATGAATAGGAAA

GTACGGATTCATTTTGAATAATAGATGTCTTTCACATCCAGCTATAACAATGAGCAATCT

CTTAATTTTTATTTAAATGGCAGTTCCAAAAAAACGTACTTCTGCATCAAAAAAGCGTAT

TCGTAAAAATATTTGGAAAAGGAAAGGATATGGGACAGCGTTAAAAGCATTTTCCTTAGG

GAAATCTCTTTCTACCGGGAATTCAAAAAGTTTTTTTGTGCG--AAAAAAAAATAAGTAA

TAAAACGTTGGAATAATCTGAATCGACTTGACTCAAAAAATTGACTCATTTCGAAAATCA

AATTAATGAATTCCATTACCTATTGAGTAAATCAATAGGAAATGGAATTCGCTCCGCTCT

TAGAATATGTAGAATAAGAATTCTTCTGTTTACCTTACTCAATTCAATTCA-AAAAAAGA

AAAAAGACTTTCTTTTTGTTGACAAAAGAATAAACAAAAGATACTCCATTTTCTTTTTAG

TATATTTTCTCATTTCCAAGGCGGGGAGTCTTATTTTCCGCATCTCCCTATTTGTTACAA

TAATACAACTTTTTCTTTTTTCTCTATATCCACTTTTTCTCTCTATCTCAAGTTGAGGAG

AGTATAAAATACACGAAAATCTTAAGAAAACTAAGGCCCTAAACTAAAATAATGAACCTT

TAAATACCTATTTGAACTAATTTTTATTCATCCATTTGAATCTTTCCTAAAAAATATTGC

AACCAATTGAATTCTTAAATCTAGACGATTGCTTATTCATAGGCTATTATGAGTTCAAGA

CAAGCCGCTATGGTGAAATTGGTAGACACGCTGCTCTTAGGAAGCAGTGCTAGAGCATCT

CGGTTCGAGTCCGAGTGGCGGCATGCCATCTTCTAAAAAAACTAAATAGATCCTATAATG

AATTCAATTCCTGATTTCTTGTAATTAAAGAACTCCTATTTTAAATTTTTATGATATTTT

CAACCTTAGAGCATATATTAACTCATATTTCTTTTTCGATTGTTTCAATTGTAATTACAA

TTCATTTGATAACCTTTTTAGTCGATGAAATCATAAAACTCTACGATTCATCAGAAAAGG

GCATGATAATGACTTTTTTCTGTATAACAGGATTATTAGTTACTCGTTGGATTTCTTCGG

GACATTTTCCACTAAGTAATTTATATGAATCATTAATCTTCCTTTCATGGAGTTTCTCCC

TTATTCATATAGTTCCATATTTCAAAAAAAATAAAAATTTTTTAAGCGCAATAATCGGCC

CAAGTGCTATTTTTACCCAAGGCTTTGCTACTTCAGGTCTTTTAACTCAAATACACGAAT

CTGAAATATTAGTACCCGCTCTTCAATCCGAGTGGTTAATAATGCACGTAAGTATGATGA

TATTGGGCTATGCAGCCCTTTTATGTGGATCATTATTATCAGTAGCACTTCTAGTCATTG

CAGTTAGAAAAAGCAGAAAGTTTTTTTTTACAAGTAATCATTTATTAAATTTAAATGGGT

CATTTTTCTTTAGTGAAATCGAATACATGAATGAAAGAAGCAATGTTTTAAAAAATACTT

CTTTTTTTTCTCCGAAGAATTATTACAGGTCGCAATTTATTCAACAATTGGATTATTGGA

GTTATCGGGTTATTAGTCTAGGATTTATCTTTTTAACCATAGGGATACTTTCTGGAGCAG

TATGGGCTAACGAAGCATGGGGATCATATTGGAGTTGGGACCCAAAGGAAACTTGGGCAT

TTATTACTTGGATCGTATTCGCGATTTATTTACATATTCGAACCAATATAAAATGGAAGG

GTATAAATTCCGCAATTGTGGCGTCTATTGGCTTTCTTATAATTTGGATATGCTATTTTG

GGGTCAATCTATTAGGAATAGGACTACATAGTTATGGTTCATTTACATTAACATCTAATT

GAATTCAAAAGGATTCAAAAAAGACTCTTACGAATAGAAAAGTGTACAGTGCATATGAGA

TAAAAAAACTTTATGTGAACTGATGAGAACCCTGTGAATCAAATATTGTAGTGATTCACA

GGGTTCGCGCCGATTCAATTTTTTTACTTAACTTAAGAGAAGAAGAAAAAGCCTTCTTTT

TTTCATTGTACAACGAACGATT-AAAAAAAAATCATAGGATTTTTTTTTTTATTCATCAA

AACTATCTATAAAAAGAATTAGATAGAATAACTTCGACCTTGTCAACTGATAGTGAAAGA

ACAAAATCGGGGTACATACCAATACCTAGTATGGGTAAAAAGATAGAGATTGAAAGAAAT

AACTCTCGCGGTCCAGAATCAAAAAAATAAGAGTTTGGAGCATTAAATATCTTGTATCCA

TAGAACATCTGGCGTGACATAGATAATGAATAAATAGGAGTTAATATCATTCCAATTGCC

ATTACAAAAGTAATTAGTATTTTTGGCATTAAAAGGTATTTTTGACTGGTAATTAGTCCA

AAAAAGACTATTAATTCGGCAACAAAACCACTCATACCTGGTAATGCAAGGGAGGCCATC

GAAAAGCTACTGAACATCGTGAATATTTTTGGCATTGGGATAGCTATTCCACCCATTTCG

TCAAGATAAACAAGACGTATTCTATCATAAGTCGTTCCTGCCAAGAAAAAAAGTGCAGCA

CCAATAAATCCATGAGAGATTATTTGTAAAAGGGCTCCATTGAGCCCTGTATCGGTGATA

GAACCAATTCCTATAATTATGAAACCCATATGAGATACAGAGGAATAGGCTATTCTTTTT

TTTAAATTCCGTTGACCGAGAGATGTTGAAGCTGCATAAATTATTTGCATTGCGCCTACT

ATCATCAACCAAGGAGAAAATATAGAATGAGCATGTGGTAATAATTCCATATTGATCCGA

ACTAATCCATACGCTCCCATTTTTAATAAGATTCCGGCTAGAAGCATACAAGTACTATAA

TGCGCTTCTCCATGGGTATCTGGTAACCATGTATGTAGGGGTATAATCGGCAATTTGACA

GCAAAAGCAATAAAAAATCCAATATAAAATATTATTTCCAAGGCCACAGGATAGGACTGA

TTGGCTAATATTTCTAAATTTAATGTTGGTTCATTAGAACCATATAAACCGATACCCAGA

ACTCCCATTAAAAGAAAAACAGAACCCCCCGCCGTGTACAAAATAAATTTGGTAGCTGAG

TACAGACGTTTTTTTCCTCCCCACATTGATAGAAGTAGATAAACGGGAATTAATTCTAAC

TCCCACATGAGGAAAAAAAGTAAAAGGTCCCGACAAGAAAATAATCCTATTTGACCGCTG

TACATTGCTAACATCAAGAAATGAAATAGTCGAGAATCCCGAGTAACTGGCCGAGCCGCT

AAAGTAGCTAAAGTAGTGATGAATCCCGTCAGTAAAATGGGTCCTATAGAAAGGCCATCT

ATTCCTAATCTCCAATGGAAATCAAAAAAATCGATCCATTTATAATCCTCCACTAGTTGG

ATTAATGGATCGCCCGATTGGAAATGATAACAGAATGCATAAGTCGTTATAAGAAGTTCT

ACAATACATATACATATTGTATACCAACGAATTCCCCTATTTCCTCTATGGGGAAGAAAG

AAAATTAAGGAACCCGCAGATATTGGCAAAATAACAATTATTGTTAACCAAGGAAAATGA

TTCGTGGTAAAGACAAGATACACTTGGGCCAGAAAAACCCGTGCTCAAAATATTTTGAGC

ACGGGTTTTGTCGGTAAAAAAATCAAATGGATTCAAGTAGAGTTTTCTGGAACGTATCAA

TAAGCTAGACCCATACTGCGAGTTGTTTCATGCCATAAATAAACTCGAACACTCAAGAAA

TCCGTTGGACAGGCGGATTCACATCTCTTACAACCAACACAGTCCTCGGTCCTTGGGGCA

GAAGCAATTTGTTTAGCTTTACATCCGTCCCAAGGTATCATTTCTAATACATCGGTGGGA

CACGCTCGGACACATTGAGTACATCCTATACATGTATCATAAATCTTTACTGAATGTGAC

ATTGGATCTATACATTTTTTTCGTCATAAATTTTCGATCTAGTAAACTTATAAATGAATC

ATATATTTAGATACCAGACGAATCAATGAGTGATCAGAATCAATCTACTTGCGGATTGGT

TTATGAGAGAGGGCCAAAATACTTTGATTTCTTATGTTTTTGCAACCACGATCCTACCTT

ACACGTAGCAAACCTGCTAATTCGAATTCATTTCAGAATAATTTCTGAATATTCGAATTT

CTATTTATATAATTAATACTATTTATTCAACAAATTGGATTGGTTAATACGAGTTGATTT

TCTGTTACGATAAATTGATGAAACAATAGCCGGTCCAATAGCTGCTTCAGCGGCTGCAAT

AGCTATAACAAAAATGGAGAAAATATCTCCTCTTAATTGACGATTATCAAAAAAACCAGA

AAATGTTACAAAATTTATATTAACTGAATTTAATATAAGTTCAAGACACATAAGGGCTCT

AACCATATTTCGACTTGTGATCAATCCGTAGATACCGATGGAAAATAAATAGGCACTCAA

AACAAGTACATGTTCAAGCATCATTAACCAACTCCTTATCAATCTCGATTCATTTCAATA

TGAACAATAATTCAAGCGATTCGATTCTAACAACAGGGAATATATTGGTAATAGATCTAC

CTAAAGCTAAAGCATTTCTATTTTAGACAGGAATTCAAATTAAAGGATTATAACATTCAT

TTCCGTTGATTTTATTTGAATGGAATTCCTAGTTTTAAAGATTTATTATTGACGAGCTAT

TGCAATTGCACCTATTAAAGCGACTAAAAGAATTATTGAAATGAGTTCAAATGGAAGAAA

AAAATCTGTTGATAAATGAATTCCAATTTGTTGGCTATTACTTATCAAATCTTGCTCTAT

AATCTGGTTTGATTTTGTAGTCCAAATAATCCCGTACCATGACGTATCCGGAATAGTAGT

AATTAGTGAAATAAAAAGACTTGTACAAACTATTGAAGTAACTCCATCCCCAACGGTCCA

AAGATGAAAATCTTTGTAATATTCTGAACCACTCATGAACATCACAGCAAAAATGATTAA

AATATTTATAGCTCCCACATAAATAAGGAGCTGCGCAGCAGCTACAAAATACGAGTTCGA

TAGAATATAGAATAAGGATATACAAAAAAGAACCAATCCCAACGAAAAGGCCGAATAAAT

TGGATTGGGAAGTAATACTACTCCTAGACTTCCTAATATAAGACCCGATCCCAGAAAGAC

TAAAAGAAAATCATGTATTGGTCCAGGTAAACCCATTTGATTTTATAGAAAAAA-ATAGA

TAAATCGAAATATTTCATGACTTTGTTGACCTGACCAGGAAAAAAGAAGTTATCTTTGAG

GATACTTCTTAATTGAATTAAATTGGAATGGGTGTGATGTGGATTGATGTAGATACAGTT

ATGGGACTACTCTTATTCTTTATCCGAAAGAGGAGTTTGAAACTCTCTATTTTGAAATCA

TTTGAATCAAGGATTTTTTCCATTTTTTATTTGAGGTGAATTCGAAATTGTTCGAATTGT

GTAATCGTCAATTATTGACACCGGTAACCGACCTAAAGCAATTTGATTATAATTCAATTC

GTGACGATTATAAGTAGAAAGTTCATATTCTTCAGTCATTGATAAACAATTTGTTGGACA

ATACTCAACGCAATTACCACAAAATATACAGATTCCAAAATCAATACTGTAATTAAGCAA

TCGTTTCTTTCGAATATCCGTTTCCAATTTCCAATCAACAACGGGTAAATCTATAGGACA

TACACGAACACATACTTCACAAGCAATGCATTTATCAAATTCAAAGTGGATTCGTCCTCG

GAAACGTTCCGATGTGATCAATTTTTCGTAGGGGTATTGAATAGTTACAGGTAAACGATT

CACATGGGACAAGGTAATCATGAAACCTTGACCAATGTACCTTGCAGCTCGTATTGTTTG

TTGACCATAATTCATGAACTCAGTTACCATAGGAAACATATCGTAAATATCTATAAATAA

TTTTATACTTGTTTCTTTCTCTTGTTTGAGACAAGTTGTGAATATAGAATATTCTATTCC

TTTACAGTGAAAGAAGTTGGAATGAAGTTGTTAATAATAGATTACCTAGAGAAATAGGTA

AAAGAAATTTCCATCCAAGATTTAATAGTTGGTCCATTCTCAGTCTCGGTAAAGTCCATC

TTGTTGCAATAGAAATGAACAAGAACAAATAAGTTTTAGCTAATGTAATAAAGATACCGA

TTATTGTTCCAAAAACTTTACCTCTTTCAAAAAGCTCAGGAACGAATATGTACGGAATAG

AAAGATTCCAACCCCCCAAGTAAAGAACTGTTACAAATAATGAAGAAACTAGTAGATTCA

GATACGAAGCAACATAAAATAAACCAAATTTGATACCTGAATATTCGGTTTGATAACCTG

CTACTAATTCCTCTTCTGCTTCTGGTAAATCAAAAGGTAATCTCTCACACTCGGCTAGAG

AAGAAATTAGAAAAACGATAAACCCTATAGGTTGACGCCACAAATTCCACCCCCAAAAAC

CATATTTTGACTGCGCTTCAACTATATCAACTGTACTTGAACTGTTAGATAATCATAGTC

GATGATAACATCACTGTTCCCATCGCTATTACAGAACCGTACATGAGATTTTCACCTCAT

ACGGCTCCTCAGAGGTCGCAAATAAATCTAAGTACCTTTCGACATTATTTATCTTGATAT

GTTTATAGGATAGATAGAGATTCAAACTCTTATCCTAAGGTAGACCAATGGAATTCTGTC

TGCTATTTCTAAGAAATATATATTTCTTAGAAATAAAAGTGCTTCTGAATTGATCTCATC

TTTTAAGAATTTTCATTTTTCTTTGTTGATTAATAACTTTATCCTTGAATAAAAAAAGAG

TTTTTTTGGAGGAATATCACATAGCTATTTCAACCTATCATTTTCCATTACGAAAAAGAA

TTAGACATTGCATTAGTTCATGCATCATGACAAGAATTCTATCTCTCTAAATAGGTATAT

AGGAAAGAAATAATAAAAAAAAATCTCTTTTTTGCAATTCTATTCTTTCGAGTTTATTTC

GTTCCTATTCTCCTTTCTCAAAAAAGGGGGACATTACCAAAGTAAAAGATTACTTCGTTC

TTGATAGTTATTTACTTAATCAGTGGATAGGAACATACTCTGGATCGAAATCATGGGGAG

TACTTCTTAATCGTTTCTACCAACTTAAAGCCCCAATTTGAATTCCTTTTAGGTACATCC

TGTTGGATAATTTACAGAATCTCCATTACTAATCCTTTGCGTATCTTGGTCTTCCTAACC

ATCCACTCATTTTTGTTAACCTTCCATTATGGTAATACATCTATGTTAATAGATAGTAAA

AACTCCATACAGTTGATCTTTTGAACCCGTTTCAAGCCATGATGCCTAATCAACCAATCT

TGGGGTAAACAGTCTCGACTGCTTTGCTTATATTTACTTTCATTTCATTCTTGTACATAG

GAAATGAGATTCAATCCCTTTAACTGCAAAT-AAAAAGCCGTTTTATTTCACTCATATAA

CTATCTGGTTTAGTTCATCAACCCGAATTCTGAATAAAAAAAAATATATATATTAAACTC

ATTTAACTTTCTTACTAGAAAGAAAAGAAATAGGGGAAATTTTATGTCTCACCGAATCAC

ACGTAGAGATATTGATAATACACATAGAGTTAATGGTATTTCATAACTAATTGATTGAGC

AGCAGCTCTTAAACCACCTAAAAAGGAATATTTATTATTTGATCCATATCCCGACATAAG

AAGTCCAACGGGAGCAAGACTTGAAACAGCGATCCATAAAAAAACACCAATACTAAGATC

GGCTAGAATAAGGCGATAACCAAAAGGAATTACTGAATAACTTAGTAAAATGGATATGAC

TGCTATGGATGGTCCGATACTGAATAAACGAGTATCCCCTCTAGATGGAAAAAGATTCTC

TTTGAAAAGTAGTTTTACCCCATCTGCTAGAGCTTGAAGAATTCCCAAGGGGCCGGCGTA

TTCAGGTCCAATACGTTGTTGTATCCCTGCAGATATTTCTCTTTCTAACCAAACAATTAC

TAGTACACCTAGTGTGATTCCTAATACAAGAGTCAAAATAGGGACAAGCACCCATATGAT

CCCATAGACTTCTTTTAAGAATTCCAATCTGGAAAACGAATGGATGGCTTGTAGTTCTGT

TGTATTATCAATTATCATTTCAACGATCAACTTCTCCCATAATGATATCTATACTACCTA

GTATCGTCATAATATCAGCCAATTTCATTCTTTTAACTAACTGAGGCAGAATTTGCAAGT

TGATAAAACCCGGTGGGCGAATTTTCCATCTCCAAGGAAAAACACTCTGATCTCCTATCA

GAAAAATTCCCAATTCTCCTTTTGGTGCTTCGACTCTTACATAAAGTTCTTGTTTGGACA

ATTCAAAAGTTGGAGAAGGTTTTTTACTAATAAATCGATATTCAAAATCATTCCATTCAG

TATCTTTTACTCTATCAAAGCGTCGGATTTCTAAATTCTCAAAGGGCCCCCCTGGAATTC

CTTCCAGAGCCTGTTGGATAATTTTTATGGATTCTGTCATTTCACTGATTCGTACTAAAT

AACGAGCTAATGAATCCCCTTCTTTTTGCCATTGAACCTCCCAATCAAATTCGTCGTAAC

ACTCATAATGATCAACTTTACGAAGGTCCCATTGTATTCCGGAAGCTCGTAGCATTGGTC

CTGATAAACCCCAATTTAGTGCTTCTTCTCCTCCAATAATGCCTACGCCCTCAACTCGTT

CTAAAAAAATAGGATTCCGTGTAATAAGCTTTTGATATTCAGCAACCCCTGTTAAAAAAT

AATCACAAAAATCCAAACATTTATCTATCCATCCATGAGGTAGATCAGCAGCTATTCCTC

CGATACGAAAATAATTATGCATCATTCGCATACCGGTGGCAGCTTCGAATAGGTCATATA

TCAATTCTCGTTCTCGAAAAATATAGAAGAAAGGGGTCTGTGCCCCAATATCTGCCATAA

AAGGACCAAGCCATAACAAATGAGAAGCTATACGACTCAACTCCAACATAATGGCTCTGA

TATAGCTAGCCCTTTTAGGTACTTGAATATTGCCTAGTTGTTCGGGTCCATTTACGGTTA

TTGCTTCTGTGAACATAGTAGCTAAATAATCCCAACGTGTTACATAAGGCAAATATTGTA

TAATTGTTCGGTTTTCCGCAATTTTCTCCATTCCTCTGTGTAAATAACCCAATATTGGTT

CACAGTCAATAACATCTTCACCATCGAGAGTAACGATAAGTCGAAGAACACCATGCATTG

ATGGGTGGTGAGGACCCATATTGACTATCATGAGGTCTTTTCTTGTAGTTGGTGCAATCA

TAAGTTTTTTACCGAGTCATTCTTCCATGAATTGCTGAAAGTAAAAAGAAGTTCATCAAA

ATTGAAACGCATAAGTTCAAATGATATCACTCTTTAAATTAACGAGTTTTTGTCTCTCGA

ATATCCAACCGATCAATTAATTCTTTATAACGTACTCTATTTTTTTTTGACAAATAAGCC

AGTAATCGTTGACGTTTTCCCAAAATTTTTCGCAAACCTCTCTGAGATGAATAGTCTTTT

TTGTGCAATTCCAAATGTGAAGTAAGTCTCCTTATCTTAGTGGTGAAACTGAATACTTGA

AATTCAACAGACCCTCTGTTTTCTTCATTTTCTTTTTGAGAAATAACCGAAATGAATGAA

TTTCTTACCATAAAAAAAAGCCTCCTCTCCCTTTTTACAGATATGGATTTTACCGATCAG

TAATAATAATGTCATTCATTTTAATGTGGTATATACAATAATCTAATTCAAATTTCTTTA

TGAACTCCTAATTTTATCAATTCAAATGAATCAATCCAATTGAAAATTAGATTTAGATAG

GGAGAGAAAAGGATTGGCACATTTTCGTATTCACAAAAGCGAAGAATTTAGACCTAAAAT

GAAGAGGGTTCTGTTGATTCATTTCTAGATCGAATTGATACATTATTCATTTAGTATTGG

ATATTTAGAATGAAATGTAAGACAGGACGTGTGATGTGTGTATTTATTTGCTTTCATATA

TCCTATATAGTAGAGGATATATAGGAAAAATGGACTATCAACGAATTTTCAATCGTGGAT

ACAAATGTATCCTTAACATACTGAAACGACTGCCATTATTGGTATCAAACCAATAGCGAT

TCATACAAGCTAAATCTTCTAATCGATAATTAGGCCAAAGAAAGAACTTCAATTTCATTA

ATTGATTTTTCTCTCTATCAAGGTGATTACTGTCACCTAGAACTTGGCTGCTATTCTTTT

CGTTGCAAAATACAGGATTTCCATCTACATCATTCCTATTCTTTGAATTGAAAGAAATTA

GAATTCTTAATTTTCTACGACGCCTAAATGAGAAAATATTTTCAGGAACAAGCAAATCCA

AATGATTTTTGTCTCTATTTCTTGTTATTCTTTCATGTGGTGGAATAGATTCATCAAAAT

TATTCTTAGCAACATATCTTTGCTCTCGGTATCTTTGATTAGTTTGGTGCTTACTCTTAT

GAACCAACAAAATACCGATGGTTTGATACATAATAAACTGTCCGTCGTTTTTTACAGACA

GACGAATGGGTTCGATAATAAATATTCCCTTTTTCATCAATTCTGGAAGAGTTAAATTCT

TCTGAATCAGCATTATATCCAAACTCATTTCTCCCCTTTGAATCGAGGATATAGAAATTT

TTCTTGGATCTATTAGTCTAAGCAGGAAACAATATACCTTAATATTATTGATCAGTCTTT

GATTCAAAGTATCGTCCCATCTCAATTGAAAAAGCAAATAACGTTTCAGGAACAAATCTA

GTTCTGCTTCCTTACTTTTGTATTGTTTTTTCTTTTTACCTTTTTTCATGTCCGATCTCG

CATAATCTTCTTCAATATCTTTTTGTTGGTTTGAAAGAACGGATCCAAGATCCCCTTGGC

TTGTGGTTTTTTTTTCTTCTTGATTTCGATTCTTTATTTTTTTATTCGATGGTATCAAAA

AACTTCCCTTTTGCTTTTCATTAATCTTTTGATTTTGATTACTATTTTCATTTCTATTCA

AATTTAAAAGAAGTAATTTGCTTGGTATAAACCAAGATTTCGTTTTATATGTATTATAAA

GTAACAAAAATTCTGGGAAGAACCAAAGTTCCAGATTCAATATGGGACGCTTTAGTATTT

TTTCATTCATCCCCATCCAATCAAAAAAGACTTTTGGGGAGTTTGGTAAATTCATTTCTG

GAATCATAAGATAAAAAAGATTTTTTTTATCAATTATTTGATAATTATTAGTAACAATCT

GAGTATTTTGATTACTATTGGCATCGATCGTGATCCAGGCCTCAATATCGACTTTTTGTC

TAAGATCAAAATTGATAATTTTCCAATCCAAATATTTCCTATCGGGAGTTTTTTCCATAT

ATAGAATATCGCCCTTTCCTAGATAATTATTGATAGGGATACTCCTCAGCATATCAAAAA

AAGTGTCTTTATATGTGTTGTAATTATAAGAAATCTCTTGATTCTTATTTCCTTCAAACG

GCGATCTAGAAATAAATGAGTCCTTTTTATTTTCAGAATTAATAGATTTATATGATAAAA

GATCATATTTATAGTATTTTGGAAAATTATCTTTTTGATTCAATAATGAATAGACTTCCA

AAATATTTTCTTTTTTGTAATCAATTAATTGGTCTTTTTCATATAAATTCCATTTGCTGA

AATTTTTCCTTTTAACCATACGACGTTGATAAACTCTATTCCGCCATTGTTGTGGTATTA

ATCTAGACCATCTGATCTGAGATAAATCGTATTGATAATGCCCTCTTAACCAGTTTTTCC

ATTGATTCATTTCAGAACTCGGAAGTCTGTTATCCCTTAATTTAGAATGAACTATTCCTT

GTGTTTCAAAAGAATCCTTTATTTCAGGCTTAAGAAAAAAAGGGATTCCTTGATATTGAA

ATGATTTTAATTTATACAAGTTAATAACTTGGGTTTGTGATAATTTGTAAAATACATATG

CTTGTGATAAGTACGATAAGTCATAAAAAATATGTGAATTTGTCTGACTATTACTAATAT

TATAAAGTGACTTTTTTATAGTCGAAATAAACTCAATTGGATTTTTTTTTTTTTTATTAA

TTATTTCTTGACTTGTTTCATTATTGTAAATGGATTTATTCATAATTTTTTTTGTTGATT

CAAGAAATAATTTTCTCTTTATTCTGGGAATATTAATGATAGATAAAAATATATTTGTGT

AGATTCTTTCAATTAAAAATTTAAAAACAAAAGGTAATTTAGAGATTAATCGAGCATTTC

TTCTTTTTAATATTTGCCATTTTTCTAATCTTTTAGCATTATAACTTGTTTTGTTAAGAC

TAATATTTATTTCTGGAGTTACTTTTTTTTTCTCTTTTGTAATTCTTTCTATTTGATTTC

TAATTGTATTTGTTTTATCAGTCAGATCCTTCATTTTTTTTTCTGTCAATGAAGAATTTG

TCCAACCTGGAGATGCAATTTGACTAAATGACTCATGAATCATCTGATTGCTGATTATGG

GATCTCTTTCTTTTTTAGTTTCACTCGAGTCATATACTTCTACTTCTCTTGATCGAAATA

AGAGAATTGGATTCACTTTTAAGAGTTCTTTTCTTATTTTTTTAAAAAAAACGACACTTT

TGATAACCCATTTTTTTGTTTCTTTTGAAACTTTTCGAAGTAATTTTATTTTTCCTTTTA

AAATTTTTAGAAGTAGAAAATATTTCTTTTTTAATTTTCCAATTTTTTTTTCGAGTTCCT

TAAAAATGGGTTCAAAAAAAGAGGGTTGTTTTCGGGGAGAACCAAAAGGAAGTTCGGTTT

CCATTCCCAAAACTGTTAAAAAACAAAAATCATCTTTTTCTTTTTTCTTTTTCATTATTA

GATCTTTATGAGAGAATCGGAGTTTAGATCTGTGCCAAGGTTTCAGACAGAAAGGAAATA

AGATTTTTATCTGAATACCATCTGTCAACCAATTTTGTGGAAATTCTGTTTCGGACAATT

GAACACCATTATAGGTACATTTAACATGCATCTCCCTATTCCATTCCTCTAAATCCTCAT

ACCATTCAGGAAGTTGCAATAGTAGCATACGTCCAATATTTTTTATTATCAATGAAGGTA

ATATAATATATTTTCTAAAAATAGATTGAGTTATTAACATGGAACCCCTTATTACTTGCG

CAAATGGAATGGTATCCCAGGTCTCTGCTATTATTCGCGCGATCTCCTTTCTTTTGTTCT

CTTTTTTTTCTTCTCTTTTGTTTGCTCTTCTGTATACTCTACAATTTTGAACGCGGACCC

CCTCCCTACCCAATTTCTAAAAATTTTAATTGGCCCAGAGATATCAAAAGAAAAAAGAAG

GGATTTTTTTATTCTGTCCAAAAAAAGAGGGGAATGCACATTTGCTTGAAACAGTTCCCA

AATAACTATTTTACGCCTTTGAGCACGTATAGAACCTTTTATTATGCCTCGCTTAAAATC

TGATTGTTGTGAATAGCGTAGCAAAGCTACTTCGTATGTTTGATCAGGAGGATTAGTATT

CTCAGTATGCTCCTTGTTACCAGTAAAAATTACTACACGTTTGGCTTTTCTTGAACGAAT

TTCATGATCTAATGGTCCGTTTTCTTTATCTTCTCCTGCTTCGTGTTCCAACTCGCTGGT

TAATTTGTATAACCAGCGAGGTACTTTTTTACTGATTTCTTTTATTCTAATCGATTTTTT

ACTAATTTTTTGAACATTAGTATCAGTTAGAATTCTATTTTTAAAATATTTCAAATATTT

TGTTCCTTTTTCTGAATCTATTCTTCCTTCTTCTTCATCTGAAAATAAAGAAAGACCCCT

AGAATTAAAATGGATCCTGATTCTTTACCAAGTTCATTGATGAAAGTTAAGAAATCAACA

ATTTCGGTTGATAATGGTTTTTTATCAAATCGATCTATTTTCTGTTCAATTTTTTGGTAA

TCAGTATCAGGAAGAACGATACCATGAATACGATTTATCCCAACTTTCTCTATGAAATTG

TCTATCGAAGTTTTTTTTATTTTTATGATTGAAGGTGAAACGCTTTTTGTTATTGTTCTC

CGATATGGTCCGTTCAAGAAAGGATCATATATTTTGGGTAAGTATTCGTTTGTAGTATTG

TCATTACACAACCTAGTCCTTGTTTCGAGTATATTCAAAAAAAGAGATTCTTTGTCTAGG

GCTTGAATTCGATTTACAAATTCGTTGTTTAAATTATTACTTTTTTCTTTGTTGGTATAA

ACCCAATGATTGTAGAGTTCATTAGATGAGAATTTTTCTAGTGTAGGCAGAGATATCCTT

CTTTTTATCATTTCCCAAAAAGTTGACAAACTGGGTGGATATGTAAAAGATATTCTTTCC

TTTCCAGCACTTTGGCATGTGTCAAAAAAATATTGTGACATTTCATTTCTGACAGCGTTT

TCAAATCGATTATTTTTTATGTATCGAAATGGCCGATTCCAACGGTTAGAATCAAAAAGA

AGAGTCACAAGAGGTTTTTCAAACCAAAAACAAAA

>Nestegis_cunninghamii

TATCATTTTAGTTATGGGCGAACGACGGGAATTGAACCCGCGCATGGTGGATTCACAATC

CACTGCCTTAATCCACTTGGCTACATCCGCCCCTCTTAGATTTTTTTATTTTAAGACAAA

AGGTTGAATTTCGACCATTTCATTTATCTTCTTTCTTATTTGCTTATTTGTGAGATATTT

TTATCTCAGAGATAAAAAGATTAAGCAAAAATTTGAATTTTTCTCGTTTTCATTTAAATT

TAAATGTCAAAAAAACTTCGCAAAAGATGGATAGATAAATGAAAATAAAAATGCATATAG

AACGAACAAATAATACTCAATCATCAATCAACCCCTAAGAAAAATAATCCCCTTTATTTC

TTTTTCGGTAATGTAACGAATAAAAGTCTATGTAAGTACAATATGACTAAAAAAAGTAGT

AGTAATTAAAAAGAAAATAAAGGAGCAATAACGCCCTCTTGATAGAACAAGAAAGAGTTT

ATTGCTCCTTTATTTTC--TTTTTTTTTTCAATAACTCCTATACACTAAGATCGGGTCTT

ATCCATTTGTAGATGGAACTTCGATAGAAGCTAGGTCTAGAGGGAAGTTATGAGCATTAC

GTTCATGCATAACTTCCATACCAAGGTTAGCACGGTTAATGATATCAGCCCAAGTATTAA

TTACACGGCCTTGACTATCAACTACGGATTGGTTGAAATTGAAACCATTTAGGTTGAAAG

CCATAGTGCTGATACCTAAAGCAGTGAACCAGATACCTACTACAGGCCAAGCAGCTAGGA

AGAAGTGTAACGAACGAGAATTGTTGAAACTAGCATATTGGAAGATTAATCGGCCAAAAT

AACCATGAGCGGCTACGATATTATAAGTTTCTTCCTCTTGACCGAATCTGTAACCTTCAT

TAGCAGATTCATTTTCTGTGGTTTCCCTGATCAAACTAGAAGTTACCAAGGAACCATGCA

TAGCGCTGAATAGGGAGCCGCCGAATACACCAGCTACGCCTAACATGTGAAATGGGTGCA

TAAGGATGTTGTGCTCAGCCTGGAATACAATCATGAAGTTGAAAGTACCAGAAATTCCTA

GAGGCATACCATCAGAAAAACTTCCTTGACCAATTGGGTAGATCAAGAAAACAGCGGTAG

CAGCTGCAACAGGAGCTGAATATGCAACAGCAATCCAAGGTCGCATACCCAGACGGAAAC

TAAGCTCCCACTCACGACCCATGTAACAAGCTACACCAAGTAAGAAGTGTAGAACAATTA

GTTCATAAGGACCCCCGTTGTATAACCATTCATCAACGGATGCCGCTTCCCAGATTGGGT

AAAAGTGCAAACCTATAGCTGCAGAAGTAGGAATAATGGCACCTGAGATAATATTGTTTC

CGTAAAGTAGAGATCCAGAAACAGGTTCACGAATACCATCAATATCTACTGGAGGAGCAG

CAATGAAGGCAATAATAAATACAGAAGTTGCGGTCAATAAGGTAGGGATCATCAAAACAC

CAAACCATCCAATGTAAAGACGGTTTTCGGTGCTGGTTATCCAGTTACAGAAGCGACCCC

ATAGGCTTTCGCTTTCGCGTCTCTCTAAAATTGCAGTCATGGTAAAATCTTGGTTTATTT

AATCATCAGGGACTCCCAAGCACACTAATTTTCTATAATATAAATCGAAATAGAAAATGG

AAGGCTTGTTATTTAACAGTATAATATGACTTATATGGTCGTGTCAA-----CCAATCCA

ATAACTATCTAGATTTATTCGAATTTTTTGTAAATGAATGAAGTGGATTGCAAAAAGAAA

ATAAAGATTTCTATACATAAAATTTCGATATGACAGTGGGTTGCCCGGGATTCGAACCCG

GAACTAGTCGGATGGAGTAGAAAATTTCCTTGTTATGTTAATTAAATAAGGAAAAACCCC

TCCCCAAGCCGTGCTTGCATTTTTCATTGCACACGGCTTTCTCTATGTATACATCATTTC

CTTTCTTATAAAGACTTTAAAAAGTTGAATACTCAGTTGATTTAACCCTTATTACATACT

ACATCAACATTTCAGAATAGCGGAAATCACATTTTTATTTTATTATCTCTTCATCCATTT

AATTTAGGAAAAATTTCTATTTCCAAGCTTTCAGAATAATTATTTATGATTGACCAGATC

ATTGATACAAATAATATCCAAATACCAAATCCGACTTCTATATACTCCCCGCAAAGTGGA

AGAAGCTTTTGGGAAGGTCAAAGAAAGAACTTGTTCTTCCGACATAAGAAATTCTTCCAA

TAATTCCGAGCCTAATCTTTTCAAAAAAGCACGTACAGTACTTTTGTGTTTCCGAGCCAA

AGTTCTAGCACAAGAAAGTCGAAGTATATACTTTATTCGATACAAACTCTTTTTTTTGGA

AGATCCGCTATGATAATGAGAAAGATTTCTGCATATACGCCCAAATCGATCAATAATATC

AGAATCTGATAAATCAGCCCGAACCGGCTTACTAATGGGATACCCTAGTACGTTACAAAA

TTTCGCTTTAGCCAATGATCCAATCAGAGGAATAATTGGAACAAGAGTATCGAACTTCTT

AATAGCATTATTGATTAGAAATGAATTTTCTAGAATTTGACTCCGTACCACTGAAGCGTT

TAGTCGCACACTTGAAAGATAGCCCACAAATTCAAGGGAATGATTAGATAATTGGTTTAG

ATAAATCCTTCTTGGATGAAACCACAGCGAAAAATAACATTGCCAAAAAGTTACAAGGTA

ATATTTCCATTTATTCATCAAAAGAGGCGTCCCTTTTGAAGCCAGAATTGATTTTCCTTG

ATACCTAACATAATGCATGAAAGGATCCTTGAACAAGCATAGATTGGTCTGAAAATCCTT

AACAAAGACGTCTACAAGACGTTCTATTTTTCCATAGAAATATATTCGTTCAAGAAGAGC

TCCAGAAGATGTTGATCGTAAATGAGAAGATTGGTTACGTAGAAAGACGAAAATAGATTC

GTATTCACATACATGAGAATTATATAAGAAGAAGAATAATCTTTGATTCCTTTTTGAAAA

AGAAGAACTGGCTTTCTTTGGAGTAATAAGACTATTCCAATTACAATACTCGTGAAGAAA

GAATCGTAATAAATGCAAAGAAGAGGCATCTTTTACCCAATAGCGAAGAGTTTGAACCAA

GATTTCCAGATGGATGGGGTGGGGTATTAGTATATCTAACACAAAATTTAAATGTGAAAA

ATTGTCCTCTAAAAAAGGAAATATTGAATGAATTGATCGTAAATTATGAGATTTTACTAT

CTTTTTTTTTTTCCCTTCTAGAGAAAGTATTAATCGTAGAGAAAATGGAATTTCCACAAT

AAATGCAAACCCCTCTGATATGATTTGAGGATACAAATTCTTGTTGCGCCCCAAAAATTG

ATTTTGGTTAGAATCATTAGCAGAAATAAGAAAATGATTCTGTTGATACATTCGAGTAAT

TAACCGTTTTACAATTAGTAAACTGGATTTATTGTCATAACCTGGATTTTCCAACAAAAT

CGATCTATTGAAACCCTGATCATGAGCAAGTCCATAAATATACTCCTGAAAGATAAGTGG

ATATAAGAAGTCGTGTTGTTGAGATCTATCTAGCTGTAAATATCTTTTGATTTCCTCCAT

TTGAAATTTTATTTGAACCAAAGTTAGAAGAATTTTGTGGGTTATCAAATGATACATAGT

GCGATACAGTCAAAACAAGGTATTCTAGTAAGAATAGATACCTCGGAAACAGGTAAACTT

ATCAACAGATTCTCTACCCTCTCTTTTTTCCATTTCATTTAATTGGTCTATGTTATAGGA

TAACAAGATGGTTAGAAATCTTTTATTTTTTGAACCTAATCGCTCTTTTGATTTCGG--A

AAAAAACTTTCTTTATCAATATACTGCTTCTTTTACATACGCATCTCCATTCCATAATGG

AGAATGCCAATAGTTAGGATTCATTAAAAAAATATAGAATCCACTCATGGGGGGAGAAGT

CCTTGCCGTATCAGGCACTAATCTATTTTTAACGTCTAATTAGATCGGGAAATTATTCAA

ATTTAAGAACAGAAGCTGGTTGCTTTTTCTTTCCCATAATTAATTGAAGCCATAGGGCCC

TATCCATTTATTCATTCGACCCAACTTTTTTTTGTTCCGTTCCAAGAATTCGAACAGGGT

TTTGTACCGATCCGATAAAAATGAAATAAACTCAGAACTATCCGTTGATACGACATGCTA

TTTTTTCCATTCATTCCCTTTCAGGATCAGTCGCGGTCTTCCAAACTTTACCGATGGTAT

GGACGAATTCCTCGCTTCATCCAAATGTGTAAAAGATTCTAGCCGCACTTAAAAGCCGAG

TACTCTACCGTTGAGTTAGCAACCCGAAGAAAATAAAACATAATTTAAATATTCAAATTA

AAGGGTGTGAAGATACAATCTAAATCAATTATCAATTAAATAATGAGAAAATAAATTAGA

CGAGGTAATCAAACAATTGAGCTAAGAAATCTAAAAAAAAAAAAGATTTTCTAATGGATT

AGAAACATAAAAATGAATAGATCAGATCAAAATACAAGAAATTATCAGATAAAAGAAAAA

ATATATATATAGAGAGAGAATTGTCAAAATTGATAGAGTACCTCTTATGTTATCTAC--T

TTTTTTTTC--AATTAAAAACCTCTTGTATCAATATCAAAGAAAGGAATGAGGTAAAGTA

AAAAAACAAACCTATGGTACGGAAATAAATAGATCCACTTCACTGAATTATATTTGTTCG

ATACACTGTTGTCAATATAAATGTTGAGAAAATAATACAATGGAAAAAAACAAAAGAAAT

TTCATTGAAATCTTTTTTTTTTTAACTCAAAAAATTTCAATTTAACAATGAAATAATATT

CCAAATTGTCTTGGATTGACACTAGATATCTAATCTACATAGATACAAAAAAGTGTAGAT

GGGAGAATAAGAATTGAAAAAAAAAA--ATATTGGATTTTTAATGTATTTCATCAATCTA

AGTGGAATAAACAAACTGGATTCCTTGTTGGTTAGTCAAATTCCAACGAAAACCACATAA

TTGAAAGAAATGAAATGTCCGAATTTGAGATTTATATGATCAATAAACTAAGGTTTTGTT

GTTATCGACCATGGAATTCGACAGAAGCAATGAGAAATAGATACGAATAAAGCAGGATTA

AGGGGGGAAATAAAAAAATAGTAGAGAAAAGTTATACAAAGTTATATAAAAATCACTACC

CCCCCTTGGTATTTCTTTAATTGAATTTCGTTTGATTAGGTGGAAGTTCCTTAAAAACCT

CTGCCTTCTTTAAAATATCCTGAACAGTTCCTGTAGGTTGAGCACCCTTTTCAAGGAAAT

ATAGAATAGCAGGAACATTTAAATAAGTTTGATTCTTCAGTGGATCATAAAAACCCACTT

TTCGAAGATCTTTTCCTTCTCTTCGGGATCGAACATCAATTGCAACGATTCGATAGACGG

CTCATTGGGATAGATGTAGATGAACAATACCCCCCCCTAGAAACGTATAGGAAGTTTTCT

CCTCGTACGGCTCGAGAAAAAATGATTTGATTCTAAGTTTTGTCTATGTATGGAATTCTA

ATAAATGACAAATGAGCCTATAAAATAAATTAGACTATGATTTAAGTCTTTTTTT-TTCT

TCTTCCTTCCTGAAAATGAAAAAGAAACCATTCGTACTCATAACTCAAGTTGGATAACTC

TCAAATAGCTTAAAGGAAAAAATCTTTAATAAATTTCATTTATTGAGTGGTCTTTACCCC

CTTTTGTTTGTCTCGTTTAAAATTCTATTTTGATTCTTCAGTCTGATCCAGTTATTGAGA

CTATCGAGACAATTA-AAAGGGTGTTTCCTTGTTCTGGGATCCTTTATCTTTGTTTTAAA

TCATTGGGTTTAGACATTACTTCGGTGCTTCTTAATCCTTTCAAAATGGCAGCAACATAC

CCTTTTTTGTGATTTCTCTTTCTATCAAAGAATCCTACGGACAGTTGATTCCCGCGTGAT

ACACTTTGGATCGAAAACGTTTGATCAATTCCAACAGGTTTTGCTTTTGAATTGGAAACT

TGCTCAAATTGGATCCTTTCCATTTCTATCTCGAAGATATATTTACGAAGTTGTTCCAAT

TTATTGATTGGCATTAACCCTAGATCCTTGCCCCCAAGAAATGAATTAATCCTTTCCACT

CGAGCTCCATCGTGGACTATTTACAACCCAACAAAAAGAAAAAACGAAGGGTTCGAGTGG

AACAGAACAAACGATGTCGAGCCAAGAGCACCTTCATTCCTATATAAATATAAAATAGCG

GATGTAAAAATCCACAACGGATCTGTCCTTCAAGTCGCACGTTGCTTTCTACCACATCGT

TTCAAACGAAGTTTTACCATAACATTCCTCTAATTTGGAACCGGTATGGAATTGATTCAA

TCATGGAATCATGAATAGTCATTGGTTCAGTTGTACATAGACATCGCGTAATCTATACTT

TTTCTTCTGATATGTGTAAAGATTTTTCTGAAGAAGTCTTAGCAAGACACCATCTTTAAC

ATATATATAAAGAAATAGAAATAGATAAACGAATAAATAAGTTCTTTTTGAGTCTGCTAC

TTCAAGTGACTCAATAGGATAGATTGACCTATTTCCTACTTTTTGAGTACCAAAAATTCA

ACTTACAAGGAATCATTCAAAATTTTGATTAAAACTATTTCGAATGGAAAAAGGTTCCTA

TTTAGACATCATTAAAAGATAAGTCTTTTTTTTTTTAATTGACCCATCACTGATTTCAAA

TAGATAAATAGATCACAGAAAAGAAGAAAGAAATCCCA-TTTTTTTTCATGAGATGGATA

AAAAAACTGATGTAAGGTAAGATTTGAATCTTTATTCTTTTCATCTGATTGCGAAAATCC

AAATCGAAAAAATCGAGAGGGGTTTTCGTATCTATCAGATAGACTGAACAATTGTCACTG

TTATAGATATTCTATAACACTTAATTTAACTAATTTTAGTTTAAAAAATTTAAGGGATCC

CAAACCTTTTTCACAAAAACCGAAAGACTATTGTCATTGAAATAGAACGATACATATAAG

CTAAACATTTCCTCATTTTATATGCATTTTGTTTAACATGGGGTTGAGTAATATTTCAAT

AATCGAATCTTGTCATAAAGTGAATAAAAGGGATAGGATAAATCACCCCTGCCTCAATCC

AATCGTTACATAGATTTACAATTCTTCATTATAGCCAAAC-AAAAAAAATACCTAAATAA

AATAAAAAAACGAGATTCAAAGAAAATACATCGATTCCATAACATATAAACATATATAAA

TATGTTATTTGATCATTTGTTAATGAGATTTGGACAGATACAGATATTTAAATTAATACT

GATTATCTCCTATCCACTCCCCTAT-TCTTTCTATGGGAATGATAGAGC-AAAAAAAAAG

GAATCCTGATCCGGTATGGGAAATGACTTGTCTAGTTACAAAGACAAACAATAAGTCCAA

ATTTAGTCAAGCTTTAGTCTAACCCACTAAGAGACTTAGTCCTATTGATTGAATTTAATT

AGTATCAAGAACTCGCTCTTGTTTCGAATTCAGAGATCTCGAGAAAAATCTAATTACTAA

TTAGACTCCCTCATTT--------------------------------------------

------------------------------------------------------------

------------------------------------------------------------

------------------------------------------------------------

------------------------------------------------------------

------------------------------------------------------------

---------------------AATGGATATGGCTCTGGGACGGAAGGATTCGAACCTCCG

AATAGCGGGACCAAAACCCGTTGCCTTACCACTTGGCCACGCCCCATTATCAATTTCTAA

TCTACACTAAGAAACAATAATATTGTTATTGGTTGTTTGTCAACTCCAGTCCAAATATCT

ATAGAATAGATTATTTAGGATTTTAATCCATATAGATATAGAATTCAACTCAATTTATTG

ATCATTACATATAATTCAATTAAGATATTGTATGAAAGTATGATTTCTTCTATTCTCCTT

TGAGAATTGGAGGATTTTTGATTGGGTGGGTTCAAAGAAAAAGAAGGA-TTTTTGTATAC

CTTACTTCCTTTCTTCCTTTTTCCTTATATCAATAACTCAATCAAAATGCAATTATCTCC

AAGAACAAAATGTCTGTTATGCTTAATATCTTTAGTTTGATCTGTATTTGTCTTAATTCT

GCCCTTTATTCGAGTAGTTTTTTCTTCGGCAAATTGCCCGAAGCCTATGCTTTTTTGAAT

CCAATCGTAGATGTTATGCCAGTCATACCTGTGTTTTTTTTTCTCTTAGCCTTTGTTTGG

CAAGCTGCTGTAAGTTTTCGATGAGATCCTTAATAATATCCTAGAAGATTCATGATTTCT

TCGAGAAAAAATTCTCTAACAATTGATAAAACCAGATAAGTTTTAGAGTCTGAACCCTCG

ATTCACACATTGAAATTCTTGGATAGTCGCCATAAATCCGGCTTACCCCATTTCCTCCTT

TTTTTGACCCTTTTCCAGTGAAAGACCCTAACCCTATTTCTATTAGGGTCTCCCACAATA

TCGAATTTGGATATGAAAGAAATTTTTGTTAATGAAAAACTCTTATTCCAAATAAATTTC

TGACAATTCATTTCTATTTCTAGAAAAACCTCATTTCTTGGTGTCAAAATAGGATATGTG

GTAGAAAAATGGAAGATCTATTCTC-AAAAAAATCATCTTGGAGATTGTGTAATGCTTAC

TCTGAAACTCTTCGTTTATACCGTAGTGATATTTTTTGTTTCTCTCTTCATCTTTGGATT

CCTATCTAATGATCCAGGACGTAATCCTGGACGTGAAGAATAAAATCCAAAGGGTTTTTC

CTTGGTTAATTTTCAAATTTTCTTAGGATTTTTTCTATTCCACACGTTTAACTAAGATTT

CAAAAATTTGAAAAATAAATAAATAAATCAAGTCATCAACGGAACCGGAAAGAGAGGGAT

TCGAACCCTCGGTACGAATAACTCGTACAACGGATTAGCAATCCGACGCTTTAGTCCACT

CAGCCATCTCTCCCAATTGAAAAAGAGAATTACTACATTACACATAATGTAAGGAGTCTT

TCTTT-CTCTATTCTATAGAGATATACAAATCAGGAATTTCTTTTAGATTAGATAAAGGA

AGGGCTCGAACGAGCCTATAAATAAAAAAATAAAAATAAAG-AAAAAAAAAGAAGACATC

TTTGTGTTGATTTTGTTCGAAAGGCCCTTCTTATTCTGATGGCCTGGCCTGGTCAGTACC

TAGCCGGGCCCCTTTTTTTGTTCAAACGAATTATAGATAAATAATGATTTATTTGATTTG

AAAACAAAAATGCTTGTTATTTATTATATTCAATTGAATATAAAATATTCAAGCAACAAC

AAAAAGAAGAAAGACTATTTACATTCTTTTTCTTTTTCTATTTGAATTTTAGTTTCATTT

TCGGAACTAAAAAGGAGACTATCGATTTTTCCACAATGCATTTTTCTGTTATGATTTTAG

TGTTTTTTGTGATCCGTAGCTATCAAAACTTCTTAGCAAAAGAGAAAACTTTAGTTATTT

AATTTAAATAAAATGAACCCTCCTTTCAGAATCTCATTAAATTGTAAAC-CCCCCCCACG

AAAAATTTCAACACTCTAATTTTGATGATTCTTTGATGATCCAATCCTATCTTGATCATG

CCCAATTCTCCTGTTCGACAAAAGGTCCATTTGTATACAATAATCGCATTGTAGCGGGTA

TAGTTTAGTGGTAAAAGTGTGATTCGTTCTATTACCCCTTTAATAGTTAAAGGGTCTTTC

GGTCTTATTCATATTCCGATCAAAAACTTTATTTCTTAAAAGGATTTAATCCTTTACCTC

TCAATGAAAAATTCGAGAAAAAAATAAAAATTCTCGTGATTTCTATCCATGAGTCACTTA

GAAAGTGAAAAGTTGGATTATGAAATTGCGAAACATAATTTTTGAATTGGATCAAGACTT

CCAATTAAATAAGTATGAGTAAAGGATCCATGGCTGAAGATAGAAAGTCGATTTCTAATC

GTAACTAAATCTTCCATTTTTGATTTGTAGAGAAAGAAATTGAAGCAAAATAGCTATTAA

ACGATGACTTTGGTTTACTAGAGACATCGACATATTGTTTTAGCTCGGTGGAAACAAAAT

CCTTTTCCTTAAGATCCTCTCAAATAGAAATAGAGAACGAAGTAACTAGAAAGATTGTTA

TAATCACCTTCTTCTAGAAGGATCATCTAGAAAGCGATTCGTTTTGTCTGTATTCAGACA

AAAAGCTGACATAGGTGTTATGGGTAGAATTTTGTTCACATCTTAGATCTAGGAATTTAC

TCATCTTCCATAAAGGAGCCGAATGAAACCAAAGTTTCATGTTCGGTTTTGAATTAGAGA

CGTTCAAAATGCTGAATCGACGTCGACTATAACCCCTAGCCTTCCAAGCTAACGATGCGG

GTTCGATTCCCGCTACCCGCTTATATCCTTTTTCTAAATATTCGATTCGATATATTCTAG

AATATACTCTAATTAGAATTAGTGAATCATTGAATATACAATTCCAAAAAATTTCTCACA

TACAATCCGAT-TTTTTTTAACAAGAGGTAGAAAAGTCAAAATACG-AAAAAATCGGAAT

GAACAGCGTCCATTGTCTAATGGATAGGACAGAGGTCTTCTAAACCTTTGGTATAGGTTC

AAATCCTATTGGACGCAATTTATTTCCATATATTTTTGATTTCGATAGCAAGAAATACTT

T-TTACATAATTTGAATCCGAGACACTTGATTCC-TTTTTTTTTTAAGATAAAAGTGAGC

AATTTCTTTATGTTATGCTTGTTCCTGAAGTAGGAATCGGTCCGTTTGTTCCTGAATAGC

TTCTTTCAAAAGGACTTCTGCTTCCTCGGTAAATGTCTTAGTAGAAGATATGATTTCTTG

GAACTGAGGTTTATTAGTTTTTAAGTAAGTACGTAACTCAACAAGAAATTTCCTTACCTG

TCCAATTTCTAATGAATCAAGATAACCATTTGTTCCGGTATAAATAGTCATTATCTGTTC

TTCTACCGTGAGAGGAGCTGCTTGGGATTGTTTAAGCAATTCACGTAATCGTTGACCTCT

TGCCAATTGATTCTGAGTAGCTTTATCGAGATCCGAAGCAAATTGTGCAAAGGCTTCTAA

TTCTGCGAATTGCGCCAGTTCCAATTTTAATTTACCAGCTACTTGTTTCATGGCTTTAAT

TTGAGCTGCAGACCCTACTCTGGAAACGGAGATACCCACATTAATAGCAGGTCTGATTCC

AGCATTGAATAGATCGGCGGATAAGAATATTTGTCCATCAGTAATGGAAATTACATTAGT

AGGAATATAAGCCGAAACATCTCCCGATTGAGTTTCAACTATTGGTAAGGCGGTCATACT

TCCTTCACCTAAACTAGAACTTGATTTAGCGGCTCTTTCCAAAAGGCGTGAATGCAAATA

AAAAACATCCCCTGGATAAGCTTCACGACCAGGCGGTCTTCGTAATAGAAGAGACATTTG

GCGATAAGCTTGGGCTTGTTTGGAGAGATCATCATAAATGATTAAAGTGTGTTGTTTACG

GTACATAAAATATTCAGCCAAAGCTGCTCCTGTATAAGGAGCAAGGTATTGTAATGTAGC

AGGGGAATCCGCCGTTTCGGCTACCACAATAGTGTATTCCATCGCCCCCCTTTCCTGTAA

AGTAGTTACTACCTGAGCCACAGAAGATGCTTTTTGACCAATAGCTACATAAACACATAT

TACATTTTGACCTTGTTGATTGAGAATCGTATCTGTGGCTACTGCTGTTTTACCGGTCTG

TCTGTCCCCAATAATTAATTCTCGCTGACCACGTCCTATAGGGATCATCGAATCAATAGC

AATAAGCCCGGTTTGAAGAGGCTCATATACGGAACGCCGCGAAATAATACCTGGAGCGGG

AGATTCAATTAATCGAGATTCCGAAGCTGAAATTTCACCTCTACCATCAATAGGTTTAGC

CAGGGCATTTATAACACGACCCAAATAGGCCTCGCTCACTGGTATCTGAGCAATTCTTCC

TGTTGCTTTTACAGAACTTCCTTCTTGTATCATTAAACCATCACCCATTAATACAACACC

AACATTATTTGATTCCAAATTCAAAGCAATACCTATTGTACCTTCTTCAAATTCTACTAA

TTCACCCGCCATTACTTCATCAAGACCATGAATACGAGCAATGCCATCGCCTACTTGAAG

TACGGTACCAGTATTTACAATCTTTACTTCTCTATTATATTGTTCAATACGTTCACGGAT

AATATTACTAATTTCGTCGGCTCGAATGGTTACCATGAGTATTTCTTAATTATTTTTTAT

TTTGAAAAGAAAAAAAAATAATGCCTATAGTAGAAAGACTAATCAGTTATTTCTTTCATC

GCTCCCAACATGCCAATATTAGCACTAATGGTACGTAAATGTAACTCGTTGTTTAAACAA

CTATTCAGAGTTCCTAAAGCTCCTTGTAAGGCTTGTTGGAAAACCCGTTGTCGGACTTGA

TTAATCGCCCTTTGCTGTTCAAACTGAATCGTTTCATTTTTGTAATTTTCTAATTGTTCC

AAAGTCTTAGAAGTTGAATTAATCAAATTCCATTTTTCTCGCTCTATCTCAGAGTATCCA

TTCACCCGAAACTGATCTGCTTCCATTTCCACTTTCCGTAAGCGAGCCCGGGCTTTTTCC

AGCTGTTCAATGGCCCCCCCACGCAGTTCTTCTGAATTTCGAATAGTATTCAAGATCCTC

TGTTTTCGATTATCTAATAAATCACTTAATGAAAGTAGATTATCTTTCCATTCATTTCAA

AACTTCCATAATCCCTTCCCGAACCAAACATGAATCTTTCGATTCATTTGGCTCTCACGC

TCAATTACTTAGGGTAAATTCTCATA--TTTTTTTTATGAATGTAATGAGCCTATCCTCT

CTTCTTTATTCATATT-AAAAAAAACTTAATCTAATGCAAAACCAAAATACTCGGAGGAC

TCTTCTGACAAAATAAAAAATATGTAATTGTCAGCAAAGTTGTTTCTTTTTTTTCTTCAC

TTCAAATCCAAAAAATTCGTCTGACTTATACATAAGACATAGGTCGTCGATTCAGCATTG

GATAAAAGAGGGAAAATGCCCATTTTAGAATAAGTGGTTCAAATCATTTTATCGAGATGA

GTGTTCTATATCGATAAAATATTCATTTGAAAACCATCACTATATTAACATAGTGGTAGA

AAGAGTACCATGCCGCATCTAGACTTCAAACGGTTTGCTTTAACCATCTTAACAGTCCCA

CATTATTGGTTCCTAGAGAATCAAAGTAGATTTGCCAATTAATCACGAAATGCTATGGTT

CTTACATATAATTTATTAATTTCTTCAGAAGTAATTCGCGAGATCATGCACCTCTCTTTC

CTAGTTATAAAGGAAAAAGGTCCAGCTGGTTGGATCCAGCCTATTCTTGAAATAAACAAC

TCACACACACTCCCTTTCCAAAAAAGATCAATACACCAATCACTACACTTAGATTTATTG

GATTTGTTGCTAAAATATCGGTATTAAATCCGAAACTCCCGGCAGATGGCCAGTGGCCCA

AAGAAACGAAAGAATCGGTTACATTTTTCATAGGATCTCCTCTTATAGATAGACTCAAAA

ATAGATCAGAGTTCTTTTTGTATCACTTTGCCTATTTATTCTTTTCTATCGAGTCAAAAA

ATATTCGAGTTATAAAGTATGAACTACCCCCTTGCTGTTGCAACACCTCCT--AAAAAAA

AAGAGTTTCCCTTGGACTACGAACGGGAAAGGATGAAAGCGAGTCGGTATGCTAATTCCT

CATCTGCAAATCAGCCCTTCCCGTAGGGTATTTTCTCAACGAATAAGTAATTGTAGGAGT

TAAATCTTGATATAATTTGAAAAAGCAAACAAGAGGTCCAAGGCAATAAAATATGAAAAA

TATGTATTTTTCATATTTCTAAGATTAAACAAAAGGATTTGCAAATAAAAGTGCTAATGC

TACAACCAATCCATAAATTGTTAAAGCTTCCATAAAAGCTAGACTAAGCAATAGAGTACC

TCGTATTTTTCCCTCTGCCTCGGGCTGTCTCGCGATACCCTCTACAGCTTGACCCGCAGC

AGTCCCTTGACCAACTCCAGGTCCAATAGAAGCAAGCCCTACGGCCAATCCAGCAGCAAT

AACAGAAGCGGCAGAAATCAGTGGATTCATGATAAGTTCCTCGTACCAAAAAAAAAGAAA

TGGTTAATGATACAATCAACCAATGAATTATGACTTAATTATTCCATCGGTAGGATTCAT

CCAGTCGAAGTAACTAACAACTTCGAATTGAAGTAATAATATTATTGAATCATCAGAACT

ATTTCGATATCTCTATTTTTCGTTTCTATCCACAGAATCTTTGTGAATCCATA-GACTTT

CGTCCTTGCATTTCTTGGTTCCGAACTGTTATTTCAATTCTTCCATCTTTTTCTTGATTT

CATCCCCTTATTCAGTCAATTCACAGTCACAACGAGATGGAAGGACTTCTATTGGAATCC

ACATACAGTAGTAGAGAAAATGAAATATATCTTTAGTTCATATATAACTAGTCAATATCT

AATATCACATATACATGTCTTTCTTCCCTAACGTAAACCAAGCATTCATCTTAGATTCAA

TCGAATTCGAGAATCAATCGTCGAAACATCTACAAGAGTTGGCTTAGAACCATTCAAATA

CATATATCTAGTTCGACTCCCCTCTTCGAATCAATAATTAATCCATTTTTCAGGAATCCC

GTTTTTTGTAAATTATTTTCTCCCTTCCACTTTCTTTATCATTATTCCACCCGAATACAA

TCTAAATGAGCAAATTAATTAGGGCGAATTATTGCATATGCATAGAAATATCATTATTTG

ATTGATCTAAGTTCATGCAATTTATTATTTATTAATATTTTCTTTTGGTCAATTGTTGAA

TAAAATCAACTGAAAGTAGAATAGTTTCTCGTGTTTTTTATTTAATTACACGTGGTAAAC

ATATATCTTATTCCAATTTTGATTTGAATAACAAGATTGGCTGACCAATATAAAGCGAAT

ATTCGTTGAGGGAAGGTATGATAGTAAGTGGACGAAAAGGGAATTTTAGGAAAAAGATCT

AGATCTCTTTCCCCTTT-TTTGTTCCTATTACTTTCTATTTTCTATCGTATATAGCGTCG

TTGTATATTTCTATTCCTTAAGTCAATTATCTTGAGCCACACATACATTGCTTTGCATTA

AGCT--AAAAAAAGACTATTTCAATGATGGCCCTCCATAGATTCACCTATATAAGCCGCG

GCTAAAGTTGCAAAAATAAGAGCTTGAATACCACTTGTAAATAATCCAAGGAACATGACA

GGGATAGGAACCACTGAAGGTACTAAAGAAACAAGAACAACAACTACTAATTCATCCGCT

AAGATATTCCCGAAAAGTCGAAAACTAAGTGATAAGGGCTTTGTGAAATCTTCTAAGATG

TTAATGGGTAAAAGGATTGGAGTTGGTTGAATATATTTCCCGAAATAACTTAATCCTTTT

TTGGTAAGACCCGCATAGAAATATGCCACTGACGTGAGTAAAGCCAAAGCAACGGTAGTA

TTTATATCATTCGTGGGTGCGGCTAACTCTCCATGAGGTAATTGTATGATTTTCCAAGGT

AAAAGAGCTCCTGACCAATTAGAAACAAAAATAAATAGAAACATAGTTCCAATAAAAGGA

ACCCAAGGGCCATATTCTTCTCCAATTTGAGTTTTACTCACATCTCGAATGAATTCAAGA

ACATATTCGAAGAAATTCTGACCCCCAGTCGGAATGGTTTGTGGGTTCCGAACAGCTATA

GTAGCTGAACCTAATAAGATAGCAATTACAACCCAAGAAGTAATAAGTACTTGGCCATGG

ACTTGGAAACCCCCTATTTGCCAATAGAAATGTTGGCCTACTTCCACAGCGGATATATCG

TATAACCCTTTTAGTGTGTTGATGGAACATGATAACACATTCATATTGCCCTCTG-AAAA

AAATCGAACTTTAAACAAAATTATTTTGATTCCACCATCTCTTTGCCTACTTGAATCGGA

TATTTGAAATACCAACTAATATTTGGAATACTAACTAATCACATAATATCCCCAGTTATT

TTTATCTCTTTTTAATTCAGAAATAATAACCGATTCCATAAATCTATCGGATTTTTGAAG

TCAATTATTTATCTTATTATTAATCAAGGATTTCTTATATAGCTAGAACGACCCTCACAA

ATTGCGAATACTAATTTGTTAAGAATTAAGCGGATTGAAGATATAGCGTCATCATTCGCT

GGAATCGAAATATCTGCAAGATCAGGGTCACAATTTGTATCGATTAAACAAATTGTTGGA

ATTCCCAAAGTGATACACTCTCGCAGGGCCGTATATTCTTCGTGCTGATCAACGATAATT

ACAATATCGGGTAACCCTGTCATATATTTAATCCCGCCCAGATATGTTTGCAAGCGAGAT

AATTGCCTTTTCAACATAGCTGCATCCCTTTTCGGAAGACGGTTGAGTCTTCCTGTTTTT

TGTTCCATTCTCAAGTCCCTGAACTTATGAAGTCTCGTTTCTGTAGTGGACCAATTCGTT

AACATACCGCCGAGCCATTTTTTATTAACATAATGACACCGGGCCTTTATCGCAGCCCAT

GCTACTGAATTAGCTGCTTTATTTTTGGTACCAACAATTAAGAATTGTTTTCCCCTACTT

GCTGCATCAAAAACCAAATCACAAGCTTCGGATAAGAAACGAGCAGTTCTAGTAAGATTT

GTAATATGAATACCTTTACGCTTTGCAGAGATATAAGGTGCCATTTTAGGATTCCATTTC

CTAGTACCATGGCCAAAATGAACTCCTGCCTCCATCATCTCTTCCAAATTGATGTTCCAA

TATCTTCTTGTCATTT--CCCCCCCCAAAAAAAAAAGAAAGGAGGAACTCTGAACTGAAA

TAAATAATTGTTCCGATGGAACCTTCTCTTCTACCGTAGATTGGCCGTAGATAGACGAAC

AAGCCATTACTTTTCTATTCATTATTATTTTGATTACCAAATCAAATGACTGCACCAAAT

ACAGATAGTCAAAAAGATGAATCTGCTCTTAGGAATCATTAAATCCTATAAATGATTGTT

CTGATGTATCATGGAAATTCTTTGAAAGACAAGAATCAAATAATTTTCTGTGGTGAAACA

AAATATCTCTCATTTCCCCCTCGAATAGATTGTTTTTTTTTGTTTCCAAAAGACTGTTGT

TATCTTGTTTTGAGGGGGGCACTAATCCTTTAAATCCGGTACCAAGGGGTATCATACCCC

CCAAAACAACGTTCTCTTTCAGGCCTTTCAACCAATCGATCCGACCCCGGAGAGCTGCTT

TTGCTAAAACTCGAGCAGTTTCTTGAAAACTCGCTTCAGATATGAAACTTTGAGTATTGA

GAGATGCTCTTGTTATTCCCAATAAGACGGCTCGGTAACAGACCGCTTCTTCCAAAGCGC

GCCCCATTCGTTCCGCTCGCAATAATCCAATTAATTCTCCGGGTGAAAAAACATTAGACA

TTCCATCTTCTGAAACCAACACTTTTGATGTTATTTGGCGTACAATAATTTCTATATGCC

TATTATGAATCTGCACCCCCTGGGATCGATAAACCTTTTGGATCTTATTAACCAAAGAGA

TACGACTTTGCACTATAGTTAGCTCAGCACCAATCAAGAAGCCCCACGGCATTCCAAGAA

TTCTTGTTATACGTTCGTTCCAACCCTCAACCCTCTTTTCTAGATTCATCGATATTGAAT

CAACCGAACGCACTTCTAACACCTGTTCCACTTTTGGAAGCCCCTGGGTTATATCACCAG

ATCTCGATTTTTCATATATAAATGTAATTAATGTATCTCCTTCGTACAGGATTTCCCCAT

AATGGCCATGAACAGTTGCTCCTGGAGTGGCCAAATAAGGCTTAGCTGATCGTATTACTA

CAGAATCAACTTGAACAAGTATAACTTGACCCGATTTTAGGTGTGGTGCATTTTTGGCTA

TACATACATTTTCACAAATAAACTGTCCAAGACTAATTATTGTAGATGTTTCTTGACAAT

AATTGTCATGGAGAAAATACCAATTCAAATTGAATGGATTCAAAATAATGTTACTGCCTG

GCTCGGGGTTATAAATTTTATCATTTTCATCCATTAAATAATATTTAATTACTTGAAAAG

TCTGTTTTAAATTGTCAAGTTGCAAATAGTTATTTACCAAGATCTGATTATGAGTTATTA

AATGGTAAAATGAATAAAAATTCACAATTGTAAAGGCTGTTCCAAAAGGCCCCAGCGAAT

TCTTAATTGGAATTCGAGGATCCTTTGTAATTTGAATTGATTCTTTTATCACATTGTGAT

ATTTTATTACATCGTTGAATGGACCCATTCGAAAACAATTGGATGATGACAAAATTATCA

ACGATTGGAATCCCGTATTTCTATTCAACAACGTATGAATAGTTCCTTGATTTTGGTTAA

GGGATTGTTGAATTCTTGCCTTGGAATAAATGGAAGAAAACGGATTGATATTGGTGCAAT

CTGATCCATTATCAGAGAGCAATCCTGAACCCGATGGATCATTCCTTTTTCCGATATATG

AAATAGGGGATTTCACTAAGTCGATTCTTAGGAAATGTCGAATCAAACCATTTGTCCTTA

TTTCAACAAAGGAAGCACGGGCTTCTTCACTAGAAGAACTTTTTTTTTTGTCTTGGTCCC

AATTCAATACTAAACAAGTCCGAACTAATTGAATATCTGTATCAGAAATTCCTCGAATGG

GTTTGCCATTTCCATAAAGGATATAATTGACAACTCGAAGTTGCACATTATCCCTTTCCT

GCAACAGATCCGGGGGAAAAAGTGTTGCTAAATTTATACCGTCCGTTATTTCATATGTGA

CGACAAGTCGAACCAAAACAAAATACTTTTTCTTACTAGGTGTGATCCGTTGAACATAGA

TCCAATTTTTCCATTTTTTTGATTCCTTGGAATTTCGTTTTCCTGTTCCTGGTGGTATCA

AAACGCCGCTATGTCGAGATATCTTATCGGTCTCTCCAGGAAAATGGATATCTCCAGAAA

AGATTTTAAGTTCTATTCTTTTTTTTTTTCTCTCCACTCGGACAAACCCGCCTACTCGGC

TTCTTATATTTAAAGTAATTTGTGTATCTACCCCAATGAGACTATTGTTCCGTACCATTA

TGGAAGAAGATCCGGGTAAGATATGCACTTCCTCGGGAATAAAAAAAAACCGATCTACTT

TCATTTGGTATTTTGGCCTAAATGCCTTGACTCCTCGATACTCAATCAAATCCTCTTTTT

TGACGATTGAATGCATTTCTACAGTCCCATATTTAGTAATGCCCGAACTCTTTCTTCTGT

ATCGAGGATCATCAAAATAAGCAAGAATACTATTTCTACGGAAAATGCCATTTGGAGGGA

TTTCAATCGAGATACCTGAACAGGGCATTAGTTCGTTCTCACGTTCTTGAATCGATTGGA

GTGGAATGATGAATCTATTTCTCCGCCTCTTTGAGAATAAATCAGAATTCTGGTGGAGAA

TGGTCGGATATATGAGATTACAACGACCATTACATCTAATTCGGTTAAGGTCCGAATAAT

CAGAAATCCTATCTTCTTTTTTTTTTTTACCATAAAAATACGAACTAAAGACTTTTTGTC

TCTCCGGATCATTAGTTTCTGAGAGGTTAGAAGTATATCTTCGCTTGACAGAACGAGAAT

GCGCGTTTATTTGATCTTGATCCTTGTGGAGCGAAAGTGAGACTAGACTGGATCTGCACG

GCCCTCCTAATAATATCCATAAATGACTTGTTTTTGGTAATAGATGAACATTACCATATG

TAAATTCAGGTGGATGATACACATCAGTACTCCAGTGCATTTCTCCGTCTGAGTCAGAAT

AAATATGTTTTCGAACCTTCTCTTTAAAATTCAAAGTGGATGTTCCTGCGCGAATCTCAG

CAATCACTTGTTCTGATTCTACATATTGATCGTTTTGAACTAAAAGAAAACTTTTGGATG

GAATATTTACATTATGTAGAATATCTTCACTCTCAATAGTTACATACAAGTCTATAGAAC

ATAGAAAGGCGGGATGTCCATGACGTGTACGTGTCGGATGAACCAAATCCTCATTGAATT

TGATTTTTCCATTAGAAGGGGCCCGCACATGTTCTGCAGTACCCCCCGTGAATACTCCGC

CGGTATGAAAAGTTCTTAATGTTAATTGAGTACCCGGTTCTCCAATCGATTGTCCTGCAA

TAATACCTACAGCTTCTCCTAATTCAACCAGGTCGCCATGAGTAGGACTCCGACCATAAC

ATAATCGACAGATCCAAGACGCACTCCTACAAGTAAAGGGAGTTCGAATAGATATTGGTT

GTGCTCGAAAGGTTATGAATCGATTTACAAGTCCAATCCCAATGTCTTGATTTCTACTGG

CAATACATCGTGTGCCCGCATATATATCATCTGCTAATACACGACCCATTAATGTTTGGA

TCAAAATCCTTTCCGGCATCATCCCATTCCGAGGACTCACAGAAATACCCCGGATGGTGC

CACAATCTGTCCGACGTACAACAATGTGTTGAACTACTTCAACAAGTCTGCGCGTGAGAT

ATCCAGCATCGGATGTTCGTACAGCAGTATCCACAACCCCTTTACGGGCTCCGTAGCAAG

AAATGATATATTCTGTTAAAGAGAGTCCTTCGCGTAAATTGCTTTGAATGGGTAAATCAA

TCATTTGTCCTTGAGGGTCCGACATTAAGCCTCTCATACCTACTAATTGATGTACCTGAG

ATGCATTTCCTCTAGCTCCCGAAAAAGACATTATATGAACTGGATTAAAGGGGTCAGTCA

TCCTAAAATTAGGATTCATTTCTTGTCGCAAATATTCACTTGTAGCATACCATATTTCAA

TGGATTGGCGTAATTTTTCTACCGCGTGTACATTCCCATAATGATGGTGTTTTTCCAAAA

TCAAACTTTGTTGTTCAGCATCTTGAACTAGCCATCGCTTAGAAGGTATTGTTAAAAGAT

CATCAATTCCTAATGAAATGGATGTAGCAGTAGCTTGGTGGAAACCCAGAGTCTTTACTT

GATCCAGGATGTGTGATGTATATGCCATTCCGAAGTGATCTATTAATCTACTAATAAGTC

GTTTCATGGCAGTTCCATCTATCACTTTATTGTGAAATACCAGATTGGCCCGTTCTGCCA

TAAGTACCTCCATATTCTGCTGAGTACGATTCGACAATGGGTTTGAGTCAGTGATTGGAA

AACTTCCTTTTCTCGATCTTGATTCGCATAGAAATTCCGGAACTATGGGCCTAGTTGAAC

TGAAGAAACCCGAATTCCTACTGGTATTATAGAATTCCTTAACTTAGTTCGGTACCATAT

GAACAGGCCTGAGAAAACCCCTGTACGGCTTCTTCGATTTCTCGATAAAGAGAAATATGA

CCAACAGTGGTTCGGATGTATATAAAAAGAATTTCTTTTTTTATACTTCTTACTATTAGA

TAGTGTCCATAAATCTCATAATAAGTACCTAAAGATTCATAGTGAACTTCGATGGGACTT

TCTCTTGAAGCAATAACGCGTTGATCTAGTCGCCACCGGAGCCACAAAGGACTATCTAAA

TTGATTCGTTTCTGCCGATAAGCTCCAATTGCATCATAGGAATTAGAAAAAAAGGGTTCT

TTCGTATACTTATAGTTATTATTGTTACTTCTTTGATTTTGATAGTTTCTGCGATTCCAC

GGATTATACCTATTTACACAAATACCTCGACGATTCCCGCTCGTTAATACATAGAGTCCA

ATAAGCATATCTTGAGTTGGTATGGAAATGGGATCCCCAATAGCTGGAGACAAAAGATTC

ATATGAGAAAACATAAGTAAACGGGCCTCCGCTTGAGCCTCCAAAGATAAAGGTACATGA

ACAGCCATTTGATCCCCATCAAAATCTGCATTGAATCCCTTACAAACTAATGGATGTAAA

CAAATAGCACGCCCCTCCACTAAAACGGGCTGGAATGCCTGTATGCCTAATCTATGCAGA

GTGGGTGCTCTATTCAGCAATACAGGATGCCCCTGCATAACTTCCTGAAGTATTTCCCAT

ACAATCGGTTCTTTTTCCCGAATTTTACTCTTAGCAACTCCTATATTCGAAGCAAGATGT

TGTCTAATTAGACCACGAATTACAAATGTCTGGAAAAGCTCTATTGCTATTTCGCGCGGC

AATCCACATCGATGTAATGAAAGTGAAGGACCCACGACAATGACGGAACGACCTGAATAA

TCAACCCGTTTCCCCAGCAGAGTCTCACGAAATCTTCCCTCTTTGCCTTCAATTACATCT

GAAAATGACTTGTAAACTTTATTATGACCGTCCCTCATTGGTTGTCCACGGATTCCATTA

TCAAGAAGTGTATCCACGGCTTCTTGTACTAATTTCTCCTGACACATTACTAATTCTCCT

GGCGTGGATTTACTTGTTGTTAATAGATCGGTAAGAGTATTGTTCCGATAGATAACTCTT

CTATAGAGTTCATTAATATCCGAGCTCATTAGTTTACCCCCATCTATCTGAATGATGGGT

CTCAACTCAGGAGGAAGAACTGGTAATAAACACAAAACCATCCATTCTGGTTCTATATTT

GTTCGAATAAAATGCTTAGCCAATTCCATGCGTCTAACTAAAAAATCCTTTCTTCTTCCA

ACCTTTCGATCTTCCCATTCATTTCCTGTGGGTCCTTCTTCCCCTAATTCTTTCCATTCT

ACCAACGAATTATCTAGAATAATTCGTAAATCTAGATCGGCTAATTGTTCTCGGATAGCA

CCTGCACCAGTAGAGATTTCTCGATTGCGAAATGTATCGAAGCCTTGGGTAGTAAAAAAC

AGCGGGATGCTGTATTTCCAAGATTGAATTTCATATTCGAATAAACCTCGTAATCGTAAG

AAAGTGGGTTTTTTAGTTATGGGCCTAGCAAAAGAAAAATTGGGATAGGATCCTATAGGA

TCTCCCCCCTTCAAAATCGGACGTGAAAGTTTCCTTTCATCCGGCTCAAGTAGGTACACC

AAATAAAGAAAGGAGTTCTCGCTTGCAAATTCTAGAAAACCCCAAAACAAAAAGATCTAC

TCCTTACTCAAGTTCCCAGTGAAAACCAAGCAAGATTTCATTGATTCCGCCTTCTTTTTA

TTTAGATTTTCTTAATTCGTTATTCAATTACGACATAAATGAAATGTGAAATTCTTGAGT

AGTCTACTTCCCTTCGAATGATGAATCCCTTAATTCTTAATTTAAGAA-TTAAAGTGAAA

GTAAAGGAGTACCTTGGAATTCATAAGGGATTTACTTGTCTATGTATTGTTCCATTCGAT

CTTTTAGGTCCCGACTTCACCTCGACGGTTATGCTACGATGCCCTTAAAGCCTATACGCG

ATGGATAGACTCCTGTAACCATGACATATTTGCGCTTACTTGAACATAATTTCTTTCCAA

AAGAAAGGTTAATTCCACAAAACAAAAAAGTTTTTTTACGAGGTACAAATATTTGTATTT

ATTTGTTACGAAATCGACCATAGATCAATTCCCCTTTTGATTTGGGAGTATTGACTACAC

CCCTAATTCTGAGCTTCATGTTACTCCTGCCAAGTGACATGTCAGGTCCAGGGCATCCCA

ATTGGATTGACTGGGATGACAGTTTATCATTCCGAATCTGTAAAATCATAATTTGGATCA

AATCACACATCGCAGTATACTAGGCCTTCTAATTCTTTAAGAGGTTTATCTAAAAGATTC

GCAATATAACTAGGAAGACGTTTTAAATACCACACATGGGTTACTGGGCATGCGAGTTTG

ATGTAGCCCATTTGATATCTTCGTATGCGAGAATCGACAAATTCGACTCCGCATTGTTCA

CAAAATTTCGGGTCTTCTTTTTCATCTCCGATTATTCGATAATTTCCACAAGCACAAATT

CCACTTTTTATAGGCCCAAAAATTCTTTCACAAAATAATCCATCTTTTTCAGGTTTATTG

GTTTTGTAATGAAAAGTATAGGGTTTTGTCACCTCTCCAACGATCTCTCCATTAGGCAGG

ATTTTAGTGGCCCAAGCACTTATTTGTTGAGGAGAAACTAATCCAATTCGTAGCTGTTGA

TGTTTATACCGATCGATCATAGAAGAAAAATTCTGATTCATTCCGATTAAGCTTCCTTCC

TATTAATCTGGAAGTTTTTCTCAGATACAAGGAAATGGTTCAGTTCCAGAGCTAAAGATC

GTAATTCTCGAACGAGCAATCGAAAAGATTCTGGAGCATCCTCAGGATTAGGTATTGTTC

CTCCAATGATCGTAGTACCAAGTACTTCTTGGCGCGCTCTAATATGATCAGATTTATAAG

TAAGCATCTCTTGTAAAATATGAGCAACACCAAATCCTTCTAGAGCCCAAACCTCCATTT

CTCCTACTCGTTGCCCCCCTTGTTTTGCCCTTCCTCTAAGGGGTTGTTGTGTAACAAGCG

CATAATGTCCACTGGAACGTCCATGGATTTTATCATCAACTTGATGAATTAATTTCAAGA

TATAAGACTTTCCTATTAGAACGGGTTGTTCAAAAGGATTCCCCGACCTTCCATCAAATA

TTCTGCTTTTTCCTGGATACTCGGGTTCAAATACCCATGGATTCGCTGTTTGCTTACTGG

CTTCATATAATTCAGAAAACACTAGTTTTCTCGAAGCTTCTTGTTCATATCTCTCATCAA

AAGGCGCTATTCGATAATGTCTGTCTAGCAGACCCCCTGCTAACCCGAGCGAACATTCAA

ATATCTGTCCTACATTCATTCGTGAAGGTACTCCTAATGGGTTGAAAACCATATCAACAG

GTCTTCCATCTTGCAGATAAGGCATATCTTGTCTAGGCAAAATTTTGGAAATGATACCTT

TATTTCCGTGTCTTCCAGCTACTTTGTCGCCTACTTTAATTTCACGTTTCTGTGAAATAT

ATATACGAATCGTTTCTGGATTATAACTAGAACCCCCCCTTTTCTGGATCCACCTCACAT

CAATAACCCGGCCCCTACCACCTATAGGTAGTTTTAAACAAGTTTCTTTTGAAGTAGATA

CCTGAATGCCAAGTATGGCTCGTAACAATCTATCTTCGGGGGCATACGACGATTCTTTCA

CCATTTGGGGCGTTAATTTACCTACTAAAATATCACCCGTCTCTACCCAAGATCCCAGCA

TCACAATTCCATTTTTGTCTAAATTGCGGAGTAAATGGGCTTCTAAATGTGGTATTTCGT

TAGTGATCCTTTCAGGGCCTTGGCTTGTCACATGAATCTGAATCTCATATTTCCGTATAT

GAAAAGAAGTATAAATATCTTCATATACCAAACGCTCACTAATGAGTACTGCATCTTCAG

AATTGTAACCTTCCCATGGCATATAAGCTACTAATACGTTTTTCCCCAAAGCAAGTTCGC

CACCAACCGTAGCAGCACCATCTGCTAAAATTTGTCCCTTTTTAATACATTTACCCCGCT

GAACCTGGGGTTTTTGATGCATACAAGTATTTTTGTTGGAACGTTGATACATAACTAATG

GAATGCTTAGAGTATCTCCATTACCTGAGAAAAGGATCTTTTCAGTATCGGTATAAATGA

TCTTTCCCTCACGTTCGGCTATAGCAAGAGCCCCTGAATCTAGAGCTGCTTGTCGTTCCA

ACCCAGTTCCAACAATGCATTTCTCGGGCCGAGAAAGCGGAACTGCTTGACGTTGCATAT

TAGAACTCATTAAAGCCCTATTCGCATCATTATGTTCGATAAAAGGAATGAGGGAAGCTC

CAATAGAAAAATATTGGAAGGGAAAAATACTTCGAAGATGAACCTGTTCCCATGCAATAG

TCAGGAATTCTTGACGGTATCGAGCTGGAACAACCTGTTCTTCCTGAATATCCTGATTCA

ACGCCAAAGAATTTCCTGCCGCTACCATATAGTATTCATCTCTACCTGGTGATAAATAAA

GCATCCGTACCCCTGTTGATCTTTCAGAAATTTCATAAAATGGGCTTTCTAGAGATCCCC

AATGACCAATCCTCGCATGAATTGCTAAGGATCCAATAAGTCCAACATTGATTCCCTCAG

ATGTGTCAATTGGGCAAATACGCCCATAGTGACTAGGATGGATATCTCGTATCCGAAAAC

TAGCAGTTCGCCCTGTTAGTCCTCCAGGGCCTAAATAACTCAATTTTCTCCCATGAACTA

TTTGTGTCAATGGATTAGTTCGATCCAAAACTTGAGATAAGGGGTGTAAACCGAAAAAAG

ATTCATAAGTGGTTGTTAATGGAGTTGAAGTTACTAAATTCTGAGGAGTCGGTATCAATT

TGTGCCGAATTGCTCCACATATAGTTCCTCGAACCACATTTTCTAAACGAACCAGAGCCA

ATCCGAATTGATCTTGTAAAAGATCCGCTACAGAACGAATACGTTTATTTTTCAAATGAT

TCATATCGTCAAGTGTACCCATTCCAAATTTCAGTTCAATCAAATGATCAGCGGCTGCCA

ATATGTCTCGTGGTAACAAAAATGTATTGTTCTGGGGTATATCAAGGTTCAGTCTTCGGT

TCATATTTCGTCGACCAATCCTTCCTAATTCACATCTTTGTTGAAAGAACTTCTTTTGTA

ATTCCTTACATAAGGATTCAGAAAATACCGGATCCCCACCTACACAAGCAAATTGTTGAT

AAAACTCCAAAATGGCATTTTCTTTTGATCCAATTTTTTTTCTCTCCTTATCATTCAGAA

AAGACAAAAAAATTTCAGGATAACAAACATTGTCTAGAATTTCTCTTAGATTCGAACCCA

TAGCTGATGATAGAACTAGAATAGATATTTTTTGTTTCCTACTCACACGAGCCCATATCC

TTGCTTTTCTATCAATCTCTAATTCTGATCTTCCTCCCCAATCTGATATTATGGTGCCGG

TATAGACCGAAATTCCGTTATGGTCCAATTCTGATCGGTAATAAATACCGGGGCTTTGCA

ATATTTGATTGATCACAATTCTATATATTCCATTTACTATAGAAGTTCCCAGGGAATTCA

TTAGAGGAATGTTTCCAATAAAAATGGTTTGTTCTTGCATATCCCTACTAGTTTTCCAAA

TTAATCCCCCGGATACATATAATTCAGAAGAATATGTGAGTGATTCATACACAGCATCTC

TTTCTTTTATCAACGGTTCTACCAATTGATATCTTTCCACAAATAATTGAAATTCTATTT

CTTGATCTGTATCTTCAATTTTTGGAAACTTATAAAGTTCTTCTGTCAAACCCTGATTAA

TGAACCTATAAAATCCTTCAAATTGTATCTGATTAAATCCAGGTATTGTAGACATTCCCT

CATTTCCATCCCCGAGCATTTTGAATTTCCCATTTCTCAAAAAATCCCATT-ATTGGTCC

ATTCTTCATCGAATTAGCGAATTAGATAGATGATCTAGCAATGATGGAATTTCTATTCTG

TTTACTGAATCACATGAAATTTTACCCAACTCCATATTTGTAATGGAATATATGAACTAC

GTATGAACGGAGGAATAAAGAGAATTTTCTACTGGAATTTGCAACAGATCCAAAGGAATT

GATAAAACATTCCTAGAAACAGAATTCTGCTACTTAGACTTATTAAGGTATAGGGTTTTG

TATAGAATAGAAAAACCAAAATGATTGCAATTCTACCATTATTATGATATTACATATTCC

AATTTGAGAAAAATAAAAGGATTCGGCATTTGATCTTTTCACTGAGATAAAGACACAAAA

ATCAGAAACAATATACGATTTTTTTAGCACTTAACCACCTTTATGTTATGGATTTCATTG

TTCAAAAAATGATTCGCAGAGAAAATAGATATTTTGACTTTACTGATTTTTGAGTAGATA

TAGGATTTAACTGTATAAGAAGGGTTGCATTTATTAAACACGTGTGCTGTGCAGATAGCT

ATAATATCCGACTTTTCTTTTTTTGCCGTTCTATTCGGAACAGCCGGGGTCGTGCTCTAT

CAAAACGAAATTTCTATTCAATGCAAAATGGAAGTATGATAATTTTGATAATTCCCTATC

GACAACATATCTAAATAATAGATATCTGTGTAACAATTTCTGTTCTGGGGTTTACATATA

CTCATCTATTTTGTTATAATAGAAATTGAGAAGGATTTTTTGATTGAAAAAATCAATACT

GATTAGTTCTGTCTCAATTTGTATTTTCTAATGTCATTAGGAAAACACAATTTGGAGATT

CAAATCCCAGAATTGTTCATGAATTCGAAGTAAGCAGTCAATAGTTAATGCTTTAAATTT

GTCCTATATTTTGGCTAAAAATCCACATTTTATTTCTCAATAGAAAAATGAGATATTTTT

TAGCATTGTGGATTTTCAGATACTATACAATCAATCGAAGGGATGGATCAAATCCAATCA

AAAAAAGGAGGTTTCTTTTAGGAAAAGGATTAAGAAAAATAGGACTCAAAATGCAAGTAC

AATAAAAATTCCGTAATCCAGGAAAATTATTATATCCATTTTGTATCATTTTGGCGGCAT

GGCCGAGTGGTAAGGCGGAGGACTGCAAATCCTTTTTCCCCAGTTCAAATCCGGGTGTCG

CCTGATCAACAAAAAGCTCTAAATCTTTTCTTCTCTTCTGTTCTGTTGATACTTGTTTGA

TTCTAAGCATCTGGTCTGAGGGTTTTCTAAAAGATTGTGAATCCTTGCATCTAGGATTCA

AGGAAATATTGTAATCTAATGATAGAGGGGCTGTCAAGACTTCACGATTCCCTTCTATTA

CTAAGGTAGTGTCGAATGACTGGATCTTGACTTAGATTGGAGAGCCTGATAGGAAATCTG

ATTCATGTGGAAAGGGGCGGAAGTTGCTTTATTTTATATACGACGGACTCGCGAGAATCT

TGGAGTGCTCAGGTATTCAATCAATATTAGATTAGATGGATAATTGACTTTTATAGAAAA

AGGGGCAAAACCAAATTCTTTCTTTTCGGCAACCCCCCTAGTCAAGCCCCCCTCTTTCAC

AGCGATAATCGGGAAAGGGGATACGGATTAGATCAAACAAAGGGGGAAATAGGGGTCTGT

AATAGATAGTGATTTCTCTTTTTTAGTGATTTCCCCCTTTATGTTTTTACTTACGAAGGT

CAACAAAAAC-AAAAAAAGAATAGGCCTTATTATTCCTACATGTTCCCATTCCCTTGAGA

TGTTACTACGTATTTTGCTTGTGTTTAATCTTTCACGATTCAAAAAAGAATCATTTATTG

GATTGGTTTCTCAATAGTGTTCGGTCAGAATCCCTTTTTGACTCTGCACCATTGATTCCA

CTATTATTAGAAAGGAATAATGGAATAATTCCTTCATATTTATAGAGATAGGGGACATAA

CTCACATGGATATAGTAAGTCTCGCCTGGGCTGCTTTAATGGTAGTCTTTACATTTTCCC

TTTCACTCGTAGTGTGGGGAAGAAGTGGACTTTAGAAGTACTACTAATTGAGTTGAGGAA

TCAAACTGTATCAATTGTTTTATAGATCGTTCTGCAACGCGTTTTGAACTATTTAAAATC

AAAATATCTGAATTTCCAATTCCATTGGAGTCCAATGGAGTAATGTATGATAGGAATCAT

ACTCTTTCAATCAAAGAACTATTTCAATGATTCCCATGTTTGTATTTCGAAAGGAAAGGG

ATCCAGATGATTGGAAATTTTTTCCAATCTAATTCTTCTGAAATTTTCTATTTCAATTAA

GGGGCTCTTACTATCCTTATAGATTAAGATTAGATGGATACTGAGGAAGACCAGACCTTT

TTTTGATCCCTCTTGACTCTTCAAAGAAGAAGTCGTTTTGTTAAGTGTATACGCACTTTC

TATGAGAAATGATATAGACATAGTGGTTGTCTAACGAGATATGCAATAATAAGATCTTGC

CTCAGGCGAGTCACATATTGCGCATTTACCGATGGGTTTCTAATTTTAGAAAGGAGATTT

TATCTTTATCGACTTATTTGATATCATGGTTCGGGCGTTAAAAATCGGTGAGGTTTACTC

TTCCTTTTCGAAATCCGAAGAAGTGCCCGTGGTCCTCCTGTCAATAGTTAAATCAATTAT

TTCTTCGGAATACTAAA-AAAAAGATTACTACGCGATTTTAGTAATCTATATGCCCATAT

CGTTTTTCAATCATTGATTCTTTCCATAAATACCGATATTCAGATTGGAAATCATAAAAA

ATCTAGTAATTCGAATCATAATTCGAATCATAAGATAAGAGTTAAGGCGATTATTTCAGA

TTGATCGGAACAAGTAGATGTAGCAAATAAATAGAATTGGGTGCTATGTCAATTCCATAC

AATATAGGGAATTTATATACACATATATGAAGAGAATATTGTAGATTGATCTATATAGAA

TGAAGCCTCTATCTTTATTCTAGAGTAGAACTTTATAGACTAAGAGATAGATAGTATGGT

AAGAAAGATCTTTCTTACCATACTATCGAATTCATAAAATACTGCCGATTATAGTCCGCT

CATTTCATTTAAGACGCGAAATTGGAATCCTTTTCATTTTACTTCGTCCATTTTTGATAA

GAACTCAGAAGGAAAGTTTCATTCAAATGAA-TTTGAAAATTGAATTGAATTAATCATTT

TGACTGACTGTTTTTACGTAAATGATAAGTAGAAAAGCGGTAGGAACTAGAATGAATAGT

GCAGTCGCAATAAATGCAAGAATATTTACTTCCATAATCTCATCGGTTTTTTTACTTCGC

AATAACTCGGGATTTAATCCCATAGAGATGATAAATCTTTCGCCTGTAAATTCAATGAAT

GAATTACCTCTCGACGATCTTGAATCGGATCAATATCATGAATAACAATATCTGAGCTAT

CAAATCAATTCGTCGTCGAGACTTGAATAGTATAACATAGGAAGTTCTTTTATCCATACC

GAATCCAAACTTGGATTCCTGACCCAATCAATCCAAAATTCCTTTATTTATCATTTGTTT

CCCTTCTTTTTTCTATAACCTACCTTACGTCTTCCTTGTACAATCATCTGATGATCATCA

GATTGCCCTTCCACTTCGATTAGTCACATAGTTACAAACCCAAACAAACAAGAAAAGCGA

AATGG-AAAAAAAAGGGTTAAGTTCTAAACTCCTTGTGATTTTTTGGAAGACGAAGACAA

AGAAGTTTGATAAAGATGAGGCCGGTATAAAAGATCTAATATCACTATTTTAGTGTTTGT

TATTTCTTCGATGGGACCTTAAAAATGGAAAAATAGGAAATAAAAAAAGCCCCTTTGTTT

TGGAAATTCAATTCTGCCCCCTGACATCCTTTCATAGAAAGGGAGAAATTAATTGATGTA

TTTATTGGATCCGTCGGGACTGACGGGGCTCGAACCCGCAGCTTCCGCCTTGACAGGGCG

GTGCTCTGACCAATTGAACTACAATCCCAGGGAAATAAGGGATCTAGCAGAAAATTTTAT

TCTTTTTTTATCTTCGTATTTCGTATGGGGTATTTCGGAAGGACAAGGGGATTATACAAT

CTCATGGTAGATTGGCGAATTATTGGGCCGAGCTGGATTTGAACCAGCGTAGGCATATTG

CCAACGAATTTACAGTCCGTCCCCATTAACCGCTCGGGCATCGACCCAGGAAGAATCCAC

TTTAGGCTTATTGGTAATCCATGATCAACTTCCTTTCGTAGTACCCTACCCCCAGGGGAA

TTCGAATCCCCGCTGCCTCCTTGAAAGAGAGATGTCCTAAACCACTAGACGATGGGGGCC

GACTTGCCCAACCGCCCTCATACTATGATCATAGTATGAACAGTTTTTTGAAATTGTCAA

TATAATGGAATGGTATGATTAGACTCGCGGGATCTTTCCGTTTTTCAGAATTGTATAGAA

TTTTTTGATTCGTCATCCATATTCATGAATCGTTCATTAGAATATTAGAATCGCCACACT

CTATATAAATAGAGTATAGAAATCTATATTCTTTAGAA-----TAATTAAAAAAAAAAAA

GAAATACAAACAACTAAAAATAATATGAGGGATAGGATTTGTTCAGGGAATGATTGGTCC

GTCAGAAAACAAAAGGGAGGGTTAATTTCGATTTTTTTGCTTTCATTCATTGTTAAGATG

AGATATCCTTATCTCTATCTCACACTAAGACGGGAAATTAACAACCAATAAATCTAGTAA

GCGGGATCAAGAAGTTATCGAAAATTTTCTCTAAGAATTTAGTTCAGGGGACAAGTAGAA

TCTCTTCATCACATGAAATATCTTTAAATTTATGTAAAATTGGTAAGTGTACACGTATGT

TATGTATCAATCAAGTGAATTTTGTTTTAATGGGGATCAATTCAATAAAATAAATTAGGG

TCGGTCTTGAATTCATTTTATTTTACCCTAGACTTGCTAGGTAAATCCATTTGATTATTC

AAGAATCAGCCACTAGCCACTACGAGTCTACTGCATGTACTTATGTATATAATATATGTA

CATATAGAGATTTTATCTACATAGTGACTCATTCGGGAATTAAATCAAATAAGCCCTTTT

AACTCAGTGGTAGAGTAACGCCATGGTAAGGCGTAAGTCATCGGTTCAAATCCGATAAGG

GGCTTTGGTTTTTTCATAAAAGTGCAGCCGTAGTATTCAGAAAATAGAGATATTTTTTTT

TATTTGGAATAAAAAAGTAACTAACTGGATAATACGTTATCATTATACTGAGTTAGAGTA

TAGTAGTTCTAGTTAGAAAGTTGAACATTTTTTCAGTCAATTCTCATTATTATGAATAAT

GATAAGCCGCCTCTTGAATCACCAAAGATCCCTATTTTACATTATACCAATCAAATCCAT

TGGAAAGATTCGAAATCAACAAAAGAAAAAGTAAGTGGACCTGACCTATTGAATTATGAC

TATATCCGCTATTCTGATATTAAAATTCGATAGAGATGAAATTGGAATTGGAACAGTGGA

CCCCCCTTTTTTTTATTTCATTTCTTTGGACCTCGAAAGAATTTGTCGATATTTCCGATT

AAATCTTCTTGTTCCTAGATTTTCTATGGGAATAAATTGTTATTCCCTTCCTCTACAGAG

AAACCTTTCTTCCAAGTCACAAGATAAGAGCCATTTCCATTATCTTTCTTTGATTACAGA

TCAAGATGAATTTATATCTATCTAAGTATATTTAGATAGATATCTATCAGATCGTGGCTT

CATGTACCAAACATTTCTATATCGCTGCATCCGATATTTTTGTTCCGACAGTGTGATGGA

GAATGGATGCGAGAAAGAGACTTTCATTTTTTTTTAAGGAAAAAAATAGGAAATTCTCTC

TTTCTAAGAGATTAAACTCAATAGAAAAATATTCGAAGTATCTTTTTTGCTTTGACCCGT

GGGAAGATATACTCTGGAGTTTTAGATTTATCTGAAGGAAAAGGAGATAGAACAAAGAAG

ACACTCAAAGAAAATGAAAAAAAAAAAAATGGAATCAAATAATGTAATTGGAATAGTTTA

GTATACATAGAAATTAGAATAATCTATAAATATCTTTATTTTTCTCAATCTCACGAACAA

GATCTAAGAATAACATTAGTTAATCGAACAAGAGGGGGGTAGATCGGAGGATCAGTTAGT

AGTGAGAGAGGGGATCACTTGTTCCTTGAAAAGTTCTTTCAAAGGATTCATCTATCTGAT

TGATGAATCATAAGAAGACAATTCATGGTTCATATTCTTAGTAAGAAAGAATAATCAAAT

TGAGTTCATGGATTTCCCTAGGTCAGTTTATGGGCCAATCCAATAAAGTATTTTTATCTT

CGAAACCCATTGGAAGGGGCAGTGTAAGAGAAATCATAGAGAAATGATCGAATCTTCGGA

CGCCCCGAAAATACTGTGAGGTGCTCGGAAATGGTCGAAGTAGTTGAATAGGAGGATCAC

TATGACTATAGCCCTTGGTAAATTTACCAAAGACGAAAATGATTTATTTGATATTATGGA

TGACTGGTTACGGAGGGACCGTTTCGTTTTTGTAGGCTGGTCCGGTCTATTACTCTTTCC

TTGTGCCTATTTCGCTCTAGGAGGTTGGTTCACAGGTACAACCTTTGTAACTTCATGGTA

TACCCATGGATTGGCTAGTTCCTATTTGGAAGGCTGTAATTTCTTAACGGCCGCAGTTTC

TACTCCTGCTAATAGTTTAGCGCATTCTTTATTGTTACTGTGGGGTCCTGAAGCACAAGG

AGATTTTACTCGTTGGTGTCAATTAGGCGGTCTGTGGACTTTTGTTGCTCTCCATGGCGC

TTTCGGACTAATAGGTTTCATGTTACGTCAATTCGAGCTTGCTCGATCTGTTCAATTGCG

ACCTTATAATGCAATCGCATTCTCTGGTCCAATTGCTGTTTTTGTTTCTGTATTCCTGAT

TTATCCACTAGGTCAGTCTGGTTGGTTCTTTGCACCTAGTTTTGGTGTAGCAGCTATATT

TCGATTCATCCTCTTTTTTCAAGGATTTCATAATTGGACGCTAAACCCATTTCATATGAT

GGGAGTTGCCGGTGTATTGGGTGCTGCTTTGCTATGCGCTATTCATGGTGCTACCGTGGA

AAATACTTTATTTGAAGATGGTGATGGTGCAAATACATTCCGCGCTTTTAACCCAACTCA

AGCTGAAGAAACTTATTCAATGGTCACCGCTAACCGCTTTTGGTCCCAAATCTTTGGGGT

TGCTTTTTCCAATAAACGTTGGTTACATTTCTTTATGTTATTTGTACCAGTAACCGGTTT

ATGGATGAGTGCTCTTGGAGTAGTCGGTCTGGCCCTGAACCTACGCGCCTATGACTTCGT

TTCTCAGGAAATTCGCGCAGCGGAAGATCCTGAATTTGAGACTTTCTACACCAAAAATAT

TCTATTAAACGAAGGTATTCGTGCTTGGATGGCAGCTCAAGATCAGCCTCATGAAAACCT

TATATTCCCTGAGGAGGTTCTACCACGTGGAAACGCTCTTTAATGGAACTTTAGCTGTAA

CTGGTCGTGACCAAGAAACCACCGGTTTTGCTTGGTGGGCCGGGAATGCTCGACTTATTA

ATTTATCCGGTAAACTACTAGGAGCTCATGTAGCCCATGCCGGATTAATCGTATTCTGGG

CCGGAGCAATGAACCTATTTGAAGTGGCTCATTTCGTACCAGAGAAGCCTATGTATGAAC

AAGGATTAATTTTACTTCCCCATCTAGCTACTCTAGGTTGGGGGGTAGGTCCTGGGGGGG

AAGTTATAGACACCTTTCCCTACTTTGTATCTGGAGTACTTCATTTAATTTCCTCTGCAG

TATTGGGCTTTGGCGGTATTTATCATGCACTTCTGGGACCTGAGACACTTGAGGAATCTT

TTCCATTCTTCGGTTATGTATGGAAAGATAGAAATAAAATGACCACAATTTTAGGTATTC

ACTTAATCTTGTTAGGTCTAGGCGCTTTTCTTCTAGTATTCAAGGCTCTTTATTTTGGGG

GCGTATATGATACTTGGGCTCCGGGAGGGGGAGATGTAAGAAAAATTACCAACTTGACCC

TTAGCCCAAGTATTATATTTGGTTATTTACTAAAATCGCCCTTTGGAGGGGAAGGATGGA

TTGTTAGTGTGGACGATTTAGAAGATATAATCGGAGGACATGTATGGTTAGGTTCCATTT

GTATACTTGGTGGAATCTGGCATATCTTAACCAAACCCTTCGCATGGGCTCGACGCGCTC

TTGTATGGTCTGGGGAGGCTTACTTATCTTATAGTTTAGCGGCTTTATCCATCTTTGGTT

TCACTGCTTGTTGTTTTGTCTGGTTCAATAATACCGCTTATCCTAGCGAGTTTTACGGAC

CTACTGGACCAGAAGCTTCTCAAGCTCAAGCATTTACTTTTCTAGTTAGAGACCAACGTC

TTGGGGCTAATGTAGGATCTGCGCAAGGACCTACAGGTTTAGGTAAATATCTAATGCGTT

CCCCAACCGGAGAAGTCATTTTTGGAGGAGAAACTATGCGTTTTTGGGATCTGCGTGCTC

CTTGGTTAGAACCTCTAAGAGGTCCAAATGGATTGGACTTGAGTAGGTTGAAAAAAGACA

TACAACCTTGGCAAGAACGGCGTTCCGCAGAATATATGACTCATGCTCCTTTAGGTTCTT

TAAATTCTGTGGGTGGCGTAGCTACCGAGATTAATGCAGTTAATTATGTCTCTCCTAGAA

GTTGGTTAGCTACCTCTCATTTTGTTCTAGGATTCTTTTTCTTCGTAGGTCATTTGTGGC

ACGCGGGAAGGGCTCGTGCAGCTGCAGCAGGATTTGAAAAAGGAATTGATCGTGATTTTG

AACCTGTTCTTTCCATGACCCCTCTTAATTGAGATGAGACAGGAGATCCAATGCTTGGAT

TGAAGTAAGAATCACTTTGATTCTATCATACATA-TTGGGATCGGGTTATACTTAAAAAG

TATTCCTTTTTC--TTTTTTTTTTTACTCATTTATATCTAATCTATTTTTTCTGGCTTGG

CTAAGTGGGATAGCCGAGCCATTCCCCTTTCTTTATGATACCAATCCGAGCCAAACCAAT

AGAAACAAATCTATTCAATGAGA-AAAAAAAGGAGAGAGAGGGATTCGAACCCTCGATAG

TTC-TTGTTCAAAACTATACCGGTTTTCAAGACCGGGGCTATCAACCACTCAGCCATCTC

TCCGAAAGACAATTTTTATTTTATTCCTCCGAATAGAACATGGCCATAGGGGTGGATACC

ACTACTATCTGTAGAAAGATCTCAGGTGTG-AATCTACCGATGGATCTATCTATCCGTAT

ATATATAATCCAGCATGTCCATTTGTGAAATATAAAAAACAAAATTCCATTTCCCCTGAC

TCCATGTATGAATAAAGTGGTCAAAGGGGTAGTAATAAGTCATATAGAATCAATTGATTC

ATGGTAAACTCAAATCCCTCGATGATGTATTTTATTACAATTTTTTGGCTGATAGAGGGA

TCAAATGGTATAGTTCATTTGTTGGTATCTTGGAGGATTAAAAGCATGACTCTTGTTTTC

CAATTGGCTGTTTTTGCATTAATTGCTACTTCATCAATCTTATTGATTAGTGTACCCGTT

GTATTTGCTTCTCCTGATGGTTGGTCAAGTAACAAAAATGTTGTATTTTCCGGTACATCA

TTATGGATTGGGTTAGTCTTTCTGGTGGGTATCCTTAATTCTCTCATCTCTTGAACCTAT

TCGTCCCAGATCCAAAACCGAAATGACCCCCCCCAAAAA--AATTTTCTCGGTTATGAGA

CACATTACAATTTAATATAAGTCCCCAAAATAAAATGGGGGGGTCAAACTTCTTGCAAAC

TTCTTGAATTAAAAAAAAATACAATTTCAATTTATTATATTAATAATATAAATCGCCCCG

AAGAGAGTCTCTGGCCCGACACTGCACAAATACGATCCAGG----TATATATATCATATA

TGTGTGGATATATTGTGTATTAAGAACAAAAATTGCGGATATGGTCGAATGGTAAAATTT

CTCTTTGCCAAGGAGAAGATGCGGGTTCGATTCCCGCTATCCGCCGAAGATCAAGATAAA

GTTATTTTTCCTTTAATATGATAAAGGATTGGGTATAGTTGGCCATGATAGTGTCGCGAG

TCTATCCCTTCTTTTTTTCCTTTCCTACCA-CCCCCCTTTTTGGGGGGGTAATTAATTAC

TAGTTAACAGAGCCAAACCCCCTTTTGATAAAAAAAGATATTGCGGAGACAGGATTTGAA

CCCGTGACCTCAAGGTTATGAGCCTTGCGAGCTACCAAACTGCTCTACCCCGCGTTGAAG

AGAAGAAGTGAAAACTAATAGACAAACAAGAATTGAATGTGCCCCTCTACCATATCTGTA

CAAATAGAGTAGCCCATTTATACAGAATGGTAAAGAGGCCCTATATGATCATCGACCATA

GAAATGAAAGGTTAATCCTTACCAACTTGATCTTGTTGCTCCTGGCAACAAACACGCATG

AACCATTTCACGAAGTATGTGTCCGGATAGTCCAAAGTCTCGATAGTTAGCTCTCGGTCT

TCCGGTCGAAAAACAACGTCGATGAAGGCGTGTAGGTGCACTATTCCGTGGTGGGGATTG

TAACTTTCCATAAATTTCCCATTTGTCACTCAACGATGGAACTTTGCTTATTTCTTTTTT

TGAGGATCGACGAATCAAATGATATTTCTGTTCCAATTTTTGCCTCTTCTTCTCCCTCTG

AATCAAACTTTTCTTTGCCATAATGGTTCAGGTCCTATTAGTATCCATGATACAAGTCGA

ATCCTAGATGTAGAAATATAAGAAGGTGGACCCTCTCTCCATCGAAAGAAATGAGATTAT

CGCGGATACAACACATTAAAAAATTAGCCAAATTTCCCCGATGTAGAGGCAATCAAAAAA

GCCGCATAAGTGAATATATAACCTACAGAAAAGTGGGCTAATCCAACCAATCTTGCTTGG

ACAATGGAAAGAGCCACAGGTTTATCTCTCCATCGAATCAAATTGGCCAAAGGTGTGCGT

TCATGAGCCCATGCTAAAGTTTCAATCAATTCCTGCCAATATCCACGCCAGGAAATTAAG

AACATAAATCCAGTAGCCCAAACAAGATGTCCAAATAAGAACATCCATGCCCAGACCGAT

AAACTATTCATACCAAAAGGATTATATCCATTGATAAGTTGTGAAGAGTTTAACCATAAA

TAATCTCTTAGCCAACCCATCAAGTAAGTGGAAGATTCATTAAACTGTGAAACGTTACCC

TGCCATAATGTGATGTGCTTCCAATGCCAATAAAAAGTAACCCATCCAATAGTATTTAAC

ATCCAAAAAACTGCCAAATAAAATGCGTCCCATGCCGAAATATCACAAGTACCCCCTCGT

CCCGGGCCGTCGCACGGAAAACTATAACCGAAATCCTTTTTATCTGGCATTAACTTGGAA

CCGCGTGCATCTAAAGCACCTTTTACTAAGATCAATGTAGTTGTATGTAAACCCAGAGCA

ATAGCATGATGAACCAAAAAGTCTCCAGGACCTATTGTCAAGAATAATGAATTACTATTT

TCATTAATAGCATTTAACCAACCGGGCAACCAGATGCTTCGACCCGCATTGAATGCCGGA

CCGCTCGTTGAAGATAAAAGTACATCGAACCCATATGAAGTTTTCCCATGAGCCGATTGT

ATCCATTGAGCAAATATAGGTTCGATCAAAATTTGCTTTTCTGGAGTACCAAAGGCAAGC

ATGACATCATTATGAACATAAAGTCCCAAGGTATGGAATCCCAGAAAGAGGCTGGCCCAA

CTTAAATGAGATATGATAGCTTCTTTATGGTCTAACATTCTTGCCAATACATTATCTTCA

TTTTGCTCCGGATTGTAATCTCTAATGAAAAATATAGCTCCATGAGCAAAAGCTCCTGTC

ATGATGAATCCTGCGATATATTGGTGATGAGTATATAATGCAGCTTGAGTAGTAAAGTCT

TGTGCTATGAATGCATAAGCAGGTAAAGAGTACATGTGTTGAGCTACTAAGGAAGTAATA

ACCCCTAAAGAAGCTAGAGCAAGGCCTAATTGAAAATGAAGCGAATTATTGATTGTGTCA

TAAAGACCCTTATGCCCTCGTCCCAATCGTCCCCCCGGAGGAATATGTGCATCTAAAAGA

TCTTTGATACTGTGTCCAATCCCGAAATTCGTTCGATACATATGACCAGCAACAAGAAAA

ATAAATGCAATAGCTAGATGATGATGAGCAATATCGGTCAGCCATAAACTTTGTGTTTGT

GGATGGAATCCCCCGAGAAGAGTTAGAATGGCAGTTCCTGCTCCTTGGGAGGTCCCAAAT

AAATGACTACTTGAATCAGGGTTTTGAGCATAAAGATTCCACTGACCTGTAAAAAGTGGG

CCTAACCCTTGGGGATGCGGTAATACCTCTAAGAAATTATTCCATCGAACATACTCCCCT

CTGGATCCAGGAATAGCGACATGTACTAAATGCCCTGTCCAAGCCAAGGAACTTACGCCG

AAGAGTCCTGACAAATGATGATTCAGACGAGATTCGGCATTTTTGAACCACGAAACGCTC

GGTTTCCATTTCGGTTGTAGGTGTAACCAACCTGCTATTAAGGATATGGCAGAAAGAAAT

AATAGAAAAAGAGCTCCAGTATAAAGATCTTCATTAGTGCGTAAACCGATTGTATACCAC

CACTGATAAACACCAGAATAAGCGATATTCACTGGGCCAAGAGCACCCCCTCGAGTAAAA

GCTTCCACAGCCGGTTGACCAAAATGAGGATCCCAAATCGCATGAGCAATAGGTCTTACA

TGTAAAGGGTCCTGTACCCATGATTCAAAATTTCCTTGCCAAGCTACATGAAACAGATTT

CCGGAAGTCCACAGAAAAATTATTGCTAATTGACCGAAGTGAGAAGCAAAAATATTCTGA

TAAAGACGTTCCTCAGTAATATCATCATGACTCTCGAAGTCATGTGCGGTAGCAATACCA

AACCAAATACGACGAGTAGTGGGGTCCTGAGCTAAGCCTTGGCTAAACCTTGGAAATCTT

AATGCCATAATGCCTTTCAAATCCTCCTAGCCATTATCCTACTGCAATAATTCTTGCTAA

GAAGAACGCCCATGTTGTGGCAATTCCACCCAGAAGGTAATGGGTTACTCCTACAGCACG

TCCTTGTACAATGCTCAAGGCTCTCGGCTGAGTAGCAGGAGCAACTTTTAATTTATTATG

AGCCCAAACGATGGATTCAATAAGTTCTTGCCAATAACCACGTCCGCTGAATAGAAACAT

CAAACTAAAAGCCCATACAAAATGAGCACCTAGGAAAAAAAGTCCATATGCAGATAATGA

AGAACCATAAGACTGAATTACCTGGGATGCCTGTGCCCATAAGAAATCCCGGAGCCACCC

ATTAATAGTAATAGAACTCTGGGCGAAGTTTCCTCCCGTGATATGAGTTACTACCCCTTG

ATCACTTATACTGCCCCAAACATCTGACTGCATTTTCCAACTGAAATGGAATATTACTAC

CGAAATGGAATTGTACATCCAGAATAGTCCTAAGAAGACATGATCCCAAGCCGATACTTG

ACATGTACCCCCTCTTCCAGGTCCATCACAAGGAAAACGAAAACCAAGATTTGCTTTATC

CGGTATCAAACGGGAGCTACGAGCAAATAGAACACCTTTCAGGAGTATCAATACCGTGAC

ATGAATCGTAAATGCATGGATGTGATGTACCAAAAAATCCGCGGTTCCTAACGGAATAGG

TAACAAAGCCACTTTGCCACCCACTGCTACTAAATCACCACCCCCCCAAGTTAAACTGGT

GCTTGCTGTTGCACCAGGAGCCGTTGCGCCAGGTGCTAAAGCATGGGTGTTTTGTATCCA

TTGAGCAAAAACGGGTTGTAATTGTATAGCGGTATCTGAAAACATATCTTGAGGACGCCC

TAAAGCGCTCATGGTATCATTATGAATATATAAACCAAAACTGTGAAAGCCTAGAAAGAT

ACATGCCCAGTTGAGATGTGATATGATTGCATCACGATGTCTAAGGACACGATCTAATAG

ATCGTTGTATCGAGTAGTTGGATCATAGTCTCTTACCATAAAAATGGCTGCATGCGCAGC

AGCACCAACTATGAGAAATCCACCAATCCACATGTGATGTGTAAACAATGACAGTTGGGT

CCCATAGTCAGTAGCTAGATATGGATAAGGGGGCATGGAATACATATGGTGAGCTACAAC

AATGGTTAAAGAGCCTAACATAGCTAGGTTAAGAGATAATTGAGCATGCCATGACGTTGT

TAGGATCTCATATAGGCCTTTATGACCCTGACCTGTAAATGGACCTTTATGAGCTTCTAA

AATATCTTTTAGGCCATGGCCAATGCCCCAGTTAGTCCTATACATGTGACCCGCTATCAG

GAACAGAATTGCAATAGCTAAATGATGGTGTGCAATATCGGTCAGCCATAGACCCCCAGT

TACTGGATCTAATCCTCCACGAAAAGTAAGAAATTCCGCATATTTTGACCAATTCAAGGT

GAAAAATGGGGTTGCTCCCTCGGCAAAACTTGGATAAAGTTGAGCCAAAAGTTCTCGATT

CAAGATAAATTCATGAGGAAGCGGTATCTCTTTAGGATCTACTCCAGCGTTTAAAAATTG

GTTAATCGGTAAAGATACATGTACTTGATGCCCCGCCCAAGAGAGAGACCCAAGTCCTAG

TAGCCCCGCTAAATGGTGATTCAACATAGATTCTACATCTTGAAACCAAGCCAATTTTGG

GGCCGCTTTATGATAATGAAACCAACCAGCAAAAAGCATTAACGCTGCAAAGACCAATGC

ACCGATTGCGGTACAATAGAGTTGTAATTCACTAGTTATTCCAGATGCTCGCCAAATCTG

AAAAAACCCAGAGGTTATTTGTATTCCTCGGAAACCCCCGCCCACATCACCATTCAATAT

TTCTTGGCCCACTATTGGCCAAACCACCTGGGCACTAGGCCCAATGTGAGTTGGATCACT

TAGCCACGCTTCATAATTGGAAAAACGAGCACCGTGGAAATACATACCGCTCAGCCAAAG

AAAGATGATGGAGAGTTGACCGAAATGGGCACTAAATACTTTTCGAGAGATCTCCTCCAA

ATCACTGGTATGGCTATCGAAATCGTGAGCATCAGCATGTAGGTTCCAGATCCAAGTGGT

AGTATCAGGTCCTTTAGCTATTGTTCTTGAGAAATGGCCCGGTCTGGCCCATTCCTCGAA

AGAAGTTTTTACGGGATCCCTATCCACCAAAATTTTGACTTCTGGTTCCGGCGAACGAAT

AATCATTGAGTCCTCCTCTTTCCGGACAACACATACAAAGAGACCCGCCAACAGTCAAAT

AATTAGTAAACCTTTGAGAGATATTTCTATAATTAGTTTGTTTCTCTTCTATCTCCCATT

TATCTATTTTCTTTAGTTATTCACTAGAGCAATTAGGATCTGGAAGTCGATCCGGGGCAA

GTGTTCGGATCTATTATGACATAGCCATGAGGCGCTCAACGGACCTTTTTTATCTTATAA

AATCTTATAAAACCTTTTTGTAGCTTTGGATTGATGCAAAAACGACTTTTTTGTACAACC

TAGTGTAGATTCATATCTCAATTAGAAGGTCTTAGATAGAGCTGCTTTATCTTTTCCATA

GATGATATGAATTACTCTATTCCAAATCACGCGAGCAGCCATTACTAAGAGACATCCCGG

TATATA--TTTTATTTAGTTGTTAATTTAGATTTAAATTTAGGTTTTTAAAAAAGAATTC

GTTTTCATATTAATAATAAAATTAAAGTAAAGAAGTATATTTTTGACTCTATCCCTTTTT

TATCCCTACGAAATATCAAATGAAATAGAACGCTTAGAAGGGATATAATGAAATTCTTTG

ATTGGATCTTCCCAAAGCAAAGGAATGATCCA-TTTTTTGATTTGACTGATGGGGCCAAC

AAACAAAAAATTCTAACAAATATATATAAATAATAGAATAGAATTAATAAACTAAATAAA

CGGCGTCTTCTTTTATTCGAAACGCCCCGTGATCTTCAACCAATTATGTGCTTCAATATA

ATTACCAGGAGTAAGCGCTATAGCCTGTTTCCAATACTCAGCTGCTTGATCAAACCAAGC

CTCCGCAATTTCAGAATCTCCCTGGCGAATGGCCTGTTCTCCCCGGTCGGAATAGGTAGG

TTAATTCCTTCCCTTAGAACCGTACTTGAGAGTTTCCTAACTCATACGGCTCAGCAATCA

TTTGGTGTCCCCCTTTAATCTACCATATCTAACGAATGAGATTTCTGCTGGATCTATCCC

ATTTTGGGGGTTAACCAAAGAGGTTCATTACCTGAGTTTTAAACTGAAATTTGGATTCAA

TTTGGATTAATAATCCGTTTTATTTCGTTTTATCTTTTTTCCCACCTTCAGAAGAATAAA

TTCCTCCTATCGTTAGAATTTTCTGAAAGGTAACTATCTCGGTTTCATATATAAATTTCT

ATAGAATCTTTGAAAAAGACTTTCCTTCTTAAGAAAGAAAAGACTTACTATCTTTGGGAT

CTGATCCTACACCGCTGCTCAAGACTTTAGTGGATCGACTCTATTACATAAGTTGATTCC

TAATTTTTATCTCACATCATGAGTAGGTATATCTACCATCATGACATAAGTACGCAGTTA

TTATCGTATCGGCCCAAAACCTCGCTAATTGATCTTTACGGTGCTTCCTCTATCAATTAG

ATCCTTTTTTTATCCATAGAAAAAAGTAGCTAGGCATATCTATTTCTTCATATTTTGACT

TCTATGAAGTTTCTTTCTTTGCTACAGCTGATAAAAATCGTTGTTTTAGACGATGCATAT

GTAGAAAGCCTATTTTTTTCGTATTTACTAGCAGA-TTTTTTTTTCTTTCTATAGTGGAG

ATAGTCGCACGTAATGACAGATCACGGCCATATTATTAAAAGCTTGGGGTAAGAATGGGT

TTCGTTCTAGTGCTCGAAAATAATATTCCAAAGCTTTCGTATGTTCTCCATTACTTGTAT

GGATAAGACCTATATTATAGAGTATATAACTTCGATCATAGGGATCAATTTCTAGTCGCA

TAGCTTCATAATAATTCTGTAAAGCTTCCGCATAATTTCCTTCGGATTGAGCTGACATCC

GTTACGGTCGTAATTCAAGTAAAAGAATCTCCGTTCCAGAACCGTACGTGAGATTTTCAT

CTCATACGGCTCCTCCCTTATGTGCATAAGGAGAATAATACATGAAATCAAAAAAGACGA

AAATATTCTCATTATGAACTGAGCAGGGCTAGTGTTTTTACAAGAAATCTCTAGCCAACC

TTCCTGCAAGAGATCTTTTCTTAACATCAAGCGTGTTGGGACTAGATAGAAATGAGAACT

CCAACAATTTCTTTGTTTTCAACGCCTCCTAATTTCCAGGAATTAGTCACTTCAACAGCC

TTCGATGGTTATACGGGTATCCAAAGTACGAACGAGATGGATGTTTGTTGTCCCAACCAT

TCTTTTAGTCCCGAGCCCGATAAGGAAAGGGATAATTTCTAACAAAGTTTTCGTGTTGTT

GATTCCTAGGTGTAGTGCTTCTTCCCCTATGCTGCCTATTGGTACTAGTGGAGTAGGATT

GACCCATAATAAAGAACCCCTAGGTGTAACCTTTCGCTCAATACTAGAATCAAAATTGAA

ACATAGCATCTGAGGTTGCATTAATCGAGGATACACGACAGAAGGAATTGTTCTATTTCC

AAACTTCACCTTCAACAAGCGTAGATTTATTTCCAAAATTTTCCTGAATCACGTGTCTTT

CTCGTAAGACTGAGAGAAATAAAAAAAAAAA-AGAATCAAATCACACCATCTCTGTAATA

GGTAAATGCCTCTTTTTCTCCTGAAGTTGTCGGAATTATTCGTAATAAGATATTGGCTAC

AATTGAAAAGGTCTTATCAATAAAATTTCCATTTATCCGCGATCTAGGCATAGGTAGCAA

TCCATTCTATAATTCTTCTCATTACCTCTCGTGGGAAAATGATCCCACAAAGAAAAGAAT

TGTACAGTACGAAATAACATAAAAATAGATTGATTAAAAAAAAGATTATGGGCCTTCTGT

TCCTGAATCAATACAAAATAGTTGAACCCGATTCGATTTCATACTAATGTAGTAGTATAC

GGAACTATTCCGATTTCATTGAAGTTACAGATTAGAGTAACTCGATAAATTTGGATTGAA

TTATGATACAAAAACGAAAAAAGATCGAATAATCATTCTATGATGAAAATAGATAGAATA

ACCGCCTCTTTTGTCTGTTGTGTACATAGCAGGTATACAATCAACTATACAAAATGTTTT

AGAAATTTCTAATAGAGGGGGTTTTGTTGTTGAGAACTCTAAAACTGGAAAGGAATTTAA

TAATTTGTAGGGTATGGAATCATAGTCTCTATAGAATTATGATAGAAAGGTATCCATTAA

CCCTAGTCTAAAAAATCTAGACCCATCAATCAGTTGATTCATTCTAATTCATTGATTTAA

TCCATTCTAAATATTAGAAATAGAAGGGATAAATTTTTGCAAACATGAATCCCATTTTTC

ACAATTTTTCGTAAGAAAAGAATTTGATCTTTATCTCGGAGCCTCGAAGGAAATAGCTTT

CTTTACTTTGATGAAAAATTTTCGATTTTTATTTG-TATAGTTAAAATGGATGTACCTAT

CCCAATAATCTATAATGGAATATGAATAACTCCCT-ATTCACTCGGTTTTTGGTTCATAA

TCATTATGTAGGAGAGGTGGCCGAGTGGTTCAAGGCGTAGCATTGGAACTGCTATGTAGG

CTTTTGTTTACCGAGGGTTCGAATCCCTCTCTTTCCGTACCTTCACTTAATAGACCGATT

TGACTAACAACACTGGATCAAATAGCAATGGAGACCTTTATTCCACTAGTTAGACCTTTC

ATTGATATAGATTCTCTATTCCTAATTGCCGTGACACGTAAAATACTATAAAATAGGAAA

AATTCTGGAAAGAAAATGAAAATATCCCTTCGGTCTATGATACATATACATAAATGGGAG

AAAATCCGGATCAAACCCGTATTTTTCTTACTTAATCTTAGGTTCATTTACTTCGATGAA

AGGGAAGAAAATTGCCCGAACCCTTGTTATTTTATTTGAGTTTAGGTTTAAGTCTGACGA

GAATAATATTCTACGACTAGCAATTCATTTATTTTCAAACCAACCCATTTACTATCTATT

ATTTGATTGACTAATCCTTTATATTGGAATGGGTGAAGGGTCAAATGGTTTGGCAATTCC

TCATGAGGGGATGAGTCGAGAGAATTTTGAATCAGAGTTCTGGATTTTTGTTCATCCTTC

GTCATAATAATATCTCGGGGTTTGCAGCGATAACTTGGTATATCTACTATACGCCCATTA

ACTAAAATATGTCTATGGTTAACTAATTGGCGGGCTGCGGGAATAGTCGAAGCCATACCC

AATCGAAAAAGGATGTTATCCAAACGCATTTCAAGTAATTGTAGTAAAACTTGACCTGTT

GACCCCTTGGCTTTTCCGGCGATACGAACGTATTTAAGTAATTGTCGTTCTGTAAGACCA

TAGTGAAAACGCAATTTTTGTTTTTCTTCTAGACGAATACGATATTGAGATTTTTTCCCG

GAACGTGATTGGTTTCTAAGATCACTTCCGGCTTTAGGCCTTTTATTAGTTAGTCCTGGT

AAAGCCCCCAGGCGGCGTATTTTTTTGAAACGAGGTCCTCGGTAACGCGACATAAAGACT

CCTTATTCCTAGTTAGAATTAAT-TTTTTTTTATTTTACAGAATAAACCTAAACTAAAAC

TGAACTAAATGAAGCGAAATCTACTGAAGTAGTGTACTTGTACTATAAAGAAGAATGAGG

TAAATTGGATAAATATCCAGACTTTCTATTATTATATATATATAGAAAGGATCCTTTTCC

TGACATAGTTGGAAGTTCCTATAACTTCCAAAATTCATGGATTTTGGAAAAGGGGGAAGG

CACTTTTTCAATATTCTTTGATTTCAAAGGGACATTATCAATCATTAAAAAATGGAATAA

AAAATGAAGGAAAAGCCGGCTATCGGAATCGAACCGATGACCATCGCATTACAAATGCGA

TGCTCTAACCTCTGAGCTAAGCAGGCCCACATAATAGAAACTTTCTATCCATAGGAATTC

AATACACTACACTATAGTGTCTATAGAATATAGAAAAGGATAGAATATAGAATTTCAAAT

AAATATTAAATATTCTAGAACATAACCATTAATATAGCGATATAAAATTTCGATTTCTTT

ATCACAATTCTAATATTATTAGTATAGTAAATCTTAAATATTTTTAGATAGTCAAATTTT

CTTTTTCATTTTTGTTTGAATTCACATGACATTTGAAATTCTTTTTGTTACACTTCTCTA

TATTTATATTTTGAATTCTATTAATTCTTTCGAATTCGAATTATTTAATTGATTAGAACT

AATCAAACATTCCTCTGCTTTCATTCGTAAAGCGGAAAGAAAAGAAAAAAGAATCGACCG

TTCAAGTATTGCAAATTGCATGGGAAAAGTGGCAGGAAGAGAGACATATATATGGGGTAT

ATATCCATCTATATTGAATTGCCGATACAGAAATGATAAAATCCAATTTGATTGAATCAA

ATACGGGTTTCCGATAAGGAAGAAATCAAAGAGGGGAAAATAC-TTACAATGAAATCCTA

ATCTCGGGAAGGGGGATATGGCGAAATTGGTAGACGCTACGGACTTAATTGGATTGAGCC

TTGGTATGGAAACCTACTAAGTGGTAACTTTCAAATTCAGAGAAACCCCGGAATTAATAA

AAATGGGCAATCCTGAGCCAAATCCTGTTTTCCCAAAACAAAGGTTCAGAAAGAAAAAAG

GATAGGTGCAGAGACTCAATGGAAGCTGTTCTAACAAATGGAGTGGACTGCGTTGGTAGA

GGAATCTTTCCATCGAAACTTCAGAAAGGATGAAGGATAAACGTATATATTGAATACTAT

ATTAAATGATTAATGACGACTCGAATCTCTATCTGTATTTTTTTATATGAAAAATGGAAG

AATTGATTCCACATTGAAGAAAGAATCGAATATTCATTGATCAAATCATTCACTCCATAG

TCTGATAGATCTTTTAAAGAACTGATTAATCGGACGAGAATAAAGATAGAGTCCCATTCT

ACATGTCAATACCGGCAACAATGAAATTTATAGTAAAAGGAAAATCCGTCGACTTTAAAA

ATCGTGAGGGTTCAAGTCCCTCTATCCCCAAAAAGCCTATTTGACTCCCCAAATATTTAT

CCTATCCCTTTTTCATTAGCGGTTCCAAATTCCTTATCTTTCTCATTCACTCTATAAACG

TATTTGAGCGTAAATGATTTTCTCTTCTCACATGTGATATATAATACACATCCAAATTAA

GCAAGGAATCCCCATTTGAATGATTCACAGTCAATATCATTACTCATACTGAAATTGACA

AAGTCGTCTTTTTGAAGATCCAATAAATTCCAGGACTTGGAGAAAACTTTGTAATCCCCC

CTTGTCCTTTTAATTGACATAGACCCCAGTCATCTAATAAAATGAGGATGGGATGTTACA

TTGGGAATGGTCGGGATAGCTCAGCTGGTAGAGCAGAGGACTGAAAATCCTCGTGTCACC

AGTTCAAATCTGGTTCCTGGCACATGATTCATTTGTATGAGTCTCTCAATTAGAAATTAA

TTGATATGAATCGAGATACATATTCATTTCTAATCTAGATCATAATACATACTTTTATCT

ATCTTAGCGAGATATACCCCATCTATATAGATGGGTAGAGTTTTTTAAATAAAGTATCTA

AAAGAATTCAATTCTATTTTCTC-TTTTTTTTCTTTCGTTCAAAAAATCTGTTAATACTT

CATACATATTTGAAAAGTTCAATTAGTTGGTTGAAAGACTAAAAAGTCGAAGTTGAAAGA

GACAAGATTCGGTTCAGATACCAT--AAAAATAAAGAAAATCTGATACCCTTTCATTTCT

TTGTATTTTCTCTTTCATATTCGAT-----------------------------------

------------------------------------------------------------

--------------------------------------------------------ACGA

A-CCCCAATTCTGTCTTTTTTTGTTAGCCTATCCATAATTCCTAATATAAATATATAAAC

CAAACTTCAATTAGAATTATTCTGGTTAATCTAGAACCGAAAGTACAATCCTTGAATCTC

TGAAATTGTATAAGTGGAAATTAGTTTCTTATCATTCAATGAGCATCTTGTATTTCGTAA

AAATTGGGGGCAATATAATCCTTACGTAAGGGCCATCCTATCCAACTTTCAGGCATTAAG

ATACGTTTCAAGCGTGGATGATTATCATAAGAGATTCCCAACATATCATAAGATTCTCGT

TCTTGAAAATCCACACTTTTCCAAACCCAGAAAACGGACGGAATTCTAGGATTCCTCCTT

GAGGCAAATACTTTTATGCATACCTCTTCTGGTTGATCCACACCATACTCTATTCTCGTA

AGATGATACACACTAGCTAACAGTCCGCCAGGTGCTACATCATAGGCACATTGGGAGCGT

AGATAATTGTAACCATATACATATAAAATGACGGCAATGGAATGCCAATCCTTGGGCTTT

ATTTGTAAAGTCTCTATTCCTTGATAATCAAAGCCCAAAGATCTATGAATTAGCCCATGC

TTGACTAGCCAAGCAGACAAACGACCCTGCATCTTTTTTATCTCCCGCATTTTTAGCTAG

AATATTTCACATTCTCGATCAAATTTATGAAGATTCACCCACTACTTGTTATTCTGTACA

ATACAAAGGAATCCTGTCTAATTCACTAATTCGTGAGAAGATACTGAATTTTTATATTTG

AAAAAGTTTTCAGTAGGGATCTCTGAAGTAGATGGCGGTTGATAAAGGAATCCTTGATCA

TAATTTCCAGTATTCATACTGCGTCCAATATGAAACTTGTGATTGGTAGTAAAACACCGA

TTTGCTTGTTGAGACCTAATTCTATCTTCATAGATTTCTCGAGATATTTTCTTACGAAGT

TTTGTTATAGCATCTATAACTGCTTCCGGTTTAGGTGGACAACCTGGCAAATAGACATCC

ACGGGAATTAGCTTATCGACTCCCCGAACAGTACTATAAGAATCGGTACTGAACATCCCT

CCTGTAATTGTACAGGCTCCCATAGCAATAACATATTTTGGTTCGGGCATTTGCTCATAT

AATCTCACTAAAGAGGGTGCCATTTTCATTGTTACTGTTCCGGCTGTTAAAATTAGATCC

GCTTGTCTAGGACTCGATCTTGGTACTAATCCATAACGATCAAAGTCGAATCGTGATCCT

ATTAGTGAAGCAAATTCAATAAAACAACAACTGGTACCATAGAGAAGCGGCCATAAACTA

GAGAGCCTTGACCAATTTGAAAGATCATTTAATGTAGTTGAAATAACTGAATTTTGGACT

GTTTGATCAAGTAAAGGAAACTCAATGGAATTCATAACTTTTTCAATC-TTTTTTTTTCC

CTTTTCTTTTTATTGTCTGAATATTCAGGAGCTAAGACCATTCCAATGCCCCCTTTCGCC

ATGCATAAACTAAACCAACAATTAAGATAAGCACGAAAATTAAAGCTTCTATAAATACAG

ATACACCCAATACATCGAAACTCATTGCCCATGGATAAAGAAAAACCGTTTCAACATCAA

AAACAACAAAAACGAGAGCAAACATATAATAACGGATTCGAAATTGTAACCAAGCGTCGC

CCATTGGTTCTATACCCGATTCATAACTAGAAAGTTTCTCCGGCCCTTTCCTAATCGGGG

CTAAAATCCCGGAAATGAAAAATGCCAAAATAGGAATAAGACTTGATATTATTAGAAATG

CCCAAAAAATATCATATTCGTAAAGCAAAAACATAGACGCACTCCTATGAACGTGGAAAA

TATACCGGATGGGTCGATTCGAATTGAAATTGTCAAGTCATCCATAACTGTTTAGTCAAA

ACAAGAATTCATTTTGACCAAACCATCTAGTTTCCTTTGTTTATTGTGGGGCATATCTCA

TTTCAAGATTCATCGACTGACTTGACTGGAATCCTATTTCCAGTCTACTTATTTTTATTT

CTTTTTATTTCTATTTTATTTAGTTAGTATAACTCTAACTATTACTTTTATACAAATTCT

CTTGTTTTCACCTAGGATTCTTGCTAAAGAAACCTAGTTCCAAATAAAAAGAAAATAGAA

TTCTTATTTTGTGTTTAGAATTTCTAACTTATTATTTTTAAAAT--TATATATTATTCTA

AATTCTTTAGAGATTTCTA-TTTTTTTTTTAGGAATAGAATCTGGAGTTTGTCGTTTTTT

TCTTAGTGATTTCGAATGGAACAAGTATTCAAATGTAAGAGAATGGATAGGTATTTTGAT

TTCTAATATCTTAGTGTTGGTATATTCCTTTTATTTTATTAGAGGGGTTTCTCTTGATTG

AATACAGAAAAAGAAGACCATCCCCTTTGTGCTTCGATAGGTCTAGGTAAGGTATACGAA

GAAAAAGCCTATTTGACATTGTTGACAATGAAACTTACCAAAGAGATTCGTTTCTCAACA

AACTTTGGCTTGTTGATTGTGGAC-AGGTAAATTCATATGGAATTTACCTCCGAAGATTA

ATGACGAAAGGTTGGTTTGTTTATCCACGATTGGATAAATATCGATTCGATCCCTTTTTC

TTTCAGTTTTATGTTCTGCTTTAAACGATTCCCGTGAGTGAGTTTATAGGAATAATTTGG

ATTTCGATGAACCAACCCAACCGGTCAGTTACAAGCAACAAACAATAATGAAGAAATGAA

AATTCGAATATTTTCTATTTTGAATTTTCATTTTATAGGGCTCTAGGGCTATACGGACTC

GAACCGTAGACCTTCTCGGTAAAACAGATCAAACTTATTATTATCAAAATGATCTGAACT

GTTTCAAAGACCCAACATGCATTTTTTTTGCCTTGGGCTCTTTCATTAACTGATAGAAAT

ATCAGCCAGTCTGCCATATTTTTCAAAATAAGATTAAGGAGATGGCTCCATGTGCTCTGA

TTCATTATTTGGGATTCTGATCCAGGAGCACTACCAAAGTGTTTCAAAGGTGGGGTTATC

TTGACGTAGGTCTGCCTCTGGCCTAGATCAACCTAAGTTAAATGAAGTCTCTATCGTTCG

GCTTAAAAAATAAAATATGAAACTTCATACACCTTAAAGTTCATAGGACGAAAAGAGATT

TTTTGAGGACCTTATACTCATTATGCCTAGCATTGAATGGACTGGTATTGACCTTATCAA

TATCTCAAATCAATGTATGGGGTCTGTTTGGTACCTAAATGGGCACCAAAATCGGACCGA

ACCATTTGTCAGGCTACTGTTCCCTCACAGTTATGGAGTAAGACATCGATTTCTCAATAA

GATGCATTTTTTTGATTGTATGATGGACCCCCCTGAAAAACATTGGCGCGCGTGTAAACG

AGGTGCTCTACCAACTGAGCTATAGCCCTTAGTGCTTGTGATACATATTTTATCATGGAG

ATAATTTCTTGTCAAGATGAATATTCTATGATCCAACATCCTCCATTTTTGAGTGGTATT

GCTTGTATTAGTATTGCTCATAAGTAATATGATATTTATAATCCATCGATGTCATGGGTT

CCATTTGGTTATCTTTGGGATGATAAATGACCTACTTAACTCAGTGGTTAGAGTATTGCT

TTCATACGGCGGGAGTCATTGGTTCAAATCCAATAGTAGGTAGAACTTATTAGATACCGG

AGTCAATGGTACCTAATAAGTTTTTCGACCCACCCTCTTTTATTTTTATTGATTTTGTAT

CTTTTTTATTTATTTTATTTTCGTTGTAGCAAAATAGGATTCTGCTTGATTGGATTCACT

CGACAGAATCCTATCAAAATAGGCTTAGAAACAGAACTTCTTTTGATTATTCGAACGCGC

CAACTAGTTAGGAAATCACATTGACAGCCTCTACTCTTGTCCTAGCTCGTCGGAGAGCTA

GATTCGCCTCAATTATTTGTCTCTTTCCTTCAGCTTTTCTCAAATTAGCTTCGGCTATTT

CAAGAGTTTGCTGAGCTTCTTGTGGATCAATGTCACTACCCTTTTCCGCATCATTTACTA

AAATAATGATCTCATTATTTCCTATTCTAGCAAAACCACCCATCAGAGCCATCGTTAACC

ATTGGTCCTTAAAGCGTATTCTCAAAATCCCTATATCTACAGCTGTAGCAATAGGGGCAT

GATTTGGTAATACGCCAATTTGACCACTATTAGTATATAAAATGATTTCTTTCACTTCTG

AATCCCAAACAATTCGATTAGGGGTCAGTACACAAAGATTTAAAGTCATTTCTTCAAATT

GCTCTCCATTTCTAAGTTCATAGCCTTCGCGGTAGCTTCATCGATATTACCTACCAAATA

AAAGGCCTGTTCAGGAAGACCATCTAATTCTCCGGAAAGGATCAATTGAAACCCTCTAAT

GGTTTCTGCTAGACCAACATATTTCCCTGGAGAACCGGTAAATACTTCTGCTACAAAAAA

AGGTTGTGATAAGAAACGCTCAATTTTTCGTGCTCTTGCTACGGTTAAACGATCCTCTTC

GGATAATTCGTCCAACCCAAGGATAGCTATAATGTCCTGAAGCTCTTTGTAACGTTGTAA

AGTTTGCTTAACTCTTTGCGCAGTTTCGTAATGTTCCTCACCAACGATCCGAGGTTGAAG

CATGGTTGACGTTGAATCTAAAGGATCTACTGCTGGATAGATCCCCTTGGCAGCTAATCC

TCTTGATAGTACGGTAGTAGCATCTAAATGTGCAAATGTCGTAGCAGGAGCAGGGTCGGT

CAAATCGTCTGCAGGTACATAAACTGCTTGAATAGAAGTTATGGACCCTTCTTTGGTAGA

AGTAATTCTTTCTTGTAAAGTACCCATTTCGGTACTCAGGGTAGGTTGATAACCGACAGC

GGAAGGCATTCTACCCAATAAGGCCGATACTTCGGATCCTGCTTGGACGAAACGGAAGAT

ATTGTCGATAAATAGAAGTACGTCTTGTTCATTAACATCTCGGAAATATTCCGCCATAGT

TAGGGCAGTCAAACCAACTCTCATACGAGCTCCAGGCGGTTCATTCATCTGGCCGTAAAC

TAGAGCCACTTTTGATTCTGCAATATTTTCTTCATTAATCACTCCAGATTCTTTCATTTC

CATGTAAAGATCATTTCCTTCACGAGTACGTTCACCCACTCCGCCAAATACGGATACGCC

CCCATGGGCTTTGGCAATATTGTTAATCAATTCCATAATGAGTACCGTTTTACCAACTCC

AGCTCCTCCAAATAGTCCGATTTTTCCCCCACGGCGATAAGGGGCTAAAAGATCTACTAC

TTTAATTCCTGTTTCAAAAATAGATAATTTTGTATCTAACTGTATAAAGGCAGGCGCAGA

TCTATGAATAGGAGATGTTGTACGAATATCTACAGGACCTAAATTATCAACAGGCTCTCC

AAGCACGTTGAAAATTCGTCCCAGAGTCGCTCCACCGACCGGAACACTTAGAGGAGCTCC

TGTGTCAATCACTTCCATTCCTCTCATCAGACCATCTGTAGCACTCATAGCTACAGCTCT

AACTCGATTATTTCCTAATAATTGCTGTACCTCACAAGTCACATTAATTGCTTGACTAAC

AGTATCTCGACCTTTAACTACCAGAGCGTTATAAATATTAGGCATCTTGCCCGGCGGAAA

GGCTACATCTAGTACCGGACCGATGATTTGGACGATACGGCCCAGGTTTTGTTTTTCAAG

CGTGGAAACCCCAGAACCAGAAGTAGTAGGATTAATTCTCATAATAATTAATAATAAACA

AAATATGTCGAAATTTTTTTTTGCGAAAATTATCGAATTCAAAATAAAGGTCCGATAGCA

CGGAGATCAGTTAATTCAATAAGAAATGTCAATAAGAAATGGGAGTTAGCACTCGATTTC

GTTGGTACCATTCAATCGAATCCAATTCAATTGTTTACTTATTCATCCACTTGCAATTTT

CAAAATCTTGAAAATTGCAAGTGGATGAATAAGAATCTTGAGAAAGTTTTTCATTTGTCT

ATAATGATAGACAATCCCATCTATATTATAGAAAATATTCTATCGAATTCGAACCTGAAC

TCTATTTACATTAGGATTCATTATTTCTATATCATCGGTGCTTCGTATTTTTTATTTCAG

CATATCGATTTACGCTTACGCCTAGCCTATATTTTTTCTTTTCGTTTTTTTATACCCTTT

CATAGACGAATTCCGCATATTTTCACATCTAGGATTTACATATACAACATATATCACTGT

CAAGAGGGAATTTCTTATTAGTTAGGTTAGGTATTTCGATTCCAAAAAAGGT-AAAAAAA

AATTGGGTTGCGCTATATATATGAAAGAGTATACAATAATGATGTATTTGGTAAATCAAA

TACCATGGTCTAATAATCAAACATTCTGATTAGTTGATAATATTAGTATTAGTTGGGAAG

TTTGTGAAAGATTCCTGTGAAAAGTTTCATTAACGCCTAATTCGTGTCGAGTAGACCTTG

TTGTTGTCAGAATTCTTAATTCATGAGTTGTAGGGAGGGATTTATGTCACCACAAACAGA

GACTAAAGCAAGTGTTGGATTCAAAGCGGGTGTTAAAGAGTACAAATTGACTTATTATAC

TCCTGAATACGAAACCAAAGATACTGATATCTTGGCAGCATTCCGAGTAACTCCTCAACC

TGGAGTTCCGCCTGAAGAAGCAGGGGCTGCGGTAGCTGCCGAATCTTCTACTGGCACATG

GACAACTGTGTGGACCGATGGACTTACCAGCCTTGATCGTTACAAAGGGCGATGCTACCA

TATTGAGCCCGTTCCTGGAGAAGCAGATCAATATATCTGTTATGTAGCTTACCCTTTAGA

CCTTTTTGAAGAAGGTTCTGTTACTAACATGTTTACTTCCATTGTAGGTAATGTATTTGG

GTTCAAAGCCCTGCGTGCTCTACGTCTGGAAGATCTGCGAATCCCTCCTGCTTATGTTAA

AACTTTCCAAGGCCCGCCTCATGGGATCCAAGTTGAGAGAGATAAATTGAACAAGTATGG

TCGTCCCCTGTTGGGATGTACTATTAAACCAAAATTGGGGTTATCTGCTAAAAACTATGG

TAGAGCAGTTTATGAATGTCTTCGCGGTGGACTTGATTTTACCAAAGATGATGAGAACGT

GAACTCCCAGCCATTTATGCGTTGGAGAGATCGTTTCTTATTTTGTGTCGAAGCAATTTA

TAAATCACAGGCTGAAACAGGTGAAATCAAAGGGCATTACTTGAATGCTACTGCGGGTAC

ATGCGAAGAAATGATGAAAAGAGCTGTATTTGCTAGAGAATTGGGAGCTCCTATCGTAAT

GCATGACTACTTAACAGGAGGATTCACTGCAAATACTAGCTTGGCTCATTATTGCCGAGA

TAATGGACTACTTCTTCACATTCACCGTGCAATGCATGCAGTTATTGATAGACAGAAGAA

TCATGGTATACACTTCCGTGTACTAGCTAAAGGGTTACGTATGTCTGGTGGAGATCATAT

TCACTCTGGTACCGTAGTAGGTAAACTTGAAGGGGAAAGAGACATCACTTTGGGCTTTGT

TGATTTACTGCGTGATGATTTTATTGAAAAAGATCGAAGTCGCGGTATTTATTTCACTCA

AGATTGGGTCTCTCTACCAGGTGTTCTGCCCGTGGCTTCAGGGGGTATTCACGTTTGGCA

TATGCCTGCTCTGACCGAGATCTTTGGGGATGATTCCGTACTACAGTTCGGTGGAGGAAC

TATAGGGCACCCTTGGGGTAATGCGCCAGGTGCCGTAGCTAACCGAGTAGCTCTAGAAGC

ATGTGTAAAAGCTCGTAATGAAGGACGTGATCTTGCTTCTGAGGGTAATGTAATTATCCG

TGAGGCTAGCAAATGGAGTCCTGAACTAGCTGCTGCTTGTGAGGTATGGAAAGAGATCAA

ATTTGAGTTTGCCGCAATGGATACTCTATAAGTAAGATAACAAGTAATTACCCTCCGTTC

TCTTAATTGAAAACTCGGCCCAATCTTTTACTAAAAGGATTGAGCCGAATACAAAGATTC

TATTGCATGTATTTTGGCTAAATCTATACTTCTCCAGATATACAAGATTTGAAATACAAA

ATCTAAGACTAAATCCAAATCGAAGACTCAAATGTTTCTATTGTTGTTTTGGATCCACAA

TTAATCCTATGGATCTTTAGGATTGGTATATTTTTTCTATATCCTGTAGTTTCCCTGAAT

CGAGCGAAGTATCACAAATCTTTCTACCCATCCTGTATATTGTCCTTTTCGTTCCATGTT

GGAATAGAACCTTAATTTCTTACTTGTTATTAGTTAGTTATTAGACGAGATTTTACGAA-

AAAAAATTCTTTCTAGGAGAGAACAAATC-T--TTTTTTTTTGATGCGAAGACATAGGAG

AAACTACTCTTTTTCATTATATTTAGAATGAAAAGGGATTCCATCATATTCATATATAGT

GAAGTCTTACCCCGGATTCCCACAAAAGAAAATTCTTTTTCACAGTTAATAATGATAGTG

AGTGGATTCCTATGTTTATTCTGATAGGAAATGAAAATATTCAAATAAAGAATTTTGGAT

CGAATGACTATTCATCTATTGTATTTTTATGCAAACAAATAGGGGGGAAGAAAACTCTAT

GGAAAGGTGGTGGTTTAATTCGATGGTGTTTAAGAAGGAGTTAGAACGCAGGTATGGAAT

AAATAAATCAATGGACAATCTTGGTCCTATTGAAAATACTAGTCAAAGTGAAGATACGAA

TAGAAAAGCTAAAAACATTCATAGTTGGAGGGGTCGTGACAATTCTAGTTACAGTAATGT

TGATCATTTATTCGGCATCAAAGACATTCGGAATTTCATCTCTGATGATACTTTTTTAGT

TAGAGATAGTAATGGAGACAGTTATTCTATCTATTTTGATATTGAAAATCAGATTTTTGA

AATTGACAATGATCATTCTTTTCTGAGTGAACTAGAAAGTTCTTTTTATAGTAATCGGAA

TTCTAGTTATCTGAATAATGGATCTACGAGTGAAGATCCCTACTACAATCGTTCCATGTA

TGATACTCAATATAGTTGGAATAATCACATTAATAGTTGTATTGACAGTTATCTTCAGTC

TCAAATCTATATGGATACGTCCATTGTAAGTGATAGTAGTGACAGTTACATTTCTAGGTG

TATTTTTGGTAAACATACAAATAGTAGTGAAAGCGCGAGGTCCGGTATACGAACCCACAC

GAAGAGTAGTGATTTAACTCTAAGAGAAAGGTCTAATGATCTCGATGTAACTCAAAAATA

CAGGCATTTGTGGGTTCAATGCGAAAATTGTTATGAATTAAATTATAAGAAATTTTTGAA

ATCAAAAACAAATATTTGTGAACAATGTGGATATCATTTGAAAATGAGTAGTTCAGATAG

AATCGAACTTTCGATCGATCCCGGTACTTGGCATCCTATGGATGAAGACATGGTCTCTCT

GGATCCCATTGGATTTCATTCGGAGGAGGAGCCTTATAAAGATCGTATTGATTCTTATCA

AAGAAAGACAGGATTAACTGAGGCTGTTCAAACAGGCATAGGTCAACTAAATGGTATTCC

CGTAGCAATTGGGGTTATGGATTTTCAGTTTATGGGGGGTAGTATGGGATCCGTAGTCGG

GGAGAAAATCACCCGTTTGATTGAGTACGCTACCGATCAATTTCTACCTCTTATTGTAGT

GTGCGCTTCCGGGGGGGCACGCATGCAAGAAGGAAGTTTGAGCTTGATGCAAATGGCTAA

AATATCGTCTGCTTTATATGATTATCAATCAAATAAAAAGTTATTTTATGTATCAATCCT

TACATCTCCTACTACTGGTGGGGTAACAGCTAGTTTTGGTATGTTGGGAGATATCATTAT

TGCTGAACCCAATTCCTATATTGCATTTGCGGGTAAAAGAGTAATTGAACAAACATTGAA

TAAAACAGTACCTGAAGGTTCACAAGCGGCTGAATATTTATTCCAGAAGGGCTTATTTGA

CCTAATTGTACCACGTAATCCTTTAAAAAGCGTTCTGAGTGAGTTATTTAAGCTCCACGC

TTTCTTTCCTTTGAATTAAATTCAATCAAGTAGAGCACACAAAATTCAATTAGTTTATTT

GTAGCAAACAAGTAGTTAGTTTATAAGAATCAAAGTAAATAAGAATGGAGTTTTCTTTGA

TGACCTAAGATCTAATTGTAGAAAGAATAAAAAGTTGCGGATAACTCTTTTTTTTACCTA

GAATCCCGATTACTAATTAAGAAGTCTCTATCAACAAGATAAAAGAGTGAATTCTTCCTT

TCGTGAAATTAGGCAAATAAAATGAATTTCGTCTTATG-------TATATAATCAAATAG

AGAAAAGATAGATATATAGTTTTTTATCTTTCTCTATCTCCCGAAAACCCCATTTGCACT

AAAAATTCCTGTTGGGTCGCATTCTAACGAATCTTTCGATAATCTGTAAGAAACTCTTTC

TTTATTAAAAATTCGAAGACAAGAACAAAAGACAAAGAAATGAAGAAAAATAATAAAGTG

AATTATAATACATATCTTTCATGTAGAAAGATGAATAAGTCCATTTATTTAGTTCTACAT

TCCTTGGACTTATTCTATATACTCACTTAGATATATAGATACTTATTTCTAT-ACTAAGA

ATTTGAAATTTAATTAATTAATAATAATTACAATTCTTAATTATAATTATTATAAGATAT

TTATTTTTTATAAAAAATAAATAATAGCAGGTACAAATAGTAAATCGAGGTACCCATTTT

ATGACAACTTTCAATTTCCCCTCTATTTTTGTGCCTTTAGTAGGCCTAGTATTTCCGGCA

ATTGCAATGGCTTCTTTATCTCTTCATGTTCAAAAAAACAAGATTGTTTAGATCTGATGG

GACCCGATCTCATCCG--TTTTTTTTTCAAAACTTAGACTTGTAGCATAACACAGATATC

TATTTCGAAAAATATGGTCTAACGTGTAATTTCCGCCGAACATAAAGGAAAAAGTTCTTA

TGCCTGCATAAAAGGATCTATGGGTAAATGAATTCTAGCTAGTTTCAAATAGATCAGGAT

CGCTGGATGGCTAAAATGTAAAGTCGGTGGATCTATAGGTATATCAATATGTATAGTGGG

CTCATATGAAGGGTATGTTATTATTTTAGATCTAACCAATTTGATGAATTACTCCTAAAG

---------------------GTTCACATCAAACTAGTGCTAGTTGATGAGAGTTACTTC

GGAAACAAAAA-AAAGTAAAGTCAAATTCATTTGGGGTATTCTCTCAATTCCAATAAAAT

GCAATCAGATCAAGTATGAGTTGGCGATCAGAAGATATATGGATAGAACTTATAACGGGG

TCTCGAAAACTAAGTAATTTATGCTGGGCCCTTATCCTTTTTTTAGGTTCATTAGGATTC

TTATTGGTTGGAACTTCCAGTTATCTTGGTAGAAATTTGATATCTTTTTTTCCGTCTCAG

CAAATCATTTTTTTTCCACAAGGGATCGTGATGTCTTTCTACGGGATCGCGGGTCTCTTT

ATTAGTTCCTATTTGTGGTGCACAATTTCCTGGAATGTAGGTAGTGGTTATGATCGATTC

GATAGAAAGGAAGGGATAGTGTGTATTTTTCGTTGGGGATTTCCTGGAAAAAATCGTCGC

ATATTCCTCCGATTCCTTATAAAGGATATTCAGTCCGTTAGAATAGAAGTTAAAGAGGGT

ATTTATGCTCGTCGTGTCCTTTATATGGATATCAGAGGCCAGGGGGCCATTCCCTTGACC

CGTACTGATGAGAATTTGACTCCACGAGAAATTGAACAAAAAGCCGCCGAATTGGCCTAT

TTCTTGCGCGTACCAATTGAAGTCTTTTGAGAAATGTAAATATGGGCTGAAGAATGAATG

CTTTCTCAGCAGGAGGGCAAAATGAAAGAATCCTC--TTTTTTTTCTATAACATAACTTA

ACTGAAGTTTTGTCAGAACGTTAAGTCGAGCCAAAGCCGACATATATGGAACAACCATAA

AAGAAAACTCTTTTTGTGGCGTATACAAATCCACGCAACTCAATTCAACAACAAGTATAA

CAAATTGAACTAATAGATTCAATTCATCTCATATATCAAACGATTTCGAAAGAAAGAAAT

TTAAGTTCAATATTTGTTGGAATTGATACTTTAGATGCAGATAAATCATATCTTGTAAAT

TATTTC-TTTTTGTCAATCGACCTTTTTTTATCCTTTCTTTTGTATTCCTTAATAACCAA

CAATTGGTTTTCTTATAATGATAACTGGACAATTTCTGTCTTGTTTTGCTACTCATTTTT

TTTTGATCATCACAATATCTTTCTCTCAATTATTCTATTCCTGGCTATGGGTAATCGGTG

GAATTTTTTCGAAATATTGGATATTTTGATAGAAAAGGAATTCTTTCGTCTCAAAATCTC

AATAATATTCATTCCTTAAAGTGCTTCTTTCGTTCATTCGGAGACACTTGTTTCGAATTT

GACCAATTGAGATATCTGAAAACAATATTTTTTATTATTTCTTCATTCAAATTCGAAGTG

GCATCTTAGTCTATTTCTGTATTCTTTCTAGATTCAAACAAAATCACAAATAAAATAGAT

TCATAGGTTTGATATCTTGTATAGAACTCATTGGTGAAAGAAATATTCGATAGATCACAT

AGAGCCGACGAATGAGGTGGGTTGATTAACAATTCACAGATGAAAAAATGGCAAAAAATA

AAGCATTCACTCCTCTTTTGTATTTTGCATCTATAGTATTTTTGCCCTGGTGGATTTCTC

TCTCATTTACGAAAAGTATGGAATCTTGGGTTACTAATTGGTGGAATACTGGGCAATCTG

AAATTTTTTTGAATGATATTCAAGAAAAGAGTATTCTAGAAAAGTTCATAGAATTAGAGG

AAATCCTCTTCTTGGACGAAATGATCAAGGAATACTCGGAGACACATTTACAAAAGCTTG

GTATAGGAATCCACAAAGAAACGATCCAATTAATCAAGATACACAATGAGGATCGTATCC

ATACGATTTTGCACTTCTCGACAAATATAATCTGTTTTGTTATTCTAAGTGGTTATTCTG

TTTTTGGTAATGAAGAACTTGTTATTCTTAACTCTTGGGCTCAGGAATTCCTATATAACT

TAAGCGACACAGTAAAAGCTTTTTCAATTCTTTTATTAACGGATTTATGTATCGGATTCC

ATTCACCCCACGGTTGGGAACTAATGATTGGCTCTGTCTACAAAGATTTTGGATTTGTTC

ATAATGATCAAATTATATCTGGTCTTGTTTCCACTTTTCCAGTCATTCTAGATACTATTT

TAAAATATTGGATTTTCCGTTATTTAAATCGTGTATCTCCGTCACTTGTAGTTATTTATC

ATTCAATGAATGACTGATAAATGATCCACCGATATTAATCTAATCCAATTAGAATGTTTG

TTACTTTGTAGTTCTACATAAGCATTAAAAATCGTACTTACTCTTTAGATTTCTAACCAT

CCGGGGAATTCATCCTATATTATTCCAGTAAAATGATTCCAGTAAATAGCAGAATCGTGG

ATAGGGAACTATACTAGCAACCTACTCAATTTATTGTAGAAATTTTTGGATCAATTATTA

GACCATGCAAACTAGAAATACTTTTTCTTGGATAAAGGAACAGATTACTCGATCTATTTC

CGTATCGCTCATGATATATATAATAACTCGGACATCCATTTCAAGTGCATATCCCATTTT

TGCACAGCAGGGTTATGAAAATCCACGAGAAGCGACTGGGCGTATTGTATGTGCCAATTG

CCATTTAGCTAATAAGCCCGTGGATATTGAGGTTCCACAAGCGGTACTTCCTGATACTGT

ATTTGAAGCAGTTGTTCGAATTCCTTATGATAAGCAACTGAAACAAGTTCTTGCTAATGG

TAAGAAAGGAGGTTTGAATGTAGGGGCTGTTCTTATTTTACCGGAGGGGTTTGAATTAGC

CCCTACCGATCGTATTTCTCCCGAGATGAAAGAAAAGATAGGCAATTTGTCTTTTCAGAG

CTATCGCCCCAATAAAAAAAATATTCTTGTGATAGGGCCTGTGCCTGGTCAGAAATATAG

TGAAATCACCTTTCCTATTCTTTCCCCCGACCCTGCTACTAAGAAAGATGTTCACTTCTT

AAAATATCCTATATACGTAGGCGGGAACAGGGGAAGGGGTCAGATTTATCCCGACGGAAG

CAAGAGTAACAATACAGTTTATAATGCTACAGCAGCGGGTATAGTAAGCAAAATCATACG

AAAAGAAAAGGGGGGATATGAAATATCCATAACAGATCCATCGGATGGACGTCAAGTGGT

TGATATTATCCCTCCAGGACCAGAACTTCTTGTTTCAGAGGGTGAATCCATCCAATTTGA

TCAACCATTAACGAGTAATCCTAACGTGGGCGGATTTGGTCAGGGAGATGCAGAAATAGT

ACTTCAAGATCCATTACGTGTCCAAGGCCTTTTGTTCTTCTTGGCATCTGTTATTTTGGC

ACAAATCTTTTTGGTTCTTAAAAAGAAACAGTTTGAGAAGGTTCAATTGGCCGAAATGAA

TTTCTAGACTCGCGGATTTATCGACATCCAGTTCGTAAAAAGAACCAAATTCTTGTTGTC

GATTATGATTTTGTATGATAAAAAAA-AATGAAATTATGAAAAACCTTTTTCTTGTTTAT

ACTC-TTTTTCTACGGAATTCCTTGTACGGCATTCCGAGTCATAATAGGTAGATTTTTTT

TGAAGAAGACTATTTATTTGACTTTACCCCCTCTTTCTTTGTTTTTTTTAGCCAAATTGA

AGTGGTGCACCTATGTTACTATTGCCAGATTTCAATGTCATAAAATTGGACTAGATATTA

GCA-TAAGTAAGCGGGGAGCAAATAATTCTAGGAGGGATTATTCGTCTTCCTAGTCTTCG

ACACAAGA-AAGGGGTGTAGAAAATTCCTTTTCTTGTGTCGAAAGAGTAATGATTTTTGA

TCCTGTTCGTCAAAAATGCCTAGTCTTGGTTTCGGTTTTTCGAGATGTATCAGAACTTTT

TCGATTTTTTTCATATAATAATATAAAATAATATAATAAGTAGTGGACAAACAAAAAAAA

ACACAAAAAAAGAGGGAATTTTATTGATTAAATACAATGAACTTCTAAAAAATTTGAATT

TGGCTGAGATACTCAAATAAATAGAGTAATAGAAAGTATTTGTACGATATCTAGTCGACC

AAAATATATATCATCCAGGAAGTTGAGTGATTCCCCCTTTCTTCTAACTTGGAAAGTACC

C-ATAGATACTGTCAAGTAACAGGTGTTCTGAATCAATCAATGAAGTTCATTTTTCAAAA

GCATCATCAGAAAAAGTTTTTTGAAACAGCAGAAAAATAAATCTACTTTGTCATTTAGAC

GAAAAAAA-GACTCTGATTCTTAAGAACCCAACGGGCCTTTTCCCCTCGAATCAGACAAA

CAAAGAAGGGAATCCCGTTGAGTTCTTACGCTTTCATGTCTACAACTCAATTCATCCGAT

TACTACAGGGATGAACCCAATCCGGAATATGAACCATAAAAGAAAATACCTATTAAACCA

ATCACAAGAATACCAGTTACAGTACCTATTATCCAAAGAGGAATCCTTCCAGTAGTATCG

GCCATTTACTCTACTTCCCTCCAATTTCATCAAGTGGTCATGCTACAGACATAAACAGTC

ATGGATAATTATGAGATGAGATCCTTCCGAATGGGCTAAGAGAATGCCTAGAATTCTTAT

TTCTTTTCTTTCGTTTTCCTAATTGAAGAAATAATTGGAAAATAAAACAGCAAGTACAAA

AATGAGTAATAACCCCCAGTAGAGACTGGTACGATTCAATTCAACATTTTGTTCGTTCGG

GTTTGATTGTGTCATAGCTCTATAATTCGGATTAGGTTTATCGTTGGATGAACTGCATTG

CTGATATTGACCCCAAAAAAAAGACGGTAGGTACAGCTAGGCCGTGAACAGCCAACCATC

GTACTGTAAAAATTGGATAGGTTCGATCTATAGTCATTAGATTAGGGCCTCCTAAAACGA

TCTACTAAATTCATCGAGTTGTTCCAAAGGATCAAAACGGCCAGTTATTAATGGAATTCC

TTGTCGGCTCTCTGTAAAATATTCGTTTGGCCGAGGGCTTCCAAATACATCGTAAGCTAA

ACCGGTACTGACGAATAACCAACCCGCAATGAATAGGGAAGGTATAGTAATGCTATGAAT

GACCCAGTATCGAATACTGGTAATAATATCAGCAAAAGAACGTTCTCCTGTGCTTCCAGA

CATGCTCAGCTCCACATATTCTTGTACAGTCAAAGGGGATCGATTCCGTAAAAGATGAGA

TCAGTAAAAGGCAATCACTGAAATTGCATCCTTGTAGGATCGTCAATATTGTACCGAGGG

CGTCTTTAGAGTATACCGAATCAGTATAACTATCCTTCTTCTGACACAGCAACGCAATTT

GAATCAGTATCGAATCGAAAGGAAGCTTCCTTTCCTTTACCCGTTGATGTAAAATGATGC

TCTATATTAATAGAAAATTCTTACAATGAAAGAGATTATCATATTCCCACAATTTAAGTA

GATGCGCGAGATCTAGAAATTTCCTTTTCGTAGTTGTAGAAGCAGTTTTTTTTGTTGGAA

TCCTTTTTTTAATTGGTTAATCGTCCAGTAACAAATAAGAGTAGTAGAGCGTATTCGATG

AAAGAAAGCGAAGAAAGAATAAAATAATTGGAATCCATAGTTGTGATGCATTGTTGTATT

GGATCGAGATACAAATCTTGATCTAGCTACAAGGATGAGGCTTTATTTAAAAATATGGAA

AGCCAAATTGTAAAAACTAAAAACGATAATAGAAATTACTAGTTTTAGAATCTAATTGGA

TTTTTTTCTAGTGATCCATGTGATACCTTTTTTCTTCACATTCATTCAAGATATTATGGG

AATGAACCTATTACTGAATTTAATGAATTAAACTTAATTAAGGTAAAAAAAGTTTTATAA

GGTCACTGTTCACTCTAAAATAGAAAATGAATTCGATACAATTCAAAAATCGATTTTTGA

ATTGTATTCCATAAAAATTTTATTCACAAATAAAAATTCAAAAAGAGTTTCA-TTTTTGA

GTGAAGTTACACAATCCAGTTCGTATTATTAGTTTATGCTCAATGAATCGGTTGATAGGA

ATCGCGAGATGGATAAATGTTACAAATGATGAATCAATTTCGTTTTATATGCGCCTGTCA

CTTTATCTTTGTTCGTGCCATATATAATGATAGATGAATCAAAAACTTTCAATTGAACTT

ATTCTTTCAATTGGTATTTTTGCGTATCCTCCTATTTTACAAAAATAGAAATTTAGGTAA

ATGCTTTCTAAACATATGTATAAAAATAACATATTTCATTTAGCTCCTTCATGCTTACTA

TAACTAGTTATTTCGGTTTTTTACTAGCGGCTTTAACTATAACTTCAGCTCTATTTATTG

GTCTGAGCAAGATACGACTTATTTGAAATTTATATTTGAAAGAAATAATTCCTAAAAGAA

ATCTTTCTGTGAGATTCGGTGTATTCTATAGTTACTTACTGCGTAAATTCTGGGTCATTG

AGATTCACGTCAATTCGGATTAATATTTAGGTATAGATATTACCTTTTTTTTTTCTCCTT

TTCAAAAAATTGAAATGATTGAAGTTTTTCTATTTGGAATCGTGTTAGGTCTAATTCCTA

TTACTTTGGCTGGATTATTCGTAACTGCATATTTACAATACAGGCGTGGTGATCAGTTGG

ACCTTTGATTAATTAACATTTCTTTTTTTGATTGACCTCCTCCTCCTTTCTTTAATCCAC

AGGAGGTCAAATTCTGATTGCTGTGCAAGTGAATGAATCTATTTCATTCTAATTCGATCT

ACGAAGAAAAAATCACGCTCTGTAGGATTTGAACCTACGACATCGGGTTTTGGAGACCCA

CGTTCTACCGAACTGAACTAAGAGCGCTTTCTTATCAGAATAAGAGAAGACTGTAAAGAA

AAGGATTATTTTTGTAACCCTAATCCATTTTCATTTTGTCTGCATATACTATATAGTTTC

AAAAAAATGAAAGATTCTGCCCAATTTGAATGGATCTCAGTTGATTCCTCGTTACTGCTC

AAAGGAGCAGTAATAGGTAGGGATGACAGGATTTGAACCCGTGACATTTTGTACCCAAAA

CAAACGCGCTACCAAGCTGCGCCACATCCCTTCAATTGTTCTACAGTGTCATTGTAGAGA

ATTCCTGTCTTGTTTTCCACATCCTTAGTTGTTCCATTGATATACACAATTTTTCTGCCC

ATTTCGTATTTTTGGTTTTAATAAGAAAAGTAAAAAACTTATTATATATATACGAAATAT

AGAACCCATTGTAAAAAAAATGAGTATTTTTCGGAAATACTCGCTACGAGGGGATC-TTT

TTTTTTCTGTTTTAAGAAAAAGAAAATCTTATGGATCATTGTACATTTCAATTTGAATTA

GGGATTCCGTGTACAACTATAAGTGGTCCTTAACTACATATCTATCTGATCATATATGCA

TTATCTTTATCTTTATGTATTACAATAAATAAAAGAAGGAGGGTTTTCAATGCGAGATCT

AAAAACATATCTCTCCGTTGCACCAGTACTAAGTACGCTATGGTTCGGGGCTTTAGCAGG

TCTATTGATAGAGATTAATCGTTTTTTCCCGGATGCGTTGACATTCCCCTTTTTTTCATT

CTAGTTATTGACATGGGAAGGAATGAAGAAGATTAGAGATACAATCAAATATCTGTGACT

AATACTAATCCCCCC-TTTTTCTCTTTTTTCCCTTTTTAGAATAAGGGAGGAAAGAGAAA

GAATAAAAGTAGATCCAACTTCTGCGAGACTCGGGTTCAAGTTCGAATTAAATGAATAAT

GAATATTAATAATAGAGGAATGGGGGTAGAGTAGAAAATGCAGATCTAGGGCAAGAATAC

AAGAACAAGATCTTTAACTGAAATACCGTACTTCAATTTTAAATATAGTTTAGAAATCCG

TTGTCTTACTTATTATTTACTATGGCTTTGATTTATTATTACTTTATTGATTTTGATCTT

TTAGAATTGGATTTCAAGTTAGTAACTTCTATTTTTTCCTTCCTTTTCTTCGTTTCGAAT

CGAAAATAAAAGAATTGAGTAAATCAAAAATCCAAAGGAGGTTCATGGCTAAGGGGAAAG

ATGCGCGAGTAACGGTGATTTTGGAATGTACCAGTTGTGTCCGAAATGGTGTTAAGAAGG

TATCAAGGGGCATTTCCAGATATATTACTCAAAAGAACCGGCACAATACGCCTAATCGAT

TAGAATTGAGAAAATTCTGTCCCTATTGTTACAAACATATGATTCATGGGGAAATAAAGA

AATAGATCGAACAGAGTATATCTTAGTCTTTCAAGGAAGGGTAAAAAATGACATTATATA

TAACATATTTAAATAGAA-AAA-AAATCCTATTTGGGGTTAAAATGAATTACAATTAAGA

AAGAAAAATAGGATTTTGGGATAAGAAATAAACTAAACAAAAAAACACAAAAAACCATGG

ATAAATCCAAGCGACCTTTTCTTAAATCCAAGCGGTCTTTTCGTAGGCGTTTGCCCCCGA

TTCAATCGGGGGATCGAATTGATTATAGAAACATGAGTTTAATTAGTCGATTTATTAGTG

AACAAGGAAAAATATTATCTAGACGAGTGAATAGATTGACCTTGAAACAACAACGATTAA

TTACTATTGCTATAAAACAAGCTCGTATTTTATCTTTGTTACCTTTTCTCAATAATGAGA

AACAATTTGAAAGAACCGAGTCGACCGCTAGAACTACTGGTCTTAGAACCAGAAATAAAT

AGGCTTATTCTTTGTTCACTTGAATTAGAATTCCAATCAGAACTCAAACACAGATTGTTG

TTTTGTTCGACAAATCCGGGAATCCAGATTTTATTATCGTGTCGTAAG--AAAAAAAACG

AATCGGAAAAT-AAAAATTTAAATGTGTTCATTCATTTTGACTACTTTAGCATATTTTCT

CATAGTAATTTTGACTCTACCTTCCCGGAGTTCATTCTCCGGGGAACTCCGTTTAAATTA

TTCCGGTGGATTCTTTCCAATCTACTTCTTTTATGATCTCGTTGGAAATCATATAAAGAC

AATTCCTATTTGATATAGCTATTTGTGCAAGTATTTTACGATTAAGAAGCAACTGTCTCT

TGTATAGATCATGTATTAATTTACTATAACTATAAGATACTCCCTTTTCGCGAATTACTG

CGTTTATCCGAGTGATCCACAAACGACGAAAATTTCTCTTTTGCTTATCCCTATCCCGAT

GAGCCGAAACCAAAGCTCTTATTTTCTGTTGAGTAATAGTTCGAGTAAGTCTTGAATGAG

CCCCTCGAAAGCTTGATGCAAATAAACGAATTTTTGTTCTACGTCTCCGAGCTATATATC

CGCGTTTAATTCTGGTCATTGAATAAATGAAACTTTGACGAATAACTAATTGATTTCCTT

TCTTTCAGTTATTCTTTTCCCCTTTCCTGGTCTATTAATAACCAAACGGATTTTGCCAAT

GTAT--AAAAAAAAAATTCCAATGGCTTTGGCTACTATAACCTTCCCGACCACGATTTTT

TC-TTTTTTTTAGGTATTTCACTGCGAAATACGAAAGAAATAAGAAATTTTATTCTTCTA

AGTGTGAAAAATATAGTAAAAAGAAATATAAATTAAATGGATAAAGAAATAGTGGGTTCC

GTCGTTTCTATGGTTACTTCTTAAACGGTGAGGTCTTCTCTATACACCGGAGCCTTTACT

TCATTTAATCAATGTTATTGGTAACTTGTATAGTTCACACCACACTCTTTGGCTCTACCC

ATGAATTATCCAGTAATAGGTCTTTCACAATGAGATCCACCTATACAGTAACGGTATTTA

ATTAGGAAAGTTAGCTGGGTAGCTGACCCTCTTAGTCCGTTCTTGACAGAGTGGGAGCTT

CATTTTTCTGTTTTTGAAATTGAAATAAGATTTCCTCCGCTTAATAGATAACCATTTGCT

ACCAATGGAGAATTGCTTCTCATCTTAAATTCAGGTGATTGGATTTGCACCAATGGAAAC

CATAAACTTCATACACAATAGAGGGATCGATTTGCTTATTTTTAGATAGTGAATGGGGTT

CTTTCTTCCATTCTATCCTATTTACTGGTACTGATCATTGATACTGGAAAGCGGTTTTCT

TGCTT-TTTTTTGTGCCAGCTCATGATCTAAACGAGTCGCACATACACCCTAGTACATGT

TCCTCGACGCTGAGGACATCCCCGAAGAGCGGGGGATTTCGTGACATTTCGAATTGGCTG

TCTTGTATTTCTAATAAGTTGTTTAATAGTTGGCATGTTGAATCATATACATAATGGGCT

GGTTTAGATTGATCCTAACCGGATGATTATGAATTATTTCTATTTAATAGAATATTAAAC

TCGTAGATAAAATCTCAAATCACGGATTTTTATAAAATCCATCTTATTTTCATTCAACTG

CTACAAGATCAACAATTCCATAAGCTTGGGCTTCTGTTGCTGACATAAAAACATCTCTTT

CCATGTCTTCAGATACAACCCATAAGGGTTTGCCCGTTCTTTGTACATAAACCTTTGTGA

GGGTTTCGCGTAGTTTCAGCAGTTCTTCCGCTTCCAGGACAAATTCTCCCGTTTGTGCCT

CATAAAAAGAACTAGCAGGTTGATGGATCATTACCCTGATGATATAACAGTTCTCTATCT

CGCGTGATGAAACGAAGAGAAAAGAAAGAAAGATAAAGAATAATAGGAAAA-AA-AAGAT

AGAATTGAACAACCGTACGGGCATTATCTTTTGTGCATTGCATACGGCTCTACAATAAAA

TTGACCCTTACCTTCCATTGAAGAAAGAGAAAAATAGAATCTATCAGACCCAGATGGATA

AATGATCAAATTGCCACCCTTCCTTTCAGAGGAGTTAAAAAATACTATGATGGCTCCGTT

GCTTTCTATTTTTAAATTGATTCTTTTTTTTGTCTTTGATTCAGCAATCCCAAAGTTTCT

TTTTGATCCAATCAAATAAGGAAAAATCTTGTTTTTTTTTCGCCCTCTTTTTTTATAACA

TAAATATTGTTAAGAGCCCTTCGATGTGAAAACAAAAAAGTTTGTGACGCTGAACTGGAC

TCCCGATAGATAAGAGAAATCGGAAATACCTTTTATCTCATACTACTCTCTCGATACATA

ATCGAATCTTTTGAAAAAAAAACAAGACAAAAATTTTGCATATCGAATTCGAAGTGCCAT

GCTATTATTACTTAATATTCATATGGCGAAGGCATAGTCTTCTTTTTTCTCTCAAAT--A

AAAAAACCTCATTGGCGCCAAGCGTGAGGGAATGCTAGACGTTTGGTAATTTCTCCTCCA

ACCAAGATAAAAGATCCCATTGATGCGGCTAATCCCATGCATATTGTATGGACATCTGGT

CGCACAAATTGCATAGTATCGTAAACAGCTACTCCAGGTATTACCCATCCGCCAGGAGAG

TTTATAAACAAATACAGATCTTTGGTATCATCCTCGATACTGAGATATACCATAAGACCA

ATAAGTTGATTCGAGAGCTCGCTATCAACTTCTTGGCCTAAAAAAAGTAATCTTTCTCGA

TAAAGTCGGTTGATTAGGGTAAAATTGTATCCCTTAGGAACCGTACATGCACCTTTTGAC

GCATACGGTTCAAAAAATAATTGCG--AAAAAAAAAGAATCAATGTATAGATTCAAGTCC

TCTTTCTTTGTTCCTATTCTTTTTTCATAGCAGGTTTTTTCTGACTTCTAATGAAAGGAC

TTTTTCTTCGATTTTTCAATAAAGACGAATTTGAACTTCTTTCTTCCTTAGAATAGAAAA

AAAGTCACTAAACTTATCGAATTAACTTCTCATTGATGTATTGTTTCATCGAGATTCAAT

CCAAATCACGATGGTATTTTCTTGTTCCTGAATGGGTCTCTTTCATCTTTTTAGGTTTAT

GCTCTACTCCGGGTAAAGATCCGCCCGATTTTGATTTGCACATATAGGACAAATCTTCCC

ATTACCATTTCTTTTTGTTATGACTTTCTTTTTTTTTTCAATTCATTTCATACCTTTCAC

CAAGTATTTAGTTTGAGATTCCCTCGCTTGACAAATAGGATCTCTTTACAAATACCAAAC

AGGAATCATTTATGATACAAGTAGTAATCATAGATATATTACCAATTGGGTTTTTTCTAA

ACGGAGCCTGGATACTTCATTTTTTAGTCCAACCAAGCCAACCATAAATTATTCTAATTG

ATAATAGTAATGTGAATCCCCCCAAACAATGGATCTAATTGCGCTTCACGCTCCAAATTT

TTGATGATTCAATTTATCTTTC-TTGGGCGAAACAGAGGATATCTCGATCGGGGGAGAGA

ACGGGGAAATCCCATATGACCCAATATATCTGACAAGTCGCACTATACGTCAACCCAAGC

TGCATCTTCCTCTCCAGGACTTCGGAAAGGTACTTTTGGAACACCAATAGGCATTAATTG

AAAGAAAAAAGAACTAAGTACTATATTTTACTTTGATGTGGAAACGTAACAACATTATTT

TATTGTCTTTATAATATTGGTTTTATCGTATTTATTTTATCCATAGATTAGAAAAATTCA

TAAAGAAAGACAAAAGAAGAAATAAAGGAAAATTTTGACGAATAGGGCCTTCTAATGAGG

AATAAGGAAGGACACATTTACTGATAGAAAATGGTATCAACCACCCATTGCGTATTGGTA

CTTATCGGGTATAGAATAAATCTGCTTCTCTTTGTTCCTACGAATAGAATTGTTTCATTA

TTACCAATAGAATAGAACAAATAGTAACCCTTGTTCAGTGGATTATTTCAGAACAAGGGG

AGTCCATAGAATAGTCATAGTATAGCTTTTCCAATGCAATAAAGTTACGTAGTGTCTATT

TATCTTTGATAAAGAGGTATTTTCCATGGGTTTACCTTGGTATCGTGTTCATACCGTTGT

ATTGAATGATCCCGGTCGGTTGCTTTCCGTTCATATAATGCATACAGCTCTGGTTGCTGG

TTGGGCAGGTTCGATGGCTCTGTATGAATTAGCAGTTTTTGATCCTTCTGACCCTGTTCT

TGATCCAATGTGGAGACAGGGTATGTTTGTTATACCTTTCATGACTCGTTTAGGAATAAC

CAATTCATGGGGAGGTTGGAGTATCACAGGAGGGACTGTAACGAATCCGGGGATTTGGAG

TTACGAAGGTGTAGCTGGGGCACATATTGTGTTTTCGGGCTTATGCTTTTTGGCAGCTAT

CTGGCATTGGGTCTATTGGGATCTAGAAATATTTTGTGATGAACGTACAGGAAAACCTTC

TTTGGATTTGCCCAAGATCTTTGGAATTCATTTATTTCTCTCAGGGGTGGCTTGCTTTGG

TTTTGGTGCATTTCATGTAACAGGCTTGTATGGTCCTGGAATATGGGTGTCCGATCCTTA

TGGACTAACGGGAAAAGTACAACCTGTAAATCCGGCGTGGGGCGTGGAAGGTTTTGATCC

TTTTGTTCCGGGAGGAATAGCTTCTCATCATATTGCAGCAGGGACATTGGGCATATTAGC

GGGTCTATTCCATCTTAGCGTCCGCCCGCCACAACGTCTATACAAAGGATTACGTATGGG

AAATATTGAAACCGTACTTTCCAGTAGTATCGCGGCTGTCTTTTTTGCTGCTTTTGTTGT

TGCTGGAACTATGTGGTATGGTTCAGCAACTACCCCCATCGAATTATTTGGGCCCACTCG

TTATCAATGGGATCAGGGTTACTTCCAGCAAGAGATATACCGAAGAGTTAGTGCTGGGCT

AGCAGAAAATCAAAGTTTATCAGAAGCCTGGTCTAAAATTCCTGAAAAATTAGCTTTTTA

TGATTATATCGGCAATAATCCGGCAAAAGGAGGATTATTCAGGGCAGGTTCAATGGATAA

CGGAGATGGAATAGCGGTTGGATGGTTAGGACACCCTATCTTTAGAGATAAAGAAGGGCG

TGAACTTTTTGTACGTCGTATGCCTACTTTTTTTGAAACATTTCCAGTCGTTTTGGTAGA

CGGCGACGGAATTGTTAGAGCTGATGTTCCTTTTAGAAGGGCAGAATCAAAGTATAGTGT

TGAACAAGTAGGTGTAACTGTTGAGTTCTACGGCGGCGAACTCAATGGAGTCAGTTATAG

TGATCCTGCTACTGTGAAAAAATATGCTAGACGCGCTCAATTGGGTGAAATTTTTGAATT

AGATCGCGCTACTTTGAAATCCGATGGTGTTTTTCGTAGCAGTCCAAGGGGTTGGTTTAC

TTTTGGGCATGCTTCGTTTGCTTTGCTCTTCTTCTTCGGACACATTTGGCATGGTGCTAG

AACCTTGTTCAGAGATGTTTTTGCCGGTATTGACCCAGATTTGGATGCTCAAGTAGAGTT

TGGAGCATTCCAAAAACTTGGGGATCCAACGACAAGAAGACAGGTAGTCTGATACAAGAC

TGCTTTGGTATCTTTCGCCTCTATTTTCTTTTTTGGGGGGAATTTTACATAGAGTACCGG

AGTTGATTTGAATCACTGCTTTTTTGACTCTTGCTCTTTCTTTATCCGAGAGATGATTCC

CAAAGAAACAAAAAACAAACAGGTATGGAAGCTATAATTGTAAACCACGATCGAATCTAT

GGAAGCATTGGTTTATACATTCCTCTTAGTCTCGACTCTAGGGATAATTTTTTTCGCTAT

CTTTTTTCGAGAACCGCCTAAAGTTCCAACTAAAAAGCTGAAATGATTTTGCATTATCTC

AATTGAAGTAATGAGCCTCCCCATATTGGGAGGCTCATTACTTCAACTAGTCCCCATGTT

CCTCGAATGGATCTCTTAGTTGTTGAGAAGGTTGCCCAAAAGCGGTATATAAGGCGTACC

CAGTAAAACTGACAAGTAAACCAGATATAAAGATGGCGACTAGGGTTGCTGTTTCCATTA

TGATTATATAATTTCAAGATCCCAATGGATCTATGATAAGATCGTTTATTTACAACGGAA

TGGTATACAAAGTCAACAGATCTCAATGAATACAATAGGATTTATGGCTACACAAACTGT

TGAGAACAGTTCTAGATCTGGTCCAAGACGAACTACTGTAGGGAGTTTATTAAAACCATT

GAATTCGGAATATGGTAAAGTAGCTCCCGGGTGGGGAACGACTCCTTTGATGGGTGTCGC

AATGGCTCTATTTGCGGTATTTCTATCTATTATTTTGGAGATTTATAATTCTTCCGTTTT

ATTGGATGGAATTTCAATGAATTAAATCTATAAGAACCGCAAAGTCCTGTCTTTTGAATA

AAAAAATGAATCAGTTAGAGCTCGGATTTCCAGCCTATTCTATTTTGGTAGTTCGATCGT

GGAATTTATTTCTTTCTGTATTTCCGGAATATGAGTGTGTGACTTGTTATAATTGATCCT

ATTGATAGTACAGAGAATGGGTCTGTTATCTTGATAGAGATGTTTCTACTTCGTCGGATA

TTTATTCTAGTATCTGGAACACGGAATATATGAACTAGATTAAGAAATATTTGAACTATG

ATTCATACTTAATATTCGACCTCGTGTCTGGACTCCAAAAAAAA-TTCAAAGAATTAGAA

T-AAAAAAAATTTTTTTTTAGTCTATCTATTGATGGAATAAGTGATGATCCAACGGTTCT

TACTCAGATAATCCTTGGCTTAACTTAGTTGAATCATCGTGGTTTTAGTATGAATTTGAG

GTTTGAATCGATTCATAGGGTCTTAACAAGATAATTCCTATCAATTCAATAATAAAGAAA

ACAAAAAAAGCCACATTAGATACATTAGATACAAAAACAAATTAAAGAAATAGTTAAAGA

GATAATTCAAGAGGCCCGTAAGGATCAACATAAAGACGATTGAGCCAACTTGATATTTTG

GTATTATCGCCACAAAGAAGAGCTTTCGGATTA-TTTTTT-TTATTCTTTCGTACATTCA

GATAAGATTGAATCAGAGATTAAGAAGTTTCAAACTTTCTATTACATATCCGTTGCAACT

AGTATTTGGGTGTTTTTGCTTGAGCTGTACGAGATGAAAGTCTCATATACGGTTCTGAGA

GGGGGATTTTCACCTATCTCAATAAAGTCTATGATTGGTTCGAAGAACGTCTCGAGATTC

AGGCGATTGCGGATGATATAACTAGTAAATACGTTCCTCCCCATGTGAATATATTTTATT

GTTTAGGGGGAATTACGCTTACTTGTTTTTTAGTACAAGTAGCTACGGGGTTTGCTATGA

CTTTTTACTATCGTCCGACCGTTACAGAGGCTTTTGCCTCTGTTCAATACATAATGACGG

AAGCTAACTTTGGTTGGTTAATCCGATCAGTTCATCGATGGTCCGCAAGTATGATGGTCC

TAATGATGATCCTGCATGTTTTTCGTGTGTATCTCACGGGTGGATTTAAAAAACCTCGCG

AATTGACTTGGGTTACAGGTGTGGTTCTGGGGGTATTGACCGCATCTTTTGGCGTAACTG

GTTATTCCTTACCTCGGGACCAAATTGGTTATTGGGCGGTGAAAATTGTAACAGGTGTAC

CTGAAGCTATTCCTGTAATAGGATCGCCTTTGGTAGAATTATTGCGTGGAAGTGCTAGTG

TGGGACAATCCACTTTGACTCGTTTTTATAGTTTACACACTTTTGTATTGCCACTTCTTA

CTGCTGTATTTATGTTAATGCACTTTCCAATGATACGTAAACAAGGTATTTCTGGTCCTT

TATAGAGAAGATATATAATAGATATTTGTAATCAATCATTTATCACTTGGAGGAGGAATA

ATAGTATTTCATTGCTACAAGTATGGATTATTGAAAATAATAATCCATGTATTTGGATAT

TTCCCTTCAACCAATCATGTCAAATAAATGTATAGTTGAGGGGAATTCTACGAAGAGAAA

ATGGATTATGGGAGTGTGTGACTTGAACTATTGATTGGTCTGTGTAGATATATGTCTGCC

ACATTGGAATTCACAACCAAATGTGTCTTTGTTCCAACCGTCGCGTAAGCCCATACAGAA

GATAGGCTGGTTCACTTGAAGAGATTCTTTTCTATGATCAGACCGAATCATGTTGTACAT

GAGCAGGCTCCGTAAGATCCAGTATAATTATAA-TAAGTGAAATGGATAAACTAAAACAG

AATCTTTATCTATTTCACTTACTTAATACTTAAAATTGAATAGTATGGAAATGCATTTAT

TTCCTCTGCATTGCTATGATCGATAATACTATCGGAGTGAAACAAGGGATTTAAAGAAAA

ACATAGGCTAGACTAGATTAGTAACAAGTAAACCTTTTGTGTG--TATCTCCAAATA-TT

TTGGAGATAAATACCAATCGTAAGGTCTGAGACGACCCAGAAAGCACTCGATCATATCAT

GATCAACTTTGTAAGCCAACTTGGGTATTGAGTATTTACTTAGAACCGAATTTTTTGCAA

TGGGTAGTTGCAATTCCGGAAAAAGAGTCAAATTTTTCTTACATTGAATCATTCATATAT

GTGTATATCATATATGTGTATATATAGGCAACATATAGATTTTTTATGGATTCATTTGGT

TCTTTTGAATCTTGCTCGAGCCGGATGATAAAAAATTATCATGTCCGGTTCCTTCGGGGG

ATGGATCTATAAGAATTCACCTATCCCAATAACAAAAAAACCTGATTTGAATGATCCTGT

ATTAAGAGCTAAATTGGCTAAAGGTATGGGTCATAATTATTACGGAGAACCCGCATGGCC

CAATGATCTTTTATATATTTTTCCAGTAGTAATTCTAGGTACTATTGCATGTAACGTAGG

CTTAGCGGTTCTAGAACCATCAATGATTGGTGAACCGGCAGATCCGTTTGCAACCCCTTT

GGAAATATTACCTGAATGGTATTTCTTTCCCGTATTTCAAATACTTCGTACAGTGCCCAA

TAAATTATTGGGTGTTCTTTTAATGGTTTCAGTACCTGCGGGATTATTAACAGTACCCTT

TTTAGAGAATGTTAATAAATTCCAAAATCCATTTCGTCGTCCAGTAGCGACAACCGTCTT

TTTGATTGGTACTGCAGTCGTTCTTTGGTTGGGTATTGGTGCAACATTACCTATTGATAA

ATCCCTAACTTTAGGTCTTTTTTAATTTGATTCAATTGTGAAATAACACGACGTGTGTAT

CTAGGGAATAGTCGCTTTCAAGCGAATTCTCCCTAGATACATCTATTCAATTCTGAATTT

CTTTCGAATATATGAATTGTGCTAAAGATTCAAAACCTATTTTCATCTTAATG-------

--AAAAAAAATCCAATAGATTTAAAACTTCTTTTTTGGTAAATCAATTGCGAAATGTTTT

TCTAGAATGACCAATATCTGTTTTATATCTTCTAGGCGCAAATGTTCAATTTTCATGAGA

TCTTCCGGACTGTTATTCAAAAGGTCCAATAATGTATATATATTGGACCTTTTGAGGCAA

TTATAGACCCTGGGAGAGAATTCTGATTGGTCAATAAAAATCGGTTTCAATGCTATTTTT

TTTTTGTTTTTTCTGAGTTTATCCAATTTATCGTGAAAGGTAAGAGGGGATAAAGGAACC

GTGTGTTGATTGTCCTCTAAAGGTAAGTTTTCTTCTTCCTTATGTAAAAAGGGAATAAAT

AAATCAATCAAATTCCGGCAGGCTTCATGAAGTGCTTCTTTCGGAGTTAAACTCCCATTT

GTCCATATTTCGAGAAAGAGTATCTCTTGTTTTTCATTCCCATTCCCATAAGAATGAATA

CTATGATTCGCATTTCGAACAGGCATGAATACAGCATCTATAGGATAACTTCCATCTTGA

AAGTTATGTGGCATTTTGATAAGATATCCGCGATTTCTCTTGATTTGTAATCCAATACAC

AAATCAATTGGTTCTGTCAAGCTAGCTATATGTTGTGTATTATCAACGATTTCTACATAA

GGTGGTAAGATGATATCTTGAGCAGTTACATATCCTGGACCTCTGACACAAATAGACGCG

TCACAAGTTCCATATAGATTACTTCTCAATACAATTTCTTTTAAATTCATGAAAATTTCA

TGGACCGATTCTTGAATACCCGCTATGGTAGAATATTCATGCGGGACGTTCTCAGATTTT

ACACGTGTGATACATGTTCCTTCTATTTCTCCAAGTAAAGCTCTTCGCATCGCAATGCCT

ATTGTGTCGGCTTGACCTTTCATAAGTGGAGACAGAATAAAGCGTCCATAATAAAGACGT

TTACTGTCTTCTCTTGATTCAACACACTTCCACTGTAGTGTCCGAGTAGATACTGTTACT

TTCTCTCGAACCATAGTAATATTATTTGATTTGATTGAATCATTTATTTCTCTTGTTTCT

CTTGAAATTTCTTCAATGTTAATTTCTACACACGTCTTTTTTTCGGGGGTCTGCAACCAT

TATGTGGCATAGGGGTTACATCCCGTACGAAAGTTAATAGTATACCACTTCTACGAATAG

CTCGTAATGCTGCGTCTCTTCCGAGACCGGGACCTTTTATCATGACTTCTGCTCGTTGCA

TACCTTGATCTACTACTGTACGAATAGCATTTGCTGCTGCAGTTTGAGCGGCAAAGGGTG

TCCCTCTTCTCGTACCCTTGAATCCACAAGTACCCGCTGAGGACCAAGAAACCACCCGAC

CCCGTACATCTGTAACCGTGACAATGGTATTATTGAAACTTGCTTGAACATGAATAACCC

CCTTTGGTATTCTACGTGCACTCTTACGTGAACCAATACGTCCATTTCTACGTGAACCAA

TTCTCGGTATAGCTTTTGCCATATTTTATCATCTCATAAATATGAGTCAGAGATATATGG

ATATATCCATTTCATGTCAAAACAGATTCCTTATTTGTACATCGAGTCCTTTAGTGAGTC

TGATTATCCTTGTCTTTGTTTATGTCTCGGGTTGGAACAAATTACTATAATGCGCCCCCG

CCTACGGATTAGGCGACATTTTTCACAAATTTTACGAACAGAAGCTCTTATTTTCATATT

TGTCATTCCTTATCTTAATTCTGAATCTACTTCTTGGAAGAAAATAAGTTTCTTGAAATT

TTTCATCTCGAATCATATTGAATAAAAACCACCTAATCCTTCCAATCCTTTTTGCGGAGT

CGATAAATTATACGTCCTCTGGTTGAATCATAACGACTTACTTCAATTTTGACTCTATCT

CCTGGCAGTATCCGTATAAAACTACGCCGGATCTTTCCTGAAACATAACCCAGGATCAGA

TCTTCATTATCTAACCGAACCCGGAACATACCATTGGGAAGCGATTCAGTAATTAAACCT

TCATGAATCCATTTTTGTTCTTTCATTCCAGGTAAAGCCTCCTTGAAGTATCAACTAATG

GAGGAGGAACGATATTAGACAACTCGTCCCTTTTCTTTTTTTTAGAAATAGGAAGAAGTT

TCGGATCCAATTTGGATATTAAAAGGATTACCATATATAACACAAAATTTCTCCGCCGAT

TCCTTCGAGTCGAGCCTCTCGGTCTGTCATTATACCTCGAGAAGTAGAAAGAATTACAAT

CCCCATCCCACCTAAAATTCTAGGAATTCGTTGAGAGTTAGAATAGATTCGTAGACCGGG

TCGACTGATCCGTTTTAAATTTAAAAAATTTCTATAGAGTCTTTTCCTATTCCTTCTATG

CCGCAGGTTTAAAACCAAAAAAGATTTGTTTTTTTCTCGATGTTTTCTAACGTTTTCAAT

AAAACCTTCTCGGAAAAGTATTTTAACAATATTTTCGGTAATATTAGTAGATGCGATGCG

AACCACTCTTTTTCTATCCATATCAGCATTTCGTATAGAGGTTATTATCTCAGCAATAGT

GTCCCTACCCATGGTGAACTAAAATTATGGGTGCCCCAAAATTGGATATAATCAACATGT

TTTTCTTTTTTTTTTTTACTT-ATTTGTTTTATGAATATGAATTATTAAAGGTATATGCG

TGAGACACAATCTACTAATTAATCTAGTTCTTAATCTATTTCTTTCAAATACCCACTATA

AACATATCAGTGATCTCATTTTATAATACCTCGGGAGCTAATGAAACTATTTTAGTAAAA

TGGAATTGTCTCAATTCCCGGGGGATCGCACCAAAAATTCTAGTTCCTTTTGGATTTCCT

TCTTGATCAATCACAACTGCAGCATTGTCATCATATCGTATTATCATACCGCTGTCACGT

TTAAGTTCTTTACAGGTACGAACAATTACAGCTCTGACTACTTCTGATTTTTCTAGGGGC

ATATTTGGTACTGCTTCTTTGATCACAGCAACAATAACGTCACCAATATGAGCATATCGA

CGATTGCTAGCTCCTATGATTCGAATACACATCAATTCTCGAGCCCCGCTGTTATCTGCT

ACATTTAAATGGGTCTGAGGTTGAATCATATCAATTTTTTAGTCTATTCTTCCAATGCAA

AGGATGAAG-AAAAAAAAGGAATATTTTTTGTCCAAAAAAAGAAACTTTCATCCCCAAGA

TTCTTCTGTTGGTTCTACATTTTTATCCTGAAATAATGAATTGAGTTCGTATAGGCATTT

TGGATGCTGCTATTGAAATAGCCCTTCTAGCTATATTTTCGGTTACTCCACCCATTTCGT

ATAGTATTCGACCTGGTTTAACAACAGCTACCCAATATTCAGGGGATCCCTTTCCCGAAC

CCATACGTGTTTCAGCGGGTCTTACTGTAACCGGTTTGTCTGGAAATATACGGACCCATA

TTTTTCCACCACGGCGTGCATTTCGTGTCATTGCTCGTCGACCTGCTTCGATTTGTCTAG

ATGTGATCCAAGCAGGTTCAAGTGCCTGAAGAGCATATTTACCGAAACAAATATGATTAC

CTCGATAAGATATTCCCTTCATTCTTCCTCTATGTTGTTTACGGAATCTAGTTCTTTTGG

GGTTATAGTTGATGGTTGTTTCACAATTCCATCTCTACTACAGAACCGGACGTGAGAGTT

TCTTCTCATCCAGCTCCTCACGAATAAAAGGATTAAAAACATTTAATTGAAATGATTAAC

ATTTTTTGTAAAAAATTTTTTTTTTAGAACGTTCTAAAAAATCTTTATTTTGTTTTGTCC

TTTATCCAAGTTTAGCAAAAAAAAAAAAAAGTTTTCGCGGGCGAATATTTACTCTTTCAA

TCTCTATTTCATTTGTAGGGCTCGTTCGTGACTTCTCCCAATAGATGAATTAATCTCCGG

TTCATTTCGCCATCCCGACTAGTGAATCATTAAGATTCATTTTTTCAATAAAATCTTTTG

CATGCACAGGTTCCATCGTTCCCATCGCTTCGTACTTAATGATTAGGTCCGAATTCTACA

ATGGAGCTCATAATCCAATTTGTTCCTGAGTCAATCTTCTTAGTCTTTATTGGCTCCAAG

CTCTTTATTTTTTTCTTGATTCATTTAATATTTATGAATCAATTAGTATTGATGCTTTAT

TACACTGTCTTTTATGAGATGACTCGTA-ACCTTACATATTGGAATTCTATATCATTGAT

ATTCTTTTTCTCTCTTTCTCTCATCCTTCCATTTATCCACACCTTTACTTCTTTCATTTT

GTTTTACAACTTCTAATTAAAGTTTATGC-AAAAAAAAATTTCAGTTGCTACAAAGATAT

GACTGATTTATCATATCTTGACTGGTTCTTTATATCCAGATAATACGAAGTGATGAGTTG

GTTATTAGTTCATACTATGGGGCTGGTCCTTTTTTAATCCTAACCCTAAAAAACCAACGA

GTCACACACTAAGCATAGCATTTATATCAAATGGTCAATTGAATTTTTATTCAACCCTAT

AGAATTAAGAATTAGAATTGCTCATTTTGATTCACCAGAAAAAGAATGAACGAGTTTCTA

TTTTTTTTTCTATCAATGGATAGAAGGGAAAGACAAGTAAAGGTTTCTTATTCTTCGTCT

ATAAATATCCAAATTTTGATACCCAAGACCCCATAGATAGTTCGAACTGTATAGGAACAA

TAATCAATTTTAGCTCGAATGGTTTGTAGGGGAACCCTACCTTCTCTGATCCATTCGACA

CGTGCAATTTCTTTTCCGTCGATACGTCCTGCAATTTGTACTTGAATTCCTTTTGTATCT

GCTTGTTCAGTTAATTCAATAGCTTTTTTCATTGCTTTTCGAAATGAAACTCTATTCTTT

AATTGTCCGGCTATAAATTCTGCAAGAATATTAGGGTTTCCATAAGGTTTTGCAATTCTT

GTGATAGCAACGTTAAGTTTTTGGTTCACATAATTAAATTCTTTTTCTAGATTAATCTGT

AATTCTTGGATTCCTCGCGTTCTATTTTCTATTAATAACTTTGGGAATCCCATAAAGATT

ATGACCTGGATCAAATCAATTCTTTTTTTAATTTCTATACGTGCAATTCCCTCGACGCCG

GAGGATATTCTCATATTCTTTTGTATATAATTTTTGATAAAATCTCTTATTTTTTGATCT

TCTTGTAAACCCTCAGAATAATTTTTGGGTTGTGCAAACCAAAGGGAATGATGACCTTGG

GTTGTACCAAGTCTGAAACCAAGTGGATTTATTTTTTGTCCCATACTCCCCCAATACTAT

ACATATCATGATATACCATAGTTGTATACTTATTTTTCCATCTAGGTTTTTTTAACGAAT

ATATCTGTCCATATTCATCATGTAAAGATATATCTTTCACTCCAATAGTTATATGACAGG

TAGGCCTTTTTATCGGATAACTACGTCCTCGAGCTCGAGGTTTGAATTTCTTCACGGTAG

TACCCTCGTTAACTTCGGCCTTACTAATGACTAAATTGGCTTCGTTGGAACCCATATTGT

AACTAGCATTTGCTGCTGCAGAGTAAACCAATTTAAAAATGGGATAACATGCTCTATAGG

GCATGAGTTCTAGTATCATAAGTGTTTCCTCATAGGAGCGTCCACGAATTTGATCAATTA

CTCTTCTTGCTTTGTCAGCCGATAGAGATATATGTCGACCTAAAGCGTATACTTCTGTTT

TTTTCTTCTTTCGCATAGGGTTTACCTCCTATTAATGAATCATAAGTATCTATCTATAAT

ATATATAAAAAAAAA--AGATTAACGCCGAGATCTATTATCGCTTTTTGCATGTCCTCGG

AAATTTAAAGTAGGTACAAATTCTCCTAATTTGTGTCCTACCATAC-GATCTGTTATATA

AGTAGGCAAATGTTCCTTACCATTATGAATAGCAATTGTATGGCCAATCATTGTGGGTAT

AATGGTAGATGCCCGGGACCAAGTTACTATTATTTCTTTTTCTGCTTTTTTATTAAGCTT

ATCAATTTTTCTTAATAAATGATTGACTACAAAAGGATTTTTTTTTAGTGAACGTGCCAC

AACTTCCTCCTATTTTTTTTTTGTTTTTGTAAAGACGAAGAAAGAAATTCTATTTTCTCG

CCTATTTACTACGGCGACGAAGAATCAAATTATCACTATATTTATTCCTTTTTCTACTTC

TGCTTCCAAGTGCAGGATAACCCCAAGGGGTTGTGGGTTTTTTTCTACCAATTGGGGCTC

TCCCTTCACCACCCCCATGGGGATGGTCTACAGGGTTCATAACTACTCCTCTTACTACAG

GACGCTTACCTAGCCAACGCTTAGATCCGGCTCTACCCAAACTTTTCTGGTTCGCCCCAA

CATTCCCCACTTGTCCGACTGTTGCTGAGCAGTTTTTGGATATCAAACGGACCTCCCCAG

AAGGTAATTTTAATGTGGCCGATTTCCCCTCTTTTGCAATCAGTTTCGCTACAGCACCCG

CTGCTCTAACTAATTGTCCACCCTTTCCAAGTGTGATTTCTATGTTATGTATGGCCGTGC

CTAAGGGCATATCGGTTGAAGTAGATTCTTCTTTTTGATCAATCAAAACCCCTTCCCAAA

CTGTACAAGCTTCTTCCAAAGCATACGGCTTTCTGGATGTAGATGATGATATCTATACAG

ATGGATCTTTTATATATCGTACAATGAAGTACCACA----TGGATATCTATATGAATCCA

AATCTGCCGAATCACTCATGGTATGATCTTCTACATCCTAGGTCTTCCCGTTCCGTCATC

TGGCTTATGTTCTTCATGTAGCATTCAGACCGAATGACTCTATGAAATTACGTCGATACT

TCCACATATTATGGGTAACGTAGGAGACATCTCTATTTTTCCCCCGGGGAATCTTTAGAA

TTCCCACTGCTTAGCTTTCAATTCGCCTCTGACCATCAAATGAAATGTGAATAACCCGTC

CTCCTCTCTTTGAAAGTTTGAAAGAAGGGGCGCTTCCGGTTCTGTCGGTGCTTGAAACAA

TTTTGTCTTCTCCATATTACTATATCTCTATAGTCAATAATTTTATATGAGGAACTACTG

AACTCAATCACTTGCTGCCGTTACTCTTCAGTTTTCTGTTGAGGTCTATCCTGTAGAGGT

ACTCAAATTGGATCAGTGATCGATTTCTAGGTTTCGTCGTAAACCTAATTGGTTACTTCC

AATTACGTAAATCAATAGTTCAAACCGCACTCAAAGGTAGGGCATTTCCCATTTTTATAG

GAACTTCTGTACCAGAAACAATGGTATCTCCAATTATAGCCCCTCTGGGATGTAAAATAT

ATCTCTTCTCACCATCCCCATAGTGTATGAGACAAATGTATGCATTTCGATTAGGGTCGT

ATTCTATGGTTACGATTCTACCATATATGTCTTTTTCATTCCGTCGAAAATCGATTTTAC

GGTATAGACGCTTATGACCTCCCCCTCTATGCCTTGCGGTAATGATTCCTCTGGCATTAC

GACCTTTACCACAATGATGCTGTCCATAGATCAAATTATTTCGTGGATTGGATTTCACTT

GACTGTCTACGGTTCCATTGCGTGTGCTCGGGGTAGAAGTTTTGTATAAATGTATCGCCA

TGCTATTAAGTATTTTGATTTAAGTTCTTTTCTTTCTAAGAGGTGGAATAGAATAACCCG

GTTGAAGCGTAATGATCATACGTCTGTAATGCATTGTATGTCCCATAATAGGTCCCATTC

TTCTACCCTTTCCCGGAAGTCGATGACTATTCATAGCTATTACCTTGACACCAAAGAAGA

GTTCGACCCAATGCTTTATTTCTGTCCTAGTTGATCCTGATTCGACATTAGAAGTATATT

GATTTTTCCCCAATAACCGAATACTTTTGTCTGTAAATACTGCATATTTGATTCCATCCA

TAAATCGATTTTCTTCCCTATGAGTTCTAGTCTCAATAAGAATGCTAGTTCTTACTGTTC

ATATATTATGATATGAATATACCACACCAATTCGTTATGTATGGATGATGAGATTCCATT

GATACAGAGCCAATTCCAATAGACTTATTGGAGGGTCCCATTGGCGTGCATCCAGTAGGA

ATTGAACCTACGAATTCGCCAATTATGAGTTGGGCGCTTTAACCATTCAGCCATGGATGC

TTAGCGGGGATCCTCGTACATGGTGAATAACCAAATTCCAATTGAAATGAAATCTTTAGG

ATAAATCAATGCAATTTAGGAGGAATCAATGAAAGGACATCAATTCAAATCCTGGATTTT

CGAATTGAGAGAGATCAAGAATTCTCACTATTTCTTAGATTCATGGACCCAATTCAATTC

AGTGGGATCTTTCATTCACATTTTTTTCCACCAAGAACGTTTTCTAAAACTCTTTGACCC

CCGAATTTTGAGTATCCTACTTTCACGCAATTCACAGGGGTCAACAAGCAATCGATATTT

CACGATCAAGGGTGTAATACTCTTTGTAGTAGCGGTCCTTATATATCGTATTAACAATCG

AAATATGGTCGAAAGAAAAAATCTCTATTTGAGAGGGCTTCTTCCTATACCTATGAATTC

CATTGGACCCAGAAATGATACATTGGAAGAATCCGTTGGGTCTTCCAATATCAATAGGTT

GATTGTTTCGCTCCTGTATCTTCCAAAAGGAAAAAAGATCTCTGAGAGTTGTTTCCTGAA

TCCGAAAGAGAGTACTTGGGTTCTCCCAATAACTAAAAAGTGTAGCATGCCTGAATCTAA

CTGGGGTTCGCGGTGGTGGAGGAACTGGATCGGAAAAAAGAGGGATTCTAGTTGTAAGAT

ATCTAATGAAACCGTCGCTGGAATTGAGATCTTATTCAAAGAGAAAGATATCAAATATCT

GGAGTTTCTTTTTGTATATTATATGGATGATCCGATCCGCAAGGACCATGATTGGGAATT

GTTTGATCGTCTTTCTCTGAGGAAGAGGCGAAATAGAATCAACTTGAATTCGGGACCGCT

ATTCGAAATCTTAGTGAAACACTGGATTTCTTATCTCATGTCTGCTTTTCGTGAAAAAAT

ACCAATTGAAGTGGAGGGTTTCTTCAAACAACAAGGGGCTGGGTCAACTATTCAATCAAA

TGATATTGAGCATGTTTCCCATCTCTTCTCGAGAAACAAGTGGGCTATTTCTTTGCAAAA

TTGTGCTCAATTTCATATGTGGCAATTCCGCCAAGATCTCTTCCTTAGTTGGGGGAAGAA

TCCGCACGAATCGGATTTTTTGAGGAACGTATCGAGAGAGAATTTGATTTGGTTAGACAA

TGTGTGGTTGGTAAACAAGGATCGGTTTTTTAGAAAGGTACGGAATGTATCGTCAAATAT

TCAATATGATTCCACAAGATCTAGTTTCGTTCAAGTAACGGATTCTAGCCAACTGAAAGG

ATCTTCTGATCAATCCAGAGATCATTTGGATTCCATTAGTAATGAGGATTCGGAATATCA

CACATTGATCAATCAAAGAGAGATTCAACAACTAAAAGAAAGATCGATTCTTTGGGATCC

TTCCTTTCTTCAAACGGAAGGAACAGAGATAGAATCAGACCGATTCCCGAAATGCCTTTC

TGGATATTCCTCAATGTCCCGGCTATTCACGGAACGTGAGAAGCAGATGATTAATCATCT

GCTTCCGGAAGAAATCGAAGAATTTCTTGGGAATCCTACAAGATCCGTTCGTTCTTTTTT

CTCTGATAGATGGTCAGAACTTCATCTGGGTTCGAATCCTACTGAGAGGTCCACTAGAGA

TCAGAAATTGTTGAAGAAACAACAAGATCTTTCTTTTGTCCCTTCCAGGCGATCGGAAAA

TAAAGAAATGGTTAATATATTCAAGATAATTACGTATTTACAAAATACTGTCTCAATTCA

TCCTATTTCATCAGATCCGGGGTGTGATAGGGTTCCGAAGGATGAACCGGATATGGACAG

TTCCAATAAGATTTCATTCTTGAACAAAAATCCATTTTTTGATTTATTTCATCTATTCCA

TGACCGGAACAGGCGAGGATACACGTTACACCACGATTTTGAATCAGAAGAGAGATTTCA

AGAAATGGCAGATCTATTCACTCTATCAATAACCGAGCCGGATCTGGTGTATCATAAGGG

ATTTGCCTTTTCTATTGATTCCTACGGATTGGATCAAAAACAATTCTTGAATGAGGCCAG

GGATGAATCGAAAAAGAAATCTTTATTGGTTCTACCTCCTATTTTTTATGAAGAGAATGA

ATCTTTTTCTCGAAGGATCAGAAAAAAATGGGTCCGGATCTCCTGCGGGAATGATTTGGA

AGATCCAAAACCAAAAATAGTGGTATTTGCTAGCAACAACATAATGGAGGCAGTCAATCA

ATATAGATTGATCCGAAATCTGATTCAAATCCAATATAGTACCTATGGGTACATAAGAAA

TGTATTGCATCGATTCTTTTTAATGAATCGATCCGATCGCAACTTCGAATATGGAATTCA

AAGGGATCAAATAGGAAAGGATACTCTGAATCATAGAACTATAATGAAATATACGATCAA

CCAACATTTATCGAATTTGAAAAAGAGTCAGTTCGATCCTCTTATCTTGATTTCTCGAAC

CGAGAAATCCATGAATCGGGATCCTGATGCATATAGATACAAATGGTCCAATGGGAGCAA

GAATTTCCAGGAACATTTGGAACATTTCGTTTCTGAGCAGAAGAGCCGTTTGCAAGTAGT

GTTCGATCAATTACGTATTAATCAATATTCGATTGATTGGTCTGAGGTTATCGACAAAAA

AGATTTGTCTAAGCCACTTCGTTTCTTTTTGTCCAAGTCACTTCTTTTTTTGTCCAAGTT

GCTTTTCTTTTTGTCTAACTCACTTCCTTTTTTCTGTGTGAGTTTCGGGAATATCCCCAT

TCATAGGTCCGAGATCTACATCTATGAATTGAAAGGTCCGAATGATCAACTCTGCAATCA

GTTGTTAGAATCAATAGGTCTTCAAATTGTTCATTTGAAAAAATGGAAACCCTTCTTATT

GGATGATCATGATACTTCCCGAAAATCGAAATTCTTGATCAATGGAGGAACAATATCACC

CTTTTTGTTCAATAAGATCCCAAAGTGGATGATTGACTCATTCCATACTAGAAATAATCG

CAGGAAATCCTTTGATAACACGGATTCCTATTTCTCAATGATATCCCACAATCAAGACAA

TTGGCTGAATCCCGTGAAACCATTTCATAGAAGTTCATTGATATCTTCTTTTTATAAAGC

AAATCGACTTCGATTCTTGAATAATCCACATCACTTCTGCTTCTATTGTAACACAAGATT

CCCCTTTTCTGTGGAAAAGGCCCGTATCTATAATTATGATTTTACGTATGGACAATTCCT

CAATATCTTGTTCATTCGCAACAAAATATTTTCTTTGTGCGTCGGTAAAAAAAAACATGC

TTTTGGGGGGAGAGATACTATTTCACCAATCGAGTCACAGGTATCTAACATATTCATACC

TAACTATTTTCCACAAAGTGGTGACGAAACGTATAACTTGTACAAATCTTTCCATTTTCC

AAGTCGATACGATCCATTCGTTCGTAGAACTATTTACTCGATCGCAGACATTTCTGGAAC

ACCTCTAACAGAGGGACAAATAGTCAATTTTGAAAGAACTTATTGTCAACCTCTTTCAGA

TATGAATCTATCTGATTCAGAAGGGAAGAACTTGCATCAGTATCTCAATTTCAATTCAAA

CATGGGTTTGATTCACACTCCATGTTCTGAGAAATATTTACCATCCGAAAAGAGGAAAAA

ACGGAGTCTTTGTCTAAAGAAATGCGTTGAGAAAGGGCAGATGTATAGAAGAGATAGTGC

TTTTTCAACTCTCTCAAAATGGAATCTATTCCAAACATATATGCCATGGTTCCTTACTTC

GACAGGGTACAAATATCTAAATTTGATATTTTTAGATACTTTTTCAGACCTATTGCCGAT

ACTAAGTAGCAGTCAAAAATTTGTATCCATTTTTCATGATATTATGCATGGATCAGGTAT

ATCATGGCGAATTCTTCAGAAAAAATTGTGTCTTCCACAATGGAATCTGATAAGTGAGAT

TTCGAGTAAGTGTTTACATAGTCTTCTTCTGTCCGAAGAAATGATTCATCGAAATAATGA

GTCACCATTGATATCGACACATCTGAGATCGCCAAATGTTCGGGAGTTCCTCTATTCAAT

CCTTTTCCTTCTTCTTGTTGCTGGATATCTCGTTCGTACACATCTTCTCTTTGTTTCCCG

GGCCTCTAGTGAGTTACAGACAGAGTTCAAAAAGGTCAAATCTTTGATGATTCCATCATC

TATGATTGAGTTGCGAAAACTTCTGGATAGGTATCCTACATCTGAACCGAATTCTTTCTG

GTTAAAGAATCTCTTTCTAGTTGCTCTGGAACAATTAGGAGATTCTCTAGAAGAAATACG

GGGTTCTGCTTCTGGCGGCAACATGCTTGGTCCCGCTTATGGGGTCAAATCAATACGTTC

TAAGAAGAAATATTTGAATATCAATCTCATCGATCTCATACCAAATCCCATCAATCGAAT

CACTGTTTCGAGAAATACGAGACATCTAAGTCATACAAGTAAAGAGATCTATTCATTGAT

AAGAAAAAGAAAAAACGTGAACGGGGATTGGATTGATGATAAAATAGAATCCTGGGTCGC

GAACAGTGATTCGATTGATGATGAAGAAAGAGAATTCTTGGTTCAGTTCTCCGCCTTAAC

GACAGAAAAAAGGATTGATCAAATTCTATTGAGTCTGACTCATAGTGATCATTTATCAAA

GAATGACTCTGGTTATCAAATGATTGAACAACCGGGAGCAATTTACTTACGATACTTAGT

TGACATTCATAAAAAGTATCTATTGAATTATGAGTTCAATACATCCTGTTTAGCAGAAAG

ACGGGTATTCCTTGCTCATTATCAGACAATCACTTATTCACAAACTTCGTGTGGGACTAA

TACTTTTCATTTCCCATCTCATGGAAAACCCTTTTCGCTCCGCTTAGCCTTATCCCCCTC

TAGGGGGATTTTAGTGATAGGTTCTATAGGAACTGGACGATCCTATTTGGTCAAATACCT

AGCGACAAACTCCTATGTTCCTTTCATTACGGTATTTCTGAACAAGTTCCTGGATAACAA

GCCTAAAGGTTTTCTTATTGATGATATCAATATTGATGATAGTGACGATATTGATGATAG

TGACAATATTGATGCTAGTGACGATATTGATGCTAGTGACGATATCGATCGTGACCTTGA

TACGGAGCTGGAACTGCTAACTATGATGAATGCGCTAACTATGGATATGATGCCGGAAAT

AGACCGATTTTATATCACCCTTCAATTCGAATTAGCAAAAGCAATGTCTCCTTGCATAAT

ATGGATTCCAAACATTCATGATCTGGATGTGAATGAGTCGAATTACTTATCCCTCGGTCT

ATTAGTGAACCATCTCTCTGAAAGATGTTCCACTAGAAATATTCTTGTTATTGCTTCGAC

TCATATTCCCAAAAAAGTGGATCCCGCTCTAATAGCTCCGAATAAATTAAATACGTGCAT

TAAGATACGAAGGCTTCTTATTCCACAACAACAAAAGCACTTTTTCACTCTTTCATATAC

TAGGGGATTTCACTTGGAAAAGAAAATGTTCCATACTAATGGATTCGGGTCCATAACCAT

GGGTTCCAATGCACGAGATCTTGTAGCACTTACCAATGAGGCCCTATCGATTAGTATTAC

ACAGAAGAAATCAATTATAGACACTAATACAATTAGATCCGCTCTTCATAGACAAACTTG

GGATTTGCGATCCCAGGTAAGATCGGTTCAGGATCATGGGATCCTTTTCTATCAGATAGG

AAGGGCTGTAGCACAAAATGTACTTCTAAGTAATTGCCCCATAGATCCTATATCTATCTA

TATGAAGAAGAGATCATGTAACGAAGGGGATTCTTATTTGTCCAAATGGTACTTCGAACT

TGGAACGAGCATGAAGAAATTAACGATACTTCTTTATCTTTTGAGTTGTTCTGTCGGATC

GGTCGCTCAAGATCTTTGGTCTCTACCCGGACCCGATGAAAAAAATGGGATCACTTCTTA

TGGACTCGTTGAGAATGATTCTGATCTAGTTCATGGCCTATTAGAAGTAGAAGGCGCTTT

GGTGGGATCTTCACGGACAGAAAAAGATTGCAGTCAGTTTGATAATGATCGAGTGACATT

GCTTCTTCGGCCCGAACCGAGGAATCCCTTAGATATGATGCAAAACGGATTTTGTTCTAT

CTTTGATCAGAGATTTCTCTATGAAAAATACGAATCGGAGTTTGAAGAAGGGGAGGGAGA

AGGAGCCCTTGACCCGCAACAGATAGAGGAGGATTTATTCAATCACATAGTTTGGGCTCC

TAGAATATGGCGCCCTTGGGGCTTTCTATTTGATTGTATCGAAAGGCCCAATGAATTGGG

ATTTCCCTATTGGTCCAGGTCATTTCGGGGCAAGCGGATCATTTATGATGAAGAGGATGA

GCTTCAAGAGAATGATTCGGAGTTCTTGCAGAGTGGAACCATGCAGTACCAGACACGAGA

TAGATCTTCCAAAGAACAAGGCCTTTTTCGAATAAGCCAATTCATTTGGGACCCTGCAGA

TCCGCTCTTTTTCCTATTCAAAGATCAGCCCCCTGGCTCTGTGTTTTCACATCGAGAATT

ATTTGCAGATGAAGAGATGTCAAAGGGGCTTCTTACTTCCCAAACAGATCCTCCTACATC

TATATATAAACGCTGGTTTATCAAGAATACACAAGAAAAGCACTTCGAATTGTTGATTAA

TCGTCAGAGATGGCTTAGAACCAATAGTTCATTATCTAATGGATCTTTCCGTTCTAATAC

TCTATCCGAGAGTTATCAGTATTTATCAAATCTGTTCCTATCTAACGGAACGCTATTGGA

TCAAATGACAAAGACATTGTTGAGAAAAAGATGGCTTTTCCCGGATGAAATGAAAATTGG

ATTCATGTAACAGGAGAAAGATTTCCCATTCCTTAGCCGGAAAGATATGTGGCCATGAAA

GAGAAAGAGGGATTAAGTAGAACAGAATTGACTGGGTGGTAGAGTCGTGGAAACGCTTCT

TTCTTCCATATTTTGGACCTTAGCTCCATGGAACAATATGTTACTGCTGAAACACGGAAG

AATTGAAATCTTAGATCAAAACACTATGTATGGATGGTATGAACTGCCCAAACAAGAATT

CTTGAACAGCGAACAACCAGTTCAGATATTCACGACCAAGAAGTACTGGATTCTCTTTCG

GATAGGCCCTGAAAGGAGAAGGAAGGCTGGAATGCCAACAGGCGTCTATTATTGAATTCA

CCCGACCCGATAGTACCCATTTTGGGAACGTCCAGTGCCAAAGTCACTGAATGGGTAAGT

CGCCAATCCCTGGACTATGTAATCTGCTGGGTTACGGGCGGGCATTTTACCAGAGGTTTC

TAATCTACCCTTGTGTGATTCCTGTTGAAGCATATACTCGGGGGTGGGTGCAGGGCGGAC

GATTTTAAAGCAGACTCCCCATTCATTAGATAGAGAAGATCACCAAGATTTCGTGATTCG

CTGCCGAACTTATTCCAATTCCAAGAGCTCGGATCGAATCGGTATATACCGATTCGATCC

GAGCTCTCTTATTGAATTGCTCATTCAATGAGCATTCTCAATATTATGCCTTGAAGAGGA

CTCGAACCTCCACGCTCTTTAGCACGAGATTTTGAGTCTCGCGTGTCTACCATTTCACCA

CCAAGGCATCTTGAAAGTGAATCCTATTCCATGAATATGATATCTATCTAGTGTGATGTA

TGGAATATATGACAAGGGTGGAGTATTTCTATTGATCGGCCCGAGTTGGACATCCAATTG

CTTCGATTTGAATTATCCGGAGAATGCCTTATGTATATATCAAAAAGATGGCCAATCAAA

CCTATTTCTCGATTCAATAGAAGCTCAAAGAGGTGATATAGGGTCCCAAATAACGAGAGA

TATGTAAAAAGCAGGTCCGATTATTACGCCTATTCCTAATCCTAAATGGAATGGAACGAC

GTAGGGATCCATATGTAAACATAGTATCTATTTAGATACGCTCGAATGACCCCTTGAGAA

TGTATATAACCCTATTCCGGTCTGGTCCGGTATGGAATGAACTTATAATCATGGAATCGA

CTCGATCATCAGATTATAAGTTCATAACCCTAGCCCATTCCCATTTTGGTCGGAACAGAT

CTACTAATTCTTTGATTCCAGTTAGTAAGAGGGATCTTGAACTAAGAAATAGACCCTAGA

AGCTAAAAAAGGGTATCCTGAGCAATTGCAATAATCGGGTTCATTGATATTCCAGGTATA

GTAGATGCTATCACACATACAATCATACTCAATTCGATGGAATTGTTTGATCTTAAAGGA

GATCTTCTATAATTTCGCACGTGAGGGGTGATTTCTTGGTTTCGTCCAGTCATTAATAAC

TTGATTATTTTTAGATAATAGTAGATAGAAACAACGCTTGTAAGGAGTCCTATTAAAACC

AAGGAATATAGGCCTGCCTGCCATCCACACCAGAATAAATAGAGTTTTCCGAAAAAACCT

GCTAGTGGAGGAAGACCTCCTAGGGATAAGAGACATAGGGCTAAAGAGAGAGCCAAAAAA

GGATCTTTCGTGTATAATCCTGCATAATCTCGAATGTTATCAGTTCCGGTACGTAGACCA

AATAATACAATGCAAGCAAAAGTTCCTAGATTCATGGAGATATAGAACAGCATATAAGTT

ATCATGCTTGCATATCCATCATTTGAGTCTCCAACAATTATTCCAATAATTACATATCCG

ATTTGACCTATGGACGAATATGCAAGCATACGTTTCATGCTTGTTTGAGTAATAGCAATG

ATATTTCCCAATATCATGCTAAGAATAGCTAGGATTTCCAGAAGAAGATGCCATTCGTTT

GATGAGAAATAAAAAGGAATATCGAAAATTCGAGTGGCTGAAGCTGAAGCAGCTACTTTC

GAAGTAACAGAAAGAAAAGCAACGACTGGAGTGGGAGAGTCAGAGTCGAAAAGAGGATTC

CTCACTTCTTTCTCTCATTCAAAACCGTGCATGAGACTTTCATCTCACACGGCTCCTAAG

TGATAAAAGAAAGAAGAACTCATCTTCTTTCTTTTTTGATTACCTTCCTCGCGTATGTAT

AAGACCGAATCCATTCGATTTCTAAAAAAGATTACTAATCCTTAACTTTTCGAGGAATCC

TTCATCAGTGGTTGTGAATGACTGATTTTTTCAATCTTTTCGACCTTGGTTCCGTAGGAG

CAAGTCAGAAAGATTGAGAAATAGAACCATCTGATTTAATTCGTTCTCAATAGCCATGAA

ATGATCATCTTAGGGTGATCCTTTTGTCGACGGATGCTCCTATTACACTCGTAGTCTCTG

AAGGATGAGAACCAACTATGTAGCATCTACATCAAGAATTCAAGTATTGTATACGTCATT

AGTCCGATCCTTTGTAGGAACTACCCGTAATAACGAACTTGCAAAATGGATCTGTTTATC

ATAAAGAGATTCGTCGTTCCTGACCCTGCTTCACCTTAATTGTTATTTGAACAAGTAAAA

GTTCTGTCTTGGTCCGAGTGGGGATAGCATTTCTCTTCTGCATGTCCATGGAGTTTTGAA

AAATCCAAACATCTCAGAGATAGATAGAGAGGTAGGAATTTCTCGAACGAACCGCACTCC

TTCGTATACGTCAGGAGTCCATTGATGAGAAGGGGCTGGGGAAAGCTTGAACCCAATTCC

TACAGTGATGAATATGAGCGCAATTGAAATTCCTGGGGAGTTATACATTTGTGTATTGAT

AAGACCATTCACTATTTCTTGAAGCTCGATCTCTCCCCCGGATGAACCATATAGCCAAGA

GAAACCATGAACCAGAATAGAAGAGCTTGCCCCACCCATGAGTAAATATTTCATAGTAGC

CTCATTAGACCGTACATCTTTCTTGGTATATCCAGATAATAGGTAGGAGCATAAACTGAA

ACATTCTGGAGCTACAAAGATAGTTATTAAATCGTTAGCACCGCATAAAAACATTCCTCC

TAGAGTAGCTGTTAATACGAATAAGAGAAACTCTGTTATAGCCATTTCTGTACATTCAAT

GTACTCTACGGATAGAGGAATACATAGAGTTGAACATAGTAAAATAAGAAATTGAAAGAT

TTCGTTGAAATTGTTCGTTTGGAAATTTCCCGAAAAGCTAATCATAGGTTCTTCTCTCCA

TCGGAACAATAGGGCCGTTATGCTCATTACTAAACTTGTTGAAGAGATGAAATATAACCA

AGGTATATCTTTTTGATCAGAGGTTGAATCGATCATCAGAAGAAGAATTAGGCCAAAAAT

TAGGATACATTCTGGGAAAATCAAACTTCCATCGAAGAGAAGCAAATGAAAGGCTTTCAT

AAAAATTCTCGTAGAATCGAGAATGAAGTTTTCATTCTGTACATGCCAGATCATGAATTA

GTAACTGCATCCAATTTCAAAAAAAAATCCCAATTGTGTCGAACTTTCCATTTTTGGAAT

GGAATAGGATCAAGATCAAACCTTATTCCATGGTATTTACATGAGGTTCCTCTTTAAGAA

AGTCCCCGAGAGGGCTTAGTTGATCCATGATTTATGTTTCATCTTTCGTTTCCTTTTCGT

TTGTTTCGAGAAATCTATCGATCAATTCCGATTCTTTCTTTTTCTCTTGATTCTTTTCCG

ATCGAGATGTATAGATCCTGTTCATGGATTAACGAAAATGTGCAAAAGCTCTATTTGCCT

CTGCCATTTTATGAGTCTCTTCCTTTTTGCGTATGGCATCGCCACTCCCTTTGGCAGCAT

CCACTAATTCGGAACTTAATTTGAAAGCCATATTTCGACCCGGACGTTTTCGGGATGCCG

CTAATAACCAACGAATGGCAAGTGCTTTTCCTTGTGTGGATCCTATTTCAATGGGAACTT

GATGAGTTGATCCACCTACACGTCTTGCTTTTACTGCTATATCGGGAGTTACTCCACGTA

TTGCTTGACGTAAAACAGATAGTGGATTTGTTTCTGTCTTTTGTTGAATCTTTTTCATGG

CTCGATAGATAATTTGATAAGCCAATGATTTTTTTCCGTGTTTCAGAATACGGTTAACCA

ACATGTTAACTAATCGATTACGATAAATTGGATCGGATTTTGCTGTTTTTTCTTCTGCAG

TACCTCGACGTGACATGAGCGTGAAAGGGGTTCAAGAATCCGTTTTCTTTTTATAAGGGC

TAAAATCACTTA-TTTTTTTTTTGCTTTTTTACCCCATATTGTAGGGTGGATCTCGAAAG

ATATGAAAGATCTCCCTCCAAGCCGTACATACGACTTTCATCGAATACGGCTTTCCGCAG

AATTCTATATGTATCTTTGAGATCGAGTATGGAATTCTGTTTACTCACTTTAAATTGAGT

ATCCGTTTCCCTCCCTTTCCTGCTAGGATTGGAAATCCTGTATTTTACATATCCATACGA

TTGAGTCCTTGGGTTTCCGAAATAGTGTAAAAAGAAGTGCTTCGAATCATTGCTATTTGA

CTCGGACCTGTTCTAAAAAAGTCGAGGTATTTCGAATTGTTTGTTGACACGGACAAAGTC

AGGGAAAACCTCTGAAATTATTTCCATATTGAACCTTGGACATATAAGAGTTCCGAATCG

AATCTCTTTAGAAAGAAGATCTTTTGTCTCATGGTAGCCTGCTCCAGTCCCCTTACGAAA

CTTTCGTTATTGGGTTAGCCATACACTTCACATGTTTCTAGCGATTCACATGGCATCATC

AAATGATACAAGTCTTGGATAAGAATCTACAACGCACTAGAACGCCCTTGTTGACGATCC

TTTACTCCGACAGCATCTAGGGTTCCTCGAACAATGTGATATCTTACACCGGGTAAATCC

TTAACCCTTCCCCCTCTTACTAAGACTGAAGAATGTTCTTGTGAATTATGGCCAATACCG

GGTATATAAGCAGTGATTTCAAATCCAGAGGTTAATCGTACTCTGGCAACTTTACGTAAG

GCAGAGTTTGGTTTTTTTGGGGTGATAGTGGAAAAGTTGACAGATAAGTCACCCTTACTG

CCACTCTACAGAACCGTACATGAGATTTTCACCTCATACGGCTCCTCGTTCAATTCTTTC

GAATTCATTGGATCCTTTTCCGCGTTCGAGAATCCCCTCCCTTCTTCCACTCTGTCCCGA

AGAGTAACTAGGACCATTTAGTCACGTTTTCATGTTCCAATTGAACACTTTCCATTTTTG

ATTATTTTCAAAGGAGAAGATTATTCTCTTTACCAAACATATGCGGATCCAATCACGATC

CTATAATAAGAACAAGAGATCTTTCTCGATCAATCCCTTTGCCCCTCATTCTTCGAGAAT

CAGAAAGATCCTTTTCAAGTTTGAATTTGTTCATTTGGAATCTGGGTTCTTCTACTTCAT

TTTTATTTAATATTTTTCCCTCTCTTTTTTTTTATATCATTCCTTAAGTCCCATAGGTTT

GATCCTGTAGAATTTGACCCATTTTCTCATTGAACGAAGGGTACGAAATCAATCAGATTG

ATTTTTCGATCAAAAGTACTATGTGAAATCTTCGGTTTTTTCCTCTTCCTCTATCCCTAT

CCCATAGGTACAGTGTTTGAATCAATAGAGAACCTTTTCTTCTGTATGAATCGATCTTAT

TCCATTCCAATTCCTTCCCGATACCTCCCAAGGAAAATATCGAATGGATCCCAAATTGAC

GGGTTAGTGTGAGCTTATCCATGCGGTTATGCACTCTTCGAATAGGAATCCGTTTTCTGA

AAGATCCTGGCTTTCGTACTTTGGTGGGTCTCCGAGATCCTTTCGATGACCTATGTTGTG

TTGAAGGGATATCTATCTAATCCGATCGATTGCGTAAAGCCCGCGATAGCAACGGAACCG

GGGAAAGTATACAGAAAAGATAGTTCTTTTCTATTATATTAGTATTTTCTATTATATTAG

ATTAGTATTAGTTAGTGATCCCGGCTTAGTGAGTCCTTTCTTCCGTGATGAACTGTTGGC

ACCAGTCCTACATTTTGTCTCTGTGGACCGAGGAGAAAAGGGGCTCGGCGTGTACATGAG

AGAAGCAAGGAGGTCAACCTCTTTCAAATATACAACATGGATTCTGGCAATGCAATGTAG

TTGGACTCTCATGTCGATCCGAATGAATCATCCTTTCCGCGGAGGTCAATCTTTGCCTGC

TAGGCAAGAGGAGAGCAAGTTACAAATTCCGTCTCGGTAGGACATGTATTTCTATTACTA

TGAAATTCATAAATGAAGTAGTTCATGGTGGGGTTACCATTATCCTTCTTGTAGTGACGA

ATCTTGTATGTGTTCTTAAGAAAAGGAATTTGTCCATTTTTCGGGGTCTCAAAGGGCGTG

GAAACACATAAGAACTCTTGAATGGAAATGGAAAAGAGATGTAACTCCAGTTCCTTCGGA

ATCGCTAGTCAATCCTATTTCCGATAGGGGCAGTTGACAATTTAATCCGATTTTGACCAT

TATTTTCATATCCGTAATAGTGCGAAAAGAAGGCCCGGCTCCAAGTTGTTCAAGAATAGT

GGCGTTGAGTTTCTCGACCCTTTGACTTAGGATTAGTCAGTTCTATTTCTCGATGGGGGC

AGGGAAGGGATATAACTCAGCGGTAGAGTGTCACCTTGACGTGGTGGAAGTCATCAGTTC

GAGCCTGATTATCCCTAAACCCAATGTGAGTTCTTCTATTTGGATTTGCTCCCCCGCCGT

GATTCAATGAGAATGGATAAGAGGCTCGTGGGATTGACGTGAGGGGGTAGGGATGGCTAT

ATTTCTGGGAGCGAACTCCGGGCGAATATGAAGCGCATGGATACAAGTTATGCCTTGGAG

TGAAAGACAATTCCGAATCCGCTTTGTCTACGAACAAGGAAGCTATAAGTAATGCAACTA

TGAATCTCATGGAGAGTTCGATCCTGGCTCAGGATGAACGCTGGCGGCATGCTTAACACA

TGCAAGTCGGACGGGAAGTGGTGTTTCCAGTGGCGGACGGGTGAGTAACGCGTAAGAACC

TGCCCTTGGGAGGGGAACAACAGCTGGAAACGGCTGCTAATACCCCGTAGGCTGAGGAGC

AAAAGGAGGAATCCGCCCGAGGAGGGGCTTGCGTCTGATTAGCTAGTTGGTGAGGCAATA

GCTTACCAAGGCAATGATCAGTAGCTGGTCCGAGAGGATGATCAGCCACACTGGGACTGA

GACACGGCCCAGACTCCTACGGGAGGCAGCAGTGGGGAATTTTCCGCAATGGGCGAAAGC

CTGACGGAGCAATGCCGCGTGGAGGTAGAAGGCCCACGGGTCGTGAACTTCTTTTCCCGG

AGAAGAAGCAATGACGGTATCT-GGGGAATAAGCATCGGCTAACTCTGTGCCAGCAGCCG

CGGTAAGACAGAGGATGCAAGCGTTATCCGGAATGATTGGGCGTAAAGCGTCTGTAGGTG

GCTTTTTAAGTCCGCCGTCAAATCCCAGGGCTCAACCCTGGACAGGCGGTGGAAACTACC

AAGCTGGAGTACGGTAGGGGCAGAGGGAATTTCCGGTGGAGCGGTGAAATGCGTAGAGAT

CGGAAAGAACACCAACGGCGAAAGCACTCTGCTGGGCCGACACTGACACTGAGAGACGAA

AGCTAGGGGAGCGAATGGGATTAGATACCCCAGTAGTCCTAGCCGTAAACGATGGATACT

AGGCGCTGTGCGTATCGACCCGTGCAGTGCTGTAGCTAACGCGTTAAGTATCCCGCCTGG

GGAGTACGTTCGCAAGAATGAAACTCAAAGGAATTGACGGGGGCCCGCACAAGCGGTGGA

GCATGTGGTTTAATTCGATGCAAAGCGAAGAACCTTACCAGGGCTTGACATGCCGCGAAT

CCTCTTGAAAGAGAGGGGTGCCTTCGGGAACGCGGACACAGGTGGTGCATGGCTGTCGTC

AGCTCGTGCCGTAAGGTGTTGGGTTAAGTCCCGCAACGAGCGCAACCCTCGTGTTTAGTT

GCCATCGTTGAGTTTGGAACCCTGAACAGACTGCCGGTGATAAGCCGGAGGAAGGTGAGG

ATGACGTCAAGTCATCATGCCCCTTATGCCCTGGGCGACACACGTGCTACAATGGCCGGG

ACAAAGGGTCGCGATCCCGCGAGGGTGAGCTAACCCCAAAAACCCGTCCTCAGTTCGGAT

TGCAGGCTGCAACTCGCCTGCATGAAGCCGGAATCGCTAGTAATCGCCGGTCAGCCATAC

GGCGGTGAATTCGTTCCCGGGCCTTGTACACACCGCCCGTCACACTATGGGAGCTGGCCA

TGCCCGAAGTCGTTACCTTAACCGCAAGGAGGGGGATGCCGAAGGCAGGGCTAGTGACTG

GAGTGAAGTCGTAACAAGGTAGCCGTACTGGAAGGTGCGGCTGGATCACCTCCTTTTCAG

GGAGAGCTAATGCTTGTTGGGTATTTTGGTTTGACACTGCTTCACACCCAAAACAAAAAG

AAGGGAGCTACGTCTAAGTTAAACTTGGAGATGGAAGTCTTCTTTCGTTTCTCGACGTTG

AAGTAAGACCAAGCTCATGAGCTTATTATCCTAGGTCGGAACAAGTTGATAGGATCCCCT

TTTTTACGTCCCCATGTCCCCCCGTGTGGCGACATGGGGGCGAAAAAAGGAAAGAGAGGG

ATGGGGTTTCTCTCGCTTTTGGCATAGCGGGCCCCCAGTGGGAGGCTCGCACGACGGGCT

ATTAGCTCAGTGGTAGAGCGCGCCCCTGATAATTGCGTCGTTGTGCCTGGGCTGTGAGGG

CTCTCAGCCACATGGATAGTTCAATGTGCTCATCGGCGCCTGACCCTGAGATGTGGATCA

TCCAAGGCACATTAGCATGGCGTACTCCTCCTGTTCGAACCGGGGTTTGAAACCAAACCT

CTCCTCAGGAGGATAGATGGGGCGATTCGGGTGAGATCCAATGTAGATCCAACTTTCGAT

TCACTCGTGGGATCCGGGCGGTCCGGGGGGGACCACCACGGCTCCTCTCTTCTCGAGAAT

CCATACATCCCTTATCAGTGTATGGACAGCTATCTCTCGAGCACAGGTTTAGGTTCGGCC

TCAATGGGAAAAGAAAATGGAGCACCTAACAACGCATCTTCACAGACCAAGAACTACGAG

ATCACCCCTTTCATTCTGGGGTGACGGAGGGATCGTACCATTCGAGCCGTTTTTTTTTCA

TGCTTTTCCCGGAGGTCTGGAGAAAGCTGCAATCAAGAGGATTTCCCTAATCCTCCCTTC

CCGAAAGGAAGAGCGTGAAATTCTTTTTCCTTTCCGCAGGGACCAGGAGATTGGATCTAG

CCGTAAGAAGAATGCTTGGTATAAATAACTCACTTCTTGGTCTTCGACCCCCTCAGTCAC

TACGAACGCCCCCGATCAGTGCAATGGGATGTGTCTATTTATCTATCTCTTGACTCGAAA

TGGGAGCAGGTTTGAAAAAGGATCTTAGAGTGTCTAGGGTTGGGCCAGGAGGGTCTCTTA

ACGCCTTCTTTTTTCTTCTCATCGGAGTTATTTCACAAAGACTCGCAGGGTAAGGAAGAA

GGGGGGAACAAGCACACTTGGAGAGCGCAGTACAACGGAGAGTTGTATGCTGCGTTCGGG

AAGGATGAATCGCTCCCGAAAAGGAATCTATTGATTCTCTCCCAATTGGTTGGACCGTAG

GTGCGATGATTTACTTCACGGGCGAGGTCTCTGGTTCAAGTCCAGGATGGCCCAGCTGCG

CCAGGGAAAAGAATAGAAGAAGGATCTGACTACTTCATGCATGCTCCACTTGGCTCGGGG

GGATATAGCTCAGTTGGTAGAGCTCCGCTCTTGCAATTGGGTCGTTGCGATTACGGGTTG

GATGTCTAATTGTCCAGGCGGTAATGATAGTATCTTGTACCTGAACCGGTGGCTCACTTT

TTCTAAGTAATGGGGAAGAGGACCGAAACATGCCACTGAAAGACTCTACTGAGACAAAGA

TGGGCTGTCAAGAACGTAGAGGAGGTAGGATGGGCAGTTGGTCAGATCTAGTATGGATCA

TACATGGACGGTAGTTGGAGTCGGCGGCTCTCCCAGGGGTCCCTCATCTGAGATCCCTGG

GGAAGAGGATCAAGTTGGCCCTTGCGAACAGCTTGATGCACTATCTCCCTTCAACCCTTT

GAGCGAAATGCGGCAAAAGAAAAGGAAGGAAAATCCATGGACCGACCCCATCATCTCCAC

CCCGTAGGAACTACGAGATCACCCCAAGGGCGCCTTTGGCATCCAGGGGTCACGGACCGA

CCATAGAACCCTGTTCAATAAGTGGAACGCATTAGCTGTCTGTTCTCAGGTTGGGCAGTA

AGGGTCGGAGAAGGGCAATCACTCATTCTTAAAACCAGCGTTCTTAAGACCAAAGAGTCG

GGCGGAAAGGGGGGAAAGCTCTCCGTTCCTGGTTCTCCTGTAGCTGGAACCTCCGGAACC

ACAAGAATCCTTAGTTAGAATGGGATTCCAACTCAGCACCTTTTGAGTGAGATTTTGAGA

AGAGTTGCTCTTTGGAGAGCACAGTACGATGAAAGTTGTAAGCTGTGTTCGGGGGGGAGT

TATTGTCTATCGTCGGCCTCTATGGTAGAATCAGTCGGGGGGCCTGAGAGGCGGTGGTTT

ACCCTGCGGCGGATGTCAGCGGTTCGAGTCCGCTTATCTCCAACTCATGAACTTAGCCGA

TACAAAGCTATATGATAGCACCCAATTTTTCCGATTCGGCGGTTCGATCTATGATTTATC

ATTCATGGACGTTGATAAGATCCATCCATTTAGCAGCACCTTAGGATGGCATAGCCTTAG

AAGGGCGAGGTTCAAACGAGGAAAGGCTTACGGTGGATACCTAGGCACCCAGAGACGAGG

AAGGGCGTAGTAATCGACGAAATGCTTCGGGGAGTTGAAAATAAGCATAGATCCGGAGAT

TCCCGAATAGGGCAACCTTTCGAACTGCTGCTGAATCCATGGGCAGGCAAGAGACAACCT

GGCGAACTGAAACATCTTAGTAGCCAGAGGAAAAGAAAGCAAAAGCGATTCCCGTAGTAG

CGGCGAGCGAAATGGGAGCAGCCTAAACCGTGAAAACGGGGTTGTGGGAGAGCAATACAA

GCGTCGTGCTGCTAGGCGAAGCAGCACGAATGCTGCACCCTAGATGGCGAAAGTCCAGTA

GCCGAAAGCATCACTAGCTTACGCTCTGACCCGAGTAGCATGGGACACGTGGAATCCCGT

GTGAATCAGCAAGGACCACCTTGCAAGGCTAAATACTCCTGGGTGACCGATAGCGAAGTA

GTACCGTGAGGGAAGGGTGAAAAGAACCCCCATCGGGGAGTGAAATAGAATATGAAACCG

TAAGCTCCCAAGCAGTGGGAGGAGCCAGGTCTCTGACCGCGTGCCTGTTGAAGAATGAGC

CGGCGACTCATAGGCAGTGGCTTGGTTAAGGGAACCCACCGGAGCCGTAGCGAAAGCGAG

TCTTCATAGGGCAATTGTCACTGCTTATGGACCCGAACCTGGGTGATCTATCCATGACCA

GGATGAAGCTTGGGTGAAACTAAGTGGAGGTCCGAACCGACTGATGTTGAAGAATCAGCG

GATGAGTTGTGGTTAGGGGTGAAATGCCACTCGAACCCAGAGCTAGCTGGTTCTCCCCGA

AATGCGTTGAGGCGCAGCAGTTGACTGGACATCTAGGGGTAAAGCACTGTTTCGGTGCGG

GCCGCGAGAGCGGTACCAAATCGAGGCAAACTCTGAATACTAGATATGACCTCAAAATAA

CAGGGGTCAAGGTCGGCCAGTGAGACGATGGGGGATAAGCTTCATCGTCGAGAGGGAAAC

AGCCCAGATCACCAGCTAAGGCCCCTAAATGATCGCTCAGTGATAAAGGAGGTAGGGGTG

CAGAGACAGCCAGGAGGTTTGCCTAGAAGCAGCCACCCTTGAAAGAGTGCGTAATAGCTC

ACTGATCGAGCGCTCTTGCGCCGAAGATGAACGGGGCTAAGCGATCTGCCGAAGCTGTGG

GATGTAAAAATACATCGGTAGGGGAGCGTTCCGCCTTAGAAGGAAGCCCCCGCGCGAGCA

GTGGTGGACGAAGCGGAAGCGAGAATGTCGGCTTGAGTAACGCAAACATTGGTGAGAATC

CAATGCCCCGAAAACCTAAGGGTTCCTCCGCAAGGTTCGTCCACGGAGGGTGAGTCAGGG

CCTAAGATCAGGCCGAAAGGCGTAGTCGATGGACAACAGGTGAATATTCCTGTACTACCC

CTTGTTGGTCCCGAGGGACGGAGGAGGCTAGGTTAGCCGAAAGCTGGTTATCGGTTCAAG

AACGTAAGGTGCCCCTGCTTTTTCAGGGTAAGAAGGGGTAGAGAAAATGCCTCGAGCCAA

TGTTCGAGCACCAGGCGCTACGGCGCTGAAGTAACCCACGCCATACTCCCAGGAAAAGCT

CGAACGACCTTTAAACAAAAGGGTACCTGTACCCGAAACCGACACAGGTGGGTAGGTAGA

GAATACCTAGGGGCGCGAGACAACTCTCTCTAAGGAACTCGGCAAAATAGCCCCGTAACT

TCGGGAGAAGGGGTGCCTCCTCACAAAGGGGGTCGCAGTGACCAGGCCCGGGCGACTGTT

TACCAAAAACACAGGTCTCCGCAAAGTCGTAAGACCATGTATGGGGGCTGACGCCTGCCC

AGTGCCGGAAGGTCAAGGAAGTTGGTGACCTGATGACAGGGGAGCCGGCGACCGAAGCCC

CGGTGAACGGCGGCCGTAACTATAACGGTCCTAAGGTAGCGAAATTCCTTGTCGGGTAAG

TTCCGACCCGCACGAAAGGCGTAACGATCTGGGCACTGTCTCGGAGAGAGGCTCGGTGAA

ATAGACATGTCTGTGAAGATGCGGACTACCTGCACCTGGACAGAAAGACCCTATGAAGCT

TCACTGTTCCCTGGGATTGGGTTTGGGCCTTTCCTGCGCAGCTTAGGTGGAAGGCGAAGA

AGGCCTCCTTCCGGGGGGGCCCGAGCCATCAGTGAGATACCACTCTGGAAGAGCTAGAAT

TCTAACCTTGTGTCAGGACCTACGGGCCAAGGGACAGTCTCAGGTAGACAGTTTCTATGG

GGCGTAGGCCTCCCAAAAGGTAACGGAGGCGTGCAAAGGTTTCCTCGGGCCGGACGGAGA

TTGGCCCTCGAGTGCAAAGGCAGAAGGGAGCTTGACTGCAAGACCCACCCGTCGAGCAGG

GACGAAAGTCGGCCTTAGTGATCCGACGGTGCCGAGTGGAAGGGCCGTCGCTCAACGGAT

AAAAGTTACTCTAGGGATAACAGGCTGATCTTCCCCAAGAGCTCACATCGACGGGAAGGT

TTGGCACCTCGATGTCGGCTCTTCGCCACCTGGGGCTGTAGTATGTTCCAAGGGTTGGGC

TGTTCGCCCATTAAAGCGGTACGTGAGCTGGGTTCAGAACGTCGTGAGACAGTTCGGTCC

ATATCTGGTGTGGGCGTTAGAGCATTGAGAGGACCTTTCCCTAGTACGAGAGGACCGGGA

AGGACGCACCTCTGGTGTACCAGTTATCGTGCCCACGGTAAACGCTGGGTAGCCAAGTGC

GGAGCGGATAACTGCTGAAAGCATCTAAGTAGTAAGCCCACCCCAAGATGAGTGCTCTCC

TATTCCGACTTCCCCAGAGCTTCCGGTAGCACAGCCGAGACAGCGACGGGTTCTCTGCCC

CTGCGGGGATGGAGCGACAGAAGTTTTGAGAATTCAAGAGAAGGTCACGGCGAGACGAGC

CGTTTATCATTACGATAGGTGTCAAGTGGAAGTGCAGTGATGTATGCAGCTGAGGCATCC

TAACAGACCGGTAGACTTGAACCTTGTTCCTACATGACCTGATCAATTCGATCAGGCACT

CGCCATCTATTTTCATTGTTCAAATCTTTGACAACACGAAAAACCATTGTTCAACTCTTT

GACAACATGAAAAAACCAAAAGCTCTGCCCTCCCTCTCTATCGGATGGAAGGGCAGAGGC

CTTTGGTGTCCCCTCCAGTCAAGAATTGGGGCCTCACAATCACTAGCCAATAGGCTTTTC

TCTCATGCCTTTCTTCGTTCATGGTTCGATATTCTGGTGTCCTAGGCGTAGAGGAACCAC

ACCAATCCATCCCGAACTTGGTGGTTAAACTCTACTGCGGTGACGATACTGTAGGGGAGG

TCCTGCGGAAAAATAGCTCGACGCCAGGATGATAAAAAGCTTAACACCTCTCATTCTTAT

TCCTTTTTCAACGAAAAAAATGAAAAATAAAAAGGTCGTCTTATTCAAAACCCCAATTAT

GACATCCCTTCTCTCCCACTTCACACCTCGGAACGCACCGTTCTTATAGAGATAAACGCG

CTTTCACATCTTCTTAACCCGAAATGAAATGGCTGGGGAGGGGAGAGGAAAGGTTCCTTT

TTTTTGAGGGTACTCCCGGGAACAGATCCAGTGGAGACGGGGTGGGGCCTGTAGCTCAGA

GGATTAGAGCACGTGGCTACGAACCACGGTGTCGGGGGTTCGAATCCCTCCTCGCCCACA

ACCGGCCCAAAAGGGAAGTACCTTTCCCTCTGGGGGTAGGAAAATCATGATCGGGATAGC

GGACCAAAAGCTATGGAACTTGGGTGTGGGTCTTTGGAATGGCTTTTTCTTTTTATTTAT

TATTTATCGTGAATGATGGAATCATTACAAATAGTATG-CCCCCGGCCCATCCGCGTATT

TTTTTGTTTTACGCCCCGTAACTCTTCCTCAGCCAGGCTTGGGCAGAATAGCAGAGCAAG

TACAAGTATTAGTAGCATAACAAAAATGCCTTCCTCGTCATTAATATGTTTGCTCGCGGC

AATTGTGAACTCTCGGGAGAATGATGCAGTGCTAGTACATCTGAGAATTCTTAATTGGCT

AGTTGTAAATAGCCCCAGGGCTATGGAACAAAGGATTATCCCGGACCTACACCGAGGTAT

TGACGGTGATTTTCAAATCTCGCAGAACAGAATGTGATACGATGAGATAGAAACAAAGAC

AGGGAACAGGTTACCTACTCTTAACGGTCAAAGCGAGCCCCTTTATTCTGAATTCGTTAA

TTCAGAATGAATCAAATCTCCCCAAGTAGGATTCGAACCTACGACCAATCGGTTAACAGC

CGACCGCTCTACCACTGAGCTACTGAGGAACAACGGGAGATTAGATCTCATAGAGTTCAA

TTCCCGTTCTCAACCCATGACCAATATGAGCTCGAAGCTTCCTTCGTAACTCCCGGAACT

TCTTCGTAGTGGCTCCCTTCCATGTCTCATTTCAGAGGGAACCTCAAAGTGGCTCTATTT

CATTATATTCCATCCATATCCCAATTCCATTCATTTAATACCCCTTTGGTGTCATTGACA

TAACAGATGTCGTTTCTAGTCTATCTCTTTCTATTTCTTTTCTATATATGGAAAGTTCAA

AAATCATCATATAATAATCCAGAAATTGCAATAGAAAAGAAATAAGGGAGGTTTGTGATG

ATTTTTCAATCTTTTCTACTAGGTAATCTAGTATCCTTATGCATGAAGATAATCAATTCG

GTCGTTGTGGTCGGACTCTATTATGGATTTCTGACCCCATTCTCCATAGGGCCCTCTTAT

CTCTTCCTTCTCCGAGCTCAGGTTATGGAAGAAGGAACCGAGAAGAAGGTATCAGCAACA

ACTGGTTTTATTACGGGACAGCTCATGATGTTCATATCGATCTATTATGCGCCAATGCAT

CTAGCATTGGGTAGACCTCATACAATAACTGCCCTAGCTCTACCATATCTTTTGTTTCAT

TTCTTCTGGAACAATCACAAACACTTTTTTGATTATGGATCTAGAAATTCAATGCGTAAT

TTCAGCATTCAATGTGTATTCCTGAATAATCTCATTTTTCAATTATTCAACCATTTCATT

TTACCAAGTTCAATGTTAGCCAGATTAGTCAACATTTATATGTTTCGATGCAACAACAAG

ATGTTATTTGTAACAAGTAGTTTTGTTGGTTGGTTAATTGGTCACATTTTATTCATGAAA

TGGCTTGGATTGGTATTAGTCTGGATACGGCAAAATCATTCTATTAGATCGAATAAGTAC

ATTCGATCTAATAAGTACCTTGTGTCAGAATTGAGAAATTCTATGGCTCGGATCTTTAGT

ATTCTCTTATTTCTTACCTGTGTCTACTATTTAGGCAGAATACCGTCACCCATTCTTACT

AAGAAATGGAAAGAAACCTCAAAAATGGAAGAAAAGGTGGAAAGTGAGGAAGAAAGAGAT

GTAGAAATAGAAACAGCTTCCGAAATGAAGGGGACTAAACAGGAACAAGAGAGATCCGCC

GAAGAAGATCCTTCTCCTTCCTTTTTTTCGGAAGAAAAGGAGGATCCGAACAAAATCGAT

GAAACGGAAGAAATCCGAGTGAATGGAAAGGAAAAAACACAGGATGAATTCCACTTTCGA

TTTACAGAGACAGGCTCTAAAAATAGCCCAGTTTATGAAGATTCTTATCTGATGAATATC

AATGAAAATCACGACAATTCCAAATTCAAAATACTTATGAAAATAAAGACCTAATAAAAA

TATTGATACACAAGATAAATAGAAGAAAAGATAAGAAGATATGCGTCCACCCCCTACATA

TTTGATACCTTCTCCTACAAAGAAACTCATAACACCAACCCCATTCGTAATTCCATCAAT

TACTTGGCGATCAAAAAAATTAGTTAATTGGGCTAATCCTCTTACCGCCCCAGTTAAGAA

TGTTGTATAAAAAGCATCTATATAAGCACGATTATATGACCAATTATATAGACCATTTAG

AATTTTGTCCCAAAGACTTCTCTTAGGACCTGTTTTAACAAATAAATTTATTAAGTCGAA

ATTTTTGAAAGAGGAATAAATGGGTTTATATAAAAAGGACGCTATAAATATTCCGAAATA

AGCTATACTAACTGAAAAAATTGCATCTTTCCAAAATTCATACCAATCAATCGAATCATT

CAACTTTTGATGTAAAAGGTTTATAGACGGAGTTAACCATTTGGTTAATATATCCAATTC

TTGATTGAAAGGAATTCCTAGAGATCCAACGAACAAAGTAAAGATGCCCAATACAAGTAA

AGGAAATAACATAGTATTGTCGGATTCATAAGGATAGGAATAATCCTTTTTATTCTCAAA

ATGAACAATAGTAATAAAAGGTTCTACCCTGTTTCTTACATTTTTATCACTTCGATATGT

CTTTTTTGAAAAAAAAGAAGAACTTTTACTATTATTCATTTTTAATAAACGAAAATTTTT

GTTAATTCTTTTGGAACCCCCTTTTCCCCATAGAGATATTGAATAGAAAGGGGTATTTTG

TTTGCCACTGTAATTTTGAAAATGAACGTTTAAATGCCCTTCAAAAGTAAGTAAATAGAT

CCGAAACATATAAAATGCGGTTAATCCTGCTGTGGTCCAAGCTATTATTGCGAAAATCGG

CGAATACAACCAACTATCATTAAGAATTTCATCTTTTGACCAAAAACAAGCAAGAGGTGG

AATACCACAAAGAGAAATTGTACCTAATAAAAAAGAAATTTGGGTAATGGGTACATGTTT

TGTTAAACCACCCATAAGAGCCATATTCTGACTTTTATCTGGAGAATAGCCAACAATAGT

TTCCATTGAATGAATAATAGATCCAGACCCTAAAAATAATAATGCTTTGGAATAAGCATG

AGTAATCAAATGAAATAAAGCACTTCGATAAGATCCCATTCCTAGAGCTAACATCATATA

ACCCAATTGAGACATGGTCGAATAGGCTAAACCCCTCTTAATGTCTTTTTGAGCAAGAGC

TAAAGTAGCTCCTAATAATACTGTTATTATTCCTATGAACGAGATTAAATTCATTATGTA

AGGTATAACTATAAAAAGAGGAAGAAGGCGAGCTACAAGAAAAATTCCCGCTGCTACCAT

AGTAGCAGCATGTATAAGAGCCGAAATAGGAGTAGGTCCCTCCATAGCATCAGGTAACCA

TACATGAAGGGGAAATTGTGCAGATTTCGCAACTGCACCGGTAAATAATAGAGCAGCACA

CAAAGTAACAAATGAAGAATTGACTTTATTATTATAAATCAAGTTATTTAATATTTCGAA

TAAATCTCGAAATTCGAAACTACCTGTTATCCAATAAAAACCTAAAATTCCTAATAATAA

ACCAAAATCCCCTACACGATTAGTTACAAACGCTTTTTGACAAGCATTTGCCGCAATAGG

TCGTGTGAACCAAAACCCTATTAATAGATAGGAACACATTCCAACCAATTCCCAAAAAAT

ATAAATTTGTATCAAATTTGAACTAGTAACTAATCCTAACATGGAAGTACTGAAAAAACT

CATATAAGCAAAAAATCTCAAGTATCCTTGATCATGAGCCATATAATTATCACTATAAAT

AAGAACCATAATTCCAACAGTAGTGATTAAAATTGACATAATAGAAGTAAGTGGATCGAT

CAAGTAGCCGAATTCTAAAGAAAAATCATTATTGATGGTCCAAGACCATACATATTGATA

GATACAACTACTATTTATTTGCTGAATAGACAGATTAATTGAAAAAATCATGACTATACT

TAACAATAAAATACTCGGAAAGGCCCACATACGACGAAGATTTTTTGTTGCCGTCGGAAA

AAGAAGAAGTCCGACTCCTATTAACATAGGAACTGGAAGTGGAACAAAAGGTATGATCCA

CGCATATTGATATGTCTGTTCCATAAAAAAAGTTTTTTAATTAATTGTTTCCGATTCACC

GGATCTTACCTCTTTTGAAAGAAGTCAATAAAAAAATCAAGATATGCACTAATTTAAATA

GAATTTTCTAATTTTTGATTCTTACTTATTCTGCTAAATATTCCAAATATTCAAATCAAG

AAGTTCCAATTGGTCAAATCATATGAAAGAAAAAGATTAATTACTAGTCTCTGAAAATTA

AATAATTAGCCGTATTTTTACCGAGTTTGACCAGTTAATAG-AAAAAAA-GCATATTCTT

CTTCTTTTTTCTATTTTTAGTAATATTTTATGTCATTTTCACATATCTTTAACCTATCTA

TTTTTTT-T-ATTTTATAACAAAATATTTTGTAGAAAAGAAATTCCTAATGAATAGGAAA

GTACGGATTCATTTTGAATAATAGATGTCTTTCACATCCAGCTATAACAATGAGCAATCT

CTTAATTTTTATTTAAATGGCAGTTCCAAAAAAACGTACTTCTGCATCAAAAAAGCGTAT

TCGTAAAAATATTTGGAAAAGGAAAGGATATGGGACAGCGTTAAAAGCATTTTCCTTAGG

GAAATCTCTTTCTACCGGGAATTCAAAAAGTTTTTTTGTGCG--AAAAAAAAATAAGTAA

TAAAACGTTGGAATAATCTGAATCGACTTGACTCAAAAAATTGACTCATTTCGAAAATCA

AATTAATGAATTCCATTACCTATTGAGTAAATCAATATGAAATGGAATTCGCTCCGCTCT

TAGAATATGTAGAATAAGAATTCTTCTGTTTACCTTACTCAATTCAATTCA-AAAAAAGA

AAAAAGACTTTCTTTTTGTTGACAAAAGAATAAACAAAAGATACTCCATTTTCTTTTTAG

TATATTTTCTCATTTCCAAGGCGGGGAGTCTTATTTTCCGCATCTCCCTATTTGTTACAA

TAATACAACTTTTTCTTTTTTCTCTATATCCACTTTTTCTCTCTATCTCAAGTTGAGGAG

AGTATAAAATACACGAAAATCTTAAGAAAACTAAGGCCCTAAACTAAAATAATGAACCTT

TAAATACCCATTTGAACTAATTTTTATTCATCCATTTGAATCTTTCCTAAAAAATATTGC

AACCAATTGAATTCTTAAATCTAGACGATTGCTTATTCATAGGCTATTATGAGTTCAAGA

CAAGCCGCTATGGTGAAATTGGTAGACACGCTGCTCTTAGGAAGCAGTGCTAGAGCATCT

CGGTTCGAGTCCGAGTGGCGGCATGCCATCTTCTAAAAAAACTAAATAGATCCTATAATG

AATTCAATTCCTGATTTCTTGTAATTAAAGAACTCCTATTTTAAATTTTTATGATATTTT

CAACCTTAGAGCATATATTAACTCATATTTCTTTTTCGATTGTTTCAATTGTAATTACAA

TTCATTTGATAACCTTTTTAGTCGATGAAATCATAAAACTCTACGATTCATCAGAAAAGG

GCATGATAATGACTTTTTTCTGTATAACAGGATTATTAGTTACTCGTTGGATTTATTCGG

GACATTTTCCACTAAGTAATTTATATGAATCATTAATCTTCCTTTCATGGAGTTTCTCCC

TTATTCATATAGTTCCATATTTCAAAAAAAATAAAAATTTTTTAAGCGCAATAATCGGCC

CAAGTGCTATTTTTACCCAAGGCTTTGCTACTTCAGGTCTTTTAACTCAAATACACGAAT

CTGAAATATTAGTACCCGCTCTTCAATCCGAGTGGTTAATAATGCACGTAAGTATGATGA

TATTGGGCTATGCAGCCCTTTTATGTGGATCATTATTATCAGTAGCACTTCTAGTCATTG

CAGTTAGAAAAAGCAGAAAGTTTTTTTTTACAAGTAATCATTTATTAAATTTAAATGGGT

CATTTTTCTTTAGTGAAATCGAATACATGAATGAAAGAAGCAATGTTTTAAAAAATACTT

CTTTTTTTTCTCCGAAGAATTATTACAGGTCGCAATTTATTCAACAATTGGATTATTGGA

GTTATCGGGTTATTAGTCTAGGATTTATCTTTTTAACCATAGGGATACTTTCTGGAGCAG

TATGGGCTAACGAAGCATGGGGATCATATTGGAGTTGGGACCCAAAGGAAACTTGGGCAT

TTATTACTTGGATCGTATTCGCGATTTATTTACATATTCGAACCAATATAAAATGGAAGG

GTATAAATTCCGCAATTGTGGCGTCTATTGGCTTTCTTATAATTTGGATATGCTATTTTG

GGGTCAATCTATTAGGAATAGGACTACATAGTTATGGTTCATTTACATTAACATCTAATT

GAATTCAAAAGGATTCAAAAAAGACTCTTACGAATAGAAAAGTGTACAGTGCATATGAGA

TAAAAAAACTTTATGTGAACTGATGAGAACCCTGTGAATCAAATATTGTAGTGATTCACA

GGGTTCGCGCCGATTCAATTTTTTTACTTAACTTAAGAGAAGAAGAAAAAGCCTTCTTTT

TTTCATTGTACAACGAACGATT-AAAAAAAAATCATAGGA--TTTTTTTTTATTCATCAA

AACTATCTATAAAAAGAATTAGATAGAATAACTTCGACCTTGTCAACTGATAGTGAAAGA

ACAAAATCGGGGTACATACCAATACCTAGTATGGGTAAAAAGATAGAGATTGAAAGAAAT

AACTCTCGCGGTCCAGAATCAAAAAAATAAGAGTTTGGAGCATTAAATATCTTGTATCCA

TAGAACATCTGGCGTGACATAGATAATGAATAAATAGGAGTTAATATCATTCCAATTGCC

ATTACAAAAGTAATTAGTATTTTTGGCATTAAAAGGTATTTTTGACTGGTAATTAGTCCA

AAAAAGACTATTAATTCGGCAACAAAACCACTCATACCTGGTAATGCAAGGGAGGCCATC

GAAAAGCTACTGAACATCGTGAATATTTTTGGCATTGGGATAGCTATTCCACCCATTTCG

TCAAGATAAACAAGACGTATTCTATCATAAGTCGTTCCTGCCAAGAAAAAAAGTGCAGCA

CCAATAAATCCATGAGAGATTATTTGTAAAAGGGCTCCATTGAGCCCTGTATCGGTGATA

GAACCAATTCCTATAATTATGAAACCCATATGAGATACAGAGGAATAGGCTATTCTTTTT

TTTAAATTCCGTTGACCGAGAGATGTTGAAGCTGCATAAATTATTTGCATTGCGCCTACT

ATCATCAACCAAGGAGAAAATATAGAATGAGCATGTGGTAATAATTCCATATTGATCCGA

ACTAATCCATACGCTCCCATTTTTAATAAGATTCCGGCTAGAAGCATACAAGTACTATAA

TGCGCTTCTCCATGGGTATCTGGTAACCATGTATGTAGGGGTATAATCGGCAATTTGACA

GCAAAAGCAATAAAAAATCCAATATAAAATATTATTTCCAAGGCCACAGGATAGGACTGA

TTGGCTAATATTTCTAAATTTAATGTTGGTTCATTAGAACCATATAAACCGATACCCAGA

ACTCCCATTAAAAGAAAAACAGAACCCCCCGCCGTGTACAAAATAAATTTGGTAGCTGAG

TACAGACGTTTTTTTCCTCCCCACATTGATAGAAGTAGATAAACGGGAATTAATTCTAAC

TCCCACATGAGGAAAAAAAGTAAAAGGTCCCGACAAGAAAATAATCCTATTTGACCGCTG

TACATTGCTAACATCAAGAAATGAAATAGTCGAGAATCCCGAGTAACTGGCCGAGCCGCT

AAAGTAGCTAAAGTAGTGATGAATCCCGTCAGTAAAATGGGTCCTATAGAAAGGCCATCT

ATTCCTAATCTCCAATGGAAATCAAAAAAATCGATCCATTTATAATCCTCCACTAGTTGG

ATTAATGGATCGCCCGATTGGAAATGATAACAGAATGCATAAGTCGTTATAAGAAGTTCT

ACAATACATATACATATTGTATACCAACGAATTCCCCTATTTCCTCTATGGGGAAGAAAG

AAAATTAAGGAACCCGCAGATATTGGCAAAATAACAATTATTGTTAACCAAGGAAAATGA

TTCGTGGTAAAGACAAGATACACTTGGGCCAGAAAAACCCGTGCTCAAAATATTTTGAGC

ACGGGTTTTGTCGGTAAAAAAATCAAATGGATTCAAGTAGAGTTTTCTGGAACGTATCAA

TAAGCTAGACCCATACTGCGAGTTGTTTCATGCCATAAATAAACTCGAACACTCAAGAAA

TCCGTTGGACAGGCGGATTCACATCTCTTACAACCAACACAGTCCTCGGTCCTTGGGGCA

GAAGCAATTTGTTTAGCTTTACATCCGTCCCAAGGTATCATTTCTAATACATCGGTGGGA

CACGCTCGGACACATTGAGTACATCCTATACATGTATCATAAATCTTTACTGAATGTGAC

ATTGGATCTATACATTTTTTTCGTCATAAATTTTCGATCTAGTAAACTTATAAATGAATC

ATATATTTAGATACCAGACGAATCAATGAGTGATCAGAATCAATCTACTTGCGGATTGGT

TTATGAGAGAGGGCCAAAATACTTTGATTTCTTATGTTTTTGCAACCACGATCCTACCTT

ACACGTAGCAAACCTGCTAATTCGAATTCATTTCAGAATAATTTCTGAATATTCGAATTT

CTATTTATATAATTAATACTATTTATTCAACAAATTGGATTGGTTAATACGAGTTGATTT

TCTGTTACGATAAATTGATGAAACAATAGCCGGTCCAATAGCTGCTTCAGCGGCTGCAAT

AGCTATAACAAAAATGGAGAAAATATCTCCTCTTAATTGACGATTATCAAAAAAACCAGA

AAATGTTACAAAATTTATATTAACTGAATTTAATATAAGTTCAAGACACATAAGGGCTCT

AACCATATTTCGACTTGTGATCAATCCGTAGATACCGATGGAAAATAAATAGGCACTCAA

AACAAGTACATGTTCAAGCATCATTAACCAACTCCTTATCAATCTCGATTCATTTCAATA

TGAACAATAATTCAAGCGATTCGATTCTAACAACAGGGAATATATTGGTAATAGATCTAC

CTAAAGCTAAAGCATTTCTATTTTAGACAGGAATTCAAATTAAAGGATTATAACATTCAT

TTCCGTTGATTTTATTTGAATGGAATTCCTAGTTTTAAAGATTTATTATTGACGAGCTAT

TGCAATTGCACCTATTAAAGCGACTAAAAGAATTATTGAAATGAGTTCAAATGGAAGAAA

AAAATCTGTTGATAAATGAATTCCAATTTGTTGGCTATTACTTATCAAATCTTGCTCTAT

AATCTGGTTTGATTTTGTAGTCCAAATAATCCCGTACCATGACGTATCCGGAATAGTAGT

AATTAGTGAAATAAAAAGACTTGTACAAACTATTGAAGTAACTCCATCCCCAACGGTCCA

AAGATGAAAATCTTTGTAATATTCTGAACCACTCATGAACATCACAGCAAAAATGATTAA

AATATTTATAGCTCCCACATAAATAAGGAGCTGCGCAGCAGCTACAAAATACGAGTTCGA

TAGAATATAGAATAAGGATATACAAAAAAGAACCAATCCCAACGAAAAGGCCGAATAAAT

TGGATTGGGAAGTAATACTACTCCTAGACTTCCTAATATAAGACCCGATCCCAGAAAGAC

TAAAAGAAAATCATGTATTGGTCCAGGTAAACCCATTTGATTTTATAGAAAAAA-ATAGA

TAAATCGAAATATTTCATGACTTTGTTGACCTGACCAGGAAAAAAGAAGTTATCTTTGAG

GATACTTCTTAATTGAATTAAATTGGAATGGGTGTGATGTGGATTGATGTAGATACAGTT

ATGGGACTACTCTTATTCTTTATCCGAAAGAGGAGTTTGAAACTCTCTATTTTGAAATCA

TTTGAATCAAGGATTTTTTCCATTTTTTATTTGAGGTGAATTCGAAATTGTTCGAATTGT

GTAATCGTCAATTATTGACACCGGTAACCGACCTAAAGCAATTTGATTATAATTCAATTC

GTGACGATTATAAGTAGAAAGTTCATATTCTTCAGTCATTGATAAACAATTTGTTGGACA

ATACTCAACGCAATTACCACAAAATATACAGATTCCAAAATCAATACTGTAATTAAGCAA

TCGTTTCTTTCGAATATCCGTTTCCAATTTCCAATCAACAACGGGTAAATCTATAGGACA

TACACGAACACATACTTCACAAGCAATGCATTTATCAAATTCAAAGTGGATTCGTCCTCG

GAAACGTTCCGATGTGATCAATTTTTCGTAGGGGTATTGAATAGTTACAGGTAAACGATT

CACATGGGACAAGGTAATCATGAAACCTTGACCAATGTACCTTGCAGCTCGTATTGTTTG

TTGACCATAATTCATGAACTCAGTTACCATAGGAAACATATCGTAAATATCTATAAATAA

TTTTATACTTGTTTCTTTCTCTTGTTTGAGACAAGTTGTGAATATAGAATATTCTATTCC

TTTACAGTGAAAGAAGTTGGAATGAAGTTGTTAATAATAGATTACCTAGAGAAATAGGTA

AAAGAAATTTCCATCCAAGATTTAATAGTTGGTCCATTCTCAGTCTCGGTAAAGTCCATC

TTGTTGCAATAGAAATGAACAAGAACAAATAAGTTTTAGCTAATGTAATAAAGATACCGA

TTATTGTTCCAAAAACTTTACCTCTTTCAAAAAGCTCAGGAACGAATATGTACGGAATAG

AAAGATTCCAACCCCCCAAGTAAAGAACTGTTACAAATAATGAAGAAACTAGTAGATTCA

GATACGAAGCAACATAAAATAAACCAAATTTGATACCTGAATATTCGGTTTGATAACCTG

CTACTAATTCCTCTTCTGCTTCTGGTAAATCAAAAGGTAATCTCTCACACTCGGCTAGAG

AAGAAATTAGAAAAACGATAAACCCTATAGGTTGACGCCACAAATTCCACCCCCAAAAAC

CATATTTTGACTGCGCTTCAACTATATCAACTGTACTTGAACTGTTAGATAATCATAGTC

GATGATAACATCACTGTTCCCATCGCTATTACAGAACCGTACATGAGATTTTCACCTCAT

ACGGCTCCTCAGAGGTCGCAAATAAATCTAAGTACCTTTCGACATTATTTATCTTGATAT

GTTTATAGGATAGATAGAGATTCAAACTCTTATCCTAAGGTAGACCAATGGAATTCTGTC

TGCTATTTCTAAGAAATATATATTTCTTAGAAATAAAAGTGCTTCTGAATTGATCTCATC

TTTTAAGAATTTTCATTTTTCTTTGTTGATTAATAACTTTATCCTTGAATAAAAAAAGAG

TTTTTTTGGAGGAATATCACATAGCTATTTCAACCTATCATTTTCCATTACGAAAAAGAA

TTAGACATTGCATTAGTTCATGCATCATGACAAGAATTCTATCTCTCTAAATAGGTATAT

AGGAAAGAAATAAT-AAAAAAAATCTCTTTTTTGCAATTCTATTCTTTCGAGTTTATTTC

GTTCCTATTCTCCTTTCTCAAAAAAGGGGGACATTACCAAAGTAAAAGATTACTTCGTTC

TTGATAGTTATTTACTTAATCAGTGGATAGGAACATACTCTGGATCGAAATCATGGGGAG

TACTTCTTAATCGTTTCTACCAACTTAAAGCCCCAATTTGAATTCCTTTTAGGTACATCC

TGTTGGATAATTTACAGAATCTCCATTACTAATCCTTTGCGTATCTTGGTCTTCCTAACC

ATCCACTCATTTTTGTTAACCTTCCATTATGGTAATACATCTATGTTAATAGATAGTAAA

AACTCCATACAGTTGATCTTTTGAACCCGTTTCAAGCCATGATGCCTAATCAACCAATCT

TGGGGTAAACAGTCTCGACTGCTTTGCTTATATTTACTTTCATTTCATTCTTGTACATAG

GAAATGAGATTCAATCCCTTTAACTGCAAAT-AAAAAGCCGTTTTATTTCACTCATATAA

CTATCTGGTTTAGTTCATCAACCCGAATTCTGAATAAAAAAAAATATATATATTAAACTC

ATTTAACTTTCTTACTAGAAAGAAAAGAAATAGGGGAAATTTTATGTCTCACCGAATCAC

ACGTAGAGATATTGATAATACACATAGAGTTAATGGTATTTCATAACTAATTGATTGAGC

AGCAGCTCTTAAACCACCTAAAAAGGAATATTTATTATTTGATCCATATCCCGACATAAG

AAGTCCAACGGGAGCAAGACTTGAAACAGCGATCCATAAAAAAACACCAATACTAAGATC

GGCTAGAATAAGGCGATAACCAAAAGGAATTACTGAATAACTTAGTAAAATGGATATGAC

TGCTATGGATGGTCCGATACTGAATAAACGAGTATCCCCTCTAGATGGAAAAAGATTCTC

TTTGAAAAGTAGTTTTACCCCATCTGCTAGAGCTTGAAGAATTCCCAAGGGGCCGGCGTA

TTCAGGTCCAATACGTTGTTGTATCCCTGCAGATATTTCTCTTTCTAACCAAACAATTAC

TAGTACACCTAGTGTGATTCCTAATACAAGAGTCAAAATAGGGACAAGCACCCATATGAT

CCCATAGACTTCTTTTAAGAATTCCAATCTGGAAAACGAATGGATGGCTTGTAGTTCTGT

TGTATTATCAATTATCATTTCAACGATCAACTTCTCCCATAATGATATCTATACTACCTA

GTATCGTCATAATATCAGCCAATTTCATTCTTTTAACTAACTGAGGCAGAATTTGCAAGT

TGATAAAACCCGGTGGGCGAATTTTCCATCTCCAAGGAAAAACACTCTGATCTCCTATCA

GAAAAATTCCCAATTCTCCTTTTGGTGCTTCGACTCTTACATAAAGTTCTTGTTTGGACA

ATTCAAAAGTTGGAGAAGGTTTTTTACTAATAAATCGATATTCAAAATCATTCCATTCAG

TATCTTTTACTCTATCAAAGCGTCGGATTTCTAAATTCTCAAAGGGCCCCCCTGGAATTC

CTTCCAGAGCCTGTTGGATAATTTTTATGGATTCTGTCATTTCACTGATTCGTACTAAAT

AACGAGCTAATGAATCCCCTTCTTTTTGCCATTGAACCTCCCAATCAAATTCGTCGTAAC

ACTCATAATGATCAACTTTACGAAGGTCCCATTGTATTCCGGAAGCTCGTAGCATTGGTC

CTGATAAACCCCAATTTAGTGCTTCTTCTCCTCCAATAATGCCTACGCCCTCAACTCGTT

CTAAAAAAATAGGATTCCGTGTAATAAGCTTTTGATATTCAGCAACCCCTGTTAAAAAAT

AATCACAAAAATCCAAACATTTATCTATCCATCCATGAGGTAGATCAGCAGCTATTCCTC

CGATACGAAAATAATTATGCATCATTCGCATACCGGTGGCAGCTTCGAATAGGTCATATA

TCAATTCTCGTTCTCGAAAAATATAGAAGAAAGGGGTCTGTGCCCCAATATCTGCCATAA

AAGGACCAAGCCATAACAAATGAGAAGCTATACGACTCAACTCCAACATAATGGCTCTGA

TATAGCTAGCCCTTTTAGGTACTTGAATATTGCCTAGTTGTTCGGGTCCATTTACGGTTA

TTGCTTCTGTGAACATAGTAGCTAAATAATCCCAACGTGTTACATAAGGCAAATATTGTA

TAATTGTTCGGTTTTCCGCAATTTTCTCCATTCCTCTGTGTAAATAACCCAATATTGGTT

CACAGTCAATAACATCTTCACCATCGAGAGTAACGATAAGTCGAAGAACACCATGCATTG

ATGGGTGGTGAGGACCCATATTGACTATCATGAGGTCTTTTCTTGTAGTTGGTGCAATCA

TAAGTTTTTTACCGAGTCATTCTTCCATGAATTGCTGAAAGTAAAAAGAAGTTCATCAAA

ATTGAAACGCATAAGTTCAAATGATATCACTCTTTAAATTAACGAGTTTTTGTCTCTCGA

ATATCCAACCGATCAATTAATTCTTTATAACGTACTCTATTTTTTTTTGACAAATAAGCC

AGTAATCGTTGACGTTTTCCCAAAATTTTTCGCAAACCTCTCTGAGATGAATAGTCTTTT

TTGTGCAATTCCAAATGTGAAGTAAGTCTCCTTATCTTAGTGGTGAAACTGAATACTTGA

AATTCAACAGACCCTCTGTTTTCTTCATTTTCTTTTTGAGAAATAACCGAAATGAATGAA

TTTCTTACCATAAAAAAAAGCCTCCTCTCCCTTTTTACAGATATGGATTTTACCGATCAG

TAATAATAATGTCATTCATTTTAATGTGGTATATACAATAATCTAATTCAAATTTCTTTA

TGAACTCCTAATTTTATCAATTCAAATGAATCAATCCAATTGAAAATTAGATTTAGATAG

GGAGAGAAAAGGATTGGCACATTTTCGTATTCACAAAAGCGAAGAATTTAGACCTAAAAT

GAAGAGGGTTCTGTTGATTCATTTCTAGATCGAATTGATACATTATTCATTTAGTATTGG

ATATTTAGAATGAAATGTAAGACAGGACGTGTGATGTGTGTATTTATTTGCTTTCATATA

TCCTATATAGTAGAGGATATATAGGAAAAATGGACTATCAACGAATTTTCAATCGTGGAT

ACAAATGTATCCTTAACATACTGAAACGACTGCCATTATTGGTATCAAACCAATAGCGAT

TCATACAAGCTAAATCTTCTAATCGATAATTAGGCCAAAGAAAGAACTTCAATTTCATTA

ATTGATTTTTCTCTCTATCAAGGTGATTACTGTCACCTAGAACTTGGCTGCTATTCTTTT

CGTTGCAAAATACAGGATTTCCATCTACATCATTCCTATTCTTTGAATTGAAAGAAATTA

GAATTCTTAATTTTCTACGACGCCTAAATGAGAAAATATTTTCAGGAACAAGCAAATCCA

AATGATTTTTGTCTCTATTTCTTGTTATTCTTTCATGTGGTGGAATAGATTCATCAAAAT

TATTCTTAGCAACATATCTTTGCTCTCGGTATCTTTGATTAGTTTGGTGCTTACTCTTAT

GAACCAACAAAATACCGATGGTTTGATACATAATAAACTGTCCGTCGTTTTTTACAGACA

GACGAATGGGTTCGATAATAAATATTCCCTTTTTCATCAATTCTGGAAGAGTTAAATTCT

TCTGAATCAGCATTATATCCAAACTCATTTCTCCCCTTTGAATCGAGGATATAGAAATTT

TTCTTGGATCTATTAGTCTAAGCAGGAAACAATATACCTTAATATTATTGATCAGTCTTT

GATTCAAAGTATCGTCCCATCTCAATTGAAAAAGCAAATAACGTTTCAGGAACAAATCTA

GTTCTGCTTCCTTACTTTTGTATTGTTTTTTCTTTTTACCTTTTTTCATGTCCGATCTCG

CATAATCTTCTTCAATATCTTTTTGTTGGTTTGAAAGAACGGATCCAAGATCCCCTTGGC

TTGTGGTTTTTTTTTCTTCTTGATTTCGATTCTTTATTTTTTTATTCGATGGTATCAAAA

AACTTCCCTTTTGCTTTTCATTAATCTTTTGATTTTGATTACTATTTTCATTTCTATTCA

AATTTAAAAGAAGTAATTTGCTTGGTATAAACCAAGATTTCGTTTTATATGTATTATAAA

GTAACAAAAATTCTGGGAAGAACCAAAGTTCCAGATTCAATATGGGACGCTTTAGTATTT

TTTCATTCATCCCCATCCAATCAAAAAAGACTTTTGGGGAGTTTGGTAAATTCATTTCTG

GAATCATAAGATAAAAAAGATTTTTTTTATCAATTATTTGATAATTATTAGTAACAATCT

GAGTATTTTGATTACTATTGGCATCGATCGTGATCCAGGCCTCAATATCGACTTTTTGTC

TAAGATCAAAATTGATAATTTTCCAATCCAAATATTTCCTATCGGGAGTTTTTTCCATAT

ATAGAATATCGCCCTTTCCTAGATAATTATTGATAGGGATACTCCTCAGCATATCAAAAA

AAGTGTCTTTATATGTGTTGTAATTATAAGAAATCTCTTGATTCTTATTTCCTTCAAACG

GCGATCTAGAAATAAATGAGTCCTTTTTATTTTCAGAATTAATAGATTTATATGATAAAA

GATCATATTTATAGTATTTTGGAAAATTATCTTTTTGATTCAATAATGAATAGACTTCCA

AAATATTTTCTTTTTTGTAATCAATTAATTGGTCTTTTTCATATAAATTCCATTTGCTGA

AATTTTTCCTTTTAACCATACGACGTTGATAAACTCTATTCCGCCATTGTTGTGGTATTA

ATCTAGACCATCTGATCTGAGATAAATCGTATTGATAATGCCCTCTTAACCAGTTTTTCC

ATTGATTCATTTCAGAACTCGGAAGTCTGTTATCCCTTAATTTAGAATGAACTATTCCTT

GTGTTTCAAAAGAATCCTTTATTTCAGGCTTAAGAAAAAAAGGGATTCCTTGATATTGAA

ATGATTTTAATTTATACAAGTTAATAACTTGGGTTTGTGATAATTTGTAAAATACATATG

CTTGTGATAAGTACGATAAGTCATAAAAAATATGTGAATTTGTCTGACTATTACTAATAT

TATAAAGTGACTTTTTTATAGTCGAAATAAACTCAATTGGATTTTTTTTTTTTTTATTAA

TTATTTCTTGACTTGTTTCATTATTGTAAATGGATTTATTCATAATTTTTTTTGTTGATT

CAAGAAATAATTTTCTCTTTATTCTGGGAATATTAATGATAGATAAAAATATATTTGTGT

AGATTCTTTCAATTAAAAATTTAAAAACAAAAGGTAATTTAGAGATTAATCGAGCATTTC

TTCTTTTTAATATTTGCCATTTTTCTAATCTTTTAGCATTATAACTTGTTTTGTTAAGAC

TAATATTTATTTCTGGAGTTACTTTTTTTTTCTCTTTTGTAATTCTTTCTATTTGATTTC

TAATTGTATTTGTTTTATCAGTCAGATCCTTCATTTTTTTTTCTGTCAATGAAGAATTTG

TCCAACCTGGAGATGCAATTTGACTAAATGACTCATGAATCATCTGATTGCTGATTATGG

GATCTCTTTCTTTTTTAGTTTCACTCGAGTCATATACTTCTACTTCTCTTGATCGAAATA

AGAGAATTGGATTCACTTTTAAGAGTTCTTTTCTTATTTTTTTAAAAAAAACGACACTTT

TGATAACCCATTTTTTTGTTTCTTTTGAAACTTTTCGAAGTAATTTTATTTTTCCTTTTA

AAATTTTTAGAAGTAGAAAATATTTCTTTTTTAATTTTCCAATTTTTTTTTCGAGTTCCT

TAAAAATGGGTTCAAAAAAAGAGGGTTGTTTTCGGGGAGAACCAAAAGGAAGTTCGGTTT

CCATTCCCAAAACTGTTAAAAAACAAAAATCATCTTTTTCTTTTTTCTTTTTCATTATTA

GATCTTTATGAGAGAATCGGAGTTTAGATCTGTGCCAAGGTTTCAGACAGAAAGGAAATA

AGATTTTTATCTGAATACCATCTGTCAACCAATTTTGTGGAAATTCTGTTTCGGACAATT

GAACACCATTATAGGTACATTTAACATGCATCTCCCTATTCCATTCCTCTAAATCCTCAT

ACCATTCAGGAAGTTGCAATAGTAGCATACGTCCAATATTTTTTATTATCAATGAAGGTA

ATATAATATATTTTCTAAAAATAGATTGAGTTATTAACATGGAACCCCTTATTACTTGCG

CAAATGGAATGGTATCCCAGGTCTCTGCTATTATTCGCGCGATCTCCTTTCTTTTGTTCT

CTTTTTTTTCTTCTCTTTTGTTTGCTCTTCTGTATACTCTACAATTTTGAACGCGGACCC

CCTCCCTACCCAATTTCTAAAAATTTTAATTGGCCCAGAGATATCAAAAGAAAAAAGAAG

GGATTTTTTTATTCTGTCCAAAAAAAGAGGGGAATGCACATTTGCTTGAAACAGTTCCCA

AATAACTATTTTACGCCTTTGAGCACGTATAGAACCTTTTATTATGCCTCGCTTAAAATC

TGATTGTTGTGAATAGCGTAGCAAAGCTACTTCGTATGTTTGATCAGGAGGATTAGTATT

CTCAGTATGCTCCTTGTTACCAGTAAAAATTACTACACGTTTGGCTTTTCTTGAACGAAT

TTCATGATCTAATGGTCCGTTTTCTTTATCTTCTCCTGCTTCGTGTTCCAACTCGCTGGT

TAATTTGTATAACCAGCGAGGTACTTTTTTACTGATTTCTTTTATTCTAATCGATTTTTT

ACTAATTTTTTGAACATTAGTATCAGTTAGAATTCTATTTTTAAAATATTTCAAATATTT

TGTTCCTTTTTCTGAATCTATTCTTCCTTCTTCTTCATCTGAAAATAAAGAAAGACCCCT

AGAATTAAAATGGATCCTGATTCTTTACCAAGTTCATTGATGAAAGTTAAGAAATCAACA

ATTTCGGTTGATAATGGTTTTTTATCAAATCGATCTATTTTCTGTTCAATTTTTTGGTAA

TCAGTATCAGGAAGAACGATACCATGAATACGATTTATCCCAACTTTCTCTATGAAATTG

TCTATCGAAGTTTTTTTTATTTTTATGATTGAAGGTGAAACGCTTTTTGTTATTGTTCTC

CGATATGGTCCGTTCAAGAAAGGATCATATATTTTGGGTAAGTATTCGTTTGTAGTATTG

TCATTACACAACCTAGTCCTTGTTTCGAGTATATTCAAAAAAAGAGATTCTTTGTCTAGG

GCTTGAATTCGATTTACAAATTCGTTGTTTAAATTATTACTTTTTTCTTTGTTGGTATAA

ACCCAATGATTGTAGAGTTCATTAGATGAGAATTTTTCTAGTGTAGGCAGAGATATCCTT

CTTTTTATCATTTCCCAAAAAGTTGACAAACTGGGTGGATATGTAAAAGATATTCTTTCC

TTTCCAGCACTTTGGCATGTGTCAAAAAAATATTGTGACATTTCATTTCTGACAGCGTTT

TCAAATCGATTATTTTTTATGTATCGAAATGGCCGATTCCAACGGTTAGAATCAAAAAGA

AGAGTCACAAGAGGTTTTTCAAACCAAAAACCAAA

>Osmanthus_austrocaledonicus

TATCATTTTAGTTATGGGCGAACGACGGGAATTGAACCCGCGCATGGTGGATTCACAATC

CACTGCCTTAATCCACTTGGCTACATCCGCCCCTCTTAGATTTTTTGATTTTAAGACAAA

AGGTTGAATTTCGACCATTTCATTTATCTTCTTTCTTATTTGCTTATTTGTGAGATATTT

TTATCTCAGAGATAAAAAGATTAAGCAAAAATTAGAATTTTTCTCGTTTTAATTTAAATT

TAAATGTCAAAAAAACTTCGCAAAAGATGGATAGATAAATGAAAATAAAAATGCATATAG

AACGAACAAATAATACTCAATCATCAATCAACCCCTAAGAAAAATAATCCCCTTTATTTC

TTTTTCGGTAATGTAACGAATAAAAGTCTATGTAAGTACAATATGACTAAAAAAAGTAGT

AGTAATTAAAAAGAAAATAAAGGAGCAATAACGCCCTCTTGATAGAACAAGAAAGAGTTT

ATTGCTCCTTTATTTTC-TTTTTTTTTTTCAATAACTCCTATACACTAAGATCGGGTCTT

ATCCATTTGTAGATGGAACTTCGATAGAAGCTAGGTCTAGAGGGAAGTTATGAGCATTAC

GTTCATGCATAACTTCCATACCAAGGTTAGCACGATTAATGATATCAGCCCAAGTATTAA

TTACACGGCCTTGACTATCAACTACGGATTGGTTGAAATTGAAACCATTTAGGTTGAAAG

CCATAGTGCTGATACCTAAAGCAGTGAACCAGATACCTACTACAGGCCAAGCAGCTAGGA

AGAAGTGTAACGAACGAGAGTTGTTGAAACTAGCATATTGGAAGATTAATCGGCCAAAAT

AACCATGAGCGGCTACGATATTATAAGTTTCTTCCTCTTGACCGAATCTGTAACCTTCAT

TAGCAGATTCATTTTCTGTGGTTTCCCTGATCAAACTAGAAGTTACCAAGGAACCATGCA

TAGCGCTGAATAGGGAGCCGCCGAATACACCAGCTACGCCTAACATGTGAAATGGGTGCA

TAAGGATGTTGTGCTCAGCCTGGAATACAATCATGAAGTTGAAAGTACCAGAAATTCCTA

GAGGCATACCATCAGAAAAACTTCCTTGACCAATTGGGTAGATCAAGAAAACAGCGGTAG

CAGCTGCAACAGGAGCTGAATATGCAACAGCAATCCAAGGTCGCATACCCAGACGGAAAC

TAAGCTCCCACTCACGACCCATGTAACAAGCTACACCAAGTAAGAAGTGTAGAACAATTA

GTTCATAAGGACCCCCGTTGTATAACCATTCATCAACGGATGCCGCTTCCCAGATTGGGT

AAAAGTGCAAACCTATAGCTGCAGAAGTAGGAATAATGGCACCTGAGATAATATTGTTTC

CGTAAAGTAGAGATCCAGAAACAGGTTCACGAATACCATCAATATCTACTGGAGGAGCAG

CAATGAAGGCAATAATAAATACAGAAGTTGCGGTCAATAAGGTAGGGATCATCAAAACAC

CAAACCATCCAATGTAAAGACGGTTTTCGGTGCTGGTTATCCAGTTACAGAAGCGACCCC

ATAGGCTTTCGCTTTCGCGTCTCTCTAAAATTGCAGTCATGGTAAAATCTTGGTTTATTT

AATCATCAGGGACTCCCAAGCACACTAATTTTCTATAATATAAATCGAAATAGAAAATGG

AAGGCTTGTTATTTAACAGTATAATATGACTTATATGGTCGTGTCAACCAATCCAATCCA

ATAACTATCTAGATTTATTCGAATTTTTTGTAAATGAATGAAGTGGATTGCAAAAAGAAA

ATACAGATTTCTATACATAAAATTTCGATATGACAGTGGGTTGCCCGGGATTCGAACCCG

GAACTAGTCGGATGGAGTAGATAATTTCCTTGTTATGTTAATTAAATAAGGAAAAACCCC

TCCCCAAGCCGTGCTTGCATTTTTCATTGCACACGGCTTTCTCTATGTATACATCATTTC

CTTTCTTATAAAGACTTTCAAAAGTTGAATACTCAGTTGATTTAACCCTTATTACATACT

ACATCAACATTTCAGAATAGTGGAAATCACATTTTTATTTTGTTATCTCTTCATCCATTT

AATTTAGGAAAAATTTCTATTTCCAAGCTTTCAGAATAATTATTTATGATTGACCAGATC

ATTGATACAAATAATATCCAAATACCAAATCCGACTTCTATATACTCCCCGCAAAGTGGA

AGAAGCTTTTGGGAAGGTCAAAGAAAGAACTTGTTCTTCCGACATAAGAAATTCTTCCAA

TAATTCCGAGCCTAATCTTTTCAAAAAAGCACGTACAGTACTTTTGTGTTTCCGAGCCAA

AGTTCTAGCACAAGAAAGTCGAAGTATATACTTTATTCGATACAAACTCTTTTTTTTGGA

AGATCCGCTATGATAATGAGAAAGATTTCTGCATATACGCCCAAATCGATCAATAATATC

AGAATCTGATAAATCAGCCCGAACCGGCTTACTAATGGGATACCCTAGTACGTTACAAAA

TTTCGCTTTAGCCAATGATCCAATCAGAGGAATAATTGGAACAAGAGTATCGAACTTCTT

AATAGCATTATTGATTAGAAATGAATTTTCTAGAATTTGACTCCGTACCACTGAAGCGTT

TAGTCGCACACTTGAAAGATAGCCCACAAATTCAAGGGAATGATTAGATAATTGGTTTAG

ATAAATCCTTCTTGGATGAAACCACAGCGAAAAATAACATTGCCAAAAAGTTACAAGGTA

ATATTTCCATTTATTCATCAAAAGAGGCGTCCCTTTTGAAGCCAGAATTGATTTTCCTTG

ATACCTAACATAATGCATGAAAGGATCCTTGAACAAGCATAGATTGGTCTGAAAATCCTT

AACAAAGACGTCTACAAGACGTTCTATTTTTCCATAGAAATATATTCGTTCAAGAAGAGC

TCCAGAAGATGTTGATCGTAAATGAGAAGATTGGTTACGTAGAAAGACGAAAATAGATTC

GTATTCACATACATGAGAATTATATAAGAAGAAGAATAATCTTTGATTCCTTTTTGAAAA

AGAAGAACTGGCTTTCTTTGGAGTAATAAGACTATTCCAATTACAATACTCGTGAAGAAA

GAATCGTAATAAATGCAAAGAAGAGGCATCTTTTACCCAATAGCGAAGAGTTTGAACCAA

GATTTCCAGATGGATGGGGTGGGGTATTAGTATATCTAACACAAAATTTAAATGTGAAAA

ATTGTCCTCTAAAAAAGGAAATATTGAATGAATTGATCGTAAATTATGAGATTTTACTAT

CTTTTTTTTTTTCCCTTCTAGAGAAAGTATTAATCGTAGAGAAAATGGAATTTCCACAAT

AAATGCAAACCCCTCTGATATGATTTGAGGATACAAATTCTTGTTGCGCCCCAAAAATTG

ATTTTGGTTAGAATCATTAGCAGAAATAAGAAAATGATTCTGTTGATACATTCGAGTAAT

TAACCGTTTTACAATTAGTAAACTGAATTTATTGTCATAACCTGGATTTTCCAACAAAAT

CGATCTATTGAAACCCTGATCATGAGCAAGTCCATAAATATACTCCTGAAAGATAAGTGG

ATATAAGAAGTCGTGTTGTTGAGATCTATCTAGCTGTAAATATCTTTGGATTTCCTCCAT

TTGAAATTTTATTTGAACCAAAGTTAGAAGAATTTTGTGGGTTATCAAATGATACATAGT

GCGATACAGTCAAAACAAGGTATTCTAGTAAGAATAGATACCTCGGAAACAGGTAAACTT

ATCAACAGATTCTCTACCCTCTCTTTTTTCCATTTCATTTAATTGGTCTATGTTATAGGA

TAACAAGATGGTTAGAAATCTTTTATTTTTTGAACCTAATCGCTCTTTTGATTTCGG--A

AAAAAACTTTCTTTATCAATATACTGCTTCTTTTACATACGCATCTCCATTCCATAATGG

AGAATGCCAATAGTTAGGATTCATTAAAAAAATATAGAATCCACTCATGGGGGGAGGAGT

CCTTGCCGTATCAGGCACTAATCTATTTTTAACGTCTAATTAGATCGGGAAATTATTCAA

ATTTAAGAACAGAAGCTGGTTGCTTTTTCTTTCCCATAATTAATTGAAGCCATAGGGCCC

TATCCATTTATTCATTCGACCCAACTTTTTTTTGTTCCGTTCCAAGAATTCGAACAGGGT

TTTGTACCGATCCGATAAAAATGAAATAAACTCAGAACTATCCGTTGATACGACATGCTA

TTTTTTCCATTCATTCCCTTTCAGGATCAGTCGCGGTCTTCCAAACTTTACCGATGGTAT

GGACGAATTCCTCGCTTCATCCAAATGTGTAAAAGATTCTAGCCGCACTTAAAAGCCGAG

TACTCTACCGTTGAGTTAGCAACCCGAAGAAAATAAAACATAATTTAAATATTCAAATTA

AAGGGTGTGAAGATACAATCTAAATCAATTATCAATTAAATAATGAGAAAATAAATTAGA

CGAGGTAATCAAACAATTGAGCTAAGAAATCTAAAAAAAAAACAGATTTTCTAATGGATT

AGAAACATAAAAATGAATAGATCAGATCAAAATACAAGAAATTATCAGATAAAAGAAAAA

ATATATATATAGAGAGAGAATTGTCAAAATTGATAGAGTACCTCTTATGTTATCTAC--T

TTTTTTTTTCAATTAAAAAACCTCTTGTATCAATATCAAAGAAAGGAATGAGGTAAAGTA

AAAAAACAAACCTATGGTACGGAAATAAATAGATCCACTTCACTGAATTATATTTGTTCG

ATACACTGTTGTCAATATAAATGTTGAGAAAATAATACAATGGAAAAAAACAAAAGAAAT

TTCATTGAAATCTTTTTTTTTTAAACTCAAAAAATTTCAATTTAACAATGAAATAATATT

CCAAATTGTCTTGGATTGACACTAGATATCTAATCTACATAGATACAAAAAAGTGTAGAT

GGGAGAATAAGAATTG-AAAAAAAAA--ATATTGGATTTTTAATGTATTTCATCAATCTA

AGTCGAATAAACAAACTGGATTCCTTGTTGGTTAGTCAAATTCCAACGAAAACCACATAA

TTGAAAGAAATGAAATGTCCGAATTTTAGATTTATATGATCAATAAACTAAGGTTTTGTT

GTTATCGACCATGGAATTCGACAGAAGCAATGAGAAATAGATACGAATAAAGCAGGATTA

AGGGGGGAAATAAAAAAATAGTAGAGAAAAGTTATACAAAGTTATATAAAAATCACTACC

CCCCCTTGGTATTTCTTTAATTGAATTTCGTTTGATTAGGTCGAAGTTCCTTAAAAACCT

CTGCCTTCTTTAAAATATCCTGAACAGTTCCTGTAGGTTGAGCACCCTTTTCAAGGAAAT

ATAGAATAGCAGGAACATTTAAATAAGTTTGATTCTTCAGTGGATCATAAAAACCCACTT

TTCGAAGATCTTTTCCTTCTCTTCGGGATCGAACATCAATTGCAACGATTCGATAGACGG

CTCATTGGGATAGATGTAGATGAACAATACCCCCCCCTAGAAACGTATAGGAAGTTTTCT

CCTCGTACGGCTCGAGAAAAAATGATTTGATTCTAAGTTTTGTCTATGTATGGAATTCTA

ATAAATGACAAATGAGCCTATAAAATCAATTAGACTATGATTTAAGTCTTTTTTT-TTCT

TCTTCCTTCCTGAAAATGAAAAAGAAACCATTCGTACTCATAACTCAAGTTGGATAACTC

TCAAATAGCTTAAAGGAAAAAATCTTTAATAAATTTCATTTATTGAGTGGTCTTTACCCC

CTTTTGTTTGTCTCGTTTAAAATTCTATTTTGATTCTTCAGTCTGATCCAGTTATTGAGA

CTATCGAGACAATTA-AAAGGGTGTTTCCTTGTTCTGGGATCCTTTATCTTTGTTTTAAA

TCATTGGGTTTAGACATTACTTCGGTGCTTCTTAATCCTTTCAAAATGGCAGCAACATAC

CCTTTTTTGTGATTTCTCTTTCTATCAAAGAATCCTACGGACAGTTGATTCCCGCGTGAT

ACACTTTGGATCGAAAACGTTTGATCAATTCCAACAGGTTTTGCTTTTGAATTGGAAACT

TGCTCAAATTGGATCCTTTCCATTTCTATCTCGAAGATATATTTACGAAGTTGTTCCAAT

TTATTGATTGGCATTAACCCTAGATCCTTGCCCCCGAGAAATGAATTAATCCTTTCCACT

CGAGCTCCATCGTGGACTATTTACAACCCAACAAAAAGAAAAAACGAAGGGTTCGAGTGG

AACAGAACAAACGATGTCGAGCCAAGAGCACCTTCATTCCTATATAAATATAAAATAGCG

GATGTAAAAATCCACAACGGATCTGTCCTTCAAGTCGCACGTTGCTTTCTACCACATCGT

TTCAAACGAAGTTTTACCATAACATTCCTCTAATTTGGAACCGGTATGGAATTGATTCAA

TCATGGAATCATGAATAGTCATTGGTTCAGTTGTACATAGACATCGCGTAATCTATACTT

TTTCTTCTGATATGTGTAAAGATTTTTCTGAAGAAGTCTTAGCAAGACACCATCTTTAAC

ATATATATAAAGAAATAGAAATAGATAAACGAATAAATAAGTTCTTTTTGAGTCTGCTAC

TTCAAGTGACTCAATAGGATAGATTGACCTATTTCCTACTTTTTGAGTGCCAAAAATTCA

ACTTACAAGGAATCATTCCAAATTTTTATTAAAACTATTTCGAATGGAAAAAGGTTCCTA

TTTAGACATCATTAAAAGATAAGTCTTTTTTTTTTTAATTGACCCATCACTGATTTCAAA

TAGATAAATAGATCACAGAAAAGAAGAAAGAAATCCCATTTTTTTTTCATGAGATGGATA

AAAAAACTGATGTAAGGTAAGATTTGAATCTTTATTCTTTTCATCTGATTGCGAAAATCC

AAATCGAAAAAATCGAGAGGGGTTTTCGTATCTATCAGATAGACTGAACAATTGTCACTG

TTATAGATATTCTATAACACTTAATTTAACTAATTTTAGTTTAAAAAATTTAAGGGATCC

CAAACCTTTTTCACAAAAACCGAAAGACTATTGTCATTGAAATAGAACGATACATATAAG

CTAAACATTTCCTCATTTTATATGCATTTTGTTTAACATGGGGTTGAGTAATATTTCAAT

AATCGAATCTTGTCATAAAGTGAATAAAAGGGATAGGATAAATCACCCCTGCCTCAATCC

AATCGTTACATAGATTTACAATTCTTCATTATAGCCAAAC-AAAAAAAATAACTAAATAA

AATAAAAAAACGAGATTCAAAGAAAATACATCGATTCCATAACATATAAACATATATAAA

TATGTTATTTGATCATTTGTTAATGAGATTTGGACAGATACAGATATTTAAATTAATACT

GATTATCTCCTATCCACTCCCCTAT-TCTTTCTATGGGAATGATAGAGC-AAAAAAAAAG

GAATCCTGATCCGGTATGGGAAATGACTTGTCTAGTTACAAAGACAAACAATAAGTCCAA

ATTTAGTCAAGCTTTAGTCTAACCCACTAAGAGACTTAGTCCTATTGATTGAATTTAATT

AGTATCAAGAACTCGCTCTTGTTTCGAATTCAGAGATCTCGAGAAAAATCTAATTACTAA

TTAGACTCCCTCATTTACGGGATAAATAATAAGAGAATATAGATTCTTTTGCATCTCGAT

TCAAAATACATCCATCGTTGAAAATTCAAATAGATATGGGATGGAAAGTTTTTCCAAGTA

ACTAACTTCCCTAAATATCAAAGGGAATGAACATATGATGAAAAGCCCACCCAACTTTTT

TGAATTCAAACTCTTTTGTTTTGATCCATGTTCTCATCTTACCTGATAAAAAATAGATTC

AGGTCCAGATGCGTGTCATTTGAACTCGATTCAGTTCAGTTTGTGAAAAAAGATATGATT

CATATAAGATATAAAAGAATCACATTCCATCTAAAATTAGACTTTAAACAGAAAGTTGTT

CAAGAAAACAATCTTTATAAAAATGGATATGGCTCTGGGACGGAAGGATTCGAACCTCCG

AATAGCGGGACCAAAACCCGTTGCCTTACCACTTGGCCACGCCCCATTATCAATTTCTAA

TCTACACTAAGAAACAATAATATTGTTATTGGTTGTTTGTCAACTCCAGTCCAAATATCT

ATAGAATAGATTATTTAGGATTTTAATCCATATAGATATAGAATTCAACTAAATTTATTG

ATCATTACATATAATTCAATTAAGATATTGTATGAAAGTATGATTTCTTCTATTCTCCTT

TGAGAATTGGAGGATTTTTGATTGGGTGGGTTCAAAGAAAAAGAAGGA-TTTTTGTATAC

CTTACTTCCTTTCTTCCTTTTTCCTTATATCAATAACTCAATCAAAATGCAATTATCTCC

AAGAACAAAATGTCTGTTATGCTTAATATCTTTAGTTTGATCTGTATTTGTCTTAATTCT

GCCCTTTATTCGAGTAGTTTTTTCTTCGGCAAATTGCCCGAAGCCTATGCTTTTTTGAAT

CCAATCGTAGATGTTATGCCAGTCATACCTGTGTTTTTTTTTCTCTTAGCCTTTGTTTGG

CAAGCTGCTGTAAGTTTTCGATGAGATCCTTAATAATATCCTAGAAGATTCATGATTTCT

TCGAGAAAAAATTCTCTAACAATTGATAAAACCAGATAAGTTTTAGAGTCTGAACCCTCG

ATTCACACATTGAAATTCTTGGATAGTCGCCATAAATCCGGCTTACCCCATTTCCTCCTT

TTTTTGACCCTTTTCCAGTGAAAGACCCTAACCCTATTTCTATTAGGGTCTCCCACAATA

TCGAATTTGGATATGAAAGAAATTTTTGTTAATGAAAAATTCTTATTCCAAATAAATTTC

TGACAATTCATTTCTATTTCTAGAAAAACCTCATTTCTTGGTGTCAAAATAGGATATGTG

GTAGAAAAATGGAAGATCTATTCTC-AAAAAAATCATCTTGGAGATTGTGTAATGCTTAC

TCTGAAACTCTTCGTTTATACCGTAGTGATATTTTTTGTTTCTCTCTTCATCTTTGGATT

CCTATCTAATGATCCAGGACGTAATCCTGGACGTGAAGAATAAAATCCAAAGGGTTTTTC

CTTGGTTAATTTTCAAATTTTCTTAGGATTTTTTCTATTCCACACGTTTAACTAAGATTT

CAAAAATTTGAAAAATAAATAAATAAATCAAGTCATCAACGGAACCGGAAAGAGAGGGAT

TCGAACCCTCGGTACGAATAACTCGTACAACGGATTAGCAATCCGACGCTTTAGTCCACT

CAGCCATCTCTCCCAATTGAAAAAGAGAATTACTACATTACACATAATGTAAGGAGTCTT

TCTTT-CTCTATTCTATAGAGATATACAAATCAGGAATTTCTTTTAGATTAGATAAAGGA

AGGGCTCGAACGAGCCTATAAATAAAAAAATAAAAATAAAG-AAAAAAAAAGAAGACATC

TTTGTGTTGATTTTGTTCGAAAGGCCCTTCTTATTCTGATGGCCTGGCCTGGTCAGTACC

TAGCCGGGCCCCTTTTTTTGTTCCAACGAATTATAGATAAATAATGATTTATTTGATTTG

AAAACAAAAATGCTTGTTATTTATTATATTCAATTGAATATAAAATATTCAAGCAACAAC

AAAAAGAAGAAAGACTATTTACATTCTTTTTCTTTTTCTATTTGAATTTTAGTTTCATTT

TCGGAACTAAAAAGGAAACTCTCGATTTTTCCACAATGCATTTTTCTGTTATGATTTTAG

TGTTTTTTGTGATCCGTAGCTATCAAAACTTCTTAGCAAAAGAGAAAACTTTAGTTATTT

AATTTAAATAAAATGAACCCTCCTTTCAGAATCTCATTAAATTGTAAAT-CCCCCCCACG

AAAAATTTCAACACTCTAATTTTGATGATTCTTTGATGATCCAATCCTATCTTGATCATG

CCCAATTCTCCTGTTCGACAAAAGGTCCATTTGTATACAATAATCGCATTGTAGCGGGTA

TAGTTTAGTGGTAAAAGTGTGATTCGTTCTATTACCCCTTTAATAGTTAAAGGGTCTTTC

GGTCTTATTCATATTCCGATCAAAAACTTTATTTCTTAAAAGGATTTAATCCTTTACCTC

TCAATGAAAAATTCGAGAAAAAAATAAAAATTCTCGTGATTTCTATCCATGAGTCACTTA

GAAAGTGAAAAGTTGGATTATGAAATTGCGAAACATAATTTTTGAATTGGATCAAGACTT

CCAATTGAATAAGTATGAGTAAAGGATCCATGGCTGAAGATAGAAAGTCGATTTCTAATC

GTAACTAAATCTTCCATTTTTGATTTGTAGAGAAAGAAATTGAAGCAAAATAGCTATTAA

ACGATGACTTTGGTTTACTAGAGACATCGACATATTGTTTTAGCTCGGTGGAAACAAAAT

CCTTTTCCTTAAGATCCTCTCAAATAGAAATAGAGAACGAAGTAACTAGAAAGATTGTTA

TAATCACCTTCTTCTAGAAGGATCATCTAGAAAGCGATTCGTTTTGTCTGTATTCAGACA

AAAAGCTGACATAGGTGTTATGGGTAGAATTTTGTTCACATCTTAGATCTAGGAATTTAC

TCATCTTCCATAAAGGAGCCGAATGAAACCAAAGTTTCATGTTCGGTTTTGAATTAGAGA

CGTTCAAAATGCTGAATCGACGTCGACTATAACCCCTAGCCTTCCAAGCTAACGATGCGG

GTTCGATTCCCGCTACCCGCTTATATCCTTTTTCTAAATATTCGATTCGATATATTCTAG

AATATACTCTAATTAGAATTAGTGAATCATTGAATATACAATTCCAAAAAATTTCTCACA

TACAATCCGAT-TTTTTTTAACAAGAGGTAGAAAAGTCAAAATACG-AAAAAATCGGAAT

GAACAGCGTCCATTGTCTAATGGATAGGACAGAGGTCTTCTAAACCTTTGGTATAGGTTC

AAATCCTATTGGACGCAATTTATTTCCATATATTTTTGATTTCGATAGCAAGAAATACTT

T-TTACATAATTTGAATCCGAGACACTTGATTCCTTTTTTTTTTTAAGATAAAAGTGAGC

AATTTCTTTATGTTATGCTTGTTCCTGAAGTAGGAATCGGTCCATTTGTTCCTGAATAGC

TTCTTTCAAAAGGACTTCTGCTTCCTCGGTAAATGTCTTAGTAGAAGATATGATTTCTTG

GAACTGAGGTTTATTAGTTTTTAAGTAAGTACGTAACTCAACAAGAAATTTCCTTACCTG

TCCAATTTCTAATGAATCAAGATAACCATTTGTTCCGGTATAAATAGTCATTATCTGTTC

TTCTACCGTGAGAGGAGCTGCTTGGGATTGTTTAAGCAATTCACGTAATCGTTGACCTCT

TGCCAATTGATTCTGAGTAGCTTTATCGAGATCCGAAGCAAATTGTGCAAAGGCTTCTAA

TTCTGCGAATTGCGCCAGTTCCAATTTTAATTTACCAGCTACTTGTTTCATGGCTTTAAT

TTGAGCTGCAGACCCTACTCTGGAAACGGAGATACCCACATTAATAGCAGGTCTGATTCC

AGCATTGAATAGATCGGCGGATAAGAATATTTGTCCATCAGTAATGGAAATTACATTAGT

AGGAATATAAGCCGAAACATCTCCCGATTGAGTTTCAACTATTGGTAAGGCGGTCATACT

TCCTTCACCTAAACTAGAACTTGATTTAGCGGCTCTTTCCAAAAGGCGTGAATGCAAATA

AAAAACATCCCCTGGATAAGCTTCACGACCAGGCGGTCTTCGTAATAGAAGAGACATTTG

GCGATAAGCTTGGGCTTGTTTGGAGAGATCATCATAAATGATTAAAGTGTGTTGTTTACG

GTACATAAAATATTCAGCCAAAGCTGCTCCTGTATAAGGAGCAAGGTATTGTAATGTAGC

AGGGGAATCCGCCGTTTCGGCTACCACAATAGTGTATTCCATCGCCCCCCTTTCCTGTAA

AGTAGTTACTACCTGAGCCACAGAAGATGCTTTTTGACCAATAGCTACATAAACACATAT

TACATTTTGACCTTGTTGATTGAGAATCGTATCTGTGGCTACTGCTGTTTTACCGGTCTG

TCTGTCCCCAATAATTAATTCTCGCTGACCACGTCCTATAGGGATCATCGAATCAATAGC

AATAAGCCCGGTTTGAAGAGGCTCATATACGGAACGCCGCGAAATAATACCTGGAGCGGG

AGATTCAATTAATCGAGATTCCGAAGCTGAAATTTCACCTCTACCATCAATAGGTTTAGC

CAGGGCATTTATAACACGACCCAAATAGGCCTCGCTCACTGGTATCTGAGCAATTCTTCC

TGTTGCTTTTACAGAACTTCCTTCTTGTATCATTAAACCATCACCCATTAATACAACACC

AACATTATTTGATTCCAAATTCAAAGCAATACCTATTGTACCTTCTTCAAATTCTACTAA

TTCACCCGCCATTACTTCATCAAGACCATGAATACGAGCAATGCCATCGCCTACTTGAAG

TACGGTACCAGTATTTACAATCTTTACTTCTCTATTATATTGTTCAATACGTTCACGGAT

AATATTACTAATTTCGTCGGCTCGAATGGTTACCATGAGTATTTCTTAATTATTTTTTAT

TTTTAAAAGAAAAAAAAATAATGCCTATAGTAGAAAGACTAATCAGTTATTTCTTTCATC

GCTCCCAACATGCCAATATTAGCACTAATGGTACGTAAATGTAACTCGTTGTTTAAACAA

CTATTCAGAGTTCCTAAAGCTCCTTGTAAGGCTTGTTGGAAAACCCGTTGTCGGACTTGA

TTAATCGCCCTTTGCTGTTCAAACTGAATCGTTTCATTTTTGTAATTTTCTAATTGTTCC

AAAGTCTTAGAAGTTGAATTAATCAAATTCCATTTTTCTCGCTCTATCTCAGAGTATCCA

TTCACCCGAAACTGATCTGCTTCCATTTCCACTTTCCGTAAGCGAGCCCGGGCTTTTTCC

AGCTGTTCAATGGCCCCCCCACGCAGTTCTTCTGAATTTCGAATAGTATTCAAGATCCTC

TGTTTTCGATTATCTAATAAATCACTTAATGAAAGTAGATTATCTTTCCATTCATTTCAA

AACTTCCATAATCCCTTCCCGAACCAAACATGAATCTTTCGATTCATTTGGCTCTCACGC

TCAATTACTTAGGGTAAATTCTCATA-TTTTTTTTTATGAATGTAATGAGCCTATCCTCT

CTTCTTTATTCATATT-AAAAAAAACTTAATCTAATGCAAAACCAAAATACTCGGAGGAC

TCTTCTGACAAAATAAAAAATATGTAATTGTCAGCAAAGTTGTTTCTTTTTTTTCTTCAC

TTCAAATCCAAAAAATTCTTCTGACTTATACATAAGACATAGGTCGTCGATTCAGCATTG

GATAAAAGAGGGAAAATGCCCATTTTAGAATAAGTGGTTCAAATCATTTTATCGAGATGA

GTGTTCTATATCGATAAAATATTCATTTGAAAACCATCACTATATTAACATAGTGGTAGA

AAGAGTACCATGCCGCATCTAGACTTCAAACGGTTTGCTTTAACCATCTTAACAGTCCCA

CATTATTGGTTCCTAGAGAATCAAAGTAGATTTGCCAATTAATCACGAAATGCTATGGTT

CTTACATATAATTTATTAATTTCTTCAGAAGTAATTCGCGAGATCATGCACCTCTCTTTC

CTAGTTATAAAGGAAAAAGGTCCAGCTGGTTGGATCCAGCCTATTCTTGAAATAAACAAC

TCACACACACTCCCTTTCCAAAAAAGATCAATACACCAATCACTACACTTAGATTTATTG

GATTTGTTGCTAAAATATCGGTATTAAATCCGAAACTCCCGGCAGATGGCCAGTGGCCCA

AAGAAACGAAAGAATCGGTTACATTTTTCATAGGATCTCCTCTTATAGATAGACTCAAAA

ATAGATCAGAGTTCTTTTTGTATCACTTTGCCTATTTATTCTTTTCTATCGAGTCAAAAA

ATATTCGAGTTATAAAGTATGAACTACCCCCTTGCTGTTGCAACACCTCCT--AAAAAAA

AAGAGTTTCCCTTGGACTACGAACGGGAAAGGATGAAAGCGAGTCGGTATGCTAATTCCT

CATCTGCAAATCAGCCCTTCCCGTAGGGTATTTTCTCAACGAATAAGTAATTGTAGGAGT

TAAATCTTGATATAATTTGAAAAAGCAAACAAGAGGTCCAAGGCAATAAAATATGAAAAA

TATGTATTTTTCATATTTCTAAGATTAAACAAAAGGATTTGCAAATAAAAGTGCTAATGC

TACAACCAATCCATAAATTGTTAAAGCTTCCATAAAAGCTAGACTAAGCAATAGAGTACC

TCGTATTTTTCCCTCTGCCTCGGGCTGTCTCGCGATACCCTCTACAGCTTGACCCGCAGC

AGTCCCTTGACCAACTCCAGGTCCAATAGAAGCAAGCCCTACGGCCAATCCAGCAGCAAT

AACAGAAGCGGCAGAAATCAGTGGATTCATGATAAGTTCCTCGTACCAAAAAAAAAGAAA

TGGTTAATGATACAATCAACCAATGAATTATGACTTAATTATTCCATCGGTAGGATTCAT

CCAGTCGAAGTAACTAACAACTTCGAATTGAAGTAATAATATTATTGAATCATCAGAACT

ATTTCGATATCTCTATTTTTCGTTTCTATCCACAGAATCTTTGTGAATCCATA-GACTTT

CGTCCTTGCATTTCTTGGTTCCGAACTGTTATTTCAATTCTTCCATCTTTTTCTTGATTT

CATCCCCTTATTCAGTCAATTCACAGTCACAACGAGATGGAAGGACTTCTATTGGAATCC

ACATACAGTAGTAGAGAAAATGAAATATATCTTTAGTTCATATATAACTAGTCAATATCT

AATATCACATATACATGTCTTTCTTCCCTAACGTAAACCAAGCATTCATCTTAGATTCAA

TCGAATTCGAGAATCAATCGTCGAAACATCTACAAGAGTTGGCTTAGAACCATTCAAATA

CATATATCTAGTTCGACTCCCCTCTTCGAATCAATAATTAATCCATTTTTCAGGAATCCC

GTTTTTTGTAAATTATTTTCTCCCTTCCACTTTCTTTATCATTATTCCACCCGAATACAA

TCTAAATGAGCAAATTAATTAGGGCGAATTATTGCATATGCATAGAAATATCATTATTTG

ATTGATCTAAGTTCATGCAATTTATTATTTATTAATATTTTCTTTTGGTCCATTGTTGAA

TAAAATCAACTGAAAGTAGAATAGTTTCTCGTGTTTTTTATTTAATTACACGTGGTAAAC

ATATATCTTATTCCAATTTTGATTTGAATAACAAGATTGGCTGACCAATATAAAGCGAAT

ATTCGTTGAGGGAAGGTATGATAGTAAGTGGACGAAAAGGGAATTTTAGGAAAAAGATCT

AGATCTCTTTCCCCTTT-TTTGTTCCTATTACTTTCTATTTTCTATCGTATATAGCGTCG

TTGTATATTTCTATTCCTTAAGTCAATTATCTTGAGCCACACATACATTGCTTTGCATTA

AGCT--AAAAAAAGACTATTTCAATGATGGCCCTCCATAGATTCACCTATATAAGCCGCG

GCTAAAGTTGCAAAAATAAGAGCTTGAATACCACTTGTAAATAATCCAAGGAACATGACA

GGGATAGGAACCACTGAAGGTACTAAAGAAACAAGAACAACAACTACTAATTCATCCGCT

AAGATATTCCCGAAAAGTCGAAAACTAAGTGATAAGGGCTTTGTGAAATCTTCTAAGATG

TTAATGGGTAAAAGGATTGGAGTTGGTTGAATATATTTCCCGAAATAACTTAATCCTTTT

TTGGTAAGACCCGCATAGAAATATGCCACTGACGTGAGTAAAGCCAAAGCAACGGTAGTA

TTTATATCATTCGTGGGTGCGGCTAACTCTCCATGAGGTAATTGTATGATTTTCCAAGGT

AAAAGAGCTCCTGACCAATTAGAAACAAAAATAAATAGAAACATAGTTCCAATAAAAGGA

ACCCAAGGGCCATATTCTTCTCCAATTTGAGTTTTACTCACATCTCGAATGAATTCAAGA

ACATATTCGAAGAAATTCTGACCCCCAGTCGGAATGGTTTGTGGGTTCCGAACAGCTATA

GTAGCTGAACCTAATAAGATAGCAATTACAACCCAAGAAGTAATAAGTACTTGGCCATGG

ACTTGGAAACCCCCTATTTGCCAATAGAAATGTTGGCCTACTTCCACAGCGGATATATCG

TATAACCCTTTTAGTGTGTTGATGGAACATGATAACACATTCATATTGCCCTCTG-AAAA

AAATCGAACTTTAAACAAAATTATTTTGATTCAACCATCTCTTTGCCTACTTGAATCGGA

TATTTGAAATACCAACTAATATTTGGAATACTAACTAATCACATAATATCCCCAGTTATT

TTTATCTCTTTTTAATTCAGAAATAATAACCGATTCCATAAATCTATCGGATTTTTGAAG

TAAATTATTTATCTTATTATTAATCAAGGATTTCTTATATAGCTAGAACGACCCTCACAA

ATTGCGAATACTAATTTGTTAAGAATTAAGCGGATTGAAGATATAGCGTCATCATTCGCT

GGAATCGAAATATCTGCAAGATCAGGGTCACAATTTGTATCGATTAAACAAATTGTTGGA

ATTCCCAAAGTGATACACTCTCGCAGGGCCGTATATTCTTCGTGCTGATCAACGATAATT

ACAATATCGGGTAACCCTGTCATATATTTAATCCCGCCCAGATATGTTTGCAAGCGAGAT

AATTGCCTTTTCAACATAGCTGCATCCCTTTTCGGAAGACGGTTGAGTCTTCCTGTTTTT

TGTTCCATTCTCAAGTCCCTGAACTTATGAAGTCTCGTTTCTGTAGTGGACCAATTCGTT

AACATACCGCCGAGCCATTTTTTATTAACATAATGACACCGGGCCTTTATCGCAGCCCAT

GCTACTGAATTAGCTGCTTTATTTTTGGTACCAACAATTAAGAATTGTTTTCCCCTACTT

GCTGCATCAAAAACCAAATCACAAGCTTCGGATAAGAAACGAGCAGTTCTAGTAAGATTT

GTAATATGAATACCTTTACGCTTTGCAGAGATATAAGGTGCCATTTTAGGATTCCATTTC

CTAGTACCATGGCCAAAATGAACTCCTGCCTCCATCATCTCTTCCAAATTGATGTTCCAA

TATCTTCTTGTCATTT--CCCCCCCCAAAAAAAAAAGAAAGGAGGAACTCTGAACTGAAA

TAAATAATTGTTCCGATGGAACCTTCTCTTCTACCGTAGATTGGCCGTAGATAGACGAAC

AAGCCATTACTTTTCTATTCATTATTATTTTGATTACCAAATCAAATGACTGCACCAAAT

ACAGATAGTCAAAAAGATGAATCTGCTCTTAGGAATCATTAAATCCTATAAATGATTGTT

CTGATGTATCATGGAAATTCTTTGAAAGACAAGAATCAAATAATTTTCTGTGGTGAAACA

AAATATCTCTCATTTCCCCCTCGAATAGATTGTTTTTTTTTGTTTCCAAAAGACTGTTGT

TATCTTGTTTTGAGGGGGGCACTAATCCTTTAAATCCGGTACCAAGGGGTATCATACCCC

CCAAAACAACGTTCTCTTTCAGGCCTTTCAACCAATCGATCCGACCCCGGAGAGCTGCTT

TTGCTAAAACTCGAGCAGTTTCTTGAAAACTCGCTTCAGATATGAAACTTTGAGTATTGA

GAGATGCTCTTGTTATTCCCAATAAGACGGCTCGGTAACAGACCGCTTCTTCCAAAGCGC

GCCCCATTCGTTCCGCTCGCAATAATCCAATTAATTCTCCGGGTGAAAAAACATTAGACA

TTCCATCTTCTGAAACCAACACTTTTGATGTTATTTGGCGTACAATAATTTCTATATGCC

TATTATGAATCTGCACCCCCTGGGATCGATAAACCTTTTGGATCTTATTAACCAAAGAGA

TACGACTTTGCACTATAGTTAGCTCAGCACCAATCAAGAAGCCCCACGGCATTCCAAGAA

TTCTTGTTATACGTTCGTTCCAACCCTCAACCCTCTTTTCTAGATTCATCGATATTGAAT

CAATCGAACGCACTTCTAACACCTGTTCCACTTTTGGAAGCCCCTGGGTTATATCACCAG

ATCTCGATTTTTCATATATAAATGTAATTAATGTATCTCCTTCGTACAGGATTTCCCCAT

AATGGCCATGAACAGTTGCTCCTGGAGTGGCCAAATAAGGCTTAGCTGATCGTATTACTA

CAGAATCAACTTGAACAAGTATAACTTGACCCGATTTTAGGTGTGGTGCATTTTTGGCTA

TACATACATTTTCACAAATAAACTGTCCAAGACTAATTATTGTAGATGTTTCTTGACAAT

AATTGTGATGGAGAAAATACCAATTCAAATTGAATGGATTCAAAATAATGTTACTGCCTG

GCTCGGGGTTATAAATTTTATCATTTTCATCCATTAAATAATATTTAATTACTTGAAAAG

TCTGTTTTAAATTGTCAAGTTGCAAATAGTTATTTACCAAGATCTGATTATGAGTTATTA

AATGGTAAAATGAATAAAAATTCACAATTGTAAAGGCTGTTCCAAAAGGCCCCAGCGAAT

TCTTAATTGGAATTCGAGGATCCTTTGTAATTTGAATTGATTCTTTTATCACATTGTGAT

ATTTTATTACATCGTTGAATGGACCCATTCGAAAACAATTGGATGATGACAAAATTATCA

ACGATTGGAATCCCGTATTTCTATTCAACAACGTATGAATAGTTCCTTTATTTTGGTTAA

GGGATTGTTGAATTCTTGCCTTGGAATAAATGGAAGAAAACGGATTGATATTGGTGCAAT

CTGATCCATTATCAGAGAGCAATCCTGAACCCGATGGATCATTCCTTTTTCTGATATATG

AAATAGGGGATTTCACTAAGTCGATTCTTAGGAAATGTCGAATCAAACCATTTGTCCTTA

TTTCAACAAAGGAAGCACGGGCTTCTTCACTAGAAGAACTTTTTTTTTTGTCTTGGTCCC

AATTCAATACTAAACAAGTCCGAACTAATTGAATATCTGTATCAGAAATTCCTCGAATGG

GTTTGCCATTTCCATAAAGGATATAATTGACAACTCGAAGTTGCACATTATCCCTTTCCT

GCAACAGATCCGGGGGAAAAAGTGTTGCTAAATTTATACCGTCCGTTATTTCATATGTGA

CGACAAGTCGAACCAAAACAAAATACTTTTTCTTACTAGGTGTGATCCGTTGAACATAGA

TCCAATTTTTCCATTTTTTTGATTCCTTGGAATTTCGTTTTCCTGTTCCTGGTGGTATCA

AAACGCCGCTATGTCGAGATATCTTATCGGTCTCTCCAGGAAAATGGATATCTCCAGAAA

AGATTTTAAGTTCTATTCTTTTTTTTTTTCTCTCCACTCGGACAAACCCGCCTACTCGGC

TTCTTATATTTAAAGTAATTTGTGTATCTACCCCAATGAGACTATTGTTCCGTACCATTA

TGGAAGAAGATCCGGGTAAGATATGCACTTCCTCGGGAATAAAAAAAAACCGATCTACTT

TCATTTGGTATTTTGGCCTAAATGCCTTGACTCCTCGATACTCAATCAAATCCTCTTTTT

TGACGATTGAATGCATTTCTACAGTCCCATATTTAGTAATGCCCGAACTCTTTCTTCTGT

ATCGAGGATCATCAAAATAAGCAAGAATACTATTTCTACGGAAAATGCCATTTGGGGGGA

TTTCAATCGAGATACCTGAACAGGGCATTAGTTCGTTCTCACGTTCTTGAATCGATTGGA

GTGGAATGATGAATCTATTTCTCCGCCTCTTTGAGAATAAATCAGAATTCTGGTGGAGAA

TGGTCGGATATATGAGATTACAACGACCATTACATCTAATTCGGTTAAGGTCCGAATAAT

CAGAAATCCTATCTTCTTTTTTTTTTTTACCATAAAAATACGAACTAAAGACTTTTTGTC

TCTCCGGATCATTAGTTTCTGAGAGGTTAGAAGTATATCTTCGCTTGACAGAACGAGAAT

GCGCGTTTATTTGATCTTGATCCTTGTGGAGCGAAAGTGAGACTAGACTGGATCTGCACG

GCCCTCCTAATAATATCCATAAATGACTTGTTTTTGGTAATATATGAACATTACCATATG

TAAATTCAGGTGGATGATACACATCAGTACTCCAGTGCATTTCTCCGTCTGAGTCAGAAT

AAATATGTTTTCGAACCTTCTCTTTAAAATTCAAAGTGGATGTTCCTGCGCGAATCTCAG

CAATCACTTGTTCTGATTCTACATATTGATCGTTTTGAACTAAAAGAAAACTTTTGGATG

GAATATTTACATTATGTAGAATATCTTCACTCTCAATAGTTACATACAAGTCTATAGAAC

ATAGAAAGGCGGGATGTCCATGACGTGTACGTGTCGGATGAACCAAATCCTCATTGAATT

TGATTTTTCCATTAGAAGGGGCCCGCACATGTTCTGCAGTACCCCCCGTGAATACTCCGC

CGGTATGAAAAGTTCTTAATGTTAATTGAGTACCCGGTTCTCCAATCGATTGTCCTGCAA

TAATACCTACAGCTTCTCCTAATTCAACCAGGTCGCCATGAGTAGGACTCCGACCATAAC

ATAATCGACAGATCCAAGACGCACTCCTACAAGTAAAGGGAGTTCGAATAGATATTGGTT

GTGCTCGAAAGGTTATGAATCGATTTACAAGTCCAATCCCAATGTCTTGATTTCTACTGG

CAATACATCGTGTGCCCGTATATATATCATCTGCTAATACACGACCCATTAATGTTTGGA

TAAAAATCCTTTCCGGCATCATCCCATTCCGAGGACTCACAGAAATACCCCGGATGGTGC

CACAATCTGTCCGACGTACAACAATGTGTTGAACTACTTCAACAAGTCTGCGCGTGAGAT

ATCCAGCATCGGATGTTCGTACAGCAGTATCCACAACCCCTTTACGGGCTCCGTAGCAAG

AAATGATATATTCTGTTAAAGAGAGTCCTTCGCGTAAATTGCTTTGAATGGGTAAATCAA

TCATTTGTCCTTGAGGGTCCGACATTAAGCCTCTCATACCTACTAATTGATGTACCTGAG

ATGCATTTCCTCTAGCTCCCGAAAAAGACATTATATGAACTGGATTAAAGGGGTCAGTCA

TCCTAAAATTAGGATTCATTTCTTGTCGCAAATATTCACTTGTAGCATACCATATTTCAA

TGGATTGGCGTAATTTTTCTACCGCGTGTACATTCCCATAATGATGGTGTTTTTCCAAAA

TCAAACTTTGTTGTTCAGCATCTTGAACTAGCCATCGCTTAGAAGGTATTGTTAAAAGAT

CATCAATTCCTAATGAAATGGATGTAGCAGTAGCTTGGTGGAAACCCAGAGTCTTTACTT

GATCCAGGATGTGTGATGTATATGCCATTCCGAAGTGATCTATTAATCTACTAATAAGTC

GTTTCATGGCAGTTCCATCTATCACTTTATTGTGAAATACCAGATTGGCCCGTTCTGCCA

TAAGTACCTCCATATTCTGCTGAGTACGATTCGACAATGGGTTTGAGTCAGTGATTGGAA

AACTTCCTTTTCTCGATCTTGATTCGCATAGAAATTCCGGAACTATGGGCCTAGTTGAAC

TGAAGAAACCCGAATTCCTACTGGTATTATAGAATTCCTTAACTTAGTTCGGTACCATAT

GAACAGGCCTGAGAAAACCCCTGTACGGCTTCTTCGATTTCTCGATAAAGAGAAATATGA

CCAACAGTGGTTCGGATGTATATAAAAAGAATTTCTTTTTTTATACTTCTTACTATTAGA

TAGTGTCCATAAATCTCATAATAAGTACCTAAAGATTCATAGTGAACTTCGATGGGACTT

TCTCTTGAAGCAATAACGCGTTGATCTAGTCGCCACCGGAGCCACAAAGGACTATCTAAA

TTGATTCGTTTCTGCCGATAAGCTCCAATTGCATCATAGGAATTAGAAAAAAAGGGTTCT

TTCGTATACTTATAGTTATTATTGTTACTTCTTTGATTTTGATAGTTTCTGCGATTCCAC

GGATTATACCTATTTACACAAATACCTCGACGATTCCCGCTCGTTAATACATAGAGTCCA

ATAAGCATATCTTGAGTTGGTATGGAAATGGGATCCCCAATAGCTGGAGACAAAAGATTC

ATATGAGAAAACATAAGTAAACGGGCCTCCGCTTGAGCCTCCAAAGATAAAGGTACATGA

ACAGCCATTTGATCCCCATCAAAATCTGCATTGAATCCCTTACAAACTAATGGATGTAAA

CAAATAGCACGCCCCTCCACTAAAACGGGCTGGAATGCCTGTATGCCTAATCTATGCAGA

GTGGGTGCTCTATTCAGCAATACAGGATGCCCCTGCATAACTTCCTGAAGTATTTCCCAT

ACAATCGGTTCTTTTTCCCGAATTTTACTCTTAGCAACTCCTATATTCGAAGCAAGATGT

TGTCTAATTAGACCACGAATTACAAATGTCTGGAAAAGCTCTATTGCTATTTCGCGCGGC

AATCCACATCGATGTAATGAAAGTGAAGGACCCACGACAATGACGGAACGACCTGAATAA

TCAACCCGTTTCCCCAGCAGAGTCTCACGAAATCTTCCCTCTTTGCCTTCAATTACATCT

GAAAATGACTTGTAAACTTTATTATGACCGTCCCTCATTGGTTGTCCACGGATTCCATTA

TCAAGAAGTGTATCCACGGCTTCTTGTACTAATTTCTCCTGACACATTACTAATTCTCCT

GGCGTGGATTTACTTGTTGTTAATAGATCGGTAAGAGTATTGTTCCGATAGATAACTCTT

CTATAGAGTTCATTAATATCCGAGCTCATTAGTTTACCCCCATCTATCTGAATGATGGGT

CTCAACTCAGGAGGAAGAACTGGTAATAAACACAAAACCATCCATTCTGGTTCTATATTT

GTTCGAATAAAATGCTTAGCCAATTCCATGCGTCTAACTAAAAAATCCTTTCTTCTTCCA

ACCTTTCGATCTTCCCATTCATTTCCTGTGGGTCCTTCTTCCCCTAATTCTTTCCATTCT

ACCAACGAATTATCTAGAATAATTCGTAAATCTAGATCGGCTAATTGTTCTCGGATAGCA

CCTGCACCAGTAGAGATTTCTCGATTGCGAAATGTATCGAAGCCTTGGGTAGTAAAAAAC

AGCGGGATGCTGTATTTCCAAGATTGAATTTCATATTCGAATAAACCTCGTAATCGTAAG

AAAGTGGGTTTTTTAGTTATGGGCCTAGCAAAAGAAAAATTGGGATAGGATCCTATAGGA

TCTCCCCCCTTCAAAATCGGACGTGAAAGTTTCCTTTCATCCGGCTCAAGTAGGTACACC

AAATAAAGAAAGGAGTTCTCGCTTGCAAATTCTAGAAAACCCCAAAACAAAAAGATCTAC

TCCTTACTCAAGTTCCCAGTGAAAACCAAGCAAGATTTCATTGATTCCGCCTTCTTTTTA

TTTAGATTTTCTTAATTCGTTATTCAATTACGACATAAATGAAATGTGAAATTCTTGAGT

AGTCTACTTCCCTTCGAATGATGAATCCCTTAATTCTTAATTTAAGAA-TTAAAGTGAAA

GTAAAGGAGTACCTTGGAATTCATAAGGGATTTACTTGTCTATGTATTGTTCCATTCGAT

CTTTTAGGTCCCGACTTCACCTCGACGGTTATGCTACGATGCCCTTAAAGCCTATACGCG

ATGGATAGACTCCTGTAACCATGACATATTTGCGCTTACTTGAACATAATTTCTTTCCAA

AAGAAAGGTTAATTCCACAAAACAAAAAAGTTTTTTTACGAGGTACAAATATTTGTATTT

ATTTGTTACGAAATCGACCATAGATCAATTCCCCTTTTGATTTGGGAGTATTGACTACAC

CCCTAATTCTGAGCTTCATGTTACTCCTGCCAAGTGACATGTCAGGTCCAGGGCATCCCA

ATTGGATTGACTGGGATGACAGTTTATCATTCCGAATCTGTAAAATCATAATTTGGATCA

AATCACACATCGCAGTATACTAGGCCTTCTAATTCTTTAAGAGGTTTATCTAAAAGATTC

GCAATATAACTAGGAAGACGTTTTAAATACCACACATGGGTTACTGGGCATGCGAGTTTG

ATGTAGCCCATTTGATATCTTCGTATGCGAGAATCGACAAATTCGACTCCGCATTGTTCA

CAAAATTTCGGGTCTTCTTTTTCATCTCCGATTACTCGATAATTTCCACAAGCACAAATT

CCACTTTTTATAGGCCCAAAAATTCTTTCACAAAATAATCCATCTTTTTCAGGTTTATTG

GTTTTGTAATGAAAAGTATAGGGTTTTGTCACCTCTCCAACGATCTCTCCATTAGGCAGG

ATTTTAGTGGCCCAAGCACTTATTTGTTGAGGAGAAACTAATCCAATTCGTAGCTGTTGA

TGTTTATACCGATCGATCATAGAAGAAAAATTCTGATTCATTCCGATTAAGCTTCCTTCC

TATTAATCTGGAAGTTTTTCTCAGATACAAGGAAATGGTTCAGTTCCAGAGCTAAAGATC

GTAATTCTCGAACGAGCAATCGAAAAGATTCTGGAGCATCCTCAGGATTAGGTATTGTTC

CTCCAATGATCGTAGTACCAAGTACTTCTTGGCGCGCTCTAATATGATCAGATTTATAAG

TAAGCATCTCTTGTAAAATATGAGCAACACCAAATCCTTCTAGAGCCCAAACCTCCATTT

CTCCTACTCGTTGCCCCCCTTGTTTTGCCCTTCCTCTAAGGGGTTGTTGTGTAACAAGCG

CATAATGTCCACTGGAACGTCCATGGATTTTATCATCAACTTGATGAATTAATTTCAAGA

TATAAGACTTTCCTATTAGAACGGGTTGTTCAAAAGGATTCCCCGACCTTCCATCAAATA

TTCTGCTTTTTCCTGGATACTCGGGTTCAAATACCCATGGATTCGCTGTTTGCTTACTGG

CTTCATATAATTCAGAAAACACTAGTTTTCTCGAAGCTTCTTGTTCATATCTCTCATCAA

AAGGCGCTATTCGATAATGTCTGTCTAGCAGACCCCCTGCTAACCCGAGCGAACATTCAA

ATATCTGTCCTACATTCATTCGTGAAGGTACTCCTAATGGGTTGAAAACCATATCAACAG

GTCTTCCATCTTGCAGATAAGGCATATCTTGTCTAGGCAAAATTTTGGAAATGATACCTT

TATTTCCGTGTCTTCCAGCTACTTTGTCGCCTACTTTAATTTCACGTTTCTGTGAAATAT

ATATACGAATCGTTTCTGGATTATAACTAGAACCCCCCCTTTTCTGGATCCACCTTACAT

CAATAACCCGGCCCCTACCACCTATAGGTAGTTTTAAACAAGTTTCTTTTGAAGTAGATA

CCTGAATGCCAAGTATGGCTCGTAACAATCTATCTTCGGGGGCATACGACGATTCTTTCA

CCATTTGGGGCGTTAATTTACCTACTAAAATATCACCCGTCTCTACCCAAGATCCCAGCA

TCACAATTCCATTTTTGTCTAAATTGCGGAGTAAATGGGCTTCTAAATGTGGTATTTCGT

TAGTGATCCTTTCAGGGCCTTGGCTTGTCACATGAATCTGAATCTCATATTTCCGTATAT

GAAAAGAAGTATAAATATCTTCATATACCAAACGCTCACTAATGAGTACTGCATCTTCAG

AATTGTAACCTTCCCATGGCATATAAGCTACTAATACGTTTTTCCCCAAAGCAAGTTCGC

CACCAACCGTAGCAGCACCATCTGCTAAAATTTGTCCCTTTTTAATACATTTACCCCGCT

GAACCTGGGGTTTTTGATGCATACAAGTATTTTTGTTGGAACGTTGATACATAACTAATG

GAATGCTTAGAGTATCTCCATTACCTGAGAAAAGGATCTTTTCAGTATCGGTATAAATGA

TCTTTCCCTCACGTTCGGCTATAGCAAGAGCCCCTGAATCTAGAGCTGCTTGTCGTTCCA

ACCCAGTTCCAACAATGCATTTCTCGGGCCGAGAAAGCGGAACTGCTTGACGTTGCATAT

TAGAACTCATTAAAGCCCTATTCGCATCATTATGTTCGATAAAAGGAATGAGGGAAGCTC

CAATAGAAAAATATTGGAAGGGAAAAATACTTCGAAGATGAACCTGTTCCCATGCAATAG

TCAGGAATTCTTGACGGTATCGAGCTGGAACAACCTGTTCTTCCTGAATATCCTGATTCA

ACGCCAAAGAATTTCCTGCCGCTACCATATAGTATTCATCTCTACCTGGTGATAAATAAA

GCATCCGTACCCCTGTTGATCTTTCAGAAATTTCATAAAATGGGCTTTCTAGAGATCCCC

AATGACCAATCCTCGCATGAATTGCTAAGGATCCAATAAGTCCAACATTGATTCCCTCAG

ATGTGTCAATTGGGCAAATACGCCCATAGTGACTAGGATGGATATCTCGTATCCGAAAAC

TAGCAGTTCGCCCTGTTAGTCCTCCAGGGCCTAAATAACTCAATTTTCTCCCATGAACTA

TTTGTGTCAATGGATTAGTTCGATCCAAAACTTGAGATAAGGGGTGTAAACCGAAAAAAG

ATTCATAAGTGGTTGTTAATGGAGTTGAAGTTACTAAATTCTGAGGAGTCGGTATCAATT

TGTGCCGAATTGCTCCACATATAGTTCCTCGAACCACATTTTCTAAACGAACCAGAGCCA

ATCCGAATTGATCTTGTAAAAGATCCGCTACAGAACGAATACGTTTATTTTTCAAATGAT

TCATATCGTCAAGTGTACCCATTCCAAATTTCAGTTCAATCAAATGATCAGCGGCTGCCA

ATATGTCTCGTGGTAACAAAAATGTATTGTTCTGGGGTATATCAAGGTTCAGTCTTCGGT

TCATATTTCGTCGACCAATCCTTCCTAATTCACATCTTTGTTGAAAGAACTTCTTTTGTA

ATTCCTTACATAAGGATTCAGAAAATACCGGATCCCCACCTACACAAGCAAATTGTTGAT

AAAACTCCAAAATGGCATTTTCTTTTGATCCAATTTTTTTTCTCTCCTTATCATTCAGAA

AAGACAAAAAAATTTCAGGATAACAAACATTGTCTAGAATTTCTCTTAGATTCGAACCCA

TAGCTGATGATAGAACTAGAATAGATATTTTTTGTTTCCTACTCACACGAGCCCATATCC

TTGCTTTTCTATCAATCTCTAATTCTGATCTTCCTCCCCAATCTGATATTATGGTGCCGG

TATAGACCGAAATTCCGTTATGGTCCAATTCTGATCGGTAATAAATACCGGGGCTTTGCA

ATATTTGATTGATCACAATTCTATATATTCCATTTACTATAGAAGTTCCCAGGGAATTCA

TTAGAGGAATGTTTCCAATAAAAATGGTTTGTTCTTGCATATCCCTACTAGTTTTCCAAA

TTAATCCCCCGGATACATATAATTCAGAAGAATATGTGAGTGATTCATACACAGCATCTC

TTTCTTTTATCAACGGTTCTACCAATTGATATCTTTCCACAAATAATTGAAATTCTATTT

CTTGATCTGTATCTTCAATTTTTGGAAACTTATAAAGTTCTTCTGTCAAACCCTGATCAA

TGAACCTATAAAATCCTTCAAATTGTATCTGATTAAATCCAGGTATTGTAGACATTCCCT

CATTTCCATCCCCGAGCATTTTGAATTTCCCATTTCTCAAAAAATCCCATT-ATTGGTCC

ATTCTTCATCGAATTAGCGAATTAGATAGATGATCTAGCAATGATGGAATTTCTATTCTG

TTTACTGAATCACATGAAATTTTACCCAACTCCATATTTGTAATGGAATATATGAACTAC

GTATGAACGGAGGAATAAAGAGAATTTTCTACTGGAATTTGCAACAGATCCAAAGGAATT

GATAAAACATTCCTAGAAACAGAATTCTGCTACTTAGACTTATTAAGGTATAGGGTTTTG

TATAGAATAGAAAAACCAAAATGATTGCAATTCTACCATTATTATGATATTACATATTCC

AATTTGAGAAAAATAAAAGGATTCGGCATTTGATCTTTTCACTGAGATAAAGACACAAAA

ATCAGAAACAATATACGATTTTTTTAGCACTTAACCACCTTTATGTTATGGATTTCGTTG

TTCAAAAAATGATTCGCAGAGAAAAGAGATATTTTTACTTTACTGATTTTTGAGTAGATA

TAGGATTTAACTGTATAAGAAGGGTTGCATTTATTAAACACGTGTGCTGTGCAGATAGCT

ATAATATCCGACTTTTCTTTTTTTGCCGTTCTATTCGGAACAGCCGGGGTCGTGCTCTAT

CAAAACGAAATTTCTATTCAATGCAAAATGGAAGTATGATAATTTTGATAATTCCCTATC

GACAACATATCTAAATAATAGATATCTGTGTAACAATTTCTGTTCTGGGGTTTACATATA

CTCATCTATTTTGTTATAATAGAAATTGAGAAGGATTTTTTGATTGAAAAAATCAATACT

GATTAGTTCTGTCTCAATTTGTATTTTATAATGTCATTAGGAAAACACAATTTGGAGATT

CAAATCCCAGAATTGTTCATGAATTCGAAGTAAGCAGTCAATAGTTAATGCTTTAAATTT

GTCCTATATTTTGGCTAAAAATCCACATTTTATTTCTCAATAGAAAAATGAGATATTTTT

TAGCATTGTGGATTTTCAGATACTATACAATCAATCGAAGGGATGGATCAAATCCAATCA

AAAAAAGGAGGTTTCTTTTAGGAAAAGGATTAAGAAAAATAGGACTCAAAATGCAAGTAC

AATAAAAATTCCGTAATCCAGGAAAATTATCATATCCATTTTGTATCATTTTGGCGGCAT

GGCCGAGTGGTAAGGCGGAGGACTGCAAATCCTTTTTCCCCAGTTCAAATCCGGGTGTCG

CCTGATCAACAAAAAGCTCTAAATCTTTTCTTCTCTTCTGTTCTGTTGATACTTGTTTGA

TTCTAAGCATCTGGTCTGAGGGTTTTCTAAAAGATTGTGAATCCTTGCATCTAGGATTCA

AGGAAATATTGTAATCTAATGATAGAGGGGCTGTCAAGACTTCACGATTCCCTTCTATTA

CTAAGGTAGTGTCGAATGACTGGATCTTGACTTAGATTGGAGAGCCTGATAGGAAATCTG

ATTCATGTGGAAAGGGGCGGAAGTTGCTTTATTTTATATACGACGGACTCGCGAGAATCT

TGGAGTGCTCAGGTATTCAATCAATATTAGATTAGATGGATAATTGACTTTTATAGAAAA

AGGGGCAAAACCAAATTCTTTCTTTTCGGCAA--CCCCTAGTCAAGCCCCCCTCTTTCAC

AGCGATAATCGGGAAAGGGGATACGGATTAGATCAAACAAAGGGGGAAATAGGGGTCTGT

AATAGATAGTGATTTCTCTTTTTTAGTGATTTCCCCCTTTATGTTTTTACTTACGAAGGT

CAACAAAAAC-AAAAAAAGAATAGGCCTTATTATTCCTACATGTTCCCATTCCCTTGAGA

TGTTACTACGTATTTTGCTTGTGTTTAATCTTTCACGATTCAAAAAAGAATCATTTATTG

GATTGGTTTCTCAATAGTGTTCGGTCAGAATCCCTTTTTGACTCTGCACCATTGATTCCA

CTATTATTAGAAAGGAATAATGGAATAATTCCTTCATATTTATAGAGATAGGGGACATAA

CTCACATGGATATAGTAAGTCTCGCCTGGGCTGCTTTAATGGTAGTCTTTACATTTTCCC

TTTCACTCGTAGTGTGGGGAAGAAGTGGACTTTAGAAGTACTACTAATTGAGTTGAGGAA

TCAAACTGTATCAATTGTTTTATAGATCGTTCTGCAACGCGTTTTGAACTATTTAAAATC

AAAATATCTGAATTTCCAATTCCATTGGAGTCCAATGGAGTAATGTATGATAGGAATCAT

ACTCTTTCAATCAAAGAACTATTTCAATGATTCCCATGTTTGTATTTCGAAAGGAAAGGG

ATCCAGATGATTGGAAATTTTTTCCAATCTAATTCTTCTGAAATTTTCTATTTCAATTAA

GGGGCTCTTACTATCCTTATAGATTAAGATTAGATGGATACTGAGGAAGACCAGACCTTT

TTTTGATCCCTCTTGACTCTTCAAAGAAGAAGTCGTTTTGTTAAGTGTATACGCACTTTC

TATGAGAAATGATATAGACATAGTGGTTGTCTAACGAGATATGCAATAATAAGATCTTGC

CTCAGGCGAGTCACATATTGCGCATTTACCGATGGGTTTCTAATTTTAGAAAGGAGATTT

TATCTTTATCGACTTATTTGATATCATGGTTCGGGCGTTAAAAATCGGTGAGGTTTACTC

TTCCTTTTCGAAATCCGAAGAAGTGCCCGTGGTCCTCCTGTCAATAGTTAAATCAATTAT

TTCTTCGGAATACTAAA-AAAAAGATTACTACGCGATTTTAGTAATCTATATGCCCATAT

CGTTTTTCAATCATTGATTCTTTCCATAAATACCGATATTCAGATTGGAAATCATAAAAA

ATCTAGTAATTCGAATCATAATTAGAATCATAAGATAAGAGTTAAGGCGATTATTTCAGA

TTGATCGGAACAAGTAGATGTAGCAAATAAATAGAATTGGGTGCTATGTCAATTCCATAC

AATATAGGGAATTTATATACACATATATGAAGAGAATATTGTAGATTGATCTATATAAAA

TGAAGCCTCTATCTTTATTCTAGAGTAGAACTTTATAGACTAAGAGATAGATAGTATGGT

AAGAAAGATCTTTCTTACCATACTATCGAATTCATAAAATACTGCCGATTATAGTCCGCT

CATTTCATTTAAGACGCGAAATTGGAATCCTTTTCATTTTACTTCGTCCATTTTTGATAA

GAACTCAGAAGGAAAGTTTCATTCAAATTCA-TTTGAAAATGGAATTGAATTAATCATTT

TGACTGACTGTTTTTACGTAAATGATAAGTAGAAAAGCGGTAGGAACTAGAATGAATAGT

GCAGTCGCAATAAATGCAAGAATATTTACTTCCATAATCTCATCGGTTTTTTTACTTCGC

AATAACTCGGGATTTAATCCCATAGAGATGATAAATCTTTCGCCTGTAAATTCAATGAAT

GAATTACCTCTCGACGATCTTGAATCGGATCAATATCATGAATAACAATATCTGAGCTAT

CAAATCAATTCGTCGTCGAGACTTGAATAGTATAACATAGGAAGTTCTTTTATCCATACC

GAATCCAAACTTGGATTCCTGACCCAATCAATCCAAAATTCCTTTATTTATCATTTGTTT

CCCTTCTTTTTTCTATAACCTACCTTACGTCTTCCTTGTACAATCATCTGATGATCATCA

GATTGCCCTTCCACTTCGATTAGTCACATAGTTACAAACCCAAACAAACAAGAAAAGCGA

AATGG-AAAAAAAAGAGTTAAGTTCTAAACTCCTTGTGATTTTTTGGAAGACGAAGACAA

AGAAGTTTGATAAAGATGAGGCCGGTATAAAAGATCTAATATCACTATTTTAGTGTTTGT

TATTTCTTCGATGGGACCTTAAAAATGGAAAAATAGGAAATAAAAAAAGCCCCTTTGTTT

TGGAAATTCAATTCTGCCCCCTGACATCCTTTCATAGAAAGGGAGAAATTAATTGATGTA

TTTATTGGATCCGTCGGGACTGACGGGGCTCGAACCCGCAGCTTCCGCCTTGACAGGGCG

GTGCTCTGACCAATTGAACTACAATCCCAGGGAAATAAGGGATCTAGCAGAAAATTTTAT

TCTTTTTTTATCTTCGTATTTCGTATGGGGTATTTCGGAAGGACAAGGGGATTATACAAT

CTCATGGTAGATTGGCGAATTATTGGGCCGAGCTGGATTTGAACCAGCGTAGGCATATTG

CCAACGAATTTACAGTCCGTCCCCATTAACCGCTCGGGCATCGACCCAGGAAGAATCCAC

TTTAGGCTTATTGGTAATCCATGATCAACTTCCTTTCGTAGTACCCTACCCCCAGGGGAA

TTCGAATCCCCGCTGCCTCCTTGAAAGAGAGATGTCCTAAACCACTAGACGATGGGGGCC

GACTTGCCCAACCGCCCTCATACTATGATCATAGTATGAACAGTTTTTTGAAATTGTCAA

TATAATGGAATGGTATGATTAGACTCGCGGGATCTTTCCGTTTTTCAGAATTGTATAGAA

TTTTTTGATTCGTCATCCATATTCATGAATCGTTCATTAGAATATTAGAATCGCCACACT

CTATATAAATAGAGTATAGAAATCTATATTCTTTAGAATAATTTAATTAAAAAAAAAAAA

GAAATACAAACAACTAAAAATAATATGAGGGATAGGATTTGTTCAGGGAATGATTGGTCC

GTCAGAAAACAAAAGGGAGGTTTAATTTCGATTTTTTTGCTTTCATTCATTGTTAAGATG

AGATATCCTTATCTCTATCTCACACTAAGACGGGAAATTAACAACCAATAAATCTAGTAA

GCGGGATCAAGAAGTTATCGAAAATTTTCTCTAAGAATTTAGTTCAGGGGACAAGTAGAA

TCTCTTCATCACATGAAATATCTTTAAATTTATGTAAAATTGGTAAGTGTACACGTATGT

TATGTATCAATCAAGTGAATTTTGTTTTAATGGGGATCAATTCAATAAAATAAATTAGGG

TCGGTCTTGAATTCATTTTATTTTACCCTAGACTTGCTAGGTAAATCCATTTGATTATTC

AAGAATCAGCCACTAGCCACTACGAGTCTACTGCATGTACTTATGTATATAATATATGTA

CATATAGAGATTTTATCTACATAGTGACTCATTCGGGAATTAAATCAAATAAGCCCTTTT

AACTCAGTGGTAGAGTAACGCCATGGTAAGGCGTAAGTCATCGGTTCAAATCCGATAAGG

GGCTTTGGTTTTTTCATAAAAGTGCAGCCGTAGTATTCAGAAAATAGAGATA-TTTTTTT

TATTTGGAATAAAAAAGTAACTAACTGGATAATACGTTATCATTATACTGAGTTAGAGTA

TAGTAGTTCTAGTTAGAAAGTTGAACATTTTTTCAGTCAATTCTCATTATTATGAATAAT

GATAAGCCGCCTCTTGAATCACCAAAGATCCCTATTTTACATTATACCAATCAAATCCAT

TGGAAAGATTCGAAATCAACAAAAGAAAAAGTAAGTGGACCTGACCTATTGAATTATGAC

TATATCCGCTATTCTGATATTAAAATTCGATAGAGATGAAATTGGAATTGGAACAGTGGA

CCCCCCTTTTTTTTATTTCATTTCTTTGGACCTCGAAAGAATTTGTCGATATTTCCGATT

AAATCTTCTTGTTCCTAGATTTTCTATGGGAATAAATTGTTATTCCCTTCCTCTACAGAG

AAACCTTTCTTCCAAGTCACAAGATAAGAGCCATTTCCATTATCTTTCTTTGATTACAGA

TCAAGATGAATTTATATCTATCTAAGTATATTTAGATAGATATCTATCAGATCGTGGCTT

CATGTACCAAACATTTCTATATCGCTGCATCCGATATTTTTGTTCCGACAGTGTGATGGA

GAATGGATGCGAGAAAGAGACTTTCA-TTTTTTTTAAGGAAAAAAATAGGAAATTCTCTC

TTTCTAAGAGATTAAACTCAATAGAAAAATATTCGAAGTATCTTTTTTGCTTTGACCCGT

GGGAAGATATACTCTGTAGTTTTAGATTTATCTGAAGGAAAAGGAGATAGAACAAAGAAG

ACACTCAAAGAAAATGAAAAAAAAAAAAATGGAATAAAATAATGTAATTGGAATAGTTTA

GTATACATAGAAATTAGAATAATCTATAAATATCTTTATTTTTCTCAATCTCACGAACAA

GATCTAAGAATAACATTAGTTAATCGAACAAGAGGGGGGTAGATCGGAGGATCAGTTAGT

AGTGAGAGAGGGGATCGCTTGTTCCTTGAAAAGTTCTTTCAAAGGATTCATCTATCTGAT

TGATGAATCATAAGAAGACAATTCATGGTTCATATTCTTAGTAAGAAAGAATAATCAAAT

TGAGTTCATGGATTTCCCTAGGTCAGTTTATGGGCCAATCCAATAAAGGATTTTTATCTT

CGAAACCCATTGGAAGGGGCAGTGTAAGAGAAATCATAGAGAAATGATCGAATCTTCGGA

CGCCCCGAAAATACTGTGAGGTGCTCGGAAATGGTCGAAGTAGTTGAATAGGAGGATCAC

TATGACTATAGCCCTTGGTAAATTTACCAAAGACGAAAATGATTTATTTGATATTATGGA

TGACTGGTTACGGAGGGACCGTTTCGTTTTTGTAGGCTGGTCCGGTCTATTACTCTTTCC

TTGTGCCTATTTCGCTCTAGGAGGTTGGTTCACAGGTACAACCTTTGTAACTTCATGGTA

TACCCATGGATTGGCTAGTTCCTATTTGGAAGGCTGTAATTTCTTAACGGCCGCAGTTTC

TACTCCTGCTAATAGTTTAGCGCATTCTTTATTGTTACTGTGGGGTCCTGAAGCACAAGG

AGATTTTACTCGTTGGTGTCAATTAGGCGGTCTGTGGACTTTTGTTGCTCTCCATGGCGC

TTTCGGACTAATAGGTTTCATGTTACGTCAATTCGAGCTTGCTCGATCTGTTCAATTGCG

ACCTTATAATGCAATCGCATTCTCTGGTCCAATTGCTGTTTTTGTTTCTGTATTCCTGAT

TTATCCACTAGGTCAGTCTGGTTGGTTCTTTGCACCTAGTTTTGGTGTAGCAGCTATATT

TCGATTCATCCTCTTTTTTCAAGGATTTCATAATTGGACGCTAAACCCATTTCATATGAT

GGGAGTTGCCGGTGTATTGGGTGCTGCTTTGCTATGCGCTATTCATGGTGCTACCGTGGA

AAATACTTTATTTGAAGATGGTGATGGTGCAAATACATTCCGCGCTTTTAACCCAACTCA

AGCTGAAGAAACTTATTCAATGGTCACCGCTAACCGCTTTTGGTCCCAAATCTTTGGGGT

TGCTTTTTCCAATAAACGTTGGTTACATTTCTTTATGTTATTTGTACCAGTAACCGGTTT

ATGGATGAGTGCTCTTGGAGTAGTCGGTCTGGCCCTGAACCTACGCGCCTATGACTTCGT

TTCTCAGGAAATTCGCGCAGCGGAAGATCCTGAATTTGAGACTTTCTACACCAAAAATAT

TCTATTAAACGAAGGTATTCGTGCTTGGATGGCAGCTCAAGATCAGCCTCATGAAAACCT

TATATTCCCTGAGGAGGTTCTACCACGTGGAAACGCTCTTTAATGGAACTTTAGCTGTAA

CTGGTCGTGACCAAGAAACCACCGGTTTTGCTTGGTGGGCCGGGAATGCTCGACTTATTA

ATTTATCCGGTAAACTACTAGGAGCTCATGTAGCCCATGCCGGATTAATCGTATTCTGGG

CCGGAGCAATGAACCTATTTGAAGTGGCTCATTTCGTACCAGAGAAGCCTATGTATGAAC

AAGGATTAATTTTACTTCCCCATCTAGCTACTCTAGGTTGGGGGGTAGGTCCTGGGGGGG

AAGTTATAGACACCTTTCCCTACTTTGTATCTGGAGTACTTCATTTAATTTCCTCTGCAG

TATTGGGCTTTGGCGGTATTTATCATGCACTTCTGGGACCTGAGACACTTGAGGAATCTT

TTCCATTCTTCGGTTATGTATGGAAAGATAGAAATAAAATGACCACAATTTTAGGTATTC

ACTTAATCTTGTTAGGTCTAGGCGCTTTTCTTCTAGTATTCAAGGCTCTTTATTTTGGGG

GCGTATATGATACTTGGGCTCCGGGAGGGGGAGATGTAAGAAAAATTACCAACTTGACCC

TTAGCCCAAGTATTATATTTGGTTATTTACTAAAATCGCCCTTTGGAGGGGAAGGATGGA

TTGTTAGTGTGGACGATTTAGAAGATATAATCGGAGGACATGTATGGTTAGGTTCCATTT

GTATACTTGGTGGAATCTGGCATATCTTAACCAAACCCTTCGCATGGGCTCGACGCGCTC

TTGTATGGTCTGGGGAGGCTTACTTATCTTATAGTTTAGCGGCTTTATCCATCTTTGGTT

TCACTGCTTGTTGTTTTGTCTGGTTCAATAATACCGCTTATCCTAGCGAGTTTTACGGAC

CTACTGGACCAGAAGCTTCTCAAGCTCAAGCATTTACTTTTCTAGTTAGAGACCAACGTC

TTGGGGCTAATGTAGGATCTGCGCAAGGACCTACAGGTTTAGGTAAATATCTAATGCGTT

CCCCAACCGGAGAAGTCATTTTTGGAGGAGAAACTATGCGTTTTTGGGATCTGCGTGCTC

CTTGGTTAGAACCTCTAAGAGGTCCAAATGGATTGGACTTGAGTAGGTTGAAAAAAGACA

TACAACCTTGGCAAGAACGGCGTTCCGCAGAATATATGACTCATGCTCCTTTAGGTTCTT

TAAATTCTGTGGGTGGCGTAGCTACCGAGATTAATGCAGTTAATTATGTCTCTCCTAGAA

GTTGGTTAGCTACCTCTCATTTTGTTCTAGGATTCTTTTTCTTCGTAGGTCATTTGTGGC

ACGCGGGAAGGGCTCGTGCAGCTGCAGCAGGATTTGAAAAAGGAATTGATCGTGATTTTG

AACCTGTTCTTTCCATGACCCCTCTTAATTGAGATGAGACAGGAGATCCAATGCTTGGAT

TGAAGTAAGAATCACTTTGATTCTATCATACATA-TTGGGATCGGGTTATACTTAAAAAG

TATTCCTTTTTC-TTTTTTTTTTTTACTCATTTATATCTAATCTATTTTTTCTGGCTTGG

CTAAGTGGGATAGCCGAGCCATTCCCCTTTCTTTATGATACCAATCCGAGCCAAACCAAT

AGAAACAAATCTATTCAATGAGAAAAAAAAAGGAGAGAGAGGGATTCGAACCCTCGATAG

TTC-TTGTTCAAAACTATACCGGTTTTCAAGACCGGGGCTATCAACCACTCAGCCATCTC

TCCGAAAGACAATTTTTATTTTATTCCTCCGAATAGAACATGGCCATAGGGGTGGATACC

ACTACTATCTGTAGAAAGATCTCAGGTGTG-AATCTACCGATGGATCTATCTATCCGTAT

ATATATAATCCAGCATGTCCATTTGTGAAATATAAAAAACAAAATTCCATTTCCCCTGAC

TCCATGTATGAATAAAGTGGTCAAAGGGGTAGTAATAAGTCATATAGAATCAATTGATTC

ATGGTAAACTCAAATCCCTCGATGATGTATTTTATTACAATTTTTTGGCTGATAGAGGGA

TCAAATGGTATAGTTCATTTGTTGGTATCTTGGAGGATTAAAAGCATGACTCTTGTTTTC

CAATTGGCTGTTTTTGCATTAATTGCTACTTCATCAATCTTATTGATTAGTGTACCCGTT

GTATTTGCTTCTCCTGATGGTTGGTCAAGTAACAAAAATGTTGTATTTTCCGGTACATCA

TTATGGATTGGGTTAGTCTTTCTGGTGGGTATCCTTAATTCTCTCATCTCTTGAACCTAT

TCGTCCCAGATCCAAAACCGAAATGACCCCCCCCAAAAA--AATTTTCTCGGTTATGAGA

CACATTACAATTTAATATAAGTCCCCAAAATAAAATGGGGGGGTCAAACTTCTTGCAAAC

TTCTTGAATT-AAAAAAAATACAATTTCAATTTATTATATTAATAATATAAATCGCCCCG

AAGAGAGTCTCTGGCCCGACACTGCACAAATACGATCCAGGTATATATATATATCATATA

TGTGTGGATATATTGTGTATTAAGAACAAAAATTGCGGATATGGTCGAATGGTAAAATTT

CTCTTTGCCAAGGAGAAGATGCGGGTTCGATTCCCGCTATCCGCCGAAGATCAAGATAAA

GTCATTTTTCCTTTAATATGATAAAGGATTGGGTATAGTTGGCCATGATAGTGTCGCGAG

TCTATCCCTTCTTTTTTTCCTTTCCTACCA-CCCCCCTTTTTGGGGGGGTAATTAATTAC

TAGTTAACAGAGCCAAACCCCCTTTTGATAAAAAAAGATATTGCGGAGACAGGATTTGAA

CCCGTGACCTCAAGGTTATGAGCCTTGCGAGCTACCAAACTGCTCTACCCCGCGTTGAAG

AGAAGAAGTGAAAACTAATAGACAAACAAGAATTGAATGTGCCCCTCTACCATATCTGTA

CAAATAGAATAGCCCATTTATACAGAATGGTAAAGAGGCCCTATATGATCATCGACCATA

GAAATGAAAGGTTAATCCTTACCAACTTGATCTTGTTGCTCCTGGCAACAAACACGCATG

AACCATTTCACGAAGTATGTGTCCGGATAGTCCAAAGTCTCGATAGTTAGCTCTCGGTCT

TCCGGTCGAAAAACAACGTCGATGAAGGCGTGTAGGTGCACTATTCCGTGGTGGGGATTG

TAACTTTCCATAAATTTCCCATTTGTCACTCAACGATGGAACTTTTCTTATTTCTTTTTT

TGAGGATCGACGAATCAAATGATATTTCTGTTCCAATTTTTGCCTCTTCTTCTCCCTCTG

AATCAAACTTTTCTTTGCCATAATGGTTCAGGTCCTATTAGTATCCATGATACAAGTCGA

ATCCTAGATGTAGAAATATAAGAAGGTGGACCCTCTCTCCATCGAAAGAAATGAGATTAT

CGCGGATACAACACATTCAAAAATTAGCCAAATTTCCCCGATGTAGAGGCAATCAAAAAA

GCCGCATAAGTGAATATATAACCTACAGAAAAGTGGGCTAATCCAACCAATCTTGCTTGG

ACAATGGAAAGAGCCACAGGTTTATCTCTCCATCGAATCAAATTGGCCAAAGGTGTGCGT

TCATGAGCCCATGCTAAAGTTTCAATCAATTCCTGCCAATATCCACGCCAGGAAATTAAG

AACATAAATCCAGTAGCCCAAACAAGATGTCCAAATAAGAACATCCATGCCCAGACCGAT

AAACTATTCATACCAAAAGGATTATATCCATTGATAAGTTGTGAAGAGTTTAACCATAAA

TAATCTCTTAGCCAACCCATCAAGTAAGTGGAAGATTCATTAAACTGTGAAACGTTACCC

TGCCATAATGTGATGTGCTTCCAATGCCAATAAAAAGTAACCCATCCAATAGTATTTAAC

ATCCAAAAAACTGCCAAATAAAATGCGTCCCATGCCGAAATATCACAAGTACCCCCTCGT

CCCGGGCCGTCGCACGGAAAACTATAACCGAAATCCTTTTTATCTGGCATTAACTTGGAA

CCGCGTGCATCTAAAGCACCTTTTACTAAGATCAATGTAGTTGTATGTAAACCCAGAGCA

ATAGCATGATGAACCAAAAAGTCTCCAGGACCTATTGTCAAGAATAATGAATTACTATTT

TCATTAATAGCATTTAACCAACCGGGCAACCAGATGCTTCGACCCGCATTGAATGCCGGA

CCGCTCGTTGAAGATAAAAGTACATCGAACCCATATGAAGTTTTCCCATGAGCCGATTGT

ATCCATTGAGCAAATATAGGTTCGATCAAAATTTGCTTTTCTGGAGTACCAAAGGCAAGC

ATGACATCATTATGAACATAAAGTCCCAAGGTATGGAATCCCAGAAAGAGGCTGGCCCAA

CTTAAATGAGATATGATAGCTTCTTTATGGTCTAACATTCTTGCCAATACATTATCTTCA

TTTTGCTCCGGATTGTAATCTCTAATGAAAAATATAGCTCCATGAGCAAAAGCTCCTGTC

ATGATGAATCCTGCGATATATTGGTGATGAGTATATAATGCAGCTTGAGTAGTAAAGTCT

TGTGCTATGAATGCATAAGCAGGTAAAGAGTACATGTGTTGAGCTACTAAGGAAGTAATA

ACCCCTAAAGAAGCTAGAGCAAGGCCTAATTGAAAATGAAGCGAATTATTGATTGTGTCA

TAAAGACCCTTATGCCCTCGTCCCAATCGTCCCCCCGGAGGAATATGTGCATCTAAAAGA

TCTTTGATACTGTGTCCAATCCCGAAATTCGTTCGATACATATGACCAGCAACAAGAAAA

ATAAATGCAATAGCTAGATGATGATGAGCAATATCGGTCAGCCATAAACTTTGTGTTTGT

GGATGGAATCCCCCGAGAAGAGTTAGAATGGCAGTTCCTGCTCCTTGGGAGGTCCCAAAT

AAATGACTACTTGAATCAGGGTTTTGAGCATAAAGATTCCACTGACCTGTAAAAAGTGGG

CCTAACCCTTGGGGATGCGGTAATACCTCTAAGAAATTATTCCATCGAACATACTCCCCT

CTGGATCCAGGAATAGCGACATGTACTAAATGCCCTGTCCAAGCCAAGGAACTTACGCCG

AAGAGTCCTGACAAATGATGATTCAGACGAGATTCGGCATTTTTGAACCACGAAACGCTC

GGTTTCCATTTCGGTTGTAGGTGTAACCAACCTGCTATTAAGGATATGGCAGAAAGAAAT

AATAGAAAAAGAGCTCCAGTATAAAGATCTTCATTAGTGCGTAAACCGATTGTATACCAC

CACTGATAAACACCAGAATAAGCGATATTCACTGGGCCAAGAGCACCCCCTCGAGTAAAA

GCTTCCACAGCCGGTTGACCAAAATGAGGATCCCAAATCGCATGAGCAATAGGTCTTACA

TGTAAAGGGTCCTGTACCCATGATTCAAAATTTCCTTGCCAAGCTACATGAAACAGATTT

CCGGAAGTCCACAGAAAAATTATTGCTAATTGACCGAAGTGAGAAGCAAAAATATTCTGA

TAAAGACGTTCCTCAGTAATATCATCATGACTCTCGAAGTCATGTGCGGTAGCAATACCA

AACCAAATACGACGAGTAGTGGGGTCCTGAGCTAAGCCTTGGCTAAACCTTGGAAATCTT

AATGCCATAATGCCTTTCAAATCCTCCTAGCCATTATCCTACTGCAATAATTCTTGCTAA

GAAGAACGCCCATGTTGTGGCAATTCCACCCAGAAGGTAATGGGTTACTCCTACAGCACG

TCCTTGTACAATGCTCAAGGCTCTCGGCTGAGTAGCAGGAGCAACTTTTAATTTATTATG

AGCCCAAACGATGGATTCAATAAGTTCTTGCCAATAACCACGTCCGCTGAATAGAAACAT

CAAACTAAAAGCCCATACAAAATGAGCACCTAGGAAAAAAAGTCCATATGCAGATAATGA

AGAACCATAAGACTGAATTACCTGGGATGCCTGTGCCCATAAGAAATCCCGGAGCCACCC

ATTAATAGTAATAGAACTCTGGGCGAAGTTTCCTCCCGTGATATGAGTTACTACCCCTTG

ATCACTTATACTGCCCCAAACATCTGACTGCATTTTCCAACTGAAATGGAATATTACTAC

CGAAATGGAATTGTACATCCAGAATAGTCCTAAGAAGACATGATCCCAAGCCGATACTTG

ACATGTACCCCCTCTTCCAGGTCCATCACAAGGAAAACGAAAACCAAGATTTGCTTTATC

CGGTATCAAACGGGAGCTACGAGCAAATAGAACACCTTTCAGGAGTATCAATACCGTGAC

ATGAATCGTAAATGCATGGATGTGATGTACCAAAAAATCCGCGGTTCCTAACGGAATAGG

TAACAAAGCCACTTTGCCACCCACTGCTACTAAATCACCACCCCCCCAAGTTAAACTGGT

GCTTGCTGTTGCACCAGGAGCCGTTGCGCCAGGTGCTAAAGCATGGGTGTTTTGTATCCA

TTGAGCAAAAACGGGTTGTAATTGTATAGCGGTATCTGAAAACATATCTTGAGGACGCCC

TAAAGCGCTCATGGTATCATTATGAATATATAAACCAAAACTGTGAAAGCCTAGAAAGAT

ACATGCCCAGTTGAGATGTGATATGATTGCATCACGATGTCTAAGGACACGATCTAATAG

ATCGTTGTATCGAGTAGTTGGATCATAGTCTCTTACCATAAAAATGGCTGCATGCGCAGC

AGCACCAACTATGAGAAATCCACCAATCCACATGTGATGTGTAAACAATGACAGTTGGGT

CCCATAGTCAGTAGCTAGATATGGATAAGGGGGCATGGAATACATATGGTGAGCTACAAC

AATGGTTAAAGAGCCTAACATAGCTAGGTTAAGAGATAATTGAGCATGCCATGACGTTGT

TAGGATCTCATATAGGCCTTTATGACCCTGACCTGTAAATGGACCTTTATGAGCTTCTAA

AATATCTTTTAGGCCATGGCCAATGCCCCAGTTAGTCCTATACATGTGACCCGCTATCAG

GAACAGAATTGCAATAGCTAAATGATGGTGTGCAATATCGGTCAGCCATAGACCCCCAGT

TACTGGATCTAATCCTCCACGAAAAGTAAGAAATTCCGCATATTTTGACCAATTCAAGGT

GAAAAATGGGGTTGCTCCCTCGGCAAAACTTGGATAAAGTTGAGCCAAAAGTTCTCGATT

CAAGATAAATTCATGAGGAAGCGGTATCTCTTTAGGATCTACTCCAGCGTTTAAAAATTG

GTTAATCGGTAAAGATACATGTACTTGATGCCCCGCCCAAGAGAGAGACCCAAGTCCTAG

TAGCCCCGCTAAATGGTGATTCAACATAGATTCTACATCTTGAAACCAAGCCAATTTTGG

GGCCGCTTTATGATAATGAAACCAACCAGCAAAAAGCATTAACGCTGCAAAGACCAATGC

ACCGATTGCGGTACAATAGAGTTGTAATTCACTAGTTATTCCAGATGCTCGCCAAATCTG

AAAAAACCCAGAGGTTATTTGTATTCCTCGGAAACCCCCGCCCACATCACCATTCAATAT

TTCTTGGCCCACTATTGGCCAAACCACCTGGGCACTAGGCCCAATGTGAGTTGGATCACT

TAGCCACGCTTCATAATTGGAAAAACGAGCACCGTGGAAATACATACCGCTCAGCCAAAG

AAAGATGATGGAGAGTTGACCGAAATGGGCACTAAATACTTTTCGAGAGATCTCCTCCAA

ATCACTGGTATGGCTATCGAAATCGTGAGCATCAGCATGTAGGTTCCAGATCCAAGTGGT

AGTATCAGGTCCTTTAGCTATTGTTCTTGAGAAATGGCCCGGTCTGGCCCATTCCTCGAA

AGAAGTTTTTACGGGATCCCTATCCACCAAAATTTTGACTTCTGGTTCCGGCGAACGAAT

AATCATTGAGTCCTCCTCTTTCCGGACAACACATACAAAGAGACCCGCCAACAGTCAAAT

AATTAGTAAACCTTTGAGAGATATTTCTATAATTAGTTTGTTTCTCTTCTATCTCCCATT

TATCTATTTTCTTTAGTTATTCACTAGAGCAATTAGGATCTGGAAGTCGATCCGGGGCAA

GTGTTCGGATCTATTATGACATAGCCATGAGGCGCTCAACGGACCTTTTTTATCTTATAA

AATCTTATAAAACCTTTTTGTAGCTTTGGATTGATGCAAAAACGACTTTTTTGTACAACC

TAGTGTAGATTCATATCTCAATTAGAAGGTCTTAGATAGAGCTGCTTTATCTTTTCCATA

GATGATATGAATTACTCTATTCCAAATCACGCGAGCAGCCATTACTAAGAGACATCCCGG

TATATA--TTTTATTTAGTTGTTAATTTAGATTTAAATTTAGGTTTTTAAAAAAGAATTC

GTTTTCATATTAATAATAAAATTAAAGTAAAGAAGTATATTTTTGACTCTATCCCTTTTT

TATCCCTACGAAATATCAAATGAAATAGAACGCTTAGAAGGGATATAATGAAATTCTTTG

ATTGGCTCTTCCCAAAGCAAAGGAATGATCCA-TTTTTTTATTTGACTGATGGGGCCAAC

AAACAATAAATTCTAACAAATATATATAAATAATAGAATAGAATTAATAAACTAAATAAA

CGGCGTCTTCTTTTATTCGAAACGCCCCGTGATCTTCAACCAATTATGTGCTTCAATATA

ATTACCAGGAGTAAGCGCTATAGCCTGTTTCCAATACTCAGCTGCTTGATCAAACCAAGC

CTCCGCAATTTCAGAATCTCCCTGGCGAATGGCCTGTTCTCCCCGGTCGGAATAGGTAGG

TTAATTCCTTCCCTTAGAACCGTACTTGAGAGTTTCCTAACTCATACGGCTCAGCAATCA

TTTGGTGTCCCCCTTTAATCTACCATATCTAACGAATGAGATTTCTGCTGGATCTATCCC

ATTTTGGGGGTTAACCAAAGAGGTTCATTACCTGAGTTTTAAACTGAAATTTGGATTCAA

TTTGGATTAATAATCCGTTTTATTTCGTTTTATCTTTTTTCCCACCTTCAGAAGAATAAA

TTCCTCCTATCGTTAGAATTTTCTGAAAGGTAACTATCTCGGTTTCATATATAAATTTCT

ATAGAATCTTTGAAAAAGACTTTCCTTCTTAAGAAAGAAAAGACTTACTATCTTTGGGAT

CTGATCCTACACCGCTGCTCAAGACTTTAGTGGATCGACTCTATTACATAAGTTGATTCC

TAATTTTTATCTCACATCATGAGTAGGTATATCTACCATCATGACATAAGTACGCAGTTA

TTATCGTATCGGCCCAAAACCTCGCTAATTGATCTTTACGGTGCTTCCTCTATCAATTAG

ATCCTTTTTTTATCCATAGAAAAAAGTAGCTAGGCATATCTATTTCTTCATATTTTGACT

TCTATGAAGTTTCTTTCTTTGCTACAGCTGATAAAAATCGTTGTTTTAGACGATGCATAT

GTAGAAAGCCTATTTTTTTCGTATTTACTAGCAGATTTTTTTTTTCTTTCTATAGTGGAG

ATAGTCGCACGTAATGACAGATCACGGCCATATTATTAAAAGCTTGGGGTAAGAATGGGT

TTCGTTCTAGTGCTCGAAAATAATATTCCAAAGCTTTCGTATGTTCTCCATTACTTGTAT

GGATAAGACCTATATTATAGAGTATATAACTTCGATCATAGGGATCAATTTCTAGTCGCA

TAGCTTCATAATAATTCTGTAAAGCTTCCGCATAATTTCCTTCGGATTGAGCTGACATCC

GTTACGGTCGTAATTCAAGTAAAAGAATCTCCGTTCCAGAACCGTACGTGAGATTTTCAT

CTCATACGGCTCCTCCCTTATGTGCATAAGGAGAATAATACATGAAATCAAAAAAGACGA

AAATATTCTCATTATGAACTGAGCAGGGCTAGTGTTTTTACAAGAAATCTCTAGCCAACC

TTCCTGCAAGAGATCTTTTCTTAACATCAAGCGTGTTGGGACTAGATAGAAATGAGAACT

CCAACAATTTCTTTGTTTTCAACGCCTCCTAATTTCCAGGAATTAGTCACTTCAACAGCC

TTCGATGGTTATACGGGTATCCAAAGTACGAACGAGATGGATGTTTGTTGTCCCAACCAT

TCTTTTAGTCCCGAGCCCGATAAGGAAAGGGATAATTTCTAACAAAGTTTTCGTGTTGTT

GATTCCTAGGTGTAGTGCTTCTTCCCCTATGCTGCCTATTGGTACTAGTGGAGTAGGATT

GACCCATAATAAAGAACCCCTAGGTGTAACCTTTCGCTCAATACTAGAATCAAAATTGAA

ACATAGCATCTGAGGTTGCATTAATCGAGGATACACGACAGAAGGAATTGTTCTATTTCC

AAACTTCACCTTCAACAAGCGTAGATTTATTTCCAAAATTTTCCTGAATCACGTGTCTTT

CTCGTAAGACTGAGAGAAATAAAAAAAAAAAAAGAATCAAATCACACCATCTCTGTAATA

GGTAAATGCCTCTTTTTCTCCTGAAGTTGTCGGAATTATTCGTAATAAGATATTGGCTAC

AATTGAAAAGGTCTTATCAATAAAATTTCCATTTATCCGCGATCTAGGCATAGGTAGCAA

TCCATTCTATAATTCTTCTCATTACCTCTCGTGGGAAAATGATCCCACAAAGAAAAGAAT

TGTACAGTACGAAATAACATAAAAATAGATTGATTAAAAAAAAGATTATGGGCCTTCTGT

TCCTGAATCAATACAAAATATTTGAACCCGATTCGATTTCATACTAATGTAGTAGTATAC

GGAACTATTCCGATTTCATTGAAGTTACAGATTAGAGTAACTCGATAAATTTGGATTGAA

TTATGATACAAAAACGAAAAAAGATCGAATAATCATTCTATGATGAAAATAGATAGAATA

ACCGCCTCTTTTGTCTGTTGTGTACATAGCAGGTATACAATCAACTATACAAAATGTTTT

AGAAATTTCTAATAGAGGGGGTTTTGTTGTTGAGAACTCTAAAACTGGAAAGGAATTTAA

TAATTTGTAGGGTATGGAATCATAGTCTCTATAGAATTATGATAGAAAGGTATCCATTAA

CCCTAGTCTAAAAAATCTAGACCCATCAATCAGTTGATTCATTCTAATTCATTGATTTAA

TCCATTCTAAATATTAGAAATAGAAGGGAGAAATTTTTGCAAACATGAATCCCATTTTTA

ACAATTTTTCGTAAGAAAAGAATTTGATCTTTATCTCGGAGCCTCGAAGGAAATAGCTTT

CTTTACTTTGATGAAAAATTTTCGATTTTTATTTG-TATAGTTAAAATGGATGTACCTAT

CCCAATAATCTATAATGGAATATGAATAACTCCCT-ATTCACTCGGTTTTTGGTTCATAA

TCATTATGTAGGAGAGGTGGCCGAGTGGTTCAAGGCGTAGCATTGGAACTGCTATGTAGG

CTTTTGTTTACCGAGGGTTCGAATCCCTCTCTTTCCGTACCTTCACTTAATAGACCGATT

TGACTAACAACACTGGATCAAATAGCAATGGAGACCTTTATTCCACTAGTTAGACCTTTC

ATTGATATAGATTCTCTATTCCTAATTGCCGTGACACGTAAAATACTATAAAATAGGAAA

AATTCTGGAAAGAAAATGAAAATATCCCTTCGGTCTATGATACATATACATAAATGGGAG

AAAATCCGGATCAAACCCGTATTTTTCTTACTTAATCTTAGGTTCATTTACTTCGATGAA

AGGGAAGAAAATTGCCCGAACCCTTGTTATTTTATTTGAGTTTAGGTTTAAGTCTGACGA

GAATAATATTCTACGACTAGCAATTCATTTATTTTCAAACCGACCCATTTACTATCTATT

ATTTGATTGACTAATCCTTTATATTGGAATGGGTGAAGGGTCAAATGGTTTGGCAATTCC

TCATGAGGGGATGAGTCGAGAGAATTTTGAATCAGAGTTCTGGATTTTTGTTCATCCTTC

GTCATAATAATATCTCGGGGTTTGCAGCGATAACTTGGTATATCTACTATACGCCCATTA

ACTAAAATATGTCTATGGTTAACTAATTGGCGGGCTGCGGGAATAGTCGAAGCCATACCC

AATCGAAAAAGGATGTTATCCAAACGCATTTCAAGTAATTGTAGTAAAACTTGACCTGTT

GACCCCTTGGCTTTTCCGGCGATACGAACGTATTTAAGTAATTGTCGTTCTGTAAGACCA

TAGTGAAAACGCAATTTTTGTTTTTCTTCTAGACGAATACGATATTGAGATTTTTTCCCG

GAACGTGATTGGTTTCTAAGATCACTTCCGGCTTTAGGCCTTTTATTAGTTAGTCCTGGT

AAAGCCCCCAGGCGGCGTATTTTTTTGAAACGAGGTCCTCGGTAACGCGACATAAAGACT

CCTTATTCCTAGTTAGAATTAATTTTTTTTTTATTTTACAGAATAAACCTAAACTAAAAC

TGAACTAAATGAAGCGAAATCTACTGAAGTAGTGTACTTGTACTATAAAGAAGAATGAGG

TAAATTGGATAAATATCCAGACTTTCTATTATTATATATATATAGAAAGGATCCTTTTCC

TGACATAGTTGGAAGTTCCTATAACTTCCAAAATTCATGGATTTTGGAAAAGGGGGAAGG

CACTTTTTCAATATTCTTTGATTTCAAAGGGACATTATCAATCATTAAAAAATGGAATAA

AAAATGAAGGAAAAGCCGGCTATCGGAATCGAACCGATGACCATCGCATTACAAATGCGA

TGCTCTAACCTCTGAGCTAAGCAGGCCCACATAATAGAAACTTTCTATCCATAGGAATTC

AATACACTACACTATAGTGTCTATAGAATATAGAAAAGGATAGAATATAGAATTTCAAAT

AAATATTAAATATTCTAGAACATAACCATTAATATAGCGATATAAAATTTCGATTTCTTT

ATCACAATTCTAATATTATTAGTATAGTAAATCTTAAATATTTTTAGATAGTCAAATTTT

CTTTTTCATTTTTGTTTGAATTCACATGACATTTGAAATTCTTTTTGTTACACTTCTCTA

TATTTATATTTTGAATTCTATTAATTCTTTCGAATTCGAATTATTTAATTGATTAGAACT

AATCAAACATTCCTCTGCTTTCATTCGTAAAGCGGAAAGAAAAG-AAAAAGAATCGACCG

TTCAAGTATTGCAAATTGCATGGGAAAAGTGGCAGGAAGAGAGACATATATATGGGGTAT

ATATCCATCTATATTGAATTGCCGATACAGAAATGATAAAATCCAATTTGATTGAATCAA

ATACGGGTTTCCGATAAGGAAGAAATCAAAGAGGGGAAAATACTTTACAATGAAATCCTA

ATCTCGGGAAGGGGGATATGGCGAAATTGGTAGACGCTACGGACTTAATTGGATTGAGCC

TTGGTATGGAAACCTACTAAGTGGTAACTTTCAAATTCAGAGAAACCCCGGAATTAATAA

AAATGGGCAATCCTGAGCCAAATCCTGTTTTCCCAAAACAAAGGTTCAGAAAGAAAAAAG

GATAGGTGCAGAGACTCAATGGAAGCTGTTCTAACAAATGGAGTGGACTGCGTTGGTAGA

GGAATCTTTCCATCGAAACTTCAGAAAGGATGAAGGATAAACGTATATATTGAATACTAT

ATTAAATGATTAATGACGACTCGAATCTCTATCTGTATTTTTTTATATGAAAAATGGAAG

AATTGATTCCACATTGAAGAAAGAATCGAATATTCATTGATCAAATCATTCACTCCATAG

TCTGATAGATCTTTTAAAGAACTGATTAATCGGACGAGAATAAAGATAGAGTCCCATTCT

ACATGTCAATACCGGCAACAATGAAATTTATAGTAAAAGGAAAATCCGTCGACTTTAAAA

ATCGTGAGGGTTCAAGTCCCTCTATCCCCAAAAAGCCTATTTGACTCCCCAAATATTTAT

CCTATCCCTTTTTCATTAGCGGTTCCAAATTCCTTATCTTTCTCATTCACTCTATAAACG

TATTTGAGCGTAAATGATTTTCTCTTCTCACATGTGATATATAATACACATCCAAATTAA

GCAAGGAATCCCCATTTGAATGATTCACAGTCAATATCATTACTCATACTGAAATTTACA

AAGTCGTCTTTTTGAAGATCCAATAAATTCCAGGACTTGGAGAAAACTTTGTAATCCCCC

CTTGTCCTTTTAATTGACATAGACCCCAGTCATCTAATAAAATGAGGATGGGATGTTACA

TTGGGAATGGTCGGGATAGCTCAGCTGGTAGAGCAGAGGACTGAAAATCCTCGTGTCACC

AGTTCAAATCTGGTTCCTGGCACATGATTCATTTGTATGAGTCTCTCAATTAGAAATTAA

TTGATATGAATCGAGATACATATTCATTTCTAATCTAGATCATAATACATACTTTTATCT

ATCTTAGCGAGATATACCCCATCTATATAGATGGGTAGAGTTTTTTAAATAAAGTATCTA

AAAGAATTCAATTCTATTTTCTC-TTTTTTTTCTTTCGTTCAAAAAATCTGTTAATACTT

CATACATATTTGAAAAGTTCAATTAGTTGGTTGAAAGACTAAAAAGTCGAAGTTGAAAGA

GACAAGATTCGGTTCAGATACCAT--AAAAATAAAGAAAATCTGATACCCTTTCATTTCT

TTGTATTTTCTCTTTCATATTCGATTTCTTAATTCGTCCCGACATGACTTTCTAGAACCG

GTCTAAGTAATGTGCGCAGTACAAAGTTCATGATGCAGAACGCGTTTGATTCATCCTATT

GGTTTGGCTTGGCTCATACGAAATAAGTAGCTTCCAAATAAATGTAAGAATTCCCAACGA

A-CCCCAATTCTGTCTTTTTTTGTTAGCCTATCCATAATTCCTAATATAAATATATAAAC

CAAACTTCAATTAGAATTATTCTGGTTAATCTAGAACCGAAAGTACAATCCTTGAATCTC

TGAAATTGTATAAGTGGAAATTAGTTTCTTATCATTCAATGAGCATCTTGTATTTCATAA

AAATTGGGGGCAATATAATCCTTACGTAAGGGCCATCCTATCCAACTTTCAGGCATTAAG

ATACGTTTCAAGCGTGGATGATTATCATAAGAGATTCCCAACATATCATAAGATTCTCGT

TCTTGAAAATCCACACTTTTCCAAACCCAGAAAACGGACGGAATTCTAGGATTCCTCCTT

GAGGCAAATACTTTTATGCATACCTCTTCTGGTTGATCCACACCATACTCTATTCTCGTA

AGATGATACACACTAGCTAACAGTCCGCCAGGTGCTACATCATAGGCACATTGGGAGCGT

AGATAATTGTAACCATATACATATAAAATGACAGCAATGGAATGCCAATCCTTGGGCTTT

ATTTGTAAAGTCTCTATTCCTTGATAATCAAAGCCCAAAGATCTATGAATTAGCCCATGC

TTGACTAGCCAAGCAGACAAACGACCCTGCATCTTTTTTATCTCCCGCATTTTTAGCTAG

AATATTTCACATTCTCGATCAAATTTATGAAGATTCACCCACTACTTGTTATTCTGTACA

ATACAAAGGAATCCTGTCTAATTCACTAATTCGTGAGAAGATACTGAATTTTTATATTTG

AAAAAGTTTTCAGTAGGGATCTCTGAAGTAGATGGCGGTTGATAAAGGAATCCTTGATCA

TAATTTCCAGTATTCATACTGCGTCCAACATGAAACTTGTGATTGGTAGTAAAACACCGA

TTTGCTTGTTGAGACCTAATTCTATCTTCATAGATTTCTCGAGATATTTTCTTACGAAGT

TTTGTTATAGCATCTATAACTGCTTCCGGTTTAGGTGGACAACCTGGCAAATAGACATCC

ACGGGAATTAGCTTATCGACTCCCCGAACAGTACTATAAGAATCGGTACTGAACATCCCT

CCTGTAATTGTACAGGCTCCCATAGCAATAACATATTTTGGTTCGGGCATTTGCTCATAT

AATCTCACTAAAGAGGGTGCCATTTTCATTGTTACTGTTCCGGCTGTTAAAATTAGATCC

GCTTGTCTAGGACTCGATCTTGGTACTAATCCATAACGATCAAAGTCGAATCGTGATCCT

ATTAGTGAAGCAAATTCAATAAAACAACAACTGGTACCATAGAGAAGCGGCCATAAACTA

GAGAGCCTTGACCAATTTGAAAGATCATTTAATGTAGTTGAAATAACTGAATTTTGGACT

GTTTGATCAAGTAAAGGAAACTCAATGGAATTCATAACTTTTTCAATC-TTTTTTTTTCC

CTTTTCTTTTTATTGTCTGAATATTCAGGAGCTAAGACCATTCCAATGCCCCCTTTCGCC

ATGCATAAACTAAACCAACAATTAAGATAAGCACGAAAATTAAAGCTTCTATAAATACAG

ATACACCCAATACATCGAAACTCATTGCCCATGGATAAAGAAAAACCGTTTCAACATCAA

AAACAACAAAAACGAGAGCAAACATATAATAACGGATTCGAAATTGTAACCAAGCGTCGC

CCATTGGTTCTATACCCGATTCATAACTAGAAAGTTTCTCCGGCCCTTTCCTAATCGGGG

CTAAAATCCCGGAAATGAAAAATGCCAAAATAGGAATAAGACTTGATATTATTAGAAATG

CCCAAAAAATATCATATTCGTAAAGCAAAAACATAGACGCACTCCTATGAACGTGGAAAA

TATACCGGATGGGTCGATTCGAATTGAAATTTTCAAGTCATCCATAACTGTTTAGTCAAA

ACAAGAATTCATTTTGACCAAACCATCTAGTTTCCTTTGTTTATTGTGGGGCATATCTCA

TTTCAAGATTCATCGACTGACTTGACTGGAATCCTATTTCCAGTCTACTTATTTTTATTT

CTTTTTATTTCTATTTTATTTAGTTAGTATAACTCTAACTATTACTTTTATACAAATTCT

CTTGTTTTCACCTAGGATTCTTGCTAAAGAAACCTAGTTCCAAATAAAAAGAAAATAGAA

TTCTTATTTTGTGTTTAGAATTTCTAACTTATTATTTTTAAAAT--TATATATTATTCTA

AATTCTTTAGAGATTTCTATTTTTTTTTTTAGGAATAGAATCTGGAGTTTGTCGTTTTTT

TCTTAGTGATTTCGAATGGAACAAGTATTCAAATGTAAGAGAATGGATAGGTATTTTGAT

TTCTAATATCTTAGTGTTGGTATATTCCTTTTATTTTATTAGAGGGGTTTCTCTTGATTG

AATACAGAAAAAGAAGACCATCCCCTTTGTGCTTCGATAGGTCTAGGTAAGGTATACGAA

GAAAAAGCCTATTTGACATTGTTGACAATGAAACTTACCAAAGAGATTCGTTTCTTAACA

AACTTTGGCTTGTTGATTGTGGAC-AGGTAAATTCATATGGAATTTACCTCCGAAGATTA

ATGACGAAAGGTTGGTTTGTTTATCCACGATTGGATAAATATCGATTCGATCCCTTTTTC

TTTCAGTTTTATGTTCTGCTTTAAACGATTCCCGTGAGTGAGTTTATAGGAATAATTTGG

ATTTCGATGAACCAACCCAACCGGTCAGTTACAAGCAACAAACAATAATGAAGAAATGAA

AATTCGAATATTTTCTATTTTGAATTTTCATTTTATAGGGCTCTAGGGCTATACGGACTC

GAACCGTAGACCTTCTCGGTAAAACAGATCAAACTTATTATTATCAAAATGATCTGAACT

GTTTCAAAGACCCAACATGCATTTTTTTTGCCTTGGGCTCTTTCATTAACTGATAGAAAT

ATCAGCCAGTCTGCCATATTTTTCAAAATAAGATTAAGGAGATGGCTCCATGTGCTCTGA

TTCATTATTTGGGATTCTGATCCAGGAGCACTACCAAAGTGTTTCAAAGGTGGGGTTATC

TTGACGTAGGTCTGCCTCTGGCCTAGATCAACCTAAGTTAAATGAAGTCTCTATCGTTCG

GCTTAAAAAATAAAATATGAAACTTCATACACCTTAAAGTTCATAGGACGAAAAGAGATT

TTTTGAGGACCTTATACTCATTATGCCTAGCATTGAATGGACTGGTATTGACCTTATCAA

TATCTCAAATCAATGTATGGGGTCTGTTTGGTACCTAAATGGGCACCAAAATCGGACCGA

ACCATTTGTCAGGCTACTGTTCCCTCACAGTTATGGAGTAAGACATCGATTTCTCAATAA

GATGCATTTTTTTGATTGTATGATGGACCCCCCTGAAAAACATTGGCGCGCGTGTAAACG

AGGTGCTCTACCAACTGAGCTATAGCCCTTAGTGCTTGTGATACATATTTTATCATGGAG

ATAATTTCTTGTCAAGATGAATATTCTATGATCCAACATCCTCCATTTTTGAGTGGTATT

GCTTGTATTAGTATTGCTCATAAGTAATATGATATTTATAATCCATCGATGTCATGGGTT

CCATTTGGTTATCTTTGGGATGATAAATGACCTACTTAACTCAGTGGTTAGAGTATTGCT

TTCATACGGCGGGAGTCATTGGTTCAAATCCAATAGTAGGTAGAACTTATTAGATACCGG

AGTCAATGGTACCTAATAAGTTTTTCGACCCACCCTCTTTTATTTTTATTGATTTTGTAT

CTTTTTTATTTATTTTATTTTCGTTGTAGCAAAATAGGATTCTGCTTGATTGGATTCACT

CGACAGAATCCTATCAAAATAGGCTTAGAAACAGAACTTCTTTTGATTATTCGAACGCGC

CAACTAGTTAGGAAATCACATTGACAGCCTCTACTCTTGTCCTAGCTCGTCGGAGAGCTA

GATTCGCCTCAATTATTTGTCTCTTTCCTTCAGCTTTTCTCAAATTAGCTTCGGCTATTT

CAAGAGTTTGCTGAGCTTCTTGTGGATCAATGTCACTACCCTTTTCCGCATCATTTACTA

AAATAATGATCTCATTATTTCCTATTCTAGCAAAACCACCCATCAGAGCCATCGTTAACC

ATTGGTCCTTAAAGCGTATTCTCAAAATCCCTATATCTACAGCTGTAGCAATAGGGGCAT

GATTTGGTAATACGCCAATTTGACCACTATTAGTATATAAAATGATTTCTTTCACTTCTG

AATCCCAAACAATTCGATTAGGGGTCAGTACACAAAGATTTAAAGTCATTTCTTCAAATT

GCTCTCCATTTCTAAGTTCATAGCCTTCGCGGTAGCTTCATCGATATTACCTACCAAATA

AAAGGCCTGTTCAGGAAGACCATCTAATTCTCCGGAAAGGATCAATTGAAACCCTCTAAT

GGTTTCTGCTAGACCAACATATTTCCCTGGAGAACCGGTAAATACTTCTGCTACAAAAAA

AGGTTGTGATAAGAAACGCTCAATTTTTCGTGCTCTTGCTACGGTTAAACGATCCTCTTC

GGATAATTCGTCCAACCCAAGGATAGCTATAATGTCCTGAAGCTCTTTGTAACGTTGTAA

AGTTTGCTTAACTCTTTGCGCAGTTTCGTAATGTTCCTCACCAACGATCCGAGGTTGAAG

CATGGTTGACGTTGAATCTAAAGGATCTACTGCTGGATAGATCCCCTTGGCAGCTAATCC

TCTTGATAGTACGGTAGTAGCATCTAAATGTGCAAATGTCGTAGCAGGAGCAGGGTCGGT

CAAATCGTCTGCAGGTACATAAACTGCTTGAATAGAAGTTATGGACCCTTCTTTGGTAGA

AGTAATTCTTTCTTGTAAAGTACCCATTTCGGTACTCAGGGTAGGTTGATAACCGACAGC

GGAAGGCATTCTACCCAATAAGGCCGATACTTCGGATCCTGCTTGGACGAAACGGAAGAT

ATTGTCGATAAATAGAAGTACGTCTTGTTCATTAACATCTCGGAAATATTCCGCCATAGT

TAGGGCAGTCAAACCAACTCTCATACGAGCTCCAGGCGGTTCATTCATCTGGCCGTAAAC

TAGAGCCACTTTTGATTCTGCAATATTTTCTTCATTAATCACTCCAGATTCTTTCATTTC

CATGTAAAGATCATTTCCTTCACGAGTACGTTCACCCACTCCGCCAAATACGGATACGCC

CCCATGGGCTTTGGCAATATTGTTAATCAATTCCATAATGAGTACCGTTTTACCAACTCC

AGCTCCTCCAAATAGTCCGATTTTTCCCCCACGGCGATAAGGGGCTAAAAGATCTACTAC

TTTAATTCCTGTTTCAAAAATAGATAATTTTGTATCTAACTGTATAAAGGCAGGCGCAGA

TCTATGAATAGGAGATGTTGTACGAATATCTACAGGACCTAAATTATCAACAGGCTCTCC

AAGCACGTTGAAAATTCGTCCCAGAGTCGCTCCACCGACCGGAACACTTAGAGGAGCTCC

TGTGTCAATCACTTCCATTCCTCTCATCAGACCATCTGTAGCACTCATAGCTACAGCTCT

AACTCGATTATTTCCTAATAATTGCTGTACCTCACAAGTCACATTAATTGCTTGACTAAC

AGTATCTCGACCTTTAACTACCAGAGCGTTATAAATATTAGGCATCTTGCCCGGCGGAAA

GGCTACATCTAGTACCGGACCGATGATTTGGACGATACGGCCCAGGTTTTGTTTTTCAAG

CGTGGAAACCCCAGAACCAGAAGTAGTAGGATTAATTCTCATAATAATTAATAATAAACA

AAATATGTCGAAATTTTTTTTTGCGAAAATTATCGAATTCAAAATAAAGGTCCGATAGCA

CGGAGATCAGTTAATTCAATAAGAAATGTCAATAAGAAATGGGAGTTAGCACTCGATTTC

GTTGGTACCATTCAATCGAATCCAATTCAATTGTTTACTTATTCATCCACTTGCAATTTT

CAAGTTTTTGAAAATTGCAAGTGGATGAATAAGAATCTTGAGAAAGTTTTTCATTTGTCT

ATAATGATAGACAATCCCATCTATATTATAGAAAATATTCTATCGAATTCGAACCTGAAC

TCTATTTACATTAGGATTCATTATTTCTATATCATCGGTGCTTCGTATTTTTTATTTCAG

CATATCAATTTACGCTTACGCCTAGCCTATATTTTTTCTTTTCGTTTTTTTATACCCTTT

CATAGACGAATTCCGCATATTTTCACATCTAGGATTTACATATACAACATATATCACTGT

CAAGAGGGAATTTCTTATTAGTTAGGTTAGGTATTTCGATTCCAAAAAAGGT-AAAAAAA

AATTGGGTTGCGCTATATATATGAAAGAGTATACAATAATGATGTATTTGGTAAATCAAA

TACCATGGTCTAATAATCAAACATTCTGATTAGTTGATAATATTAGTATTAGTTGGGAAG

TTTGTGAAAGATTCCTGTGAAAAGTTTCATTAACGCCTAATTCGTGTCGAGTAGACCTTG

TTGTTGTCAGAATTCTTAATTCATGAGTTGTAGGGAGGGATTTATGTCACCACAAACAGA

GACTAAAGCAAGTGTTGGATTCAAAGCGGGTGTTAAAGAGTACAAATTGACTTATTATAC

TCCTGAATACGAAACCAAAGATACTGATATCTTGGCAGCATTCCGAGTAACTCCTCAACC

TGGAGTTCCGCCTGAAGAAGCAGGGGCTGCGGTAGCTGCCGAATCTTCTACTGGCACATG

GACAACTGTGTGGACCGATGGACTTACCAGCCTTGATCGTTACAAAGGGCGATGCTACCA

TATTGAGCCCGTTCCTGGAGAAGCAGATCAATATATCTGTTATGTAGCTTACCCTTTAGA

CCTTTTTGAAGAAGGTTCTGTTACTAACATGTTTACTTCCATTGTAGGTAATGTATTTGG

GTTCAAAGCCCTGCGTGCTCTACGTCTGGAAGATCTGCGAATCCCTCCTGCTTATGTTAA

AACTTTCCAAGGCCCGCCTCATGGGATCCAAGTTGAGAGAGATAAATTGAACAAGTATGG

TCGTCCCCTGTTGGGATGTACTATTAAACCAAAATTGGGGTTATCTGCTAAAAACTATGG

TAGAGCAGTTTATGAATGTCTTCGCGGTGGACTTGATTTTACCAAAGATGATGAGAACGT

GAACTCCCAGCCATTTATGCGTTGGAGAGATCGTTTCTTATTTTGTGTCGAAGCAATTTA

TAAATCACAGGCTGAAACAGGTGAAATCAAAGGGCATTACTTGAATGCTACTGCGGGTAC

ATGCGAAGAAATGATCAAAAGAGCTGTATTTGCTAGAGAATTGGGAGCTCCTATCGTAAT

GCATGACTACTTAACAGGAGGATTCACTGCAAATACTAGCTTGGCTCATTATTGCCGAGA

TAATGGACTACTTCTTCACATTCACCGTGCAATGCATGCAGTTATTGATAGACAGAAGAA

TCATGGTATACACTTCCGTGTACTAGCTAAAGGGTTACGTATGTCTGGTGGAGATCATAT

TCACTCTGGTACCGTAGTAGGTAAACTTGAAGGGGAAAGAGACATCACTTTAGGCTTTGT

TGATTTACTGCGTGATGATTTTATTGAAAAAGATCGAAGTCGCGGTATTTATTTCACTCA

AGATTGGGTCTCTCTACCAGGTGTTCTGCCCGTGGCTTCAGGGGGTATTCATGTTTGGCA

TATGCCTGCTCTGACCGAGATCTTTGGGGATGATTCCGTACTACAGTTCGGTGGAGGAAC

TTTAGGGCACCCTTGGGGTAATGCGCCAGGTGCCGTAGCTAACCGAGTAGCTCTAGAAGC

ATGTGTAAAAGCTCGTAATGAAGGACGTGATCTTGCTTCTGAGGGTAATGTAATTATCCG

TGAGGCTAGCAAATGGAGTCCTGAACTAGCTGCTGCTTGTGAGGTATGGAAAGAGATCAA

ATTTGAGTTTGCCGCAATGGATACTCTATAAGTAAGATAACAAGTAATTACCCTCCGTTC

TCTTAATTGAAAACTCGGCCCAATCTTTTACTAAAAGGATTGAGCCGAATACAAAGATTC

TATTGCATGTATTTTGGCTAAATCTATACTTCTCCAGATATACAAGATTTGAAATACAAA

ATCTAAGACTAAATCCAAATCGAAGACTCAAATGTTTCTATTGTTGTTTTGGATCCACAA

TTAATCCTATGGATCTTTAGGATTGGTATATTTTTTCTATATCCTGTAGTTTCCCTGAAT

CGAGCGAAGTATCACAAATCTTTCTACCCATCCTGTATATTGTCCTTTTCGTTCCATGTT

GGAATAGAACCTTAATTTATTACTTGTTATTAGTTAGTTATTAGACGAGATTTTACGAA-

AAAAAATTCTTTCTAGGAGAGAACAAATA-T--TTTTTTTTTGATGCGAAGACATAGGAG

AAACTACTCTTTTTCATTATATTTAGAATGAAAAGGGATTCCATCATATTCATATATAGT

GAAGTCTTACCCCGGATTCCCACAAAAGAAAATTCTTTTTCATAGTTAATAATGATAGTG

AGTGGATTCCTATGTTTATTCTGATAGGAAATGAAAATATTCAAATAAAGAATTTTGGAT

CGAATGACTATTCATCTATTGTATTTTTATGCAAACAAATAGGGGGGAAGAAAGCTCTAT

GGAAAGGTGGTGGTTTAATTCGATGGTGTTTAAGAAGGAGTTAGAACGCAGGTATGGAAT

AAATAAATCAATGGACAATCTTGGTCCTATTGAAAATACTAGTCAAAGTGAAGATACGAA

TAGAAAAGCTAAAAACATTCATAGTTGGAGGGGTCGTGACAATTCTAGTTACAGTAATGT

TGATCATTTATTCGGCATCAAAGACATTCGGAATTTCATCTCTGATGATACTTTTTTAGT

TAGAGATAGTAATGGAGACAGTTATTCTATCTATTTTGATATTGAAAATCAGATTTTTGA

AATTGACAATGATCATTCTTTTCTGAGTGAACTAGAAAGTTCTTTTTATAGTAATCGGAA

TTCTAGTTATCTGAATAATGGATCTACGAGTGAAGATCCCTACTACAATCGTTCCATGTA

TGATACTCAATATAGTTGGAATAATCACATTAATAGTTGTATTGACAGTTATCTTCAGTC

TCAAATCTATATGGATACGTCCATTGTAAGTGATAGTAGTGACAGTTACATTTCTAGGTG

TATTTTTGGTAAACATACAAATAGTAGTGAAAGCGCGAGGTCCGGTATACGAACCCACAC

GAAGAGTAGTGATTTAACTCTAAGAGAAAGGTCTAATGATCTCGATGTAACTCAAAAATA

CAGGCATTTGTGGGTTCAATGCGAAAATTGTTATGAATTAAATTATAAGAAATTTTTGAA

ATCAAAAACAAATATTTGTGAACAATGTGGATATCATTTGAAAATGAGTAGTTCAGATAG

AATCGAACTTTCGATCGATCCCGGTACTTGGCATCCTATGGATGAAGACATGGTCTCTCT

GGATCCCATTGGATTTCATTCGGAGGAGGAGCCTTATAAAGATCGTATTGATTCTTATCA

AAGAAAGACAGGATTAACTGAGGCTGTTCAAACAGGCATAGGTCAACTAAATGGTATTCC

CGTAGCAATTGGGGTTATGGATTTTCAGTTTATGGGGGGTAGTATGGGATCCGTAGTCGG

GGAGAAAATCACCCGTTTGATTGAGTACGCTACCGATCAATTTCTACCTCTTATTGTAGT

GTGCGCTTCCGGGGGGGCACGCATGCAAGAAGGAAGTTTGAGCTTGATGCAAATGGCTAA

AATATCGTCTGCTTTATATGATTATCAATCAAATAAAAAGTTATTTTATGTATCAATCCT

TACATCTCCTACTACTGGTGGGGTAACAGCTAGTTTTGGTATGTTGGGAGATATCATTAT

TGCTGAACCCAATTCCTATATTGCATTTGCGGGTAAAAGAGTAATTGAACAAACATTGAA

TAAAACAGTACCTGAAGGTTCACAAGCGGCTGAATATTTATTCCAGAAGGGCTTATTTGA

CCTAATTGTACCACGTAATCCTTTAAAAAGCGTTCTGAGTGAGTTATTTAAGCTCCACGC

TTTCTTTCCTTTGAATTAAATTCAATCAAGTAGAGCACACAAAATTCAATTAGTTTATTT

GTAGCAAACAAGTAGTTAGTTTATAAGAATCAAAGTAAATAAGAATGGAGTTTTCTTTGA

TGACCTAAGATCTAATTGTAGAAAGAATAAAAAGTTGCGGATAACTCTTTTTTTTACCTA

GAATCCCGATTACTAATTAAGAAGTCTCTATCAACAAGATAAAAGAGTGAATTCTTCCTT

TCGTGAAATTAGGCAAATAAAATGAATTTCGTCTTATGTCTTATGTATATAATCAAATAG

AGAAAAGATAGATATATAGTTTTTTATCTTTCTCTATCTCCCGAAAACCCCATTTGCACT

AAAAATTCCTGTTGGGTCGCATTCTAACGAATCTTTCGATAATCTGTAAGAAACTCTTTC

TTTATTAAAAATTCGAAGACAAGAACAAAAGACAAAGAAATGAAGAAAAATAATAAAGTG

AATTATAATACATATCTTTCATGTAGAAAGATGAATAAGTCCATTTATTTAGTTCTACAT

TCCTTGGACTTATTCTATATACTCACTTAGATATATAGATACTTATTTCTAT-ACTAAGA

ATTTGAAATTTAATTAATTAATAATAATTACAATTCTTAATTATAATTATTATAAGATAT

TTATTTTTTATAAAAAATAAATAATAGCAGGTACAAATAGTAAATCGAGGTACCCATTTT

ATGACAACTTTCAATTTCCCCTCTATTTTTGTGCCTTTAGTAGGCCTAGTATTTCCGGCA

ATTGCAATGGCTTCTTTATCTCTTCATGTTCAAAAAAACAAGATTGTTTAGATCTGATGG

GACCCGATCTCATCCGTTTTTTTTTTTCAAAACTTAGACTTGTAGCATAACACAGATATC

TATTTCGAAAAATATGGTCTAACGTGTAATTTCCGCCGAACATAAAGGAAAAAGTTCTTA

TGCCTGCAGAAAAGGATCTATGGGTAAATGAATTCTAGCTAGTTTCAAATAGATCAGGAT

CGCTGGATGGCTAAAATGTAAAGTCGGTGGATCTATAGGTATATCAATATGTATAGTGGG

CTCATATGAAGGGTATGTTATTATTTTAGATCTAACCAATTTGATGAATTACTCCTAAAG

GTTCACATCAAACTAGTGCTAGTTCACATCAAACTAGTGCTAGTTGATGAGAGTTACTTC

GGAAACAAAAA-AAAGTAAAGTCAAATTCATTTGGGGTATTCTCTCAATTCCAATAAAAT

GCAATCAGATCAAGTATGAGTTGGCGATCAGAAGATATATGGATAGAACTTATAACGGGG

TCTCGAAAACTAAGTAATTTATGCTGGGCCCTTATCCTTTTTTTAGGTTCATTAGGATTC

TTATTGGTTGGAACTTCCAGTTATCTTGGTAGAAATTTGATATCTTTTTTTCCGTCTCAG

CAAATCATTTTTTTTCCACAAGGGATCGTGATGTCTTTCTACGGGATCGCGGGTCTCTTT

ATTAGTTCCTATTTGTGGTGCACAATTTCCTGGAATGTAGGTAGTGGTTATGATCGATTC

GATAGAAAGGAAGGGATAGTGTGTATTTTTCGTTGGGGATTTCCTGGAAAAAATCGTCGC

ATATTCCTCCGATTCCTTATAAAAGATATTCAGTCCGTTAGAATAGAAGTTAAAGAGGGT

ATTTATGCTCGTCGTGTCCTTTATATGGATATCAGAGGCCAGGGGGCCATTCCCTTGACC

CGTACTGATGAGAATTTGACTCCACGAGAAATTGAAAAAAAAGCCGCCGAATTGGCCTAT

TTCTTGCGCGTACCAATTGAAGTCTTTTGAGAAATGTAAATATGGGCTGAAGAATGAATG

CTTTCTCAGCAGGAGGGCAAAATGCAAGAATCCTC--TTTTTTTTCTATAACATAACTTA

ACTGAAGTTTTGTCAGAACGTTAAGTCGAGCCAAAGCCGACATATATGGAACAACCATAA

AAGAAAACTCTTTTTGTGGCGTATACAAATCCACGCAACTCAATTCAACAACAAGTATAA

CAAATTGAACTAATAGATTCAATTCATCTCATATATCAAACGATTTCGAAAGAAAGAAAT

TTAAGTTCAATATTTGTTGGAATTGATACTTTAGATGCAGATAAATCATATCTTGTAAAT

TATTTCTTTTTTGTCAATCGACCTTTTTTTATCCTTTCTTTTGTATTCCTTAATAACCAA

CAATTGGTTTTCTTATAATGATAACTGGACAATTTCTGTCTTGTTTTGCTACTCATTTTT

TTTTGATCATCACAATATCTTTCTCTCAATTATTCTATTCCTGGCTATGGGTAATCGGTG

GAATTTTTTCGAAATATTGGATATTTTGATAGAAAAGGAATTCTTTCGTCTCAAAATCTC

AATAATATTCATTCCTTAAAGTGCTTCTTTCGTTCATTCGGAGACACTTGTTTCGAATTT

GACCAATTGAGATATCTGAAAACAATATTTTTTATTATTTCTTCATTCAAATTCGAAGTG

GCATCTTAGTCTATTTCTGTATTCTTTCTAGATTCAAACAAAATCACAAATAAAATAGAT

TCATAGGTTTGATATCTTGTATAGAACTCATTGGTGAAAGAAATATTCGATAGATCACAT

AGAGCCGACGAATGAGGTGGGTTGATTAACAATTCACAGATGAAAAAATGGCAAAAAATA

AAGCATTCACTCCTCTTTTGTATTTTGCATCTATAGTATTTTTGCCCTGGTGGATTTCTC

TCTCATTTACGAAAAGTATGGAATCTTGGGTTACTAATTGGTGGAATACTGGGCAATCTG

AAATTTTTTTGAATGATATTCAAGAAAAGAGTATTCTAGAAAAGTTCATAGAATTAGAGG

AAATCCTCTTCTTGGACGAAATGATCAAGGAATACTCGGAGACACATTTACAAAAGCTTG

GTATAGGAATCCACAAAGAAACGATCCAATTAATCAAGATACACAATGAGGATCGTATCC

ATACGATTTTGCACTTCTCGACAAATATAATCTGTTTTGTTATTCTAAGTGGTTATTCTG

TTTTTGGTAATGAAGAACTTGTTATTCTTAACTCTTGGGCTCAGGAATTCCTATATAACT

TAAGCGACACAGTAAAAGCTTTTTCAATTCTTTTATTAACGGATTTATGTATCGGATTCC

ATTCACCCCACGGTTGGGAACTAATGATTGGCTCTGTCTACAAAGATTTTGGATTTGTTC

ATAATGATCAAATTATATCTGGTCTTGTTTCCACTTTTCCAGTCATTCTCGATACTATTT

TAAAATATTGGATTTTCCGTTATTTAAATCGTGTATCTCCGTCACTTGTAGTTATTTATC

ATTCAATGAATGACTGATAAATGATCCACCGATATTAATCTAATCCAATTAGAATGTTTG

TTACTTTGTAGTTCTACATAAGCATTAAAAATCGTACTTACTCTTTAGATTTCTAACCAT

CCGGGGAATTCATCCTATATTATTCCAGTAAAATGATTCCAGTAAATAGCAGAATCGTGG

ATAGGGAACTATACTAGCAACCTACTCAATTTATTGTAGAAATTTTTGGATCAATTATTA

GACCATGCAAACTAGAAATACTTTTTCTTGGATAAAGGAACAGATTACTCGATCTATTTC

CGTATCGCTCATGATATATATAATAACTCGGACATCCATTTCAAGTGCATATCCCATTTT

TGCACAGCAGGGTTATGAAAATCCACGAGAAGCGACTGGGCGTATTGTATGTGCCAATTG

CCATTTAGCTAATAAGCCCGTGGATATTGAGGTTCCACAAGCGGTACTTCCTGATACTGT

ATTTGAAGCAGTTGTTCGAATTCCTTATGATAAGCAACTGAAACAAGTTCTTGCTAATGG

TAAGAAAGGAGGTTTGAATGTAGGGGCTGTTCTTATTTTACCGGAGGGGTTTGAATTAGC

CCCTACCGATCGTATTTCTCCCGAGATGAAAGAAAAGATAGGCAATTTGTCTTTTCAGAG

CTATCGCCCCAATAAAAAAAATATTCTTGTGATAGGGCCTGTGCCTGGTCAGAAATATAG

TGAAATCACCTTTCCTATTCTTTCCCCCGACCCTGCTACTAAGAAAGATGTTCACTTCTT

AAAATATCCTATATACGTAGGCGGGAACAGGGGAAGGGGTCAGATTTATCCCGACGGAAG

CAAGAGTAACAATACAGTTTATAATGCTACAGCAGCGGGTATAGTAAGCAAAATCATACG

AAAAGAAAAGGGGGGATATGAAATATCCATAACAGATCCATCGGATGGACGTCAAGTGGT

TGATATTATCCCTCCAGGACCAGAACTTCTTGTTTCAGAGGGTGAATCCATCCAATTTGA

TCAACCATTAACGAGTAATCCTAACGTGGGCGGATTTGGTCAGGGAGATGCAGAAATAGT

ACTTCAAGATCCATTACGTGTCCAAGGCCTTTTGTTCTTCTTGGCATCTGTTATTTTGGC

ACAAATCTTTTTGGTTCTTAAAAAGAAACAGTTTGAGAAGGTTCAATTGGCCGAAATGAA

TTTCTAGACTCGCGGATTTATCGACATCCAGTTCGTAAAAAGAACCAAATTCTTGTTGTC

GATTATGATTTTGTATGATAAAAAAA-AATGAAATTATGAAAAACCTTTTTCTTGTTTAT

ACTC-TTTTTCTACGGAATTCCTTGTACGGCATTCCGAGTCATAATAGGTAGATTTTTTT

TGAAGAAGACTATTTATTTGACTTTACCCCCTCTTTCTTTGTTTTTTTTAGCCAAATTGA

AGTGGTGCACCTATGTTACTATTGCCAGATTTCAATGTCATAAAATTGGACTAGATATTA

GCA-TAAGTAAGCGGGGAGCAAATAATTCTAGGAGGGATTATTCGTCTTCCTAGTCTTCG

ACACAAGA-AAGGGGTGTAGAAAATTCCTTTTCTTGTGTCGAAAGAGTAATGATTTTTGA

TCCTGTTCGTCAAAAATTCCTAGTCTTGGTTTCGGTTTTTCGAGATGTATCAGAACTTTT

TCGATTTATTTCGTATAATAATATAAAATAATATAATAAGTAGTGGACAAACAAAAAAAA

ACACAAAAAAAGAGGGAATTTTATTGATTAAATACAATGAACTTCTAAAAAATTTGAATT

TGGCTGAGATACTCAAATAAATAGAGTAATAGAAAGTATTTGTACGATATCTAGTCGACC

AAAATATATATCATCCAGGAAGTTGAGTGATTCCCCCTTTCTTCTAACTTGGAAAGTACC

C-ATAGATACTGTCAAGTAACAGGTGTTCTGAATCAATCAATGAAGTTCATTTTTCAAAA

GCATCATCAGAAAAAGTTTTTTGAAACAGCAGAAAAATAAATCTACTTTGTCATTTAGAC

GAAAAAAA-GACTCTGATTCTTAAGAACCCAACGGGCCTTTTCCCCTCGAATCAGACAAA

CAAAGAAGGGAATCCCGTTGAGTTCTTACGCTTTCATGTCTACAACTCAATTCATCCGAT

TACTACAGGGATGAACCCAATCCGGAATATGAACCATAAAAGAAAATACCTATTAAACCA

ATCACAAGAATACCAGTTACAGTACCTATTATCCAAAGAGGAATCCTTCCAGTAGTATCG

GCCATTTACTCTACTTCCCTCCAATTTCATCAAGTGGTCATGCTACAGACATAAACAGTC

ATGGATAATTATGAGATGAGATCCTTCCGAATGGGCTAAGAGAATGCCTAGAATTCTTAT

TTATTTTCTTTCGTTTTCCTAATTGAAGAAATAATTGGAAAATAAAACAGCAAGTACAAA

AATGAGTAATAACCCCCAGTAGAGACTGGTACGATTCAATTCAACATTTTGTTCGTTCGG

GTTTGATTGTGTCATAGCTCTATAATTCGGATTAGGTTTATCGTTGGATGAACTGCATTG

CTGATATTGACCCCAAAAAAAAGACGGTAGGTACAGCTAGGCCGTGAACAGCCAACCATC

GTACTGTAAAAATTGGATAGGTTCGATCTATAGTCATTAGATTAGGGCCTCCTAAAACGA

TCTACTAAATTCATCGAGTTGTTCCAAAGGATCAAAACGGCCAGTTATTAATGGAATTCC

TTGTCGGCTCTCTGTAAAATATTCGTTTGGCCGAGGGCTTCCAAATACATCGTAAGCTAA

ACCGGTACTGACGAATAACCAACCCGCAATGAATAGGGAAGGTATAGTAATGCTATGAAT

GACCCAGTATCGAATACTGGTAATAATATCAGCAAAAGAACGTTCTCCTGTGCTTCCAGA

CATGCTCAGCTCCACATATTCTTGTACAGTCAAAGGGGATCGATTCCGTAAAAGATGAGA

TCAGTAAAAGGCAATCACTGAAATTGCATCCTTGTAGGATCGTCAATATTGTACCGAGGG

CGTCTTTAGAGTATACCGAATCAGTATAACTATCCTTCTTCTGACACAGCAACGCAATTT

GAATCAGTATCGAATCGAAAGGAAGCTTCCTTTCCTTTACCCGTTGATGTAAAATGATGC

TCTATATTAATAGAAAATTCTTACAATGAAAGAGATTATCATATTCCCACAATTTAAGTA

GATGCGCGAGATCTAGAAATTTCCTTTTCGTAGTTGTAGAAGCAGTTTTTTTTGTTGGAA

TCCTTTTTTTAATTGGTTAATCGTCCAGTAACAAATAAGAGTAGTAGAGCGTATTCGATG

AAAGAAAGCGAAGAAAGAATAAAATAATTGGAATCCATAGTTGTGATGCATTGTTGTATT

GGATCGAGATACAAATCTTGATCTAGCTACAAGGATGAGGCTTTATTTAAAAATATGGAA

AGCCAAATTGTAAAAACTAAAAACGATAATAGAAATTACTAGTTTTAGAATCTAATTGGA

TTTTTTTCTAGTGATCCATGTGATACCTTTTTTCTTCACATTCATTCAAGATATTATGGG

AATGAACCTATTACTGAATTTAATGAATTAAACTTAATTAAGGTAAAAAAAGTTTTATAA

GGTCACTGTTCACTCTAAAATAGAAAATGAATTCGATACAATTCAAAAATCGATTTTTGA

ATTGTATTCCATAAAAATTTTATTCACAAATAAAAATTCAAAAAGAGTTTCA-TTTTTGA

GTGAAGTTACACAATCCAGTTCGTATTATTAGTTTATGCTCAATGAATCGGTTGATAGGA

ATCGCGAGATGGATAAATGTTACAAATGATGAATCAATTTCGTTTTATATGCGCCTGTCA

CTTTATCTTTGTTCGTGCCATATATAATGATAGATGAATCAAAAACTTTCAATTGAACTT

ATTCTTTCAATTGGTATTTTTGCGTATCCTCCTATTTTACAAAAATAGAAATTTAGGTAA

ATGCTTTCTAAACATATGTATAAAAATAACATATTTCATTTAGCTCCTTCATGCTTACTA

TAACTAGTTATTTCGGTTTTTTACTAGCGGCTTTAACTATAACTTCAGCTCTATTTATTG

GTCTGAGCAAGATACGACTTATTTGAAATTTATATTTGAAAGAAATAATTCCTAAAAGAA

ATCTTTCTGTGAGATTCGGTGTATTCTATAGTTACTTACTGCGTAAATTCTGGGTCATTG

AGATTCACGTCAATTCGGATTAATATTTAGGTATAGATATTACCTTTTTTTTTTCTCCTT

TTCAAAAAATTGAAATGATTGAAGTTTTTCTATTTGGAATCGTGTTAGGTCTAATTCCTA

TTACTTTGGCTGGATTATTCGTAACTGCATATTTACAATACAGGCGTGGTGATCAGTTGG

ACCTTTGATTAATTAACATTTCTTTTTTTGATTGACCTCCTCCTCCTTTCTTTAATCCAC

AGGAGGTCAAATTCTGATTGCTGTGCAAGTGAATGAATCTATTTCATTCTAATTCGATCT

ACGAAGAAAAAATCACGCTCTGTAGGATTTGAACCTACGACATCGGGTTTTGGAGACCCA

CGTTCTACCGAACTGAACTAAGAGCGCTTTCTTATCAGAATAAGAGAAGACTGTAAAGAA

AAGGATTATTTTTGTAACCCTAATCCATTTTCATTTTGTCTGCATATACTATATAGTTTC

AAAAAAATGAAAGATTCTGCCCAATTTGAATGGATCTCAGTTGATTCCTCGTTACTGCTC

AAAGGAGCAGTAATAGGTAGGGATGACAGGATTTGAACCCGTGACATTTTGTACCCAAAA

CAAACGCGCTACCAAGCTGCGCCACATCCCTTCAATTGTTCTACAGTGTCATTGTAGAGA

ATTCCTGTCTTGTTTTCCACATCCTTAGTTGTTCCATTGATATACACAATTTTTCTGCCC

ATTTCGTATTTTTGGTTTTAATAAGAAAAGTAAAAAACTTATTATATATATACGAAATAT

AGAACCCATTGTAAAAAAAATGAGTATTTTTCGGAAATACTCGCTACGAGGGGATCTTTT

TTTTTTCTGTTTTAAGAAAAAGAAAATCTTATGGATCATTGTACATTTCAATTTGAATTA

GGGATTCCGTGTACAACTATAAGTGGTCCTTAACTACATATCTATCTGATCATATATGCA

TTATCTTTATCTTTATGTATTACAATAAATAAAAGAAGGAGGGTTTTCAATGCGAGATCT

AAAAACATATCTCTCCGTTGCACCAGTACTAAGTACGCTATGGTTCGGGGCTTTAGCAGG

TCTATTGATAGAGATTAATCGTTTTTTCCCGGATGCGTTGACATTCCCCTTTTTTTCATT

CTAGTTATTGACATGGGAAGGAATGAAGAAGATTAGAGATACAATCAAATATCTGTGACT

AATACTAATCCCCCCTTTTTTCTCTTTTTTCCCTTTTTAGAATAAGGGAGGAAAGAGAAA

GAATAAAAGTAGATCCAACTTCTGCGAGACTCGGGTTCAAGTTCGAATTAAATGAATAAT

GAATATTAATAATAGAGGAATGGGGGTAGAGTAGAAAATGCAGATCTAGGGCAAGAATAC

AAGAACAAGATCTTTAACTGAAATACCGTACTTCAATTTTAAATATAGTTTAGAAATCCG

TTGTCTTACTTATTATTTACTATGGCTTTGATTTATTATTACTTTATTGATTTTGATCTT

TTAGAATTGGATTTCAAGTTAGTAACTTCTATTTTTTCCTTCCTTTTCTTCGTTTCGAAT

CGAAAATAAAAGAATTGAGTAAATCAAAAATCCAAAGGAGGTTCATGGCTAAGGGGAAAG

ATGCGCGAGTAACGGTGATTTTGGAATGTACCAGTTGTGTCCGAAATGGTGTTAAGAAGG

TATCAAGGGGCATTTCCAGATATATTACTCAAAAGAACCGGCACAATACGCCTAATCGAT

TAGAATTGAGAAAATTCTGTCCCTATTGTTACAAACATATGATTCATGGGGAAATAAAGA

AATAGATCGAACAGAGTATATCTTAGTCTTTCAAGGAAGGGTAAAAAATGACATTATATA

TAACATATTTAAATAGAAAAAA-AAATCCTATTTGGGGTTAAAATGAATTACAATTAAGA

AAGAAAAATAGGATTTTGGGATAAGAAATAAACTAAACAAAAAAACACAAAAAACCATGG

ATAAATCCAAGCGACCTTTTCTTAAATCCAAGCGGTCTTTTCGTAGGCGTTTGCCCCCGA

TTCAATCGGGGGATCGAATTGATTATAGAAACATGAGTTTAATTAGTCGATTTATTAGTG

AACAAGGAAAAATATTATCTAGACGAGTGAATAGATTGACCTTGAAACAACAACGATTAA

TTACTATTGCTATAAAACAAGCTCGTATTTTATCTTTGTTACCTTTTCTCAATAATGAGA

AACAATTTGAAAGAACCGAGTCGACCGCTAGAACTACTGGTCTTAGAACCAGAAATAAAT

AGGCTTATTCTTTGTTCACTTGAATTAGAATTCCAATCAGAACTCAAACACAGATTGTTG

TTTTGTTCGACAAATCCGGGAATCCAGATTTTATTATCGTGTCGTAAG-AAAAAAAAACG

AATCGGAAAAT-AAAAATTTAAATGTGTTCATTCATTTTGACTACTTTAGCATATTTTCT

CATAGTAATTTTGACTCTACCTTCCCGGAGTTCATTCTCCGGGGAACTCCGTTTAAATTA

TTCCGGTGGATTCTTTCCAATCTACTTCTTTTATGATCTCGTTGGAAATCATATAAAGAC

AATTCCTATTTGATATAGCTATTTGTGCAAGTATTTTACGATTAAGAAGCAACTGTCTCT

TGTATAGATCATGTATTAATTTACTATAACTATAAGATACTCCCTTTTCGCGAATTACTG

CGTTTATCCGAGTGATCCACAAACGACGAAAATTTCTCTTTTGCTTATCCCTATCCCGAT

GAGCCGAAACCAAAGCTCTTATTTTCTGTTGAGTAATAGTTCGAGTAAGTCTTGAATGAG

CCCCTCGAAAGCTTGATGCAAATAAACGAATTTTTGTTCTACGTCTCCGAGCTATATATC

CGCGTTTAATTCTGGTCATTGAATAAATGAAACTTTGACGAATAACTAATTGATTTCCTT

TCTTTCAGTTATTCTTTTCCCCTTTCCTGGTCTATTAATAACCAAACGGATTTTGCCAAT

GTAT--AAAAAAAAAATTCCAATGGCTTTGGCTACTATAACCTTCCCGACCACGATTTTT

TC-TTTTTTTTAGGTATTTCACTGCGAAATACGAAAGAAATAAGAAATTTTATTCTTCTA

AGTGTGAAAAATATAGTAAAAAGAAATATAAATTAAATGGATAAAGAAATAGTGGGTTCC

GTCGTTTCTATGGTTACTTCTTAAACGGTGAGGTCTTCTCTATACACCGGAGCCTTTACT

TCATTTAATCAATGTTATTGGTAACTTGTATAGTTCACACCACACTCTTTGGCTCTACCC

ATGAATTATCCAGTAATAGGTCTTTCACAATGAGATCCACCTATACAGTAACGGTATTTA

ATTAGGAAAGTTAGCTGGGTAGCTGACCCTCTTAGTCCGTTCTTGACAGAGTGGGAGCTT

CATTTTTATGTTTTTGAAATTGAAATAAGATTTCCTCCGCTTAATAGATAACCATTTGCT

ACCAATGGAGAATTGCTTCTCATCTTAAATTCAGGTGATTGGATTTGCACCAATGGAAAC

CATAAACTTCATACACAATAGAGGGATCTATTTGCTTATTTTTAGATAGTGAATGGGGTT

CTTTCTTCCATTCTATCCTATTTACTGGTACTGATCATTGATACTGGAAAGCGGTTTTGT

TGCTT-TTTTTTGTGCCAGCTCATGATCTAAACGAGTCGCACATACACCCTAGTACATGT

TCCTCGACGCTGAGGACATCCCCGAAGAGCGGGGGATTTCGTGACATTTCGAATTGGCTG

TCTTGTATTTCTAATAAGTTGTTTAATAGTTGGCATGTTGAATCATATACATAATGGGCT

GGTTTAGATTGATCCTAACCGGATGATTATGAATTATTTCTATTTAATAGAATATTAAAC

TCGTAGATAAAATCTCAAATCACGGATTTTTATAAAATCCATCTTATTTTCATTCAACTG

CTACAAGATCAACAATTCCATAAGCTTGGGCTTCTGTTGCTGACATAAAAACATCTCTTT

CCATGTCTTCAGATACAACCCATAAGGGTTTGCCCGTTCTTTGTACATAAACCTTTGTGA

GGGTTTCGCGTAGTTTCAGCAGTTCTTCCGCTTCCAGGACAAATTCTCCCGTTTGTGCCT

CATAAAAAGAACTAGCAGGTTGATGGATCATTACCCTGATGATATAACAGTTCTCTATCT

CGCGTGATGAAACGAAGAGAAAAGAAAGAAAGATAAAGAATAATAGGAAAA-AAAAAGAT

AGAATTGAACAACCGTACGGGCATTATCTTTTGTGCATTGCATACGGCTCTACAATAAAA

TTGACCCTTACCTTCCATTGAAGAAAGAGAAAAATAGAATCTATCAGACCCAGATGGATA

AATGATCAAATTGCCACCCTTCCTTTCAGAGGAGTTAAAAAATACTATGATGGCTCCGTT

GCTTTCTATTTTAAAATTGATTCTTTTTTTTGTCTTTGATTCAGCAATCCCAAAGTTTCT

TTTTGATCC---------AGGAAAAATCTTGTTTTTTTTTCGCCCTCTTTTTTTATAACA

TAAATATTGTTAAGAGCCCTTCGATGTGAAAACAAAAAAGTTTGTGACGCTGAACTGGAC

TCCCGATAGATAAGAGAAATCGGAAATACCTTTTATCTCATACTACTCTCTCGATACATA

ATCGAATCTTTTGAAAAAAAAACAAGACAAAAATTTTGCATATCGAATTCGAAGTGCCAT

GCTATTATTACTTAATATTCATATGGCGAAGGCATAGTCTTCTTTTTTCTCTCAAAT--A

AAAAAACCTCATTGGCGCCAAGCGTGAGGGAATGCTAGACGTTTGGTAATTTCTCCTCCA

ACCAAGATAAAAGATCCCATTGATGCGGCTAATCCCATGCATATTGTATGGACATCTGGT

CGCACAAATTGCATAGTATCGTAAACAGCTACTCCAGGTATTACCCATCCGCCAGGAGAG

TTTATAAACAAATACAGATCTTTGGTATCATCCTCGATACTGAGATATACCATAAGACCA

ATAAGTTGATTCGAGAGCTCGCTATCAACTTCTTGGCCTAAAAAAAGTAATCTTTCTCGA

TAAAGTCGGTTGATTAGGGTAAAATTGTATCCCTTAGGAACCGTACATGCACCTTTTGAC

GCATACGGTTCAAAAAATAATTGCGAAAAAAAAAAAGAATCAATGTATAGATTCAAGTCC

TCTTTCTTTGTTCCTATTCTTTTTTCATAGCAGGTTTTTTCTGACTTCTAATGAAAGGAC

TTTTTCTTCGATTTTTCAATAAAGACGAATTTGAACTTCTTTCTTCCTTATAATAGAAAA

AAAGTCACTAAACTTATCGAATTAACTTCTCATTGATGTATTGTTTCATCGAGATTCAAT

CCAAATCACGATGGTATTTTCTTGTTCCTGAATGGGTCTCTTTCATCTTTTTAGGTTTAT

GCTCTACTCCGGGTAAAGATCCGCCCGATTTTGATTTGCACATATAGGACAAATCTTCCC

ATTACCATTTCTTTTTGTTATGACTTTCTTTTTTTTTTCAATTCATTTCATACCTTTCAC

CAAGTATTTAGTTTGAGATTCCCTCGCTTGACAAATAGGATCTCTTTACAAATACCAAAC

AGGAATCATTTATGATACAAGTAGTAATCATAGATATATTACCAATTGGGTTTTTTCTAA

ACGGAGCCTGGATACTTCATTTTTTAGTCCAACCAAGCCAACCATAAATTATTCTAATTG

ATAATAGTAATGTGAATCCCCCCAAACAATGGATCTAATTGCGCTTCACGCTCCAAATTT

TTGATGATTCAATTTATCTTTC-TTGGGCGAAACAGAGGATATCTCGATCGGGGGAGAGA

ACGGGGAAATCCCATATGACCCAATATATCTGACAAGTCGCACTATACGTCAACCCAAGC

TGCATCTTCCTCTCCAGGACTTCGGAAAGGTACTTTTGGAACACCAATAGGCATTAATTG

AAAGAAAAAAGAACTAAGTACTATATTTTACTTTGATGTGGAAACGTAACAACATTATTT

TATTGTCTTTATAATATTGGTTTTATCGTATTTATTTTATCCATAGATTAGAAAAATTCA

TAAAGAAAGACAAAAGAAGAAATAAAGGAAAATTTTGACGAATAGGGCCTTCTAATGAGG

AATAAGGAAGGACACATTTACTGATAGAAAATGGTATCAACCACCCATTGCGTATTGGTA

CTTATCGGGTATAGAATAAATCTGCTTCTCTTTGTTCCTACGAATAGAATTGTTTCATTA

TTACCAATAGAATAGAACAAATAGTAACCCTTGTTCAGTGGATTATTTCAGAACAAGGGG

AGTCCATAGAATAGTCATAGTATAGCTTTTCCAATGCAATAAAGTTACGTAGTGTCTATT

TATCTTTGATAAAGAGGTATTTTCCATGGGTTTACCTTGGTATCGTGTTCATACCGTTGT

ATTGAATGATCCCGGTCGGTTGCTTTCCGTTCATATAATGCATACAGCTCTGGTTGCTGG

TTGGGCAGGTTCGATGGCTCTGTATGAATTAGCAGTTTTTGATCCTTCTGACCCTGTTCT

TGATCCAATGTGGAGACAGGGTATGTTTGTTATACCTTTCATGACTCGTTTAGGAATAAC

CAATTCATGGGGAGGTTGGAGTATCACAGGAGGGACTGTAACGAATCCGGGGATTTGGAG

TTACGAAGGTGTAGCTGGGGCACATATTGTGTTTTCGGGCTTATGCTTTTTGGCAGCTAT

CTGGCATTGGGTCTATTGGGATCTAGAAATATTTTGTGATGAACGTACAGGAAAACCTTC

TTTGGATTTGCCCAAGATCTTTGGAATTCATTTATTTCTCTCAGGGGTGGCTTGCTTTGG

TTTTGGTGCATTTCATGTAACAGGCTTGTATGGTCCTGGAATATGGGTGTCCGATCCTTA

TGGACTAACGGGAAAAGTACAACCTGTAAATCCGGCGTGGGGCGTGGAAGGTTTTGATCC

TTTTGTTCCGGGAGGAATAGCTTCTCATCATATTGCAGCAGGGACATTGGGCATATTAGC

GGGTCTATTCCATCTTAGCGTCCGCCCGCCACAACGTCTATACAAAGGATTACGTATGGG

AAATATTGAAACCGTACTTTCCAGTAGTATCGCGGCTGTCTTTTTTGCTGCTTTTGTTGT

TGCTGGAACTATGTGGTATGGTTCAGCAACTACCCCCATCGAATTATTTGGGCCCACTCG

TTATCAATGGGATCAGGGTTACTTCCAGCAAGAGATATACCGAAGAGTTAGTGCTGGGCT

AGCAGAAAATCAAAGTTTATCAGAAGCCTGGTCTAAAATTCCTGAAAAATTAGCTTTTTA

TGATTATATCGGCAATAATCCGGCAAAAGGAGGATTATTCAGGGCAGGTTCAATGGATAA

CGGAGATGGAATAGCGGTTGGATGGTTAGGACACCCTATCTTTAGAGATAAAGAAGGGCG

TGAACTTTTTGTACGTCGTATGCCTACTTTTTTTGAAACATTTCCAGTCGTTTTGGTAGA

CGGCGACGGAATTGTTAGAGCTGATGTTCCTTTTAGAAGGGCAGAATCGAAGTATAGTGT

TGAACAAGTAGGTGTAACTGTTGAGTTCTACGGCGGCGAACTCAATGGAGTCAGTTATAG

TGATCCTGCTACTGTGAAAAAATATGCTAGACGCGCTCAATTGGGTGAAATTTTTGAATT

AGATCGCGCTACTTTGAAATCCGATGGTGTTTTTCGTAGCAGTCCAAGGGGTTGGTTTAC

TTTTGGGCATGCTTCGTTTGCTTTGCTCTTCTTCTTCGGACACATTTGGCATGGTGCTAG

AACCTTGTTCAGAGATGTTTTTGCCGGTATTGACCCAGATTTGGATGCTCAAGTAGAGTT

TGGAGCATTCCAAAAACTTGGGGATCCAACGACAAGAAGACAGGTAGTCTGATACAAGAC

TGCTTTGGTATCTTTCGCCTCTATTTTCTTTTTTGGGGGGAATTTTACATAGAGTACCGG

AGTTGATTTGAATCACTGCTTTTTTTACTCTTGCTCTTTCTTTATCCGAGAGATGATTCC

CAAAGAAACAAAAAACAAACAGGTATGGAAGCTATAATTGTAAACCACGATCGAATCTAT

GGAAGCATTGGTTTATACATTCCTCTTAGTCTCGACTCTAGGGATCATTTTTTTCGCTAT

CTTTTTTCGAGAACCGCCTAAAGTTCCAACTAAAAAGCTGAAATGATTTTGCATTATCTC

AATTGAAGTAATGAGCCTCCCCATATTGGGAGGCTCATTACTTCAACTAGTCCCCATGTT

CCTCGAATGGATCTCTTAGTTGTTGAGAAGGTTGCCCAAAAGCGGTATATAAGGCGTACC

CAGTAAAACTGACAAGTAAACCAGATATAAAGATGGCGACTAGGGTTGCTGTTTCCATTA

TGATTATATAATTTCAAGATCCCAATGGATCTATGATAAGATCGTTTATTTACAACGGAA

TGGTATACAAAGTCAACAGATCTCAATGAATACAATAGGATTTATGGCTACACAAACTGT

TGAGAACAGTTCTAGATCTGGTCCAAGACGAACTACTGTAGGGAGTTTATTAAAACCATT

GAATTCGGAATATGGTAAAGTAGCTCCCGGGTGGGGAACGACTCCTTTGATGGGTGTCGC

AATGGCTCTATTTGCGGTATTTCTATCTATTATTTTGGAGATTTATAATTCTTCCGTTTT

ATTGGATGGAATTTCAATGAATTAAATCTATAAGAACCGCAAAGTCCTGTCTTTTGAATA

AAAAAATGAATCAGTTAGAGCTCGGATTTCCAGCCTATTCTATTTTGGTAGTTCGATCGT

GGAATTTATTTCTTTCTGTATTTCCGGAATATGAGTGTGTGACTTGTTATAATTGATCCT

ATTGATAGTACAGAGAATGGGTCTGTTATCTTGATAGAGATGTTTCTACTTCGTCGGATA

TTTATTCTAGTATCTGGAACACGGAATATATGAACTAGATTAAGAAATATTTGAACTATG

ATTCATACTTAATATTCGACCTCGTGTCTGGACTCCAAAAAAAA-TTCAAAGAATTAGAA

T-AAAAAAAATTTTTTTTTAGTCTATCTATTGATGGAATAAGTGATGATCCAACGGTTCT

TACTCAGATAATCCTTGGCTTAACTTAGTTGAATCATCGTGGTTTTAGTATGAATTTGAG

GTTTGAATCGATTCATAGGGTCTTAACAAGATAATTCCTATCAATTCAATAATAAAGAAA

ACAAAAAAAGCCACATTAGATACATTAGATACAAAAACAAATTAAAGAAATAGTTAAAGA

GATAATTCAAGAGGCCCGTAAGGATCAACATAAAGACGATTGAGCCAACTTGATATTTTG

GTATTATCGCCACAAAGAAGAGCTTTCGGATTA-TTTTTT-TTATTCTTTCGTACATTCA

GATAAGATTGAATCAGAGATTAAGAAGTTTCAAACTTTCTATTACATATCCGTTGCAACT

AGTATTTGGGTGTTTTTGCTTGAGCTGTACGAGATGAAAGTCTCATATACGGTTCTGAGA

GGGGGATTTTCACCTATCTCAATAAAGTCTATGATTGGTTCGAAGAACGTCTCGAGATTC

AGGCGATTGCGGATGATATAACTAGTAAATACGTTCCTCCCCATGTGAATATATTTTATT

GTTTAGGGGGAATTACGCTTACTTGTTTTTTAGTACAAGTAGCTACGGGGTTTGCTATGA

CTTTTTACTATCGTCCGACCGTTACAGAGGCTTTTGCCTCTGTTCAATACATAATGACGG

AAGCTAACTTTGGTTGGTTAATCCGATCAGTTCATCGATGGTCCGCAAGTATGATGGTCC

TAATGATGATCCTGCATGTTTTTCGTGTGTATCTCACGGGTGGATTTAAAAAACCTCGCG

AATTGACTTGGGTTACAGGTGTGGTTCTGGGGGTATTGACCGCATCTTTTGGCGTAACTG

GTTATTCCTTACCTCGGGACCAAATTGGTTATTGGGCGGTGAAAATTGTAACAGGTGTAC

CTGAAGCTATTCCTGTAATAGGATCGCCTTTGGTAGAATTATTGCGCGGAAGTGCTAGTG

TGGGACAATCCACTTTGACTCGTTTTTATAGTTTACACACTTTTGTATTGCCACTTCTTA

CTGCTGTATTTATGTTAATGCACTTTCCAATGATACGTAAACAAGGTATTTCTGGTCCTT

TATAGAGAAGATATATAATAGATATTTGTAATCAATCATTTATCACTTGGAGGAGGAATA

ATAGTATTTCATTGCTACAAGTATGGATTATTGAAAATAATAATCCATGTATTTGGATAT

TTCCCTTCAACCAATCATGTCAAATAAATGTAGAGTTGAGGGGAATTCTACGAAGAGAAA

ATGGATTATGGGAGTGTGTGACTTGAACTATTGATTGGTCTGTGTAGATATATGTCTGCC

ACATTGGAATTCACAACCAAATGTGTCTTTGTTCCAACCGTCGCGTAAGCCCATACAGAA

GATAGGCTGGTTCACTTGAAGAGATTCTTTTCTATGATCAGACCGAATCATGTTGTACAT

GAGCAGGCTCCGTAAGATCCAGTATAATTATAA-TAAGTGAAATGGATAAACTAAAACAG

AATCTTTATCTATTTCACTTACTTAATACTTAAAATTGAATAGTATGGAAATGCATTTAT

TTCCTCTGCATTGCTATGATCGATAATACTATCGGAGTGAAACAAGGGATTTAAAGAAAA

ACATAGGCTAGACTAGATTAGTAACAAGTAAACCTTTTGTGTG--TATCTCCAAATA-TT

TTGGAGATAAATACCAATCGTAAGGTCTGAGACGACCCAGAAAGCACTCGATCATATCAT

GATCAACTTTGTAAGCCAACTTGGGTATTGAGTATTTACTTAGAACCGAATTTTTTGCAA

TGGGTAGTTGCAATTCCGGAAAAAGAGTCAAATTTTTCTTACATTGAATCATTCATATAT

GTGTATATCATATATGTGTATATATAGGCAACATATAGATTTTTTATGGATTCATTTGGT

TCTTTTGAATCTTGCTCGAGCCGGATGATAAAAAATTATCATGTCCGGTTCCTTCGGGGG

ATGGATCTATAAGAATTCACCTATCCCAATAACAAAAAAACCTGATTTGAATGATCCTGT

ATTAAGAGCTAAATTGGCTAAAGGTATGGGTCATAATTATTACGGAGAACCCGCATGGCC

CAATGATCTTTTATATATTTTTCCAGTAGTAATTCTAGGTACTATTGCATGTAACGTAGG

CTTAGCGGTTCTAGAACCATCAATGATTGGTGAACCGGCAGATCCGTTTGCAACCCCTTT

GGAAATATTACCTGAATGGTATTTCTTTCCCGTATTTCAAATACTTCGTACAGTGCCCAA

TAAATTATTGGGTGTTCTTTTAATGGTTTCAGTACCTGCGGGATTATTAACAGTACCCTT

TTTAGAGAATGTTAATAAATTCCAAAATCCATTTCGTCGTCCAGTAGCGACAACCGTCTT

TTTGATTGGTACTGCAGTCGCTCTTTGGTTGGGTATTGGTGCAACATTACCTATTGATAA

ATCCCTAACTTTAGGTCTTTTTTAATTTGATTCAATTGTGAAATAACACGACGTGTGTAT

CTAGGGAATAGTCGCTTGAAAGCGAATTCTCCCTAGATACATCTATTCGATTCTGAATTT

CTTTCGAATATATGAATTGTGCTAAAGATTCAAAACCTATTTTCATCTTAATGAAAAAAA

AAAAAAAAAATCCAATAGATTTAAAACTTCTTTTTTGGTAAATCAATTGCGAAATGTTTT

TCTAGAATGACCAATATCTGTTTTATATCTTCTAGGCGCAAATGTTCAATTTTCATGAGA

TCTTCCGGACTGTTATTCAAAAGGTCCAATAATGTATATATATTGGACCTTTTGAGGCAA

TTATAGACCCTGGGAGAGAATTCTGATTGGTCAATAAAAATCGGTTTCAATGCTATTTTT

TTTTTGTTTTTTCTGAGTTTATCCAATTTATCGTGAAAGGTAAGAGGGGATAAAGGAACC

GTGTGTTGATTGTCCTCTAAAGGTAAGTTTTCTTCTTCCTTATGTAAAAAGGGAATAAAT

AAATCAATCAAATTCCGGCAGGCTTCATGAAGTGCTTCTTTCGGAGTTAAACTCCCATTT

GTCCATATTTCGAGAAAGAGTATCTCTTGTTTTTCATTCCCATTCCCATAAGAATGAATA

CTATGATTCGCATTTCGAACAGGCATGAATACAGCATCTATAGGATAACTTCCATCTTGA

AAGTTATGTGGCATTTTGATAAGATATCCGCGATTTCTCTTGATTTGTAATCCAATACAC

AAATCAATTGGTTCTGTCAAGCTAGCTATATGTTGTGTATTATCAACGATTTCTACATAA

GGTGGTAAGATGATATCTTGAGCAGTTACATATCCTGGACCTCTGACACAAATAGACGCG

TCACAAGTTCCATATAGATTACTTCTCAATACAATTTCTTTTAAATTCATTAAAATTTCA

TGGACCGATTCTTGAATACCCGCTATGGTAGAATATTCATGCGGGACGTTCTCAGATTTT

ACACGTGTGATACATGTTCCTTCTATTTCTCCAAGTAAAGCTCTTCGCATCGCAATGCCT

ATTGTGTCGGCTTGACCTTTCATAAGTGGAGACAGAATAAAGCGTCCATAATAAAGACGT

TTACTGTCTTCTCTTGATTCAACACACTTCCACTGTAGTGTCCGAGTAGATACTGTTACT

TTCTCTCGAACCATAGTAATATTATTTGATTTGATTGAATCATTTATTTCTCTTGTTTCT

CTTGAAATTTCTTCAATGTTAATTTCTACACACGTCTTTTTTTCGGGGGTCTGCAACCAT

TATGTGGCATAGGGGTTACATCCCGTACGAAAGTTAATAGTATACCACTTCTACGAATAG

CTCGTAATGCTGCGTCTCTTCCGAGACCGGGACCTTTTATCATGACTTCTGCTCGTTGCA

TACCTTGATCTACTACTGTACGAATAGCATTTGCTGCTGCAGTTTGAGCGGCAAAGGGTG

TCCCTCTTCTCGTACCCTTGAATCCACAAGTACCCGCTGAGGACCAAGAAACCACCTGAC

CCCGTACATCTGTAACCGTGACAATGGTATTATTGAAACTTGCTTGAACATGAATAACCC

CCTTTGGTATTCTACGTGCACTCTTACGTGAACCAATACGTCCATTTCTACGTGAACCAA

TTCTCGGTATAGCTTTTGCCATATTTTATCATCTCATAAATATGAGTCAGAGATATATGG

ATATATCCATTTCATGTCAAAACAGATTCCTTATTTGTACATCGAGTCCTTTAGTGAGTC

TGATTATCCTTGTCTTTGTTTATGTCTCGGGTTGGAACAAATTACTATAATGCGCCCCCG

CCTACGGATTAGGCGACATTTTTCACAAATTTTACGAACAGAAGCTCTTATTTTCATATT

TGTCATTCCTTATCTTAATTCTGAATCTACTTCTTGGAAGAAAATAAGTTTCTTGAAATT

TTTCATCTCGAATCATATTGAATAAAAACCACCTAATCCTTCCAATCCTTGTTGCGGAGT

CGATAAATTATACGTCCTCTGGTTGAATCATAACGACTTACTTCAATTTTGACTCTATCT

CCTGGCAGTATCCGTATAAAACTACGCCGGATCTTTCCTGAAACATAACCCAGGATCAGA

TCTTCATTATCTAACCGAACCCGGAACATACCATTGGGAAGCGATTCAGTAATTAAACCT

TCATGAATCCATTTTTGTTCTTTCATTCCAGGTAAAGCCTCCTTGAAGTATCAACTAATG

GAGGAGGAACGATATTAGACAACTCGTCCCTTTTCTTTTTTTTAGAAATAGGAAGAAGTT

TCGGATCCAATTTGGATATTAAAAGGATTACCATATATAACACAAAATTTCTCCGCCGAT

TCCTTCGAGTCGAGCCTCTCGGTCTGTCATTATACCTCGAGAAGTAGAAAGAATTACAAT

CCCCATCCCACCTAAAATTCTAGGAATTCGTTGAGAGTTAGAATAGATTCGTAGACCGGG

TCGACTGATCCGTTTTAAATTTAAAAAATTTCTATAGAGTCTTTTCCTATTCCTTCTATG

CCGCAGGTTTAAAACCAAAAAAGATTTGTTTTTTTCTCGATGTTTTCTAACGTTTTCAAT

AAAACCTTCTCGGAAAAGTATTTTAACAATATTTTCGGTAATATTAGTAGATGCGATGCG

AACCACTCTTTTTCTATCCATATCAGCATTTCGTATAGAGGTTATTATCTCAGCAATAGT

GTCCCTACCCATGGTGAACTAAAATTATGGGTGCCCCAAAATTGGATATAATCAACATGT

TTTTCTTTTTTTTTTTTACTT-ATTTGTTTTATGAATATGAATTATTAAAGGTATATGCG

TGAGACACAATCTACTAATTAATCTAGTTCTTAATCTATTTCTTTCAAATACCCACTATA

AACATATCAGTGATCTCATTTTATAATACCTCGGGAGCTAATGAAACTATTTTAGTAAAA

TGGAATTGTCTCAATTCCCGGGGGATCGCACCAAAAATTCTAGTTCCTTTTGGATTTCCT

TCTTGATCAATCACAACTGCAGCATTGTCATCATATCGTATTATCATACCGCTGTCACGT

TTAAGTTCTTTACAGGTACGAACAATTACAGCTCTGACTACTTCTGATTTTTCTAGGGGC

ATATTTGGTACTGCTTCTTTGATCACAGCAACAATAACGTCACCAATATGAGCATATCGA

CGATTGCTAGCTCCTATGATTCGAATACACATCAATTCTCGAGCCCCGCTGTTATCTGCT

ACATTTAAATGGGTCTGAGGTTGAATCATATCAATTTTTTAGTCTATTCTTCCAATGCAA

AGGATGAAG--AAAAAAAGGAATATTTTTTGTCCAAAAAAAGAAACTTTCATCCCCAAGA

TTCTTCTGTTGGTTCTACATTTTTATCCTGAAATAATGAATTGAGTTCGTATAGGCATTT

TGGATGCTGCTATTGAAATAGCCCTTCTAGCTATATTTTCGGTTACTCCACCCATTTCGT

ATAGTATTCGACCTGGTTTAACAACAGCTACCCAATATTCAGGGGATCCCTTTCCCGAAC

CCATACGTGTTTCAGCGGGTCTTACTGTAACCGGTTTGTCTGGAAATATACGGACCCATA

TTTTTCCACCACGGCGTGCATTTCGTGTCATTGCTCGTCGACCTGCTTCGATTTGTCTAG

ATGTGATCCAAGCAGGTTCAAGTGCCTGAAGAGCATATTTACCGAAACAAATATGATTAC

CTCGATAAGATATTCCCTTCATTCTTCCTCTATGTTGTTTACGGAATCTAGTTCTTTTGG

GGTTATAGTTGATGGTTGTTTCACAATTCCATCTCTACTACAGAACCGGACGTGAGAGTT

TCTTCTCATCCAGCTCCTCACGAATAAAAGGATTAAAAACATTTAATTGAAATGATTAAC

ATTTTTTGTAAAAAATTTTTTTTTTAGAACGTTCTAAAAAATCTTTATTTTGTTTTGTCC

TTTATCCAAGTTTAGCAAAAAAAAAAAAAAGTTTTCGCGGGCGAATATTTACTCTTTCAA

TCTCTATTTCATTTGTAGGGCTCGTTCGTGACTTCTCCCAATAGATGAATTAATCTCCGG

TTCATTTCGCCATCCCGACTAGTGAATCATTAAGATTCATTTTTTCAATAAAATCTTTTG

CATGCACAGGTTCCATCGTTCCCATCGCTTCGTACTTAATGATTAGGTCCGAATTCTACA

ATGGAGCTCATAATCCAATTTGTTCCTGAGTCAATCTTCTTAGTCTTTATTGGCTCCAAG

CTCTTTATTTTTTTCTTGATTCATTTAATATTTATGAATCAATTAGTATTGATGCTTTAT

TACACTGTCTTTTATGAGATGACTCGTAGACCTTACATATTGGAATTCTATATCATTGAT

ATTCTTTTTCTCTCTTTCTCTCATCCTTCCATTTATCCACACCTTTACTTCTTTCATTTT

GTTTTACAACTTCTAATTAAAGTTTATGCAAAAAAAAAATTTCAGTTGCTACAAAGATAT

GACTGATTTATCATATCTTGACTGGTTCTTTATATCCAGATAATACGAAGTGATGAGTTG

GTTATTAGTTCATACTATGGGGCTGGTCCTTTTTTAATCCTAACCCTAAAAAACCAACGA

GTCACACACTAAGCATAGCATTTATATCAAATGGTCAATTGAATTTTTATTCAACCCTAT

AGAATTAAGAATTAGAATTGCTCATTTTGATTAACCAGAAAAAGAATGAACGAGTTTCTA

TTTTTTTTTCTATCAATGGATAGAAGGGAAAGACAAGTAAAGGTTTCTTATTCTTCGTCT

ATAAATATCCAAATTTTGATACCCAAGACCCCATAGATAGTTCGAACTGTATAGGAACAA

TAATCAATTTTAGCTCGAATGGTTTGTAGGGGAACCCTACCTTCTCTGATCCATTCGACA

CGTGCAATTTCTTTTCCGTCGATACGTCCTGCAATTTGTACTTGAATTCCTTTTGTATCT

GCTTGTTCAGTTAATTCAATAGCTTTTTTCATTGCTTTTCGAAATGAAACTCTATTCTTT

AATTGTCCGGCTATAAATTCTGCAAGAATATTAGGGTTTCCATAAGGTTTTGCAATTCTT

GTGATAGCAACGTTAAGTTTTTGGTTCACATAATTAAATTCTTTTTCTAGATTAATCTGT

AATTCTTGGATTCCTCGCGTTCTATTTTCTATTAATAACTTTGGGAATCCCATAAAGATT

ATGACCTGGATCAAATCAATTCTTTTTTGAATTTCTATACGTGCAATTCCCTCGACGCCG

GAGGATATTCTCATATTCTTTTGTATATAATTTTTGATAAAATCTCTTATTTTTTGATCT

TCTTGTAAACCCTCAGAATAATTTTTTGGTTGTGCAAACCAAAGGGAATGATGACCTTGG

GTTGTACCAAGTCTGAAACCAAGTGGATTTATTTTTTGTCCCATACTCCCCCAATACTAT

ACATATCATGATATACCATAGTTGTATACTTATTTTTCCATCTAGGTTTTTTTAACGAAT

ATATCTGTCCATATTCATCATGTAAAGATATATCTTTCACTCCAATAGTTATATGACAGG

TAGGCCTTTTTATCGGATAACTACGTCCTCGAGCTCGAGGTTTGAATTTCTTCACGGTAG

TACCCTCGTTAACTTCGGCCTTACTAATGACTAAATTGGCTTCGTTGGAACCCATATTGT

AACTAGCATTTGCTGCTGCAGAGTAAACCAATTTAAAAATGGGATAACATGCTCTATAGG

GCATGAGTTCTAGTATCATAAGTGTTTCCTCATAGGAGCGTCCACGAATTTGATCAATTA

CTCTTCTTGCTTTGTCAGCCGATAGAGATATATGTCGACCTAAAGCGTATACTTCTGTTT

TTTTCTTCTTTCGCATAGGGTTTACCTCCTATTAATGAATCATAAGTATCTATCTATAAT

ATATATAAAAAAAAAAAAGATTAACGCCGAGATCTATTATCGCTTTTTGCATGTCCTCGG

AAATTTAAAGTAGGTACAAATTCTCCTAATTTGTGTCCTACCATAC-GATCTGTTATATA

AATAGGCAAATGTTCCTTACCATTATGAATAGCAATTGTATGGCCAATCATTGTGGGTAT

AATGGTAGATGCCCGGGACCAAGTTACTATTATTTCTTTTTCTGCTTTTTTATTAAGCTT

ATCAATTTTTCTTAATAAATGATTGACTACAAAAGGATTTTTTTTTAGTGAACGTGCCAC

AACTTCCTCCTATTTTTTTTTTGTTTTTGTAAAGACGAAGAAATAAATTCGATTTTCTCG

CCTATTTACTACGGCGACGAAGAATCAAATTATCACTATATTTATTCCTTTTTCTACTTC

TGCTTCCAAGTGCAGGATAACCCCAAGGGGTTGTGGGTTTTTTTCTACCAATTGGGGCTC

TCCCTTCACCACCCCCATGGGGATGGTCTACAGGGTTCATAACTACTCCTCTTACTACAG

GACGCTTACCTAGCCAACGCTTAGATCCGGCTCTACCCAAACTTTTCTGGTTCGCCCCAA

CATTCCCCACTTGTCCGACTGTTGCTGAGCAGTTTTTGGATATCAAACGGACCTCCCCAG

AAGGTAATTTTAATGTGGCCGATTTCCCCTCTTTTGCAATCAGTTTCGCTACAGCACCCG

CTGCTCTAACTAATTGTCCACCCTTTCCAAGTGTGATTTCTATGTTATGTATGGCCGTGC

CTAAGGGCATATCGGTTGAAGTAGATTCTTCTTTTTGATCAATCAAAACCCCTTCCCAAA

CTGTACAAGCTTCTTCCAAAGCATACGGCTTTCTGGATGTAGATGATGATATCTATACAG

ATGGATCTTTTATATATCGTACAATGAAGTACCACATGGGTGGATATCTATATGAATCCA

AATCTGCCGAATCACTCATGGTATGATCTTCTACATCCTAGGTCTTCCCGTTCCGTCATC

TGGCTTATGTTCTTCATGTAGCATTCAGACCGAATGACTCTATGAAATTACGTCGATACT

TCCACATATTATGGGTAACGTAGGAGACATCTCTATTTTTCCCCCGGGGAATCTTTAGAA

TTCCCACTGCTTAGCTTTCAATTCGCCTCTGACCATCAAATGAAATGTGAATAACCCGTC

CTCCTCTCTTTGAAAGTTTGAAAGAAGGGGCGCTTCCGGTTCTGTCGGTGCTTGAAACAA

TTTTGTCTTCTCCATATTACTATATCTCTATAGTCAATAATTTTATATGAGGAACTACTG

AACTCAATCACTTGCTGCCGTTACTCTTCAGTTTTCTGTTGAGGTCTATCCTGTAGAGGT

ACTCAAATTGGATCAGTGATCGATTTCTAGGTTTCGTCGTAAACCTAATTGGTTACTTCC

AATTACGTAAATCAATAGTTCAAACCGCACTCAAAGGTAGGGCATTTCCCATTTTTATAG

GAACTTCTGTACCAGAAACAATGGTATCTCCAATTATAGCCCCTCTGGGATGTAAAATAT

ATCTCTTCTCACCATCCCCATAGTGTATGAGACAAATGTATGCATTTCGATTAGGGTCGT

ATTCTATGGTTACGATTCTACCATATATGTCTTTTTCATTCCGTCGAAAATCGATTTTAC

GGTATAGACGCTTATGACCTCCCCCTCTATGCCTTGCGGTAATGATTCCTCTGGCATTAC

GACCTTTACCACAATGATGCTGTCCATAGATCAAATTATTTCGTGGATTGGATTTCACTT

GACTGTCTACGGTTCCATTGCGTGTGCTCGGGGTAGAAGTTTTGTATAAATGTATCGCCA

TGCTATTAAGTATTTTGATTTAAGTTCTTTTCTTTCTAAGAGGTGGAATAGAATAACCCG

GTTGAAGCGTAATGATCATACGTCTGTAATGCATTGTATGTCCCATAATAGGTCCCATTC

TTCTACCCTTTCCCGGAAGTCGATGACTATTCATAGCTATTACCTTGACACCAAAGAAGA

GTTCGACCCAATGCTTTATTTCTGTCCTAGTTGATCCTGATTCGACATTAGAAGTATATT

GATTTTTCCCCAATAACCGAATACTTTTGTCTGTAAATACTGCATATTTGATTCCATCCA

TAAATCTATTTTCTTCCCTATGAGTTCTAGTCTCAATAAGAATGCTAGTTCTTACTGTTC

ATATATTATGATATGAATATACCACACCAATTCGTTATGTATGGATGATGAGATTCCATT

GATACAGAGCCAATTCCAATAGACTTATTGGAGGGTCCCATTGGCGTGCATCCAGTAGGA

ATTGAACCTACGAATTCGCCAATTATGAGTTGGGCGCTTTAACCATTCAGCCATGGATGC

TTAGCGGGGATCCTCGTACATGGTGAATAACCAAATTCCAATTGAAATGAAATCTTTAGG

ATAAATCAATGCAATTTAGGAGGAATCAATGAAAGGACATCAATTCAAATCCTGGATTTT

CGAATTGAGAGAGATCAAGAATTCTCACTATTTCTTAGATTCATGGACCCAATTCAATTC

AGTGGGATCTTTCATTCACATTTTTTTCCACCAAGAACGTTTTCTAAAACTCTTTGACCC

CCGAATTTTGAGTATCCTACTTTCACGCAATTCACAGGGGTCAACAAGCAATCGATATTT

CACGATCAAGGGTGTAATACTCTTTGTAGTAGCGGTCCTTATATATCGTATTAACAATCG

AAATATGGTCGAAAGAAAAAATCTCTATTTGAGAGGGCTTCTTCCTATACCTATGAATTC

CATTGGACCCAGAAATGATACATTGGAAGAATCCGTTGGGTCTTCCAATATCAATAGGTT

GATTGTTTCGCTCCTGTATCTTCCAAAAGGAAAAAAGATCTCTGAGAGTTGTTTCCTGAA

TCCGAAAGAGAGTACTTGGGTTCTCCCAATAACTAAAAAGTGTAGCATGCCTGAATCTAA

CTGGGGTTCGCGGTGGTGGAGGAACTGGATCGGAAAAAAGAGGGATTCTAGTTGTAAGAT

ATCTAATGAAACCGTCGCTGGAATTGAGATCTTATTCAAAGAGAAAGATATCAAATATCT

GGAGTTTCTTTTTGTATATTATATGGATGATCCGATCCGCAAGGACCATGATTGGGAATT

GTTTGATCGTCTTTCTCTGAGGAAGAGGCGAAATAGAATCAACTTGAATTCGGGACCGCT

ATTCGAAATCTTAGTGAAACACTGGATTTCTTATCTCATGTCTGCTTTTCGTGAAAAAAT

ACCAATTGAAGTGGAGGGTTTCTTCAAACAACAAGGGGCTGGGTCAACTATTCAATCAAA

TGATATTGAGCATGTTTCCCATCTCTTCTCGAGAAACAAGTGGGCTATTTCTTTGCAAAA

TTGTGCTCAATTTCATATGTGGCAATTCCGCCAAGATCTCTTCCTTAGTTGGGGGAAGAA

TCCGCACGAATCGGATTTTTTGAGGAACGTATCGAGAGAGAATTTGATTTGGTTAGACAA

TGTGTGGTTGGTAAACAAGGATCGGTTTTTTAGAAAGGTACGGAATGTATCGTCAAATAT

TCAATATGATTCCACAAGATCTAGTTTCGTTCAAGTAACGGATTCTAGCCAACTGAAAGG

ATCTTCTGATCAATCCAGAGATCATTTGGATTCCATTAGTAATGAGGATTCGGAATATCA

CACATTGATCAATCAAAGAGAGATTCAACAACTAAAAGAAAGATCGATTCTTTGGGATCC

TTCCTTTCTTCAAACGGAAGGAACAGAGATAGAATCAGACCGATTCCCGAAATGCCTTTC

TGGATATTCCTCAATGTCCCGGCTATTCACGGAACGTGAGAAGCAGATGATTAATCATCT

GCTTCCGGAAGAAATCGAAGAATTTCTTGGGAATCCTACAAGATCCGTTCGTTCTTTTTT

CTCTGATAGATGGTCAGAACTTCATCTGGGTTCGAATCCTACTGAGAGGTCCACTAGAGA

TCAGAAATTGTTGAAGAAACAACAAGATCTTTCTTTTGTCCCTTCCAGGCGATCGGAAAA

TAAAGAAATGGTTAATATATTCAAGATAATTACGTATTTACAAAATACTGTCTCAATTCA

TCCTATTTCATCAGATCCGGGGTGTGATAGGGTTCCGAAGGATGAACCGGATATGGACAG

TTCCAATAAGATTTCATTCTTGAACAAAAATCCATTTTTTGATTTATTTCATCTATTCCA

TGACCGGAACAGGCGAGGATACACGTTACACCACGATTTTGAATCAGAAGAGAGATTTCA

AGAAATGGCAGATCTATTCACTCTATCAATAACCGAGCCGGATCTGGTGTATCATAAGGG

ATTTGCCTTTTCTATTGATTCCTACGGATTGGATCAAAAACAATTCTTGAATGAGGCCAG

GGATGAATCGAAAAAGAAATCTTTATTGGTTCTACCTCCTATTTTTTATGAAGAGAATGA

ATCTTTTTCTCGAAGGATCAGAAAAAAATGGGTCCGGATCTCCTGCGGGAATGATTTGGA

AGATCCAAAACCAAAAATAGTGGTATTTGCTAGCAACAACATAATGGAGGCAGTCAATCA

ATATAGATTGATCCGAAATCTGATTCAAATCCAATATAGTACCTATGGGTACATAAGAAA

TGTATTGCATCGATTCTTTTTAATGAATCGATCCGATCGCAACTTCGAATATGGAATTCA

AAGGGATCAAATAGGAAAGGATACTCTGAATCATAGAACTATAATGAAATATACGATCAA

CCAACATTTATCGAATTTGAAAAAGAGTCAGTTCGATCCTCTTATCTTGATTTCTCGAAC

CGAGAAATCCATGAATCGGGATCCTGATGCATATAGATACAAATGGTCCAATGGGAGCAA

GAATTTCCAGGAACATTTGGAACATTTCGTTTCTGAGCAGAAGAGCCGTTTGCAAGTAGT

GTTCGATCAATTACGTATTAATCAATATTCGATTGATTGGTCTGAGGTTATCGACAAAAA

AGATTTGTCTAAGCCACTTCGTTTCTTTTTGTCCAAGTCACTTCTTTTTTTGTCCAAGTT

GCTTTTCTTTTTGTCTAACTCACTTCCTTTTTTCTGTGTGAGTTTCGGGAATATCCCCAT

TCATAGGTCCGAGATCTACATCTATGAATTGAAAGGTCCGAATGATCAACTCTGCAATCA

GTTGTTAGAATCAATAGGTCTTCAAATTGTTCATTTGAAAAAATGGAAACCCTTCTTATT

GGATGATCATGATACTTCCCGAAAATCGAAATTCTTGATCAATGGAGGAACAATATCACC

CTTTTTGTTCAATAAGATCCCAAAGTGGATGATTGACTCATTCCATACTAGAAATAATCG

CAGGAAATCCTTTGATAACACGGATTCCTATTTCTCAATGATATCCCACAATCAAGACAA

TTGGCTGAATCCCGTGAAACCATTTCATAGAAGTTCATTGATATCTTCTTTTTATAAAGC

AAATCGACTTCGATTCTTGAATAATCCACATCACTTCTGCTTCTATTGTAACACAAGATT

CCCCTTTTCTGTGGAAAAGGCCCGTATCTATAATTATGATTTTACGTATGGACAATTCCT

CAATATCTTGTTCATTCGCAACAAAATATTTTCTTTGTGCGTCGGTAAAAAAAAACATGC

TTTTGGGGGGAGAGATACTATTTCACCAATCGAGTCACAGGTATCTAACATATTCATACC

TAACTATTTTCCACAAAGTGGTGACGAAACGTATAACTTGTACAAATCTTTCCATTTTCC

AAGTCGATACGATCCATTCGTTCGTAGAACTATTTACTCGATCGCAGACATTTCTGGAAC

ACCTCTAACAGAGGGACAAATAGTCAATTTTGAAAGAACTTATTGTCAACCTCTTTCAGA

TATGAATCTATCTGATTCAGAAGGGAAGAACTTGCATCAGTATCTCAATTTCAATTCAAA

CATGGGTTTGATTCACACTCCATGTTCTGAGAAATATTTACCATCCGAAAAGAGGAAAAA

ACGGAGTCTTTGTCTAAAGAAATGCGTTGAGAAAGGGCAGATGTATAGAAGAGATAGTGC

TTTTTCAACTCTCTCAAAATGGAATCTATTCCAAACATATATGCCATGGTTCCTTACTTC

GACAGGGTACAAATATCTAAATTTGATATTTTTAGATACTTTTTCAGACCTATTGCCGAT

ACTAAGTAGCAGTCAAAAATTTGTATCCATTTTTCATGATATTATGCATGGATCAGGTAT

ATCATGGCGAATTCTTCAGAAAAAATTGTGTCTTCCACAATGGAATCTGATAAGTGAGAT

TTCGAGTAAGTGTTTACATAGTCTTCTTCTGTCCGAAGAAATGATTCATCGAAATAATGA

GTCACCATTGATATCGACACATCTGAGATCGCCAAATGTTCGGGAGTTCCTCTATTCAAT

CCTTTTCCTTCTTCTTGTTGCTGGATATCTCGTTCGTACACATCTTCTCTTTGTTTCCCG

GGCCTCTAGTGAGTTACAGACAGAGTTCAAAAAGGTCAAATCTTTGATGATTCCATCATC

TATGATTGAGTTGCGAAAACTTCTGGATAGGTATCCTACATCTGAACCGAATTCTTTCTG

GTTAAAGAATCTCTTTCTAGTTGCTCTGGAACAATTAGGAGATTCTCTAGAAGAAATACG

GGGTTCTGCTTCTGGCGGCAACATGCTTGGTCCCGCTTATGGGGTCAAATCAATACGTTC

TAAGAAGAAATATTTGAATATCAATCTCATCGATCTCATACCAAATCCCATCAATCGAAT

CACTGTTTCGAGAAATACGAGACATCTAAGTCATACAAGTAAAGAGATCTATTCATTGAT

AAGAAAAAGAAAAAACGTGAACGGGGATTGGATTGATGATAAAATAGAATCCTGGGTCGC

GAACAGTGATTCGATTGATGATGAAGAAAGAGAATTCTTGGTTCAGTTCTCCGCCTTAAC

GACAGAAAAAAGGATTGATCAAATTCTATTGAGTCTGACTCATAGTGATCATTTATCAAA

GAATGACTCTGGTTATCAAATGATTGAACAACCGGGAGCAATTTACTTACGATACTTAGT

TGACATTCATAAAAAGTATCTATTGAATTATGAGTTCAATACATCCTGTTTAGCAGAAAG

ACGGGTATTCCTTGCTCATTATCAGACAATCACTTATTCACAAACTTCGTGTGGGACTAA

TACTTTTCATTTCCCATCTCATGGAAAACCCTTTTCGCTCCGCTTAGCCTTATCCCCCTC

TAGGGGGATTTTAGTGATAGGTTCTATAGGAACTGGACGATCCTATTTGGTCAAATACCT

AGCGACAAACTCCTATGTTCCTTTCATTACGGTATTTCTGAACAAGTTCCTGGATAACAA

GCCTAAAGGTTTTCTTATTGATGATATCAATATTGATGATAGTGACGATATTGATGATAG

TGACAATATTGATGCTAGTGACGATATTGATGCTAGTGACGATATCGATCGTGACCTTGA

TACGGAGCTGGAACTGCTAACTATGATGAATGCGCTAACTATGGATATGATGCCGGAAAT

AGACCGATTTTATATCACCCTTCAATTCGAATTAGCAAAAGCAATGTCTCCTTGCATAAT

ATGGATTCCAAACATTCATGATCTGGATGTGAATGAGTCGAATTACTTATCCCTCGGTCT

ATTAGTGAACCATCTCTCTGAAAGATGTTCCACTAGAAATATTCTTGTTATTGCTTCGAC

TCATATTCCCAAAAAAGTGGATCCCGCTCTAATAGCTCCGAATAAATTAAATACGTGCAT

TAAGATACGAAGGCTTCTTATTCCACAACAACAAAAGCACTTTTTCACTCTTTCATATAC

TAGGGGATTTCACTTGGAAAAGAAAATGTTCCATACTAATGGATTCGGGTCCATAACCAT

GGGTTCCAATGCACGAGATCTTGTAGCACTTACCAATGAGGCCCTATCGATTAGTATTAC

ACAGAAGAAATCAATTATAGACACTAATACAATTAGATCCGCTCTTCATAGACAAACTTG

GGATTTGCGATCCCAGGTAAGATCGGTTCAGGATCATGGGATCCTTTTCTATCAGATAGG

AAGGGCTGTAGCACAAAATGTACTTCTAAGTAATTGCCCCATAGATCCTATATCTATCTA

TATGAAGAAGAGATCATGTAACGAAGGGGATTCTTATTTGTCCAAATGGTACTTCGAACT

TGGAACGAGCATGAAGAAATTAACGATACTTCTTTATCTTTTGAGTTGTTCTGCCGGATC

GGTCGCTCAAGATCTTTGGTCTCTACCCGGACCCGATGAAAAAAATGGGATCACTTCTTA

TGGACTCGTTGAGAATGATTCTGATCTAGTTCATGGCCTATTAGAAGTAGAAGGCGCTTT

GGTGGGATCTTCACGGACAGAAAAAGATTGCAGTCAGTTTGATAATGATCGAGTGACATT

GCTTCTTCGGCCCGAACCGAGGAATCCCTTAGATATGATGCAAAACGGATTTTGTTCTAT

CTTTGATCAGAGATTTCTCTATGAAAAATACGAATCGGAGTTTGAAGAAGGGGAGGGAGA

AGGAGCCCTTGACCCGCAACAGATAGAGGAGGATTTATTCAATCACATAGTTTGGGCTCC

TAGAATATGGCGCCCTTGGGGCTTTCTATTTGATTGTATCGAAAGGCCCAATGAATTGGG

ATTTCCCTATTGGTCCAGGTCATTTCGGGGCAAGCGGATCATTTATGATGAAGAGGATGA

GCTTCAAGAGAATGATTCGGAGTTCTTGCAGAGTGGAACCATGCAGTACCAGACACGAGA

TAGATCTTCCAAAGAACAAGGCCTTTTTCGAATAAGCCAATTCATTTGGGACCCTGCAGA

TCCGCTCTTTTTCCTATTCAAAGATCAGCCCCCTGGCTCTGTGTTTTCACATCGAGAATT

ATTTGCAGATGAAGAGATGTCAAAGGGGCTTCTTACTTCCCAAACAGATCCTCCTACATC

TATATATAAACGCTGGTTTATCAAGAATACACAAGAAAAGCACTTCGAATTGTTGATTAA

TCGTCAGAGATGGCTTAGAACCAATAGTTCATTATCTAATGGATCTTTCCGTTCTAATAC

TCTATCCGAGAGTTATCAGTATTTATCAAATCTGTTCCTATCTAACGGAACGCTATTGGA

TCAAATGACAAAGACATTGTTGAGAAAAAGATGGCTTTTCCCGGATGAAATGAAAATTGG

ATTCATGTAACAGGAGAAAGATTTCCCATTCCTTAGCCGGAAAGATATGTGGCCATGAAA

GAGAAAGAGGGATTAAGTAGAACAGAATTGACTGGGTGGTAGAGTCGTGGAAACGCTTCT

TTCTTCCATATTTTGGACCTTAGCTCCATGGAACAATATGTTACTGCTGAAACACGGAAG

AATTGAAATCTTAGATCAAAACACTATGTATGGATGGTATGAACTGCCCAAACAAGAATT

CTTGAACAGCGAACAACCAGTTCAGATATTCACGACCAAGAAGTACTGGATTCTCTTTCG

GATAGGCCCTGAAAGGAGAAGGAAGGCTGGAATGCCAACAGGCGTCTATTATTGAATTCA

CCCGACCCGATAGTACCCATTTTGGGAACGTCCAGTGCCAAAGTCACTGAATGGGTAAGT

CGCCAATCCCTGGACTATGTAATCTGCTGGGTTACGGGCGGGCATTTTACCAGAGGTTTC

TAATCTACCCTTGTGTGATTCCTGTTGAAGCATATACTCGGGGGTGGGTGCAGGGCGGAC

GATTTTAAAGCAGACTCCCCATTCATTAGATAGAGAAGATCACCAAGATTTCGTGATTCG

CTGCCGAACTTATTCCAATTCCAAGAGCTCGGATCGAATCGGTATATACCGATTCGATCC

GAGCTCTCTTATTGAATTGCTCATTCAATGAGCATTCTCAATATTATGCCTTGAAGAGGA

CTCGAACCTCCACGCTCTTTAGCACGAGATTTTGAGTCTCGCGTGTCTACCATTTCACCA

CCAAGGCATCTTGAAAGTGAATCCTATTCCATGAATATGATATCTATCTAGTGTGATGTA

TGGAATATATGACAAGGGTGGAGTATTTCTATTGATCGGCCCGAGTTGGACATCCAATTG

CTTCGATTTGAATTATCCGGAGAATGCCTTTTGTATATATCAAAAAGATGGCCAATCAAA

CCTATTTCTCGATTCAATAGAAGCTCAAAGAGGTGATATAGGGTCCCAAATAACGAGAGA

TATGTAAAAAGCAGGTCCGATTATTACGCCTATTCCTAATCCTAAATGGAATGGAACGAC

GTAGGGATCCATATGTAAACATAGTATCTATTTAGATACGCTCGAATGACCCCTTGAGAA

TGTATATAACCCTATTCCGGTCTGGTCCGGTATGGAATGAACTTATAATCATGGAATCGA

CTCGATCATCAGATTATAAGTTCATAACCCTAGCCCATTCCCATTTTGGTCGGAACAGAT

CTACTAATTCTTTGATTCCAGTTAGTAAGAGGGATCTTGAACTAAGAAATAGACCCTAGA

AGCTAAAAAAGGGTATCCTGAGCAATTGCAATAATCGGGTTCATTGATATTCCAGGTATA

GTAGATGCTATCACACATACAATCATACTCAATTCGATGGAATTGTTTGATCTTAAAGGA

GATCTTCTATAATTTCGCACGTGAGGGGTGATTTCTTGGTTTCGTCCAGTCATTAATAAC

TTGATTATTTTTAGATAATAGTAGATAGAAACAACGCTTGTAAGGAGTCCTATTAAAACC

AAGGAATATAGGCCTGCCTGCCATCCACACCAGAATAAATAGAGTTTTCCGAAAAAACCT

GCTAGTGGAGGAAGACCTCCTAGGGATAAGAGACATAGGGCTAAAGAGAGAGCCAAAAAA

GGATCTTTCGTGTATAATCCTGCATAATCTCGAATGTTATCAGTTCCGGTACGTAGACCA

AATAATACAATGCAAGCAAAAGTTCCTAGATTCATGGAGATATAGAACAGCATATAAGTT

ATCATGCTTGCATATCCATCATTTGAGTCTCCAACAATTATTCCAATAATTACATATCCG

ATTTGACCTATGGACGAATATGCAAGCATACGTTTCATGCTTGTTTGAGTAATAGCAATG

ATATTTCCCAATATCATGCTAAGAATAGCTAGGATTTCCAGAAGAAGATGCCATTCGTTT

GATGAGAAATAAAAAGGAATATCGAAAATTCGAGTGGCTGAAGCTGAAGCAGCTACTTTC

GAAGTAACAGAAAGAAAAGCAACGACTGGAGTGGGAGAGTCAGAGTCGAAAAGAGGATTC

CTCACTTCTTTCTCTCATTCAAAACCGTGCATGAGACTTTCATCTCACACGGCTCCTAAG

TGATAAAAGAAAGAAGAACTCATCTTCTTTCTTTTTTGATTACCTTCCTCGCGTATGTAT

AAGACCGAATCCATTCGATTTCTAAAAAAGATTACTAATCCTTAACTTTTCGAGGAATCC

TTCATCAGTGGTTGTGAATGACTGATTTTTTCAATCTTTTCGACCTTGGTTCCGTAGGAG

CAAGTCAGAAAGATTGAGAAATAGAACCATCTGATTTAATTCGTTCTCAATAGCCATGAA

ATGATCATCTTAGGGTGATCCTTTTGTCGACGGATGCTCCTATTACACTCGTAGTCTCTG

AAGGATGAGAACCAACTATGTAGCATCTACATCAAGAATTCAAGTATTGTATACGTCATT

AGTCCGATCCTTTGTAGGAACTACCCGTAATAACGAACTTGCAAAATGGATCTGTTTATC

ATAAAGAGATTCGTCGTTCCTGACCCTGCTTCACCTTAATTGTTATTTGAACAAGTAAAA

GTTCTGTCTTGGTCCGAGTGGGGATAGCATTTCTCTTCTGCATGTCCATGGAGTTTTGAA

AAATCCAAACATCTCAGAGATAGATAGAGAGGTAGGAATTTCTCGAACGAACCGCACTCC

TTCGTATACGTCAGGAGTCCATTGATGAGAAGGGGCTGGGGAAAGCTTGAACCCAATTCC

TACAGTGATGAATATGAGCGCAATTGAAATTCCTGGGGAGTTATACATTTGTGTATTGAT

AAGACCATTCACTATTTCTTGAAGCTCGATCTCTCCCCCGGATGAACCATATAGCCAAGA

GAAACCATGAACCAGAATAGAAGAGCTTGCCCCACCCATGAGTAAATATTTCATAGTAGC

CTCATTAGACCGTACATCTTTCTTGGTATATCCAGATAATAGGTAGGAGCATAAACTGAA

ACATTCTGGAGCTACAAAGATAGTTATTAAATCGTTAGCACCGCATAAAAACATTCCTCC

TAGAGTAGCTGTTAATACGAATAAGAGAAACTCTGTTATAGCCATTTCTGTACATTCAAT

GTACTCTACGGATAGAGGAATACATAGAGTTGAACATAGTAAAATAAGAAATTGAAAGAT

TTCGTTGAAATTGTTCGTTTGGAAATTTCCCGAAAAGCTAATCATAGGTTCTTCTCTCCA

TCGGAACAATAGGGCCGTTATGCTCATTACTAAACTTGTTGAAGAGATGAAATATAACCA

AGGTATATCTTTTTGATCAGAGGTTGAATCGATCATCAGAAGAAGAATTAGGCCAAAAAT

TAGGATACATTCTGGGAAAATCAAACTTCCATCGAAGAGAAGCAAATGAAAGGCTTTCAT

AAAAATTCTCGTAGAATCGAGAATGAAGTTTTCATTCTGTACATGCCAGATCATGAATTA

GTAACTGCATCCAATTTCCAAAAAAAATCCCAATTGTGTCGAACTTTCCATTTTTGGAAT

GGAATAGGATCAAGATCAAACCTTATTCCATGGTATTTACATGAGGTTCCTCTTTAAGAA

AGTCCCCGAGAGGGCTTAGTTGATCCATGATTTATGTTTCATCTTTCGTTTCCTTTTCGT

TTGTTTCGAGAAATCTATCGATCAATTCCGATTCTTTCTTTTTCTCTTGATTCTTTTCCG

ATCGAGATGTATAGATCCTGTTCATGGATTAACGAAAATGTGCAAAAGCTCTATTTGCCT

CTGCCATTTTATGAGTCTCTTCCTTTTTGCGTATGGCATCGCCACTCCCTTTGGCAGCAT

CCACTAATTCGGAACTTAATTTGAAAGCCATATTTCGACCCGGACGTTTTCGGGATGCCG

CTAATAACCAACGAATGGCAAGTGCTTTTCCTTGTGTGGATCCTATTTCAATGGGAACTT

GATGAGTTGATCCACCTACACGTCTTGCTTTTACTGCTATATCGGGAGTTACTCCACGTA

TTGCTTGACGTAAAACAGATAGTGGATTTGTTTCTGTCTTTTGTTGAATCTTTTTCATGG

CTCGATAGATAATTTGATAAGCCAATGATTTTTTTCCGTGTTTCAGAATACGGTTAACCA

ACATGTTAACTAATCGATTACGATAAATTGGATCGGATTTTGCTGTTTTTTCTTCTGCAG

TACCTCGACGTGACATGAGCGTGAAAGGGGTTCAAGAATCCGTTTTCTTTTTATAAGGGC

TAAAATCACTTA-TTTTTTTTTTGCTTTTTTACCCCATATTGTAGGGTGGATCTCGAAAG

ATATGAAAGATCTCCCTCCAAGCCGTACATACGACTTTCATCGAATACGGCTTTCCGCAG

AATTCTATATGTATCTTTGAGATCGAGTATGGAATTCTGTTTACTCACTTTAAATTGAGT

ATCCGTTTCCCTCCCTTTCCTGCTAGGATTGGAAATCCTGTATTTTACATATCCATACGA

TTGAGTCCTTGGGTTTCCGAAATAGTGTAAAAAGAAGTGCTTCGAATCATTGCTATTTGA

CTCGGACCTGTTCTAAAAAAGTCGAGGTATTTCGAATTGTTTGTTGACACGGACAAAGTC

AGGGAAAACCTCTGAAATTATTTCCATATTGAACCTTGGACATATAAGAGTTCCGAATCG

AATCTCTTTAGAAAGAAGATCTTTTGTCTCATGGTAGCCTGCTCCAGTCCCCTTACGAAA

CTTTCGTTATTGGGTTAGCCATACACTTCACATGTTTCTAGCGATTCACATGGCATCATC

AAATGATACAAGTCTTGGATAAGAATCTACAACGCACTAGAACGCCCTTGTTGACGATCC

TTTACTCCGACAGCATCTAGGGTTCCTCGAACAATGTGATATCTTACACCGGGTAAATCC

TTAACCCTTCCCCCTCTTACTAAGACTGAAGAATGTTCTTGTGAATTATGGCCAATACCG

GGTATATAAGCAGTGATTTCAAATCCAGAGGTTAATCGTACTCTGGCAACTTTACGTAAG

GCAGAGTTTGGTTTTTTTGGGGTGATAGTGGAAAAGTTGACAGATAAGTCACCCTTACTG

CCACTCTACAGAACCGTACATGAGATTTTCACCTCATACGGCTCCTCGTTCAATTCTTTC

GAATTCATTGGATCCTTTTCCGCGTTCGAGAATCCCCTCCCTTCTTCCACTCTGTCCCGA

AGAGTAACTAGGACCATTTAGTCACGTTTTCATGTTCCAATTGAACACTTTCCATTTTTG

ATTATTTTCAAAGGAGAAGATTATTCTCTTTACCAAACATATGCGGATCCAATCACGATC

CTATAATAAGAACAAGAGATCTTTCTCGATCAATCCCTTTGCCCCTCATTCTTCGAGAAT

CAGAAAGATCCTTTTCAAGTTTGAATTTGTTCATTTGGAATCTGGGTTCTTCTACTTCAT

TTTTATTTAATATTTTTCCCTCTCTTTTTTTTTATATCATTCCTTAAGTCCCATAGGTTT

GATCCTGTAGAATTTGACCCATTTTCTCATTGAACGAAGGGTACGAAATCAATCAGATTG

ATTTTTCGATCAAAAGTACTATGTGAAATCTTCGGTTTTTTCCTCTTCCTCTATCCCTAT

CCCATAGGTACAGTGTTTGAATCAATAGAGAACCTTTTCTTCTGTATGAATCGATCTTAT

TCCATTCCAATTCCTTCCCGATACCTCCCAAGGAAAATATCGAATGGATCCCAAATTGAC

GGGTTAGTGTGAGCTTATCCATGCGGTTATGCACTCTTCGAATAGGAATCCGTTTTCTGA

AAGATCCTGGCTTTCGTACTTTGGTGGGTCTCCGAGATCCTTTCGATGACCTATGTTGTG

TTGAAGGGATATCTATCTAATCCGATCGATTGCGTAAAGCCCGCGATAGCAACGGAACCG

GGGAAAGTATACAGAAAAGATAGTTCTTTTCTATTATATTAGTATTTTCTATTATATTAG

ATTAGTATTAGTTAGTGATCCCGGCTTAGTGAGTCCTTTCTTCCGTGATGAACTGTTGGC

ACCAGTCCTACATTTTGTCTCTGTGGACCGAGGAGAAAAGGGGCTCGGCGTGTACATGAG

AGAAGCAAGGAGGTCAACCTCTTTCAAATATACAACATGGATTCTGGCAATGCAATGTAG

TTGGACTCTCATGTCGATCCGAATGAATCATCCTTTCCGCGGAGGTCAATCTTTGCCTGC

TAGGCAAGAGGAGAGCAAGTTACAAATTCCGTCTCGGTAGGACATGTATTTCTATTACTA

TGAAATTCATAAATGAAGTAGTTCATGGTGGGGTTACCATTATCCTTCTTGTAGTGACGA

ATCTTGTATGTGTTCTTAAGAAAAGGAATTTGTCCATTTTTCGGGGTCTCAAAGGGCGTG

GAAACACATAAGAACTCTTGAATGGAAATGGAAAAGAGATGTAACTCCAGTTCCTTCGGA

ATCGCTAGTCAATCCTATTTCCGATAGGGGCAGTTGACAATTTAATCCGATTTTGACCAT

TATTTTCATATCCGTAATAGTGCGAAAAGAAGGCCCGGCTCCAAGTTGTTCAAGAATAGT

GGCGTTGAGTTTCTCGACCCTTTGACTTAGGATTAGTCAGTTCTATTTCTCGATGGGGGC

AGGGAAGGGATATAACTCAGCGGTAGAGTGTCACCTTGACGTGGTGGAAGTCATCAGTTC

GAGCCTGATTATCCCTAAACCCAATGTGAGTTCTTCTATTTGGATTTGCTCCCCCGCCGT

GATTCAATGAGAATGGATAAGAGGCTCGTGGGATTGACGTGAGGGGGTAGGGATGGCTAT

ATTTCTGGGAGCGAACTCCGGGCGAATATGAAGCGCATGGATACAAGTTATGCCTTGGAG

TGAAAGACAATTCCGAATCCGCTTTGTCTACGAACAAGGAAGCTATAAGTAATGCAACTA

TGAATCTCATGGAGAGTTCGATCCTGGCTCAGGATGAACGCTGGCGGCATGCTTAACACA

TGCAAGTCGGACGGGAAGTGGTGTTTCCAGTGGCGGACGGGTGAGTAACGCGTAAGAACC

TGCCCTTGGGAGGGGAACAACAGCTGGAAACGGCTGCTAATACCCCGTAGGCTGAGGAGC

AAAAGGAGGAATCCGCCCGAGGAGGGGCTTGCGTCTGATTAGCTAGTTGGTGAGGCAATA

GCTTACCAAGGCAATGATCAGTAGCTGGTCCGAGAGGATGATCAGCCACACTGGGACTGA

GACACGGCCCAGACTCCTACGGGAGGCAGCAGTGGGGAATTTTCCGCAATGGGCGAAAGC

CTGACGGAGCAATGCCGCGTGGAGGTAGAAGGCCCACGGGTCGTGAACTTCTTTTCCCGG

AGAAGAAGCAATGACGGTATCT-GGGGAATAAGCATCGGCTAACTCTGTGCCAGCAGCCG

CGGTAAGACAGAGGATGCAAGCGTTATCCGGAATGATTGGGCGTAAAGCGTCTGTAGGTG

GCTTTTTAAGTCCGCCGTCAAATCCCAGGGCTCAACCCTGGACAGGCGGTGGAAACTACC

AAGCTGGAGTACGGTAGGGGCAGAGGGAATTTCCGGTGGAGCGGTGAAATGCGTAGAGAT

CGGAAAGAACACCAACGGCGAAAGCACTCTGCTGGGCCGACACTGACACTGAGAGACGAA

AGCTAGGGGAGCGAATGGGATTAGATACCCCAGTAGTCCTAGCCGTAAACGATGGATACT

AGGCGCTGTGCGTATCGACCCGTGCAGTGCTGTAGCTAACGCGTTAAGTATCCCGCCTGG

GGAGTACGTTCGCAAGAATGAAACTCAAAGGAATTGACGGGGGCCCGCACAAGCGGTGGA

GCATGTGGTTTAATTCGATGCAAAGCGAAGAACCTTACCAGGGCTTGACATGCCGCGAAT

CCTCTTGAAAGAGAGGGGTGCCTTCGGGAACGCGGACACAGGTGGTGCATGGCTGTCGTC

AGCTCGTGCCGTAAGGTGTTGGGTTAAGTCCCGCAACGAGCGCAACCCTCGTGTTTAGTT

GCCATCGTTGAGTTTGGAACCCTGAACAGACTGCCGGTGATAAGCCGGAGGAAGGTGAGG

ATGACGTCAAGTCATCATGCCCCTTATGCCCTGGGCGACACACGTGCTACAATGGCCGGG

ACAAAGGGTCGCGATCCCGCGAGGGTGAGCTAACCCCAAAAACCCGTCCTCAGTTCGGAT

TGCAGGCTGCAACTCGCCTGCATGAAGCCGGAATCGCTAGTAATCGCCGGTCAGCCATAC

GGCGGTGAATTCGTTCCCGGGCCTTGTACACACCGCCCGTCACACTATGGGAGCTGGCCA

TGCCCGAAGTCGTTACCTTAACCGCAAGGAGGGGGATGCCGAAGGCAGGGCTAGTGACTG

GAGTGAAGTCGTAACAAGGTAGCCGTACTGGAAGGTGCGGCTGGATCACCTCCTTTTCAG

GGAGAGCTAATGCTTGTTGGGTATTTTGGTTTGACACTGCTTCACACCCAAAACAAAAAG

AAGGGAGCTACGTCTAAGTTAAACTTGGAGATGGAAGTCTTCTTTCGTTTCTCGACGTTG

AAGTAAGACCAAGCTCATGAGCTTATTATCCTAGGTCGGAACAAGTTGATAGGATCCCCT

TTTTTACGTCCCCATGTCCCCCCGTGTGGCGACATGGGGGCGAAAAAAGGAAAGAGAGGG

ATGGGGTTTCTCTCGCTTTTGGCATAGCGGGCCCCCAGTGGGAGGCTCGCACGACGGGCT

ATTAGCTCAGTGGTAGAGCGCGCCCCTGATAATTGCGTCGTTGTGCCTGGGCTGTGAGGG

CTCTCAGCCACATGGATAGTTCAATGTGCTCATCGGCGCCTGACCCTGAGATGTGGATCA

TCCAAGGCACATTAGCATGGCGTACTCCTCCTGTTCGAACCGGGGTTTGAAACCAAACCT

CTCCTCAGGAGGATAGATGGGGCGATTCGGGTGAGATCCAATGTAGATCCAACTTTCGAT

TCACTCGTGGGATCCGGGCGGTCCGGGGGGGACCACCACGGCTCCTCTCTTCTCGAGAAT

CCATACATCCCTTATCAGTGTATGGACAGCTATCTCTCGAGCACAGGTTTAGGTTCGGCC

TCAATGGGAAAAGAAAATGGAGCACCTAACAACGCATCTTCACAGACCAAGAACTACGAG

ATCACCCCTTTCATTCTGGGGTGACGGAGGGATCGTACCATTCGAGCCGTTTTTTTTTCA

TGCTTTTCCCGGAGGTCTGGAGAAAGCTGCAATCAAGAGGATTTCCCTAATCCTCCCTTC

CCGAAAGGAAGAGCGTGAAATTCTTTTTCCTTTCCGCAGGGACCAGGAGATTGGATCTAG

CCGTAAGAAGAATGCT---------TAACTCACTTCTTGGTCTTCGACCCCCTCAGTCAC

TACGAACGCCCCCGATCAGTGCAATGGGATGTGTCTATTTATCTATCTCTTGACTCGAAA

TGGGAGCAGGTTTGAAAAAGGATCTTAGAGTGTCTAGGGTTGGGCCAGGAGGGTCTCTTA

ACGCCTTCTTTTTTCTTCTCATCGGAGTTATTTCACAAAGACTCGCAGGGTAAGGAAGAA

GGGGGGAACAAGCACACTTGGAGAGCGCAGTACAACGGAGAGTTGTATGCTGCGTTCGGG

AAGGATGAATCGCTCCCGAAAAGGAATCTATTGATTCTCTCCCAATTGGTTGGACCGTAG

GTGCGATGATTTACTTCACGGGCGAGGTCTCTGGTTCAAGTCCAGGATGGCCCAGCTGCG

CCAGGGAAAAGAATAGAAGAAGGATCTGACTACTTCATGCATGCTCCACTTGGCTCGGGG

GGATATAGCTCAGTTGGTAGAGCTCCGCTCTTGCAATTGGGTCGTTGCGATTACGGGTTG

GATGTCTAATTGTCCAGGCGGTAATGATAGTATCTTGTACCTGAACCGGTGGCTCACTTT

TTCTAAGTAATGGGGAAGAGGACCGAAACATGCCACTGAAAGACTCTACTGAGACAAAGA

TGGGCTGTCAAGAACGTAGAGGAGGTAGGATGGGCAGTTGGTCAGATCTAGTATGGATCA

TACATGGACGGTAGTTGGAGTCGGCGGCTCTCCCAGGGGTCCCTCATCTGAGATCCCTGG

GGAAGAGGATCAAGTTGGCCCTTGCGAACAGCTTGATGCACTATCTCCCTTCAACCCTTT

GAGCGAAATGCGGCAAAAGAAAAGGAAGGAAAATCCATGGACCGACCCCATCATCTCCAC

CCCGTAGGAACTACGAGATCACCCCAAGGGCGCCTTTGGCATCCAGGGGTCACGGACCGA

CCATAGAACCCTGTTCAATAAGTGGAACGCATTAGCTGTCTGTTCTCAGGTTGGGCAGTA

AGGGTCGGAGAAGGGCAATCACTCATTCTTAAAACCAGCGTTCTTAAGACCAAAGAGTCG

GGCGGAAAGGGGGGAAAGCTCTCCGTTCCTGGTTCTCCTGTAGCTGGAACCTCCGGAACC

ACAAGAATCCTTAGTTAGAATGGGATTCCAACTCAGCACCTTTTGAGTGAGATTTTGAGA

AGAGTTGCTCTTTGGAGAGCACAGTACGATGAAAGTTGTAAGCTGTGTTCGGGGGGGAGT

TATTGTCTATCGTCGGCCTCTATGGTAGAATCAGTCGGGGGGCCTGAGAGGCGGTGGTTT

ACCCTGCGGCGGATGTCAGCGGTTCGAGTCCGCTTATCTCCAACTCATGAACTTAGCCGA

TACAAAGCTATATGATAGCACCCAATTTTTCCGATTCGGCGGTTCGATCTATGATTTATC

ATTCATGGACGTTGATAAGATCCATCCATTTAGCAGCACCTTAGGATGGCATAGCCTTAG

AAGGGCGAGGTTCAAACGAGGAAAGGCTTACGGTGGATACCTAGGCACCCAGAGACGAGG

AAGGGCGTAGTAATCGACGAAATGCTTCGGGGAGTTGAAAATAAGCATAGATCCGGAGAT

TCCCGAATAGGGCAACCTTTCGAACTGCTGCTGAATCCATGGGCAGGCAAGAGACAACCT

GGCGAACTGAAACATCTTAGTAGCCAGAGGAAAAGAAAGCAAAAGCGATTCCCGTAGTAG

CGGCGAGCGAAATGGGAGCAGCCTAAACCGTGAAAACGGGGTTGTGGGAGAGCAATACAA

GCGTCGTGCTGCTAGGCGAAGCAGCACGAATGCTGCACCCTAGATGGCGAAAGTCCAGTA

GCCGAAAGCATCACTAGCTTACGCTCTGACCCGAGTAGCATGGGACACGTGGAATCCCGT

GTGAATCAGCAAGGACCACCTTGCAAGGCTAAATACTCCTGGGTGACCGATAGCGAAGTA

GTACCGTGAGGGAAGGGTGAAAAGAACCCCCATCGGGGAGTGAAATAGAATATGAAACCG

TAAGCTCCCAAGCAGTGGGAGGAGCCAGGTCTCTGACCGCGTGCCTGTTGAAGAATGAGC

CGGCGACTCATAGGCAGTGGCTTGGTTAAGGGAACCCACCGGAGCCGTAGCGAAAGCGAG

TCTTCATAGGGCAATTGTCACTGCTTATGGACCCGAACCTGGGTGATCTATCCATGACCA

GGATGAAGCTTGGGTGAAACTAAGTGGAGGTCCGAACCGACTGATGTTGAAGAATCAGCG

GATGAGTTGTGGTTAGGGGTGAAATGCCACTCGAACCCAGAGCTAGCTGGTTCTCCCCGA

AATGCGTTGAGGCGCAGCAGTTGACTGGACATCTAGGGGTAAAGCACTGTTTCGGTGCGG

GCCGCGAGAGCGGTACCAAATCGAGGCAAACTCTGAATACTAGATATGACCTCAAAATAA

CAGGGGTCAAGGTCGGCCAGTGAGACGATGGGGGATAAGCTTCATCGTCGAGAGGGAAAC

AGCCCAGATCACCAGCTAAGGCCCCTAAATGATCGCTCAGTGATAAAGGAGGTAGGGGTG

CAGAGACAGCCAGGAGGTTTGCCTAGAAGCAGCCACCCTTGAAAGAGTGCGTAATAGCTC

ACTGATCGAGCGCTCTTGCGCCGAAGATGAACGGGGCTAAGCGATCTGCCGAAGCTGTGG

GATGTAAAAATACATCGGTAGGGGAGCGTTCCGCCTTAGAAGGAAGCCCCCGCGCGAGCA

GTGGTGGACGAAGCGGAAGCGAGAATGTCGGCTTGAGTAACGCAAACATTGGTGAGAATC

CAATGCCCCGAAAACCTAAGGGTTCCTCCGCAAGGTTCGTCCACGGAGGGTGAGTCAGGG

CCTAAGATCAGGCCGAAAGGCGTAGTCGATGGACAACAGGTGAATATTCCTGTACTACCC

CTTGTTGGTCCCGAGGGACGGAGGAGGCTAGGTTAGCCGAAAGCTGGTTATCGGTTCAAG

AACGTAAGGTGCCCCTGCTTTTTCAGGGTAAGAAGGGGTAGAGAAAATGCCTCGAGCCAA

TGTTCGAGCACCAGGCGCTACGGCGCTGAAGTAACCCACGCCATACTCCCAGGAAAAGCT

CGAACGACCTTTAAACAAAAGGGTACCTGTACCCGAAACCGACACAGGTGGGTAGGTAGA

GAATACCTAGGGGCGCGAGACAACTCTCTCTAAGGAACTCGGCAAAATAGCCCCGTAACT

TCGGGAGAAGGGGTGCCTCCTCACAAAGGGGGTCGCAGTGACCAGGCCCGGGCGACTGTT

TACCAAAAACACAGGTCTCCGCAAAGTCGTAAGACCATGTATGGGGGCTGACGCCTGCCC

AGTGCCGGAAGGTCAAGGAAGTTGGTGACCTGATGACAGGGGAGCCGGCGACCGAAGCCC

CGGTGAACGGCGGCCGTAACTATAACGGTCCTAAGGTAGCGAAATTCCTTGTCGGGTAAG

TTCCGACCCGCACGAAAGGCGTAACGATCTGGGCACTGTCTCGGAGAGAGGCTCGGTGAA

ATAGACATGTCTGTGAAGATGCGGACTACCTGCACCTGGACAGAAAGACCCTATGAAGCT

TCACTGTTCCCTGGGATTGGGTTTGGGCCTTTCCTGCGCAGCTTAGGTGGAAGGCGAAGA

AGGCCTCCTTCCGGGGGGGCCCGAGCCATCAGTGAGATACCACTCTGGAAGAGCTAGAAT

TCTAACCTTGTGTCAGGACCTACGGGCCAAGGGACAGTCTCAGGTAGACAGTTTCTATGG

GGCGTAGGCCTCCCAAAAGGTAACGGAGGCGTGCAAAGGTTTCCTCGGGCCGGACGGAGA

TTGGCCCTCGAGTGCAAAGGCAGAAGGGAGCTTGACTGCAAGACCCACCCGTCGAGCAGG

GACGAAAGTCGGCCTTAGTGATCCGACGGTGCCGAGTGGAAGGGCCGTCGCTCAACGGAT

AAAAGTTACTCTAGGGATAACAGGCTGATCTTCCCCAAGAGCTCACATCGACGGGAAGGT

TTGGCACCTCGATGTCGGCTCTTCGCCACCTGGGGCTGTAGTATGTTCCAAGGGTTGGGC

TGTTCGCCCATTAAAGCGGTACGTGAGCTGGGTTCAGAACGTCGTGAGACAGTTCGGTCC

ATATCTGGTGTGGGCGTTAGAGCATTGAGAGGACCTTTCCCTAGTACGAGAGGACCGGGA

AGGACGCACCTCTGGTGTACCAGTTATCGTGCCCACGGTAAACGCTGGGTAGCCAAGTGC

GGAGCGGATAACTGCTGAAAGCATCTAAGTAGTAAGCCCACCCCAAGATGAGTGCTCTCC

TATTCCGACTTCCCCAGAGCTTCCGGTAGCACAGCCGAGACAGCGACGGGTTCTCTGCCC

CTGCGGGGATGGAGCGACAGAAGTTTTGAGAATTCAAGAGAAGGTCACGGCGAGACGAGC

CGTTTATCATTACGATAGGTGTCAAGTGGAAGTGCAGTGATGTATGCAGCTGAGGCATCC

TAACAGACCGGTAGACTTGAACCTTGTTCCTACATGACCTGATCAATTCGATCAGGCACT

CGCCATCTATTTTCATTGTTCAAATCTTTGACAACACGAAAAACCATTGTTCAACTCTTT

GACAACATGAAAAAACCAAAAGCTCTGCCCTCCCTCTCTATCGGATGGAAGGGCAGAGGC

CTTTGGTGTCCCCTCCAGTCAAGAATTGGGGCCTCACAATCACTAGCCAATAGGCTTTTC

TCTCATGCCTTTCTTCGTTCATGGTTCGATATTCTGGTGTCCTAGGCGTAGAGGAACCAC

ACCAATCCATCCCGAACTTGGTGGTTAAACTCTACTGCGGTGACGATACTGTAGGGGAGG

TCCTGCGGAAAAATAGCTCGACGCCAGGATGATAAAAAGCTTAACACCTCTCATTCTTAT

TCCTTTTTCAACGAAAAAAATGAAAAATCAAAAGGTCGTCTTATTCAAAACCCCAATTAT

GACATCCCTTCTCTCCCACTTCACACCTCGGAACGCACCGTTCTTATAGAGATAAACGCG

CTTTCACATCTTCTTAACCCGAAATGAAATGGCTGGGGAGGGGAGAGGAAAGGTTCCTTT

TTTTTGAGGGTACTCCCGGGAACAGATCCAGTGGAGACGGGGTGGGGCCTGTAGCTCAGA

GGATTAGAGCACGTGGCTACGAACCACGGTGTCGGGGGTTCGAATCCCTCCTCGCCCACA

ACCGGCCCAAAAGGGAAGTACCTTTCCCTCTGGGGGTAGGAAAATCATGATCGGGATAGC

GGACCAAAAGCTATGGAACTTGGGTGTGGGTCTTTGGAATGGCTTTTTCTTTTTATTTAT

TATTTATCGTGAATGATGGAATCATTACAAATAGTATG-CCCCCGGCCCATCCGCGTATT

TTTTTGTTTTACGCCCCGTAACTCTTCCTCAGCCAGGCTTGGGCAGAATAGCAGAGCAAG

TACAAGTATTAGTAGCATAACAAAAATGCCTTCCTCGTCATTAATATGTTTGCTCGCGGC

AATTGTGAACTCTCGGGAGAATGATGCAGTGCTAGTACATCTGAGAATTCTTAATTGGCT

AGTTGTAAATAGCCCCAGGGCTATGGAACAAAGGATTATCCCGGACCTACACCGAGGTAT

TGACGGTGATTTTCAAATCTCGCAGAACAGAATGTGATACGATGAGATAGAAACAAAGAC

AGGGAACAGGTTACCTACTCTTAACGGTCAAAGCGAGCCCCTTTATTCTGAATTCGTTAA

TTCAGAATGAATCAAATCTCCCCAAGTAGGATTCGAACCTACGACCAATCGGTTAACAGC

CGACCGCTCTACCACTGAGCTACTGAGGAACAACGGGAGATTAGATCTCATAGAGTTCAA

TTCCCGTTCTCAACCCATGACCAATATGAGCTCGAAGCTTCCTTCGTAACTCCCGGAACT

TCTTCGTAGTGGCTCCCTTCCATGTCTCATTTCAGAGGGAACCTCAAAGTGGCTCTATTT

CATTATATTCCATCCATATCCCAATTCCATTCATTTAATACCCCTTTGGTGTCATTGACA

TAACAGATGTCGTTTCTAGTCTATCTCTTTCTATTTCTTTTCTATATATGGAAAGTTCAA

AAATCATCATATAATAATCCAGAAATTGCAATAGAAAAGAAATAAGGGAGGTTTGTGATG

ATTTTTCAATCTTTTCTACTAGGTAATCTAGTATCCTTATGCATGAAGATAATCAATTCG

GTCGTTGTGGTCGGACTCTATTATGGATTTCTGACCACATTCTCCATAGGGCCCTCTTAT

CTCTTCCTTCTCCGAGCTCAGGTTATGGAAGAAGGAACCGAGAAGAAGGTATCAGCAACA

ACTGGTTTTATTACGGGACAGCTCATGATGTTCATATCGATCTATTATGCGCCTCTGCAT

CTAGCATTGGGTAGACCTCATACAATAACTGCCCTAGCTCTACCATATCTTTTGTTTCAT

TTCTTCTGGAACAATCACAAACACTTTTTTGATTATGGATCTAGAAATTCAATGCGTAAT

TTCAGCATTCAATGTGTATTCCTGAATAATCTCATTTTTCAATTATTCAACCATTTCATT

TTACCAAGTTCAATGTTAGCCAGATTAGTCAACATTTATATGTTTCGATGCAACAACAAG

ATGTTATTTGTAACAAGTAGTTTTGTTGGTTGGTTAATTGGTCACATTTTATTCATGAAA

TGGCTTGGATTGGTATTAGTCTGGATACGGCAAAATCATTCTATTAGATCGAATAAGTAC

ATTCGATCTAATAAGTACCTTGTGTCAGAATTGAGAAATTCTATGGCTCGGATCTTTAGT

ATTCTCTTATTTCTTACCTGTGTCTACTATTTAGGCAGAATACCGTCACCCATTCTTACT

AAGAAATGGAAAGAAACCTCAAAAATGGAAGAAAAGGTGGAAAGTGAGGAAGAAAGAGAT

GTAGAAATAGAAACAGCTTCCGAAATGAAGGGGACTAAACAGGAACAAGAGAGATCCGCC

GAAGAAGATCCTTCTCCTTCCTTTTTTTCGGAAGAAAAGGAGGATCCGAACAAAATCGAT

GAAACGGAAGAAATCCGAGTGAATGGAAAGGAAAAAACACAGGATGAATTCCACTTTCGA

TTTACAGAGACAGGCTCTAAAAATAGCCCAGTTTATGAAGATTCTTATCTGATGAATATC

AATGAAAATCACGACAATTCAAAATTCAAAATACTTATGAAAATAAAGACCTAATAAAAA

TATTGATACACAAGATAAATAGAAGAAAAGATAAGAAGATATGCGTCCACCCCCTACATA

TTTGATACCTTCTCCTACAAAGAAACTCATAACACCAACCCCATTCGTAATTCCATCAAT

TACTTGGCGATCAAAAAAATTAGTTAATTGGGCTAATCCTCTTACCGCCCCAGTTACGAA

TGTTGTATAAAAAGCATCTATATAAGCACGATTATATGACCAATTATATAGACCATTTAG

AATTTTGTCCCAAAGACTTCTCTTAGGACCTGTTTTAACAAATAAATTTATTAAGTCGAA

ATTTTTGAAAGAGGAATAAATGGGTTTATATAAAAAGGACGCTATAAATATTCCGAAATA

AGCTATACTAACTGAAAAAATAGCATCTTTCAAAAATTCATACCAATCAATCGAATCATT

CAACTTTTGATGTAAAAGGTTTATAGACGGAGTTAACCATTTGGTTAATATATCCAATTC

TTGATTGAAAGGAATTCCTAGAGATCCAACGAACAAAGTAAAGATGCCCAATACAAGTAA

AGGAAATAACATAGTATTGTCGGATTCATAAGGATAGGAATAATCCTTTTTATTCTCAAA

ATGAACAATAGTAATAAAAGGTTCTACCCTGTTTCTTACATTTTTATCACTTCGATATGT

CTTTTTTGAAAAAAAAGAAGAACTTTTACTATTATTCATTTTTAATAAACGAAAATTTTT

GTTAATTCTTTTGGAACCCCCTTTTCCCCATAGAGATATTGAATAGAAAGGGGTATTTTG

TTTGCCACTGTAATTTTGAAAATGAACGTTTAAATGCCCTTCAAAAGTAAGTAAATAGAT

CCGAAACATATAAAATGCGGTTAATCCTGCTGTGGTCCAAGCTATTATTGCGAAAATCGG

CGAATACAACCAACTATCATTAAGAATTTCATCTTTTGACCAAAAACAAGCAAGAGGTGG

AATACCACAAAGAGAAATTGTACCTAATAAAAAAGAAATTTGGGTAATGGGTACATGTTT

TGTTAAACCACCCATAAGAGCCATATTCTGACTTTTATCTGGAGAATAGCCAACAATAGT

TTCCATTGAATGAATAATAGATCCAGACCCTAAAAATAATAATGCTTTGGAATAAGCATG

AGTAATCAAATGAAATAAAGCACTTCGATAAGATCCCATTCCTAGAGCTAACATCATATA

ACCCAATTGAGACATGGTCGAATAGGCTAAACCCCTCTTAATGTCTTTTTGAGCAAGAGC

TAAAGTAGCTCCTAATAATACTGTTATTATTCCTATGAACGAGATTAAATTCATTATGTA

AGGTATAACTATAAAAAGAGGAAGAAGGCGAGCTACAAGAAAAATTCCCGCTGCTACCAT

AGTAGCAGCATGTATAAGAGCCGAAATAGGAGTAGGTCCCTCCATAGCATCAGGTAACCA

TACATGAAGGGGAAATTGTGCAGATTTCGCAACTGCACCGGTAAATAATAGAGCAGCACA

CAAAGTAACAAATGAAGAATTGACTTTATTATTATAAATCAAGTTATTTAATATTTCGAA

TAAATCCCGAAATTCGAAACTACCTGTTATCCAATAAAAACCTAAAATTCCTAATAATAA

ACCAAAATCCCCTACACGATTAGTTACAAACGCTTTTTGACAAGCATTTGCCGCAATAGG

TCGTGTGAACCAAAACCCTATTAATAGATAGGAACACATTCCAACCAATTCCCAAAAAAT

ATAAATTTGTATCAAATTTGAACTAGTAACTAATCCTAACATGGAAGTACTGAAAAAACT

CATATAAGCAAAAAATCTCAAGTATCCTTGATCATGAGCCATATAATTATCACTATAAAT

AAGAACCATAATTCCAACAGTAGTGATTAAAATTGACATAATAGAAGTAAGTGGATCGAT

CAAGTAGCCGAATTCTAAAGAAAAATCATTATTGATGGTCCAAGACCATACATATTGATA

GATACAACTACTATTTATTTGCTGAATAGACAGATTAATTGAAAAAATCATGACTATACT

TAACAATAAAATACTCGGAAAGGCCCACATACGACGAAGATTTTTTGTTGCCGTCGGAAA

AAGAAGAAGTCCGACTCCTATTAACATAGGAACTGGAAGTGGAACAAAAGGTATGATCCA

CGCATATTGATATGTCTGTTCCATAAAAAAAGTTTTTTAATTAATTGTTTCCGATTCACC

GGATCTTACCTCTTTTGAAAGAAGTCAATAAAAAAATCAAGATATGCACTAATTTAAATA

GAATTTTCTAATTTTTTATTCTTACTTATTCTGCTAAATATTCCAAATATTCAAATCAAG

AAGTTCCAATTGGTCAAATCATATGAAAGAAAAAGATTAATTACTAGTCTCTGAAAATTA

AATAATTAGCCGTATTTTTACCGAGTTTGACCAGTTAATAG-AAAAAAA-GCATATTCTT

CTTCTTTTTTCTATTTTTAGTAATATTTTATGTCATTTTCACATATCTTTAACCTATCTA

TTTTTTT-TAATTTTATAACAAAATATTTTGTAGAAAAGAAATTCCTAATGAATAGGAAA

GTACGGATTCATTTTGAATAATAGATGTCTTTCACATCCAGCTATAACAATGAGCAATCT

CTTAATTTTTATTTAAATGGCAGTTCCAAAAAAACGTACTTCTGCATCAAAAAAGCGTAT

TCGTAAAAATATTTGGAAAAGGAAAGGATATGGGACAGCGTTAAAAGCATTTTCCTTAGG

GAAATCTCTTTCTACCGGGAATTCAAAAAGTTTTTTTGTGCG--AAAAAAAAATAAGTAA

TAAAACGTTGGAATAATCTGAATCGACTTGACTCAAAAAATTGACTCATTTCGAAAATAA

AATGAATGAATTCCATTACCTATTGAGTAAATCAATATGAAATGGAATTCGCTCCGCTCT

TAGAATATGTAGAATAAGAATTCTTCTGTTTACCTTACTCAATTCAATTCA-AAAAAATA

AAAAAGACTTTCTTTTTGTTGACAAAAGAATAAACAAAAGATATTCCATTTTCTTTTTAG

TATATTTTCTCATTTCCAAGGCGGGGAGTCTTATTTTCCGCATCTCCCTATTTGTTACAA

TAATACAACTTTTTCTTTTTTCTCTATATCCACTTTTTCTCTCTATCTCAAGTTGAGGAG

AGTATAAAATACACGAAAATCTTAAGAAAACTAAGGCCCTAAACTAAAATAATGAACCTT

TAAATACCTATTTGAACTAATTTTTATTCATCCATTTGAATCTTTCCTAAAAAATATTGC

AACCAATTGAATTCTTAAATCTAGACGATTGCTTATTCATAGGCTATTATGAGTTCAAGA

CAAGCCGCTATGGTGAAATTGGTAGACACGCTGCTCTTAGGAAGCAGTGCTAGAGCATCT

CGGTTCGAGTCCGAGTGGCGGCATGCCATCTTCTAAAAAAACGAAATAGATCCTATAATG

AATTCAATTCCTGATTTCTTGTAATTAAAGAACTCCTATTTTAAATTTTTATGATATTTT

CAACCTTAGAGCATATATTAACTCATATTTCTTTTTCGATTGTTTCAATTGTAACTACAA

TTCATTTGATAACCTTTTTAGTCGATGAAATCATAAAACTCTACGATTCATCAGAAAAGG

GCATGATAATGACTTTTTTCTGTATAACAGGATTATTAGTTACTCGTTGGATTTATTCGG

GACATTTTCCACTAAGTAATTTATATGAATCATTAATCTTCCTTTCATGGAGTTTCTCCC

TTATTCATATAGTTCCGTATTTCAAAAAAAATAAAAATTTTTTAAGCGCAATAATCGGCC

CAAGTGCTATTTTTACCCAAGGCTTTGCTACTTCAGGTCTTTTAACTCAAATACACGAAT

CTGAAATATTAGTACCCGCTCTTCAATCCGAGTGGTTAATAATGCACGTAAGTATGATGA

TATTGGGCTATGCAGCCCTTTTATGTGGATCATTATTATCAGTAGCACTTCTAGTCATTG

CAGTTAGAAAAAGCAGAAAGTTTTTTTTTACAAGTAATCATTTATTAAATTTAAATGGGT

CATTTTTCTTTAGTGAAATCGAATACATGAATGAAAGAAGCAATGTTTTACAAAATACTT

CTTTTTTTTCTCCGAAGAATTATTACAGGTCGCAATTTATTCAACAATTGGATTATTGGA

GTTATCGGGTTATTAGTCTAGGATTTATCTTTTTAACCATAGGGATACTTTCTGGAGCAG

TATGGGCTAATGAAGCATGGGGATCATATTGGAGTTGGGACCCAAAGGAAACTTGGGCAT

TTATTACTTGGATCGTATTCGCGATTTATTTACATATTCGAACCAATATAAAATGGAAGG

GTATAAATTCCGCAATTGTGGCGTCTATTGGCTTTCTTATAATTTGGATATGCTATTTTG

GGGTCAATCTATTAGGAATAGGACTACATAGTTATGGTTCATTTACATTAACATCTAATT

GAATTCAAAAGGATTCAAAAAAGACTCTTACGAATAGAAAAGTGTACAGTGCATATGAGA

TAAAAAAACTTTATGTGAACTGATGAGAACCCTGTGAATCAAATATT----TGATTCACA

GGGTTCGCGCCGATTCAATTTTTTTACTTAACTTAAGAGAAGAAGAAAAAGCCTTCTTTT

TTTCATTGTACAACGAACGATT-AAAAAAAAATCATAGGATTTTTTTTTTTATTCATCAA

AACTATCTATAAAAAGAATTAGATAGAATAACTTCGACCTTGTCAACTGATAGTGAAAGA

ACAAAATCGGGGTACATACCAATACCTAGTATGGGTAAAAAGATAGAGATTGAAAGAAAT

AACTCTCGCGGTCCAGAATCAAAAAAATAAGAGTTTGGAGCATTAAATATCTTGTATCCA

TAGAACATCTGGCGTGACATAGATAATGAATAAATAGGAGTTAATATCATTCCAATTGCC

ATTACAAAAGTAATTAGTATTTTTGGCATTAAAAGGTATTTTTGACTGGTAATTAGTCCA

AAAAAGACTATTAATTCGGCAACAAAACCACTCATACCTGGTAATGCAAGGGAGGCCATC

GAAAAGCTACTGAACATCGTGAATATTTTTGGCATTGGGATAGCTATTCCACCCATTTCG

TCAAGATAAACAAGACGTATTCTATCATAAGTCGTTCCTGCCAAGAAAAAAAGTGCAGCA

CCAATAAATCCATGAGAGATTATTTGTAAAAGGGCTCCATTGAGCCCTGTATCGGTGATA

GAACCAATTCCTATAATTATGAAACCCATATGAGATACAGAGGAATAGGCTATTCTTTTT

TTTAAATTCCGTTGACCGAGAGATGTTGAAGCTGCATAAATTATTTGCATTGCGCCTACT

ATCATCAACCAAGGAGAAAATATAGAATGAGCATGTGGTAATAATTCCATATTGATCCGA

ACTAATCCATACGCTCCCATTTTTAATAAGATTCCGGCTAGAAGCATACAAGTACTATAA

TGCGCTTCTCCATGGGTATCTGGTAACCATGTATGTAGGGGTATAATCGGCAATTTGACA

GCAAAAGCAATAAAAAATCCAATATAAAATATTATTTCCAAGGCCACAGGATAGGACTGA

TTGGCTAATATTTCTAAATTTAATGTTGGTTCATTAGAACCATATAAACCGATACCCAGA

ACTCCCATTAAAAGAAAAACAGAACCCCCCGCCGTGTACAAAATAAATTTGGTAGCTGAG

TACAGACGTTTTTTTCCTCCCCACATTGATAGAAGTAGATAAACGGGAATTAATTCTAAC

TCCCACATGAGGAAAAAAAGTAAAAGGTCCCGACAAGAAAATAATCCTATTTGACCGCTG

TACATTGCTAACATCAAGAAATGAAATAGTCGAGAATCCCGAGTAACTGGCCGAGCCGCT

AAAGTAGCTAAAGTAGTGATGAATCCCGTCAGTAAAATGGGTCCTATAGAAAGGCCATCT

ATTCCTAATCTCCAATGGAAATCAAAAAAATCGATCCATTTATAATCCTCCACTAGTTGG

ATTAATGGATCGCCCGATTGGAAATGATAACAGAATGCATAAGTCGTTATAAGAAGTTCT

AAAATACATATACATATAGTATACCAACGAATTACCCTATTTCCTCTATGGGGAAGAAAG

AAAATTAAGGAACCCGCAGATATTGGCAAAATAACAATTATTGTTAACCAAGGAAAATGA

TTCGTGGTAAAGACAAGATACACTTGGGCCAGAAAAACCCGTGCTCAAAATATTTTGAGC

ACGGGTTTTGTCGGTAAAAAAATCAAATGGATTCAAGTAGAGTTTTCTGGAACGTATCAA

TAAGCTAGACCCATACTGCGAGTTGTTTCATGCCATAAATAAACTCGAACACTCAAGAAA

TCCGTTGGACAGGCGGATTCACATCTCTTACAACCAACACAGTCCTCGGTCCTTGGGGCA

GAAGCAATTTGTTTAGCTTTACATCCGTCCCAAGGTATCATTTCTAATACATCGGTGGGA

CACGCTCGGACACATTGAGTACATCCTATACATGTATCATAAATCTTTACTGAATGTGAC

ATTGGATCTATACATTTTTTTCGTCATAAATTTTCGATCTAGTAAACTTATAAATGAATC

ATATATTTAGATACCAGACGAATCAATGAGTGATCAGAATCAATCTACTTGCGGATTGGT

TTATGAGAGAGGGCCAAAATACTTTGATTTCTTATGTTTTTGCAACCACGATCCTACCTT

ACACGTAGCAAACCTGCTAATTCGAATTAATTTCAGAATAATTTCTGAATATTCGAATTT

CTATTTATATAATTAATACTATTTATTCAACAAATTGGATTGGTTAATACGAGTTGATTT

TCTGTTACGATAAATTGATGAAACAATAGCCGGTCCAATAGCTGCTTCAGCGGCTGCAAT

AGCTATAACAAAAATGGAGAAAATATCTCCTCTTAATTGACGATTATCAAAAAAACCAGA

AAATGTTACAAAATTGATATTAACTGAATTTAATATAAGTTCAAGACACATAAGGGCTCT

AACCATATTTCGACTTGTGATCAATCCGTAGATACCGATGGAAAATAAATAGGCACTCAA

AACAAGTACATGTTCAAGCATCATTAACCAACTCCTTATCAATCTCGATTCATTTCAATA

TGAACAATAATTCAAGCGATTCGATTCTAACAACAGGGAATATATTGGTAATAGATCTAC

CTAAAGCTAAAGCATTTCTATTTTAGACAGGAATTCAAATTAAAGGATTATAACATTCAT

TTCCGTTGATTTTATTTGAATGGAATTCCTAGTTTTAAAGATTTATTATTGACGAGCTAT

TGCAATTGCACCTATTAAAGCGACTAAAAGAATTATTGAAATGAGTTCAAATGGAAGAAA

AAAATCTGTTGATAAATGAATTCCAATTTGTTGGCTATTACTTATCAAATCTTGCTCTAT

AATCTGGTTTGATTTTGTAGTCCAAATAATCCCGTACCATGACGTATCTGGAATAGTAGT

AATTAGTGAAATAAAAAGACTTGTACAAACTATTGAAGTAACTCCATCCCCAACGGTCCA

AAGATGAAAATCTTTGTAATATTCTGAACCACTCATGAACATCACAGCAAAAATGATTAA

AATATTTATAGCTCCCACATAAATAAGGAGCTGCGCAGCAGCTACAAAATACGAGTTCGA

TAGAATATAGAATAAGGATATACAAAAAAGAACCAATCCCAACGAAAAGGCCGAATAAAT

TGGATTGGGAAGTAATACTACTCCTAGACTTCCTAATATAAGACCCGATCCCAGAAAGAC

TAAAAGAAAATCATGTATTGGTCCAGGTAAACCCATTTGATTTTATAGAAAAAA-ATAGA

TAAATCGAAATATTTCATGACTTTGTTGACCTGACCAGGAAAAAAGAAGTTATCTTTGAG

GATACTTCTTAATTGAATTAAATTGGAATGGGTGTGATGTGGATTGATGTAGATACAGTT

ATGGGACTACTCTTATTCTTTATCCGAAAGAGGAGTTTGAAACTCTCTATTTTGAAATCA

TTTGAATCAAGGATTTTTTCCATTTTTTATTTGAGGTGAATTCGAAATTGTTCGAATTGT

GTAATCGTCAATTATTGACACCGGTAACCGACCTAAAGCAATTTGATTATAATTCAATTC

GTGACGATTATAAGTAGAAAGTTCATATTCTTCAGTCATTGATAAACAATTTGTTGGACA

ATACTCAACGCAATTACCACAAAATATACAGATTCCAAAATCAATACTGTAATTAAGCAA

TCGTTTCTTTCGAATATCCGTTTCCAATTTCCAATCAACAACGGGTAAATCTATAGGACA

TACACGAACACATACTTCACAAGCAATGCATTTATCAAATTCAAAGTGGATTCGTCCTCG

GAAACGTTCCGATGTGATCAATTTTTCGTAGGGGTATTGAATAGTTACAGGTAAACGATT

CACATGGGACAAGGTAATCATGAAACCTTGACCAATGTACCTTGCAGCTCGTATTGTTTG

TTGACCATAATTCATGAACTCAGTTACCATAGGAAACATATCGTAAATATCTATAAATAA

TTTTATACTTGTTTCTTTCTCTTGTTTGAGACAAGTTGTGAATATAGAATATTCTATTCC

TTTACAGTGAAAGAAGTTGGAATGAAGTTGTTAATAATAGATTACCTAGAGAAATAGGTA

AAAGAAATTTCCATCCAAGATTTAATAGTTGGTCCATTCTCAGTCTCGGTAAAGTCCATC

TTGTTGCAATAGAAATGAACAAGAACAAATAAGTTTTAGCTAATGTAATAAAGATACCGA

TTATTGTTCCAAAAACTTTACCTCTTTCAAAAAGCTCAGGAACGAATATGTACGGAATAG

AAAGATTCCAACCCCCCAAGTAAAGAACTGTTACAAATAATGAAGAAACTAGTAGATTCA

GATACGAAGCAACATAAAATAAACCAAATTTGATACCTGAATATTCGGTTTGATAACCTG

CTACTAATTCCTCTTCTGCTTCTGGTAAATCAAAAGGTAATCTCTCACACTCGGCTAGAG

AAGAAATTAGAAAAACGATAAACCCTATAGGTTGACGCCACAAATTCCACCCCCAAAAAC

CATATTTTGACTGCGCTTCAACTATATCAACTGTACTTGAACTGTTAGATAATCATAGTC

GATGATAACATCACTGTTCCCATCGCTATTACAGAACCGTACATGAGATTTTCACCTCAT

ACGGCTCCTCAGAGGTCGCAAATAAATCTAAGTACCTTTCGACATTATTTATCTTGATAT

GTTTATAGGATAGATAGAGATTCAAACTCTTATCCTAAGGTAGACCAATGGAATTCTGTC

TGCTATTTCTAAGAAATATATATTTCTAATAAATAAAAGTGCTTCTGAATTGATCTCATC

TTTTAAGAATTTTCATTTTTCTTTGTTGATTAATAACTTTATCCTTGAATAAAAAAAGAG

TTTTTTTGGAGGAATATCACATAGCTATTTCAACCTATCATTTTCCATTACGAAAAAGAA

TTAGACATTGCATTAGTTCATGCATCATGACAAGAATTCTATCTCTCTAAATAGGTATAT

AGGAAAGAAATAATAAAAAAAAATCTCTTTTTTGCAATTCTATTCTTTCGAGTTTATTTC

GTTCCTATTCTCCTTTCTCAAAAAAGGGGGACATTACCAAAGTAAAAGATTACTTCGTTC

TTGATAGTTATTTACTTAATCAGTGGATAGGAACATACTCTGGATCGAAATCATGGGGAG

TACTTCTTAATCGTTTCTACCAACTTAAAGCCCCAATTTGAATTCCTTTTAGGTACATCC

TGTTGGATAATTTACAGAATCTCCATTACTAATCCTTTGCGTATCTTGGTCTTCCTAACC

ATCCACTCATTTTTGTTAACCTTCCATTATGGTAATACATCTATGTTAATAGATAGTAAA

AACTCCATACAGTTGATCTTTTGAACCCGTTTCAAGCCATGATGCCTAATCAACCAATCT

TGGGGTAAACAGTCTCGACTGCTTTGCTTATATTTACTTTCATTTCATTCTTGTACATAG

GAAATGAGATTCAATCCCTTTAACTGCAAAT-AAAAAGCCGTTTTATTTCACTCATATAA

CTATCTGGTTTAGTTCATCAACCCGAATTCTGAATAAAAAAAAATATATATATTAAACTC

ATTTAACTTTCTTACTAGAAAGAAAAGAAATAGGGGAAATTTTATGTCTCACCGAATCAC

ACGTAGAGATATTGATAATACACATAGAGTTAATGGTATTTCATAACTAATTGATTGAGC

AGCAGCTCTTAAACCACCTAAAAAGGAATATTTATTATTTGATCCATATCCCGACATAAG

AAGTCCAACGGGAGCAAGACTTGAAACAGCGATCCATAAAAAAACACCAATACTAAGATC

GGCTAGAATAAGGCGATAACCAAAAGGAATTACTGAATAACTTAGTAAAATGGATATGAC

TGCTATGGATGGTCCGATACTGAATAAACGAGTATCCCCTCTAGATGGAAAAAGATTCTC

TTTGAAAAGTAGTTTTACCCCATCTGCTAGAGCTTGAAGAATTCCCAAGGGGCCGGCGTA

TTCAGGTCCAATACGTTGTTGTATCCCTGCAGATATTTCTCTTTCTAACCAAACAATTAC

TAGTACACCTAGTGTGATTCCTAATACAAGAGTCAAAATAGGGACAAGCACCCATATGAT

CCCATAGACTTCTTTTAAGAATTCCAATCTGGAAAACGAATGGATGGCTTGTAGTTCTGT

TGTATTATCAATTATCATTTCAACGATCAACTTCTCCCATAATGATATCTATACTACCTA

GTATCGTCATAATATCAGCCAATTTCATTCTTTTAACTAACTGAGGCAGAATTTGCAAGT

TGATAAAACCCGGTGGGCGAATTTTCCATCTCCAAGGAAAAACACTCTGATCTCCTATCA

GAAAAATTCCCAATTCTCCTTTTGGTGCTTCGACTCTTACATAAAGTTCTTGTTTGGACA

ATTCAAAAGTTGGAGAAGGTTTTTTACTAATAAATCGATATTCAAAATCATTCCATTCAG

TATCTTTTACTCTATCAAAGCGTCGGATTTCTAAATTCTCAAAGGGCCCCCCTGGAATTC

CTTCCAGAGCCTGTTGGATAATTTTTATGGATTCTGTCATTTCACTGATTCGTACTAAAT

AACGAGCTAATGAATCCCCTTCTTTTTGCCATTGAACCTCCCAATCAAATTCGTCGTAAC

ACTCATAATGATCAACTTTACGAAGGTCCCATTGTATTCCGGAAGCTCGTAGCATTGGTC

CTGATAAACCCCAATTTAGTGCTTCTTCTCCTCCAATAATGCCTACGCCCTCAACTCGTT

CTAAAAAAATAGGATTCCGTGTAATAAGCTTTTGATATTCAGCAACCCCTGTTAAAAAAT

AATCACAAAAATCCAAACATTTATCTATCCATCCATGAGGTAGATCAGCAGCTATTCCTC

CGATACGAAAATAATTATGCATCATTCGCATACCGGTGGCAGCTTCGAATAGGTCATATA

TCAATTCTCGTTCTCGAAAAATATAGAAGAAAGGGGTCTGTGCCCCAATATCTGCCATAA

AAGGACCAAGCCATAACAAATGAGAAGCTATACGACTCAACTCCAACATAATGGCTCTGA

TATAGCTAGCCCTTTTAGGTACTTGAATATTGCCTAGTTGTTCGGGTCCATTTACGGTTA

TTGCTTCTGTGAACATAGTAGCTAAATAATCCCAACGTGTTACATAAGGCAAATATTGTA

TAATTGTTCGGTTTTCCGCAATTTTCTCCATTCCTCTGTGTAAATAACCCAATATTGGTT

CACAGTCAATAACATCTTCACCATCGAGAGTAACGATAAGTCGAAGAACACCATGCATTG

ATGGGTGGTGAGGACCCATATTGACTATCATGAGGTCTTTTCTTGTAGTTGGTGCAATCA

TAAGTTTTTTACCGAGTCATTCTTCCATGAATTGCTGAAAGTAAAAAGAAGTTCATCAAA

ATTGAAACGCATAAGTTCAAATGATATCACTCTTTAAATTAACGAGTTTTTGTCTCTCGA

ATATCCAACCGATCAATTAATTCTTTATAACGTACTCTATTTTTTTTTGACAAATAAGCC

AGTAATCGTTGACGTTTTCCCAAAATTTTTCGCAAACCTCTCTGAGATGAATAGTCTTTT

TTGTGCAATTCCAAATGTGAAGTAAGTCTCCTTATCTTAGTGGTGAAACTGAATACTTGA

AATTCAACAGACCCTCTGTTTTCTTCATTTTCTTTTTGAGAAATAACCGAAATGAATGAA

TTTCTTACCATAAAAAAAAGCCTCCTCTCCCTTTTTACAGATATGGATTTTACCGATCAG

TAATAATAATGTCATTCATTTTAATGTGGTATATACAATAATCTAATTCAAATTTCTTTA

TGAACTCCTAATTTTATCAATTCAAATGAATCAATCCAATTGAAAATTAGATTTAGATAG

GGAGAGAAAAGGATTGGCACATTTTCGTATTCACAAAAGCGAAGAATTTAGACCTAAAAT

GAAGAGGGTTCTGTTGATTCATTTCTAGATCGAATTGATACATTATTCATTTAGTATTGG

ATATTTAGAATGAAATGTAAGACAGGACGTGTGATGTGTGTATTTATTTGCTTTCATATA

TCCTATATAGTAGAGGATATATAGGAAAAATGGACTATCAACGAATTTTCAATCGTGGAT

ACAAATGTATCCTTAACATACTGAAACGACTGCCATTATTGGTATCAAACCAATAGCGAT

TCATACAAGCTAAATCTTCTAATCGATAATTAGGCCAAAGAAAGAACTTCAATTTCATTA

ATTGATTTGTCTCTCTATCAAGGTGATTACTGTCACCTAGAACTTGGCTGCTATTCTTTT

CGTTGCAAAATACAGGATTTCCATCTACATCATTCCTATTCTTTGAATTGAAAGAAATTA

GAATTCTTAATTTTCTACGACGCCTAAATGAGAAAATATTTTCAGGAACAAGCAAATCCA

AATGATTTTTGTCTCTATTTCTTGTTATTCTTTCATGTGGTGGAATAGATTCATCAAAAT

TATTCTTAGCAACATATCTTTGCTCTCGGTATCTTTGATTAGTTTGGTGCTTACTCTTAT

GAACCAACAAAATACCGATGGTTTGATACATAATAAACTGTCCGTCGTTTTTTACAGACA

GACGAATGGGTTCGATAATAAATATTCCCTTTTTCATCAATTCTGGAAGAGTTAAATTCT

TCTGAATCAGCATTATATCCAAACTCATTTCTCCCCTTTGAATCGAGGATATAGAAATTT

TTCTTGGATCTATTAGTCTAAGCAGGAAACAATATACCTTAATATTATTGATCAGTCTTT

GATTCAAAGTATCGTCCCATCTCAATTGAAAAAGCAAATAACGTTTCAGGAACAAATCTA

GTTCTGCTTCCTTACTTTTGTATTGTTTTTTCTTTTTACCTTTTTTCATGTCCGATCTCG

CATAATCTTCTTCAATATCTTTTTGTTGGTTTGAAAGAACGGATCCAAGATCCCCTTGGC

TTGTGGTTTTTTTTTCTTCTTGATTTCGATTCTTTATTTTTTTATTCGATGGTATCAAAA

AACTTCCCTTTTGCTTTTCATTAATCTTTTGATTTTGATTACTATTTTCATTTCTATTCA

AATTTAAAAGAAGTAATTTGCTTGGTATAAACCAAGATTTCGTTTTATATGTATTATAAA

GTAACAAAAATTCTGGGAAGAACCAAAGTTCCAGATTCAATATGGGACGCTTTAGTATTT

TTTCATTCATCCCCATCCAATCAAAAAAGACTTTTGGGGAGTTTGGTAAATTCATTTCTG

GAATCATAAGATAAAAAATATTTTTTTTATCAATTATTTGATAATTATTAGTAACAATCT

GAGTATTTTGATTACTATTGGCATCGATCGTGATCCAGGCCTCAATATCGACTTTTTGTC

TAAGATCAAAATTGATAATTTTCCAATCCAAATATTTCCTATCGGGAGTTTTTTCCATAT

ATAGAATATCGCCCTTTCCTAGATAATTATTGATAGGGATACTCCTCAGCATATCAAAAA

AAGTGTCTTTATATGTGTTGTAATTATAAGAAATCTCTTGATTCTTATTTCCTTCAAACG

GCGATCTAGAAATAAACGAGTCCTTTTTATTTTCAGAATTAATAGATTTATATGATAAAA

GATCATATTTATAGTATTTTGGAAAATTATCTTTTTGATTCAATAATGAATAGACTTCAA

AAATATTTTCTTTTTTGTAATCAATTAATTGGTCTTTTTCATATAAATTCCATTTGCTGA

AATTTTTCCTTTTAACCATACGACGTTGATAAACTCTATTCCGCCATTGTTGTGGTATTA

ATCTAGACCATCTGATCTGAGATAAATCGTATTGATAATGCCCTCTTAACCAGTTTTTCC

ATTGATTCATTTCAGAACTCGGAAGTCTGTTATCCCTTAATTTAGAATGAACTATTCCTT

GTGTTTCAAAAGAATCCTTTATTTCAGGCTTAAGAAAAAAAGGGATTCCTTGATATTGAA

ATGATTTTAATTTATACAAGTTAATAACTTGGGTTTGTGATAATTTGTAAAATACATATG

CTTGTGATAAGTACGATAAGTCATAAAAAATATGTGAATTTGTCTGACTATTACTAATAT

TATAAAGTGACTTTTTTATAGTCGAAATAAACTCAATTGGATTTTTATTTTTTTTATTAA

TTATTTCTTGACTTGTTTCATTATTGTAAATGGATTTATTCATAATTTTTTTTGTTGATT

CAAGAAATAATTTTCTCTTTATTCTGGGAATATTAATGATAGATAAAAATATATTTGTGT

AGATTCTTTCAATTAAAAATTTAAAAACAAAAGGTAATTTAGAGATTAATCGAGCATTTC

TTCTTTTTAATATTTGCCATTTTTCTAATCTTTTAGCATTATAACTTGTTTTGTTAAGAC

TAATATTTATTTCTGGAGTTACTTTTTTTTTCTCTTTTGTAATTCTTTCTATTTGATTTC

TAATTGTATTTGTTCTATCAGTCAGATCCTTCATTTTTTTTTCTGTCAATGAAGAATTTG

TCCAACCTGGAGATGCAATTTGACTAAATGACTCATGAATCATCTGATTGCTGATTATGG

GATCTCTTTCTTTTTTAGTTTCACTCGAGTCATATACTTCTACTTCTCTTGATCGAAATA

AGAGAATTGGATTCACTTTTAAGAGTTCTTTTCTTATTTTTTTAAAAAAAACGACACTTT

TGATAACCCATTTTTTTGTTTCTTTTGAAACTTTTCGAAGTAATTTTTTTTTTCCTTTTA

AAATTTTTAGAAGTAGAAAATATTTCTTTTTTAATTTTACAATTTTTTTTTCGAGTTCCT

TAAAAATGGGTTCAAAAAAAGAGGGTCGTTTTCGGGGAGAACCAAAAGGAAGTTCGGTTT

CCATTCCCAAAACTGTTAAAAAACAAAAATCATCTTTTTCTTTTTTCTTTTTCATTATTA

GATCTTTATGAGATAATCGGAGTTTAGATCTGTGCCAAGGTTTCAGACAGAAAGGAAATA

AGATTTTTATCTGAATACCATCTGTCAACCAATTTTGTGGAAATTCTGTTTCGGACAATT

GAACACCATTATAGGTACATTTAACATGCATCTCCCTATTCCATTCCTCTAAATCCTCAT

ACCATTCAGGAAGTTGCAATAGTAGCATACGTCCAATATTTTTTATTATCAATGAAGGTA

ATATAATATATTTTCTAAAAATAGATTGAGTTATTAACATGGAACCCCTTATTACTTGCG

CAAATGGAATGGTATCCCAGGTCTCTGCTATTATTCGCGCGATCTCCTTTCTTTTGTTCT

CTTTTTTTTCTTCTCTTTTGTTTGCTCTTCTGTATACTCTACAATTTTGAATGCTGACCC

CCTCCCTACCCAATTTCTAAAAATTTTAATTGGCCCAGAGATATCAAAAGAAAAAAGAAG

GGATTTTTTTATTCTGTCCAAAAAAAGAGGGGAATGCACATTTGCTTGAAACAGTTCCCA

AATAACTATTTTACGCCTTTGAGCACGTATAGAACCTTTTATTATGCCTCGCCTAAAATC

TGATTGTTGTGAATAGCGTAGCAAAGCTACTTCGTATGTTTGATCAGGAGGATTAGTATC

CTCAGTATGCTCCTTGTTACCAGTAAAAATTACTACACGTTTGGCTTTTCTTGAACGAAT

TTCATGATCTAATGGTCCGTTTTCTTTATCTTCTCCTGCTTCGTGTTCCAACTCGCTGGT

TAATTTGTATAACCAGCGAGGTACTTTTTTACTGATTTCTTTTATTCTAATCGATTTTTT

ACTAATTTTTTGAACATTAGTATCAGTTAGAATTCTATTTTTAAAATATTTCAAATATTT

TGTTCCTTTTTCTGAATCTATTCTTCCTTCTTCTTCATCTGAAAATAAAGAAAGACCCCT

AGAATTAAAATAGATCCTGATTCTTTACCAAGTTCATTGATGAAAGTTAAGAAATCAACA

ATTTCGGTTGATAATGGTTTTTTATCAAATCGATCTATTTTCTGTTCAATTTTTTGGTAA

TCAGTATCAGGAAGAACGATACCATGAATACGATTTATCCCAACTTTCTCTATGAAATTG

TCTATCGAAGTTTTTTTTATTTTTATGATTGAAGGTGAAACGCTTTTTGTTATTGTTCTC

CGATATGGTCCGTTCAAGAAAGGATCATATATTTTGGGTAAGTATTCGTTTGTAGTATTG

TCATTACACAACCTAGTCCTTGTTTCGAGTATATTCAAAAAAAGAGATTCTTTGTCTAGG

GCTTGAATTCGATTTACAAATTCGTTGTTTAAATTATTACTTTTTTCTTTGTTGGTATAA
[truncated: 3,335,794 more chars]
