# Supplementary material for: Plastid genomes reveal evolutionary shifts in elevational range and flowering time of Osmanthus (Oleaceae)
Source: Ecol Evol. 2022 Apr 1;12(4):e8777. doi: 10.1002/ece3.8777 (PMC8975774; doi:10.1002/ece3.8777)
Supplement: Supplementary file 3 — Supplementary Material [file ECE3-12-e8777-s008.docx]

**Supplementary file 1** Best-fitting models and parameters for the whole-length plastome phylogenetic analyses

| Analysis | Alignment length  (bp) | parsimony-informative sites | Best-fit model (AICc) | -LnL | df | Base frequencies | | |  | Rate parameters | | | | | |
| --- | --- | --- | --- | --- | --- | --- | --- | --- | --- | --- | --- | --- | --- | --- | --- |
|  |  |  |  |  |  | A | C | G | T | AC | AG | AT | CG | CT | GT |
| BI | 129095 | 768 | GTR+F+I+G4 | -197216.081 | 63 | 0.31 | 0.19 | 0.18 | 0.32 | 1.00 | 0.85 | 0.23 | 0.23 | 0.86 | 1.00 |
| ML | 129095 | 768 | TVM+F+R2 | -197799.383 | 63 | 0.31 | 0.19 | 0.18 | 0.32 | 1.00 | 0.85 | 0.23 | 0.23 | 0.86 | 1.00 |
