# Supplementary material for: Plastid genomes reveal evolutionary shifts in elevational range and flowering time of Osmanthus (Oleaceae)
Source: Ecol Evol. 2022 Apr 1;12(4):e8777. doi: 10.1002/ece3.8777 (PMC8975774; doi:10.1002/ece3.8777)
Supplement: Supplementary file 5 — Supplementary Material [file ECE3-12-e8777-s005.docx]

**Supplementary file 5** Trait data for reconstruction of ancestral state

| Species | Accessions number | Groups | character | |  |
| --- | --- | --- | --- | --- | --- |
|  |  |  | Mean elevation(m) | Flowering time | |
| *Osmanthus armatus* | MW648824 | *Core Osmanthus* | 500 | Autumn | |
| *Osmanthus cooperi* | MW727458 | *Core Osmanthus* | 500 | Autumn | |
| *Osmanthus fordii* | MW727462 | *Core Osmanthus* | 500 | Autumn | |
| *Osmanthus fragrans* | MT182984 | *Core Osmanthus* | 500 | Autumn | |
| *Osmanthus enervius* | MW727461 | *Core Osmanthus* | 500 | Autumn | |
| *Osmanthus insularis* | NC_042264 | *Core Osmanthus* | 500 | Autumn | |
| *Osmanthus didymopetalus* | MT182989 | *Core Osmanthus* | 500 | Autumn | |
| *Osmanthus fortunei* | MW727463 | *Core Osmanthus* | 500 | Autumn | |
| *Osmanthus heterophyllus* | MW727464 | *Core Osmanthus* | 500 | Autumn | |
| *Osmanthus urceolatus* | MH229859 | *Core Osmanthus* | 500 | Autumn | |
| *Osmanthus serrulatus* | MW727466 | ***O. serrulatus + O. yunnanensis*** | 1500 | Spring | |
| *Osmanthus yunnanensis* | MW727465 | ***O. serrulatus + O. yunnanensis*** | 1500 | Spring | |
| *Osmanthus delavayi* | MW727460 | *Siphosmanthus* | 1500 | Spring | |
| *Osmanthus suavis* | MW727467 | *Siphosmanthus* | 1500 | Spring | |
| *Osmanthus decorus* | MW727459 | *Caucasian Osmanthus* | 500 | Spring | |
| *Notelaea longifolia* | NC_042458 | *Outgroups* | 500 | Spring | |
| *Notelaea microcarpa* | NC_042459 | *Outgroups* | 500 | Spring | |
| *Notelaea venosa* | NC_042427 | *Outgroups* | 500 | Spring | |
| *Nestegis lanceolata* | NC_042456 | *Outgroups* | 500 | Spring | |
| *Nestegis apetala* | NC_036983 | *Outgroups* | 500 | Autumn | |
| *Nestegis cunninghamii* | NC_042455 | *Outgroups* | 500 | Autumn | |
| *Nestegis sandwicensis* | NC_042457 | *Outgroups* | 500 | Spring | |
| *Phillyrea angustifolia* | NC_042464 | *Outgroups* | 500 | Spring | |
| *Picconia azorica* | NC_042428 | *Outgroups* | 500 | Spring | |
| *Picconia excelsa* | NC_042466 | *Outgroups* | 500 | Spring | |
| *Phillyrea latifolia* | NC_042465 | *Outgroups* | 500 | Spring | |
| *Osmanthus austrocaledonicus* | MK299397 | *Outgroups* | 500 | Spring | |
| *Olea europaea* | MT182986 | *Outgroups* | 500 | Spring | |
|  |  |  |  |  | |
