# Supplementary figures and images for: Plastid genomes reveal evolutionary shifts in elevational range and flowering time of Osmanthus (Oleaceae)
Source: Ecol Evol. 2022 Apr 1;12(4):e8777. doi: 10.1002/ece3.8777 (PMC8975774; doi:10.1002/ece3.8777)

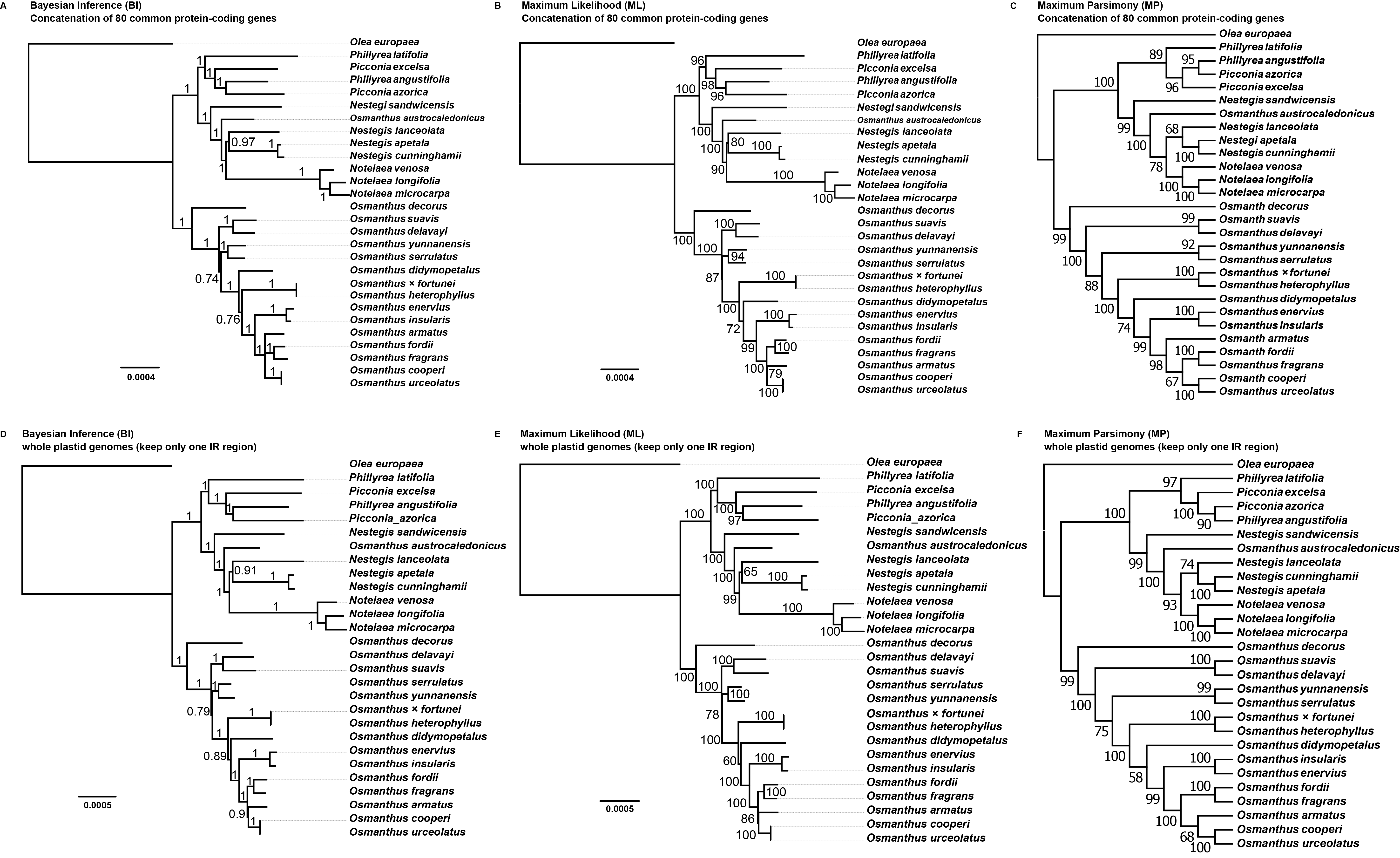

Supplement: Supplementary file 6 — Supplementary Material [file ECE3-12-e8777-s007.png]

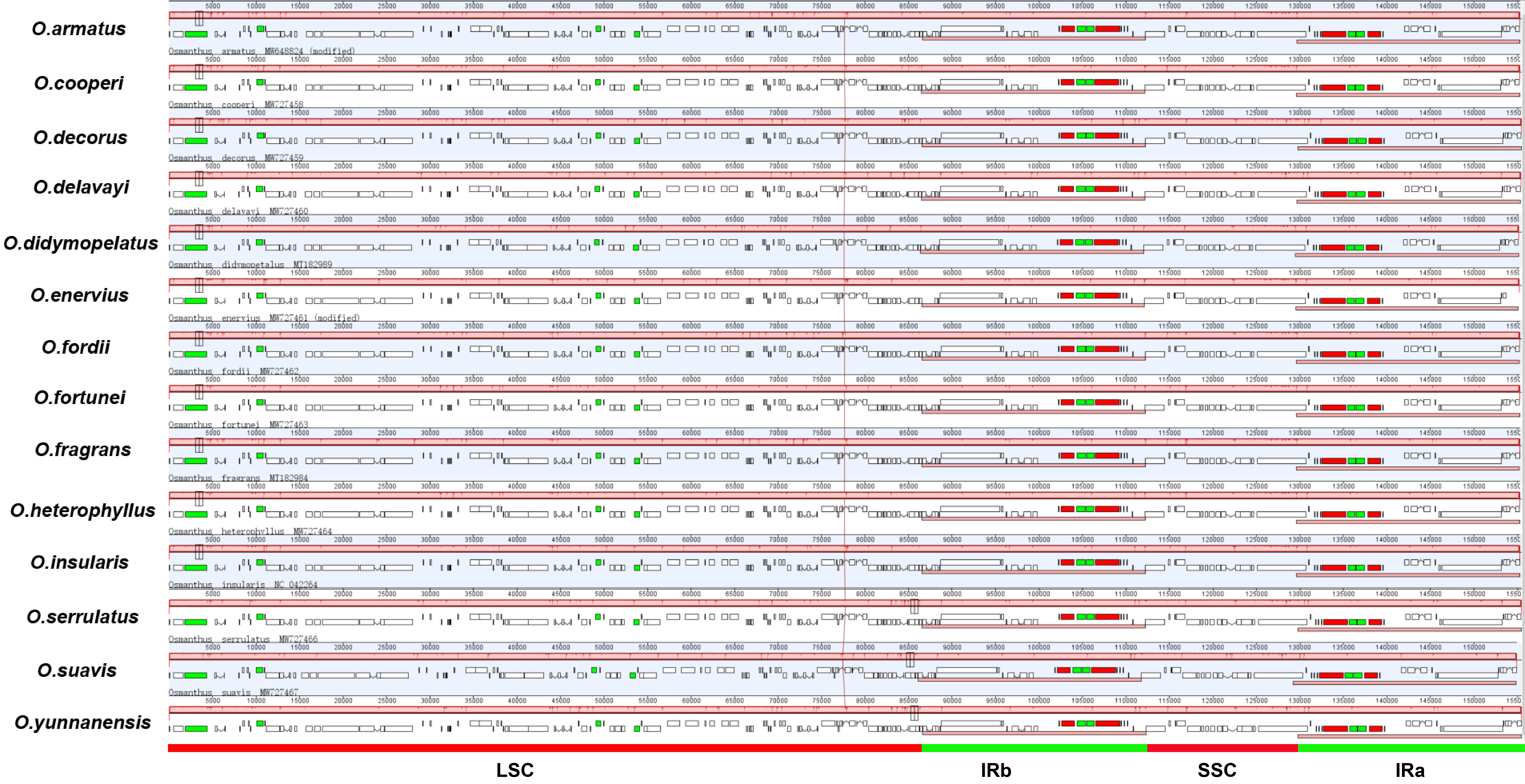

Supplement: Supplementary file 8 — Supplementary Material [file ECE3-12-e8777-s009.png]
