# Supplementary material for: Plastid genomes reveal evolutionary shifts in elevational range and flowering time of Osmanthus (Oleaceae)
Source: Ecol Evol. 2022 Apr 1;12(4):e8777. doi: 10.1002/ece3.8777 (PMC8975774; doi:10.1002/ece3.8777)

**Supplementary file 7** The results of molecular clock

| Nodelabel | Posteriormean(100Myr) | (95%Equal-tailCI) | (95%HPDCI) | HPD-CI-width |
| --- | --- | --- | --- | --- |
| 29 | 0.3233 | (0.2801,0.3696) | (0.2794,0.3688) | 0.0894 |
| 30 | 0.1583 | (0.1218,0.1900) | (0.1223,0.1904) | 0.0681 |
| 31 | 0.1266 | (0.0907,0.1635) | (0.0900,0.1626) | 0.0725 |
| 32 | 0.083 | (0.0574,0.1143) | (0.0557,0.1116) | 0.0559 |
| 33 | 0.0802 | (0.0553,0.1104) | (0.0545,0.1090) | 0.0545 |
| 34 | 0.062 | (0.0420,0.0877) | (0.0397,0.0847) | 0.0449 |
| 35 | 0.0572 | (0.0387,0.0815) | (0.0372,0.0794) | 0.0422 |
| 36 | 0.0447 | (0.0293,0.0653) | (0.0278,0.0629) | 0.0351 |
| 37 | 0.0301 | (0.0187,0.0457) | (0.0176,0.0439) | 0.0263 |
| 38 | 0.0277 | (0.0164,0.0428) | (0.0155,0.0413) | 0.0258 |
| 39 | 0.0005 | (0.0000,0.0022) | (0.0000,0.0017) | 0.0017 |
| 40 | 0.0196 | (0.0103,0.0325) | (0.0094,0.0309) | 0.0216 |
| 41 | 0.0108 | (0.0047,0.0208) | (0.0039,0.0191) | 0.0152 |
| 42 | 0.0005 | (0.0000,0.0021) | (0.0000,0.0016) | 0.0016 |
| 43 | 0.0489 | (0.0202,0.0849) | (0.0187,0.0823) | 0.0636 |
| 44 | 0.0585 | (0.0334,0.0887) | (0.0321,0.0869) | 0.0548 |
| 45 | 0.1338 | (0.1011,0.1658) | (0.1007,0.1654) | 0.0646 |
| 46 | 0.1163 | (0.0858,0.1479) | (0.0854,0.1473) | 0.0619 |
| 47 | 0.1019 | (0.0735,0.1320) | (0.0731,0.1315) | 0.0584 |
| 48 | 0.0938 | (0.0666,0.1232) | (0.0667,0.1233) | 0.0566 |
| 49 | 0.0862 | (0.0592,0.1153) | (0.0587,0.1147) | 0.056 |
| 50 | 0.0103 | (0.0042,0.0206) | (0.0036,0.0189) | 0.0153 |
| 51 | 0.0376 | (0.0226,0.0577) | (0.0213,0.0553) | 0.0341 |
| 52 | 0.0256 | (0.0138,0.0422) | (0.0124,0.0403) | 0.0279 |
| 53 | 0.1206 | (0.0887,0.1526) | (0.0894,0.1531) | 0.0637 |
| 54 | 0.0944 | (0.0644,0.1265) | (0.0655,0.1274) | 0.062 |
| 55 | 0.0804 | (0.0512,0.1120) | (0.0509,0.1116) | 0.0606 |


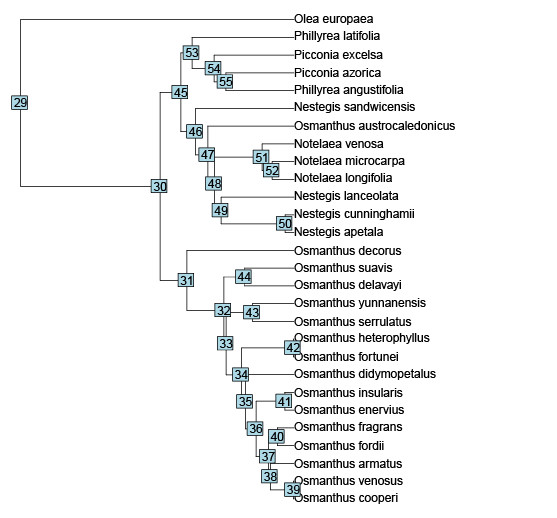

Supplement: Supplementary file 7 — Supplementary Material [file ECE3-12-e8777-s006.docx]
